# *CDX2* expression in the hematopoietic lineage promotes leukemogenesis via TGFβ inhibition

**DOI:** 10.1002/1878-0261.12982

**Published:** 2021-06-26

**Authors:** Ava Galland, Victor Gourain, Karima Habbas, Yonca Güler, Elisabeth Martin, Claudine Ebel, Manuela Tavian, Laurent Vallat, Marie‐Pierre Chenard, Laurent Mauvieux, Jean‐Noël Freund, Isabelle Duluc, Claire Domon‐Dell

**Affiliations:** ^1^ Université de Strasbourg Inserm, IRFAC / UMR‐S1113, FHU ARRIMAGE, ITI InnoVec, FMTS Strasbourg France; ^2^ Institute of Biological and Chemical Systems Karlsruhe Institute of Technology Germany; ^3^ Inserm IGBMC UMR‐S 1258 Université de Strasbourg Illkirch France; ^4^ Laboratoire d’Hématologie Centre Hospitalier Universitaire de Strasbourg France; ^5^ Département de Pathologie Centre Hospitalier Universitaire de Strasbourg France

**Keywords:** acute monoblastic leukemia, BAMBI, ectopic expression, oncogene, tumor suppressor

## Abstract

The intestine‐specific caudal‐related homeobox gene‐2 (*CDX2*) homeobox gene, while being a tumor suppressor in the gut, is ectopically expressed in a large proportion of acute leukemia and is associated with poor prognosis. Here, we report that turning on human *CDX2* expression in the hematopoietic lineage of mice induces acute monoblastic leukemia, characterized by the decrease in erythroid and lymphoid cells at the benefit of immature monocytic and granulocytic cells. One of the highly stimulated genes in leukemic bone marrow cells was BMP and activin membrane‐bound inhibitor (*Bambi*), an inhibitor of transforming growth factor‐β (TGF‐β) signaling. The CDX2 protein was shown to bind to and activate the transcription of the human *BAMBI* promoter. Moreover, in a leukemic cell line established from CDX2‐expressing mice, reducing the levels of *CDX2* or *Bambi* stimulated the TGF‐β‐dependent expression of *Cd11b*, a marker of monocyte maturation. Taken together, this work demonstrates the strong oncogenic potential of the homeobox gene *CDX2* in the hematopoietic lineage, in contrast with its physiological tumor suppressor activity exerted in the gut. It also reveals, through BAMBI and TGF‐β signaling, the involvement of CDX2 in the perturbation of the interactions between leukemia cells and their microenvironment.

## Graphical abstract

Abnormal expression of the homeodomain transcription factor caudal‐related homeobox gene‐2 (CDX2) is associated with poor prognosis of leukemia patients. Here, we report that turning on human *CDX2* expression in the hematopoietic lineage of mice induces acute monoblastic leukemia, through inhibition of the lymphocytic lineage and accumulation of immature monoblasts mediated by the stimulation of the BMP and activin membrane‐bound inhibitor that blocked transforming growth factor‐β‐dependent differentiation of monocytes.

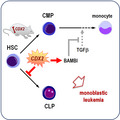

**Abbreviations**
CDX2: caudal‐related homeobox gene‐2
BAMBI: BMP and activin membrane‐bound inhibitor
TGF‐β: transforming growth factor‐β

## 1 Introduction

Major developmental genes, such as caudal‐related homeobox gene‐2 (*CDX2*) [1], have emerged beyond ontogenesis as critical players in cancer. This homeobox gene has multiple functions during embryonic development including trophectoderm formation, elongation, and patterning of the posterior body, and intestinal specification, before being selectively expressed in the gut epithelium throughout adulthood where it exerts a tumor suppressor role [2, 3, 4, 5, 6]. Conversely, it is ectopically turned on in precancerous metaplastic lesions of the foregut [1] and, beyond the digestive tract, in a high proportion of acute leukemia associated with poor prognosis (see Ref [7] and references therein). Cellular studies have shown that *Cdx2* confers oncogenic properties to murine hematopoietic stem cells *in vitro* [8, 9], while turning on its expression *in vivo* induces myelodysplastic lesions, a few of them evolving into overt leukemia [10]. However, the mechanism(s) associated with this pro‐oncogenic activity remain largely elusive. Here, we developed a mouse model of conditional ectopic expression of human CDX2 in the hematopoietic lineage. This led to acute monoblastic‐type leukemia involving the alteration of transforming growth factor‐β (TGF‐β) signaling. Interestingly, TGF‐β is an important pathway in hematopoiesis, which is frequently altered in leukemogenesis [11]. Indeed, it negatively regulates cell proliferation and stimulates differentiation and apoptosis during normal hematopoiesis, whereas these effects are often impaired in hematologic malignancies due to deletions/mutations of members of the pathway or to the deregulation of cofactors by oncoproteins [12].

## 2 Materials and methods

### 2.1 Mice

*RsCDX2* knockin mice having the human *CDX2* coding sequence preceded by a loxP‐excisable transcriptional stop sequence inserted into the *Rosa26* locus [13], transgenic *Mx1Cre* mice containing the *Cre* coding sequence placed downstream of the promoter of the interferon‐inducible gene *Mx1* [14] (Jackson Laboratory) and immunodeficient NSG mice (Charles River) were housed in the certified animal facility (#H‐67‐482‐21). Protocols were approved by the French Ministry of Agriculture under the permit APAFiS#833. Mice were genotyped by PCR using primers listed in Table S1. Three‐month‐old *RsCDX2::Mx1Cre* mice (designated as *MxCDX2*) and control littermates received 3 intraperitoneal injections of poly(I:C) (Sigma‐Aldrich) at 10 mg kg^−1^ of body weight every 2 days. For transplantation, 10^5^ bone marrow cells of *MxCDX2* or control mice were injected into the caudal vein of NSG mice.

### 2.2 Plasmids, siRNA, and luciferase assays

Plasmids pFlag (pFlag‐CMV2; Sigma‐Aldrich, Darmstadt, Germany), pFlag‐CDX2 [15], pBAMBI‐Luc [16], and pRL‐null (Promega Inc., Charbonnières les Bains, France) have been described. siRNAs are listed in Table S1. Luciferase assays were performed using the Dual Reporter Luciferase Assay (Promega Inc.).

### 2.3 Cell line establishment, cell culture, transfection, and TGF‐β treatment

Femoral bone marrow cells of *MxCDX2* mice were plated at 2 x 10^6^ cells per well in DMEM supplemented with 20% FBS, 14 ng·mL^−1^ mIL3, 24 ng·mL^−1^ mIL6, 112 ng·mL^−1^ mSCF (Promokines, Camon, France), and antibiotics for 1 week. The cell line AGK463 was established by progressive starvation of the three cytokines and reduction in the serum to 10%. Transfections used 10^6^ AGK463 cells, 50 nm siRNA alone, or 40 mm of these siRNAs with 10 nm siGLO RISK‐free Control (Horizon Perkin‐Helmer, Waterbeach, UK) and Viromer® BLUE Kit (Lipocalix, Halle, Sachsen‐Anhalt, Germany). TGF‐β treatment (20 ng·mL^−1^) was performed 24 h after transfection during 48 h before analyses.

Human K562 myeloid leukemia cells were cultured as described [17]. For RNA analyses, 10^6^ cells were nucleofected with 1.5 µg pFlag or pFlag‐CDX2 following the Amaxa protocol [18]. For luciferase assays, 1.5 × 10^5^ cells were transfected using Lipofectamine^TM^ 3000 (Invitrogen, Carlsbad, CA, USA). Analyses were performed 48 h later.

### 2.4 Serial replating assays

Femoral bone marrow cells of *MxCDX2* mice and control littermates were plated at 2 × 10^4^ cells per well in 1.1 mL methylcellulose (MethoCult GF M3434; Stem Cell Technologies, Grenoble, France) and cultured for 10 days. Colonies were counted, and cells were serially replated under the same conditions.

### 2.5 Histology, cytology, and flow cytometry

Bones were decalcified with formic acid. Histology and immunohistochemistry with anti‐CDX2 antibody (EPR2764Y; Abcam, Paris, France) were performed as described [4]. Cytotyping of bone marrow cells used May Grünwald–Giemsa staining (MGG).

Flow cytometry was performed using a Fortessa cytometer (BD Biosciences, Le Pont de Claix Cedex, France) with antibodies listed in Table S2.

### 2.6 RNA preparation, RT‐qPCR, RNA sequencing, and data analyses

RNA preparations were performed using TRI Reagent (Euromedex, Souffelweyersheim, France). The quality of total RNA was assessed on RNAchip with a Bioanalyzer (Agilent).

For RT‐qPCR experiments, RNA was extracted from the indicated cell lines and analyzed with the probes listed in Table S1.

For RNAseq experiments, RNA was extracted from the bone marrow of 6 littermate mice: 3 *RsCDX2::Mx1Cre* mice and as control 1 *RsCDX2*, 1 *Mx1Cre*, and 1 wild‐type mouse. All 6 mice were treated with poly(I:C), and RNA was prepared 6 weeks later. RNAseq data analyses were performed as described [13] using STAR (v2.5) [19] for sequence alignment against the reference mouse genome GRCm38.90, and HTSeq (v0.6.1) [20] and DESeq2 (v1.10.1) [21] for reads counting and normalization. Differentially expressed genes were selected based on |log_2_(fold change)| > 1 and *P*‐value < 0.05, corrected with the false discovery rate (FDR) multiple testing method. Gene Ontology enrichment was done with the one‐tailed exact Fisher's test, and *P*‐values were corrected with FDR multiple testing. Functional clustering used DAVID [22].

Transcription factor‐binding motif analysis used HOMER (v4.10.1) [23] in the promoter gene regions extending from −2000 bp upstream to +50 bp with respect to the transcription starting site(s). Enriched motifs were manually curated by comparing with the most up‐to‐date version of JASPAR [24] and TomTom from the MEME suite [25].

For point mutation analysis, RNAseq reads were mapped against the mouse reference genome GRCm38.90 with the gap‐aware aligner STAR as described above. Multimapped reads were not output, and alignments at splicing junctions were refined with a second pass based on the annotation of the reference genome. Duplicated reads were then flagged with MarkDuplicates from the Picard toolbox (http://broadinstitute.github.io/picard/). The remaining misaligned reads were removed with the tool SplitNCigarReads from the toolkit GATK (v4.0.9.0) [26]. single nucleotide polymorphisms (SNPs) were called with the tool HaplotypeCaller from GATK [27]. Soft‐clipped bases were discarded during SNP calling, and the threshold for emitting the SNPs was set to 20 (Phred scale). SNPs were filtered based on their quality with the tool VariantFiltration from GATK with default parameters. SNPs were finally annotated with VEP from Ensembl [28]. Recurrent mutations were defined as SNPs present in all three *MxCDX2* mice but in none of the three control littermates. Mutational signatures were tested with SIGNAL (https://signal.mutationalsignatures.com/) in comparison with published models of myeloid leukemia [29] and DNA repair mechanisms [30].

### 2.7 Western blots

Whole bone marrow or AGK463 protein extracts separated on SDS/PAGE were analyzed by western blot using mouse monoclonal anti‐CDX2 (CDX2‐88, BioGenex, Fremont, CA, USA; dilution 1/2000) and mouse monoclonal anti‐β‐actin (C4, Millipore, Dachstein, France; dilution 1/25 000) antibodies. HRP‐conjugated secondary anti‐mouse antibody was used for ECL detection (GE Company, Amersham, UK).

### 2.8 Chromatin immunoprecipitation

ChIP assays were performed using the Magna ChIP™ G Kit (Merck‐Millipore, Dachstein, France) and anti‐CDX2 antibody (EPR2764Y; Abcam) or IgG. qPCR amplification of the immunoprecipitated material used the primers listed in Table S1.

### 2.9 Statistics

Statistics used the two‐tailed *t*‐test for mean comparisons and log‐rank test to compare the survival times between *MxCDX2* or engrafted NSG and control mice (graphpad, Prism, https://www.graphpad.com/scientific‐software/prism/).

## 3 Results and Discussion

### 3.1 Oncogenic activity of CDX2 in the hematopoietic lineage *in vivo*

**Fig. 1:**
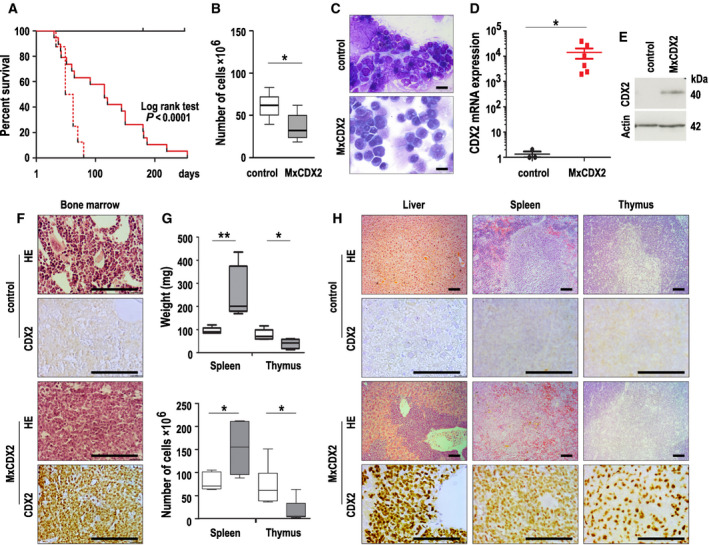
Leukemia development by ectopic expression of *CDX2* in the hematopoietic lineage. (A) Overall survival (in days) of control (*n* = 13, black line) and *MxCDX2* mice (*n* = 18, red line) after poly(I:C) injections, and of *NSG* mice after transplantation of bone marrow cells from *MxCDX2* mice (*n* = 8, red dotted line). (B) Bone marrow cellularity of control (*n* = 3) and *MxCDX2* mice (*n* = 3). Boxes extend from 25 to 75th percentile, and whiskers represent mean to max. *P < 0.05. (C) Representative cytology (MGG staining) of smears of bone marrow cells of control and *MxCDX2* mice. Bars: 25 µm. (D) Relative *CDX2* mRNA expression analyzed by RT‐qPCR in the bone marrow of control (*n* = 3) and *MxCDX2* mice (*n* = 6). Data are given means with SEM. *P < 0.02. (E) Representative western blot of CDX2 protein in the bone marrow of control and *MxCDX2* mice. (F) Histology (HE) and immunohistochemical staining of the CDX2 protein in the bone marrow of control and *MxCDX2* mice. Bars: 100 µm. (G) Weight (mg) and cellularity of the thymus and spleen of control (*n* = 3, open boxes) and *MxCDX2* mice (*n* = 3, gray boxes). Boxes extend from 25th to 75th percentile, and whiskers represent mean to max. *P < 0.05; **P < 0.01. (H) Histology (HE) and immunohistochemical staining of the CDX2 protein in the liver, spleen, and thymus of control and *MxCDX2* mice. Bars: 100 µm.

To address the oncogenic potential of ectopic expression of the human CDX2 homeoprotein in the hematopoietic lineage, *MxCDX2* mice were generated by intercrossing *RsCDX2* [13] and *Mx1Cre* [14] mice, and the resulting adult animals aged 2–3 months were treated with poly(I:C) to induce CDX2 expression. All the treated *MxCDX2* mice (*n* = 18) became moribund with a median survival time of 115 days, in contrast to treated control *RsCDX2* or *Mx1Cre* mice (*n* = 13; Fig. 1A). Comparing *MxCDX2* mice to controls 6 weeks after poly(I:C) administration revealed a pale bone marrow with reduced cellularity (Fig. 1B) and a monomorphic aspect (Fig. 1C) characterized by the accumulation of blasts expressing the *CDX2* mRNA (Fig. 1D) and protein visualized by western blot and immunohistochemistry (Fig. 1E,F). In addition, CDX2‐positive blasts invaded several organs leading to hepatomegaly and splenomegaly and in most cases thymus atrophy (Fig. 1G,H).

**Fig. 2:**
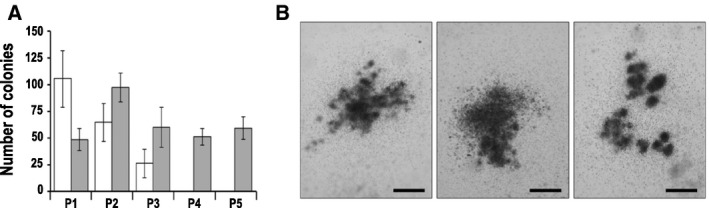
*Ex vivo* replating activity of *MxCDX2* bone marrow cells. (A) Number of colonies grown *ex vivo* from bone marrow cells of control (white boxes) and *MxCDX2* mice (gray boxes) during five consecutive passages (P1–P5). Data are given means with SEM. *n* = 4. (B) Representative pictures of colonies grown from *MxCDX2* bone marrow cells at the 5th replating step. Bars: 500 µm.

The properties of bone marrow cells were first investigated using the *ex vivo* replating assay. As shown in Fig. 2A, bone marrow cells of control mice (*n* = 4) loosed replating potential at the 3rd step. On the contrary, cells of *MxCDX2* mice (*n* = 4), while producing twice less colonies than controls at the 1st plating step in line with the reduced bone marrow cellularity, maintained their growing activity throughout the five successive plating steps of this study (Fig. 2A,B). The second line of characterization of bone marrow cells was based on transplantation assays. Noteworthy, all recipient mice transplanted with bone marrow cells of *MxCDX2* mice (*n* = 8) developed leukemia within 80 days, in contrast to the recipients transplanted with cells of control mice, which remained healthy (*n* = 5; see Fig. 1A). Leukemia in the transplanted mice was similar to that of the donors, with the expansion of CDX2‐positive blasts in the bone marrow, hepatomegaly, and splenomegaly linked to malignant cell invasion (not shown). Altogether, these data provide evidence that aberrant onset of *CDX2* in the hematopoietic lineage generates malignant blasts responsible for acute leukemia, transmissible by transplantation.

### 3.2 Myeloid vs lymphoid imbalance driven by ectopic CDX2

**Fig. 3:**
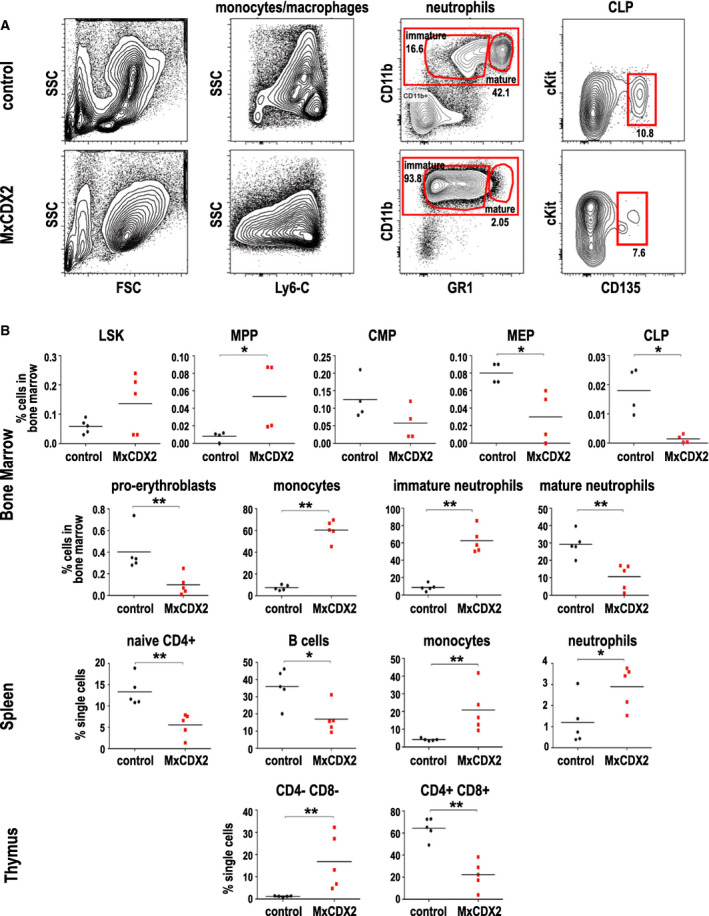
Cellular consequences of the ectopic expression of *CDX2*. (A) Example of cytometry pattern in one control and one *MxCDX2* mouse. Monocytes–macrophages are Cd11b^+^ Gr1^low^ Ly6C^+and−^ SSC^low^. Immature neutrophils are Cd11b^+^ Gr1^low^; mature neutrophils are Cd11b^+^ Gr1^high^. Percentages for neutrophils are given compared with single cells, and for CLP compared with Lin^−^ Il7R^−^ Sca1^low^ cKitl^ow^ cells. In the illustrated examples, CLP represented 0.0243% and 0.000339% of the bone marrow single cells of control and *MxCDX2* mice, respectively. (B) Percent of the indicated cell types in the bone marrow, spleen, and thymus of control and *MxCDX2* mice. CLPs: common lymphoid progenitors; CMPs, common myeloid progenitors; LSK, Lin^−^ Sca^+^ cKit^+^ progenitors; MEPs, megakaryocyte–erythroid progenitors; MPPs, multipotential progenitors. Bars represent means, *P < 0.05; **P < 0.01.

Six weeks after poly(I:C) administration, bone marrow smears from *MxCDX2* mice showed a large proportion of monoblasts, evocative of acute monoblastic leukemia. Flow cytometry and immunophenotyping highlighted the strong imbalance among bone marrow cell populations (Fig. 3A,B), characterized by the expansion of monocytic and granulocytic cells with a nonsignificant increase in Lin^−^Sca^+^cKit^+^ progenitors, a slight increase in multipotent progenitors, and a strong decay of common lymphoid progenitors. In the myeloid lineage, megakaryocyte–erythroid progenitors dropped, leading to a drastic decrease in erythroid cells, reminiscent of the pale color of the bone marrow. On the contrary, monocyte–macrophage and immature neutrophil populations largely expanded. In the thymus, inverse changes in CD4^−^CD8^−^ and CD4^+^CD8^+^ populations revealed a block in T‐cell differentiation. In the spleen, T and B cells decreased, whereas monocyte–macrophage and neutrophil populations increased. These results showed that CDX2 blocks lymphoid commitment and erythroid differentiation to the benefit of monocyte and granulocyte engagement, yet with a defect in complete differentiation.

**Fig. 4:**
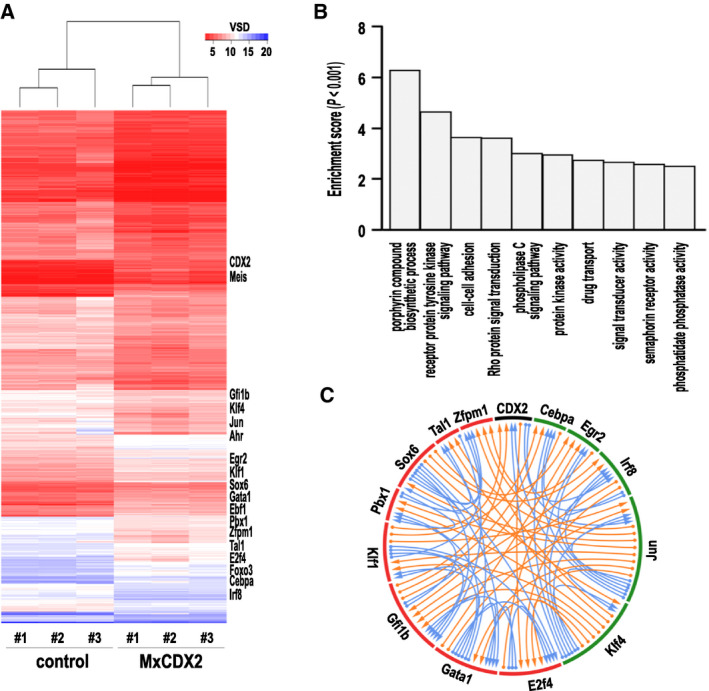
Molecular consequences of the ectopic expression of *CDX2*. (A) Heat map of the differentially expressed genes in the bone marrow of three control and three *MxCDX2* mice. The position of the *CDX2* gene and of major genes involved in hematopoiesis is indicated. (B) Gene Ontology term enrichment of the differentially expressed genes. (C) Circos plot of the relationship between the CDX2 protein or several transcription factors involved in hematopoiesis, and the presence of consensus DNA‐binding sites for these factors in their −2000 to +50 bp gene promoter. Green boxes and red boxes represent transcription factors whose genes are, respectively, upregulated and downregulated by ectopic expression of *CDX2*. Arrows connect a given transcription factor to the presence of consensus DNA‐binding site(s) for this factor in the promoters of transcription factor genes involved in hematopoiesis. Arrows are, respectively, in blue or orange when the factor and its target change in the same way or in opposite ways by ectopic expression of *CDX2*. A table version of this graph is given in Table S5.

Transcriptome analyses performed 6 weeks after poly(I:C) administration revealed 2937 differentially expressed genes in the bone marrow of *MxCDX2* compared with control mice (|log_2_(fold change)| > 1, adj*P*‐value < 0.05; Fig. 4A; Table S3). Functional annotation clustering showed enrichment in genes of the porphyrin biosynthetic pathway corroborating the drop of erythroid cells, as well as in genes involved in cell adhesion and signaling activities suggesting changes in the interactions of hematopoietic cells with their microenvironment (Fig. 4B). Differentially expressed genes also included 221 genes related to DNA binding and regulation of transcription, of which genes encoding important transcription factors for hematopoiesis and leukemogenesis (Table S4). Among them, transcription factor genes involved in monocyte emergence were upregulated (*Irf8*, *Klf4*, *Cebpa*, *Egr2, Jun*), whereas those of the erythroid (*E2f4*, *Gata1*, *Gata2*, *Gfi1b*, *Hoxa7*, *Hoxa9*, *Hoxa10*, *Klf1*, *Meis1*, *Pbx1*, *Sox6*, *Tal1*, *Zfpm1*) and lymphoid pathways (*Ets1*, *Etv5*, *Pax5*, *Pbx1*) were downregulated, consistent with the cellular phenotype. 1010 of the 2937 deregulated genes exhibited one or several consensus CDX DNA‐binding site(s) in their −2000 to +50 bp promoters, including *Irf8* and *Jun* involved in monopoiesis [31, 32]. These binding sites for CDX2 integrate into a complex pattern of deregulated genes encoding transcription factor involved in hematopoiesis and the presence of putative DNA‐binding sites in their gene promoters, suggesting a cascade of deregulations initiated by CDX2 (Fig. 4C, Table S5). Interestingly, deregulated genes also included several genes related to DNA damage and repair (*Btg2*, *Ccnd1*, *Ebf1*, *Foxo3*, *Hmga2*, *Mgmt*, *Rad23a*, *Rb1*, *Xrcc5*), suggesting genome instability. This correlated with the occurrence of a number of nucleotide changes in the transcribed sequences of *MxCDX2* mice, of which 1001 recurrent mutations present in all three mutant mice but in none of the three control littermates (Table S6). 64.6% of these changes were transitions and 26.7% transversions, the others being microdeletions or microinsertions involving < 10 nucleotides. The predicted molecular effects are given in Table S7 and summarized in Fig. S1. Mutational signature analysis best fitted with the models A (35%), B and C (20%), and D (15%) of myeloid leukemia [29]. Substitution profiling according to Ref. [30] also suggested possible contribution of defects in PMS2‐, Exo1‐, PMS1‐, and UNG‐related DNA repair mechanisms (respectively, 50%, 17%, 12%, and 12% correlation). The nucleotide changes fell into ~ 400 genes among which 20 are linked to the GO terms hematopoiesis or leukemia: *Chuk*, *Dleu2*, *Gab3*, *Hdac7*, *Hdac9*, *Hectd1*, *Kcnab2*, *Lrrk1*, *Picalm*, *Pkn1*, *Ppargc1b*, *Prdm16*, *Prdx3*, *Ptprc*, *Rpl22*, *Stap1*, *Trpm2*, *Vps33b*, *Wdr1*, and *Zfp950*.

### 3.3 CDX2 interferes with TGF‐β signaling to perturb the maturation of monoblasts

**Fig. 5:**
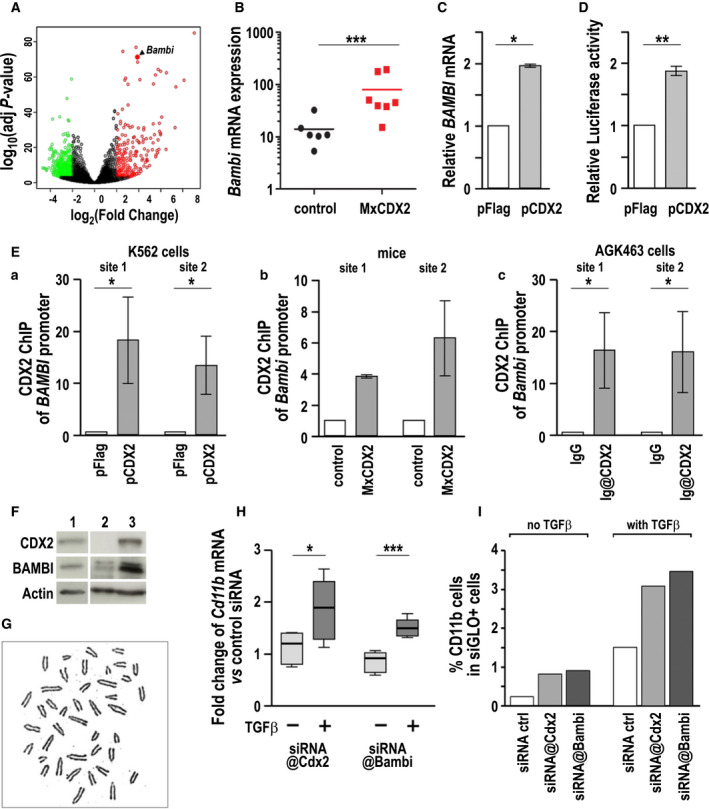
Involvement of BAMBI in the oncogenic effect of CDX2. (A) Volcano plot of the differentially expressed genes in the bone marrow of *MxCDX2* compared with control littermates. Red: upregulated genes; green downregulated genes. The position of *Bambi* is indicated. (B) Comparative expression of the *Bambi* gene by RT‐qPCR in *MxCDX2* (*n* = 7) and control mice (*n* = 6). Bars represent means, ***P < 0.001. (C) Expression of the *BAMBI* gene in human K562 cells transfected with the pFlag‐CDX2 plasmid compared to cells transfected with the control pFlag vector. *n* = 3; data are given means with SEM. *P < 0.02. (D) Luciferase activity produced by pBAMBI‐Luc in human K562 cells transfected with pFlag‐CDX2 or with pFlag. *n* = 9; data are given means with SEM. **P < 0.01. (E) CDX2 chromatin immunoprecipitation of *BAMBI* promoter fragments overlapping the CDX‐type DNA‐binding sites 1 and 2 (a) in human K562 cells transfected with pFlag‐CDX2 vs pFlag (*n* = 5), (b) in bone marrow cells of *MxCDX2* vs control mice (*n* = 2), and (c) in mouse AGK463 nuclear extracts immunoprecipitated with anti‐CDX2 antibodies or with control IgG (*n* = 5). Data are given means with SEM. *P < 0.05. (F) Expression of the CDX2 and BAMBI proteins by western blot in AGK463 cells (lane 1) in comparison with the bone marrow of control and *MxCDX2* mice (respectively, lanes 2 and 3). (G) Karyotype analysis revealing supernumerary chromosomes (*n* = 41) in the AGK463 cell line. (H) Relative *Cd11b* mRNA expression in AGK463 cells transfected with siRNA@CDX2 or siRNA@Bambi compared with control siRNA, in the absence or presence of TGF‐β (*n* = 9). Boxes extend from 25th to 75th percentile, and whiskers represent mean to max. *P < 0.05; ***P < 0.001. (I) Proportion of CD11b^+^ cells among siGLO^+^ AGK463 cells transfected with siGLO and the indicated siRNA, and treated or not with TGF‐β.

Although ectopic CDX2 is expected to alter numerous cellular and molecular functions, RNAseq analysis identified BMP and activin membrane‐bound inhibitor (*Bambi*) among the genes with the strongest stimulation in the leukemia cells of *MxCDX2* mice (Fig. 5A). It was confirmed by RT‐qPCR in an independent series of control (*n* = 6) and *MxCDX2* (*n* = 7) animals (Fig. 5B). The BAMBI protein is a nonsignaling pseudoreceptor of the TGFBR family that represses the TGF‐β pathway [33]. To investigate whether the overexpression of BAMBI may contribute to the malignant phenotype in *MxCDX2* mice, we first investigated the direct effect of CDX2 on the expression of the *Bambi* gene. In human acute myeloid leukemia cells K562, CDX2 overexpression by transfection with the plasmid pFlag‐CDX2 increased the level of endogenous *BAMBI* mRNA (Fig. 5C) and also the activity of the [−3384/+82] human *BAMBI* gene promoter [16], as shown in luciferase reporter assays (Fig. 5D). *In* *silico* analysis revealed two consensus CDX‐type binding sites within a 300 bp segment conserved between human and mice (80% sequence identity) and located, respectively, 560 and 805 bp upstream of the human and mouse *BAMBI* transcription start site. Chromatin immunoprecipitation (ChIP) with anti‐CDX2 antibody demonstrated the occupancy of both CDX‐type sites by the CDX2 protein in pFlag‐CDX2‐transfected K562 cells unlike cells transfected with the control plasmid pFlag (Fig. 5Ea). Moreover, the corresponding sites in the mouse *Bambi* gene promoter were also occupied by CDX2 *in vivo* in bone marrow cells of *MxCDX2* compared with control littermates (Fig. 5Eb).

Next, advantage was taken from the replating potential of *MxCDX2* bone marrow cells to derive a cell line, named AGK463. This cell line expressed the CDX2 and BAMBI proteins (Fig. 5F), exhibited a monoblastic phenotype, and showed aneuploidy (*n* = 41 chromosomes, Fig. 5G), in line with the genome instability mentioned above in bone marrow cells of *MxCDX2* mice. Anti‐CDX2 ChIP also demonstrated the binding of the CDX2 protein on both CDX‐type binding sites of the *Bambi* promoter in AGK463 cells (see Fig. 5Ec). These cells were used to investigate the effect of CDX2 and BAMBI on their differentiation potential in response to TGF‐β. For this purpose, cells were transfected with control siRNA or with siRNA@CDX2 or siRNA@Bambi, which decreased by twofold the corresponding mRNA levels. As shown in Fig. 5H, in the absence of TGF‐β, the level of *Cd11b* mRNA was poorly affected in cells transfected with either siRNA@CDX2 or siRNA@Bambi compared with control siRNA. On the contrary, when TGF‐β was added in the culture medium, decreasing the level of *CDX2* transcript with siRNA@CDX2 stimulated *Cd11b* expression. Moreover, *Cd11b* expression was also stimulated upon TGF‐β signaling activation in the CDX2‐expressing AGK463 cells transfected with siRNA@Bambi. By cytometry, the proportion of AGK463 cells expressing CD11b at the membrane increased by transfection with siRNA@CDX2 or siRNA@Bambi, and the increase was further strengthened after TGF‐β treatment (Fig. 5I). Thus, one of the effects of CDX2 is to jeopardize the TGF‐β‐dependent maturation of monocytes through its stimulatory effect of the expression of the TGF‐β signaling inhibitor BAMBI. BAMBI represents a mediator of the oncogenic potential of CDX2 in hematopoietic cells by compromising the impact of TGF‐β on monocyte differentiation.

## 4 Conclusion

In conclusion, this study shows that the ectopic expression of *CDX2* in the hematopoietic lineage triggers acute leukemia associated with genome instability and profound changes in gene expression patterns. The malignant phenotype observed here is more drastic and penetrant than in a recent model leading predominantly to myelodysplasia by targeting CDX2 selectively in hematopoietic stem cells [10], likely because the induction of CDX2 driven by the *Mx1Cre* allele affects more cells of the hematopoietic lineage than only stem cells. Previous studies have shown that the proliferation of acute myeloid leukemia cells is compromised when *CDX2* is knocked down, and, conversely, that inducing *Cdx2* *ex vivo* in hematopoietic progenitors increases self‐renewal [8]. The present data are in line with these observations, and they further show that CDX2 also introduces a bias in hematopoiesis in favor of the myeloid lineage, while compromising the maturation of monocytes through its stimulatory effect on a negative regulator of the TGF‐β signaling pathway, BAMBI. Thus, unlike its tumor suppressor activity at its physiological site of expression, the gut, the *CDX2* homeobox gene is oncogenic in the ectopic setting of the hematopoietic lineage. Although these puzzling effects are far from being completely understood, they highlight the context‐dependent function of this gene. A context‐dependent activity of CDX2 has already been described regarding its nontranscriptional activity on DNA repair between colon cancer and leukemia cells [18]. Recent studies highlighting the role of CDX2 in the maintenance of open chromatin domains also allow anticipating a context‐dependent effect of this homeoprotein at the transcriptional level [34, 35], since the ultimate effect of keeping open chromatin regions depends on the specific repertoire of DNA‐binding factors able to interact with these regions in each particular cell type. Besides, we have previously reported that the level of CDX2 has an impact on the cellular and molecular microenvironment in the intestinal epithelium [4], and the present study shows that it also has an effect on the way leukemia cells respond to an extracellular signaling molecule, namely TGF‐β. Because of the complexity of TGF‐β signaling with regard to the number of members of this pathway and to its connection with other pathways, further studies are needed to thoroughly explore how CDX2 interferes with them. Yet, these data underscore the role played by CDX2 as a major actor of the crosstalk with the extracellular microenvironment in both physiological and pathological situations.

## Author contributions

CD‐D, ID, and J‐NF conceived the study, analyzed the results, and wrote the manuscript. CD‐D, KH, YG, and AG performed animal, cellular, and molecular studies. VG performed sequencing and transcriptomic data analysis. EM, CE, and MT helped in immunohistochemistry, cytometry, and serial replating experiments, respectively. LM, LV, and M‐PC performed pathological evaluation of the bone marrow smears and organs at the Hematology Laboratory and at the Pathology Department of the University Hospital of Strasbourg.

## Conflict of interest

The authors declare no conflict of interest.

## Supporting information

**Fig. S1**. Proportion of the predicted consequences of the recurrent mutational changes observed in MxCDX2 mice.

**Table S1**. Primers, probes and siRNA.

**Table S2**. List of antibodies used for flow cytometry.

**Table S3**. Differentially‐expressed genes in the bone marrow of *MxCDX2* mice compared to control littermates.

**Table S4**. Differentially‐expressed genes for DNA binding/Transcription Regulatory Factors.

**Table S5**. Combined analysis of DNA‐binding sites in promoter of hematopoietic transcription factors and their respective deregulated expression.

**Table S6**. Recurrent nucleotide changes in the transcribed sequences of *MxCDX2* mice compared to control littermates.

**Table S7**. Prediction of the consequences of the nucleotide changes in *MxCDX2* mice.

## Data Availability

RNAseq data have been deposited in the GEO database under the accession number GSE120487.

## References

[1] Chawengsaksophak K (2019) Cdx2 animal models reveal developmental origins of cancers. Genes (Basel) 10: 928. 10.3390/genes10110928 PMC6895827 31739541

[2] Aoki K, Kakizaki F, Sakashita H, Manabe T, Aoki M & Taketo MM (2011) Suppression of colonic polyposis by homeoprotein CDX2 through its nontranscriptional function that stabilizes p27Kip1. Cancer Res 71, 593–602. 21224344 10.1158/0008-5472.CAN-10-2842

[3] Bonhomme C, Duluc I, Martin E, Chawengsaksophak K, Chenard MP, Kedinger M, Beck F, Freund JN & Domon‐Dell C (2003) The Cdx2 homeobox gene has a tumour suppressor function in the distal colon in addition to a homeotic role during gut development. Gut 52, 1465–1471. 12970140 10.1136/gut.52.10.1465 PMC1773830

[4] Balbinot C, Armant O, Elarouci N, Marisa L, Martin E, De Clara E, Onea A, Deschamps J, Beck F, Freund J‐N*et al*. (2018) The Cdx2 homeobox gene suppresses intestinal tumorigenesis through non‐cell‐autonomous mechanisms. J Exp Med 215, 911–926. 29439001 10.1084/jem.20170934 PMC5839756

[5] Sakamoto N, Feng Y, Stolfi C, Kurosu Y, Green M, Lin J, Green ME, Sentani K, Yasui W, McMahon M*et al*. (2017) BRAFV600E cooperates with CDX2 inactivation to promote serrated colorectal tumorigenesis. Elife 6, e20331. 28072391 10.7554/eLife.20331 PMC5268782

[6] Hryniuk A, Grainger S, Savory JGA & Lohnes D (2014) Cdx1 and Cdx2 function as tumor suppressors. J Biol Chem 289, 33343–33354. 25320087 10.1074/jbc.M114.583823 PMC4246091

[7] Darvishi M, Mashati P & Khosravi A (2018) The clinical significance of CDX2 in leukemia: a new perspective for leukemia research. Leuk Res 72, 45–51. 30096576 10.1016/j.leukres.2018.07.021

[8] Scholl C, Bansal D, Dohner K, Eiwen K, Huntly BJ, Lee BH, Rucker FG, Schlenk RF, Bullinger L, Dohner H*et al*. (2007) The homeobox gene CDX2 is aberrantly expressed in most cases of acute myeloid leukemia and promotes leukemogenesis. J Clin Invest 117, 1037–1048. 17347684 10.1172/JCI30182 PMC1810574

[9] Thoene S, Rawat VP, Heilmeier B, Hoster E, Metzeler KH, Herold T, Hiddemann W, Gokbuget N, Hoelzer D, Bohlander SK*et al*. (2009) The homeobox gene CDX2 is aberrantly expressed and associated with an inferior prognosis in patients with acute lymphoblastic leukemia. Leukemia 23, 649–655. 19158837 10.1038/leu.2008.355

[10] Vu T, Straube J, Porter A, Bywater M, Song A, Ling V, Cooper L, Pali G, Bruedigam C, Jacquelin S*et al*. (2020) Hematopoietic stem and progenitor cell‐restricted Cdx2 expression induces transformation to myelodysplasia and acute leukemia. Nat Commun 15, 3021. 10.1038/s41467-020-16840-2 PMC7296000 32541670

[11] Bataller A, Montalban‐Bravo G, Soltysiak K & Garcia‐Manero G (2019) The role of TGFβ in hematopoiesis and myeloid disorders. Leukemia 3, 1076–1089. 10.1038/s41375-019-0420-1 PMC11789621 30816330

[12] Blank U & Karlsson S (2015) TGF‐β signaling in the control of hematopoietic stem cells. Blood 125, 3542–3550. 25833962 10.1182/blood-2014-12-618090

[13] Grall E, Gourain V, Naïr A, Martin E, Birling M‐C, Freund J‐N & Duluc I (2019) Severe head dysgenesis resulting from imbalance between anterior and posterior ontogenetic programs. Cell Death Dis 10, 812. 31649239 10.1038/s41419-019-2040-0 PMC6813351

[14] Kuhn R, Schwenk F, Aguet M & Rajewsky K (1995) Inducible gene targeting in mice. Science 269, 1427–1429. 7660125 10.1126/science.7660125

[15] Balbinot C, Vanier M, Armant O, Nair A, Penichon J, Soret C, Martin E, Saandi T, Reimund J‐M, Deschamps J*et al*. (2017) Fine‐tuning and autoregulation of the intestinal determinant and tumor suppressor homeobox gene CDX2 by alternative splicing. Cell Death Differ 24, 2173–2186. 28862703 10.1038/cdd.2017.140 PMC5686355

[16] Sekiya T, Adachi S, Kohu K, Yamada T, Higuchi O, Furukawa Y, Nakamura Y, Nakamura T, Tashiro K, Kuhara S*et al*. (2004) Identification of BMP and activin membrane‐bound inhibitor (BAMBI), an inhibitor of transforming growth factor‐beta signaling, as a target of the beta‐catenin pathway in colorectal tumor cells. J Biol Chem 279, 6840–6846. 14660579 10.1074/jbc.M310876200

[17] Lozzio CB & Lozzio BB (1975) Human chronic myelogenous leukemia cell‐line with positive Philadelphia chromosome. Blood 45, 321–334. 163658

[18] Renouf B, Soret C, Saandi T, Delalande F, Martin E, Vanier M, Duluc I, Gross I, Freund J‐N & Domon‐Dell C (2012) Cdx2 homeoprotein inhibits non‐homologous end joining in colon cancer but not in leukemia cells. Nucleic Acids Res 40, 3456–3469. 22189105 10.1093/nar/gkr1242 PMC3333856

[19] Dobin A, Davis CA, Schlesinger F, Drenkow J, Zaleski C, Jha S, Batut P, Chaisson M & Gingeras TR (2013) STAR: ultrafast universal RNA‐seq aligner. Bioinformatics 29, 15–21. 23104886 10.1093/bioinformatics/bts635 PMC3530905

[20] Anders S, Pyl PT & Huber W (2015) HTSeq–a Python framework to work with high‐throughput sequencing data. Bioinformatics 31, 166–169. 25260700 10.1093/bioinformatics/btu638 PMC4287950

[21] Love MI, Huber W & Anders S (2014) Moderated estimation of fold change and dispersion for RNA‐seq data with DESeq2. Genome Biol 15, 550. 25516281 10.1186/s13059-014-0550-8 PMC4302049

[22] Huang DW, Sherman BT & Lempicki RA (2009) Systematic and integrative analysis of large gene lists using DAVID bioinformatics resources. Nat Protoc 4, 44–57. 19131956 10.1038/nprot.2008.211

[23] Heinz S, Benner C, Spann N, Bertolino E, Lin YC, Laslo P, Cheng JX, Murre C, Singh H & Glass CK (2010) Simple combinations of lineage‐determining transcription factors prime cis‐regulatory elements required for macrophage and B cell identities. Mol Cell 38, 576–589. 20513432 10.1016/j.molcel.2010.05.004 PMC2898526

[24] Khan A, Fornes O, Stigliani A, Gheorghe M, Castro‐Mondragon JA, van der Lee R, Bessy A, Chèneby J, Kulkarni SR, Tan G*et al*. (2018) JASPAR 2018: update of the open‐access database of transcription factor binding profiles and its web framework. Nucleic Acids Res 46, D260–D266. 29140473 10.1093/nar/gkx1126 PMC5753243

[25] Bailey T, Johnson J, Grant C & Noble W (2015) The MEME suite. Nucleic Acids Res 43, W39–W49. 25953851 10.1093/nar/gkv416 PMC4489269

[26] Van der Auwera GA, Carneiro MO, Hartl C, Poplin R, Del Angel G, Levy‐Moonshine A, Jordan T, Shakir K, Roazen D, Thibault J*et al*. (2013) From FastQ data to high confidence variant calls: the Genome Analysis Toolkit best practices pipeline. Curr Protoc Bioinformatics 43, 11.10.1–11.10.33. 25431634 10.1002/0471250953.bi1110s43 PMC4243306

[27] Poplin R, Ruano‐Rubio V, DePristo MA, Fennell TJ, Carneiro MO, Van der Auwera GA, Kling DE, Gauthier LD, Levy‐Moonshine A, Roazen D*et al*. (2017) Scaling accurate genetic variant discovery to tens of thousands of samples. bioRxiv [PREPRINT].

[28] McLaren W, Gil L, Hunt SE, Riat HS, Ritchie GRS, Thormann A, Flicek P & Cunningham F (2016) The ensembl variant effect predictor. Genome Biol 17, 122. 27268795 10.1186/s13059-016-0974-4 PMC4893825

[29] Degasperi A, Amarante TD, Czarnecki J, Shooter S, Zou X, Glodzik D, Morganella S, Nanda AS, Badja C, Koh G*et al*. (2020) A practical framework and online tool for mutational signature analyses show inter‐tissue variation and driver dependencies. Nat Cancer 1, 249–263. 32118208 10.1038/s43018-020-0027-5 PMC7048622

[30] Zou X, Koh GCC, Nanda AS, Degasperi A, Urgo K, Roumeliotis TI, Agu CA, Side L, Brice G, Perez‐Alonso V*et al*. (2020) Dissecting mutational chanisms underpinning signatures caused by replication errors and endogenous DNA damage. bioRxiv [PREPRINT]. 10.1038/s43018-021-00200-0 PMC7611045 34164627

[31] Friedman AD (2007) C/EBPα Induces PU.1 and interacts with AP‐1 and NF‐κB to regulate myeloid development. Blood Cells Mol Dis 39, 340–343. 17669672 10.1016/j.bcmd.2007.06.010 PMC2083642

[32] Kurotaki D, Osato N, Nishiyama A, Yamamoto M, Ban T, Sato H, Nakabayashi J, Umehara M, Miyake N, Matsumoto N*et al*. (2013) Essential role of the IRF8‐KLF4 transcription factor cascade in murine monocyte differentiation. Blood 121, 1839–1849. 23319570 10.1182/blood-2012-06-437863 PMC3591803

[33] Onichtchouk D, Chen Y, Dosch R, Gawantka V, Delius H, Massagué J & Niehrs C (1999) Silencing of TGF‐beta signalling by the pseudoreceptor BAMBI. Nature 401, 480–485. 10519551 10.1038/46794

[34] Saxena M, Roman AKS, O’Neill NK, Sulahian R, Jadhav U & Shivdasani RA (2017) Transcription factor‐dependent “anti‐repressive” mammalian enhancers exclude H3K27me3 from extended genomic domains. Genes Dev 31, 2391–2404. 29321178 10.1101/gad.308536.117 PMC5795785

[35] Verzi MP, Shin H, San Roman AK, Liu XS & Shivdasani RA (2013) Intestinal master transcription factor CDX2 controls chromatin access for partner transcription factor binding. Mol Cell Biol 33, 281–292. 23129810 10.1128/MCB.01185-12 PMC3554120

