# Supplementary material for: CDX2 expression in the hematopoietic lineage promotes leukemogenesis via TGFβ inhibition
Source: Mol Oncol. 2021 Jun 26;15(9):2318–29. doi: 10.1002/1878-0261.12982 (PMC8410536; doi:10.1002/1878-0261.12982)
Supplement: Supplementary file 1 — Fig. S1. Proportion of the predicted consequences of the recurrent mutational changes observed in MxCDX2 mice. [file MOL2-15-2318-s003.pdf]

Figure S1

Proportion of the predicted consequences of the recurrent mutational changes observed in *MxCDX2* mice.

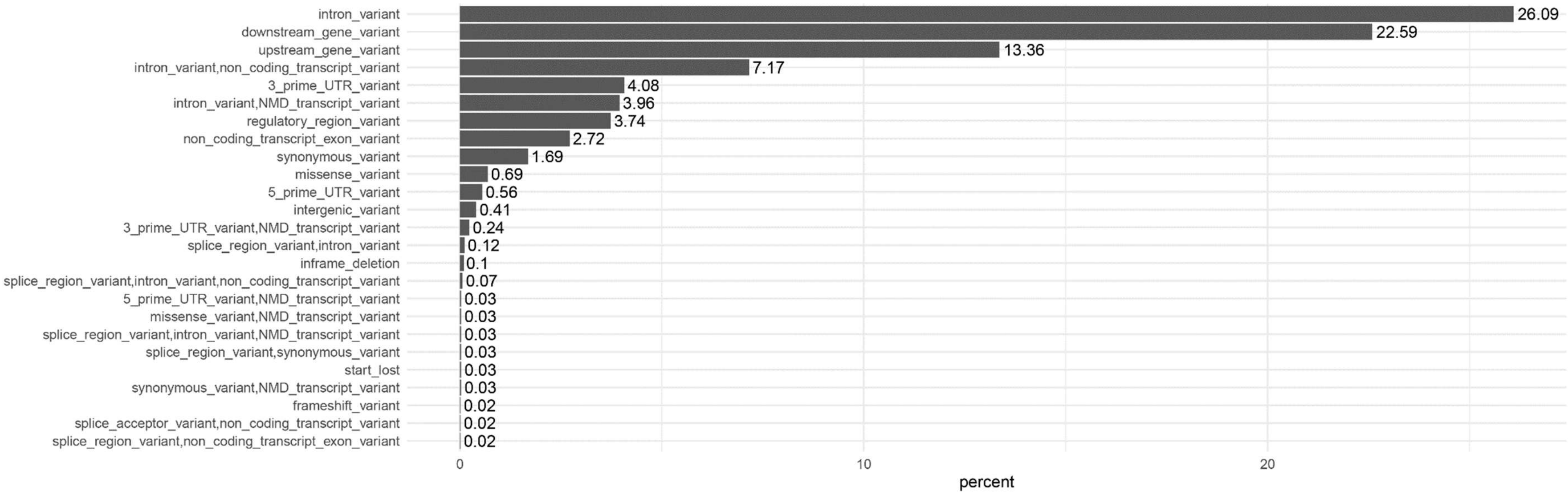

Data represented here are plotted from the column “Consequence” of the Supplementary Table S7.
