# Supplementary material for: CDX2 expression in the hematopoietic lineage promotes leukemogenesis via TGFβ inhibition
Source: Mol Oncol. 2021 Jun 26;15(9):2318–29. doi: 10.1002/1878-0261.12982 (PMC8410536; doi:10.1002/1878-0261.12982)
Supplement: Supplementary file 2 — Table S1. Primers, probes and siRNA. [file MOL2-15-2318-s001.docx]

**Table S1**

Primers, probes and siRNA.

| **Genotyping** | **5’>3’ primer sequences** | **Supplier** |
| --- | --- | --- |
| RsCDX2-F | GTGGTTTGTCCAAACTCATCA | Invitrogen |
| RsCDX2-R | CACGTGGTAACCGCCGTAGTC | Invitrogen |
| Mx1Cre-F | CGGTCGATGCAACGAGTGATGAGG | Invitrogen |
| Mx1Cre-R | CCAGAGACGGAAATCCATCGCTCG | Invitrogen |
| **ChIP** | **5’>3’ primer sequences** |  |
| mBambi_S1_F | TCATCTCCATCTTGGTTCCTTTAGG | Invitrogen |
| mBambi_S1_R | GACCTGATCAATATCAGTCCCACAG | Invitrogen |
| mBambi_S2_F | CGTTAAGTGTCTCTAGCAGGCTCC | Invitrogen |
| mBambi_S2_R | GGAGTAGGGTGCACATAGTCAAGAAG | Invitrogen |
| hBambi_S1_F | CTACAGCAAGACCACCTTGAGTACCT | Invitrogen |
| hBambi_S1_R | CTGACCCTGATCAATATCAGCCC | Invitrogen |
| hBambi_S2_F | GAGAACCTAGCGGCTCCTAACGG | Invitrogen |
| hBambi_S2_R | TGGGGTCTGCATTCAGCCTTC | Invitrogen |
| **TaqMan Probes** | **Reference** |  |
| mCdx2 | Mm01212280_m1 | Applied Biosystems |
| mBambi | Mm01238922_g1 | Applied Biosystems |
| mITGAM/Cd11b | Mm00434455_m1 | Applied Biosystems |
| mTbp | Mm00446973_m1 | Applied Biosystems |
| hCDX2 | Hs00230919_m1 | Applied Biosystems |
| hBambi | Hs03044164_m1 | Applied Biosystems |
| hTBP | Hs00427620_m1 | Applied Biosystems |
| **siRNA** | **Reference** |  |
| si@CDX2 | Silencer® Select, ID=s2876 | Ambion |
| si@Bambi | Silencer® Select,ID=s86069 | Ambion |
| siControl | Silencer® Select Negative Control #2 | Ambion |
| siGLO | siGLO RISC-Free Control | Horizon Perkin-Elmer |
