# Supplementary material for: CDX2 expression in the hematopoietic lineage promotes leukemogenesis via TGFβ inhibition
Source: Mol Oncol. 2021 Jun 26;15(9):2318–29. doi: 10.1002/1878-0261.12982 (PMC8410536; doi:10.1002/1878-0261.12982)
Supplement: Supplementary file 3 — Table S2. List of antibodies used for flow cytometry. [file MOL2-15-2318-s007.docx]

**Table S2**

List of antibodies used for flow cytometry.

| **Antibody** | **Format** | **Clone** | **Supplier** |
| --- | --- | --- | --- |
| B220 | Biotin | RA3-6B2 | e-biosciences |
| B220 | PE-Cy7 | RA3-6B2 | e-biosciences |
| BP1 | FITC | 6C3 | BD Biosciences |
| CD3 | Biotin | 145-2C11 | e-biosciences |
| CD3 | PerCP-Cy5.5 | 145-2C11 | Biolegend |
| CD4 | APC-H7 | GK1.5 | BD Biosciences |
| CD4 | Biotin | GK1.5 | BioLegend |
| CD8 | Alexa 700 | 53‐6.7 | e-Biosciences |
| CD8 | APC-eFluor780 | 53-6.7 | e-Biosciences |
| CD8 | Biotin | 53-6.7 | e-biosciences |
| CD11b | Biotin | M1/70 | e-biosciences |
| CD11b (Mac-1) | PerCP-Cy5.5 | M1/70.15 | e-Biosciences |
| CD11b | FITC | ICRF44 | BD Biosciences |
| CD11c | APC | N418 | e-Biosciences |
| CD11c | Biotin | N418 | e-biosciences |
| CD16/CD32 | APC-Cy7 | 2.4G2 | BD Biosciences |
| CD16/CD32 | purified | 2.4G2 | BD Biosciences |
| CD19 | PerCP-Cy5.5 | 1D3 | BD Biosciences |
| CD24 | Biotin | 30-F1 | Souther Biotech |
| CD34 | FITC | RAM34 | BD Biosciences |
| CD43 | PE | S7 | BD Biosciences |
| CD44 (Pgp-1) | PE-CF594 | IM7.8.1 | BD Biosciences |
| CD48 | APC | HM48-1 | eBiosccience |
| CD49b | Biotin | DX5 | e-biosciences |
| CD62L | FITC | MEL-14 | BD Biosciences |
| CD71 | BV 605 | C2 | BD Biosciences |
| CD93 (AA4.1) | APC | AA4.1 | eBiosccience |
| CD117(cKit) | APC | 2B8 | e-biosciences |
| CD117(cKit) | APC-Cy7 | 2B8 | BioLegend |
| CD127 | PE-CF594 | SB/99 | BD Biosciences |
| CD135 (Flt-3) | PE | A2F10.1 | e-biosciences |
| CD150 | PerCP-Cy5.5 | TC15-12F12.2 | BioLegend |
| Gr-1 | Biotin | RB6-8C5 | e-biosciences |
| IgM | Cy5 | n.a. | Southern Biotech |
| Ly-6C | Alexa 700 | Al-21 | BD Biosciences |
| Ly-6G (Gr-1) | eFluor® 450NC | RB6‐8C5 | e-Biosciences |
| Sca1 | PE-Cy7 | D7 | BD Biosciences |
| Siglec-F | PE | E50-2440 | BD Biosciences |
| Streptavidine | Alexa 405 |  | Invitrogen |
| Streptavidine | PE-Cy7 |  | BD Biosciences |
| TER-119 | PE | TER-119 | BD Biosciences |
| TER-119 (Ly-76) | Biotin | TER-119 | eBioscciences |
