# Supplementary material for: CDX2 expression in the hematopoietic lineage promotes leukemogenesis via TGFβ inhibition
Source: Mol Oncol. 2021 Jun 26;15(9):2318–29. doi: 10.1002/1878-0261.12982 (PMC8410536; doi:10.1002/1878-0261.12982)
Supplement: Supplementary file 4 — Table S3. Differentially‐expressed genes in the bone marrow of MxCDX2 mice compared to control littermates. [file MOL2-15-2318-s002.docx]

**Table S3**

Differentially-expressed genes in the bone marrow of *MxCDX2* mice compared to control littermates.

| **ensGeneID** | **Gene_Symbol** | **log2-FoldChange** | **padj** | **MxCDX2 Sample_1** | **MxCDX2 Sample_2** | **MxCDX2 Sample_3** | **Ctrl Sample_1** | **Ctrl Sample_2** | **Ctrl Sample_3** |
| --- | --- | --- | --- | --- | --- | --- | --- | --- | --- |
| ENSMUSG00000060512 | 0610040J01Rik | 2,91 | 6,97217E-18 | 689,4695 | 568,1772 | 634,0528 | 69,38998 | 124,3004 | 60,50296 |
| ENSMUSG00000085972 | 1110028F11Rik | -2,07 | 0,024072535 | 16,86776 | 17,39318 | 9,515116 | 25,77342 | 27,90417 | 129,0137 |
| ENSMUSG00000046999 | 1110032F04Rik | -2,12 | 0,017171364 | 4,216939 | 1,932576 | 5,190064 | 15,86057 | 13,95208 | 19,57449 |
| ENSMUSG00000097290 | 1300002E11Rik | -1,28 | 4,23863E-09 | 154,9725 | 170,0667 | 168,6771 | 373,7146 | 366,5593 | 459,1107 |
| ENSMUSG00000033688 | 1300017J02Rik | -3,57 | 6,38977E-09 | 453,3209 | 75,37045 | 190,3023 | 3326,754 | 3108,778 | 2074,896 |
| ENSMUSG00000026051 | 1500015O10Rik | -1,46 | 0,027913963 | 11,59658 | 11,59545 | 18,16522 | 25,77342 | 31,70928 | 56,05421 |
| ENSMUSG00000097383 | 1500026H17Rik | -3,48 | 0,002063532 | 3,162704 | 4,831439 | 1,730021 | 12,88671 | 15,22046 | 79,18769 |
| ENSMUSG00000035595 | 1600002K03Rik | 1,10 | 3,85274E-06 | 565,0698 | 476,3799 | 631,4577 | 271,6122 | 224,5017 | 284,7198 |
| ENSMUSG00000031927 | 1700012B09Rik | 6,30 | 8,73054E-58 | 659,9509 | 528,5594 | 615,0225 | 6,938998 | 11,41534 | 5,338496 |
| ENSMUSG00000089827 | 1700023H06Rik | -2,32 | 0,000417452 | 9,488113 | 6,764015 | 3,460042 | 32,71242 | 36,78277 | 27,58223 |
| ENSMUSG00000032666 | 1700025G04Rik | 2,56 | 2,17883E-42 | 3688,767 | 3727,938 | 3777,501 | 521,4162 | 679,847 | 700,2328 |
| ENSMUSG00000097893 | 1700034P13Rik | -2,44 | 2,49339E-07 | 16,86776 | 13,52803 | 13,84017 | 80,29412 | 105,2748 | 55,16446 |
| ENSMUSG00000043773 | 1700048O20Rik | -1,22 | 0,021900949 | 73,79643 | 46,38181 | 83,90603 | 134,8148 | 100,2013 | 241,1221 |
| ENSMUSG00000040657 | 1700063H04Rik | -3,04 | 2,69421E-05 | 8,433878 | 2,898863 | 3,460042 | 44,60785 | 49,46648 | 25,80273 |
| ENSMUSG00000086771 | 1700080G11Rik | 8,51 | 0,013834569 | 5,271174 | 8,69659 | 27,68034 | 0 | 0 | 0 |
| ENSMUSG00000086016 | 1700084C06Rik | 1,85 | 0,024553018 | 28,46434 | 79,2356 | 22,49028 | 14,86928 | 7,610228 | 13,34624 |
| ENSMUSG00000097080 | 1700086O06Rik | -1,88 | 5,91703E-06 | 24,2474 | 43,48295 | 45,84556 | 176,4488 | 107,8116 | 136,1317 |
| ENSMUSG00000041707 | 1810011H11Rik | 1,56 | 4,94069E-15 | 1537,074 | 1314,151 | 1548,369 | 426,2527 | 517,4955 | 548,9754 |
| ENSMUSG00000073155 | 1810058I24Rik | -1,42 | 2,63407E-08 | 2997,189 | 2639,898 | 3097,603 | 6753,628 | 6613,288 | 10027,48 |
| ENSMUSG00000063018 | 2010204K13Rik | -2,30 | 1,83717E-05 | 13,70505 | 10,62917 | 15,57019 | 54,5207 | 46,92974 | 95,20318 |
| ENSMUSG00000029882 | 2210010C04Rik | 1,25 | 0,040068707 | 30,57281 | 76,33674 | 74,39091 | 21,80828 | 25,36743 | 29,36173 |
| ENSMUSG00000097162 | 2310010J17Rik | -1,49 | 1,9023E-07 | 45,33209 | 47,3481 | 54,49567 | 150,6754 | 124,3004 | 138,8009 |
| ENSMUSG00000085965 | 2310016D23Rik | 5,75 | 0,002081818 | 15,81352 | 22,22462 | 17,30021 | 0,991285 | 0 | 0 |
| ENSMUSG00000031983 | 2310022B05Rik | -1,49 | 1,24972E-12 | 900,3165 | 647,4128 | 717,9588 | 2081,699 | 2293,215 | 2007,275 |
| ENSMUSG00000039798 | 2600006K01Rik | -2,51 | 1,3758E-08 | 14,75929 | 9,662878 | 12,11015 | 81,28541 | 63,41857 | 63,17221 |
| ENSMUSG00000087013 | 2610027K06Rik | -2,89 | 3,17275E-06 | 10,54235 | 5,797727 | 7,785095 | 69,38998 | 76,10228 | 32,92073 |
| ENSMUSG00000079671 | 2610203C22Rik | 2,62 | 0,000313581 | 46,38633 | 27,05606 | 22,49028 | 4,956427 | 6,341857 | 4,448747 |
| ENSMUSG00000085936 | 2610307P16Rik | -1,25 | 0,016374936 | 33,73551 | 28,98863 | 60,55074 | 95,1634 | 71,02879 | 128,1239 |
| ENSMUSG00000028536 | 2610528J11Rik | -3,22 | 0,004287903 | 18,97623 | 10,62917 | 7,785095 | 25,77342 | 25,36743 | 296,2865 |
| ENSMUSG00000051339 | 2900026A02Rik | -1,19 | 0,000279795 | 445,9413 | 263,7966 | 440,2904 | 893,1482 | 720,4349 | 1010,755 |
| ENSMUSG00000044544 | 4921513I03Rik | -2,63 | 0,003175184 | 2,108469 | 6,764015 | 4,325053 | 33,70371 | 36,78277 | 12,45649 |
| ENSMUSG00000043168 | 4930426D05Rik | -2,02 | 0,004934518 | 101,2065 | 42,51666 | 103,8013 | 587,8323 | 300,604 | 113,8879 |
| ENSMUSG00000069873 | 4930438A08Rik | -1,26 | 0,000118289 | 1332,553 | 1940,306 | 1329,521 | 4099,957 | 4282,022 | 2610,525 |
| ENSMUSG00000087317 | 4930466I24Rik | -8,29 | 0,016317505 | 0 | 0 | 0 | 9,912855 | 5,073485 | 17,79499 |
| ENSMUSG00000086138 | 4930471C06Rik | 4,00 | 0,018664265 | 6,325408 | 6,764015 | 19,03023 | 0,991285 | 0 | 0,889749 |
| ENSMUSG00000050345 | 4930486L24Rik | -1,72 | 0,032722527 | 23,19316 | 14,49432 | 26,81533 | 30,72985 | 45,66137 | 136,1317 |
| ENSMUSG00000020992 | 4930512B01Rik | -2,15 | 0,00861283 | 6,325408 | 3,865151 | 2,595032 | 18,83442 | 20,29394 | 16,90524 |
| ENSMUSG00000086430 | 4930551O13Rik | -2,86 | 0,004644009 | 2,108469 | 2,898863 | 1,730021 | 17,84314 | 21,56231 | 9,787243 |
| ENSMUSG00000085084 | 4930570G19Rik | 4,11 | 2,83727E-06 | 35,84398 | 131,4151 | 51,90064 | 2,973856 | 1,268371 | 8,007744 |
| ENSMUSG00000078157 | 4931440F15Rik | -3,23 | 0,001455528 | 7,379643 | 4,831439 | 10,38013 | 19,82571 | 29,17254 | 162,8241 |
| ENSMUSG00000097216 | 4932441J04Rik | 3,43 | 7,16966E-07 | 37,95245 | 59,90984 | 51,90064 | 3,965142 | 1,268371 | 8,007744 |
| ENSMUSG00000049357 | 4933408B17Rik | -2,43 | 0,000184395 | 61,14562 | 27,05606 | 11,24514 | 226,0131 | 139,5208 | 167,2729 |
| ENSMUSG00000045877 | 4933415A04Rik | -2,85 | 0,034030906 | 1,054235 | 1,932576 | 2,595032 | 4,956427 | 8,878599 | 26,69248 |
| ENSMUSG00000047988 | 4933428G20Rik | -2,49 | 0,00104988 | 5,271174 | 6,764015 | 2,595032 | 33,70371 | 29,17254 | 18,68474 |
| ENSMUSG00000045928 | 4933440M02Rik | -2,87 | 0,002636364 | 5,271174 | 3,865151 | 3,460042 | 42,62527 | 40,58788 | 8,897494 |
| ENSMUSG00000054944 | 5330416C01Rik | -4,08 | 0,000215622 | 3,162704 | 0,966288 | 0 | 24,78214 | 17,7572 | 23,13348 |
| ENSMUSG00000062046 | 5730460C07Rik | -2,95 | 0,000273271 | 12,65082 | 0,966288 | 5,190064 | 50,55556 | 57,07671 | 36,47972 |
| ENSMUSG00000032985 | 5730522E02Rik | 2,85 | 0,019521587 | 6,325408 | 17,39318 | 12,97516 | 1,982571 | 1,268371 | 1,779499 |
| ENSMUSG00000025573 | 6030468B19Rik | -2,98 | 1,8977E-09 | 92,77266 | 37,68522 | 29,41036 | 392,549 | 341,1919 | 522,2829 |
| ENSMUSG00000018451 | 6330403K07Rik | -1,74 | 0,00148258 | 12,65082 | 12,56174 | 8,650106 | 35,68628 | 31,70928 | 44,48747 |
| ENSMUSG00000075585 | 6330403L08Rik | -1,25 | 0,012962389 | 22,13893 | 27,05606 | 27,68034 | 51,54684 | 45,66137 | 85,41594 |
| ENSMUSG00000031824 | 6430548M08Rik | -1,16 | 2,26064E-05 | 5784,586 | 7207,541 | 5560,288 | 15812,99 | 15377,73 | 10319,31 |
| ENSMUSG00000085058 | 8030453O22Rik | 1,45 | 0,004647988 | 44,27786 | 46,38181 | 65,7408 | 22,79957 | 12,68371 | 21,35398 |
| ENSMUSG00000048489 | 8430408G22Rik | -1,46 | 0,001279958 | 87,50148 | 62,80871 | 127,1566 | 186,3617 | 223,2334 | 354,1202 |
| ENSMUSG00000079069 | 8430423G03Rik | 3,14 | 8,81118E-05 | 22,13893 | 39,6178 | 44,98055 | 6,938998 | 1,268371 | 3,558997 |
| ENSMUSG00000086449 | 9030204H09Rik | -8,48 | 0,022613509 | 0 | 0 | 0 | 1,982571 | 2,536743 | 32,92073 |
| ENSMUSG00000021185 | 9030617O03Rik | 1,06 | 1,69245E-06 | 1387,373 | 1811,79 | 1795,762 | 885,2179 | 712,8247 | 802,5539 |
| ENSMUSG00000053168 | 9030619P08Rik | -2,62 | 0,000239391 | 22,13893 | 14,49432 | 25,08531 | 62,45098 | 69,76042 | 246,4606 |
| ENSMUSG00000097100 | 9230104M06Rik | 2,30 | 0,038036608 | 8,433878 | 21,25833 | 10,38013 | 1,982571 | 2,536743 | 3,558997 |
| ENSMUSG00000097177 | 9330159M07Rik | -1,41 | 0,005468175 | 42,16939 | 75,37045 | 64,87579 | 178,4314 | 216,8915 | 90,75444 |
| ENSMUSG00000097194 | 9330175E14Rik | 1,42 | 6,10158E-06 | 806,4896 | 742,109 | 461,9157 | 268,6384 | 219,4282 | 264,2556 |
| ENSMUSG00000094447 | 9430069I07Rik | 3,31 | 0,002828651 | 23,19316 | 20,29204 | 18,16522 | 4,956427 | 1,268371 | 0 |
| ENSMUSG00000085447 | 9530026F06Rik | -4,57 | 0,003158891 | 2,108469 | 0 | 0 | 19,82571 | 17,7572 | 8,897494 |
| ENSMUSG00000036411 | 9530077C05Rik | -1,39 | 0,005694952 | 142,3217 | 191,325 | 89,9611 | 466,8955 | 432,5146 | 208,2014 |
| ENSMUSG00000073386 | 9830107B12Rik | -1,75 | 3,31032E-06 | 930,8893 | 831,9738 | 528,5215 | 2938,17 | 3111,315 | 1668,28 |
| ENSMUSG00000087528 | 9830144P21Rik | -1,82 | 3,51821E-07 | 84,33878 | 76,33674 | 42,38552 | 265,6645 | 213,0864 | 234,0041 |
| ENSMUSG00000097493 | 9930014A18Rik | 1,68 | 0,002894493 | 121,237 | 213,5496 | 142,7267 | 24,78214 | 44,393 | 79,18769 |
| ENSMUSG00000069893 | 9930111J21Rik1 | 1,07 | 4,27158E-06 | 930,8893 | 961,4564 | 753,4242 | 450,0436 | 351,3389 | 455,5517 |
| ENSMUSG00000087178 | A230056P14Rik | -1,61 | 2,88745E-08 | 64,30832 | 46,38181 | 45,84556 | 152,658 | 168,6934 | 155,7061 |
| ENSMUSG00000097666 | A330094K24Rik | -4,99 | 0,0005052 | 0 | 1,932576 | 0 | 17,84314 | 15,22046 | 27,58223 |
| ENSMUSG00000097353 | A430046D13Rik | 1,08 | 4,21449E-05 | 724,2593 | 1065,815 | 936,8065 | 451,0349 | 366,5593 | 474,2364 |
| ENSMUSG00000045838 | A430105I19Rik | -1,78 | 5,12931E-05 | 20,03046 | 33,82007 | 17,30021 | 89,21569 | 69,76042 | 84,52619 |
| ENSMUSG00000047878 | A4galt | -1,57 | 0,007407975 | 20,03046 | 21,25833 | 18,16522 | 54,5207 | 32,97765 | 88,08519 |
| ENSMUSG00000043939 | A530064D06Rik | -1,98 | 6,4993E-14 | 691,578 | 561,4132 | 498,2461 | 2279,957 | 2789,149 | 1820,427 |
| ENSMUSG00000056738 | A730036I17Rik | -3,48 | 6,75389E-08 | 53,76597 | 7,730302 | 18,16522 | 314,2375 | 317,0928 | 252,6888 |
| ENSMUSG00000085139 | A730046J19Rik | -7,87 | 0,026073192 | 0 | 0 | 0 | 9,912855 | 11,41534 | 3,558997 |
| ENSMUSG00000084866 | A930006K02Rik | -1,27 | 0,001582848 | 112,8031 | 105,3254 | 96,01617 | 367,7669 | 180,1087 | 209,0911 |
| ENSMUSG00000097855 | A930007I19Rik | 2,19 | 8,04672E-12 | 137,0505 | 131,4151 | 172,1371 | 35,68628 | 27,90417 | 32,92073 |
| ENSMUSG00000092210 | A930009A15Rik | -5,87 | 1,70916E-05 | 4,216939 | 0,966288 | 0 | 28,74728 | 20,29394 | 243,7913 |
| ENSMUSG00000083307 | AA414768 | 3,17 | 2,49712E-07 | 147,5929 | 409,706 | 275,0734 | 44,60785 | 36,78277 | 11,56674 |
| ENSMUSG00000033213 | AA467197 | -2,52 | 3,39583E-18 | 203,4673 | 123,6848 | 138,4017 | 909,0088 | 1023,576 | 739,3817 |
| ENSMUSG00000084895 | AA672651 | -8,03 | 0,020392699 | 0 | 0 | 0 | 13,878 | 7,610228 | 6,228246 |
| ENSMUSG00000042510 | AA986860 | -2,01 | 6,04251E-07 | 114,9116 | 47,3481 | 72,66089 | 292,4292 | 279,0417 | 369,246 |
| ENSMUSG00000057880 | Abat | -1,74 | 0,000141783 | 89,60995 | 43,48295 | 100,3412 | 230,9695 | 200,4027 | 347,0023 |
| ENSMUSG00000015243 | Abca1 | -1,50 | 2,40764E-06 | 1230,292 | 713,1204 | 1338,171 | 2728,018 | 3414,456 | 3166,618 |
| ENSMUSG00000031974 | Abcb10 | -2,24 | 9,43381E-12 | 4670,26 | 2594,483 | 2814,744 | 17162,13 | 18184,64 | 12290,11 |
| ENSMUSG00000040584 | Abcb1a | 1,98 | 8,38788E-07 | 566,1241 | 372,9871 | 448,0755 | 70,38127 | 114,1534 | 165,4934 |
| ENSMUSG00000028970 | Abcb1b | 1,67 | 1,01273E-06 | 1308,305 | 1324,781 | 1157,384 | 258,7255 | 376,7063 | 552,5344 |
| ENSMUSG00000042476 | Abcb4 | -2,41 | 2,67119E-36 | 1141,736 | 820,3783 | 975,7319 | 5066,46 | 5168,613 | 5350,953 |
| ENSMUSG00000026198 | Abcb6 | -1,72 | 3,00338E-10 | 1242,943 | 727,6147 | 877,9857 | 3134,445 | 2890,618 | 3375,709 |
| ENSMUSG00000029408 | Abcb9 | -1,77 | 3,79741E-05 | 209,7927 | 117,8871 | 254,3131 | 476,8083 | 601,208 | 901,3161 |
| ENSMUSG00000020865 | Abcc3 | -1,58 | 0,004768071 | 214,0097 | 166,2015 | 371,0895 | 631,4488 | 413,4891 | 1198,492 |
| ENSMUSG00000040136 | Abcc8 | -2,59 | 0,003060035 | 2,108469 | 1,932576 | 7,785095 | 27,75599 | 26,6358 | 18,68474 |
| ENSMUSG00000030249 | Abcc9 | -1,38 | 0,024479532 | 261,4502 | 70,53901 | 223,1727 | 327,1242 | 485,7862 | 634,3913 |
| ENSMUSG00000055782 | Abcd2 | 1,87 | 9,02687E-09 | 6299,053 | 10258,11 | 9068,771 | 2538,682 | 2815,784 | 1645,147 |
| ENSMUSG00000029802 | Abcg2 | -2,10 | 3,57895E-15 | 978,3298 | 591,3681 | 773,3195 | 2928,257 | 3325,67 | 3815,245 |
| ENSMUSG00000029299 | Abcg3 | -1,45 | 0,015287446 | 294,1315 | 442,5598 | 572,637 | 883,2353 | 601,208 | 2098,029 |
| ENSMUSG00000032131 | Abcg4 | -3,30 | 1,57195E-07 | 496,5446 | 100,4939 | 128,0216 | 2519,848 | 2766,318 | 1846,23 |
| ENSMUSG00000025277 | Abhd6 | 1,22 | 0,045283696 | 158,1352 | 375,886 | 301,8887 | 95,1634 | 69,76042 | 192,1859 |
| ENSMUSG00000018381 | Abi3 | -1,56 | 0,000121309 | 80,12184 | 57,01098 | 82,17601 | 201,2309 | 147,1311 | 298,9558 |
| ENSMUSG00000035258 | Abi3bp | -2,14 | 0,004067298 | 60,09138 | 16,42689 | 25,95032 | 73,35512 | 117,9585 | 258,9171 |
| ENSMUSG00000025085 | Ablim1 | -1,72 | 0,000134642 | 1900,785 | 670,6037 | 1255,13 | 5274,63 | 3774,673 | 3556,328 |
| ENSMUSG00000030861 | Acadsb | 1,56 | 2,40354E-22 | 3666,628 | 4051,645 | 4099,285 | 1256,95 | 1476,384 | 1276,79 |
| ENSMUSG00000075023 | Accsl | -5,51 | 0,01238958 | 1,054235 | 0 | 0 | 4,956427 | 6,341857 | 32,92073 |
| ENSMUSG00000020681 | Ace | -1,36 | 0,009323361 | 138,1048 | 357,5265 | 410,015 | 974,4336 | 563,1569 | 787,4282 |
| ENSMUSG00000038007 | Acer2 | -1,45 | 0,022103668 | 104,3692 | 85,03333 | 106,3963 | 118,9543 | 201,671 | 484,0237 |
| ENSMUSG00000023328 | Ache | -3,50 | 9,56193E-15 | 490,2192 | 301,4818 | 259,5032 | 2944,118 | 2573,525 | 6343,023 |
| ENSMUSG00000037872 | Ackr1 | -3,09 | 7,09395E-08 | 249,8536 | 52,17954 | 86,50106 | 1042,832 | 1119,972 | 1140,659 |
| ENSMUSG00000026348 | Acmsd | -3,65 | 2,01229E-13 | 55,87444 | 14,49432 | 21,62526 | 362,8105 | 397,0002 | 388,8205 |
| ENSMUSG00000034853 | Acot11 | 1,13 | 7,1186E-08 | 985,7095 | 1014,602 | 888,3659 | 499,6079 | 447,7351 | 371,9152 |
| ENSMUSG00000021751 | Acox2 | 1,95 | 0,026830891 | 10,54235 | 44,44924 | 42,38552 | 6,938998 | 7,610228 | 10,67699 |
| ENSMUSG00000001348 | Acp5 | -2,78 | 3,34703E-11 | 781,1879 | 299,5492 | 765,5344 | 3380,283 | 4503,987 | 4781,513 |
| ENSMUSG00000032561 | Acpp | -1,37 | 1,1697E-05 | 1240,834 | 965,3215 | 1154,789 | 3232,582 | 3477,874 | 1954,779 |
| ENSMUSG00000018796 | Acsl1 | -1,49 | 1,1054E-05 | 4575,379 | 4034,252 | 4611,371 | 12901,58 | 16550,98 | 7800,433 |
| ENSMUSG00000020333 | Acsl6 | -3,90 | 3,75316E-06 | 23,19316 | 1,932576 | 4,325053 | 185,3704 | 162,3515 | 85,41594 |
| ENSMUSG00000027452 | Acss1 | -1,24 | 7,05973E-08 | 1803,796 | 1392,421 | 2013,745 | 4306,144 | 4383,491 | 3653,311 |
| ENSMUSG00000035783 | Acta2 | -2,07 | 4,16862E-07 | 182,3826 | 99,52764 | 134,0766 | 625,5011 | 759,7544 | 361,2382 |
| ENSMUSG00000059430 | Actg2 | -3,10 | 0,002879357 | 11,59658 | 1,932576 | 6,055074 | 15,86057 | 38,05114 | 112,9982 |
| ENSMUSG00000015143 | Actn1 | -1,43 | 1,44947E-09 | 6028,114 | 8481,108 | 5773,946 | 17159,15 | 17682,36 | 19755,99 |
| ENSMUSG00000056367 | Actr3b | -1,72 | 0,003874997 | 15,81352 | 7,730302 | 22,49028 | 60,46841 | 39,31951 | 52,49521 |
| ENSMUSG00000026836 | Acvr1 | 2,19 | 3,81057E-22 | 888,7199 | 723,7496 | 954,9717 | 165,5447 | 183,9138 | 212,6501 |
| ENSMUSG00000052155 | Acvr2a | 1,36 | 1,78214E-10 | 662,0594 | 903,4791 | 806,1899 | 313,2462 | 317,0928 | 294,507 |
| ENSMUSG00000000530 | Acvrl1 | -1,23 | 1,2199E-07 | 1906,056 | 1658,15 | 1377,097 | 3263,312 | 4157,721 | 4150,681 |
| ENSMUSG00000023262 | Acy1 | 1,01 | 3,66133E-06 | 615,6731 | 741,1427 | 819,165 | 347,9412 | 342,4603 | 390,6 |
| ENSMUSG00000020926 | Adam11 | -2,41 | 0,002284486 | 109,6404 | 114,022 | 107,2613 | 245,8388 | 192,7924 | 1322,168 |
| ENSMUSG00000054555 | Adam12 | 2,40 | 1,63441E-09 | 362,6568 | 390,3803 | 440,2904 | 68,3987 | 43,12463 | 112,9982 |
| ENSMUSG00000022039 | Adam2 | -3,08 | 0,011747614 | 2,108469 | 0,966288 | 1,730021 | 8,921569 | 8,878599 | 22,24373 |
| ENSMUSG00000040537 | Adam22 | -1,62 | 0,001784721 | 76,95914 | 76,33674 | 66,60582 | 158,6057 | 138,2525 | 377,2537 |
| ENSMUSG00000027318 | Adam33 | -3,32 | 1,44323E-11 | 20,03046 | 21,25833 | 18,16522 | 235,9259 | 258,7478 | 98,76218 |
| ENSMUSG00000025473 | Adam8 | -1,71 | 0,001044952 | 7724,378 | 5269,167 | 4281,802 | 19197,23 | 28693,1 | 8593,199 |
| ENSMUSG00000059901 | Adamts14 | -1,12 | 0,048156513 | 286,7518 | 132,3814 | 100,3412 | 280,5338 | 357,6807 | 485,8032 |
| ENSMUSG00000053399 | Adamts18 | 4,87 | 0,01974855 | 7,379643 | 12,56174 | 9,515116 | 0 | 0 | 0,889749 |
| ENSMUSG00000036545 | Adamts2 | -1,68 | 0,008983121 | 147,5929 | 103,3928 | 257,7732 | 227,0044 | 530,1792 | 869,2851 |
| ENSMUSG00000043635 | Adamts3 | 2,65 | 1,63405E-16 | 1603,491 | 1446,533 | 1285,406 | 170,5011 | 190,2557 | 327,4278 |
| ENSMUSG00000032363 | Adamts7 | -1,21 | 0,041868058 | 31,62704 | 35,75265 | 54,49567 | 112,0153 | 124,3004 | 48,04647 |
| ENSMUSG00000066113 | Adamtsl1 | 2,64 | 0,000256499 | 46,38633 | 32,85379 | 56,22569 | 3,965142 | 3,805114 | 13,34624 |
| ENSMUSG00000070469 | Adamtsl3 | -1,37 | 0,037157322 | 11,59658 | 38,65151 | 44,98055 | 99,12855 | 83,71251 | 64,06195 |
| ENSMUSG00000056413 | Adap1 | 1,18 | 3,57596E-11 | 6410,801 | 6351,41 | 6620,791 | 3187,974 | 2921,059 | 2452,149 |
| ENSMUSG00000020262 | Adarb1 | -2,10 | 0,000358464 | 82,23031 | 53,14583 | 77,85095 | 144,7277 | 225,7701 | 540,9676 |
| ENSMUSG00000026567 | Adcy10 | -2,51 | 0,015575778 | 5,271174 | 0,966288 | 1,730021 | 14,86928 | 15,22046 | 14,23599 |
| ENSMUSG00000021536 | Adcy2 | -1,75 | 0,023825752 | 34,78975 | 10,62917 | 45,84556 | 49,56427 | 101,4697 | 155,7061 |
| ENSMUSG00000022220 | Adcy4 | -1,03 | 0,000419575 | 88,55572 | 122,7186 | 88,23108 | 226,0131 | 185,1822 | 199,3039 |
| ENSMUSG00000022840 | Adcy5 | -4,13 | 6,62106E-07 | 55,87444 | 42,51666 | 31,14038 | 205,1961 | 270,1631 | 1795,514 |
| ENSMUSG00000022994 | Adcy6 | -2,40 | 2,89418E-09 | 1225,021 | 767,2325 | 949,7816 | 4054,358 | 3501,973 | 7961,477 |
| ENSMUSG00000029106 | Add1 | -1,03 | 5,28542E-12 | 10657,26 | 11484,33 | 10673,37 | 23837,44 | 22848,44 | 20395,72 |
| ENSMUSG00000030000 | Add2 | -3,47 | 2,68295E-08 | 1197,611 | 209,6845 | 381,4697 | 7555,578 | 7153,614 | 5132,074 |
| ENSMUSG00000050994 | Adgb | 1,39 | 0,005678033 | 54,82021 | 96,62878 | 74,39091 | 31,72113 | 17,7572 | 35,58997 |
| ENSMUSG00000031486 | Adgra2 | 2,70 | 3,79239E-12 | 960,4078 | 2160,62 | 1372,772 | 173,475 | 285,3836 | 234,0041 |
| ENSMUSG00000044017 | Adgrd1 | -2,18 | 0,00018603 | 27,4101 | 20,29204 | 17,30021 | 58,48584 | 73,56554 | 160,1549 |
| ENSMUSG00000032915 | Adgre4 | -1,96 | 1,88803E-07 | 351,0602 | 293,7515 | 331,2991 | 1040,85 | 854,8823 | 1912,961 |
| ENSMUSG00000056492 | Adgrf5 | -1,47 | 0,000830037 | 185,5453 | 123,6848 | 202,4125 | 326,1329 | 384,3165 | 710,02 |
| ENSMUSG00000031785 | Adgrg1 | -1,79 | 0,003491203 | 2795,831 | 2647,629 | 3164,209 | 5410,436 | 4956,795 | 19332,47 |
| ENSMUSG00000031298 | Adgrg2 | -2,86 | 0,00146132 | 6,325408 | 2,898863 | 2,595032 | 12,88671 | 30,44091 | 40,92847 |
| ENSMUSG00000039116 | Adgrg6 | 1,56 | 8,80533E-08 | 407,9888 | 646,4465 | 488,731 | 151,6667 | 167,425 | 202,8629 |
| ENSMUSG00000022755 | Adgrg7 | 1,92 | 3,1522E-06 | 1940,846 | 2748,123 | 2160,796 | 343,9761 | 894,2018 | 574,7781 |
| ENSMUSG00000028184 | Adgrl2 | -1,75 | 6,50403E-05 | 234,0401 | 161,3701 | 197,2224 | 450,0436 | 494,6648 | 1041,007 |
| ENSMUSG00000022878 | Adipoq | -1,54 | 0,028644112 | 221,3893 | 175,8644 | 387,5247 | 388,5839 | 426,1728 | 1468,086 |
| ENSMUSG00000018500 | Adora2b | 1,78 | 0,002077671 | 856,0386 | 515,0314 | 976,597 | 209,1612 | 376,7063 | 96,98268 |
| ENSMUSG00000025236 | Adpgk | -1,69 | 8,62718E-07 | 12250,21 | 13301,92 | 9393,15 | 42699,62 | 46190,28 | 23678,9 |
| ENSMUSG00000033717 | Adra2a | -3,81 | 0,000144867 | 20,03046 | 5,797727 | 5,190064 | 44,60785 | 53,2716 | 332,7663 |
| ENSMUSG00000058022 | Adtrp | -1,19 | 0,00010356 | 87,50148 | 95,66249 | 94,28615 | 227,0044 | 248,6008 | 159,2651 |
| ENSMUSG00000029094 | Afap1 | 1,03 | 0,013050066 | 1311,468 | 1339,275 | 1482,628 | 748,4205 | 904,3488 | 379,0332 |
| ENSMUSG00000025083 | Afap1l2 | -1,47 | 0,006769637 | 36,89822 | 30,92121 | 37,19546 | 73,35512 | 62,1502 | 154,8164 |
| ENSMUSG00000031189 | Aff2 | -1,72 | 0,00113463 | 64,30832 | 42,51666 | 64,87579 | 206,1874 | 268,8947 | 91,64418 |
| ENSMUSG00000037138 | Aff3 | -1,61 | 0,000159561 | 887,6657 | 467,6833 | 1195,445 | 3200,861 | 2081,397 | 2514,432 |
| ENSMUSG00000054932 | Afp | -1,87 | 0,024629952 | 11,59658 | 19,32576 | 28,54535 | 45,59913 | 31,70928 | 139,6907 |
| ENSMUSG00000055013 | Agap1 | -1,19 | 0,000226679 | 1800,633 | 2234,057 | 1679,851 | 4859,281 | 5229,495 | 2943,291 |
| ENSMUSG00000040706 | Agmat | -3,59 | 0,003363503 | 3,162704 | 0 | 0,865011 | 19,82571 | 12,68371 | 14,23599 |
| ENSMUSG00000050103 | Agmo | -2,48 | 0,000239276 | 15,81352 | 8,69659 | 13,84017 | 41,63399 | 48,19811 | 123,6752 |
| ENSMUSG00000001211 | Agpat3 | -1,28 | 0,000185264 | 1081,645 | 811,6818 | 1121,919 | 2038,083 | 1891,142 | 3373,93 |
| ENSMUSG00000023827 | Agpat4 | -2,33 | 2,61139E-06 | 446,9955 | 124,6511 | 369,3595 | 1603,9 | 1775,72 | 1336,404 |
| ENSMUSG00000041936 | Agrn | -1,01 | 0,002853262 | 792,7845 | 1177,905 | 717,0938 | 2034,118 | 1982,464 | 1380,001 |
| ENSMUSG00000031980 | Agt | -1,33 | 0,044161941 | 169,7318 | 129,4826 | 273,3433 | 233,9434 | 350,0705 | 855,0491 |
| ENSMUSG00000049115 | Agtr1a | -4,09 | 3,62248E-13 | 79,06761 | 17,39318 | 35,46543 | 949,6515 | 820,6363 | 475,1262 |
| ENSMUSG00000029772 | Ahcyl2 | 1,16 | 2,95839E-08 | 3727,774 | 3758,86 | 4345,813 | 1801,166 | 2014,174 | 1476,984 |
| ENSMUSG00000019986 | Ahi1 | -1,24 | 4,65343E-09 | 404,8261 | 351,7288 | 372,8196 | 743,4641 | 960,1571 | 966,2678 |
| ENSMUSG00000069833 | Ahnak | 1,72 | 5,56503E-11 | 40623,88 | 45133,37 | 35199,01 | 15800,1 | 10546,51 | 10379,82 |
| ENSMUSG00000072812 | Ahnak2 | -1,89 | 0,027495683 | 8,433878 | 36,71894 | 22,49028 | 108,0501 | 115,4218 | 28,47198 |
| ENSMUSG00000019256 | Ahr | 1,13 | 0,03126264 | 1557,105 | 743,0753 | 963,6218 | 396,5142 | 766,0963 | 325,6483 |
| ENSMUSG00000086712 | AI427809 | -2,83 | 1,62855E-05 | 9,488113 | 3,865151 | 5,190064 | 29,73856 | 44,393 | 56,05421 |
| ENSMUSG00000028527 | Ak4 | -1,79 | 1,4274E-06 | 53,76597 | 44,44924 | 57,95571 | 138,78 | 247,3324 | 157,4856 |
| ENSMUSG00000026807 | Ak8 | 2,15 | 2,53514E-21 | 428,0193 | 483,1439 | 512,9513 | 115,9804 | 88,78599 | 114,7777 |
| ENSMUSG00000038587 | Akap12 | -1,74 | 0,04571677 | 1750,03 | 409,706 | 1189,39 | 5297,43 | 5070,949 | 824,7977 |
| ENSMUSG00000021057 | Akap5 | -2,45 | 0,001562908 | 48,4948 | 32,85379 | 58,82072 | 109,0414 | 109,0799 | 548,9754 |
| ENSMUSG00000061603 | Akap6 | -3,40 | 0,001662253 | 2,108469 | 1,932576 | 0,865011 | 15,86057 | 11,41534 | 23,13348 |
| ENSMUSG00000039166 | Akap7 | -1,07 | 0,00024364 | 244,5825 | 364,2905 | 365,0345 | 785,0981 | 570,7671 | 693,1148 |
| ENSMUSG00000049565 | Aknad1 | -3,39 | 0,000680343 | 33,73551 | 0,966288 | 6,055074 | 167,5272 | 152,2046 | 103,2109 |
| ENSMUSG00000052131 | Akr1b7 | -3,43 | 0,008020832 | 2,108469 | 0 | 2,595032 | 13,878 | 7,610228 | 29,36173 |
| ENSMUSG00000033715 | Akr1c14 | -3,46 | 0,008084176 | 3,162704 | 0 | 2,595032 | 10,90414 | 10,14697 | 41,81822 |
| ENSMUSG00000071551 | Akr1c19 | -2,70 | 0,008430994 | 2,108469 | 2,898863 | 3,460042 | 15,86057 | 8,878599 | 30,25148 |
| ENSMUSG00000028393 | Alad | -2,80 | 4,30523E-12 | 5278,553 | 2889,201 | 1992,984 | 23792,83 | 19757,42 | 27043,04 |
| ENSMUSG00000025270 | Alas2 | -3,96 | 2,7276E-17 | 10092,19 | 2819,628 | 5185,738 | 109928,6 | 90694,89 | 80904,02 |
| ENSMUSG00000053279 | Aldh1a1 | -2,37 | 6,08888E-07 | 1706,806 | 479,2787 | 1021,578 | 4997,07 | 6193,457 | 5432,81 |
| ENSMUSG00000024747 | Aldh1a7 | -2,19 | 2,02645E-05 | 211,9012 | 67,64015 | 87,36607 | 509,5207 | 714,0931 | 452,8824 |
| ENSMUSG00000035561 | Aldh1b1 | 4,70 | 9,13089E-31 | 6561,557 | 9121,757 | 11773,66 | 441,122 | 428,7095 | 184,1781 |
| ENSMUSG00000075296 | Aldh3b2 | -2,31 | 1,16041E-10 | 34,78975 | 49,28068 | 34,60042 | 218,0828 | 229,5752 | 142,3599 |
| ENSMUSG00000037263 | Aldh3b3 | -1,62 | 0,000289099 | 48,4948 | 118,8534 | 57,95571 | 250,7952 | 239,7222 | 202,8629 |
| ENSMUSG00000053644 | Aldh7a1 | -1,02 | 0,017192682 | 171,8403 | 173,9318 | 191,1673 | 277,5599 | 262,5529 | 548,9754 |
| ENSMUSG00000028307 | Aldob | -3,18 | 3,87119E-05 | 20,03046 | 3,865151 | 12,97516 | 62,45098 | 71,02879 | 199,3039 |
| ENSMUSG00000000320 | Alox12 | -3,11 | 0,020045602 | 1173,363 | 729,5473 | 1571,724 | 2379,085 | 3211,516 | 24501,92 |
| ENSMUSG00000025701 | Alox5 | -1,00 | 0,007334563 | 3542,229 | 6373,634 | 3682,35 | 9760,197 | 10470,41 | 7039,697 |
| ENSMUSG00000028028 | Alpk1 | 2,21 | 2,57991E-08 | 3060,443 | 6526,308 | 4482,485 | 1212,342 | 1158,023 | 674,43 |
| ENSMUSG00000038763 | Alpk3 | -1,66 | 0,013227614 | 9,488113 | 12,56174 | 15,57019 | 30,72985 | 26,6358 | 61,39271 |
| ENSMUSG00000028766 | Alpl | -1,04 | 0,013605529 | 68,52526 | 53,14583 | 113,3164 | 163,5621 | 157,278 | 161,9344 |
| ENSMUSG00000044037 | Als2cl | -2,23 | 0,000385781 | 112,8031 | 83,10075 | 108,9913 | 375,6972 | 173,7669 | 876,4031 |
| ENSMUSG00000040310 | Alx4 | -3,22 | 0,000926322 | 4,216939 | 0,966288 | 3,460042 | 11,89543 | 29,17254 | 39,14897 |
| ENSMUSG00000045174 | Amer3 | -7,51 | 0,04685291 | 0 | 0 | 0 | 0,991285 | 7,610228 | 10,67699 |
| ENSMUSG00000048218 | Amigo2 | -1,77 | 0,000285052 | 69,57949 | 31,8875 | 25,95032 | 150,6754 | 153,4729 | 128,1239 |
| ENSMUSG00000042225 | Ammecr1 | -1,46 | 6,06297E-05 | 1048,964 | 492,8068 | 727,4739 | 2348,355 | 2201,893 | 1700,311 |
| ENSMUSG00000041688 | Amot | 2,60 | 3,68932E-22 | 1276,678 | 1126,692 | 1293,191 | 197,2658 | 149,6678 | 261,5863 |
| ENSMUSG00000013076 | Amotl1 | -1,09 | 0,014862341 | 87,50148 | 51,21325 | 94,28615 | 132,8323 | 142,0576 | 221,5476 |
| ENSMUSG00000032531 | Amotl2 | -2,92 | 4,52485E-16 | 120,1828 | 65,70757 | 76,12093 | 548,1809 | 556,815 | 876,4031 |
| ENSMUSG00000005686 | Ampd3 | -1,50 | 4,15502E-09 | 2085,276 | 2323,922 | 2044,885 | 6841,852 | 6967,164 | 4465,652 |
| ENSMUSG00000050022 | Amz1 | -1,71 | 3,25881E-09 | 161,2979 | 199,0553 | 203,2775 | 569,9891 | 483,2495 | 785,6487 |
| ENSMUSG00000022309 | Angpt1 | -2,03 | 0,016840949 | 420,6397 | 564,3121 | 736,124 | 749,4118 | 886,5916 | 5395,44 |
| ENSMUSG00000031465 | Angpt2 | -1,41 | 0,011638127 | 66,41679 | 61,84242 | 35,46543 | 86,24184 | 223,2334 | 124,5649 |
| ENSMUSG00000033544 | Angptl1 | -2,50 | 0,000886583 | 7,379643 | 5,797727 | 7,785095 | 26,76471 | 24,09906 | 66,7312 |
| ENSMUSG00000004105 | Angptl2 | 1,06 | 0,006876431 | 199,2504 | 212,5833 | 292,3736 | 91,19826 | 92,59111 | 153,9266 |
| ENSMUSG00000028989 | Angptl7 | -2,57 | 3,20705E-06 | 34,78975 | 39,6178 | 43,25053 | 111,024 | 190,2557 | 397,718 |
| ENSMUSG00000047822 | Angptl8 | 1,73 | 0,009982149 | 29,51857 | 58,94356 | 66,60582 | 17,84314 | 21,56231 | 8,007744 |
| ENSMUSG00000031543 | Ank1 | -3,29 | 1,97215E-07 | 3931,241 | 624,2219 | 1410,832 | 21474,22 | 21200,83 | 15674,71 |
| ENSMUSG00000032826 | Ank2 | -1,26 | 0,022649802 | 17,92199 | 11,59545 | 26,81533 | 43,61656 | 43,12463 | 48,93622 |
| ENSMUSG00000069601 | Ank3 | -2,71 | 0,002328383 | 95,93536 | 42,51666 | 44,98055 | 119,9455 | 152,2046 | 924,4496 |
| ENSMUSG00000024774 | Ankrd22 | -1,18 | 7,17959E-05 | 770,6456 | 1008,804 | 743,0441 | 2057,909 | 2247,554 | 1404,025 |
| ENSMUSG00000022237 | Ankrd33b | -1,58 | 8,27334E-08 | 1139,628 | 1185,635 | 876,2557 | 4007,767 | 3255,909 | 2333,813 |
| ENSMUSG00000038354 | Ankrd35 | -4,54 | 4,02635E-07 | 42,16939 | 19,32576 | 30,27537 | 189,3355 | 191,5241 | 1756,365 |
| ENSMUSG00000040183 | Ankrd6 | -1,48 | 5,11475E-06 | 61,14562 | 41,55038 | 38,92548 | 130,8497 | 129,3739 | 133,4624 |
| ENSMUSG00000078137 | Ankrd63 | -1,93 | 0,025308795 | 13,70505 | 14,49432 | 7,785095 | 57,49456 | 65,95531 | 13,34624 |
| ENSMUSG00000078487 | Ankrd65 | -2,79 | 0,001975751 | 2,108469 | 3,865151 | 1,730021 | 18,83442 | 16,48883 | 17,79499 |
| ENSMUSG00000096140 | Ankrd66 | -2,04 | 0,000640752 | 14,75929 | 8,69659 | 9,515116 | 56,50327 | 27,90417 | 49,82596 |
| ENSMUSG00000037904 | Ankrd9 | -1,54 | 1,3564E-12 | 411,1515 | 343,9985 | 319,1889 | 1181,612 | 923,3743 | 1008,086 |
| ENSMUSG00000035189 | Ano4 | 6,70 | 7,35296E-23 | 178,1657 | 275,392 | 305,3487 | 0,991285 | 5,073485 | 1,779499 |
| ENSMUSG00000039062 | Anpep | -1,18 | 0,004748286 | 219,2808 | 188,4261 | 246,528 | 331,0893 | 734,387 | 416,4027 |
| ENSMUSG00000029338 | Antxr2 | -2,26 | 0,000552971 | 2179,103 | 1052,287 | 961,0268 | 5462,974 | 12189,05 | 2496,637 |
| ENSMUSG00000024659 | Anxa1 | -1,31 | 5,20582E-11 | 21647,66 | 28686,19 | 24680,48 | 65050,13 | 65494,89 | 55184,04 |
| ENSMUSG00000027712 | Anxa5 | 1,21 | 3,08895E-09 | 11824,3 | 13127,99 | 14328,04 | 4905,872 | 5494,585 | 6553,894 |
| ENSMUSG00000018340 | Anxa6 | 1,29 | 1,48848E-17 | 14533,68 | 12965,65 | 14752,76 | 6134,074 | 5367,748 | 5743,332 |
| ENSMUSG00000021950 | Anxa8 | 7,84 | 3,14497E-05 | 15,81352 | 80,20189 | 134,9417 | 0 | 0 | 0,889749 |
| ENSMUSG00000021322 | Aoah | 1,10 | 0,000277479 | 4282,302 | 3858,387 | 4283,532 | 2114,412 | 2355,366 | 1310,601 |
| ENSMUSG00000078651 | Aoc2 | -1,38 | 1,21388E-05 | 453,3209 | 327,5716 | 300,1587 | 1154,848 | 936,058 | 716,2482 |
| ENSMUSG00000038242 | Aox4 | -4,83 | 6,68149E-06 | 0 | 2,898863 | 2,595032 | 74,34641 | 71,02879 | 15,12574 |
| ENSMUSG00000054702 | Ap1s3 | -1,45 | 0,000344492 | 459,6463 | 197,1227 | 314,8639 | 1046,797 | 906,8855 | 704,6815 |
| ENSMUSG00000085611 | Ap3s1-ps1 | 1,16 | 0,004999927 | 103,315 | 110,1568 | 104,6663 | 56,50327 | 57,07671 | 29,36173 |
| ENSMUSG00000024897 | Apba1 | 2,85 | 5,43015E-60 | 5768,773 | 5387,054 | 6413,188 | 862,4184 | 863,7609 | 716,2482 |
| ENSMUSG00000029207 | Apbb2 | -1,82 | 0,001797949 | 66,41679 | 50,24697 | 93,42114 | 135,8061 | 180,1087 | 423,5207 |
| ENSMUSG00000032375 | Aph1b | -1,70 | 2,23083E-08 | 393,2296 | 517,9303 | 439,4254 | 1352,113 | 1935,535 | 1095,281 |
| ENSMUSG00000040613 | Apobec1 | 1,12 | 6,80481E-06 | 5450,394 | 7432,686 | 6650,201 | 3563,671 | 2511,375 | 2881,898 |
| ENSMUSG00000002992 | Apoc2 | 2,90 | 1,76217E-17 | 1218,695 | 822,3109 | 1434,188 | 166,536 | 107,8116 | 189,5166 |
| ENSMUSG00000002985 | Apoe | -1,18 | 0,04392567 | 5816,213 | 4458,452 | 10137,92 | 10025,86 | 10641,64 | 25590,97 |
| ENSMUSG00000056656 | Apol8 | -3,24 | 4,55245E-05 | 642,029 | 48,31439 | 211,9276 | 2833,094 | 3150,634 | 2511,762 |
| ENSMUSG00000004655 | Aqp1 | -3,62 | 1,28461E-10 | 7031,746 | 1524,802 | 2446,25 | 44333,26 | 52362,17 | 38205,84 |
| ENSMUSG00000042797 | Aqp11 | -1,39 | 0,004215403 | 25,30163 | 17,39318 | 35,46543 | 56,50327 | 65,95531 | 82,74669 |
| ENSMUSG00000028435 | Aqp3 | -2,03 | 0,00818255 | 5,271174 | 5,797727 | 3,460042 | 16,85185 | 22,83068 | 19,57449 |
| ENSMUSG00000030762 | Aqp8 | 4,69 | 6,65164E-05 | 18,97623 | 39,6178 | 46,71057 | 3,965142 | 0 | 0 |
| ENSMUSG00000032204 | Aqp9 | -2,28 | 5,13826E-08 | 969,896 | 389,414 | 431,6403 | 3260,338 | 2579,867 | 2865,883 |
| ENSMUSG00000037999 | Arap2 | -1,04 | 1,30782E-06 | 689,4695 | 652,2443 | 602,9124 | 1117,179 | 1357,157 | 1535,707 |
| ENSMUSG00000019852 | Arfgef3 | 1,09 | 0,024904455 | 47,44056 | 61,84242 | 82,17601 | 26,76471 | 38,05114 | 25,80273 |
| ENSMUSG00000021125 | Arg2 | -1,98 | 7,98328E-06 | 434,3447 | 325,639 | 292,3736 | 1461,155 | 1986,27 | 711,7995 |
| ENSMUSG00000030766 | Arhgap17 | 1,30 | 4,23128E-06 | 8762,799 | 13150,21 | 9493,491 | 5094,216 | 3475,337 | 4177,373 |
| ENSMUSG00000063506 | Arhgap22 | -1,74 | 0,007905575 | 13,70505 | 8,69659 | 6,920085 | 22,79957 | 41,85625 | 32,92073 |
| ENSMUSG00000049807 | Arhgap23 | -2,42 | 8,90916E-16 | 1206,045 | 619,3905 | 805,3249 | 4414,194 | 4993,578 | 4669,405 |
| ENSMUSG00000036452 | Arhgap26 | 1,26 | 5,67855E-08 | 4666,043 | 4846,9 | 5444,377 | 2374,129 | 2233,602 | 1623,793 |
| ENSMUSG00000039831 | Arhgap29 | -1,50 | 0,013903793 | 122,2912 | 58,94356 | 122,8315 | 144,7277 | 246,064 | 467,1184 |
| ENSMUSG00000022799 | Arhgap31 | 1,55 | 3,28015E-14 | 7218,345 | 6156,22 | 6334,473 | 2011,318 | 2658,506 | 2041,085 |
| ENSMUSG00000035133 | Arhgap5 | -1,33 | 0,010483613 | 202,4131 | 95,66249 | 166,082 | 291,4379 | 273,9682 | 598,8013 |
| ENSMUSG00000031355 | Arhgap6 | -2,25 | 0,005440066 | 275,1553 | 176,8307 | 307,0788 | 404,4445 | 490,8597 | 2713,736 |
| ENSMUSG00000040964 | Arhgef10l | 1,91 | 1,31148E-16 | 4388,779 | 4450,722 | 5216,879 | 1415,556 | 1347,01 | 966,2678 |
| ENSMUSG00000059495 | Arhgef12 | -2,41 | 2,35042E-20 | 1692,047 | 1042,625 | 1210,15 | 7162,037 | 6121,16 | 7649,175 |
| ENSMUSG00000032875 | Arhgef17 | -2,08 | 0,016192237 | 84,33878 | 117,8871 | 88,23108 | 115,9804 | 176,3036 | 931,5676 |
| ENSMUSG00000019467 | Arhgef25 | -2,58 | 4,83799E-10 | 45,33209 | 45,41553 | 23,35529 | 228,9869 | 162,3515 | 287,389 |
| ENSMUSG00000036885 | Arhgef26 | -2,01 | 0,002450987 | 15,81352 | 6,764015 | 12,11015 | 55,51199 | 58,34508 | 25,80273 |
| ENSMUSG00000021662 | Arhgef28 | 2,62 | 2,11853E-08 | 577,7206 | 773,9965 | 945,4566 | 65,42484 | 109,0799 | 199,3039 |
| ENSMUSG00000045094 | Arhgef37 | 2,38 | 9,59868E-06 | 2590,255 | 2808,999 | 3433,227 | 1037,876 | 379,243 | 276,7121 |
| ENSMUSG00000004562 | Arhgef40 | -2,28 | 4,37868E-11 | 206,63 | 123,6848 | 134,9417 | 624,5098 | 631,6489 | 998,2988 |
| ENSMUSG00000033542 | Arhgef5 | -1,28 | 0,001876275 | 82,23031 | 73,43787 | 96,01617 | 172,4837 | 145,8627 | 291,8378 |
| ENSMUSG00000025870 | Arl10 | 1,37 | 2,76649E-07 | 975,1671 | 1469,724 | 1308,761 | 569,9891 | 438,8565 | 439,5362 |
| ENSMUSG00000047446 | Arl4a | -1,96 | 7,52707E-13 | 810,7065 | 463,8181 | 733,529 | 2602,124 | 2673,727 | 2517,991 |
| ENSMUSG00000062590 | Armc9 | -1,19 | 3,34695E-07 | 319,4331 | 279,2572 | 278,5334 | 676,0567 | 769,9014 | 549,8651 |
| ENSMUSG00000049804 | Armcx4 | 1,45 | 0,000257325 | 666,2764 | 895,7488 | 986,1121 | 239,8911 | 232,112 | 460,8902 |
| ENSMUSG00000018909 | Arrb1 | -1,19 | 1,92439E-13 | 12457,89 | 10506,45 | 10277,19 | 25911,21 | 24274,09 | 25400,56 |
| ENSMUSG00000074794 | Arrdc3 | -1,65 | 2,12555E-11 | 1473,82 | 1416,578 | 1467,058 | 4687,789 | 5582,102 | 3437,992 |
| ENSMUSG00000022620 | Arsa | 1,05 | 4,29941E-10 | 2264,496 | 2363,54 | 2368,399 | 1107,266 | 1007,087 | 1251,877 |
| ENSMUSG00000020604 | Arsg | -1,39 | 1,80115E-05 | 248,7994 | 391,3466 | 446,3455 | 1016,068 | 772,4381 | 1052,574 |
| ENSMUSG00000036412 | Arsi | 2,03 | 0,000802424 | 110,6946 | 287,9538 | 192,8974 | 27,75599 | 81,17577 | 37,36947 |
| ENSMUSG00000030217 | Art4 | -2,90 | 2,08256E-07 | 312,0535 | 64,74128 | 195,4924 | 1645,534 | 1524,582 | 1092,612 |
| ENSMUSG00000028539 | Artn | -3,26 | 2,96211E-13 | 16,86776 | 6,764015 | 9,515116 | 115,9804 | 97,66459 | 99,65193 |
| ENSMUSG00000000325 | Arvcf | -1,65 | 3,113E-06 | 193,9792 | 123,6848 | 173,8671 | 686,9608 | 464,2239 | 387,041 |
| ENSMUSG00000026311 | Asb1 | -1,90 | 4,47146E-12 | 2752,607 | 1601,139 | 1900,428 | 7052,005 | 8343,347 | 7884,069 |
| ENSMUSG00000031382 | Asb11 | 3,11 | 0,000192998 | 18,97623 | 23,19091 | 28,54535 | 2,973856 | 2,536743 | 2,669248 |
| ENSMUSG00000038997 | Asb17 | -4,21 | 2,85066E-11 | 3,162704 | 2,898863 | 4,325053 | 48,57299 | 74,83391 | 70,2902 |
| ENSMUSG00000086438 | Asb17os | -3,76 | 1,7374E-10 | 15,81352 | 1,932576 | 7,785095 | 106,0675 | 120,4953 | 114,7777 |
| ENSMUSG00000040963 | Asgr2 | 1,21 | 0,020904105 | 68,52526 | 55,0784 | 43,25053 | 26,76471 | 15,22046 | 29,36173 |
| ENSMUSG00000029752 | Asns | -1,32 | 3,28452E-15 | 1313,576 | 1174,04 | 1087,318 | 2915,371 | 3183,612 | 2812,498 |
| ENSMUSG00000033508 | Asprv1 | -1,81 | 1,64836E-11 | 361,6025 | 544,02 | 383,1997 | 1255,959 | 1556,292 | 1721,665 |
| ENSMUSG00000076441 | Ass1 | 2,14 | 7,57799E-40 | 5296,475 | 4363,756 | 4856,169 | 1173,682 | 1054,017 | 1075,707 |
| ENSMUSG00000028373 | Astn2 | -4,64 | 0,000146393 | 4,216939 | 0,966288 | 1,730021 | 57,49456 | 106,5432 | 6,228246 |
| ENSMUSG00000026628 | Atf3 | 1,70 | 5,21913E-14 | 1183,906 | 973,0518 | 856,3605 | 307,2985 | 276,505 | 339,8843 |
| ENSMUSG00000099083 | Atf7 | 1,14 | 8,0954E-08 | 6811,411 | 6527,274 | 6419,244 | 3546,819 | 2945,158 | 2465,495 |
| ENSMUSG00000079418 | Atg4a | -1,47 | 9,62172E-16 | 803,3269 | 711,1878 | 776,7795 | 2190,741 | 2326,193 | 1838,222 |
| ENSMUSG00000021066 | Atl1 | -1,67 | 3,22047E-06 | 48,4948 | 57,97727 | 45,84556 | 185,3704 | 186,4506 | 113,8879 |
| ENSMUSG00000037621 | Atoh8 | -3,01 | 1,94892E-05 | 7,379643 | 9,662878 | 4,325053 | 30,72985 | 49,46648 | 90,75444 |
| ENSMUSG00000025324 | Atp10a | 1,50 | 7,09367E-10 | 2907,579 | 3136,57 | 2783,604 | 1158,813 | 1178,317 | 778,5307 |
| ENSMUSG00000046808 | Atp10d | 1,09 | 9,03847E-05 | 3566,476 | 4629,485 | 5528,283 | 2522,822 | 2101,691 | 1832,884 |
| ENSMUSG00000007097 | Atp1a2 | 2,36 | 0,017803477 | 16,86776 | 15,4606 | 13,84017 | 1,982571 | 1,268371 | 5,338496 |
| ENSMUSG00000040907 | Atp1a3 | 1,56 | 4,63885E-08 | 17985,24 | 19308,36 | 20902,12 | 8887,865 | 5213,006 | 5639,231 |
| ENSMUSG00000026576 | Atp1b1 | -1,57 | 0,00015667 | 1586,623 | 624,2219 | 1188,525 | 4014,706 | 3229,273 | 2845,418 |
| ENSMUSG00000041329 | Atp1b2 | -3,54 | 1,33563E-11 | 268,8299 | 74,40416 | 84,77104 | 1584,074 | 1549,95 | 1830,214 |
| ENSMUSG00000026463 | Atp2b4 | -1,06 | 5,85599E-10 | 5322,831 | 4246,835 | 4644,242 | 9633,312 | 9576,204 | 10325,54 |
| ENSMUSG00000005553 | Atp4a | -2,30 | 6,6462E-05 | 7,379643 | 5,797727 | 15,57019 | 45,59913 | 45,66137 | 52,49521 |
| ENSMUSG00000019302 | Atp6v0a1 | -1,29 | 3,25066E-08 | 2600,797 | 3319,199 | 2447,98 | 6551,406 | 5991,786 | 7946,352 |
| ENSMUSG00000038600 | Atp6v0a4 | -3,23 | 0,011064076 | 2,108469 | 1,932576 | 0 | 14,86928 | 10,14697 | 11,56674 |
| ENSMUSG00000006273 | Atp6v1b2 | 1,01 | 2,67965E-09 | 19447,47 | 21892,22 | 20513,73 | 10973,53 | 10658,12 | 8992,697 |
| ENSMUSG00000006567 | Atp7b | -3,24 | 3,5855E-15 | 176,0572 | 67,64015 | 81,31099 | 1149,891 | 1045,138 | 879,0724 |
| ENSMUSG00000039529 | Atp8b1 | -1,82 | 0,004297275 | 41,11515 | 31,8875 | 47,57558 | 72,36384 | 96,39622 | 256,2478 |
| ENSMUSG00000003341 | Atp8b3 | -2,05 | 0,037274084 | 2,108469 | 5,797727 | 3,460042 | 8,921569 | 19,02557 | 19,57449 |
| ENSMUSG00000028457 | Atp8b5 | 2,41 | 0,000739747 | 169,7318 | 602,9636 | 207,6025 | 41,63399 | 34,24603 | 107,6597 |
| ENSMUSG00000054428 | Atpif1 | -1,16 | 6,31505E-06 | 1313,576 | 1057,119 | 1266,375 | 2309,695 | 2420,053 | 3394,394 |
| ENSMUSG00000054843 | Atrnl1 | 1,24 | 2,58924E-07 | 2424,74 | 2832,19 | 3539,623 | 1386,808 | 1241,736 | 1106,848 |
| ENSMUSG00000087377 | AV099323 | -1,54 | 0,003088452 | 10,54235 | 16,42689 | 12,97516 | 43,61656 | 38,05114 | 34,70023 |
| ENSMUSG00000078349 | AW011738 | -1,44 | 1,63531E-05 | 153,9183 | 162,3364 | 87,36607 | 400,4793 | 333,5817 | 362,128 |
| ENSMUSG00000038112 | AW551984 | 3,74 | 0,029552815 | 12,65082 | 9,662878 | 6,920085 | 0 | 2,536743 | 0 |
| ENSMUSG00000002602 | Axl | -1,93 | 0,000112439 | 1541,291 | 1673,61 | 2413,38 | 4926,689 | 4261,728 | 12244,73 |
| ENSMUSG00000037053 | Azgp1 | 7,41 | 0,043084902 | 6,325408 | 10,62917 | 2,595032 | 0 | 0 | 0 |
| ENSMUSG00000097509 | B230322F03Rik | -2,28 | 0,035013209 | 2,108469 | 2,898863 | 3,460042 | 15,86057 | 6,341857 | 18,68474 |
| ENSMUSG00000043300 | B3galnt1 | -1,53 | 0,009869891 | 44,27786 | 33,82007 | 57,95571 | 77,32027 | 96,39622 | 218,8783 |
| ENSMUSG00000074892 | B3galt5 | -1,41 | 0,042464205 | 43,22362 | 16,42689 | 40,6555 | 45,59913 | 87,51762 | 133,4624 |
| ENSMUSG00000026156 | B3gat2 | -1,03 | 0,006416172 | 54,82021 | 81,16818 | 45,84556 | 126,8845 | 129,3739 | 113,8879 |
| ENSMUSG00000031803 | B3gnt3 | -1,82 | 3,88215E-10 | 89,60995 | 57,97727 | 66,60582 | 252,7778 | 281,5784 | 220,6578 |
| ENSMUSG00000074004 | B3gnt6 | 1,25 | 0,034963633 | 28,46434 | 28,02235 | 32,8704 | 14,86928 | 8,878599 | 13,34624 |
| ENSMUSG00000079445 | B3gnt7 | -1,37 | 6,16011E-05 | 111,7489 | 90,83105 | 117,6414 | 200,2397 | 357,6807 | 270,4838 |
| ENSMUSG00000059479 | B3gnt8 | 1,25 | 2,8651E-05 | 1764,789 | 2608,011 | 2445,385 | 1210,36 | 795,2688 | 861,2774 |
| ENSMUSG00000041372 | B4galnt3 | -4,06 | 0,000174995 | 4,216939 | 0 | 0,865011 | 26,76471 | 17,7572 | 36,47972 |
| ENSMUSG00000055629 | B4galnt4 | 1,52 | 0,003591904 | 321,5416 | 200,0216 | 217,1177 | 39,65142 | 104,0064 | 113,8879 |
| ENSMUSG00000022793 | B4galt4 | 3,14 | 1,05539E-24 | 2909,688 | 4659,44 | 3499,833 | 386,6013 | 324,7031 | 541,8574 |
| ENSMUSG00000052364 | B630019K06Rik | 2,24 | 2,76079E-06 | 173,9487 | 231,9091 | 268,1533 | 26,76471 | 39,31951 | 75,6287 |
| ENSMUSG00000032086 | Bace1 | 2,02 | 2,90999E-26 | 6136,7 | 5568,717 | 6466,819 | 1639,586 | 1586,733 | 1255,436 |
| ENSMUSG00000042215 | Bag2 | -1,68 | 7,02162E-21 | 268,8299 | 253,1674 | 288,0485 | 873,3225 | 934,7897 | 789,2077 |
| ENSMUSG00000024232 | Bambi | 3,97 | 1,77186E-71 | 2071,571 | 2379,967 | 2427,22 | 114,9891 | 157,278 | 168,1626 |
| ENSMUSG00000037922 | Bank1 | -2,32 | 1,14074E-13 | 432,2362 | 229,9765 | 388,3898 | 2002,397 | 1505,557 | 1719,886 |
| ENSMUSG00000039699 | Batf2 | 2,46 | 0,000448297 | 513,4123 | 234,8079 | 156,5669 | 29,73856 | 32,97765 | 100,5417 |
| ENSMUSG00000085041 | BB031773 | 9,14 | 0,005962296 | 7,379643 | 35,75265 | 21,62526 | 0 | 0 | 0 |
| ENSMUSG00000063145 | Bbs5 | 1,08 | 0,031386361 | 85,39301 | 200,0216 | 119,3715 | 56,50327 | 58,34508 | 76,51845 |
| ENSMUSG00000033187 | BC016579 | -2,60 | 0,009971505 | 10,54235 | 27,05606 | 8,650106 | 43,61656 | 26,6358 | 209,0911 |
| ENSMUSG00000085006 | BC021767 | -3,62 | 2,06486E-07 | 5,271174 | 2,898863 | 1,730021 | 35,68628 | 40,58788 | 42,70797 |
| ENSMUSG00000038543 | BC028528 | 1,49 | 1,62454E-08 | 410,0973 | 598,1321 | 502,5711 | 198,2571 | 150,9362 | 185,9576 |
| ENSMUSG00000090164 | BC035044 | 1,59 | 6,01229E-22 | 4998,127 | 4925,169 | 4958,241 | 1872,538 | 1541,071 | 1517,023 |
| ENSMUSG00000057246 | BC051142 | -1,66 | 0,008550211 | 17,92199 | 17,39318 | 11,24514 | 49,56427 | 26,6358 | 69,40045 |
| ENSMUSG00000087150 | BC064078 | 1,88 | 4,83799E-10 | 1716,294 | 1113,164 | 1540,584 | 324,1503 | 358,9491 | 506,2674 |
| ENSMUSG00000045231 | BC106179 | -2,57 | 0,039511512 | 2,108469 | 5,797727 | 0 | 13,878 | 12,68371 | 19,57449 |
| ENSMUSG00000094796 | BC147527 | 1,27 | 0,004733572 | 455,4294 | 530,492 | 252,5831 | 229,9782 | 125,5688 | 157,4856 |
| ENSMUSG00000028121 | Bcar3 | -1,56 | 9,15927E-05 | 200,3046 | 127,55 | 208,4676 | 735,5338 | 481,9811 | 366,5767 |
| ENSMUSG00000086199 | Bcas3os1 | -3,53 | 3,72034E-05 | 8,433878 | 3,865151 | 0 | 50,55556 | 41,85625 | 45,37722 |
| ENSMUSG00000000861 | Bcl11a | -1,78 | 5,04336E-14 | 1028,933 | 714,0867 | 755,1542 | 2711,166 | 2635,676 | 3235,129 |
| ENSMUSG00000089929 | Bcl2a1b | -1,90 | 0,005049347 | 11,59658 | 12,56174 | 15,57019 | 26,76471 | 40,58788 | 80,96719 |
| ENSMUSG00000007659 | Bcl2l1 | -2,29 | 3,20065E-18 | 2759,987 | 1579,881 | 2170,312 | 10933,88 | 10018,87 | 10877,19 |
| ENSMUSG00000030200 | Bcl2l14 | 1,71 | 0,000441961 | 119,1285 | 77,30302 | 84,77104 | 17,84314 | 26,6358 | 40,92847 |
| ENSMUSG00000044165 | Bcl2l15 | -2,14 | 1,66242E-05 | 31,62704 | 19,32576 | 12,11015 | 110,0327 | 93,85948 | 72,0697 |
| ENSMUSG00000046598 | Bdh1 | -1,21 | 0,018794924 | 63,25408 | 45,41553 | 115,9114 | 175,4575 | 131,9106 | 212,6501 |
| ENSMUSG00000031872 | Bean1 | -3,47 | 0,000544288 | 3,162704 | 3,865151 | 9,515116 | 24,78214 | 25,36743 | 134,3522 |
| ENSMUSG00000028545 | Bend5 | -2,34 | 1,48751E-05 | 40,06092 | 9,662878 | 32,00539 | 142,7451 | 142,0576 | 128,1239 |
| ENSMUSG00000042182 | Bend6 | -2,16 | 0,010725978 | 7,379643 | 3,865151 | 12,11015 | 18,83442 | 27,90417 | 57,83371 |
| ENSMUSG00000047844 | Bex4 | -3,97 | 1,41086E-15 | 23,19316 | 5,797727 | 11,24514 | 246,8301 | 213,0864 | 164,6036 |
| ENSMUSG00000075269 | Bex6 | 1,92 | 6,95141E-08 | 976,2214 | 841,6367 | 743,9091 | 158,6057 | 191,5241 | 327,4278 |
| ENSMUSG00000032556 | Bfsp2 | -2,10 | 0,002559402 | 67,47102 | 13,52803 | 59,68573 | 288,4641 | 191,5241 | 122,7854 |
| ENSMUSG00000074489 | Bglap3 | -3,19 | 0,030769955 | 2,108469 | 1,932576 | 0 | 9,912855 | 5,073485 | 20,46424 |
| ENSMUSG00000031375 | Bgn | -1,83 | 0,007152357 | 909,8046 | 574,9412 | 1399,587 | 1194,499 | 2979,404 | 6095,673 |
| ENSMUSG00000030103 | Bhlhe40 | -1,22 | 1,61393E-07 | 703,1746 | 486,0428 | 637,5128 | 1516,667 | 1442,138 | 1284,798 |
| ENSMUSG00000003452 | Bicd1 | -2,36 | 0,003255352 | 66,41679 | 36,71894 | 59,68573 | 95,1634 | 135,7157 | 601,4706 |
| ENSMUSG00000043782 | Bicdl2 | -1,86 | 0,007510951 | 14,75929 | 4,831439 | 10,38013 | 46,59042 | 36,78277 | 24,91298 |
| ENSMUSG00000014453 | Blk | -2,06 | 0,000466186 | 891,8826 | 257,9988 | 534,5765 | 3594,401 | 2050,956 | 1353,309 |
| ENSMUSG00000061132 | Blnk | -1,76 | 0,00046368 | 1120,652 | 417,4363 | 834,7352 | 3822,397 | 2540,548 | 1692,303 |
| ENSMUSG00000087330 | Bloodlinc | -2,89 | 4,07051E-07 | 8,433878 | 7,730302 | 6,920085 | 62,45098 | 72,29717 | 36,47972 |
| ENSMUSG00000040466 | Blvrb | -2,60 | 2,33009E-12 | 3223,85 | 1287,095 | 1905,618 | 12969,98 | 13268,43 | 12742,99 |
| ENSMUSG00000040093 | Bmf | 1,39 | 0,004301676 | 2375,191 | 4439,126 | 4314,673 | 1925,076 | 1567,707 | 750,0587 |
| ENSMUSG00000022098 | Bmp1 | -1,19 | 0,000162856 | 160,2437 | 256,0663 | 173,0021 | 374,7059 | 521,3006 | 451,9927 |
| ENSMUSG00000021835 | Bmp4 | -2,93 | 0,000199454 | 15,81352 | 23,19091 | 15,57019 | 79,30284 | 46,92974 | 287,389 |
| ENSMUSG00000039004 | Bmp6 | -1,91 | 7,1448E-05 | 45,33209 | 39,6178 | 46,71057 | 102,1024 | 134,4474 | 258,9171 |
| ENSMUSG00000002384 | Bmp8b | -1,58 | 0,031583497 | 8,433878 | 6,764015 | 6,920085 | 21,80828 | 15,22046 | 28,47198 |
| ENSMUSG00000021796 | Bmpr1a | -1,52 | 4,19141E-12 | 173,9487 | 187,4598 | 147,9168 | 448,061 | 542,8629 | 465,3389 |
| ENSMUSG00000022051 | Bnip3l | -1,67 | 3,37883E-17 | 5838,352 | 4564,744 | 5445,242 | 15351,05 | 19161,29 | 15972,78 |
| ENSMUSG00000073198 | Bnip3l-ps | -1,29 | 0,001861205 | 31,62704 | 23,19091 | 28,54535 | 54,5207 | 71,02879 | 77,40819 |
| ENSMUSG00000045160 | Bola3 | -1,49 | 3,82708E-20 | 562,9614 | 555,6155 | 557,9318 | 1480,98 | 1485,263 | 1738,57 |
| ENSMUSG00000038871 | Bpgm | -3,92 | 7,26288E-37 | 3862,716 | 1978,957 | 2707,483 | 50398,94 | 42774,55 | 36258,18 |
| ENSMUSG00000038286 | Bphl | 1,46 | 5,16527E-08 | 1433,759 | 1636,892 | 1659,09 | 485,7299 | 483,2495 | 747,3895 |
| ENSMUSG00000004031 | Brinp2 | -4,27 | 2,07846E-07 | 5,271174 | 0,966288 | 0,865011 | 49,56427 | 46,92974 | 34,70023 |
| ENSMUSG00000063952 | Brpf3 | -1,64 | 3,75542E-10 | 3845,848 | 2549,067 | 2859,725 | 9716,58 | 11146,45 | 8032,657 |
| ENSMUSG00000035390 | Brsk1 | 2,59 | 1,93953E-07 | 360,5483 | 718,9181 | 415,2051 | 132,8323 | 64,68694 | 49,82596 |
| ENSMUSG00000040859 | Bsdc1 | -1,54 | 2,817E-09 | 4508,962 | 3364,614 | 3634,774 | 12584,37 | 12293,06 | 8533,586 |
| ENSMUSG00000029082 | Bst1 | -2,19 | 2,60225E-10 | 739,0186 | 321,7738 | 521,6014 | 2404,859 | 2321,12 | 2484,18 |
| ENSMUSG00000020423 | Btg2 | -1,17 | 5,97105E-05 | 8720,63 | 5025,663 | 6516,125 | 16736,86 | 15585,75 | 13372,04 |
| ENSMUSG00000000706 | Btn1a1 | -3,66 | 1,47549E-09 | 6,325408 | 1,932576 | 8,650106 | 82,27669 | 79,90739 | 53,38496 |
| ENSMUSG00000020490 | Btnl10 | -3,42 | 7,83038E-08 | 1972,473 | 291,8189 | 735,259 | 11436,46 | 11533,3 | 9178,654 |
| ENSMUSG00000061728 | Btnl7-ps | 1,74 | 0,01733309 | 49,54903 | 62,80871 | 34,60042 | 5,947713 | 12,68371 | 24,91298 |
| ENSMUSG00000025217 | Btrc | -1,58 | 2,98828E-10 | 1098,513 | 795,2549 | 863,2806 | 3134,445 | 2918,522 | 2214,586 |
| ENSMUSG00000071317 | Bves | 3,20 | 5,95912E-06 | 39,00669 | 39,6178 | 71,79588 | 1,982571 | 6,341857 | 8,007744 |
| ENSMUSG00000097365 | C030034L19Rik | 2,14 | 9,45864E-07 | 222,4435 | 434,8295 | 227,4978 | 52,53813 | 57,07671 | 89,86469 |
| ENSMUSG00000087574 | C030037D09Rik | -1,91 | 0,011152048 | 9,488113 | 7,730302 | 6,055074 | 20,81699 | 20,29394 | 45,37722 |
| ENSMUSG00000052477 | C130026I21Rik/C130026I21Rik | -1,91 | 0,008972701 | 157,081 | 56,04469 | 30,27537 | 413,366 | 322,1663 | 175,2806 |
| ENSMUSG00000097557 | C130060C02Rik | -2,40 | 0,000188451 | 11,59658 | 6,764015 | 4,325053 | 50,55556 | 31,70928 | 35,58997 |
| ENSMUSG00000039349 | C130074G19Rik | -1,78 | 0,033542638 | 15,81352 | 27,05606 | 54,49567 | 97,14598 | 40,58788 | 196,6346 |
| ENSMUSG00000048970 | C1galt1c1 | 1,74 | 2,78681E-07 | 5149,937 | 4355,059 | 4777,453 | 1580,109 | 1830,26 | 870,1749 |
| ENSMUSG00000036887 | C1qa | -2,25 | 0,000617052 | 1137,519 | 483,1439 | 1404,777 | 2602,124 | 2766,318 | 9025,618 |
| ENSMUSG00000036905 | C1qb | -1,91 | 0,003580003 | 2217,056 | 800,0863 | 2076,89 | 3229,608 | 4301,047 | 11603,22 |
| ENSMUSG00000036896 | C1qc | -2,07 | 0,000880087 | 1284,058 | 676,4015 | 1624,49 | 2605,098 | 3135,414 | 9299,66 |
| ENSMUSG00000038527 | C1rl | -1,38 | 2,69856E-09 | 454,3752 | 466,717 | 542,3616 | 1299,575 | 1482,726 | 1033,889 |
| ENSMUSG00000079343 | C1s2 | -3,29 | 0,001530682 | 4,216939 | 5,797727 | 2,595032 | 34,69499 | 8,878599 | 78,29794 |
| ENSMUSG00000024371 | C2 | -1,54 | 0,044161933 | 34,78975 | 20,29204 | 32,8704 | 41,63399 | 52,00322 | 161,9344 |
| ENSMUSG00000047990 | C2cd4a | 6,88 | 3,72088E-22 | 198,1961 | 387,4814 | 247,393 | 0,991285 | 1,268371 | 4,448747 |
| ENSMUSG00000097254 | C430042M11Rik | 1,26 | 0,000216649 | 104,3692 | 116,9208 | 143,5918 | 51,54684 | 41,85625 | 58,72346 |
| ENSMUSG00000087365 | C430049B03Rik | -1,13 | 0,030737852 | 13,70505 | 20,29204 | 15,57019 | 33,70371 | 36,78277 | 38,25922 |
| ENSMUSG00000036377 | C530008M17Rik | -2,66 | 3,89519E-12 | 462,8091 | 267,6617 | 421,2602 | 1933,007 | 1809,966 | 3540,313 |
| ENSMUSG00000097838 | C530050E15Rik | -1,31 | 0,048626801 | 36,89822 | 67,64015 | 38,92548 | 119,9455 | 52,00322 | 183,2884 |
| ENSMUSG00000074361 | C5ar2 | -1,23 | 0,009253159 | 325,7585 | 682,1992 | 440,2904 | 1518,649 | 1188,464 | 696,6738 |
| ENSMUSG00000035031 | C8a | -8,18 | 0,019875399 | 0 | 0 | 0 | 7,930284 | 3,805114 | 18,68474 |
| ENSMUSG00000097352 | C920009B18Rik | 1,20 | 0,000907517 | 478,6226 | 964,3552 | 669,5182 | 351,9063 | 298,0673 | 268,7043 |
| ENSMUSG00000080727 | C920021L13Rik | 1,30 | 0,002420359 | 132,8336 | 237,7068 | 143,5918 | 85,25055 | 50,73485 | 72,0697 |
| ENSMUSG00000040957 | Cables1 | -1,53 | 0,014007597 | 186,5995 | 147,842 | 153,9719 | 288,4641 | 885,3232 | 236,6733 |
| ENSMUSG00000028532 | Cachd1 | -2,67 | 1,27704E-08 | 96,9896 | 32,85379 | 64,01078 | 473,8345 | 478,176 | 282,9403 |
| ENSMUSG00000051331 | Cacna1c | -2,39 | 6,01744E-05 | 7,379643 | 5,797727 | 6,055074 | 31,72113 | 30,44091 | 38,25922 |
| ENSMUSG00000015968 | Cacna1d | 1,91 | 7,33661E-08 | 739,0186 | 1074,512 | 989,5721 | 257,7342 | 158,5464 | 328,3175 |
| ENSMUSG00000031142 | Cacna1f | 2,09 | 3,15878E-05 | 160,2437 | 128,5163 | 175,5971 | 58,48584 | 31,70928 | 18,68474 |
| ENSMUSG00000020866 | Cacna1g | -3,75 | 1,31808E-07 | 166,5691 | 24,1572 | 34,60042 | 1177,647 | 1061,627 | 782,9794 |
| ENSMUSG00000022416 | Cacna1i | -3,22 | 6,78168E-09 | 156,0267 | 57,01098 | 57,95571 | 1329,314 | 577,109 | 613,9271 |
| ENSMUSG00000041460 | Cacna2d4 | -1,22 | 0,033078403 | 23,19316 | 28,02235 | 36,33044 | 55,51199 | 45,66137 | 102,3212 |
| ENSMUSG00000032076 | Cadm1 | -1,66 | 0,00033259 | 332,0839 | 266,6954 | 179,0572 | 482,756 | 764,8279 | 1215,398 |
| ENSMUSG00000005338 | Cadm3 | -2,48 | 4,75352E-09 | 129,6709 | 45,41553 | 68,33584 | 454,0087 | 435,0514 | 467,1184 |
| ENSMUSG00000029761 | Cald1 | -3,67 | 3,45033E-06 | 250,9079 | 111,1231 | 289,7785 | 722,6471 | 1429,454 | 6119,696 |
| ENSMUSG00000033033 | Calhm2 | 1,28 | 6,3742E-05 | 2414,198 | 2748,123 | 2882,215 | 1163,769 | 739,4605 | 1418,26 |
| ENSMUSG00000025468 | Caly | 3,25 | 0,039506611 | 5,271174 | 7,730302 | 15,57019 | 1,982571 | 0 | 0,889749 |
| ENSMUSG00000024617 | Camk2a | 1,65 | 1,57943E-13 | 474,4056 | 458,0204 | 490,461 | 125,8933 | 152,2046 | 176,1704 |
| ENSMUSG00000029471 | Camkk2 | 1,14 | 3,40605E-05 | 7923,628 | 9481,216 | 9079,151 | 5136,841 | 3807,651 | 3107,005 |
| ENSMUSG00000038357 | Camp | -1,18 | 0,004844658 | 76926,45 | 110852,5 | 74445,41 | 202743,7 | 275410,3 | 117948,7 |
| ENSMUSG00000044433 | Camsap3 | -2,09 | 0,004841239 | 5,271174 | 16,42689 | 9,515116 | 37,66885 | 26,6358 | 68,5107 |
| ENSMUSG00000021373 | Cap2 | -2,65 | 0,003179419 | 44,27786 | 27,05606 | 19,89524 | 55,51199 | 82,44414 | 432,4182 |
| ENSMUSG00000056737 | Capg | 1,06 | 2,57991E-08 | 14404,01 | 17207,65 | 18184,25 | 8466,569 | 8241,877 | 7109,987 |
| ENSMUSG00000058626 | Capn11 | 5,29 | 0,012946926 | 27,4101 | 50,24697 | 0 | 0,991285 | 0 | 0,889749 |
| ENSMUSG00000079110 | Capn3 | -2,99 | 0,001220381 | 94,88113 | 85,99961 | 83,90603 | 138,78 | 233,3803 | 1729,673 |
| ENSMUSG00000035547 | Capn5 | -2,19 | 1,13397E-07 | 791,7303 | 323,7064 | 576,097 | 1981,58 | 2392,148 | 3357,024 |
| ENSMUSG00000030309 | Caprin2 | -1,01 | 8,55655E-06 | 147,5929 | 170,0667 | 184,2473 | 372,7233 | 313,2877 | 326,538 |
| ENSMUSG00000027556 | Car1 | -4,12 | 7,02658E-16 | 1578,189 | 555,6155 | 457,5906 | 12434,68 | 19039,52 | 13471,7 |
| ENSMUSG00000027562 | Car2 | -3,31 | 2,72717E-09 | 13911,68 | 3129,806 | 5181,413 | 80351,62 | 82516,43 | 57582,8 |
| ENSMUSG00000041261 | Car8 | 3,53 | 7,03114E-13 | 78,01337 | 90,83105 | 88,23108 | 6,938998 | 3,805114 | 10,67699 |
| ENSMUSG00000036526 | Card11 | -1,98 | 1,21641E-05 | 741,127 | 429,9981 | 544,0917 | 1906,242 | 1340,669 | 3534,974 |
| ENSMUSG00000041849 | Card6 | -1,00 | 3,40605E-05 | 1023,662 | 1122,826 | 1209,285 | 2667,549 | 2272,921 | 1794,624 |
| ENSMUSG00000008393 | Carhsp1 | -2,04 | 5,42685E-14 | 3973,411 | 2665,022 | 2764,574 | 15000,13 | 13404,15 | 10387,82 |
| ENSMUSG00000021338 | Carmil1 | -1,34 | 0,000149075 | 228,7689 | 212,5833 | 206,7375 | 524,39 | 364,0226 | 746,4997 |
| ENSMUSG00000050357 | Carmil2 | -1,32 | 2,01896E-06 | 547,1478 | 434,8295 | 506,0312 | 1601,917 | 1041,333 | 1073,927 |
| ENSMUSG00000075289 | Carns1 | -1,03 | 0,008940175 | 321,5416 | 549,8178 | 397,0399 | 1148,9 | 618,9652 | 827,4669 |
| ENSMUSG00000060227 | Casc4 | -2,19 | 0,000193148 | 74,85067 | 31,8875 | 95,15116 | 233,9434 | 194,0608 | 494,7006 |
| ENSMUSG00000031012 | Cask | 1,20 | 0,000212633 | 2003,046 | 2361,607 | 2417,705 | 1036,885 | 1255,688 | 655,7453 |
| ENSMUSG00000074570 | Cass4 | -2,38 | 0,00113463 | 176,0572 | 161,3701 | 149,6468 | 317,2113 | 383,0481 | 1836,443 |
| ENSMUSG00000027187 | Cat | -1,31 | 3,17999E-12 | 5304,909 | 4023,622 | 4991,976 | 12292,93 | 12014,01 | 11147,67 |
| ENSMUSG00000049676 | Catsperg1 | 1,61 | 0,019380388 | 137,0505 | 238,6731 | 104,6663 | 44,60785 | 21,56231 | 89,86469 |
| ENSMUSG00000004044 | Cavin1 | -2,88 | 0,000465697 | 500,7615 | 188,4261 | 403,9599 | 718,682 | 1312,764 | 6016,485 |
| ENSMUSG00000045954 | Cavin2 | -1,99 | 0,036010569 | 992,0349 | 756,6033 | 1699,746 | 1105,283 | 1705,959 | 10859,39 |
| ENSMUSG00000037060 | Cavin3 | -2,40 | 0,004604543 | 53,76597 | 35,75265 | 46,71057 | 69,38998 | 111,6167 | 536,5189 |
| ENSMUSG00000006362 | Cbfa2t3 | 1,07 | 7,34075E-08 | 4579,596 | 5770,671 | 5463,407 | 2823,181 | 2305,899 | 2386,308 |
| ENSMUSG00000031654 | Cbln1 | -1,57 | 0,015246804 | 35,84398 | 19,32576 | 19,89524 | 40,6427 | 64,68694 | 115,6674 |
| ENSMUSG00000040380 | Cbln3 | -2,04 | 0,016986203 | 15,81352 | 3,865151 | 15,57019 | 47,5817 | 21,56231 | 74,73895 |
| ENSMUSG00000025150 | Cbr2 | 1,96 | 9,1979E-08 | 317,3247 | 405,8409 | 404,825 | 118,9543 | 55,80834 | 112,9982 |
| ENSMUSG00000053411 | Cbx7 | -1,02 | 0,000813229 | 1150,17 | 950,8272 | 938,5365 | 2719,096 | 1749,084 | 1674,508 |
| ENSMUSG00000020930 | Ccdc103 | -3,56 | 0,000332487 | 2,108469 | 0,966288 | 1,730021 | 17,84314 | 19,02557 | 19,57449 |
| ENSMUSG00000050786 | Ccdc126 | -1,16 | 4,18262E-07 | 314,162 | 281,1898 | 276,8034 | 772,2114 | 568,2304 | 603,2501 |
| ENSMUSG00000079235 | Ccdc13 | 5,86 | 0,002167323 | 12,65082 | 28,98863 | 17,30021 | 0 | 0 | 0,889749 |
| ENSMUSG00000044033 | Ccdc141 | -2,25 | 1,18272E-06 | 15,81352 | 9,662878 | 16,4352 | 54,5207 | 65,95531 | 79,18769 |
| ENSMUSG00000045790 | Ccdc149 | -2,02 | 0,000131837 | 25,30163 | 13,52803 | 10,38013 | 70,38127 | 76,10228 | 51,60546 |
| ENSMUSG00000025983 | Ccdc150 | -3,82 | 1,17338E-06 | 12,65082 | 13,52803 | 10,38013 | 91,19826 | 57,07671 | 366,5767 |
| ENSMUSG00000075225 | Ccdc162 | -3,68 | 6,28985E-06 | 5,271174 | 11,59545 | 10,38013 | 37,66885 | 78,63902 | 234,0041 |
| ENSMUSG00000035539 | Ccdc180 | 1,45 | 0,042329533 | 95,93536 | 193,2576 | 328,704 | 115,9804 | 74,83391 | 36,47972 |
| ENSMUSG00000057176 | Ccdc189 | -2,64 | 2,48443E-16 | 99,09807 | 77,30302 | 76,12093 | 478,7909 | 700,141 | 395,9385 |
| ENSMUSG00000078588 | Ccdc24 | -2,71 | 0,00666892 | 6,325408 | 2,898863 | 1,730021 | 36,67756 | 10,14697 | 23,13348 |
| ENSMUSG00000039492 | Ccdc27 | -3,22 | 2,76224E-05 | 11,59658 | 2,898863 | 2,595032 | 73,35512 | 41,85625 | 40,03872 |
| ENSMUSG00000043036 | Ccdc63 | -1,34 | 0,000966156 | 42,16939 | 39,6178 | 37,19546 | 113,9978 | 119,2269 | 68,5107 |
| ENSMUSG00000038903 | Ccdc68 | -3,34 | 6,59755E-05 | 2,108469 | 3,865151 | 2,595032 | 16,85185 | 36,78277 | 33,81048 |
| ENSMUSG00000041617 | Ccdc74a | -1,32 | 0,003208785 | 121,237 | 55,0784 | 63,14577 | 213,1264 | 223,2334 | 160,1549 |
| ENSMUSG00000041117 | Ccdc8 | -1,25 | 0,042846671 | 162,3522 | 216,4485 | 196,3574 | 233,9434 | 303,1407 | 831,0259 |
| ENSMUSG00000022665 | Ccdc80 | -1,23 | 0,007152357 | 63,25408 | 55,0784 | 124,5615 | 185,3704 | 216,8915 | 168,1626 |
| ENSMUSG00000037979 | Ccdc92 | -1,90 | 0,019541898 | 112,8031 | 62,80871 | 131,4816 | 156,6231 | 164,8883 | 827,4669 |
| ENSMUSG00000069814 | Ccdc92b | -3,37 | 4,89965E-07 | 282,5349 | 47,3481 | 88,23108 | 1946,885 | 1473,847 | 906,6546 |
| ENSMUSG00000032532 | Cck | 7,92 | 0,028848758 | 8,433878 | 1,932576 | 17,30021 | 0 | 0 | 0 |
| ENSMUSG00000004814 | Ccl24 | 2,25 | 3,11623E-05 | 34,78975 | 40,58409 | 42,38552 | 7,930284 | 8,878599 | 8,007744 |
| ENSMUSG00000000982 | Ccl3 | 1,49 | 0,000370342 | 345,789 | 687,9969 | 563,1219 | 179,4227 | 134,4474 | 253,5786 |
| ENSMUSG00000019122 | Ccl9 | 2,85 | 9,96658E-34 | 20868,58 | 19647,53 | 25530,79 | 3164,183 | 3599,638 | 2429,016 |
| ENSMUSG00000071470 | Ccnb1ip1 | -2,12 | 5,78661E-06 | 93,82689 | 65,70757 | 44,11554 | 435,1743 | 225,7701 | 220,6578 |
| ENSMUSG00000070348 | Ccnd1 | -1,68 | 3,52068E-05 | 516,575 | 218,381 | 301,8887 | 1085,458 | 906,8855 | 1329,286 |
| ENSMUSG00000023572 | Ccndbp1 | -1,19 | 1,71414E-22 | 2569,17 | 2709,471 | 2601,087 | 6081,536 | 5833,24 | 6011,147 |
| ENSMUSG00000002068 | Ccne1 | -1,55 | 1,21049E-06 | 1533,912 | 783,6594 | 1216,205 | 3516,09 | 3760,721 | 3047,392 |
| ENSMUSG00000049103 | Ccr2 | 2,75 | 1,83477E-14 | 30375,67 | 69414,25 | 45091,27 | 7232,419 | 8320,516 | 5977,336 |
| ENSMUSG00000035448 | Ccr3 | -2,22 | 0,004833448 | 443,8328 | 56,04469 | 116,7764 | 808,8889 | 750,8758 | 1307,042 |
| ENSMUSG00000040899 | Ccr6 | -2,75 | 5,00479E-11 | 44,27786 | 20,29204 | 21,62526 | 179,4227 | 163,6199 | 232,2246 |
| ENSMUSG00000037944 | Ccr7 | -2,39 | 7,97599E-06 | 312,0535 | 79,2356 | 196,3574 | 1065,632 | 739,4605 | 1277,68 |
| ENSMUSG00000029530 | Ccr9 | -1,95 | 0,002544136 | 331,0297 | 161,3701 | 312,2688 | 561,0676 | 568,2304 | 1986,81 |
| ENSMUSG00000043953 | Ccrl2 | -1,69 | 0,001482013 | 522,9004 | 187,4598 | 212,7926 | 860,4358 | 1362,231 | 752,728 |
| ENSMUSG00000086564 | Cd101 | -1,67 | 0,000695627 | 537,6597 | 1095,77 | 539,7666 | 3466,525 | 1470,042 | 1993,928 |
| ENSMUSG00000030724 | Cd19 | -2,03 | 0,000637577 | 2451,096 | 795,2549 | 1654,765 | 10763,38 | 5944,856 | 3376,599 |
| ENSMUSG00000027863 | Cd2 | -1,57 | 0,000289538 | 508,1411 | 203,8867 | 263,8282 | 1062,658 | 776,2433 | 1065,03 |
| ENSMUSG00000022661 | Cd200 | -1,89 | 3,63799E-06 | 108,5862 | 63,77499 | 123,6965 | 298,3769 | 295,5305 | 501,8186 |
| ENSMUSG00000090176 | Cd200r2 | 2,48 | 0,002872855 | 72,7422 | 28,98863 | 22,49028 | 3,965142 | 11,41534 | 7,117995 |
| ENSMUSG00000036172 | Cd200r3 | -2,05 | 0,000547393 | 73,79643 | 136,2466 | 28,54535 | 330,0981 | 282,8468 | 370,1357 |
| ENSMUSG00000062082 | Cd200r4 | 1,66 | 1,21352E-06 | 547,1478 | 604,8962 | 525,0614 | 135,8061 | 138,2525 | 253,5786 |
| ENSMUSG00000065987 | Cd209b | -4,16 | 0,00818255 | 1,054235 | 0 | 0,865011 | 9,912855 | 16,48883 | 8,007744 |
| ENSMUSG00000040165 | Cd209c | -1,55 | 0,019431459 | 17,92199 | 50,24697 | 22,49028 | 53,52942 | 83,71251 | 128,1239 |
| ENSMUSG00000031495 | Cd209d | -2,23 | 4,96502E-05 | 107,5319 | 77,30302 | 51,90064 | 191,3181 | 310,751 | 605,9193 |
| ENSMUSG00000051906 | Cd209f | -7,83 | 3,66913E-05 | 0 | 0 | 0,865011 | 154,6405 | 22,83068 | 36,47972 |
| ENSMUSG00000079168 | Cd209g | -8,61 | 0,031519603 | 0 | 0 | 0 | 38,66013 | 0 | 2,669248 |
| ENSMUSG00000030577 | Cd22 | -1,30 | 1,78305E-05 | 1562,376 | 1619,498 | 1316,546 | 4976,253 | 3151,903 | 2921,937 |
| ENSMUSG00000034028 | Cd226 | -2,79 | 0,000599229 | 234,0401 | 169,1004 | 267,2883 | 445,0872 | 612,6234 | 3568,785 |
| ENSMUSG00000056481 | Cd248 | -3,08 | 0,000186485 | 2,108469 | 3,865151 | 2,595032 | 17,84314 | 25,36743 | 29,36173 |
| ENSMUSG00000047139 | Cd24a | -1,48 | 1,33304E-11 | 28108,01 | 25703,26 | 24598,31 | 77873,4 | 83665,58 | 57877,31 |
| ENSMUSG00000026012 | Cd28 | 4,24 | 6,40132E-24 | 4652,338 | 8707,219 | 5565,478 | 217,0915 | 270,1631 | 515,1649 |
| ENSMUSG00000058728 | Cd300c | -2,96 | 1,721E-05 | 79,06761 | 63,77499 | 59,68573 | 246,8301 | 221,965 | 1105,069 |
| ENSMUSG00000044811 | Cd300c2 | -1,12 | 5,75086E-06 | 676,8187 | 843,5693 | 863,2806 | 1506,754 | 1585,464 | 2104,257 |
| ENSMUSG00000048498 | Cd300e | -2,34 | 0,000790391 | 102,2608 | 61,84242 | 66,60582 | 201,2309 | 172,4985 | 790,9872 |
| ENSMUSG00000034641 | Cd300ld | -2,07 | 6,57957E-10 | 1242,943 | 847,4344 | 849,4404 | 4107,887 | 5384,236 | 2840,97 |
| ENSMUSG00000069607 | Cd300ld3 | -2,00 | 0,00206703 | 6,325408 | 11,59545 | 8,650106 | 32,71242 | 49,46648 | 24,91298 |
| ENSMUSG00000017309 | Cd300lg | 1,49 | 6,56193E-12 | 1343,095 | 1442,668 | 1762,892 | 585,8497 | 465,4923 | 564,9908 |
| ENSMUSG00000004609 | Cd33 | -1,05 | 0,017538375 | 3029,871 | 7314,799 | 3832,862 | 10468,97 | 10630,22 | 8229,292 |
| ENSMUSG00000002944 | Cd36 | -3,17 | 3,14037E-09 | 886,6114 | 215,4822 | 365,0345 | 3825,371 | 5550,393 | 3849,946 |
| ENSMUSG00000029084 | Cd38 | -1,99 | 1,13012E-05 | 432,2362 | 147,842 | 398,7699 | 1145,926 | 1277,25 | 1470,756 |
| ENSMUSG00000032094 | Cd3d | -1,04 | 0,018265799 | 406,9346 | 468,6496 | 189,4373 | 670,109 | 807,9525 | 711,7995 |
| ENSMUSG00000023274 | Cd4 | -2,63 | 8,63223E-07 | 350,0059 | 268,628 | 469,7007 | 1091,405 | 1688,202 | 3970,062 |
| ENSMUSG00000017652 | Cd40 | 1,13 | 0,013987046 | 642,029 | 1457,162 | 887,5009 | 599,7277 | 339,9235 | 427,0797 |
| ENSMUSG00000015355 | Cd48 | 1,47 | 1,66654E-10 | 7167,742 | 8189,289 | 8061,034 | 2707,201 | 2298,289 | 3451,338 |
| ENSMUSG00000026399 | Cd55 | -2,40 | 1,93483E-15 | 612,5104 | 420,3352 | 414,3401 | 2769,652 | 1855,627 | 3012,691 |
| ENSMUSG00000032679 | Cd59a | -3,17 | 1,999E-13 | 144,4302 | 79,2356 | 118,5065 | 628,475 | 894,2018 | 1544,605 |
| ENSMUSG00000068686 | Cd59b | -3,31 | 7,75287E-09 | 22,13893 | 4,831439 | 11,24514 | 92,18955 | 126,8371 | 155,7061 |
| ENSMUSG00000015854 | Cd5l | -3,45 | 1,61511E-06 | 473,3514 | 234,8079 | 218,8477 | 1751,601 | 1182,122 | 7225,655 |
| ENSMUSG00000024670 | Cd6 | -1,40 | 0,036607501 | 210,8469 | 59,90984 | 89,9611 | 232,9521 | 229,5752 | 486,6929 |
| ENSMUSG00000028459 | Cd72 | -1,60 | 0,009234679 | 994,1434 | 368,1557 | 964,4868 | 3709,39 | 2147,353 | 1172,69 |
| ENSMUSG00000003379 | Cd79a | -2,01 | 0,001733467 | 3355,629 | 739,2102 | 1963,574 | 11148,99 | 8780,935 | 4405,149 |
| ENSMUSG00000040592 | Cd79b | -1,70 | 0,00088159 | 3683,496 | 1477,454 | 3047,432 | 12934,29 | 8651,561 | 5156,098 |
| ENSMUSG00000075122 | Cd80 | 1,69 | 5,45467E-05 | 1324,119 | 983,681 | 1105,484 | 297,3856 | 545,3997 | 216,2091 |
| ENSMUSG00000037706 | Cd81 | -1,53 | 4,14275E-11 | 1735,27 | 1450,398 | 1846,798 | 4026,602 | 4723,415 | 5762,907 |
| ENSMUSG00000027215 | Cd82 | -1,60 | 1,34382E-15 | 4572,216 | 3270,884 | 3604,499 | 11331,38 | 11804,73 | 11526,7 |
| ENSMUSG00000015396 | Cd83 | -2,49 | 2,26241E-05 | 344,7348 | 77,30302 | 211,9276 | 784,1068 | 1142,803 | 1626,462 |
| ENSMUSG00000022901 | Cd86 | -1,44 | 0,001907117 | 336,3009 | 212,5833 | 265,5582 | 405,4358 | 721,7033 | 1078,376 |
| ENSMUSG00000030342 | Cd9 | -1,96 | 1,51786E-08 | 4642,85 | 4214,947 | 3322,506 | 11805,22 | 12758,55 | 22826,52 |
| ENSMUSG00000027435 | Cd93 | 1,38 | 3,17885E-05 | 20165,4 | 19006,88 | 20377,05 | 8765,937 | 9418,926 | 4748,592 |
| ENSMUSG00000092193 | Cd9-ps | -1,98 | 0,000102345 | 12,65082 | 16,42689 | 10,38013 | 45,59913 | 43,12463 | 65,84145 |
| ENSMUSG00000027330 | Cdc25b | -1,80 | 4,06478E-09 | 4450,979 | 2470,798 | 3324,236 | 14405,36 | 10942,24 | 10439,43 |
| ENSMUSG00000026490 | Cdc42bpa | -2,26 | 5,94415E-14 | 324,7043 | 246,4034 | 245,663 | 1147,909 | 1029,918 | 1738,57 |
| ENSMUSG00000045664 | Cdc42ep2 | -1,38 | 0,005651088 | 364,7652 | 302,4481 | 373,6846 | 568,9979 | 613,8917 | 1531,259 |
| ENSMUSG00000063838 | Cdc42ep5 | -4,04 | 3,85023E-05 | 28,46434 | 7,730302 | 21,62526 | 79,30284 | 92,59111 | 779,4204 |
| ENSMUSG00000035498 | Cdcp1 | -2,43 | 0,011178098 | 9,488113 | 12,56174 | 19,89524 | 29,73856 | 27,90417 | 169,0524 |
| ENSMUSG00000000303 | Cdh1 | -2,42 | 1,35298E-10 | 180,2741 | 150,7409 | 77,85095 | 617,5708 | 702,6777 | 856,8286 |
| ENSMUSG00000031673 | Cdh11 | -1,79 | 0,00909113 | 124,3997 | 80,20189 | 152,2419 | 156,6231 | 304,4091 | 776,7512 |
| ENSMUSG00000059674 | Cdh24 | -1,03 | 0,007474859 | 93,82689 | 56,04469 | 83,90603 | 185,3704 | 168,6934 | 123,6752 |
| ENSMUSG00000031871 | Cdh5 | -1,72 | 0,00148418 | 298,3484 | 139,1454 | 249,9881 | 396,5142 | 676,0419 | 1194,933 |
| ENSMUSG00000040274 | Cdk6 | 1,51 | 7,85831E-17 | 4460,467 | 4619,822 | 5218,609 | 1506,754 | 1613,368 | 1886,269 |
| ENSMUSG00000020990 | Cdkl1 | -3,19 | 5,93994E-09 | 110,6946 | 23,19091 | 50,17061 | 571,9717 | 624,0387 | 480,4647 |
| ENSMUSG00000023067 | Cdkn1a | 1,33 | 0,018429395 | 3018,274 | 2305,563 | 3343,266 | 1741,689 | 1245,541 | 451,9927 |
| ENSMUSG00000037664 | Cdkn1c | 2,09 | 0,000216649 | 502,87 | 158,4712 | 518,1413 | 63,44227 | 91,32274 | 121,8957 |
| ENSMUSG00000073802 | Cdkn2b | 2,52 | 0,000640147 | 29,51857 | 39,6178 | 92,55613 | 6,938998 | 7,610228 | 13,34624 |
| ENSMUSG00000030878 | Cdr2 | -3,10 | 3,91383E-14 | 2364,649 | 897,6814 | 1137,489 | 13509,24 | 13591,87 | 10645,85 |
| ENSMUSG00000029330 | Cds1 | 1,69 | 2,80016E-19 | 1276,678 | 1205,927 | 1340,766 | 362,8105 | 364,0226 | 454,6619 |
| ENSMUSG00000039518 | Cdsn | 4,68 | 0,035847278 | 10,54235 | 3,865151 | 11,24514 | 0 | 0 | 0,889749 |
| ENSG00000165556 | CDX2 | 6,81 | 2,68502E-16 | 160,2437 | 149,7746 | 158,2969 | 0 | 5,073485 | 0 |
| ENSMUSG00000054169 | Ceacam10 | -1,08 | 0,022398843 | 295,1857 | 784,6257 | 474,0258 | 1220,272 | 866,2976 | 1191,374 |
| ENSMUSG00000034957 | Cebpa | 1,72 | 7,78903E-14 | 6788,218 | 6682,846 | 9540,202 | 2467,31 | 2403,564 | 2114,934 |
| ENSMUSG00000071226 | Cecr2 | -2,13 | 0,007474859 | 1953,497 | 530,492 | 1324,331 | 10118,05 | 5095,048 | 1463,638 |
| ENSMUSG00000023031 | Cela1 | -2,73 | 1,28944E-20 | 203,4673 | 126,5837 | 181,6522 | 877,2876 | 1197,343 | 1312,38 |
| ENSMUSG00000028137 | Celf3 | 1,33 | 0,049719025 | 25,30163 | 73,43787 | 43,25053 | 22,79957 | 17,7572 | 16,01549 |
| ENSMUSG00000026605 | Cenpf | -1,00 | 0,021005949 | 5206,865 | 2426,349 | 4234,227 | 7934,249 | 10101,31 | 5759,348 |
| ENSMUSG00000018509 | Cenpv | -1,73 | 3,40338E-16 | 170,786 | 167,1678 | 172,1371 | 482,756 | 573,3038 | 641,5093 |
| ENSMUSG00000020728 | Cep112 | 2,21 | 0,000162059 | 56,92868 | 59,90984 | 70,93087 | 8,921569 | 7,610228 | 23,13348 |
| ENSMUSG00000040729 | Cep126 | -3,16 | 7,33167E-05 | 10,54235 | 5,797727 | 5,190064 | 30,72985 | 40,58788 | 118,3367 |
| ENSMUSG00000073542 | Cep76 | -1,11 | 1,37224E-05 | 1591,894 | 1025,231 | 1313,951 | 2962,952 | 3039,018 | 2508,203 |
| ENSMUSG00000056973 | Ces1d | -5,24 | 6,92387E-09 | 9,488113 | 0,966288 | 6,055074 | 231,9608 | 342,4603 | 45,37722 |
| ENSMUSG00000031886 | Ces2e | -2,78 | 0,00192264 | 5,271174 | 3,865151 | 6,920085 | 20,81699 | 19,02557 | 70,2902 |
| ENSMUSG00000031877 | Ces2g | -3,57 | 1,24833E-12 | 889,7741 | 237,7068 | 336,4891 | 5543,268 | 6411,617 | 5470,179 |
| ENSMUSG00000045031 | Cetn4 | -1,66 | 0,000567517 | 11,59658 | 13,52803 | 18,16522 | 43,61656 | 50,73485 | 43,59772 |
| ENSMUSG00000028730 | Cfap57 | 2,72 | 1,87887E-12 | 364,7652 | 346,8973 | 281,1284 | 74,34641 | 45,66137 | 31,14123 |
| ENSMUSG00000026365 | Cfh | -1,99 | 8,90758E-06 | 200,3046 | 114,9882 | 258,6382 | 477,7996 | 797,8056 | 1011,645 |
| ENSMUSG00000001128 | Cfp | 1,04 | 8,13428E-09 | 16882,52 | 16630,78 | 20454,91 | 8526,046 | 8282,465 | 9461,595 |
| ENSMUSG00000032232 | Cgnl1 | 2,39 | 2,21403E-07 | 304,6738 | 419,3689 | 749,9642 | 96,15469 | 63,41857 | 121,0059 |
| ENSMUSG00000027313 | Chac1 | -1,49 | 4,1377E-05 | 93,82689 | 78,26931 | 86,50106 | 291,4379 | 279,0417 | 157,4856 |
| ENSMUSG00000020309 | Chac2 | -2,20 | 2,38523E-10 | 346,8432 | 159,4375 | 237,0129 | 1305,523 | 1118,704 | 995,6295 |
| ENSMUSG00000049422 | Chchd10 | -1,26 | 0,003179419 | 694,7407 | 372,0208 | 662,5981 | 1883,442 | 1291,202 | 954,7011 |
| ENSMUSG00000005045 | Chd5 | 1,96 | 1,45336E-06 | 65,36255 | 85,03333 | 94,28615 | 23,79085 | 16,48883 | 22,24373 |
| ENSMUSG00000064246 | Chil1 | -2,01 | 1,04681E-05 | 4888,486 | 3266,053 | 2288,818 | 20712,91 | 8579,264 | 12884,46 |
| ENSMUSG00000040809 | Chil3 | -2,79 | 1,08763E-08 | 25476,64 | 12763,7 | 19806,15 | 130083,4 | 205755,2 | 64739,05 |
| ENSMUSG00000043873 | Chil5 | -1,86 | 4,2717E-15 | 138,1048 | 150,7409 | 108,1263 | 496,634 | 517,4955 | 428,8592 |
| ENSMUSG00000026450 | Chit1 | -2,79 | 0,003131272 | 70,63373 | 4,831439 | 13,84017 | 293,4205 | 140,7892 | 180,6191 |
| ENSMUSG00000078185 | Chml | 1,36 | 3,73645E-08 | 695,7949 | 716,9855 | 605,5074 | 294,4118 | 289,1887 | 204,6424 |
| ENSMUSG00000004633 | Chn2 | 2,12 | 3,02123E-07 | 1072,157 | 1368,264 | 1590,754 | 220,0654 | 221,965 | 485,8032 |
| ENSMUSG00000030865 | Chp2 | -2,90 | 0,000835558 | 9,488113 | 18,35947 | 83,04102 | 377,6798 | 126,8371 | 327,4278 |
| ENSMUSG00000031283 | Chrdl1 | -2,39 | 0,001845744 | 119,1285 | 65,70757 | 179,9222 | 183,3878 | 474,3709 | 1260,775 |
| ENSMUSG00000032773 | Chrm1 | -1,94 | 0,010654882 | 12,65082 | 4,831439 | 5,190064 | 22,79957 | 39,31951 | 24,02323 |
| ENSMUSG00000046159 | Chrm3 | -2,22 | 2,6684E-05 | 36,89822 | 55,0784 | 41,52051 | 212,1351 | 313,2877 | 97,87243 |
| ENSMUSG00000041189 | Chrnb1 | -1,09 | 0,001245286 | 54,82021 | 51,21325 | 55,36068 | 97,14598 | 105,2748 | 139,6907 |
| ENSMUSG00000026080 | Chst10 | -3,27 | 1,1348E-09 | 137,0505 | 41,55038 | 48,44059 | 816,8192 | 878,9813 | 483,1339 |
| ENSMUSG00000030930 | Chst15 | -1,16 | 4,31275E-06 | 2054,704 | 2637,966 | 2069,97 | 6180,665 | 4700,584 | 4275,246 |
| ENSMUSG00000057337 | Chst3 | -1,95 | 0,00792292 | 1725,782 | 506,3348 | 1382,287 | 7810,338 | 4675,217 | 1494,779 |
| ENSMUSG00000058152 | Chsy3 | -3,45 | 0,003488867 | 12,65082 | 1,932576 | 6,055074 | 21,80828 | 20,29394 | 181,5089 |
| ENSMUSG00000078695 | Cisd3/Cisd3 | 1,24 | 6,3826E-05 | 410,0973 | 380,7174 | 360,7094 | 165,5447 | 114,1534 | 206,4219 |
| ENSMUSG00000070803 | Cited4 | -2,08 | 1,43439E-06 | 153,9183 | 107,2579 | 167,8121 | 442,1133 | 433,783 | 943,1343 |
| ENSMUSG00000001270 | Ckb | 2,84 | 2,92573E-05 | 5820,43 | 10452,34 | 7649,289 | 630,4576 | 398,2686 | 2322,246 |
| ENSMUSG00000030399 | Ckm | 2,61 | 0,011638074 | 81,17608 | 79,2356 | 11,24514 | 6,938998 | 3,805114 | 16,90524 |
| ENSMUSG00000056025 | Clca3a1 | -2,64 | 0,00060421 | 270,9383 | 160,4038 | 133,2116 | 366,7756 | 603,7448 | 2558,029 |
| ENSMUSG00000040663 | Clcf1 | -1,67 | 6,41955E-10 | 294,1315 | 227,0776 | 245,663 | 1019,041 | 660,8215 | 759,846 |
| ENSMUSG00000022843 | Clcn2 | -1,98 | 1,92001E-11 | 207,6842 | 143,9769 | 135,8067 | 662,1787 | 509,8853 | 746,4997 |
| ENSMUSG00000004319 | Clcn3 | -1,65 | 5,5702E-21 | 2634,533 | 2042,732 | 2271,518 | 6873,573 | 7502,416 | 7424,958 |
| ENSMUSG00000022512 | Cldn1 | -3,44 | 9,18483E-05 | 314,162 | 16,42689 | 128,8866 | 1381,852 | 2469,519 | 1136,21 |
| ENSMUSG00000022132 | Cldn10 | -1,50 | 0,019241187 | 31,62704 | 17,39318 | 42,38552 | 43,61656 | 106,5432 | 108,5494 |
| ENSMUSG00000008843 | Cldn13 | -3,35 | 1,91735E-07 | 1515,99 | 223,2125 | 534,5765 | 8321,841 | 7896,88 | 6896,447 |
| ENSMUSG00000053063 | Clec12a | 1,23 | 4,52303E-05 | 20952,92 | 33740,84 | 26752,18 | 11890,47 | 13741,54 | 9130,608 |
| ENSMUSG00000030158 | Clec12b | 2,26 | 0,013826647 | 21,08469 | 11,59545 | 17,30021 | 2,973856 | 5,073485 | 2,669248 |
| ENSMUSG00000030159 | Clec1b | -2,80 | 0,00190582 | 410,0973 | 211,617 | 585,6122 | 622,5273 | 1165,633 | 6634,861 |
| ENSMUSG00000000248 | Clec2g | 1,04 | 0,022781606 | 56,92868 | 102,4265 | 69,20085 | 40,6427 | 32,97765 | 37,36947 |
| ENSMUSG00000030365 | Clec2i | 1,76 | 0,017959779 | 1001,523 | 4495,171 | 1786,247 | 833,6711 | 298,0673 | 1023,212 |
| ENSMUSG00000067767 | Clec4b2 | -2,56 | 0,001943702 | 68,52526 | 14,49432 | 33,73541 | 235,9259 | 384,3165 | 67,62095 |
| ENSMUSG00000030144 | Clec4d | -1,49 | 0,004719997 | 2578,658 | 1773,138 | 1434,188 | 5518,486 | 8224,12 | 2526,888 |
| ENSMUSG00000074491 | Clec4g | -2,81 | 0,005469822 | 5,271174 | 15,4606 | 6,920085 | 34,69499 | 17,7572 | 141,4701 |
| ENSMUSG00000079293 | Clec7a | 2,27 | 6,71739E-13 | 11729,42 | 23016,01 | 19374,51 | 4150,512 | 3177,27 | 3860,623 |
| ENSMUSG00000002190 | Clgn | -3,55 | 5,07488E-11 | 10,54235 | 8,69659 | 6,920085 | 69,38998 | 86,24925 | 147,6984 |
| ENSMUSG00000037242 | Clic4 | -1,30 | 0,0005052 | 5887,901 | 3735,669 | 4694,412 | 8475,491 | 10193,9 | 16495,06 |
| ENSMUSG00000021097 | Clmn | 4,74 | 1,78162E-22 | 274,101 | 218,381 | 219,7127 | 4,956427 | 17,7572 | 5,338496 |
| ENSMUSG00000032024 | Clmp | -1,14 | 0,013037545 | 45,33209 | 49,28068 | 45,84556 | 64,43356 | 106,5432 | 138,8009 |
| ENSMUSG00000022037 | Clu | -2,07 | 0,018441524 | 1212,37 | 882,2208 | 1680,716 | 1312,462 | 2307,167 | 12214,48 |
| ENSMUSG00000022225 | Cma1 | -3,84 | 0,001631985 | 6,325408 | 2,898863 | 4,325053 | 13,878 | 13,95208 | 165,4934 |
| ENSMUSG00000016756 | Cmah | -2,44 | 6,07599E-07 | 1216,587 | 509,2337 | 742,1791 | 3753,007 | 2730,803 | 6871,534 |
| ENSMUSG00000030282 | Cmas | -1,15 | 2,19025E-07 | 3394,636 | 3226,435 | 3653,805 | 7171,95 | 6421,764 | 9225,811 |
| ENSMUSG00000022235 | Cmbl | -2,49 | 0,000431309 | 31,62704 | 14,49432 | 46,71057 | 123,9107 | 83,71251 | 314,0815 |
| ENSMUSG00000042190 | Cmklr1 | -1,46 | 0,000635911 | 143,3759 | 70,53901 | 148,7818 | 261,6994 | 310,751 | 422,6309 |
| ENSMUSG00000032436 | Cmtm7 | 1,06 | 5,64972E-08 | 14595,88 | 12270,89 | 14699,12 | 6968,737 | 7199,276 | 5723,758 |
| ENSMUSG00000041012 | Cmtm8 | -1,01 | 0,036472412 | 18,97623 | 21,25833 | 26,81533 | 41,63399 | 41,85625 | 51,60546 |
| ENSMUSG00000047419 | Cmya5 | -2,06 | 6,65956E-08 | 31,62704 | 32,85379 | 25,95032 | 111,024 | 100,2013 | 164,6036 |
| ENSMUSG00000038085 | Cnbd2 | 1,69 | 3,72133E-09 | 347,8975 | 497,6382 | 393,5798 | 126,8845 | 154,7413 | 104,9904 |
| ENSMUSG00000030897 | Cnga4 | -2,41 | 0,003218281 | 5,271174 | 6,764015 | 5,190064 | 45,59913 | 32,97765 | 13,34624 |
| ENSMUSG00000015202 | Cnksr3 | 1,13 | 1,59966E-08 | 1056,343 | 1001,074 | 1031,093 | 451,0349 | 408,4156 | 547,1959 |
| ENSMUSG00000053931 | Cnn3 | 1,20 | 3,91507E-06 | 4075,672 | 3515,355 | 4120,045 | 2067,821 | 1737,669 | 1287,467 |
| ENSMUSG00000025189 | Cnnm1 | -3,03 | 0,000145325 | 4,216939 | 5,797727 | 1,730021 | 39,65142 | 19,02557 | 35,58997 |
| ENSMUSG00000064105 | Cnnm2 | -1,69 | 0,033991484 | 856,0386 | 195,1901 | 291,5086 | 1126,1 | 2534,206 | 659,3043 |
| ENSMUSG00000023973 | Cnpy3 | 1,02 | 1,59914E-11 | 7219,4 | 7037,474 | 8093,039 | 3584,488 | 3892,632 | 3547,431 |
| ENSMUSG00000044629 | Cnrip1 | -1,14 | 0,001613512 | 54,82021 | 45,41553 | 75,25592 | 137,7887 | 110,3483 | 137,9112 |
| ENSMUSG00000030075 | Cntn3 | -8,38 | 0,0128118 | 0 | 0 | 0 | 12,88671 | 8,878599 | 13,34624 |
| ENSMUSG00000017167 | Cntnap1 | 1,69 | 0,000116503 | 202,4131 | 337,2344 | 156,5669 | 61,4597 | 62,1502 | 91,64418 |
| ENSMUSG00000034903 | Cobll1 | -1,59 | 8,84712E-08 | 250,9079 | 146,8757 | 224,0377 | 722,6471 | 564,4252 | 589,9038 |
| ENSMUSG00000020953 | Coch | 8,28 | 1,9337E-08 | 1006,794 | 1725,79 | 1883,993 | 0 | 1,268371 | 13,34624 |
| ENSMUSG00000022371 | Col14a1 | -4,55 | 4,61494E-09 | 6,325408 | 5,797727 | 10,38013 | 322,1678 | 46,92974 | 159,2651 |
| ENSMUSG00000028339 | Col15a1 | -2,02 | 0,001277688 | 8,433878 | 16,42689 | 23,35529 | 37,66885 | 77,37065 | 81,85694 |
| ENSMUSG00000040690 | Col16a1 | -1,57 | 0,032153057 | 37,95245 | 49,28068 | 81,31099 | 87,23312 | 96,39622 | 316,7508 |
| ENSMUSG00000063564 | Col23a1 | -1,62 | 0,000199716 | 30,57281 | 22,22462 | 19,03023 | 56,50327 | 81,17577 | 81,85694 |
| ENSMUSG00000026837 | Col5a1 | -3,12 | 1,22821E-18 | 278,318 | 135,2803 | 219,7127 | 1683,203 | 2341,413 | 1463,638 |
| ENSMUSG00000043719 | Col6a6 | 5,57 | 8,79164E-16 | 129,6709 | 116,9208 | 158,2969 | 1,982571 | 7,610228 | 0 |
| ENSMUSG00000068196 | Col8a1 | -2,04 | 0,013885943 | 32,68128 | 29,95492 | 89,9611 | 61,4597 | 200,4027 | 367,4665 |
| ENSMUSG00000079559 | Colca2 | -1,14 | 0,023762617 | 23,19316 | 16,42689 | 15,57019 | 40,6427 | 44,393 | 36,47972 |
| ENSMUSG00000057606 | Colq | -2,43 | 0,000954507 | 15,81352 | 6,764015 | 30,27537 | 85,25055 | 50,73485 | 149,4779 |
| ENSMUSG00000031458 | Coprs | 1,43 | 2,45178E-07 | 226,6605 | 220,3136 | 227,4978 | 76,32898 | 68,49205 | 104,1007 |
| ENSMUSG00000028337 | Coro2a | 1,35 | 2,57991E-08 | 5897,389 | 7266,484 | 6888,944 | 2277,974 | 2337,608 | 3270,719 |
| ENSMUSG00000041729 | Coro2b | -2,03 | 1,70833E-05 | 39,00669 | 19,32576 | 27,68034 | 76,32898 | 144,5943 | 130,7932 |
| ENSMUSG00000030785 | Cox6a2 | -2,44 | 3,417E-06 | 99,09807 | 72,47159 | 90,82611 | 282,5164 | 299,3356 | 839,9234 |
| ENSMUSG00000074218 | Cox7a1 | 1,10 | 0,006522756 | 51,6575 | 69,57272 | 70,06586 | 30,72985 | 29,17254 | 29,36173 |
| ENSMUSG00000071553 | Cpa2 | 1,90 | 0,038349184 | 11,59658 | 14,49432 | 23,35529 | 5,947713 | 3,805114 | 3,558997 |
| ENSMUSG00000042501 | Cpa6 | -1,68 | 0,017187563 | 10,54235 | 10,62917 | 7,785095 | 36,67756 | 39,31951 | 16,90524 |
| ENSMUSG00000039652 | Cpeb3 | -2,03 | 6,44563E-06 | 216,1181 | 91,79734 | 127,1566 | 757,3421 | 624,0387 | 391,4897 |
| ENSMUSG00000020300 | Cpeb4 | -1,58 | 2,51757E-07 | 4301,278 | 2250,484 | 3091,548 | 10546,29 | 9460,782 | 8819,196 |
| ENSMUSG00000025867 | Cplx2 | -1,56 | 0,005480293 | 698,9576 | 265,7291 | 623,6726 | 2131,264 | 1756,694 | 805,2232 |
| ENSMUSG00000034361 | Cpne2 | -1,54 | 6,52795E-08 | 2667,214 | 2335,518 | 2304,388 | 6982,615 | 9137,347 | 5114,279 |
| ENSMUSG00000032564 | Cpne4 | -5,64 | 0,018441524 | 0 | 0,966288 | 0 | 2,973856 | 41,85625 | 3,558997 |
| ENSMUSG00000024008 | Cpne5 | -2,02 | 0,002069921 | 32,68128 | 27,05606 | 15,57019 | 176,4488 | 59,61345 | 67,62095 |
| ENSMUSG00000022742 | Cpox | -2,18 | 1,75944E-06 | 5219,516 | 1774,104 | 2548,321 | 15735,67 | 16105,78 | 11431,5 |
| ENSMUSG00000007783 | Cpt1c | -1,33 | 0,033991484 | 14,75929 | 45,41553 | 18,16522 | 64,43356 | 67,22368 | 64,9517 |
| ENSMUSG00000026616 | Cr2 | -2,86 | 1,29596E-09 | 188,708 | 68,60643 | 69,20085 | 931,8083 | 687,4573 | 742,051 |
| ENSMUSG00000048200 | Cracr2b | 1,34 | 4,59302E-09 | 452,2667 | 472,5147 | 595,9923 | 191,3181 | 219,4282 | 191,2961 |
| ENSMUSG00000038648 | Creb3l2 | 1,08 | 0,000128495 | 4642,85 | 3463,175 | 3172,859 | 1702,037 | 2146,084 | 1473,425 |
| ENSMUSG00000035041 | Creb3l3 | -2,61 | 0,000169273 | 15,81352 | 7,730302 | 3,460042 | 60,46841 | 65,95531 | 36,47972 |
| ENSMUSG00000006360 | Crip1 | 1,31 | 8,77339E-12 | 12988,17 | 11888,24 | 15181,8 | 5904,096 | 5374,089 | 4839,347 |
| ENSMUSG00000006356 | Crip2 | -3,03 | 3,37155E-10 | 148,6471 | 54,11212 | 135,8067 | 738,5077 | 684,9205 | 1338,183 |
| ENSMUSG00000023968 | Crip3 | -3,73 | 0,000184195 | 2,108469 | 0,966288 | 1,730021 | 22,79957 | 15,22046 | 24,91298 |
| ENSMUSG00000025776 | Crispld1 | -2,06 | 0,028005973 | 6,325408 | 6,764015 | 12,97516 | 19,82571 | 17,7572 | 71,17995 |
| ENSMUSG00000031825 | Crispld2 | -1,53 | 0,006175467 | 3397,799 | 2704,64 | 2486,905 | 8576,602 | 12980,51 | 3199,539 |
| ENSMUSG00000042401 | Crtac1 | -2,88 | 9,31133E-11 | 35,84398 | 23,19091 | 26,81533 | 270,6209 | 120,4953 | 239,3426 |
| ENSMUSG00000032060 | Cryab | 1,73 | 1,38397E-05 | 140,2132 | 230,9428 | 124,5615 | 42,62527 | 57,07671 | 49,82596 |
| ENSMUSG00000066975 | Cryba4 | 1,10 | 0,004295599 | 78,01337 | 82,13446 | 77,85095 | 28,74728 | 35,5144 | 46,26697 |
| ENSMUSG00000022723 | Crybg3 | 1,07 | 0,002802087 | 2070,517 | 1977,025 | 2242,972 | 1437,364 | 830,7832 | 722,4765 |
| ENSMUSG00000091562 | Crybg3 | 1,31 | 0,000134444 | 771,6998 | 714,0867 | 778,5095 | 414,3573 | 298,0673 | 200,1936 |
| ENSMUSG00000024621 | Csf1r | 2,08 | 1,66032E-17 | 31367,7 | 39640,99 | 38052,68 | 10061,55 | 6575,237 | 9095,018 |
| ENSMUSG00000036356 | Csgalnact1 | -2,15 | 0,007836908 | 148,6471 | 123,6848 | 167,8121 | 196,2745 | 300,604 | 1458,299 |
| ENSMUSG00000030470 | Csrp3 | -2,33 | 3,82624E-06 | 18,97623 | 20,29204 | 6,920085 | 78,31155 | 73,56554 | 78,29794 |
| ENSMUSG00000027447 | Cst3 | 1,01 | 0,000202424 | 21991,34 | 23410,25 | 24748,82 | 9398,377 | 10386,69 | 15099,94 |
| ENSMUSG00000068129 | Cst7 | 1,14 | 0,014653946 | 3358,792 | 2784,841 | 3830,267 | 1469,085 | 2250,091 | 801,6642 |
| ENSMUSG00000074874 | Ctla2b | -3,33 | 6,80332E-05 | 173,9487 | 85,99961 | 92,55613 | 312,2549 | 478,176 | 2741,318 |
| ENSMUSG00000022240 | Ctnnd2 | -1,78 | 0,002069921 | 13,70505 | 21,25833 | 17,30021 | 32,71242 | 77,37065 | 70,2902 |
| ENSMUSG00000030560 | Ctsc | 1,06 | 1,52111E-05 | 14296,48 | 11436,98 | 10994,28 | 4839,456 | 6879,646 | 5934,628 |
| ENSMUSG00000004552 | Ctse | -2,82 | 0,000213543 | 12500,06 | 1679,408 | 2406,459 | 46707,39 | 33545,89 | 36547,35 |
| ENSMUSG00000083282 | Ctsf | -1,85 | 2,76944E-07 | 204,5215 | 204,853 | 208,4676 | 711,743 | 469,2974 | 1048,125 |
| ENSMUSG00000040314 | Ctsg | 2,32 | 4,06443E-05 | 50690,77 | 48486,39 | 59682,27 | 11174,76 | 17202,92 | 3525,187 |
| ENSMUSG00000038642 | Ctss | 1,16 | 9,22056E-07 | 23048,73 | 22082,58 | 24182,24 | 9002,855 | 9044,756 | 13005,47 |
| ENSMUSG00000031078 | Cttn | -2,70 | 0,000187181 | 552,419 | 258,9651 | 491,326 | 1078,519 | 1392,672 | 5980,005 |
| ENSMUSG00000000416 | Cttnbp2 | -2,42 | 0,009996259 | 7,379643 | 0,966288 | 4,325053 | 21,80828 | 30,44091 | 15,12574 |
| ENSMUSG00000042589 | Cux2 | 5,59 | 2,36748E-24 | 396,3923 | 195,1901 | 518,1413 | 7,930284 | 10,14697 | 5,338496 |
| ENSMUSG00000022865 | Cxadr | -2,02 | 0,000835558 | 87,50148 | 18,35947 | 44,11554 | 169,5098 | 209,2813 | 227,7758 |
| ENSMUSG00000034855 | Cxcl10 | 1,94 | 0,000312876 | 274,101 | 621,3231 | 243,933 | 81,28541 | 71,02879 | 144,1394 |
| ENSMUSG00000061353 | Cxcl12 | -1,80 | 0,001922882 | 8458,125 | 4473,913 | 9661,303 | 11427,54 | 24399,66 | 42860,12 |
| ENSMUSG00000023078 | Cxcl13 | -3,08 | 0,043265089 | 4,216939 | 0,966288 | 5,190064 | 2,973856 | 10,14697 | 74,73895 |
| ENSMUSG00000058427 | Cxcl2 | -3,35 | 0,017104746 | 27,4101 | 0,966288 | 6,920085 | 13,878 | 294,2622 | 51,60546 |
| ENSMUSG00000029371 | Cxcl5 | -2,23 | 0,021891693 | 59,03715 | 29,95492 | 43,25053 | 100,1198 | 40,58788 | 476,9057 |
| ENSMUSG00000048480 | Cxcr1 | 3,27 | 3,25066E-08 | 614,6189 | 1429,14 | 706,7136 | 48,57299 | 62,1502 | 172,6114 |
| ENSMUSG00000026180 | Cxcr2 | -1,59 | 1,1626E-07 | 7536,724 | 8298,48 | 6823,203 | 26662,61 | 26259,09 | 15167,56 |
| ENSMUSG00000047880 | Cxcr5 | -2,36 | 6,95526E-06 | 312,0535 | 86,9659 | 218,8477 | 1386,808 | 739,4605 | 1040,117 |
| ENSMUSG00000046668 | Cxxc5 | -1,45 | 1,16743E-07 | 636,7578 | 564,3121 | 730,9339 | 1715,915 | 1360,962 | 2203,909 |
| ENSMUSG00000019590 | Cyb561 | -1,68 | 0,02127114 | 43,22362 | 9,662878 | 19,03023 | 48,57299 | 88,78599 | 91,64418 |
| ENSMUSG00000020810 | Cygb | -1,39 | 0,036551041 | 17,92199 | 28,02235 | 39,79049 | 48,57299 | 54,53997 | 121,0059 |
| ENSMUSG00000046213 | Cym | -2,38 | 0,021040869 | 2,108469 | 2,898863 | 3,460042 | 10,90414 | 10,14697 | 23,13348 |
| ENSMUSG00000024087 | Cyp1b1 | -2,09 | 0,008493917 | 260,396 | 65,70757 | 181,6522 | 190,3268 | 1045,138 | 927,1188 |
| ENSMUSG00000026170 | Cyp27a1 | 1,52 | 0,005765672 | 1682,559 | 2979,065 | 1967,899 | 988,3116 | 315,8245 | 1007,196 |
| ENSMUSG00000028713 | Cyp4b1 | -2,47 | 0,0004469 | 66,41679 | 12,56174 | 38,92548 | 125,8933 | 183,9138 | 342,5535 |
| ENSMUSG00000083457 | Cyp4b1-ps2 | -3,70 | 6,54815E-18 | 27,4101 | 10,62917 | 22,49028 | 192,3094 | 272,6998 | 320,3098 |
| ENSMUSG00000048440 | Cyp4f16 | 2,30 | 5,9821E-14 | 2595,526 | 3903,803 | 3492,913 | 612,6144 | 520,0322 | 887,9699 |
| ENSMUSG00000091586 | Cyp4f17 | 1,32 | 0,006054031 | 120,1828 | 237,7068 | 118,5065 | 76,32898 | 46,92974 | 66,7312 |
| ENSMUSG00000062464 | Cyp4f37 | 2,59 | 0,04214848 | 5,271174 | 61,84242 | 38,06047 | 0,991285 | 7,610228 | 8,897494 |
| ENSMUSG00000061126 | Cyp4f39 | -5,18 | 0,017769045 | 1,054235 | 0 | 0 | 3,965142 | 8,878599 | 22,24373 |
| ENSMUSG00000079057 | Cyp4v3 | 1,46 | 6,53777E-07 | 1623,522 | 1722,891 | 1798,357 | 510,512 | 507,3485 | 848,8209 |
| ENSMUSG00000052821 | Cysltr1 | 3,07 | 1,96209E-56 | 1199,719 | 1294,826 | 1416,887 | 142,7451 | 145,8627 | 176,1704 |
| ENSMUSG00000033470 | Cysltr2 | -3,34 | 0,001189437 | 90,66419 | 24,1572 | 25,95032 | 104,085 | 150,9362 | 1162,902 |
| ENSMUSG00000018001 | Cyth3 | -2,13 | 4,4435E-14 | 996,2518 | 599,0984 | 780,2395 | 3284,129 | 4120,938 | 2957,527 |
| ENSMUSG00000085772 | D630024D03Rik | -2,99 | 0,004994371 | 1,054235 | 0,966288 | 3,460042 | 13,878 | 13,95208 | 16,90524 |
| ENSMUSG00000030994 | D7Ertd443e | -2,77 | 0,025182192 | 2,108469 | 4,831439 | 4,325053 | 6,938998 | 11,41534 | 58,72346 |
| ENSMUSG00000043126 | D830039M14Rik | 2,26 | 0,034941818 | 9,488113 | 36,71894 | 16,4352 | 1,982571 | 3,805114 | 7,117995 |
| ENSMUSG00000097075 | D830044I16Rik | 1,44 | 0,001491146 | 88,55572 | 164,2689 | 99,47622 | 55,51199 | 38,05114 | 36,47972 |
| ENSMUSG00000034574 | Daam1 | -1,28 | 0,001890003 | 2316,154 | 1729,655 | 2091,596 | 3252,408 | 4093,034 | 7525,5 |
| ENSMUSG00000028519 | Dab1 | 6,24 | 0,000581952 | 35,84398 | 26,08977 | 17,30021 | 0 | 1,268371 | 0 |
| ENSMUSG00000022150 | Dab2 | -1,71 | 0,005467519 | 137,0505 | 111,1231 | 185,9773 | 185,3704 | 806,6842 | 428,8592 |
| ENSMUSG00000026883 | Dab2ip | -2,12 | 8,46759E-22 | 511,3038 | 373,9534 | 442,0204 | 2082,691 | 1641,273 | 2051,762 |
| ENSMUSG00000044548 | Dact1 | -1,74 | 0,012752104 | 12,65082 | 13,52803 | 8,650106 | 25,77342 | 27,90417 | 61,39271 |
| ENSMUSG00000021559 | Dapk1 | -1,34 | 0,014958483 | 519,7377 | 537,256 | 515,5463 | 706,7865 | 915,7641 | 2347,159 |
| ENSMUSG00000032380 | Dapk2 | -2,46 | 7,00528E-14 | 417,477 | 274,4257 | 257,7732 | 1906,242 | 2072,519 | 1239,421 |
| ENSMUSG00000026385 | Dbi | 1,20 | 3,81768E-09 | 4225,373 | 4211,082 | 5285,215 | 1938,954 | 2229,797 | 1799,963 |
| ENSMUSG00000028436 | Dcaf12 | -1,17 | 3,03461E-06 | 8620,477 | 6173,613 | 7204,673 | 19489,66 | 15991,63 | 13899,66 |
| ENSMUSG00000045284 | Dcaf12l1 | -2,36 | 0,012968575 | 2,108469 | 6,764015 | 3,460042 | 10,90414 | 21,56231 | 31,14123 |
| ENSMUSG00000026571 | Dcaf6 | -1,57 | 1,5843E-16 | 1384,21 | 1170,175 | 1236,965 | 4147,538 | 3904,047 | 3229,79 |
| ENSMUSG00000036862 | Dchs1 | -1,24 | 0,04466889 | 20,03046 | 19,32576 | 41,52051 | 44,60785 | 59,61345 | 88,08519 |
| ENSMUSG00000027797 | Dclk1 | -2,30 | 0,004249281 | 11,59658 | 10,62917 | 17,30021 | 19,82571 | 63,41857 | 112,1084 |
| ENSMUSG00000028078 | Dclk2 | -2,20 | 9,87014E-16 | 249,8536 | 150,7409 | 164,352 | 946,6776 | 842,1986 | 811,4514 |
| ENSMUSG00000032500 | Dclk3 | -8,13 | 0,042380192 | 0 | 0 | 0 | 0 | 6,341857 | 23,13348 |
| ENSMUSG00000022303 | Dcstamp | -3,24 | 0,004069506 | 5,271174 | 1,932576 | 8,650106 | 21,80828 | 13,95208 | 113,8879 |
| ENSMUSG00000078515 | Ddi2 | 1,00 | 0,001022544 | 13857,92 | 8285,918 | 10753,81 | 5798,029 | 6142,722 | 4472,77 |
| ENSMUSG00000059213 | Ddn | -2,76 | 0,002484986 | 9,488113 | 6,764015 | 5,190064 | 28,74728 | 17,7572 | 96,98268 |
| ENSMUSG00000003534 | Ddr1 | -1,95 | 6,66957E-06 | 202,4131 | 110,1568 | 136,6717 | 387,5926 | 499,7383 | 850,6004 |
| ENSMUSG00000026674 | Ddr2 | -2,06 | 3,26813E-06 | 83,28454 | 40,58409 | 63,14577 | 162,5708 | 299,3356 | 317,6405 |
| ENSMUSG00000021758 | Ddx4 | 1,57 | 1,78993E-07 | 144,4302 | 206,7856 | 170,4071 | 60,46841 | 63,41857 | 52,49521 |
| ENSMUSG00000035392 | Dennd1a | 1,20 | 7,10637E-13 | 3987,116 | 4424,632 | 3624,394 | 1860,643 | 1697,081 | 1691,414 |
| ENSMUSG00000038456 | Dennd2a | 1,35 | 0,000107697 | 715,8254 | 1291,927 | 1194,58 | 494,6514 | 433,783 | 329,2073 |
| ENSMUSG00000007379 | Dennd2c | -1,59 | 1,91825E-11 | 513,4123 | 500,5371 | 427,3152 | 1388,791 | 1199,879 | 1760,814 |
| ENSMUSG00000036661 | Dennd3 | -1,07 | 0,000129114 | 1631,955 | 2003,115 | 1211,015 | 3744,085 | 3414,456 | 3018,92 |
| ENSMUSG00000053641 | Dennd4a | -1,40 | 9,56569E-07 | 6737,614 | 5553,256 | 5086,262 | 16397,84 | 18415,48 | 11058,69 |
| ENSMUSG00000030313 | Dennd5b | -1,41 | 0,002041903 | 431,182 | 185,5273 | 320,0539 | 1132,048 | 753,4126 | 600,5808 |
| ENSMUSG00000027173 | Depdc7 | 3,31 | 6,38958E-32 | 605,1307 | 591,3681 | 820,03 | 83,26798 | 55,80834 | 63,17221 |
| ENSMUSG00000022419 | Deptor | 2,76 | 5,80682E-59 | 4459,413 | 4925,169 | 5635,544 | 744,4554 | 698,8726 | 769,6332 |
| ENSMUSG00000022555 | Dgat1 | -1,37 | 0,003961233 | 5696,03 | 3958,881 | 3079,438 | 10621,62 | 16145,1 | 6172,191 |
| ENSMUSG00000030747 | Dgat2 | -1,94 | 0,000642311 | 2163,29 | 1433,005 | 1362,392 | 7287,931 | 9528,005 | 2269,751 |
| ENSMUSG00000038665 | Dgki | 5,76 | 4,48395E-60 | 1268,244 | 1102,534 | 1094,238 | 17,84314 | 11,41534 | 32,92073 |
| ENSMUSG00000011382 | Dhdh | 1,39 | 3,38756E-15 | 1137,519 | 1341,207 | 1204,96 | 509,5207 | 455,3453 | 437,7567 |
| ENSMUSG00000034449 | Dhrs11 | -2,76 | 1,13916E-13 | 1973,527 | 913,142 | 1063,963 | 10043,7 | 9853,977 | 6926,699 |
| ENSMUSG00000021094 | Dhrs7 | -1,25 | 0,000855265 | 1853,345 | 2563,562 | 1762,027 | 5696,918 | 5967,687 | 3045,612 |
| ENSMUSG00000027068 | Dhrs9 | -2,10 | 1,94276E-05 | 1917,653 | 1129,59 | 1185,065 | 5801,994 | 9374,533 | 2964,645 |
| ENSMUSG00000047842 | Diras2 | -1,73 | 0,011383754 | 257,2333 | 280,2235 | 317,4589 | 475,817 | 433,783 | 1931,646 |
| ENSMUSG00000022848 | Dirc2 | 1,04 | 2,9708E-07 | 4541,643 | 3478,636 | 3682,35 | 1813,061 | 1811,234 | 2082,014 |
| ENSMUSG00000041544 | Disp3 | -6,97 | 1,83416E-06 | 1,054235 | 0 | 0,865011 | 29,73856 | 31,70928 | 177,0601 |
| ENSMUSG00000030792 | Dkkl1 | -1,99 | 0,007594832 | 47,44056 | 18,35947 | 72,66089 | 113,0065 | 109,0799 | 326,538 |
| ENSMUSG00000031523 | Dlc1 | -1,31 | 0,033680821 | 148,6471 | 78,26931 | 143,5918 | 154,6405 | 249,8692 | 511,6059 |
| ENSMUSG00000048281 | Dleu7 | 3,42 | 1,39881E-14 | 103,315 | 107,2579 | 159,1619 | 9,912855 | 10,14697 | 14,23599 |
| ENSMUSG00000052572 | Dlg2 | -2,95 | 4,17541E-05 | 74,85067 | 56,04469 | 64,87579 | 180,414 | 249,8692 | 1084,604 |
| ENSMUSG00000020886 | Dlg4 | 1,00 | 0,040281174 | 818,0862 | 780,7605 | 764,6694 | 247,8214 | 289,1887 | 641,5093 |
| ENSMUSG00000021782 | Dlg5 | -1,51 | 0,000364936 | 100,1523 | 76,33674 | 55,36068 | 144,7277 | 270,1631 | 242,9016 |
| ENSMUSG00000040856 | Dlk1 | 7,18 | 2,41767E-16 | 112,8031 | 818,4458 | 850,3054 | 0,991285 | 5,073485 | 6,228246 |
| ENSMUSG00000045103 | Dmd | -1,75 | 0,004110724 | 16,86776 | 5,797727 | 18,16522 | 45,59913 | 40,58788 | 50,71571 |
| ENSMUSG00000030409 | Dmpk | -3,20 | 8,4547E-17 | 74,85067 | 40,58409 | 51,03562 | 587,8323 | 332,3133 | 606,8091 |
| ENSMUSG00000028610 | Dmrtb1 | 5,72 | 2,95245E-12 | 132,8336 | 106,2917 | 382,3347 | 5,947713 | 0 | 5,338496 |
| ENSMUSG00000022099 | Dmtn | -3,40 | 1,90913E-07 | 2048,378 | 300,5155 | 702,3886 | 12329,61 | 11155,33 | 8630,569 |
| ENSMUSG00000030410 | Dmwd | -2,81 | 5,55879E-18 | 301,5111 | 154,606 | 170,4071 | 1613,813 | 1457,359 | 1311,491 |
| ENSMUSG00000019027 | Dnah1 | -1,58 | 0,002924396 | 10,54235 | 14,49432 | 13,84017 | 43,61656 | 30,44091 | 41,81822 |
| ENSMUSG00000005237 | Dnah2 | 1,85 | 1,2725E-10 | 318,3789 | 503,4359 | 402,2299 | 100,1198 | 112,885 | 127,2342 |
| ENSMUSG00000052861 | Dnah6 | -2,25 | 4,96312E-05 | 65,36255 | 19,32576 | 24,2203 | 202,2222 | 143,326 | 170,8319 |
| ENSMUSG00000032285 | Dnaja4 | -2,81 | 1,41561E-07 | 287,8061 | 132,3814 | 104,6663 | 1642,56 | 1404,087 | 638,84 |
| ENSMUSG00000026203 | Dnajb2 | -2,53 | 2,57609E-10 | 610,4019 | 220,3136 | 380,6047 | 2470,283 | 2341,413 | 2164,76 |
| ENSMUSG00000081984 | Dnajb3 | -3,12 | 6,92775E-09 | 32,68128 | 21,25833 | 9,515116 | 188,3442 | 240,9906 | 119,2264 |
| ENSMUSG00000028035 | Dnajb4 | -2,16 | 7,54635E-10 | 974,1129 | 470,5822 | 617,6176 | 2835,076 | 2630,602 | 3778,766 |
| ENSMUSG00000027006 | Dnajc10 | 2,59 | 4,5684E-75 | 24407,64 | 25746,74 | 26512,57 | 3890,795 | 4491,303 | 4396,252 |
| ENSMUSG00000028528 | Dnajc6 | -1,66 | 8,45324E-06 | 205,5758 | 165,2352 | 281,9935 | 512,4946 | 616,4285 | 940,4651 |
| ENSMUSG00000026825 | Dnm1 | 2,62 | 0,00143476 | 32,68128 | 62,80871 | 52,76565 | 6,938998 | 1,268371 | 15,12574 |
| ENSMUSG00000082079 | Dnmt3c | -2,88 | 4,44047E-05 | 5,271174 | 2,898863 | 4,325053 | 22,79957 | 35,5144 | 33,81048 |
| ENSMUSG00000038608 | Dock10 | 1,43 | 3,36344E-19 | 16862,48 | 19536,41 | 19448,9 | 7016,319 | 7380,653 | 6390,18 |
| ENSMUSG00000032198 | Dock6 | 1,06 | 0,001749859 | 431,182 | 648,3791 | 493,921 | 247,8214 | 187,719 | 315,861 |
| ENSMUSG00000025558 | Dock9 | -1,21 | 0,035897276 | 554,5275 | 331,4367 | 429,0453 | 1765,479 | 630,3806 | 638,84 |
| ENSMUSG00000040631 | Dok4 | -1,25 | 2,52618E-05 | 104,3692 | 108,2242 | 81,31099 | 283,5076 | 205,4762 | 209,9809 |
| ENSMUSG00000044716 | Dok7 | -3,99 | 0,011564296 | 1,054235 | 0,966288 | 0 | 12,88671 | 8,878599 | 8,897494 |
| ENSMUSG00000019278 | Dpep1 | -1,90 | 0,003996027 | 161,2979 | 59,90984 | 148,7818 | 222,0479 | 347,5337 | 809,6719 |
| ENSMUSG00000053687 | Dpep2 | 2,80 | 1,51198E-10 | 741,127 | 1945,137 | 1796,627 | 188,3442 | 276,505 | 177,9499 |
| ENSMUSG00000031898 | Dpep3 | 2,93 | 0,000717469 | 12,65082 | 44,44924 | 44,98055 | 4,956427 | 5,073485 | 3,558997 |
| ENSMUSG00000021221 | Dpf3 | -2,70 | 4,53515E-07 | 92,77266 | 26,08977 | 38,92548 | 362,8105 | 414,7574 | 247,3503 |
| ENSMUSG00000035000 | Dpp4 | 1,00 | 0,00043566 | 3103,667 | 4370,52 | 3861,407 | 2333,486 | 1542,34 | 1783,947 |
| ENSMUSG00000060461 | Dppa5a | 7,84 | 0,032172864 | 2,108469 | 5,797727 | 18,16522 | 0 | 0 | 0 |
| ENSMUSG00000022048 | Dpysl2 | 1,06 | 0,000368151 | 1274,57 | 853,2321 | 1000,817 | 587,8323 | 391,9267 | 517,8341 |
| ENSMUSG00000024501 | Dpysl3 | -1,48 | 0,048059982 | 72,7422 | 80,20189 | 157,4319 | 112,0153 | 219,4282 | 532,9599 |
| ENSMUSG00000029265 | Dr1 | 1,11 | 0,000802745 | 9429,076 | 5196,696 | 9555,772 | 3964,151 | 3881,216 | 3365,922 |
| ENSMUSG00000029005 | Draxin | -3,51 | 7,95275E-05 | 33,73551 | 18,35947 | 35,46543 | 128,8671 | 81,17577 | 788,3179 |
| ENSMUSG00000024331 | Dsc2 | -5,16 | 2,18448E-06 | 3,162704 | 0,966288 | 0 | 69,38998 | 49,46648 | 21,35398 |
| ENSMUSG00000039497 | Dse | 1,14 | 0,006046172 | 1460,115 | 1859,138 | 1579,509 | 505,5556 | 578,3773 | 1129,982 |
| ENSMUSG00000026131 | Dst | -1,64 | 0,000279736 | 415,3685 | 297,6166 | 365,8995 | 775,1852 | 771,1698 | 1809,75 |
| ENSMUSG00000042046 | Dstyk | 1,18 | 1,49603E-06 | 4798,877 | 5310,718 | 5309,435 | 2452,44 | 2626,797 | 1710,098 |
| ENSMUSG00000029603 | Dtx1 | -2,54 | 6,2703E-10 | 91,71842 | 39,6178 | 53,63066 | 459,9565 | 317,0928 | 296,2865 |
| ENSMUSG00000039384 | Dusp10 | -1,59 | 0,004883869 | 267,7756 | 150,7409 | 178,1922 | 346,9499 | 407,1472 | 1040,117 |
| ENSMUSG00000018648 | Dusp14 | -2,62 | 0,014412197 | 12,65082 | 17,39318 | 24,2203 | 19,82571 | 43,12463 | 269,5941 |
| ENSMUSG00000027368 | Dusp2 | -1,18 | 0,011106333 | 776,971 | 415,5038 | 707,5787 | 2064,848 | 1326,716 | 911,9931 |
| ENSMUSG00000069255 | Dusp22 | 1,99 | 1,44662E-12 | 954,0824 | 1461,993 | 1348,552 | 386,6013 | 277,7733 | 283,83 |
| ENSMUSG00000053716 | Dusp7 | 1,25 | 2,44179E-07 | 2312,991 | 3164,593 | 2856,265 | 1273,802 | 957,6204 | 1272,342 |
| ENSMUSG00000037887 | Dusp8 | -3,02 | 1,00613E-05 | 215,0639 | 35,75265 | 59,68573 | 947,6689 | 994,4031 | 568,5498 |
| ENSMUSG00000031383 | Dusp9 | -2,38 | 0,00338006 | 14,75929 | 23,19091 | 24,2203 | 51,54684 | 45,66137 | 225,9963 |
| ENSMUSG00000016526 | Dyrk3 | -2,38 | 2,01842E-13 | 586,1545 | 303,4144 | 398,7699 | 2519,848 | 2398,49 | 1799,963 |
| ENSMUSG00000033788 | Dysf | -2,03 | 0,000695561 | 28,46434 | 30,92121 | 55,36068 | 109,0414 | 95,12785 | 266,0351 |
| ENSMUSG00000087406 | E130215H24Rik | -1,94 | 5,29674E-05 | 12,65082 | 12,56174 | 20,76025 | 51,54684 | 72,29717 | 54,27471 |
| ENSMUSG00000014859 | E2f4 | -1,01 | 4,18917E-06 | 5329,157 | 3964,679 | 4306,023 | 9538,149 | 9820,999 | 7993,508 |
| ENSMUSG00000022838 | Eaf2 | -1,00 | 0,035313241 | 70,63373 | 51,21325 | 61,41575 | 156,6231 | 136,9841 | 73,8492 |
| ENSMUSG00000057098 | Ebf1 | -1,70 | 0,008975363 | 946,7028 | 278,2909 | 665,1931 | 3146,34 | 2066,177 | 934,2368 |
| ENSMUSG00000010476 | Ebf3 | -2,36 | 0,003728982 | 25,30163 | 113,0557 | 60,55074 | 245,8388 | 123,032 | 649,517 |
| ENSMUSG00000057530 | Ece1 | 1,48 | 2,27828E-13 | 3529,578 | 3352,052 | 3244,655 | 1010,12 | 1221,442 | 1409,363 |
| ENSMUSG00000025465 | Echs1 | 1,33 | 4,41788E-14 | 4895,866 | 5318,448 | 6296,412 | 2116,394 | 2245,017 | 2187,894 |
| ENSMUSG00000059327 | Eda | -1,74 | 0,000321906 | 20,03046 | 15,4606 | 23,35529 | 47,5817 | 63,41857 | 85,41594 |
| ENSMUSG00000003227 | Edar | -2,41 | 0,019961732 | 4,216939 | 4,831439 | 3,460042 | 14,86928 | 8,878599 | 41,81822 |
| ENSMUSG00000095105 | Edaradd | -1,97 | 0,000987542 | 131,7793 | 61,84242 | 103,8013 | 593,78 | 418,5625 | 156,5959 |
| ENSMUSG00000031616 | Ednra | -2,88 | 1,05736E-05 | 96,9896 | 22,22462 | 83,90603 | 448,061 | 788,927 | 260,6966 |
| ENSMUSG00000022122 | Ednrb | 2,31 | 0,004868412 | 106,4777 | 130,4489 | 146,1868 | 11,89543 | 58,34508 | 8,007744 |
| ENSMUSG00000020467 | Efemp1 | -3,43 | 0,000966156 | 11,59658 | 5,797727 | 7,785095 | 12,88671 | 55,80834 | 201,0834 |
| ENSMUSG00000048915 | Efna5 | -3,65 | 0,030098874 | 1,054235 | 0,966288 | 0,865011 | 1,982571 | 8,878599 | 24,91298 |
| ENSMUSG00000003934 | Efnb3 | -1,28 | 0,032632677 | 63,25408 | 23,19091 | 25,08531 | 110,0327 | 82,44414 | 77,40819 |
| ENSMUSG00000026921 | Egfl7 | -1,15 | 0,031784699 | 171,8403 | 234,8079 | 259,5032 | 363,8018 | 296,7989 | 819,4592 |
| ENSMUSG00000020122 | Egfr | -2,38 | 0,0005052 | 32,68128 | 19,32576 | 44,98055 | 82,27669 | 109,0799 | 312,302 |
| ENSMUSG00000035105 | Egln3 | 1,20 | 6,75366E-07 | 1381,048 | 1305,455 | 1754,241 | 552,146 | 743,2656 | 642,399 |
| ENSMUSG00000037868 | Egr2 | 1,05 | 0,038169513 | 192,925 | 195,1901 | 222,3077 | 57,49456 | 79,90739 | 155,7061 |
| ENSMUSG00000074364 | Ehd2 | -1,40 | 0,005670237 | 323,6501 | 143,0106 | 345,1392 | 908,0175 | 795,2688 | 440,4259 |
| ENSMUSG00000027293 | Ehd4 | 1,05 | 0,032128094 | 7856,157 | 10106,4 | 9245,233 | 3167,157 | 2795,49 | 7206,97 |
| ENSMUSG00000012350 | Ehf | -4,40 | 0,004982361 | 2,108469 | 0 | 1,730021 | 19,82571 | 2,536743 | 57,83371 |
| ENSMUSG00000029613 | Eif2ak1 | -1,05 | 1,59914E-11 | 4950,686 | 4456,519 | 4607,046 | 10144,82 | 10190,1 | 8772,039 |
| ENSMUSG00000093661 | Eif4e3 | 1,08 | 2,4915E-11 | 3788,92 | 3741,466 | 3548,273 | 1947,876 | 1627,32 | 1659,383 |
| ENSMUSG00000087060 | Eldr | -2,08 | 0,000122856 | 103,315 | 239,6394 | 89,9611 | 421,2963 | 494,6648 | 918,2213 |
| ENSMUSG00000001542 | Ell2 | -2,03 | 6,23239E-07 | 1139,628 | 487,9753 | 587,3422 | 2841,024 | 3703,644 | 2482,401 |
| ENSMUSG00000017670 | Elmo2 | 1,44 | 8,26783E-14 | 9941,434 | 11696,91 | 9861,986 | 4343,813 | 3916,731 | 3360,583 |
| ENSMUSG00000014791 | Elmo3 | -1,57 | 8,08277E-10 | 186,5995 | 229,0102 | 169,5421 | 648,3007 | 471,8341 | 611,2578 |
| ENSMUSG00000038754 | Elovl3 | -3,37 | 0,000496611 | 2,108469 | 0,966288 | 2,595032 | 17,84314 | 25,36743 | 16,01549 |
| ENSMUSG00000021728 | Emb | 1,02 | 0,000448719 | 24637,47 | 17943 | 26727,1 | 11787,38 | 13373,71 | 8970,453 |
| ENSMUSG00000034164 | Emid1 | -1,65 | 0,000673586 | 286,7518 | 87,93219 | 174,7321 | 639,3791 | 578,3773 | 507,1571 |
| ENSMUSG00000058070 | Eml1 | -2,08 | 0,010229977 | 13,70505 | 6,764015 | 20,76025 | 39,65142 | 106,5432 | 30,25148 |
| ENSMUSG00000051166 | Eml5 | 1,75 | 1,40725E-06 | 1376,831 | 2483,36 | 1399,587 | 642,353 | 520,0322 | 403,0565 |
| ENSMUSG00000044072 | Eml6 | 1,44 | 2,27246E-05 | 378,4703 | 502,4697 | 460,1856 | 152,658 | 117,9585 | 223,3271 |
| ENSMUSG00000022505 | Emp2 | -2,02 | 0,002222026 | 30,57281 | 39,6178 | 70,93087 | 74,34641 | 204,2078 | 296,2865 |
| ENSMUSG00000022995 | Enah | -1,25 | 0,019227799 | 62,19985 | 35,75265 | 40,6555 | 62,45098 | 124,3004 | 143,2496 |
| ENSMUSG00000037419 | Endod1 | -1,68 | 3,02523E-08 | 2236,032 | 1195,298 | 1423,807 | 5094,216 | 5502,195 | 4964,801 |
| ENSMUSG00000028445 | Enho | -1,78 | 0,012771447 | 11,59658 | 22,22462 | 18,16522 | 43,61656 | 31,70928 | 103,2109 |
| ENSMUSG00000019989 | Enpp3 | -2,04 | 8,94601E-05 | 11,59658 | 12,56174 | 28,54535 | 64,43356 | 79,90739 | 74,73895 |
| ENSMUSG00000023960 | Enpp5 | -1,23 | 0,000193148 | 416,4227 | 351,7288 | 223,1727 | 816,8192 | 664,6266 | 833,6952 |
| ENSMUSG00000041608 | Entpd3 | -3,27 | 1,73471E-06 | 14,75929 | 4,831439 | 5,190064 | 46,59042 | 71,02879 | 118,3367 |
| ENSMUSG00000028906 | Epb41 | -2,25 | 6,50001E-16 | 14006,56 | 8160,3 | 9943,297 | 54049,84 | 55122,15 | 43321,01 |
| ENSMUSG00000024044 | Epb41l3 | -1,70 | 0,001462075 | 149,7013 | 97,59507 | 255,1781 | 347,9412 | 470,5658 | 817,6797 |
| ENSMUSG00000026383 | Epb41l5 | -1,74 | 0,004359858 | 61,14562 | 13,52803 | 40,6555 | 142,7451 | 130,6422 | 111,2187 |
| ENSMUSG00000023216 | Epb42 | -3,36 | 7,35487E-08 | 2487,994 | 447,3913 | 835,6002 | 15570,12 | 13836,66 | 9284,535 |
| ENSMUSG00000002808 | Epdr1 | -2,92 | 1,05379E-17 | 185,5453 | 87,93219 | 128,8866 | 824,7495 | 1069,237 | 1155,784 |
| ENSMUSG00000006445 | Epha2 | -1,40 | 0,003783674 | 309,945 | 107,2579 | 256,9081 | 686,9608 | 473,1025 | 621,9348 |
| ENSMUSG00000029710 | Ephb4 | -1,23 | 0,048215253 | 46,38633 | 47,3481 | 62,28076 | 91,19826 | 65,95531 | 207,3116 |
| ENSMUSG00000038776 | Ephx1 | -1,62 | 1,91199E-05 | 265,6672 | 146,8757 | 307,0788 | 723,6384 | 591,061 | 898,6469 |
| ENSMUSG00000022040 | Ephx2 | -1,38 | 0,025437136 | 32,68128 | 24,1572 | 32,00539 | 39,65142 | 69,76042 | 121,0059 |
| ENSMUSG00000006235 | Epor | -3,60 | 6,27616E-11 | 794,893 | 184,561 | 259,5032 | 5112,059 | 4553,453 | 5300,237 |
| ENSMUSG00000040600 | Eps8l3 | 5,53 | 0,00440975 | 8,433878 | 20,29204 | 19,89524 | 0 | 1,268371 | 0 |
| ENSMUSG00000062312 | Erbb2 | -2,31 | 2,09539E-05 | 25,30163 | 22,22462 | 23,35529 | 99,12855 | 63,41857 | 186,8474 |
| ENSMUSG00000018166 | Erbb3 | 1,54 | 0,000213561 | 784,3506 | 949,8609 | 924,6963 | 227,9957 | 209,2813 | 478,6852 |
| ENSMUSG00000030172 | Erc1 | -1,15 | 2,45827E-11 | 632,5408 | 591,3681 | 517,2763 | 1305,523 | 1229,052 | 1338,183 |
| ENSMUSG00000040640 | Erc2 | 4,84 | 8,26445E-06 | 23,19316 | 162,3364 | 100,3412 | 7,930284 | 0 | 1,779499 |
| ENSMUSG00000025198 | Erlin1 | 1,16 | 9,34542E-07 | 5662,295 | 5269,167 | 6254,892 | 2762,713 | 2918,522 | 1993,039 |
| ENSMUSG00000028644 | Ermap | -3,45 | 8,11033E-08 | 4031,394 | 633,8848 | 1372,772 | 24950,66 | 24565,82 | 16452,36 |
| ENSMUSG00000020715 | Ern1 | 1,67 | 1,53388E-13 | 9493,384 | 12919,27 | 9806,625 | 3459,586 | 2896,96 | 3746,735 |
| ENSMUSG00000057069 | Ero1lb | 1,03 | 0,001029979 | 4508,962 | 3695,085 | 3992,024 | 2441,536 | 2172,72 | 1360,427 |
| ENSMUSG00000029616 | Erp29 | 1,08 | 5,50825E-12 | 13317,09 | 12014,82 | 13689,66 | 6658,464 | 6014,617 | 5731,765 |
| ENSMUSG00000037482 | Erv3 | 10,00 | 6,865E-10 | 226,6605 | 383,6163 | 435,1003 | 0 | 0 | 0,889749 |
| ENSMUSG00000001946 | Esam | -2,94 | 0,000269046 | 226,6605 | 120,786 | 186,8423 | 424,2702 | 531,4476 | 3152,382 |
| ENSMUSG00000042379 | Esm1 | -2,22 | 0,009759322 | 99,09807 | 82,13446 | 167,8121 | 190,3268 | 200,4027 | 1234,972 |
| ENSMUSG00000019768 | Esr1 | 1,27 | 1,25756E-11 | 1103,784 | 981,7484 | 1261,185 | 473,8345 | 455,3453 | 458,2209 |
| ENSMUSG00000025366 | Esyt1 | 1,79 | 3,53094E-19 | 41952,22 | 43012,37 | 43906,21 | 14034,62 | 13163,16 | 10004,34 |
| ENSMUSG00000037681 | Esyt3 | -1,10 | 0,049446501 | 32,68128 | 30,92121 | 19,03023 | 76,32898 | 38,05114 | 61,39271 |
| ENSMUSG00000036617 | Etl4 | -1,28 | 0,007338913 | 47,44056 | 19,32576 | 35,46543 | 84,25926 | 86,24925 | 77,40819 |
| ENSMUSG00000032035 | Ets1 | -1,14 | 5,63053E-05 | 6543,635 | 5213,123 | 6193,476 | 16683,33 | 12809,28 | 9982,988 |
| ENSMUSG00000013089 | Etv5 | -1,32 | 6,06113E-06 | 255,1248 | 198,089 | 206,7375 | 406,427 | 621,502 | 618,3758 |
| ENSMUSG00000050248 | Evc2 | -1,65 | 0,010915865 | 11,59658 | 12,56174 | 10,38013 | 25,77342 | 27,90417 | 54,27471 |
| ENSMUSG00000043251 | Exoc3l | -1,45 | 0,00024234 | 122,2912 | 129,4826 | 68,33584 | 295,4031 | 220,6966 | 356,7895 |
| ENSMUSG00000021280 | Exoc3l4 | -2,86 | 0,001690707 | 49,54903 | 29,95492 | 47,57558 | 78,31155 | 109,0799 | 737,6022 |
| ENSMUSG00000034584 | Exph5 | 1,79 | 0,011462614 | 45,33209 | 42,51666 | 43,25053 | 4,956427 | 10,14697 | 22,24373 |
| ENSMUSG00000028838 | Extl1 | -2,82 | 1,70036E-05 | 5,271174 | 4,831439 | 3,460042 | 30,72985 | 29,17254 | 34,70023 |
| ENSMUSG00000025932 | Eya1 | -1,82 | 3,17812E-07 | 88,55572 | 45,41553 | 74,39091 | 257,7342 | 201,671 | 275,8223 |
| ENSMUSG00000031444 | F10 | 2,11 | 8,19106E-06 | 12274,46 | 6341,747 | 8698,546 | 1166,743 | 1884,8 | 3272,498 |
| ENSMUSG00000038235 | F11r | -2,45 | 0,007529853 | 253,0163 | 191,325 | 241,338 | 271,6122 | 437,5881 | 3042,053 |
| ENSMUSG00000039109 | F13a1 | 3,06 | 1,00996E-14 | 102982,9 | 118428,2 | 117198,6 | 15681,14 | 18168,15 | 6743,41 |
| ENSMUSG00000027249 | F2 | -3,87 | 0,00577731 | 1,054235 | 0,966288 | 0,865011 | 7,930284 | 8,878599 | 24,91298 |
| ENSMUSG00000048376 | F2r | -1,98 | 0,014609171 | 577,7206 | 261,864 | 332,1641 | 545,207 | 709,0196 | 3365,032 |
| ENSMUSG00000021678 | F2rl1 | 1,63 | 0,000957476 | 367,9279 | 269,5943 | 159,1619 | 80,29412 | 55,80834 | 119,2264 |
| ENSMUSG00000021675 | F2rl2 | -2,83 | 0,001495299 | 562,9614 | 443,5261 | 640,1078 | 895,1308 | 1263,298 | 9533,664 |
| ENSMUSG00000050147 | F2rl3 | -3,14 | 0,000524306 | 226,6605 | 154,606 | 191,1673 | 376,6885 | 498,4699 | 4168,476 |
| ENSMUSG00000028128 | F3 | -1,39 | 0,024124123 | 11,59658 | 28,98863 | 14,70518 | 50,55556 | 58,34508 | 36,47972 |
| ENSMUSG00000026579 | F5 | -2,29 | 3,4746E-07 | 3364,063 | 1500,645 | 1850,258 | 6890,425 | 10725,35 | 15284,11 |
| ENSMUSG00000031443 | F7 | 4,68 | 8,62138E-16 | 292,023 | 393,2791 | 795,8097 | 29,73856 | 20,29394 | 8,007744 |
| ENSMUSG00000086425 | F730016J06Rik | -1,43 | 8,08247E-05 | 1018,391 | 1377,926 | 937,6715 | 3645,948 | 3475,337 | 1883,599 |
| ENSMUSG00000031196 | F8 | -1,52 | 0,003017378 | 102,2608 | 45,41553 | 89,9611 | 143,7364 | 219,4282 | 316,7508 |
| ENSMUSG00000075184 | F930017D23Rik | -3,56 | 2,09164E-06 | 173,9487 | 14,49432 | 63,14577 | 1064,641 | 1054,017 | 847,9311 |
| ENSMUSG00000062515 | Fabp4 | -1,50 | 0,004326131 | 508,1411 | 471,5484 | 869,3356 | 912,9739 | 1591,806 | 2715,515 |
| ENSMUSG00000024665 | Fads2 | -1,44 | 0,000786592 | 528,1716 | 338,2007 | 563,1219 | 965,512 | 971,5724 | 1949,441 |
| ENSMUSG00000024664 | Fads3 | -1,04 | 0,000622817 | 530,2801 | 365,2568 | 445,4805 | 746,438 | 854,8823 | 1150,446 |
| ENSMUSG00000045316 | Fahd1 | -1,74 | 2,94486E-12 | 391,1211 | 300,5155 | 276,8034 | 1188,551 | 1154,218 | 896,8674 |
| ENSMUSG00000056069 | Fam105a | 1,11 | 3,58482E-05 | 3740,425 | 3971,443 | 4105,34 | 2104,499 | 1320,375 | 2059,77 |
| ENSMUSG00000049687 | Fam109b | -2,71 | 2,30891E-28 | 543,9851 | 332,403 | 394,4448 | 2641,776 | 2823,395 | 2863,213 |
| ENSMUSG00000049119 | Fam110b | -2,77 | 0,000659984 | 41,11515 | 45,41553 | 53,63066 | 119,9455 | 111,6167 | 723,3662 |
| ENSMUSG00000026796 | Fam129b | 1,47 | 0,000165246 | 5455,665 | 5265,302 | 6170,986 | 1712,941 | 3078,337 | 1310,601 |
| ENSMUSG00000050821 | Fam131a | -1,70 | 0,048061888 | 110,6946 | 84,06704 | 93,42114 | 94,17212 | 149,6678 | 693,1148 |
| ENSMUSG00000037709 | Fam13a | -1,23 | 0,010889018 | 28,46434 | 23,19091 | 35,46543 | 71,37255 | 46,92974 | 85,41594 |
| ENSMUSG00000035095 | Fam167a | -2,17 | 0,000354224 | 35,84398 | 9,662878 | 35,46543 | 118,9543 | 84,98088 | 161,0446 |
| ENSMUSG00000041817 | Fam169a | -1,31 | 0,024243427 | 26,35587 | 23,19091 | 43,25053 | 60,46841 | 116,6902 | 55,16446 |
| ENSMUSG00000074071 | Fam169b | -1,31 | 0,037370324 | 318,3789 | 102,4265 | 467,9707 | 699,8475 | 886,5916 | 619,2656 |
| ENSMUSG00000046826 | Fam187b | 1,06 | 0,028851063 | 46,38633 | 39,6178 | 43,25053 | 24,78214 | 20,29394 | 16,90524 |
| ENSMUSG00000032657 | Fam189b | 1,61 | 2,73647E-05 | 2117,958 | 2584,82 | 2223,942 | 607,658 | 509,8853 | 1145,997 |
| ENSMUSG00000046500 | Fam19a4 | 3,07 | 9,49752E-05 | 24,2474 | 49,28068 | 33,73541 | 3,965142 | 7,610228 | 1,779499 |
| ENSMUSG00000020614 | Fam20a | -1,58 | 0,03690922 | 10,54235 | 15,4606 | 30,27537 | 49,56427 | 30,44091 | 88,08519 |
| ENSMUSG00000025854 | Fam20c | 3,53 | 1,76287E-32 | 1300,926 | 1786,666 | 1671,2 | 151,6667 | 168,6934 | 94,31343 |
| ENSMUSG00000027495 | Fam210b | -2,18 | 5,00287E-12 | 1346,258 | 711,1878 | 888,3659 | 5183,432 | 4407,59 | 3784,104 |
| ENSMUSG00000042106 | Fam212a | -1,28 | 0,018310725 | 598,8053 | 473,481 | 477,4858 | 844,5752 | 725,5084 | 2195,901 |
| ENSMUSG00000021792 | Fam213a | -3,08 | 1,04688E-14 | 1177,58 | 488,9416 | 762,9393 | 5914,009 | 9059,976 | 5552,926 |
| ENSMUSG00000036002 | Fam214b | -1,50 | 5,19522E-21 | 2407,872 | 2548,101 | 2233,457 | 7205,654 | 6118,623 | 6963,179 |
| ENSMUSG00000083012 | Fam220a | -3,52 | 5,1986E-17 | 164,4606 | 65,70757 | 86,50106 | 1066,623 | 958,8887 | 1591,762 |
| ENSMUSG00000080254 | Fam220-ps | -3,70 | 0,018063698 | 1,054235 | 0 | 6,055074 | 2,973856 | 50,73485 | 40,92847 |
| ENSMUSG00000024187 | Fam234a | 2,38 | 3,18573E-34 | 7218,345 | 8070,436 | 9841,225 | 1645,534 | 1492,873 | 1692,303 |
| ENSMUSG00000030207 | Fam234b | -1,48 | 0,000137543 | 1513,881 | 1065,815 | 895,286 | 2902,484 | 4515,402 | 2260,853 |
| ENSMUSG00000032265 | Fam46a | 1,15 | 4,8446E-11 | 7210,966 | 6551,431 | 7258,304 | 3592,419 | 2975,599 | 2928,165 |
| ENSMUSG00000044468 | Fam46c | -3,30 | 2,82777E-12 | 6418,181 | 1906,486 | 2898,65 | 41804,49 | 38028,31 | 30392,06 |
| ENSMUSG00000020589 | Fam49a | 1,98 | 1,42478E-07 | 5427,2 | 6477,993 | 6216,831 | 1381,852 | 932,2529 | 2269,751 |
| ENSMUSG00000030956 | Fam53b | -1,45 | 3,64552E-05 | 5615,908 | 3850,657 | 4156,376 | 15523,53 | 13605,82 | 8019,311 |
| ENSMUSG00000036186 | Fam69b | -1,21 | 0,035477989 | 54,82021 | 90,83105 | 102,0712 | 111,024 | 158,5464 | 304,2943 |
| ENSMUSG00000020401 | Fam71b | -2,03 | 1,19397E-05 | 25,30163 | 22,22462 | 31,14038 | 100,1198 | 152,2046 | 71,17995 |
| ENSMUSG00000060568 | Fam78b | 2,78 | 3,18824E-18 | 176,0572 | 192,2913 | 225,7678 | 21,80828 | 31,70928 | 32,92073 |
| ENSMUSG00000051225 | Fam83a | 5,75 | 1,22361E-31 | 425,9108 | 276,3583 | 542,3616 | 7,930284 | 3,805114 | 10,67699 |
| ENSMUSG00000032358 | Fam83b | 7,90 | 2,18344E-06 | 64,30832 | 94,6962 | 84,77104 | 0 | 0 | 0,889749 |
| ENSMUSG00000072568 | Fam84b | 2,86 | 1,51602E-27 | 3307,134 | 3641,939 | 2644,337 | 400,4793 | 361,4858 | 557,8729 |
| ENSMUSG00000025555 | Farp1 | -1,02 | 0,005464615 | 121,237 | 88,89848 | 110,7214 | 157,6144 | 280,3101 | 214,4296 |
| ENSMUSG00000006219 | Fblim1 | -1,40 | 0,009722229 | 65,36255 | 34,78636 | 114,1814 | 157,6144 | 196,5976 | 210,8706 |
| ENSMUSG00000006369 | Fbln1 | 2,47 | 3,66397E-11 | 503,9242 | 693,7946 | 1044,933 | 105,0763 | 134,4474 | 165,4934 |
| ENSMUSG00000021186 | Fbln5 | -1,72 | 0,011747614 | 268,8299 | 99,52764 | 247,393 | 262,6906 | 646,8694 | 1115,746 |
| ENSMUSG00000048232 | Fbxo10 | -1,68 | 2,91E-12 | 533,4428 | 716,0193 | 494,7861 | 2089,63 | 1853,091 | 1648,706 |
| ENSMUSG00000047648 | Fbxo30 | -1,44 | 5,77359E-07 | 1787,982 | 1248,444 | 1407,372 | 4725,458 | 4375,881 | 2992,227 |
| ENSMUSG00000001786 | Fbxo7 | -1,21 | 5,29577E-08 | 1658,311 | 1213,657 | 1371,042 | 3420,926 | 3528,609 | 2839,19 |
| ENSMUSG00000026415 | Fcamr | -1,90 | 0,01949468 | 13,70505 | 9,662878 | 8,650106 | 70,38127 | 17,7572 | 30,25148 |
| ENSMUSG00000005339 | Fcer1a | -1,58 | 0,000384295 | 288,8603 | 299,5492 | 117,6414 | 613,6057 | 653,2112 | 847,9311 |
| ENSMUSG00000005540 | Fcer2a | -2,75 | 4,41198E-10 | 305,7281 | 195,1901 | 188,5723 | 2068,813 | 783,8535 | 1779,499 |
| ENSMUSG00000026656 | Fcgr2b | 1,53 | 7,78645E-07 | 11594,47 | 12797,52 | 13693,98 | 4837,473 | 5488,243 | 2901,473 |
| ENSMUSG00000059089 | Fcgr4 | -1,33 | 0,004748286 | 1551,834 | 673,5026 | 845,9804 | 1955,806 | 2168,915 | 3604,375 |
| ENSMUSG00000042474 | Fcmr | -2,48 | 1,09973E-06 | 492,3276 | 142,0443 | 378,8746 | 2459,379 | 1812,503 | 1363,986 |
| ENSMUSG00000026938 | Fcna | -3,13 | 1,88344E-06 | 110,6946 | 138,1792 | 502,5711 | 1584,074 | 1647,614 | 3340,119 |
| ENSMUSG00000059994 | Fcrl1 | -2,76 | 1,33991E-09 | 248,7994 | 74,40416 | 150,5118 | 1121,144 | 914,4957 | 1167,351 |
| ENSMUSG00000048031 | Fcrl5 | -3,95 | 0,000106358 | 15,81352 | 4,831439 | 12,11015 | 35,68628 | 59,61345 | 409,2847 |
| ENSMUSG00000038421 | Fcrla | -1,89 | 0,000640147 | 1443,247 | 476,3799 | 1051,853 | 5285,534 | 3647,836 | 2048,203 |
| ENSMUSG00000015852 | Fcrls | 1,76 | 0,032776936 | 13,70505 | 16,42689 | 22,49028 | 4,956427 | 6,341857 | 4,448747 |
| ENSMUSG00000024588 | Fech | -2,80 | 9,44106E-18 | 5609,583 | 2584,82 | 3752,416 | 28704,65 | 28307,51 | 25937,08 |
| ENSMUSG00000037106 | Fer1l6 | -2,03 | 1,97272E-05 | 24,2474 | 19,32576 | 51,03562 | 139,7713 | 116,6902 | 131,6829 |
| ENSMUSG00000044453 | Ffar1 | -1,82 | 4,58309E-05 | 20,03046 | 18,35947 | 18,16522 | 86,24184 | 60,88182 | 52,49521 |
| ENSMUSG00000051314 | Ffar2 | -1,26 | 3,53891E-06 | 726,3677 | 702,4912 | 653,948 | 1832,887 | 1953,292 | 1201,162 |
| ENSMUSG00000036585 | Fgf1 | -3,00 | 8,62546E-05 | 12,65082 | 16,42689 | 57,0907 | 168,5185 | 119,2269 | 402,1667 |
| ENSMUSG00000031137 | Fgf13 | -1,83 | 0,000364936 | 34,78975 | 18,35947 | 38,92548 | 146,7102 | 109,0799 | 71,17995 |
| ENSMUSG00000057967 | Fgf18 | 7,27 | 0,0471599 | 3,162704 | 5,797727 | 8,650106 | 0 | 0 | 0 |
| ENSMUSG00000031074 | Fgf3 | -1,25 | 0,010229977 | 68,52526 | 63,77499 | 107,2613 | 142,7451 | 144,5943 | 281,1608 |
| ENSMUSG00000030849 | Fgfr2 | -1,35 | 0,024431256 | 80,12184 | 70,53901 | 95,15116 | 135,8061 | 120,4953 | 368,3562 |
| ENSMUSG00000054252 | Fgfr3 | -1,49 | 0,016778876 | 12,65082 | 18,35947 | 12,97516 | 23,79085 | 52,00322 | 48,04647 |
| ENSMUSG00000039899 | Fgl2 | 1,09 | 1,67431E-05 | 8221,977 | 6391,994 | 5407,181 | 3525,011 | 3023,797 | 2845,418 |
| ENSMUSG00000028874 | Fgr | -1,29 | 0,000119832 | 3519,036 | 6931,182 | 4083,715 | 11768,54 | 11989,91 | 11765,16 |
| ENSMUSG00000026526 | Fh1 | 1,08 | 1,34608E-08 | 5781,423 | 7518,685 | 7148,447 | 3207,8 | 3423,334 | 3062,517 |
| ENSMUSG00000041842 | Fhdc1 | -3,09 | 4,1313E-09 | 1374,722 | 387,4814 | 534,5765 | 7770,687 | 7170,103 | 4554,627 |
| ENSMUSG00000023092 | Fhl1 | -4,45 | 0,000404052 | 178,1657 | 42,51666 | 87,36607 | 558,0937 | 804,1474 | 5357,181 |
| ENSMUSG00000008136 | Fhl2 | -2,34 | 5,38211E-13 | 75,9049 | 40,58409 | 57,0907 | 253,7691 | 333,5817 | 290,0583 |
| ENSMUSG00000032966 | Fkbp1a | 1,02 | 7,21646E-06 | 4884,27 | 4597,597 | 5587,103 | 2177,854 | 2269,116 | 2979,771 |
| ENSMUSG00000020635 | Fkbp1b | 2,70 | 5,45074E-32 | 783,2964 | 1093,838 | 1050,123 | 140,7625 | 159,8148 | 149,4779 |
| ENSMUSG00000025278 | Flnb | 1,80 | 7,2761E-25 | 7631,605 | 8597,063 | 7205,538 | 2098,551 | 2512,644 | 2122,052 |
| ENSMUSG00000020357 | Flt4 | -1,42 | 0,001229852 | 137,0505 | 102,4265 | 149,6468 | 285,4902 | 232,112 | 524,0624 |
| ENSMUSG00000044042 | Fmn1 | 1,84 | 3,61892E-06 | 337,3551 | 310,1784 | 400,4999 | 75,3377 | 68,49205 | 147,6984 |
| ENSMUSG00000036053 | Fmnl2 | -1,16 | 1,72479E-05 | 1122,76 | 1573,117 | 1096,833 | 2668,54 | 3352,305 | 2451,26 |
| ENSMUSG00000040181 | Fmo1 | -1,12 | 0,020392699 | 70,63373 | 69,57272 | 92,55613 | 110,0327 | 142,0576 | 254,4683 |
| ENSMUSG00000040170 | Fmo2 | -2,48 | 0,000896234 | 51,6575 | 33,82007 | 92,55613 | 101,1111 | 282,8468 | 606,8091 |
| ENSMUSG00000025175 | Fn3k | -3,69 | 1,59314E-09 | 361,6025 | 84,06704 | 118,5065 | 2837,059 | 3088,484 | 1352,419 |
| ENSMUSG00000039253 | Fn3krp | -2,00 | 7,03303E-10 | 821,2489 | 478,3125 | 499,9761 | 2200,654 | 2983,209 | 2026,849 |
| ENSMUSG00000039735 | Fnbp1l | 1,23 | 1,23135E-05 | 2039,944 | 3000,324 | 2800,039 | 913,9652 | 1089,531 | 1333,734 |
| ENSMUSG00000039286 | Fndc3b | 1,27 | 2,18005E-06 | 4174,77 | 3984,005 | 4171,081 | 1573,17 | 2218,381 | 1323,057 |
| ENSMUSG00000003545 | Fosb | -1,09 | 0,026694898 | 26,35587 | 25,12348 | 15,57019 | 51,54684 | 45,66137 | 44,48747 |
| ENSMUSG00000050295 | Foxc1 | -1,53 | 0,031152194 | 23,19316 | 25,12348 | 22,49028 | 44,60785 | 35,5144 | 123,6752 |
| ENSMUSG00000048756 | Foxo3 | -1,09 | 5,56971E-07 | 6403,422 | 5157,078 | 5662,359 | 14070,31 | 12164,95 | 10315,75 |
| ENSMUSG00000038415 | Foxq1 | -4,14 | 0,004748286 | 1,054235 | 0,966288 | 0,865011 | 33,70371 | 5,073485 | 11,56674 |
| ENSMUSG00000016552 | Foxred2 | 1,18 | 8,38788E-07 | 3827,926 | 3633,242 | 4752,368 | 1947,876 | 1991,343 | 1467,197 |
| ENSMUSG00000045551 | Fpr1 | -2,14 | 3,32938E-16 | 1105,892 | 1519,971 | 1154,789 | 6345,218 | 5979,102 | 4294,82 |
| ENSMUSG00000052270 | Fpr2 | -2,09 | 1,61434E-59 | 1786,928 | 1660,082 | 1653,035 | 7566,482 | 7062,292 | 7126,003 |
| ENSMUSG00000048285 | Frmd6 | -1,75 | 9,58435E-05 | 260,396 | 163,3026 | 333,0291 | 577,9194 | 720,4349 | 1241,2 |
| ENSMUSG00000049176 | Frmpd4 | -2,11 | 0,031892012 | 6,325408 | 8,69659 | 4,325053 | 47,5817 | 26,6358 | 8,897494 |
| ENSMUSG00000033386 | Frrs1 | 1,37 | 8,52127E-19 | 3142,674 | 3659,332 | 3351,916 | 1223,246 | 1341,937 | 1355,088 |
| ENSMUSG00000021765 | Fst | -1,95 | 0,003021623 | 27,4101 | 19,32576 | 25,08531 | 37,66885 | 88,78599 | 151,2574 |
| ENSMUSG00000020325 | Fstl3 | -1,55 | 0,004563063 | 15,81352 | 16,42689 | 20,76025 | 44,60785 | 36,78277 | 73,8492 |
| ENSMUSG00000025466 | Fuom | 1,81 | 2,58924E-07 | 281,4807 | 354,6276 | 399,6349 | 84,25926 | 73,56554 | 136,1317 |
| ENSMUSG00000046152 | Fut10 | 1,48 | 0,001149478 | 385,8499 | 329,5041 | 519,0064 | 77,32027 | 173,7669 | 190,4064 |
| ENSMUSG00000049307 | Fut4 | 1,84 | 2,06604E-41 | 2748,39 | 2835,088 | 2771,494 | 823,7582 | 776,2433 | 735,8227 |
| ENSMUSG00000036587 | Fut7 | 1,42 | 2,8263E-18 | 2599,743 | 2500,753 | 2309,578 | 980,3813 | 829,5149 | 952,0318 |
| ENSMUSG00000078612 | Fyb2 | -2,54 | 0,021002687 | 4,216939 | 6,764015 | 2,595032 | 18,83442 | 7,610228 | 51,60546 |
| ENSMUSG00000019843 | Fyn | -1,57 | 0,007788334 | 1528,64 | 883,1871 | 1033,688 | 2189,75 | 1827,723 | 6218,458 |
| ENSMUSG00000044674 | Fzd1 | 4,14 | 1,1261E-31 | 2452,15 | 2010,845 | 2750,734 | 76,32898 | 172,4985 | 161,0446 |
| ENSMUSG00000041075 | Fzd7 | 1,14 | 7,2336E-07 | 1206,045 | 840,6704 | 1072,613 | 442,1133 | 494,6648 | 480,4647 |
| ENSMUSG00000049551 | Fzd9 | -2,44 | 0,001778911 | 3,162704 | 4,831439 | 7,785095 | 20,81699 | 22,83068 | 42,70797 |
| ENSMUSG00000020235 | Fzr1 | -1,26 | 3,73645E-08 | 5628,559 | 4052,611 | 4757,558 | 12891,67 | 11759,07 | 9899,351 |
| ENSMUSG00000009633 | G0s2 | -1,84 | 4,21131E-11 | 640,9747 | 502,4697 | 384,9297 | 1805,131 | 2096,618 | 1563,29 |
| ENSMUSG00000072844 | G530011O06Rik | 1,59 | 0,012687025 | 31,62704 | 38,65151 | 32,8704 | 16,85185 | 11,41534 | 6,228246 |
| ENSMUSG00000073414 | G6b | -5,55 | 0,00032249 | 113,8574 | 9,662878 | 27,68034 | 525,3813 | 657,0164 | 5890,141 |
| ENSMUSG00000034793 | G6pc3 | 1,57 | 8,99694E-12 | 4988,639 | 5372,56 | 5587,968 | 1503,78 | 1631,126 | 2231,491 |
| ENSMUSG00000031714 | Gab1 | -1,51 | 5,57905E-07 | 396,3923 | 222,2462 | 284,5885 | 752,3857 | 870,1027 | 952,9216 |
| ENSMUSG00000031950 | Gabarapl2 | -1,23 | 3,328E-16 | 1558,159 | 1352,803 | 1405,642 | 3541,863 | 3390,357 | 3211,995 |
| ENSMUSG00000070880 | Gad1 | -3,13 | 1,40697E-05 | 5,271174 | 5,797727 | 4,325053 | 34,69499 | 27,90417 | 71,17995 |
| ENSMUSG00000036390 | Gadd45a | -1,79 | 8,87831E-14 | 839,1709 | 577,8401 | 586,4772 | 2344,39 | 2492,35 | 2080,234 |
| ENSMUSG00000021453 | Gadd45g | -1,51 | 4,07569E-05 | 415,3685 | 253,1674 | 232,6878 | 636,4053 | 871,3711 | 1049,015 |
| ENSMUSG00000021903 | Galnt15 | -1,26 | 0,022928183 | 84,33878 | 44,44924 | 93,42114 | 143,7364 | 117,9585 | 271,3736 |
| ENSMUSG00000037280 | Galnt6 | -1,17 | 0,001145797 | 947,757 | 552,7166 | 740,4491 | 1257,941 | 1612,1 | 2184,335 |
| ENSMUSG00000047261 | Gap43 | 8,25 | 3,1651E-38 | 633,5951 | 665,7723 | 854,6305 | 0,991285 | 0 | 5,338496 |
| ENSMUSG00000042680 | Garem1 | -2,69 | 1,34259E-07 | 271,9926 | 124,6511 | 97,7462 | 1021,024 | 1500,483 | 666,4223 |
| ENSMUSG00000034201 | Gas2l1 | -3,17 | 0,000186018 | 503,9242 | 316,9424 | 382,3347 | 1042,832 | 1081,921 | 8732 |
| ENSMUSG00000031451 | Gas6 | -1,99 | 0,000961802 | 790,6761 | 573,0087 | 900,476 | 1270,828 | 2115,643 | 5619,657 |
| ENSMUSG00000031162 | Gata1 | -3,00 | 2,07335E-09 | 1413,729 | 376,8522 | 745,6391 | 5666,188 | 6015,885 | 8548,712 |
| ENSMUSG00000015053 | Gata2 | -1,20 | 0,018450739 | 854,9844 | 1037,793 | 815,705 | 1456,198 | 1259,493 | 3512,731 |
| ENSMUSG00000026829 | Gbgt1 | -1,64 | 0,016059748 | 15,81352 | 6,764015 | 15,57019 | 30,72985 | 30,44091 | 56,94396 |
| ENSMUSG00000028270 | Gbp2 | 2,11 | 0,003084345 | 6385,5 | 1736,419 | 1484,358 | 530,3377 | 493,3965 | 1196,713 |
| ENSMUSG00000040264 | Gbp2b | 2,09 | 4,32405E-05 | 3383,039 | 1179,837 | 1266,375 | 344,9673 | 456,6137 | 565,8806 |
| ENSMUSG00000028268 | Gbp3 | 2,14 | 1,09734E-11 | 5121,472 | 2840,886 | 3156,424 | 823,7582 | 720,4349 | 979,6141 |
| ENSMUSG00000040253 | Gbp7 | 1,38 | 0,001126465 | 5784,586 | 2884,369 | 3513,673 | 1083,475 | 1518,24 | 2090,021 |
| ENSMUSG00000034486 | Gbx2 | -3,24 | 0,001188364 | 18,97623 | 15,4606 | 17,30021 | 38,66013 | 44,393 | 405,7257 |
| ENSMUSG00000026893 | Gca | -1,61 | 0,002618919 | 1984,07 | 1541,229 | 935,0764 | 4676,885 | 6795,934 | 2173,658 |
| ENSMUSG00000037580 | Gch1 | -1,59 | 1,91947E-06 | 1512,827 | 1068,714 | 1267,241 | 4770,066 | 4261,728 | 2525,109 |
| ENSMUSG00000032350 | Gclc | -1,17 | 2,68545E-05 | 2936,044 | 2149,99 | 2369,264 | 6311,515 | 6251,802 | 4206,735 |
| ENSMUSG00000028124 | Gclm | -1,97 | 1,73798E-12 | 3130,023 | 1735,453 | 2435,87 | 8776,842 | 9583,814 | 10152,93 |
| ENSMUSG00000091387 | Gcnt4 | 2,01 | 0,042547407 | 14,75929 | 32,85379 | 53,63066 | 1,982571 | 12,68371 | 10,67699 |
| ENSMUSG00000022659 | Gcsam | -3,22 | 0,00040525 | 177,1114 | 103,3928 | 141,8617 | 275,5774 | 421,0993 | 3248,475 |
| ENSMUSG00000021943 | Gdf10 | -2,88 | 7,85098E-05 | 4,216939 | 8,69659 | 4,325053 | 34,69499 | 26,6358 | 64,9517 |
| ENSMUSG00000025352 | Gdf11 | -1,21 | 0,000866728 | 119,1285 | 78,26931 | 106,3963 | 270,6209 | 166,1566 | 265,1453 |
| ENSMUSG00000018238 | Gdf9 | 2,24 | 1,32253E-05 | 84,33878 | 161,3701 | 88,23108 | 24,78214 | 13,95208 | 31,14123 |
| ENSMUSG00000061666 | Gdpd1 | -2,19 | 9,79913E-13 | 63,25408 | 39,6178 | 62,28076 | 216,1002 | 256,211 | 280,2711 |
| ENSMUSG00000019359 | Gdpd2 | -1,37 | 0,006370697 | 189,7623 | 141,078 | 170,4071 | 224,0305 | 394,4635 | 674,43 |
| ENSMUSG00000020932 | Gfap | -3,53 | 1,18614E-10 | 126,5082 | 27,05606 | 46,71057 | 756,3508 | 766,0963 | 785,6487 |
| ENSMUSG00000026815 | Gfi1b | -3,12 | 9,96394E-13 | 634,6493 | 232,8754 | 284,5885 | 2960,97 | 2917,254 | 4152,46 |
| ENSMUSG00000051335 | Gfod1 | -1,09 | 0,00134789 | 1759,518 | 1197,231 | 1306,166 | 3719,303 | 3211,516 | 2113,155 |
| ENSMUSG00000022103 | Gfra2 | -2,21 | 5,75086E-06 | 170,786 | 54,11212 | 162,622 | 687,9521 | 483,2495 | 618,3758 |
| ENSMUSG00000027316 | Gfra4 | -2,50 | 0,009242943 | 3,162704 | 2,898863 | 7,785095 | 23,79085 | 11,41534 | 43,59772 |
| ENSMUSG00000041625 | Ggact | -1,53 | 7,8995E-05 | 55,87444 | 46,38181 | 64,01078 | 133,8235 | 126,8371 | 217,9886 |
| ENSMUSG00000006345 | Ggt1 | -2,02 | 0,000124814 | 52,71174 | 37,68522 | 52,76565 | 167,5272 | 101,4697 | 309,6328 |
| ENSMUSG00000006344 | Ggt5 | -1,51 | 3,2853E-05 | 269,8841 | 517,9303 | 331,2991 | 1049,771 | 832,0516 | 1300,814 |
| ENSMUSG00000090019 | Gimap1 | -1,59 | 0,000157182 | 432,2362 | 200,9879 | 356,3844 | 964,5208 | 706,4828 | 1312,38 |
| ENSMUSG00000039264 | Gimap3 | -1,36 | 0,004295333 | 817,0319 | 323,7064 | 433,3703 | 1486,928 | 938,5948 | 1621,123 |
| ENSMUSG00000043505 | Gimap5 | -1,42 | 0,003677905 | 298,3484 | 123,6848 | 224,0377 | 462,9303 | 436,3197 | 823,9079 |
| ENSMUSG00000047867 | Gimap6 | -1,59 | 8,60337E-05 | 1554,996 | 693,7946 | 1191,12 | 3387,222 | 2586,209 | 4361,551 |
| ENSMUSG00000064262 | Gimap8 | -1,46 | 0,00024364 | 727,422 | 424,2003 | 397,9049 | 1500,806 | 977,9143 | 1793,735 |
| ENSMUSG00000051124 | Gimap9 | -1,37 | 0,000671238 | 546,0936 | 221,2799 | 431,6403 | 1113,214 | 982,9878 | 993,85 |
| ENSMUSG00000050953 | Gja1 | -2,08 | 1,94892E-05 | 246,6909 | 114,9882 | 177,3272 | 405,4358 | 806,6842 | 1072,148 |
| ENSMUSG00000030048 | Gkn3 | 7,17 | 5,36722E-12 | 87,50148 | 204,853 | 142,7267 | 0 | 0 | 2,669248 |
| ENSMUSG00000036395 | Glb1l2 | 5,02 | 7,78954E-18 | 94,88113 | 168,1341 | 168,6771 | 2,973856 | 5,073485 | 5,338496 |
| ENSMUSG00000056888 | Glipr1 | 1,53 | 2,77726E-15 | 9431,184 | 8911,106 | 8675,191 | 3577,549 | 3159,513 | 2606,076 |
| ENSMUSG00000052942 | Glis3 | 1,23 | 0,000107107 | 260,396 | 319,8413 | 453,2655 | 142,7451 | 149,6678 | 148,5881 |
| ENSMUSG00000021102 | Glrx5 | -1,89 | 1,0467E-17 | 2116,903 | 1447,499 | 1873,613 | 6588,083 | 6246,729 | 7383,14 |
| ENSMUSG00000044005 | Gls2 | -2,29 | 1,12219E-08 | 21,08469 | 22,22462 | 19,03023 | 131,841 | 90,05436 | 82,74669 |
| ENSMUSG00000021794 | Glud1 | 1,02 | 7,13176E-06 | 14845,73 | 18020,3 | 18999,96 | 9529,227 | 8976,264 | 7077,066 |
| ENSMUSG00000062093 | Gm10110 | -1,07 | 0,035154543 | 48,4948 | 20,29204 | 38,06047 | 65,42484 | 76,10228 | 82,74669 |
| ENSMUSG00000063087 | Gm10125 | -1,51 | 0,013243454 | 9,488113 | 13,52803 | 7,785095 | 30,72985 | 25,36743 | 31,14123 |
| ENSMUSG00000068601 | Gm10244 | -2,89 | 0,007473054 | 0 | 2,898863 | 6,055074 | 17,84314 | 16,48883 | 33,81048 |
| ENSMUSG00000070111 | Gm10286 | -2,01 | 0,011210784 | 7,379643 | 3,865151 | 6,055074 | 15,86057 | 20,29394 | 32,92073 |
| ENSMUSG00000070717 | Gm10300 | -1,68 | 0,004609179 | 10,54235 | 12,56174 | 6,920085 | 36,67756 | 27,90417 | 31,14123 |
| ENSMUSG00000072589 | Gm10371 | -3,90 | 2,08074E-06 | 71,68796 | 3,865151 | 26,81533 | 433,1917 | 692,5308 | 395,0487 |
| ENSMUSG00000072769 | Gm10419 | -2,92 | 0,003131272 | 26,35587 | 13,52803 | 31,14038 | 50,55556 | 52,00322 | 433,3079 |
| ENSMUSG00000097316 | Gm10516 | -1,10 | 0,032114135 | 36,89822 | 21,25833 | 18,16522 | 54,5207 | 50,73485 | 56,94396 |
| ENSMUSG00000073628 | Gm10552 | -1,70 | 0,019277461 | 28,46434 | 12,56174 | 9,515116 | 52,53813 | 78,63902 | 32,03098 |
| ENSMUSG00000097649 | Gm10561 | -2,66 | 0,008803307 | 4,216939 | 0,966288 | 2,595032 | 16,85185 | 21,56231 | 10,67699 |
| ENSMUSG00000099137 | Gm10603 | -2,02 | 2,33059E-08 | 205,5758 | 125,6174 | 119,3715 | 451,0349 | 776,2433 | 600,5808 |
| ENSMUSG00000090174 | Gm10612 | 2,58 | 4,4435E-14 | 195,0334 | 239,6394 | 175,5971 | 38,66013 | 40,58788 | 24,02323 |
| ENSMUSG00000073018 | Gm1070 | 1,66 | 0,001563036 | 123,3455 | 59,90984 | 83,04102 | 23,79085 | 20,29394 | 39,14897 |
| ENSMUSG00000085331 | Gm11274 | -3,45 | 0,0043242 | 1,054235 | 2,898863 | 8,650106 | 17,84314 | 15,22046 | 106,7699 |
| ENSMUSG00000084788 | Gm11342 | -3,59 | 0,000676559 | 3,162704 | 1,932576 | 5,190064 | 18,83442 | 16,48883 | 88,97494 |
| ENSMUSG00000083165 | Gm11344 | -2,36 | 0,025394117 | 5,271174 | 0,966288 | 3,460042 | 17,84314 | 8,878599 | 22,24373 |
| ENSMUSG00000080927 | Gm11430 | 10,41 | 0,001018231 | 23,19316 | 80,20189 | 51,90064 | 0 | 0 | 0 |
| ENSMUSG00000085143 | Gm11520 | -1,20 | 0,038065708 | 11,59658 | 11,59545 | 12,97516 | 27,75599 | 27,90417 | 27,58223 |
| ENSMUSG00000086795 | Gm11579 | 2,94 | 2,69366E-05 | 27,4101 | 46,38181 | 57,0907 | 3,965142 | 3,805114 | 8,897494 |
| ENSMUSG00000083355 | Gm11581 | 5,77 | 0,003074384 | 10,54235 | 28,02235 | 16,4352 | 0 | 0 | 0,889749 |
| ENSMUSG00000083748 | Gm11662 | -3,73 | 6,47216E-05 | 15,81352 | 21,25833 | 10,38013 | 82,27669 | 46,92974 | 499,1494 |
| ENSMUSG00000085844 | Gm11690 | -2,45 | 0,002134211 | 11,59658 | 2,898863 | 6,920085 | 60,46841 | 24,09906 | 31,14123 |
| ENSMUSG00000087113 | Gm11714 | -1,60 | 6,18161E-10 | 70,63373 | 75,37045 | 68,33584 | 186,3617 | 246,064 | 216,2091 |
| ENSMUSG00000085636 | Gm11769 | 1,25 | 0,001106286 | 62,19985 | 79,2356 | 70,06586 | 28,74728 | 34,24603 | 26,69248 |
| ENSMUSG00000085501 | Gm11772 | 1,29 | 0,027063932 | 31,62704 | 81,16818 | 66,60582 | 23,79085 | 26,6358 | 23,13348 |
| ENSMUSG00000086765 | Gm11827 | -1,36 | 0,007669603 | 25,30163 | 55,0784 | 42,38552 | 124,902 | 68,49205 | 121,8957 |
| ENSMUSG00000086587 | Gm11837 | -3,76 | 0,001681548 | 5,271174 | 0 | 0,865011 | 25,77342 | 41,85625 | 13,34624 |
| ENSMUSG00000086127 | Gm11934 | -2,70 | 0,038401279 | 0 | 1,932576 | 2,595032 | 9,912855 | 8,878599 | 11,56674 |
| ENSMUSG00000086839 | Gm11973 | 1,20 | 0,015669176 | 71,68796 | 94,6962 | 57,0907 | 44,60785 | 27,90417 | 24,91298 |
| ENSMUSG00000081302 | Gm12020 | -3,84 | 0,018438784 | 1,054235 | 2,898863 | 0,865011 | 1,982571 | 8,878599 | 57,83371 |
| ENSMUSG00000048852 | Gm12185 | 1,76 | 0,042344436 | 366,8737 | 136,2466 | 75,25592 | 28,74728 | 36,78277 | 104,1007 |
| ENSMUSG00000082292 | Gm12250 | 1,31 | 0,043908292 | 2939,206 | 1103,501 | 1166,034 | 458,9652 | 487,0546 | 1157,564 |
| ENSMUSG00000058287 | Gm12253 | -3,17 | 0,000708523 | 2,108469 | 4,831439 | 3,460042 | 18,83442 | 17,7572 | 56,94396 |
| ENSMUSG00000080814 | Gm12314 | -1,46 | 0,002636364 | 22,13893 | 24,1572 | 14,70518 | 59,47713 | 65,95531 | 42,70797 |
| ENSMUSG00000085980 | Gm12408 | 9,27 | 0,004211464 | 16,86776 | 32,85379 | 20,76025 | 0 | 0 | 0 |
| ENSMUSG00000086211 | Gm12462 | -3,58 | 0,046041862 | 1,054235 | 0,966288 | 0 | 10,90414 | 8,878599 | 3,558997 |
| ENSMUSG00000087357 | Gm12498 | 1,77 | 0,035477989 | 23,19316 | 32,85379 | 42,38552 | 12,88671 | 13,95208 | 2,669248 |
| ENSMUSG00000087326 | Gm12503 | -2,00 | 3,56287E-05 | 24,2474 | 21,25833 | 22,49028 | 102,1024 | 52,00322 | 115,6674 |
| ENSMUSG00000084274 | Gm12504 | -1,61 | 0,000699372 | 47,44056 | 20,29204 | 28,54535 | 108,0501 | 76,10228 | 108,5494 |
| ENSMUSG00000086952 | Gm12596 | -2,25 | 0,000498201 | 6,325408 | 6,764015 | 4,325053 | 27,75599 | 27,90417 | 26,69248 |
| ENSMUSG00000085931 | Gm12648 | 6,93 | 1,03089E-20 | 185,5453 | 223,2125 | 341,6792 | 0 | 2,536743 | 3,558997 |
| ENSMUSG00000085181 | Gm12709 | 8,09 | 1,75944E-06 | 61,14562 | 143,9769 | 77,85095 | 0 | 1,268371 | 0 |
| ENSMUSG00000083679 | Gm12892 | 2,44 | 0,026548598 | 13,70505 | 22,22462 | 17,30021 | 0,991285 | 1,268371 | 7,117995 |
| ENSMUSG00000087195 | Gm12907 | 3,31 | 0,002883886 | 17,92199 | 36,71894 | 14,70518 | 4,956427 | 0 | 1,779499 |
| ENSMUSG00000085823 | Gm13012 | 1,04 | 0,001438345 | 392,1753 | 440,6272 | 468,8357 | 266,6558 | 220,6966 | 147,6984 |
| ENSMUSG00000087698 | Gm13031 | 1,77 | 0,047608681 | 12,65082 | 23,19091 | 13,84017 | 4,956427 | 6,341857 | 3,558997 |
| ENSMUSG00000084807 | Gm13073 | -2,22 | 0,006642997 | 11,59658 | 36,71894 | 6,055074 | 77,32027 | 124,3004 | 52,49521 |
| ENSMUSG00000086560 | Gm13372 | -4,25 | 1,80121E-09 | 6,325408 | 1,932576 | 5,190064 | 79,30284 | 130,6422 | 46,26697 |
| ENSMUSG00000087196 | Gm13373 | 3,14 | 5,08831E-13 | 117,0201 | 212,5833 | 153,1069 | 23,79085 | 12,68371 | 17,79499 |
| ENSMUSG00000087187 | Gm13431 | -1,46 | 0,00686441 | 86,44725 | 236,7405 | 103,8013 | 332,0806 | 306,9459 | 538,2984 |
| ENSMUSG00000082357 | Gm13542 | -3,75 | 0,000325033 | 5,271174 | 0,966288 | 0 | 26,76471 | 22,83068 | 30,25148 |
| ENSMUSG00000087422 | Gm13564 | 8,77 | 0,007727837 | 16,86776 | 21,25833 | 12,11015 | 0 | 0 | 0 |
| ENSMUSG00000087283 | Gm13580 | -2,90 | 0,024437967 | 2,108469 | 0,966288 | 0,865011 | 8,921569 | 10,14697 | 9,787243 |
| ENSMUSG00000087362 | Gm13710 | -2,91 | 5,40041E-05 | 21,08469 | 5,797727 | 32,8704 | 103,0937 | 100,2013 | 247,3503 |
| ENSMUSG00000085852 | Gm13807 | -1,56 | 0,004748286 | 17,92199 | 23,19091 | 20,76025 | 82,27669 | 34,24603 | 64,9517 |
| ENSMUSG00000085261 | Gm13814 | 3,16 | 0,001914258 | 30,57281 | 98,56136 | 30,27537 | 11,89543 | 5,073485 | 0,889749 |
| ENSMUSG00000084992 | Gm13842 | -3,08 | 0,001993613 | 10,54235 | 0,966288 | 8,650106 | 29,73856 | 30,44091 | 109,4392 |
| ENSMUSG00000084756 | Gm13881 | -1,75 | 1,32253E-05 | 45,33209 | 60,87613 | 34,60042 | 174,4662 | 111,6167 | 186,8474 |
| ENSMUSG00000085417 | Gm13919 | -1,87 | 1,01273E-06 | 47,44056 | 25,12348 | 34,60042 | 152,658 | 126,8371 | 110,3289 |
| ENSMUSG00000087203 | Gm13986 | -2,10 | 0,000676729 | 12,65082 | 5,797727 | 11,24514 | 55,51199 | 29,17254 | 41,81822 |
| ENSMUSG00000087450 | Gm13994 | -2,68 | 0,048612818 | 4,216939 | 0 | 6,920085 | 10,90414 | 12,68371 | 48,04647 |
| ENSMUSG00000079071 | Gm14085 | -2,77 | 2,00074E-07 | 25,30163 | 14,49432 | 17,30021 | 170,5011 | 150,9362 | 66,7312 |
| ENSMUSG00000081662 | Gm14098 | -3,86 | 0,00066793 | 3,162704 | 0,966288 | 0 | 16,85185 | 20,29394 | 19,57449 |
| ENSMUSG00000085483 | Gm14198 | -4,62 | 2,1518E-13 | 11,59658 | 0,966288 | 3,460042 | 134,8148 | 138,2525 | 110,3289 |
| ENSMUSG00000085546 | Gm14252 | 5,36 | 0,008450779 | 10,54235 | 22,22462 | 8,650106 | 0 | 0 | 0,889749 |
| ENSMUSG00000078862 | Gm14326 | 1,07 | 0,002045711 | 191,8707 | 230,9428 | 218,8477 | 93,18083 | 74,83391 | 135,2419 |
| ENSMUSG00000087442 | Gm14453 | -1,65 | 0,001455727 | 110,6946 | 85,03333 | 36,33044 | 296,3944 | 253,6743 | 177,9499 |
| ENSMUSG00000079694 | Gm14862 | -4,03 | 0,003218281 | 3,162704 | 0 | 0 | 18,83442 | 15,22046 | 14,23599 |
| ENSMUSG00000086736 | Gm14902 | 7,67 | 0,047452996 | 3,162704 | 19,32576 | 0,865011 | 0 | 0 | 0 |
| ENSMUSG00000087340 | Gm15228 | -2,84 | 0,025657226 | 2,108469 | 1,932576 | 0,865011 | 14,86928 | 5,073485 | 14,23599 |
| ENSMUSG00000085634 | Gm15290 | -3,77 | 0,000802406 | 4,216939 | 0 | 0,865011 | 22,79957 | 29,17254 | 15,12574 |
| ENSMUSG00000087597 | Gm15345 | -2,55 | 0,045893468 | 3,162704 | 0 | 2,595032 | 11,89543 | 13,95208 | 8,007744 |
| ENSMUSG00000079146 | Gm15353 | -4,21 | 0,034961274 | 2,108469 | 0 | 0 | 3,965142 | 5,073485 | 27,58223 |
| ENSMUSG00000086610 | Gm15408 | -1,70 | 0,024977933 | 6,325408 | 9,662878 | 27,68034 | 57,49456 | 48,19811 | 38,25922 |
| ENSMUSG00000051107 | Gm15440 | -1,40 | 2,53911E-05 | 60,09138 | 61,84242 | 38,92548 | 129,8584 | 138,2525 | 154,8164 |
| ENSMUSG00000084964 | Gm15503 | 1,88 | 0,006367332 | 23,19316 | 42,51666 | 44,11554 | 9,912855 | 5,073485 | 14,23599 |
| ENSMUSG00000087066 | Gm15518 | -1,23 | 0,04053369 | 18,97623 | 19,32576 | 8,650106 | 35,68628 | 38,05114 | 35,58997 |
| ENSMUSG00000090185 | Gm15523 | -2,02 | 0,017819906 | 14,75929 | 4,831439 | 6,055074 | 21,80828 | 24,09906 | 56,94396 |
| ENSMUSG00000081809 | Gm15539 | 2,81 | 0,01684396 | 6,325408 | 23,19091 | 13,84017 | 1,982571 | 2,536743 | 1,779499 |
| ENSMUSG00000083183 | Gm15575 | 4,09 | 0,00010123 | 24,2474 | 49,28068 | 15,57019 | 1,982571 | 2,536743 | 0,889749 |
| ENSMUSG00000085264 | Gm15581 | 8,26 | 0,014245346 | 11,59658 | 9,662878 | 13,84017 | 0 | 0 | 0 |
| ENSMUSG00000085913 | Gm15601 | 1,10 | 0,000226679 | 193,9792 | 278,2909 | 217,1177 | 116,9717 | 114,1534 | 90,75444 |
| ENSMUSG00000087691 | Gm15674 | -2,62 | 1,98327E-06 | 50,60327 | 27,05606 | 21,62526 | 287,4728 | 105,2748 | 213,5398 |
| ENSMUSG00000086825 | Gm15675 | -2,48 | 5,2926E-07 | 186,5995 | 149,7746 | 83,04102 | 1081,492 | 402,0737 | 847,9311 |
| ENSMUSG00000089776 | Gm15684 | -1,58 | 0,0279704 | 21,08469 | 20,29204 | 21,62526 | 78,31155 | 88,78599 | 22,24373 |
| ENSMUSG00000083962 | Gm15707 | -1,25 | 0,048497949 | 14,75929 | 24,1572 | 16,4352 | 37,66885 | 29,17254 | 64,06195 |
| ENSMUSG00000085118 | Gm15774 | -1,21 | 0,002415681 | 108,5862 | 96,62878 | 61,41575 | 264,6732 | 183,9138 | 166,3831 |
| ENSMUSG00000086741 | Gm15816 | -3,76 | 5,8466E-10 | 13,70505 | 3,865151 | 7,785095 | 159,597 | 111,6167 | 68,5107 |
| ENSMUSG00000086706 | Gm15848 | -2,47 | 0,012628591 | 5,271174 | 3,865151 | 3,460042 | 41,63399 | 17,7572 | 9,787243 |
| ENSMUSG00000087480 | Gm15910 | -1,25 | 0,016143014 | 31,62704 | 25,12348 | 23,35529 | 88,22441 | 41,85625 | 59,61321 |
| ENSMUSG00000085881 | Gm15912 | -3,39 | 0,000349522 | 7,379643 | 0,966288 | 2,595032 | 24,78214 | 24,09906 | 63,17221 |
| ENSMUSG00000085723 | Gm15915 | -3,82 | 9,34899E-11 | 43,22362 | 16,42689 | 19,03023 | 221,0567 | 256,211 | 630,8323 |
| ENSMUSG00000085337 | Gm15964 | 1,42 | 0,027642074 | 108,5862 | 163,3026 | 167,8121 | 100,1198 | 31,70928 | 32,03098 |
| ENSMUSG00000085786 | Gm15987 | 1,18 | 0,000226679 | 517,6293 | 722,7833 | 514,6813 | 332,0806 | 232,112 | 211,7603 |
| ENSMUSG00000087281 | Gm16015 | -1,83 | 0,045682365 | 5,271174 | 5,797727 | 2,595032 | 21,80828 | 12,68371 | 13,34624 |
| ENSMUSG00000085526 | Gm16083 | -3,16 | 0,010954817 | 2,108469 | 2,898863 | 0 | 11,89543 | 10,14697 | 21,35398 |
| ENSMUSG00000089714 | Gm16092 | -3,74 | 0,039554811 | 0 | 1,932576 | 0 | 7,930284 | 12,68371 | 5,338496 |
| ENSMUSG00000090008 | Gm16111 | -5,40 | 0,01167205 | 1,054235 | 0 | 0 | 23,79085 | 13,95208 | 3,558997 |
| ENSMUSG00000089910 | Gm16113 | -3,64 | 0,04195925 | 3,162704 | 0 | 0 | 18,83442 | 15,22046 | 3,558997 |
| ENSMUSG00000086245 | Gm16170 | -1,95 | 0,008798153 | 17,92199 | 18,35947 | 6,920085 | 69,38998 | 24,09906 | 71,17995 |
| ENSMUSG00000089886 | Gm16184 | 2,02 | 0,018004354 | 34,78975 | 33,82007 | 22,49028 | 7,930284 | 1,268371 | 12,45649 |
| ENSMUSG00000089988 | Gm16238 | 2,12 | 4,04246E-05 | 37,95245 | 71,5053 | 64,01078 | 13,878 | 12,68371 | 13,34624 |
| ENSMUSG00000084862 | Gm16278 | -1,33 | 0,00706412 | 24,2474 | 26,08977 | 28,54535 | 66,41613 | 90,05436 | 43,59772 |
| ENSMUSG00000087129 | Gm16316 | 2,21 | 1,3886E-05 | 56,92868 | 123,6848 | 115,9114 | 27,75599 | 17,7572 | 18,68474 |
| ENSMUSG00000087538 | Gm16341 | -1,81 | 0,018370085 | 11,59658 | 5,797727 | 4,325053 | 29,73856 | 27,90417 | 17,79499 |
| ENSMUSG00000087014 | Gm16364 | -1,48 | 0,030380826 | 18,97623 | 13,52803 | 6,055074 | 38,66013 | 27,90417 | 39,14897 |
| ENSMUSG00000086915 | Gm16365 | -1,75 | 0,041840456 | 14,75929 | 4,831439 | 6,055074 | 23,79085 | 17,7572 | 43,59772 |
| ENSMUSG00000057160 | Gm16372 | 1,57 | 0,000198605 | 142,3217 | 91,79734 | 163,487 | 32,71242 | 46,92974 | 54,27471 |
| ENSMUSG00000086593 | Gm16548 | -1,93 | 0,000992701 | 13,70505 | 5,797727 | 10,38013 | 31,72113 | 41,85625 | 40,03872 |
| ENSMUSG00000089956 | Gm16553 | -2,71 | 0,007841622 | 1,054235 | 4,831439 | 1,730021 | 18,83442 | 11,41534 | 19,57449 |
| ENSMUSG00000089829 | Gm16565 | -1,14 | 0,020531515 | 24,2474 | 20,29204 | 14,70518 | 41,63399 | 41,85625 | 46,26697 |
| ENSMUSG00000097633 | Gm16617 | -3,75 | 0,00477566 | 1,054235 | 0,966288 | 0,865011 | 10,90414 | 8,878599 | 18,68474 |
| ENSMUSG00000097465 | Gm16619 | -1,39 | 0,01333114 | 9,488113 | 20,29204 | 17,30021 | 36,67756 | 38,05114 | 48,93622 |
| ENSMUSG00000097823 | Gm16701 | 2,31 | 0,00894932 | 22,13893 | 66,67386 | 27,68034 | 7,930284 | 2,536743 | 12,45649 |
| ENSMUSG00000070858 | Gm1673 | 1,51 | 0,024806098 | 52,71174 | 159,4375 | 87,36607 | 30,72985 | 21,56231 | 52,49521 |
| ENSMUSG00000097357 | Gm16793 | -3,99 | 3,80145E-18 | 46,38633 | 22,22462 | 19,89524 | 666,1438 | 367,8277 | 362,128 |
| ENSMUSG00000097749 | Gm16938 | -1,35 | 0,000818579 | 21,08469 | 37,68522 | 33,73541 | 72,36384 | 82,44414 | 81,85694 |
| ENSMUSG00000091184 | Gm17059 | -3,60 | 0,009799853 | 0 | 1,932576 | 0,865011 | 14,86928 | 8,878599 | 10,67699 |
| ENSMUSG00000091172 | Gm17120 | -3,18 | 0,003713263 | 6,325408 | 0 | 2,595032 | 34,69499 | 31,70928 | 13,34624 |
| ENSMUSG00000057359 | Gm17494 | -1,73 | 0,009292906 | 8,433878 | 6,764015 | 8,650106 | 18,83442 | 29,17254 | 31,14123 |
| ENSMUSG00000099241 | Gm18852 | 1,73 | 0,022672158 | 47,44056 | 26,08977 | 22,49028 | 8,921569 | 5,073485 | 14,23599 |
| ENSMUSG00000097187 | Gm19426 | -3,49 | 0,000677595 | 6,325408 | 1,932576 | 0 | 27,75599 | 21,56231 | 40,03872 |
| ENSMUSG00000073902 | Gm1966 | 1,38 | 2,42927E-05 | 7720,161 | 7380,506 | 10400,89 | 2429,641 | 4382,223 | 3002,014 |
| ENSMUSG00000075286 | Gm1968 | -3,97 | 0,000122511 | 21,08469 | 4,831439 | 7,785095 | 53,52942 | 48,19811 | 421,7412 |
| ENSMUSG00000097113 | Gm19705 | -1,10 | 0,005794133 | 49,54903 | 95,66249 | 64,87579 | 132,8323 | 142,0576 | 176,1704 |
| ENSMUSG00000092418 | Gm20406 | -3,32 | 0,0048713 | 1,054235 | 1,932576 | 0,865011 | 11,89543 | 13,95208 | 12,45649 |
| ENSMUSG00000092222 | Gm20506 | -2,00 | 0,00022115 | 12,65082 | 9,662878 | 12,97516 | 52,53813 | 31,70928 | 56,05421 |
| ENSMUSG00000092569 | Gm20544 | 1,06 | 0,041603468 | 81,17608 | 87,93219 | 98,61121 | 63,44227 | 40,58788 | 24,91298 |
| ENSMUSG00000096887 | Gm20594 | -1,08 | 4,66468E-05 | 1839,64 | 2091,047 | 1534,529 | 4615,425 | 3102,436 | 3793,891 |
| ENSMUSG00000097615 | Gm2061 | -3,25 | 0,031152194 | 2,108469 | 0 | 0,865011 | 7,930284 | 12,68371 | 7,117995 |
| ENSMUSG00000093553 | Gm20633 | -1,08 | 0,042399598 | 22,13893 | 34,78636 | 28,54535 | 41,63399 | 58,34508 | 80,07744 |
| ENSMUSG00000093800 | Gm20796 | 9,46 | 0,006126932 | 28,46434 | 1,932576 | 50,17061 | 0 | 0 | 0 |
| ENSMUSG00000094027 | Gm21762 | -1,90 | 0,032416139 | 21,08469 | 16,42689 | 4,325053 | 42,62527 | 25,36743 | 86,30569 |
| ENSMUSG00000096299 | Gm21814 | 1,51 | 0,009657316 | 32,68128 | 57,97727 | 59,68573 | 22,79957 | 19,02557 | 11,56674 |
| ENSMUSG00000084469 | Gm22956 | 3,46 | 3,71319E-06 | 36,89822 | 131,4151 | 46,71057 | 6,938998 | 6,341857 | 6,228246 |
| ENSMUSG00000088976 | Gm23759 | 2,99 | 0,026598292 | 12,65082 | 42,51666 | 24,2203 | 8,921569 | 0 | 0,889749 |
| ENSMUSG00000088384 | Gm24690 | 3,99 | 0,015751138 | 5,271174 | 15,4606 | 11,24514 | 0,991285 | 0 | 0,889749 |
| ENSMUSG00000089307 | Gm24843 | 12,26 | 6,21846E-05 | 94,88113 | 324,6727 | 143,5918 | 0 | 0 | 0 |
| ENSMUSG00000088371 | Gm25180 | -1,73 | 0,040772921 | 5,271174 | 35,75265 | 14,70518 | 70,38127 | 39,31951 | 74,73895 |
| ENSMUSG00000097739 | Gm2539 | 7,78 | 0,025470279 | 9,488113 | 9,662878 | 6,055074 | 0 | 0 | 0 |
| ENSMUSG00000064443 | Gm26226 | 2,29 | 0,010725978 | 16,86776 | 84,06704 | 34,60042 | 8,921569 | 5,073485 | 13,34624 |
| ENSMUSG00000097423 | Gm26522 | 2,08 | 0,000402192 | 61,14562 | 33,82007 | 42,38552 | 11,89543 | 13,95208 | 7,117995 |
| ENSMUSG00000096945 | Gm26546 | 1,97 | 0,016025224 | 25,30163 | 55,0784 | 18,16522 | 10,90414 | 5,073485 | 8,897494 |
| ENSMUSG00000097926 | Gm26575 | -2,60 | 0,036472412 | 2,108469 | 0,966288 | 1,730021 | 11,89543 | 10,14697 | 7,117995 |
| ENSMUSG00000097730 | Gm26588 | -1,93 | 0,00060225 | 9,488113 | 14,49432 | 14,70518 | 47,5817 | 67,22368 | 33,81048 |
| ENSMUSG00000097139 | Gm26626 | 1,46 | 0,01106332 | 33,73551 | 61,84242 | 34,60042 | 16,85185 | 16,48883 | 14,23599 |
| ENSMUSG00000096931 | Gm26656 | 1,12 | 0,017536478 | 152,864 | 93,72992 | 111,5864 | 74,34641 | 54,53997 | 36,47972 |
| ENSMUSG00000097090 | Gm26724 | 1,72 | 0,000295939 | 255,1248 | 485,0765 | 237,0129 | 141,7538 | 79,90739 | 74,73895 |
| ENSMUSG00000097467 | Gm26737 | -1,08 | 0,04506685 | 22,13893 | 19,32576 | 22,49028 | 57,49456 | 46,92974 | 31,14123 |
| ENSMUSG00000097840 | Gm26756 | -1,25 | 0,04614982 | 14,75929 | 22,22462 | 13,84017 | 45,59913 | 50,73485 | 24,91298 |
| ENSMUSG00000097026 | Gm26786 | 1,48 | 1,32253E-05 | 152,864 | 198,089 | 146,1868 | 70,38127 | 43,12463 | 63,17221 |
| ENSMUSG00000097892 | Gm26801 | 1,58 | 0,00029044 | 221,3893 | 260,8977 | 157,4319 | 95,1634 | 76,10228 | 43,59772 |
| ENSMUSG00000097879 | Gm26869 | 1,80 | 3,86043E-05 | 93,82689 | 137,2129 | 87,36607 | 41,63399 | 22,83068 | 26,69248 |
| ENSMUSG00000097832 | Gm26912 | 1,61 | 0,009203181 | 28,46434 | 61,84242 | 47,57558 | 12,88671 | 11,41534 | 20,46424 |
| ENSMUSG00000097970 | Gm27028 | -2,37 | 0,018398578 | 3,162704 | 2,898863 | 1,730021 | 16,85185 | 11,41534 | 11,56674 |
| ENSMUSG00000089838 | Gm2962 | -1,54 | 0,029394759 | 17,92199 | 8,69659 | 11,24514 | 51,54684 | 36,78277 | 21,35398 |
| ENSMUSG00000094392 | Gm3788 | 1,76 | 0,004783392 | 35,84398 | 28,02235 | 38,92548 | 14,86928 | 6,341857 | 8,897494 |
| ENSMUSG00000078606 | Gm4070 | 1,76 | 7,70306E-06 | 640,9747 | 370,0882 | 328,704 | 100,1198 | 136,9841 | 158,3754 |
| ENSMUSG00000089940 | Gm4117 | 1,60 | 0,003628227 | 35,84398 | 72,47159 | 45,84556 | 15,86057 | 15,22046 | 19,57449 |
| ENSMUSG00000078780 | Gm5150 | -1,37 | 3,42677E-05 | 220,3351 | 362,3579 | 256,0431 | 880,2615 | 560,6201 | 719,8072 |
| ENSMUSG00000072553 | Gm525 | 4,01 | 0,000902736 | 26,35587 | 18,35947 | 389,2548 | 7,930284 | 6,341857 | 12,45649 |
| ENSMUSG00000046687 | Gm5424 | 2,27 | 1,12268E-13 | 203,4673 | 148,8083 | 199,8174 | 37,66885 | 41,85625 | 35,58997 |
| ENSMUSG00000058163 | Gm5431 | 2,51 | 2,93105E-13 | 538,714 | 972,0855 | 664,3281 | 155,6318 | 95,12785 | 129,0137 |
| ENSMUSG00000079697 | Gm5751 | 2,53 | 0,000238965 | 33,73551 | 38,65151 | 25,95032 | 5,947713 | 2,536743 | 8,007744 |
| ENSMUSG00000062588 | Gm6104 | -1,50 | 0,018441524 | 11,59658 | 7,730302 | 8,650106 | 21,80828 | 30,44091 | 26,69248 |
| ENSMUSG00000089716 | Gm6264 | -2,15 | 0,002741639 | 8,433878 | 5,797727 | 4,325053 | 18,83442 | 35,5144 | 27,58223 |
| ENSMUSG00000078370 | Gm6316 | 2,34 | 0,019596479 | 14,75929 | 19,32576 | 16,4352 | 0,991285 | 2,536743 | 6,228246 |
| ENSMUSG00000097252 | Gm6634 | -4,50 | 1,11301E-06 | 15,81352 | 5,797727 | 12,97516 | 69,38998 | 91,32274 | 622,8246 |
| ENSMUSG00000090186 | Gm7592 | -1,31 | 0,00269807 | 24,2474 | 20,29204 | 21,62526 | 44,60785 | 62,1502 | 56,94396 |
| ENSMUSG00000063628 | Gm7665 | -1,39 | 0,006518759 | 22,13893 | 43,48295 | 21,62526 | 85,25055 | 86,24925 | 57,83371 |
| ENSMUSG00000050157 | Gm867 | -3,36 | 7,05047E-12 | 234,0401 | 62,80871 | 96,88119 | 1331,296 | 1330,522 | 1381,781 |
| ENSMUSG00000097426 | Gm8941 | -3,42 | 0,0047258 | 5,271174 | 0 | 0,865011 | 19,82571 | 30,44091 | 13,34624 |
| ENSMUSG00000095649 | Gm8979 | 1,67 | 0,012365843 | 41,11515 | 36,71894 | 21,62526 | 11,89543 | 6,341857 | 12,45649 |
| ENSMUSG00000063286 | Gm8995 | 1,11 | 2,26047E-09 | 24152,52 | 18989,49 | 19698,89 | 9501,471 | 10409,52 | 9192,89 |
| ENSMUSG00000098021 | Gm9522 | 5,53 | 0,000502815 | 2,108469 | 77,30302 | 70,06586 | 0 | 2,536743 | 0,889749 |
| ENSMUSG00000047361 | Gm973 | -3,20 | 0,011267012 | 3,162704 | 0,966288 | 0,865011 | 6,938998 | 12,68371 | 24,91298 |
| ENSMUSG00000044434 | Gm9791 | -2,56 | 0,00571206 | 2,108469 | 2,898863 | 3,460042 | 20,81699 | 11,41534 | 17,79499 |
| ENSMUSG00000053749 | Gm9920 | 4,03 | 6,15263E-18 | 142,3217 | 194,2238 | 203,2775 | 15,86057 | 3,805114 | 12,45649 |
| ENSMUSG00000068600 | Gml2 | -3,42 | 0,019547758 | 2,108469 | 0,966288 | 0 | 6,938998 | 10,14697 | 14,23599 |
| ENSMUSG00000057614 | Gnai1 | -2,04 | 0,044268556 | 10,54235 | 4,831439 | 20,76025 | 23,79085 | 24,09906 | 100,5417 |
| ENSMUSG00000040009 | Gnaz | -4,23 | 0,000507404 | 165,5149 | 56,04469 | 89,09609 | 552,146 | 583,4508 | 4677,412 |
| ENSMUSG00000027669 | Gnb4 | -1,29 | 0,000256149 | 380,5787 | 305,3469 | 328,704 | 693,8998 | 613,8917 | 1174,469 |
| ENSMUSG00000038811 | Gngt2 | -1,66 | 0,000224164 | 274,101 | 215,4822 | 355,5194 | 749,4118 | 551,7415 | 1370,214 |
| ENSMUSG00000035311 | Gnptab | 1,26 | 5,30112E-13 | 6347,547 | 7261,653 | 6883,754 | 2685,392 | 2649,628 | 3226,231 |
| ENSMUSG00000021556 | Golm1 | -2,66 | 4,52151E-07 | 781,1879 | 180,6958 | 374,5496 | 2612,037 | 2789,149 | 3066,076 |
| ENSMUSG00000050675 | Gp1ba | -3,56 | 1,84296E-05 | 520,792 | 329,5041 | 333,0291 | 1275,784 | 1506,825 | 11207,28 |
| ENSMUSG00000050761 | Gp1bb | -3,78 | 0,007428266 | 237,2028 | 189,3924 | 252,5831 | 510,512 | 684,9205 | 8159,002 |
| ENSMUSG00000047953 | Gp5 | -3,78 | 0,005061357 | 584,046 | 353,6613 | 435,1003 | 1295,61 | 1628,589 | 15986,13 |
| ENSMUSG00000078810 | Gp6 | -2,50 | 0,005802867 | 300,4569 | 171,0329 | 211,9276 | 289,4554 | 481,9811 | 3098,107 |
| ENSMUSG00000030054 | Gp9 | -3,72 | 7,77114E-06 | 396,3923 | 304,3807 | 416,0701 | 1192,516 | 1665,372 | 11817,65 |
| ENSMUSG00000029314 | Gpat3 | -1,23 | 0,000869179 | 387,9584 | 329,5041 | 301,8887 | 978,3988 | 925,9111 | 490,2519 |
| ENSMUSG00000055653 | Gpc3 | 4,75 | 1,53771E-24 | 339,4636 | 725,6821 | 543,2266 | 10,90414 | 20,29394 | 28,47198 |
| ENSMUSG00000031119 | Gpc4 | -3,75 | 1,78662E-18 | 25,30163 | 15,4606 | 23,35529 | 265,6645 | 403,3421 | 192,1859 |
| ENSMUSG00000027346 | Gpcpd1 | -1,73 | 3,45265E-15 | 3001,406 | 3538,546 | 2549,186 | 10834,75 | 10771,01 | 8646,584 |
| ENSMUSG00000023019 | Gpd1 | -2,14 | 0,000487885 | 72,7422 | 71,5053 | 132,3466 | 300,3595 | 190,2557 | 726,9252 |
| ENSMUSG00000022579 | Gpihbp1 | -1,60 | 0,017484731 | 20,03046 | 24,1572 | 30,27537 | 34,69499 | 65,95531 | 124,5649 |
| ENSMUSG00000031517 | Gpm6a | -1,65 | 0,027111138 | 75,9049 | 19,32576 | 59,68573 | 84,25926 | 133,179 | 269,5941 |
| ENSMUSG00000029816 | Gpnmb | 3,20 | 1,1499E-06 | 15944,25 | 20460,18 | 21061,28 | 1505,763 | 4166,6 | 585,4551 |
| ENSMUSG00000021306 | Gpr137b | -1,77 | 1,55732E-07 | 175,003 | 107,2579 | 170,4071 | 385,61 | 532,716 | 621,0451 |
| ENSMUSG00000049092 | Gpr137c | -1,75 | 0,000290964 | 62,19985 | 63,77499 | 31,14038 | 187,353 | 227,0385 | 112,1084 |
| ENSMUSG00000041762 | Gpr155 | -1,67 | 1,62279E-10 | 329,9755 | 257,9988 | 237,0129 | 1059,684 | 749,6075 | 820,3489 |
| ENSMUSG00000038390 | Gpr162 | -1,45 | 0,008364944 | 55,87444 | 65,70757 | 52,76565 | 113,0065 | 96,39622 | 266,0351 |
| ENSMUSG00000073008 | Gpr174 | -1,66 | 3,54051E-05 | 210,8469 | 124,6511 | 174,7321 | 480,7735 | 370,3644 | 755,3972 |
| ENSMUSG00000040133 | Gpr176 | -2,20 | 0,007629506 | 7,379643 | 11,59545 | 17,30021 | 21,80828 | 44,393 | 101,4314 |
| ENSMUSG00000058396 | Gpr182 | -1,08 | 0,023694881 | 94,88113 | 66,67386 | 107,2613 | 174,4662 | 121,7636 | 272,2633 |
| ENSMUSG00000051212 | Gpr183 | 1,56 | 3,52169E-08 | 1307,251 | 2319,091 | 1942,814 | 645,3268 | 602,4764 | 637,0605 |
| ENSMUSG00000072875 | Gpr27 | -1,69 | 0,012648354 | 24,2474 | 88,89848 | 61,41575 | 200,2397 | 93,85948 | 269,5941 |
| ENSMUSG00000026271 | Gpr35 | 1,39 | 0,000132323 | 3747,805 | 2634,101 | 3515,403 | 1011,111 | 1809,966 | 952,0318 |
| ENSMUSG00000044317 | Gpr4 | -1,71 | 0,000727801 | 13,70505 | 22,22462 | 17,30021 | 42,62527 | 55,80834 | 75,6287 |
| ENSMUSG00000056380 | Gpr50 | -4,25 | 5,3866E-05 | 3,162704 | 3,865151 | 2,595032 | 11,89543 | 43,12463 | 127,2342 |
| ENSMUSG00000047415 | Gpr68 | 1,02 | 0,03914936 | 392,1753 | 293,7515 | 519,8714 | 176,4488 | 123,032 | 293,6173 |
| ENSMUSG00000063234 | Gpr84 | -1,50 | 0,000606939 | 157,081 | 297,6166 | 208,4676 | 849,5316 | 636,7224 | 391,4897 |
| ENSMUSG00000048216 | Gpr85 | 1,72 | 0,009002535 | 32,68128 | 78,26931 | 41,52051 | 9,912855 | 21,56231 | 15,12574 |
| ENSMUSG00000068696 | Gpr88 | -3,48 | 0,000218369 | 4,216939 | 5,797727 | 7,785095 | 56,50327 | 13,95208 | 129,0137 |
| ENSMUSG00000051043 | Gprc5c | -1,27 | 0,006661927 | 43,22362 | 34,78636 | 38,06047 | 56,50327 | 111,6167 | 111,2187 |
| ENSMUSG00000045441 | Gprin3 | 1,04 | 0,04774677 | 114,9116 | 129,4826 | 211,0626 | 81,28541 | 43,12463 | 96,09293 |
| ENSMUSG00000027883 | Gpsm2 | -1,17 | 4,69356E-05 | 1833,314 | 1314,151 | 1778,462 | 3884,848 | 4392,37 | 2817,836 |
| ENSMUSG00000018339 | Gpx3 | 2,00 | 2,59796E-06 | 11298,23 | 8804,814 | 11368,83 | 1407,625 | 2479,666 | 3985,187 |
| ENSMUSG00000021760 | Gpx8 | -1,93 | 0,01660003 | 29,51857 | 47,3481 | 56,22569 | 69,38998 | 83,71251 | 352,3407 |
| ENSMUSG00000036292 | Gramd1c | -2,37 | 0,001224927 | 150,7556 | 97,59507 | 111,5864 | 249,8039 | 281,5784 | 1331,065 |
| ENSMUSG00000074259 | Gramd2 | -1,58 | 1,40607E-05 | 48,4948 | 56,04469 | 29,41036 | 129,8584 | 129,3739 | 139,6907 |
| ENSMUSG00000042351 | Grap2 | -2,81 | 3,63842E-10 | 886,6114 | 312,111 | 606,3724 | 4017,68 | 3060,58 | 5565,382 |
| ENSMUSG00000020176 | Grb10 | -1,73 | 2,1812E-06 | 142,3217 | 163,3026 | 211,9276 | 467,8867 | 442,6616 | 809,6719 |
| ENSMUSG00000019312 | Grb7 | -1,47 | 0,004211464 | 33,73551 | 22,22462 | 19,89524 | 97,14598 | 53,2716 | 58,72346 |
| ENSMUSG00000036523 | Greb1 | 5,68 | 2,58753E-13 | 100,1523 | 451,2564 | 448,0755 | 3,965142 | 2,536743 | 12,45649 |
| ENSMUSG00000001986 | Gria3 | 3,81 | 2,98919E-77 | 16534,62 | 16693,59 | 16513,92 | 1208,377 | 1393,94 | 941,3548 |
| ENSMUSG00000022564 | Grina | -1,47 | 5,30235E-09 | 11158,02 | 11244,69 | 8673,461 | 29508,59 | 33911,18 | 22570,27 |
| ENSMUSG00000063239 | Grm4 | 3,84 | 0,037490151 | 2,108469 | 12,56174 | 15,57019 | 0,991285 | 1,268371 | 0 |
| ENSMUSG00000000617 | Grm6 | -2,84 | 0,001900546 | 16,86776 | 3,865151 | 1,730021 | 75,3377 | 44,393 | 38,25922 |
| ENSMUSG00000024211 | Grm8 | -2,75 | 0,002756069 | 12,65082 | 0,966288 | 8,650106 | 28,74728 | 40,58788 | 80,07744 |
| ENSMUSG00000031364 | Grpr | 6,11 | 2,18806E-06 | 47,44056 | 60,87613 | 31,14038 | 0 | 0 | 1,779499 |
| ENSMUSG00000050105 | Grrp1 | -1,60 | 0,008145524 | 13,70505 | 10,62917 | 13,84017 | 29,73856 | 30,44091 | 55,16446 |
| ENSMUSG00000038515 | Grtp1 | -3,10 | 2,86218E-05 | 24,2474 | 21,25833 | 23,35529 | 92,18955 | 81,17577 | 416,4027 |
| ENSMUSG00000026879 | Gsn | -1,28 | 4,78409E-09 | 8030,106 | 8912,072 | 8436,448 | 17517,01 | 19106,75 | 25180,8 |
| ENSMUSG00000071723 | Gspt2 | 1,27 | 1,70912E-05 | 413,26 | 350,7625 | 271,6133 | 135,8061 | 124,3004 | 167,2729 |
| ENSMUSG00000027610 | Gss | 1,58 | 1,58136E-08 | 1238,726 | 1781,835 | 1727,426 | 451,0349 | 476,9076 | 657,5248 |
| ENSMUSG00000057933 | Gsta2 | 4,58 | 5,60677E-09 | 187,6538 | 158,4712 | 388,3898 | 2,973856 | 3,805114 | 23,13348 |
| ENSMUSG00000032348 | Gsta4 | -1,20 | 0,03761483 | 26,35587 | 16,42689 | 22,49028 | 44,60785 | 34,24603 | 70,2902 |
| ENSMUSG00000058135 | Gstm1 | 1,23 | 0,002019969 | 8965,212 | 6636,465 | 8498,729 | 3791,667 | 4503,987 | 1964,567 |
| ENSMUSG00000027890 | Gstm4 | 1,10 | 8,66638E-06 | 790,6761 | 798,1537 | 1089,048 | 411,3835 | 464,2239 | 379,0332 |
| ENSMUSG00000025068 | Gsto1 | 1,07 | 3,69637E-06 | 3592,832 | 3901,87 | 4493,73 | 1570,196 | 1927,924 | 2214,586 |
| ENSMUSG00000058216 | Gstp3 | -1,52 | 1,73471E-06 | 103,315 | 70,53901 | 80,44598 | 200,2397 | 228,3068 | 297,1763 |
| ENSMUSG00000001663 | Gstt1 | 1,15 | 0,013201941 | 689,4695 | 812,648 | 1610,65 | 356,8628 | 494,6648 | 552,5344 |
| ENSMUSG00000023982 | Guca1a | 1,12 | 0,001009253 | 210,8469 | 234,8079 | 177,3272 | 90,20698 | 72,29717 | 122,7854 |
| ENSMUSG00000033416 | Gucd1 | -1,04 | 5,84182E-09 | 2861,193 | 3099,851 | 2800,904 | 6714,968 | 6093,256 | 5269,986 |
| ENSMUSG00000033910 | Gucy1a3 | -3,54 | 6,53961E-05 | 151,8098 | 63,77499 | 128,8866 | 308,2898 | 475,6393 | 3212,885 |
| ENSMUSG00000028005 | Gucy1b3 | -4,18 | 0,002374304 | 180,2741 | 24,1572 | 62,28076 | 386,6013 | 688,7256 | 3744,065 |
| ENSMUSG00000055523 | Gucy2g | -3,16 | 0,004824014 | 5,271174 | 0,966288 | 1,730021 | 12,88671 | 13,95208 | 42,70797 |
| ENSMUSG00000025534 | Gusb | 1,06 | 2,80366E-06 | 11110,58 | 9974,989 | 11115,39 | 4991,122 | 6202,336 | 4227,199 |
| ENSMUSG00000045868 | Gvin1 | 1,69 | 9,72541E-05 | 262,5045 | 151,7072 | 278,5334 | 46,59042 | 90,05436 | 79,18769 |
| ENSMUSG00000051839 | Gypa | -3,43 | 6,81064E-09 | 3966,031 | 689,9295 | 1696,286 | 23624,32 | 25438,46 | 19391,2 |
| ENSMUSG00000090523 | Gypc | -1,61 | 1,23139E-09 | 3205,928 | 1884,261 | 2819,069 | 8192,974 | 8357,299 | 7657,183 |
| ENSMUSG00000019188 | H13 | 1,02 | 6,19328E-09 | 13404,59 | 11053,37 | 11883,52 | 6121,188 | 6372,298 | 5419,463 |
| ENSMUSG00000000031 | H19 | 9,17 | 2,6398E-49 | 3535,903 | 1237,815 | 2801,769 | 1,982571 | 3,805114 | 7,117995 |
| ENSMUSG00000020086 | H2afy2 | 2,06 | 1,94892E-05 | 113,8574 | 231,9091 | 224,0377 | 33,70371 | 36,78277 | 65,84145 |
| ENSMUSG00000037548 | H2-DMb2 | -1,11 | 0,002386736 | 1096,404 | 767,2325 | 502,5711 | 1763,497 | 1589,269 | 1768,822 |
| ENSMUSG00000067341 | H2-Eb2 | -2,58 | 1,21641E-05 | 84,33878 | 19,32576 | 43,25053 | 227,9957 | 239,7222 | 408,395 |
| ENSMUSG00000016206 | H2-M3 | 1,08 | 0,022209876 | 3279,724 | 1703,565 | 1634,87 | 1023,998 | 715,3614 | 1399,576 |
| ENSMUSG00000024334 | H2-Oa | -1,65 | 0,000893521 | 208,7385 | 92,76363 | 110,7214 | 407,4183 | 276,505 | 607,6988 |
| ENSMUSG00000041538 | H2-Ob | -1,77 | 1,30566E-08 | 1075,319 | 650,3117 | 946,3216 | 2538,682 | 2701,631 | 3854,394 |
| ENSMUSG00000000673 | Haao | 1,14 | 4,5851E-08 | 1953,497 | 2145,159 | 2354,559 | 1051,754 | 815,5628 | 1057,912 |
| ENSMUSG00000038822 | Hace1 | -1,11 | 8,12838E-08 | 1443,247 | 1456,196 | 1113,269 | 3139,401 | 2891,887 | 2647,894 |
| ENSMUSG00000027984 | Hadh | 1,18 | 2,9131E-05 | 3398,853 | 3274,749 | 4233,362 | 1281,732 | 1486,531 | 2052,652 |
| ENSMUSG00000024158 | Hagh | -2,14 | 8,24673E-20 | 1722,62 | 1160,512 | 1521,554 | 7061,918 | 6801,007 | 5596,524 |
| ENSMUSG00000006930 | Hap1 | -1,99 | 0,023110258 | 14,75929 | 33,82007 | 27,68034 | 47,5817 | 41,85625 | 212,6501 |
| ENSMUSG00000022367 | Has2 | -3,08 | 2,94179E-05 | 14,75929 | 6,764015 | 25,08531 | 173,475 | 176,3036 | 46,26697 |
| ENSMUSG00000040405 | Havcr1 | -1,53 | 0,013919268 | 104,3692 | 143,0106 | 69,20085 | 189,3355 | 183,9138 | 541,8574 |
| ENSMUSG00000069919 | Hba-a1 | -4,24 | 8,80263E-21 | 23902,66 | 7049,07 | 13730,31 | 305213,8 | 223096,4 | 313718,5 |
| ENSMUSG00000069917 | Hba-a2 | -4,21 | 4,95839E-15 | 7276,328 | 1685,206 | 3140,853 | 73725,87 | 55516,61 | 94889,99 |
| ENSMUSG00000055609 | Hba-x | -2,80 | 0,036442902 | 1,054235 | 0,966288 | 1,730021 | 6,938998 | 8,878599 | 10,67699 |
| ENSMUSG00000052305 | Hbb-bs/Hbb-bs/Hbb-bs | -4,19 | 8,85047E-20 | 101749,5 | 28530,61 | 54991,32 | 1225205 | 965236,9 | 1179486 |
| ENSMUSG00000073940 | Hbb-bt/Hbb-bt/Hbb-bt | -4,14 | 3,57981E-16 | 23302,8 | 5239,212 | 13337,6 | 251734 | 266152,5 | 221191,7 |
| ENSMUSG00000052187 | Hbb-y | -3,36 | 0,021625483 | 0 | 0,966288 | 1,730021 | 6,938998 | 11,41534 | 10,67699 |
| ENSMUSG00000020295 | Hbq1a | -4,40 | 3,03041E-18 | 9,488113 | 2,898863 | 5,190064 | 132,8323 | 102,7381 | 128,1239 |
| ENSMUSG00000073063 | Hbq1b | -4,05 | 2,35286E-22 | 105,4235 | 33,82007 | 52,76565 | 1054,728 | 1043,87 | 1081,045 |
| ENSMUSG00000026874 | Hc | -2,51 | 0,000107697 | 4,216939 | 6,764015 | 6,920085 | 39,65142 | 24,09906 | 38,25922 |
| ENSMUSG00000042770 | Hebp1 | -1,89 | 6,07326E-24 | 1431,651 | 1453,297 | 1131,434 | 4678,867 | 4826,153 | 5334,937 |
| ENSMUSG00000042744 | Hectd4 | -1,04 | 0,000724583 | 12152,16 | 7199,81 | 9022,925 | 21416,72 | 20867,25 | 15876,69 |
| ENSMUSG00000042807 | Hecw2 | -2,18 | 1,92813E-14 | 35,84398 | 30,92121 | 31,14038 | 151,6667 | 150,9362 | 140,5804 |
| ENSMUSG00000028332 | Hemgn | -3,32 | 6,0955E-06 | 3706,689 | 427,0992 | 1028,498 | 17515,02 | 21261,71 | 12760,79 |
| ENSMUSG00000022528 | Hes1 | -1,69 | 0,000380168 | 67,47102 | 76,33674 | 79,58097 | 241,8737 | 124,3004 | 353,2305 |
| ENSMUSG00000048001 | Hes5 | -1,86 | 0,011159731 | 11,59658 | 6,764015 | 7,785095 | 48,57299 | 24,09906 | 21,35398 |
| ENSMUSG00000023781 | Hes7 | 3,54 | 8,60834E-08 | 90,66419 | 110,1568 | 102,0712 | 6,938998 | 1,268371 | 16,90524 |
| ENSMUSG00000040726 | Hesx1 | 5,06 | 8,38363E-13 | 80,12184 | 114,022 | 173,8671 | 0,991285 | 2,536743 | 7,117995 |
| ENSMUSG00000025232 | Hexa | 1,26 | 1,1348E-09 | 27287,81 | 20113,28 | 25997,89 | 9597,626 | 9791,827 | 11163,69 |
| ENSMUSG00000064325 | Hhip | -4,30 | 0,02158418 | 1,054235 | 0 | 0,865011 | 4,956427 | 3,805114 | 28,47198 |
| ENSMUSG00000004328 | Hif3a | -2,76 | 2,817E-09 | 26,35587 | 15,4606 | 14,70518 | 88,22441 | 123,032 | 169,0524 |
| ENSMUSG00000039959 | Hip1 | 1,29 | 4,13131E-12 | 7030,691 | 6894,463 | 5597,483 | 2620,959 | 2895,692 | 2495,747 |
| ENSMUSG00000000915 | Hip1r | -1,18 | 2,67697E-05 | 2932,881 | 2527,809 | 2205,777 | 6872,582 | 6154,138 | 4296,6 |
| ENSMUSG00000036181 | Hist1h1c | -1,67 | 0,005222048 | 1075,319 | 1877,497 | 2121,006 | 2533,726 | 4052,446 | 9504,303 |
| ENSMUSG00000051627 | Hist1h1e | -1,13 | 0,003329373 | 26,35587 | 30,92121 | 38,92548 | 69,38998 | 73,56554 | 68,5107 |
| ENSMUSG00000069270 | Hist1h2ac | -2,37 | 0,000162042 | 17,92199 | 23,19091 | 21,62526 | 62,45098 | 67,22368 | 193,0756 |
| ENSMUSG00000018102 | Hist1h2bc | -1,65 | 1,06453E-06 | 421,6939 | 487,0091 | 513,8163 | 1052,745 | 1312,764 | 2106,037 |
| ENSMUSG00000047246 | Hist1h2be | -1,11 | 0,000479243 | 69,57949 | 100,4939 | 64,01078 | 170,5011 | 157,278 | 177,9499 |
| ENSMUSG00000095217 | Hist1h2bn | -1,42 | 0,049786774 | 28,46434 | 15,4606 | 13,84017 | 31,72113 | 38,05114 | 83,63644 |
| ENSMUSG00000069308 | Hist1h2bp | -1,90 | 0,012736574 | 11,59658 | 5,797727 | 3,460042 | 20,81699 | 26,6358 | 29,36173 |
| ENSMUSG00000056895 | Hist3h2ba | -2,02 | 1,47484E-13 | 54,82021 | 45,41553 | 64,01078 | 238,8998 | 197,8659 | 229,5553 |
| ENSMUSG00000015501 | Hivep2 | -1,10 | 0,008452041 | 931,9435 | 427,0992 | 664,3281 | 1821,983 | 1371,109 | 1137,1 |
| ENSMUSG00000028634 | Hivep3 | 1,11 | 2,05487E-07 | 2015,697 | 1598,24 | 1520,689 | 841,6014 | 714,0931 | 820,3489 |
| ENSMUSG00000003949 | Hlf | -1,46 | 0,010103286 | 137,0505 | 267,6617 | 185,1123 | 388,5839 | 315,8245 | 920,0008 |
| ENSMUSG00000032126 | Hmbs | -2,56 | 2,7222E-12 | 6290,619 | 2583,854 | 3676,295 | 25942,93 | 24686,31 | 23489,38 |
| ENSMUSG00000055632 | Hmcn2 | -1,25 | 0,023338665 | 11,59658 | 12,56174 | 16,4352 | 30,72985 | 34,24603 | 32,03098 |
| ENSMUSG00000056758 | Hmga2 | -2,10 | 0,021950977 | 10,54235 | 17,39318 | 8,650106 | 25,77342 | 22,83068 | 107,6597 |
| ENSMUSG00000015217 | Hmgb3 | -1,06 | 6,79794E-09 | 1638,281 | 1289,028 | 1525,879 | 2885,632 | 3187,417 | 3235,129 |
| ENSMUSG00000027875 | Hmgcs2 | -2,53 | 0,002400739 | 8,433878 | 3,865151 | 19,03023 | 48,57299 | 31,70928 | 101,4314 |
| ENSMUSG00000005413 | Hmox1 | -1,51 | 0,003171113 | 7268,949 | 3505,692 | 5953,868 | 10044,7 | 11975,96 | 25475,3 |
| ENSMUSG00000017950 | Hnf4a | 2,01 | 0,012802871 | 224,552 | 270,5606 | 458,4556 | 34,69499 | 30,44091 | 170,8319 |
| ENSMUSG00000007617 | Homer1 | 2,33 | 1,29824E-14 | 2403,655 | 3245,761 | 3061,272 | 595,7626 | 736,9237 | 400,3872 |
| ENSMUSG00000028572 | Hook1 | -1,06 | 1,01058E-05 | 319,4331 | 217,4148 | 269,0183 | 579,902 | 535,2527 | 558,7626 |
| ENSMUSG00000059325 | Hopx | 1,25 | 0,000417452 | 619,89 | 557,5481 | 982,652 | 273,5948 | 375,4379 | 259,8068 |
| ENSMUSG00000000938 | Hoxa10 | -1,59 | 0,016547124 | 16,86776 | 21,25833 | 12,97516 | 29,73856 | 41,85625 | 80,96719 |
| ENSMUSG00000038236 | Hoxa7 | -1,68 | 0,020621627 | 34,78975 | 40,58409 | 46,71057 | 76,32898 | 59,61345 | 254,4683 |
| ENSMUSG00000038227 | Hoxa9 | -1,70 | 0,005046736 | 64,30832 | 79,2356 | 42,38552 | 130,8497 | 119,2269 | 351,451 |
| ENSMUSG00000085645 | Hoxb5os | 4,07 | 0,009572577 | 7,379643 | 13,52803 | 12,97516 | 0,991285 | 0 | 0,889749 |
| ENSMUSG00000079277 | Hoxd3 | 3,88 | 0,000771382 | 8,433878 | 95,66249 | 29,41036 | 1,982571 | 2,536743 | 4,448747 |
| ENSMUSG00000029445 | Hpd | 3,18 | 0,003552332 | 10,54235 | 46,38181 | 19,89524 | 0,991285 | 5,073485 | 2,669248 |
| ENSMUSG00000031613 | Hpgd | 2,83 | 0,000204176 | 5938,504 | 12655,47 | 4759,288 | 641,3617 | 336,1184 | 2300,892 |
| ENSMUSG00000029919 | Hpgds | -2,59 | 3,51301E-07 | 98,04383 | 76,33674 | 149,6468 | 446,0785 | 416,0258 | 1085,494 |
| ENSMUSG00000001249 | Hpn | -2,84 | 4,55055E-12 | 126,5082 | 45,41553 | 67,47083 | 604,6841 | 579,6457 | 523,1726 |
| ENSMUSG00000035273 | Hpse | 1,88 | 1,05406E-07 | 9618,838 | 8540,052 | 9622,378 | 1963,737 | 1835,333 | 3770,758 |
| ENSMUSG00000071001 | Hrct1 | -1,88 | 0,039862709 | 9,488113 | 10,62917 | 6,055074 | 16,85185 | 17,7572 | 60,50296 |
| ENSMUSG00000037346 | Hrh4 | -2,75 | 0,009419127 | 9,488113 | 0,966288 | 2,595032 | 35,68628 | 12,68371 | 37,36947 |
| ENSMUSG00000051022 | Hs3st1 | -2,44 | 4,17195E-07 | 67,47102 | 38,65151 | 44,11554 | 168,5185 | 223,2334 | 420,8515 |
| ENSMUSG00000045216 | Hs6st1 | -1,63 | 2,33669E-10 | 2185,429 | 1310,286 | 1872,748 | 5820,828 | 5560,54 | 5260,198 |
| ENSMUSG00000062184 | Hs6st2 | -3,60 | 0,043164095 | 1,054235 | 0,966288 | 0,865011 | 1,982571 | 5,073485 | 27,58223 |
| ENSMUSG00000019878 | Hsf2 | 1,15 | 1,40759E-05 | 607,2392 | 713,1204 | 794,0797 | 392,549 | 286,6519 | 276,7121 |
| ENSMUSG00000062007 | Hsh2d | -1,17 | 0,000120081 | 279,3722 | 469,6159 | 301,8887 | 874,3138 | 734,387 | 758,0665 |
| ENSMUSG00000081189 | Hspd1-ps4 | -6,19 | 0,000246592 | 1,054235 | 0 | 0,865011 | 9,912855 | 11,41534 | 118,3367 |
| ENSMUSG00000028763 | Hspg2 | -1,07 | 0,019612195 | 181,3284 | 150,7409 | 265,5582 | 309,2811 | 336,1184 | 611,2578 |
| ENSMUSG00000064267 | Hvcn1 | -1,21 | 0,002496558 | 5366,055 | 3421,625 | 4181,461 | 10762,39 | 13211,36 | 6084,106 |
| ENSMUSG00000010051 | Hyal1 | -1,11 | 0,001170176 | 66,41679 | 57,01098 | 41,52051 | 108,0501 | 124,3004 | 122,7854 |
| ENSMUSG00000036091 | Hyal3 | -1,39 | 0,016343571 | 71,68796 | 34,78636 | 31,14038 | 112,0153 | 171,2301 | 78,29794 |
| ENSMUSG00000049287 | Iba57 | -1,52 | 1,70908E-05 | 1176,526 | 625,1882 | 866,7406 | 3018,464 | 2732,072 | 1885,379 |
| ENSMUSG00000029306 | Ibsp | -2,20 | 0,003223756 | 377,416 | 81,16818 | 526,7914 | 753,377 | 1570,244 | 2199,46 |
| ENSMUSG00000032174 | Icam5 | -2,17 | 7,33318E-05 | 12,65082 | 10,62917 | 8,650106 | 61,4597 | 34,24603 | 46,26697 |
| ENSMUSG00000000732 | Icosl | 1,03 | 7,15272E-10 | 4841,046 | 4863,327 | 4498,92 | 2484,161 | 2052,225 | 2437,913 |
| ENSMUSG00000007872 | Id3 | -1,82 | 1,93063E-05 | 206,63 | 119,8197 | 257,7732 | 673,0828 | 471,8341 | 915,5521 |
| ENSMUSG00000025950 | Idh1 | 1,30 | 1,8548E-09 | 6575,262 | 6922,486 | 8111,204 | 3338,649 | 2440,346 | 3011,802 |
| ENSMUSG00000073489 | Ifi204 | 1,37 | 9,23035E-08 | 1962,985 | 2762,617 | 2281,033 | 829,7059 | 781,3167 | 1093,502 |
| ENSMUSG00000073490 | Ifi207 | 1,52 | 5,81072E-11 | 3085,745 | 3671,894 | 4078,525 | 1222,255 | 1490,336 | 1066,809 |
| ENSMUSG00000043263 | Ifi209 | 2,15 | 1,05233E-12 | 14010,78 | 11926,89 | 14252,78 | 3976,046 | 3047,896 | 2057,99 |
| ENSMUSG00000026536 | Ifi211 | 1,79 | 3,62426E-13 | 537,6597 | 596,1996 | 432,5053 | 151,6667 | 169,9618 | 131,6829 |
| ENSMUSG00000073491 | Ifi213 | 1,56 | 0,002592341 | 985,7095 | 2127,766 | 1102,888 | 485,7299 | 262,5529 | 679,7685 |
| ENSMUSG00000067297 | Ifit1bl2 | -2,74 | 1,04957E-10 | 106,4777 | 54,11212 | 84,77104 | 710,7517 | 325,9714 | 605,0296 |
| ENSMUSG00000025491 | Ifitm1 | -1,64 | 0,000604333 | 731,6389 | 644,514 | 591,6672 | 1417,538 | 1251,883 | 3441,551 |
| ENSMUSG00000059108 | Ifitm6 | -1,45 | 2,93265E-12 | 6009,138 | 7462,641 | 7241,869 | 15821,91 | 20112,56 | 20542,53 |
| ENSMUSG00000062157 | Ifnlr1 | -2,03 | 4,66404E-09 | 575,6122 | 746,9405 | 453,2655 | 3154,27 | 2406,1 | 1672,729 |
| ENSMUSG00000010048 | Ifrd2 | -1,80 | 3,5021E-13 | 2337,238 | 1622,397 | 1645,25 | 7034,162 | 7006,483 | 5533,351 |
| ENSMUSG00000024169 | Ift140 | -1,73 | 1,30951E-08 | 2527,001 | 1910,351 | 2216,157 | 5539,303 | 9945,3 | 6601,94 |
| ENSMUSG00000020053 | Igf1 | -2,50 | 1,1856E-05 | 180,2741 | 113,0557 | 274,2084 | 626,4924 | 701,4093 | 1873,812 |
| ENSMUSG00000048583 | Igf2 | 1,97 | 0,002082568 | 100,1523 | 168,1341 | 376,2796 | 33,70371 | 62,1502 | 69,40045 |
| ENSMUSG00000023830 | Igf2r | -1,08 | 0,009807693 | 2229,706 | 2446,641 | 1730,886 | 4947,506 | 5996,86 | 2590,95 |
| ENSMUSG00000026185 | Igfbp5 | -1,28 | 8,2845E-06 | 1046,855 | 1096,737 | 1627,085 | 2656,645 | 2910,912 | 3602,595 |
| ENSMUSG00000036256 | Igfbp7 | -1,57 | 0,009147668 | 295,1857 | 207,7519 | 369,3595 | 411,3835 | 634,1857 | 1544,605 |
| ENSMUSG00000051985 | Igfn1 | -2,95 | 0,034999602 | 23,19316 | 5,797727 | 2,595032 | 183,3878 | 5,073485 | 53,38496 |
| ENSMUSG00000076615 | Ighg3 | -2,50 | 4,2859E-08 | 62,19985 | 94,6962 | 121,9665 | 648,3007 | 645,601 | 283,83 |
| ENSMUSG00000096108 | Ighv11-2 | -3,95 | 0,005024796 | 125,4539 | 53,14583 | 6,920085 | 1890,381 | 687,4573 | 290,0583 |
| ENSMUSG00000076676 | Ighv12-3 | -2,52 | 0,022665899 | 11,59658 | 2,898863 | 5,190064 | 68,3987 | 35,5144 | 8,007744 |
| ENSMUSG00000095923 | Ighv1-37 | 2,21 | 0,010749453 | 22,13893 | 21,25833 | 75,25592 | 5,947713 | 10,14697 | 9,787243 |
| ENSMUSG00000095519 | Ighv1-66 | -1,69 | 0,015091117 | 157,081 | 73,43787 | 148,7818 | 133,8235 | 668,4317 | 424,4104 |
| ENSMUSG00000094029 | Ighv3-3 | -3,51 | 0,02547808 | 9,488113 | 0,966288 | 6,920085 | 17,84314 | 177,572 | 2,669248 |
| ENSMUSG00000076655 | Ighv4-1 | -2,30 | 0,001200238 | 183,4368 | 269,5943 | 106,3963 | 970,4685 | 1541,071 | 250,9093 |
| ENSMUSG00000076633 | Ighv5-2 | -1,72 | 0,024124123 | 24,2474 | 12,56174 | 27,68034 | 91,19826 | 97,66459 | 24,91298 |
| ENSMUSG00000076677 | Ighv6-3 | -1,12 | 0,044362155 | 433,2905 | 211,617 | 402,2299 | 457,9739 | 1189,732 | 621,9348 |
| ENSMUSG00000087582 | Ighv6-7 | -3,15 | 2,38388E-06 | 70,63373 | 82,13446 | 83,90603 | 355,8715 | 1463,701 | 281,1608 |
| ENSMUSG00000094505 | Ighv8-6 | -2,12 | 0,023667289 | 3,162704 | 7,730302 | 5,190064 | 16,85185 | 12,68371 | 40,03872 |
| ENSMUSG00000076606 | Igkj3 | -1,66 | 0,008712889 | 67,47102 | 25,12348 | 30,27537 | 185,3704 | 128,1055 | 72,95945 |
| ENSMUSG00000076607 | Igkj4 | -1,51 | 0,017908496 | 104,3692 | 36,71894 | 70,06586 | 272,6035 | 232,112 | 96,09293 |
| ENSMUSG00000076608 | Igkj5 | -1,72 | 0,005557448 | 127,5624 | 41,55038 | 63,14577 | 321,1765 | 315,8245 | 129,9034 |
| ENSMUSG00000094335 | Igkv1-117 | -1,16 | 0,027419236 | 3467,378 | 1060,018 | 2585,517 | 5070,425 | 6423,032 | 4438,96 |
| ENSMUSG00000094491 | Igkv1-133 | -2,99 | 0,000607386 | 131,7793 | 33,82007 | 22,49028 | 132,8323 | 738,1921 | 620,1553 |
| ENSMUSG00000094345 | Igkv14-126 | -3,27 | 2,94966E-06 | 352,1144 | 81,16818 | 211,0626 | 3537,898 | 1941,877 | 742,051 |
| ENSMUSG00000076518 | Igkv2-112 | -3,28 | 4,18163E-07 | 15,81352 | 61,84242 | 19,03023 | 371,732 | 405,8788 | 161,9344 |
| ENSMUSG00000096715 | Igkv3-4 | -2,16 | 0,022952202 | 363,711 | 32,85379 | 175,5971 | 287,4728 | 1378,72 | 896,8674 |
| ENSMUSG00000076552 | Igkv4-61 | -2,05 | 0,01830365 | 55,87444 | 29,95492 | 173,8671 | 125,8933 | 575,8406 | 375,4742 |
| ENSMUSG00000076550 | Igkv4-63 | -3,33 | 0,000479459 | 134,942 | 39,6178 | 19,89524 | 276,5686 | 1443,407 | 226,8861 |
| ENSMUSG00000076547 | Igkv4-70 | -2,53 | 0,023186643 | 45,33209 | 40,58409 | 127,1566 | 45,59913 | 996,9399 | 193,9654 |
| ENSMUSG00000094315 | Igkv4-78 | -3,14 | 0,003452783 | 5,271174 | 0 | 3,460042 | 14,86928 | 40,58788 | 21,35398 |
| ENSMUSG00000076539 | Igkv4-81 | -2,97 | 0,002253192 | 4,216939 | 1,932576 | 2,595032 | 30,72985 | 27,90417 | 9,787243 |
| ENSMUSG00000076533 | Igkv4-90 | -1,33 | 0,004816756 | 152,864 | 78,26931 | 73,5259 | 176,4488 | 314,5561 | 275,8223 |
| ENSMUSG00000076575 | Igkv7-33 | -3,62 | 0,015880975 | 3,162704 | 2,898863 | 0,865011 | 8,921569 | 72,29717 | 3,558997 |
| ENSMUSG00000076937 | Iglc2 | -1,35 | 0,001025 | 1363,126 | 604,8962 | 869,3356 | 2942,135 | 2414,979 | 1849,789 |
| ENSMUSG00000075370 | Igll1 | -1,74 | 0,005764227 | 525,0089 | 129,4826 | 404,825 | 1665,36 | 1199,879 | 684,2173 |
| ENSMUSG00000076939 | Iglv3 | -1,35 | 6,86886E-09 | 225,6062 | 163,3026 | 179,9222 | 440,1307 | 517,4955 | 488,4724 |
| ENSMUSG00000042035 | Igsf3 | -2,63 | 2,37065E-17 | 88,55572 | 55,0784 | 99,47622 | 419,3138 | 532,716 | 553,4241 |
| ENSMUSG00000018168 | Ikzf3 | -1,55 | 0,002741639 | 1746,867 | 642,5814 | 1021,578 | 4720,501 | 3107,51 | 2141,627 |
| ENSMUSG00000032089 | Il10ra | 1,03 | 9,46368E-05 | 8225,139 | 10185,64 | 10329,09 | 4663,007 | 3690,961 | 5714,86 |
| ENSMUSG00000017057 | Il13ra1 | 1,18 | 1,9286E-06 | 2104,253 | 2579,022 | 2650,392 | 875,3051 | 1074,311 | 1291,916 |
| ENSMUSG00000023206 | Il15ra | 1,48 | 2,53539E-07 | 1530,749 | 1197,231 | 967,9468 | 382,6362 | 533,9843 | 411,0642 |
| ENSMUSG00000040717 | Il17rd | 1,43 | 0,015416908 | 56,92868 | 44,44924 | 38,92548 | 8,921569 | 22,83068 | 20,46424 |
| ENSMUSG00000039217 | Il18 | -1,40 | 0,011999822 | 103,315 | 222,2462 | 164,352 | 302,3421 | 285,3836 | 702,0123 |
| ENSMUSG00000027398 | Il1b | -1,27 | 0,016374936 | 3914,374 | 1670,712 | 1849,393 | 3598,366 | 6231,508 | 8047,783 |
| ENSMUSG00000026073 | Il1r2 | -2,28 | 0,001482141 | 1539,183 | 478,3125 | 814,84 | 3691,547 | 8489,209 | 1597,1 |
| ENSMUSG00000026069 | Il1rl1 | -2,50 | 0,000115006 | 629,3781 | 143,9769 | 154,8369 | 1783,323 | 2163,842 | 1297,255 |
| ENSMUSG00000020007 | Il20ra | -3,09 | 0,002826395 | 5,271174 | 0,966288 | 4,325053 | 11,89543 | 24,09906 | 53,38496 |
| ENSMUSG00000030745 | Il21r | -1,33 | 0,001500864 | 1261,919 | 613,5928 | 904,8011 | 2044,031 | 1746,547 | 3195,09 |
| ENSMUSG00000005465 | Il27ra | -1,34 | 0,001138601 | 351,0602 | 256,0663 | 322,6489 | 690,926 | 502,275 | 1158,454 |
| ENSMUSG00000031750 | Il34 | -2,36 | 0,000494069 | 73,79643 | 25,12348 | 58,82072 | 107,0588 | 251,1375 | 449,3234 |
| ENSMUSG00000068758 | Il3ra | 1,51 | 5,81184E-14 | 2414,198 | 3125,941 | 2744,679 | 1058,693 | 853,6139 | 990,291 |
| ENSMUSG00000027947 | Il6ra | 1,63 | 2,14022E-09 | 14185,78 | 11628,31 | 14828,01 | 5667,179 | 3968,734 | 3486,038 |
| ENSMUSG00000021756 | Il6st | 1,87 | 3,81397E-14 | 12507,44 | 16841,43 | 15671,4 | 3251,416 | 4190,699 | 4842,016 |
| ENSMUSG00000003882 | Il7r | -1,76 | 0,001149478 | 867,6352 | 328,5379 | 904,8011 | 2567,429 | 3188,686 | 1348,86 |
| ENSMUSG00000040612 | Ildr2 | -2,79 | 0,002345775 | 3,162704 | 3,865151 | 3,460042 | 10,90414 | 38,05114 | 24,02323 |
| ENSMUSG00000030890 | Ilk | -1,70 | 0,049786774 | 10,54235 | 7,730302 | 13,84017 | 20,81699 | 19,02557 | 64,06195 |
| ENSMUSG00000035270 | Impg2 | -1,58 | 0,003328308 | 41,11515 | 30,92121 | 14,70518 | 99,12855 | 92,59111 | 65,84145 |
| ENSMUSG00000074918 | Inafm2 | -1,68 | 0,002828651 | 1273,516 | 952,7598 | 1139,219 | 2161,002 | 1996,416 | 6656,215 |
| ENSMUSG00000041324 | Inhba | -1,94 | 2,08686E-07 | 263,5587 | 244,4708 | 196,3574 | 948,6602 | 1208,758 | 541,8574 |
| ENSMUSG00000032737 | Inppl1 | 1,63 | 0,001195331 | 4304,44 | 4389,845 | 2877,025 | 1648,508 | 540,3262 | 1548,164 |
| ENSMUSG00000024210 | Ip6k3 | 3,43 | 8,79238E-15 | 229,8232 | 216,4485 | 201,5475 | 15,86057 | 10,14697 | 32,92073 |
| ENSMUSG00000064065 | Ipcef1 | -1,08 | 2,64326E-05 | 1830,152 | 2322,956 | 1600,27 | 4768,083 | 3589,491 | 3811,686 |
| ENSMUSG00000029601 | Iqcd | -2,48 | 1,88344E-06 | 21,08469 | 10,62917 | 16,4352 | 126,8845 | 79,90739 | 61,39271 |
| ENSMUSG00000034312 | Iqsec1 | -1,04 | 7,5712E-05 | 4307,603 | 4560,878 | 3551,733 | 9873,203 | 9105,638 | 6546,776 |
| ENSMUSG00000041115 | Iqsec2 | 1,32 | 1,66847E-05 | 1158,604 | 1137,321 | 1063,098 | 410,3922 | 328,5082 | 605,9193 |
| ENSMUSG00000029771 | Irf5 | 1,82 | 7,26323E-13 | 12689,82 | 13468,12 | 16091,79 | 5081,329 | 3541,293 | 3372,15 |
| ENSMUSG00000041515 | Irf8 | 1,17 | 0,002110111 | 22537,43 | 36632,94 | 28243,46 | 12757,84 | 8641,414 | 17322,53 |
| ENSMUSG00000062028 | Irgc1 | -1,98 | 0,002502079 | 5,271174 | 9,662878 | 6,920085 | 35,68628 | 27,90417 | 23,13348 |
| ENSMUSG00000044792 | Isca1 | -2,49 | 3,29168E-19 | 1988,287 | 1088,04 | 1406,507 | 8765,937 | 8385,203 | 7997,067 |
| ENSMUSG00000035692 | Isg15 | -1,70 | 6,865E-10 | 1286,166 | 1056,153 | 857,2255 | 2708,192 | 3654,178 | 4055,478 |
| ENSMUSG00000039236 | Isg20 | -3,02 | 3,83162E-18 | 2026,239 | 899,6139 | 1162,574 | 10420,39 | 12130,7 | 10667,21 |
| ENSMUSG00000037206 | Islr | -1,59 | 0,01273731 | 170,786 | 139,1454 | 219,7127 | 235,9259 | 369,0961 | 984,9525 |
| ENSMUSG00000074766 | Ism1 | -1,47 | 0,02721005 | 15,81352 | 15,4606 | 23,35529 | 31,72113 | 38,05114 | 80,96719 |
| ENSMUSG00000043153 | Ispd | -1,27 | 0,004051216 | 28,46434 | 26,08977 | 45,84556 | 78,31155 | 68,49205 | 96,09293 |
| ENSMUSG00000001518 | Itfg2 | 1,18 | 0,000849889 | 1123,814 | 1033,928 | 1073,478 | 328,1155 | 678,5787 | 419,9617 |
| ENSMUSG00000042284 | Itga1 | 2,04 | 4,69582E-06 | 2676,702 | 3773,354 | 1989,524 | 843,5839 | 846,0037 | 363,0177 |
| ENSMUSG00000032243 | Itga11 | -1,31 | 0,032201279 | 20,03046 | 17,39318 | 25,95032 | 28,74728 | 71,02879 | 57,83371 |
| ENSMUSG00000015533 | Itga2 | -3,28 | 0,000109235 | 157,081 | 34,78636 | 45,84556 | 273,5948 | 423,636 | 1615,785 |
| ENSMUSG00000000555 | Itga5 | 1,13 | 4,25301E-08 | 4319,2 | 4695,192 | 4828,489 | 1723,845 | 2327,461 | 2272,42 |
| ENSMUSG00000039115 | Itga9 | -2,02 | 8,97198E-05 | 214,0097 | 93,72992 | 257,7732 | 556,1111 | 593,5978 | 1137,989 |
| ENSMUSG00000070369 | Itgad | -2,72 | 1,9932E-05 | 178,1657 | 272,4932 | 446,3455 | 1350,131 | 848,5404 | 3729,829 |
| ENSMUSG00000030789 | Itgax | -1,04 | 0,02440861 | 745,344 | 1009,771 | 884,0408 | 1399,695 | 1175,78 | 2839,19 |
| ENSMUSG00000000157 | Itgb2l | -1,39 | 2,41302E-05 | 6769,241 | 10887,16 | 6843,964 | 23996,05 | 24200,53 | 15982,57 |
| ENSMUSG00000020689 | Itgb3 | -1,97 | 0,01370747 | 7449,223 | 7550,573 | 7077,517 | 9305,197 | 11917,62 | 65562,07 |
| ENSMUSG00000025780 | Itih5 | -1,47 | 1,61203E-09 | 710,5542 | 711,1878 | 798,4048 | 1731,776 | 1845,48 | 2590,06 |
| ENSMUSG00000030102 | Itpr1 | 1,03 | 9,69441E-10 | 9692,634 | 8810,612 | 9097,316 | 4470,697 | 4034,689 | 5003,95 |
| ENSMUSG00000074825 | Itpripl1 | 1,03 | 0,000269046 | 3225,958 | 1945,137 | 2913,356 | 1381,852 | 1373,646 | 1190,485 |
| ENSMUSG00000022957 | Itsn1 | -1,54 | 3,7464E-15 | 2848,542 | 2199,271 | 2770,629 | 8318,868 | 7355,285 | 7001,438 |
| ENSMUSG00000027276 | Jag1 | -1,63 | 2,31073E-06 | 153,9183 | 95,66249 | 147,9168 | 421,2963 | 506,0802 | 305,184 |
| ENSMUSG00000063646 | Jakmip1 | -1,51 | 0,000145275 | 465,9718 | 203,8867 | 329,569 | 1153,856 | 894,2018 | 787,4282 |
| ENSMUSG00000048534 | Jaml | -1,61 | 0,004211464 | 1032,096 | 1852,374 | 680,7633 | 4488,541 | 4602,92 | 1815,089 |
| ENSMUSG00000038695 | Josd2 | -1,49 | 1,01733E-09 | 1423,217 | 1583,746 | 1205,825 | 3107,68 | 4241,434 | 4444,298 |
| ENSMUSG00000052684 | Jun | 1,23 | 8,38788E-07 | 1637,227 | 1150,849 | 1319,141 | 511,5033 | 678,5787 | 561,4319 |
| ENSMUSG00000061751 | Kalrn | -2,55 | 0,001976521 | 661,0052 | 409,706 | 590,8022 | 1017,059 | 1159,291 | 7520,162 |
| ENSMUSG00000041298 | Katnal1 | -1,06 | 1,37212E-06 | 367,9279 | 395,2117 | 387,5247 | 886,2092 | 865,0293 | 655,7453 |
| ENSMUSG00000033182 | Kbtbd12 | -3,09 | 1,43721E-09 | 11,59658 | 8,69659 | 6,055074 | 57,49456 | 71,02879 | 92,53393 |
| ENSMUSG00000040724 | Kcna2 | -1,77 | 0,026083158 | 26,35587 | 11,59545 | 40,6555 | 59,47713 | 48,19811 | 160,1549 |
| ENSMUSG00000058975 | Kcnc1 | -1,95 | 0,006980238 | 18,97623 | 19,32576 | 14,70518 | 43,61656 | 34,24603 | 126,3444 |
| ENSMUSG00000035355 | Kcnh4 | -2,13 | 0,001311163 | 12,65082 | 3,865151 | 6,920085 | 34,69499 | 38,05114 | 29,36173 |
| ENSMUSG00000025221 | Kcnip2 | 1,26 | 0,03784297 | 34,78975 | 71,5053 | 41,52051 | 15,86057 | 19,02557 | 26,69248 |
| ENSMUSG00000079056 | Kcnip3 | 1,02 | 0,003452783 | 807,5438 | 636,7837 | 851,1704 | 352,8976 | 509,8853 | 274,0428 |
| ENSMUSG00000044708 | Kcnj10 | -3,65 | 1,40759E-05 | 42,16939 | 28,02235 | 163,487 | 693,8998 | 341,1919 | 1911,182 |
| ENSMUSG00000051497 | Kcnj16 | -3,48 | 0,006309005 | 0 | 0,966288 | 6,055074 | 28,74728 | 10,14697 | 41,81822 |
| ENSMUSG00000032034 | Kcnj5 | -2,77 | 0,002348621 | 360,5483 | 143,9769 | 150,5118 | 368,7582 | 574,5722 | 3511,841 |
| ENSMUSG00000038026 | Kcnj9 | -3,07 | 0,003730784 | 2,108469 | 0,966288 | 10,38013 | 35,68628 | 17,7572 | 61,39271 |
| ENSMUSG00000045404 | Kcnk13 | -1,71 | 0,011842296 | 46,38633 | 11,59545 | 36,33044 | 63,44227 | 104,0064 | 139,6907 |
| ENSMUSG00000023243 | Kcnk5 | -1,32 | 0,005683608 | 177,1114 | 89,86477 | 227,4978 | 385,61 | 312,0193 | 540,9676 |
| ENSMUSG00000054342 | Kcnn4 | -2,66 | 4,27636E-12 | 1256,648 | 477,3462 | 738,719 | 5413,41 | 5120,415 | 5126,736 |
| ENSMUSG00000016346 | Kcnq2 | 3,96 | 0,000143229 | 14,75929 | 115,9545 | 38,92548 | 4,956427 | 1,268371 | 4,448747 |
| ENSMUSG00000056258 | Kcnq3 | 3,27 | 1,30502E-10 | 186,5995 | 402,942 | 583,8821 | 53,52942 | 29,17254 | 38,25922 |
| ENSMUSG00000041633 | Kctd12b | 1,57 | 0,000348822 | 645,1917 | 1189,5 | 988,7071 | 234,9347 | 242,2589 | 476,0159 |
| ENSMUSG00000051727 | Kctd14 | -1,34 | 5,89632E-10 | 211,9012 | 273,4594 | 275,0734 | 700,8388 | 640,5275 | 590,7936 |
| ENSMUSG00000033287 | Kctd17 | 1,56 | 0,000317655 | 455,4294 | 401,9757 | 505,1662 | 105,0763 | 112,885 | 243,7913 |
| ENSMUSG00000029866 | Kel | -3,29 | 4,2439E-09 | 2049,432 | 495,7056 | 666,9232 | 12509,03 | 10476,75 | 8373,431 |
| ENSMUSG00000022332 | Khdrbs3 | -2,90 | 2,3643E-09 | 35,84398 | 17,39318 | 32,8704 | 146,7102 | 173,7669 | 320,3098 |
| ENSMUSG00000028758 | Kif17 | 1,88 | 6,52215E-08 | 455,4294 | 929,5689 | 605,5074 | 161,5795 | 190,2557 | 190,4064 |
| ENSMUSG00000027115 | Kif18a | -1,06 | 6,09702E-05 | 986,7637 | 720,8507 | 869,3356 | 1773,41 | 2147,353 | 1462,748 |
| ENSMUSG00000014602 | Kif1a | -2,12 | 3,68799E-06 | 40,06092 | 17,39318 | 33,73541 | 100,1198 | 168,6934 | 127,2342 |
| ENSMUSG00000063077 | Kif1b | 1,15 | 2,11669E-09 | 9217,174 | 9083,105 | 9991,737 | 4211,972 | 4898,45 | 3657,76 |
| ENSMUSG00000041734 | Kirrel | -1,72 | 0,012073084 | 43,22362 | 34,78636 | 59,68573 | 61,4597 | 121,7636 | 269,5941 |
| ENSMUSG00000035773 | Kiss1r | -1,58 | 9,68658E-06 | 48,4948 | 75,37045 | 51,03562 | 198,2571 | 133,179 | 190,4064 |
| ENSMUSG00000019966 | Kitl | -1,95 | 0,001279682 | 193,9792 | 138,1792 | 265,5582 | 360,8279 | 549,2048 | 1398,686 |
| ENSMUSG00000054191 | Klf1 | -3,39 | 5,90029E-08 | 1348,366 | 240,6057 | 384,9297 | 7385,077 | 7036,924 | 6228,246 |
| ENSMUSG00000030087 | Klf15 | -1,91 | 0,011740817 | 12,65082 | 5,797727 | 15,57019 | 28,74728 | 29,17254 | 69,40045 |
| ENSMUSG00000029178 | Klf3 | -1,04 | 0,000521682 | 2829,566 | 2159,653 | 2623,577 | 5227,048 | 6554,943 | 3852,615 |
| ENSMUSG00000003032 | Klf4 | 2,04 | 6,75015E-05 | 2487,994 | 4581,17 | 4179,731 | 1409,608 | 894,2018 | 422,6309 |
| ENSMUSG00000025959 | Klf7 | -1,02 | 0,00017532 | 1013,12 | 1045,523 | 734,394 | 1537,484 | 2006,563 | 2124,721 |
| ENSMUSG00000041649 | Klf8 | 1,49 | 7,07421E-07 | 694,7407 | 666,7386 | 555,3368 | 214,1177 | 169,9618 | 297,1763 |
| ENSMUSG00000042115 | Klhdc8a | 5,64 | 1,21904E-05 | 28,46434 | 29,95492 | 43,25053 | 0,991285 | 0 | 0,889749 |
| ENSMUSG00000032609 | Klhdc8b | -1,92 | 2,469E-08 | 48,4948 | 77,30302 | 47,57558 | 230,9695 | 175,0352 | 248,2401 |
| ENSMUSG00000026455 | Klhl12 | -1,46 | 1,36442E-07 | 1498,068 | 985,6136 | 1087,318 | 3932,429 | 3118,925 | 2777,798 |
| ENSMUSG00000042514 | Klhl14 | -1,86 | 0,045022262 | 139,159 | 24,1572 | 94,28615 | 554,1286 | 295,5305 | 86,30569 |
| ENSMUSG00000042155 | Klhl23 | -1,29 | 2,25773E-18 | 460,7006 | 455,1216 | 433,3703 | 1068,606 | 1145,339 | 1084,604 |
| ENSMUSG00000090799 | Klhl33 | 2,47 | 0,002292284 | 18,97623 | 56,04469 | 35,46543 | 8,921569 | 2,536743 | 8,007744 |
| ENSMUSG00000075307 | Klhl41 | 4,97 | 1,86955E-12 | 73,79643 | 216,4485 | 122,8315 | 1,982571 | 3,805114 | 7,117995 |
| ENSMUSG00000054920 | Klhl5 | 1,10 | 4,68309E-13 | 5393,465 | 4661,372 | 5302,515 | 2429,641 | 2469,519 | 2272,42 |
| ENSMUSG00000063903 | Klk1 | -2,78 | 1,27128E-10 | 44,27786 | 23,19091 | 49,3056 | 231,9608 | 201,671 | 371,0255 |
| ENSMUSG00000063177 | Klk1b27 | -1,76 | 0,040128186 | 15,81352 | 5,797727 | 44,98055 | 62,45098 | 97,66459 | 66,7312 |
| ENSMUSG00000064023 | Klk8 | -2,06 | 0,002110111 | 49,54903 | 26,08977 | 29,41036 | 94,17212 | 74,83391 | 266,9248 |
| ENSMUSG00000079299 | Klrb1 | -1,89 | 0,022749749 | 5,271174 | 5,797727 | 3,460042 | 14,86928 | 15,22046 | 23,13348 |
| ENSMUSG00000079298 | Klrb1b | -1,69 | 0,04131333 | 122,2912 | 34,78636 | 32,00539 | 102,1024 | 150,9362 | 356,7895 |
| ENSMUSG00000030325 | Klrb1c | -1,59 | 0,001192141 | 341,5721 | 267,6617 | 185,1123 | 598,7364 | 516,2271 | 1281,239 |
| ENSMUSG00000030154 | Klrb1f | 3,40 | 6,70408E-17 | 3164,813 | 5415,077 | 4318,998 | 336,0458 | 629,1122 | 261,5863 |
| ENSMUSG00000022875 | Kng1 | -1,72 | 0,02439182 | 49,54903 | 34,78636 | 88,23108 | 79,30284 | 129,3739 | 359,4587 |
| ENSMUSG00000060459 | Kng2 | -1,79 | 0,00254335 | 44,27786 | 57,01098 | 70,93087 | 116,9717 | 126,8371 | 353,2305 |
| ENSMUSG00000020393 | Kremen1 | 1,17 | 8,96514E-06 | 1297,763 | 1604,038 | 1804,412 | 695,8824 | 569,4987 | 827,4669 |
| ENSMUSG00000023043 | Krt18 | -2,22 | 0,000212007 | 59,03715 | 13,52803 | 32,00539 | 120,9368 | 158,5464 | 204,6424 |
| ENSMUSG00000023039 | Krt7 | 2,58 | 6,68845E-08 | 637,812 | 778,828 | 447,2105 | 170,5011 | 58,34508 | 82,74669 |
| ENSMUSG00000049382 | Krt8 | -3,64 | 0,03784297 | 0 | 0,966288 | 0,865011 | 7,930284 | 11,41534 | 4,448747 |
| ENSMUSG00000067613 | Krt83 | -2,02 | 0,021768792 | 312,0535 | 167,1678 | 135,8067 | 395,5229 | 1867,043 | 229,5553 |
| ENSMUSG00000067614 | Krt86 | -1,69 | 0,000417543 | 44,27786 | 97,59507 | 52,76565 | 265,6645 | 221,965 | 140,5804 |
| ENSMUSG00000047641 | Krt87 | 3,12 | 0,014839788 | 9,488113 | 14,49432 | 12,11015 | 0 | 2,536743 | 1,779499 |
| ENSMUSG00000040213 | Kyat3 | 1,98 | 4,95886E-33 | 785,4049 | 733,4124 | 788,0246 | 194,292 | 186,4506 | 201,9731 |
| ENSMUSG00000026866 | Kynu | -2,22 | 8,90713E-05 | 87,50148 | 28,98863 | 48,44059 | 231,9608 | 156,0097 | 378,1435 |
| ENSMUSG00000035576 | L3mbtl1 | -1,99 | 0,014677918 | 5,271174 | 5,797727 | 8,650106 | 26,76471 | 39,31951 | 13,34624 |
| ENSMUSG00000032796 | Lama1 | 6,00 | 5,24942E-22 | 729,5304 | 252,2011 | 478,3509 | 1,982571 | 11,41534 | 9,787243 |
| ENSMUSG00000019899 | Lama2 | -2,41 | 0,011129667 | 4,216939 | 2,898863 | 5,190064 | 8,921569 | 32,97765 | 24,02323 |
| ENSMUSG00000015647 | Lama5 | -1,70 | 0,000415872 | 44,27786 | 46,38181 | 46,71057 | 111,024 | 100,2013 | 234,0041 |
| ENSMUSG00000002900 | Lamb1 | -1,74 | 0,001104015 | 110,6946 | 40,58409 | 96,88119 | 168,5185 | 352,6072 | 309,6328 |
| ENSMUSG00000031447 | Lamp1 | 1,04 | 2,5237E-07 | 34618,96 | 32435,38 | 43027,36 | 16547,53 | 17705,2 | 19255,96 |
| ENSMUSG00000041247 | Lamp3 | -2,49 | 0,016041599 | 3,162704 | 1,932576 | 1,730021 | 12,88671 | 11,41534 | 13,34624 |
| ENSMUSG00000047344 | Lancl3 | -2,84 | 0,000595373 | 7,379643 | 5,797727 | 9,515116 | 28,74728 | 27,90417 | 105,8802 |
| ENSMUSG00000025762 | Larp1b | -1,55 | 0,000893521 | 22,13893 | 38,65151 | 17,30021 | 73,35512 | 77,37065 | 77,40819 |
| ENSMUSG00000030742 | Lat | -2,02 | 0,027039986 | 562,9614 | 309,2121 | 803,5948 | 695,8824 | 810,4893 | 5296,678 |
| ENSMUSG00000040751 | Lat2 | 2,08 | 6,293E-20 | 7532,507 | 8641,512 | 9137,107 | 2088,638 | 1577,854 | 2333,813 |
| ENSMUSG00000026822 | Lcn2 | -1,77 | 5,50829E-10 | 36379,53 | 45810,74 | 35500,03 | 121598 | 175968,8 | 103888,9 |
| ENSMUSG00000026919 | Lcn4 | 2,73 | 0,000811264 | 28,46434 | 155,5723 | 121,9665 | 19,82571 | 6,341857 | 19,57449 |
| ENSMUSG00000026354 | Lct | -2,89 | 6,03182E-12 | 49,54903 | 38,65151 | 38,92548 | 476,8083 | 243,5273 | 220,6578 |
| ENSMUSG00000021798 | Ldb3 | -2,56 | 0,003131272 | 8,433878 | 5,797727 | 2,595032 | 17,84314 | 25,36743 | 54,27471 |
| ENSMUSG00000030246 | Ldhb | 2,07 | 4,9575E-17 | 2738,902 | 2360,641 | 2703,158 | 626,4924 | 763,5595 | 476,0159 |
| ENSMUSG00000048058 | Ldlrad3 | 2,22 | 6,24681E-15 | 2648,238 | 3066,997 | 3660,725 | 873,3225 | 521,3006 | 613,9271 |
| ENSMUSG00000027985 | Lef1 | -1,84 | 0,004502443 | 318,3789 | 73,43787 | 160,892 | 941,7212 | 644,3326 | 389,7102 |
| ENSMUSG00000038793 | Lefty1 | -2,59 | 1,02192E-06 | 52,71174 | 36,71894 | 43,25053 | 139,7713 | 208,0129 | 450,2132 |
| ENSMUSG00000044857 | Lemd2 | 1,27 | 1,1211E-16 | 5156,262 | 5556,155 | 5544,718 | 2448,475 | 2062,372 | 2236,83 |
| ENSMUSG00000057722 | Lepr | -1,96 | 0,007835389 | 327,867 | 143,9769 | 512,9513 | 423,2789 | 1283,592 | 2113,155 |
| ENSMUSG00000068220 | Lgals1 | 1,71 | 5,19155E-21 | 19532,86 | 17624,12 | 21324,24 | 5267,691 | 5955,003 | 6621,515 |
| ENSMUSG00000053964 | Lgals4 | 1,36 | 7,96029E-06 | 815,9777 | 1224,287 | 875,3907 | 475,817 | 339,9235 | 322,0893 |
| ENSMUSG00000042363 | Lgalsl | -2,29 | 0,007188912 | 723,205 | 432,8969 | 501,7061 | 738,5077 | 1075,579 | 6310,103 |
| ENSMUSG00000045312 | Lhfpl2 | -2,02 | 2,49339E-07 | 440,6701 | 320,8076 | 530,2515 | 1724,837 | 2441,615 | 1089,053 |
| ENSMUSG00000018698 | Lhx1 | -4,27 | 4,3464E-08 | 24,2474 | 3,865151 | 16,4352 | 468,878 | 295,5305 | 90,75444 |
| ENSMUSG00000000247 | Lhx2 | 2,36 | 0,002513612 | 29,51857 | 33,82007 | 16,4352 | 2,973856 | 6,341857 | 6,228246 |
| ENSMUSG00000054263 | Lifr | 1,34 | 5,3477E-05 | 3664,52 | 2472,73 | 3560,384 | 954,6079 | 1206,221 | 1679,847 |
| ENSMUSG00000070873 | Lilra5 | -2,14 | 0,001546726 | 40,06092 | 11,59545 | 27,68034 | 79,30284 | 77,37065 | 191,2961 |
| ENSMUSG00000023022 | Lima1 | 1,15 | 1,56141E-10 | 3389,365 | 3021,582 | 3322,506 | 1405,643 | 1656,493 | 1315,05 |
| ENSMUSG00000029674 | Limk1 | 1,01 | 0,010272954 | 2280,31 | 2263,046 | 2299,198 | 840,6101 | 840,9302 | 1703,87 |
| ENSMUSG00000050966 | Lin28a | -1,15 | 0,00060225 | 1376,831 | 1498,712 | 1111,539 | 3621,166 | 3271,13 | 1963,677 |
| ENSMUSG00000032207 | Lipc | -1,66 | 0,0099594 | 13,70505 | 8,69659 | 25,08531 | 67,40741 | 43,12463 | 40,03872 |
| ENSMUSG00000053846 | Lipg | 4,29 | 1,03602E-09 | 3619,188 | 10615,64 | 6679,612 | 111,024 | 233,3803 | 726,9252 |
| ENSMUSG00000024766 | Lipo3 | 1,04 | 0,001956307 | 333,1382 | 387,4814 | 513,8163 | 233,9434 | 149,6678 | 214,4296 |
| ENSMUSG00000057604 | Lmcd1 | -1,58 | 0,016594257 | 25,30163 | 10,62917 | 44,98055 | 75,3377 | 101,4697 | 66,7312 |
| ENSMUSG00000028063 | Lmna | -3,44 | 1,9008E-10 | 886,6114 | 297,6166 | 538,9016 | 4291,275 | 4049,91 | 10350,45 |
| ENSMUSG00000033060 | Lmo7 | -1,58 | 0,025393788 | 23,19316 | 28,98863 | 36,33044 | 60,46841 | 43,12463 | 160,1549 |
| ENSMUSG00000048814 | Lonrf2 | -5,60 | 0,010025328 | 0 | 0,966288 | 0 | 18,83442 | 25,36743 | 2,669248 |
| ENSMUSG00000016239 | Lonrf3 | 1,27 | 0,001897691 | 636,7578 | 631,9522 | 561,3919 | 296,3944 | 325,9714 | 138,8009 |
| ENSMUSG00000032334 | Loxl1 | -2,09 | 0,004768302 | 64,30832 | 24,1572 | 71,79588 | 72,36384 | 265,0896 | 346,1125 |
| ENSMUSG00000025185 | Loxl4 | 5,08 | 2,47032E-24 | 360,5483 | 172,9655 | 346,8692 | 6,938998 | 11,41534 | 8,007744 |
| ENSMUSG00000049929 | Lpar4 | -2,36 | 0,044453278 | 3,162704 | 0,966288 | 4,325053 | 6,938998 | 12,68371 | 24,02323 |
| ENSMUSG00000021608 | Lpcat1 | -1,47 | 7,32125E-07 | 3454,727 | 2383,832 | 2583,787 | 8970,142 | 8593,216 | 5691,727 |
| ENSMUSG00000033192 | Lpcat2 | -1,33 | 5,96186E-07 | 1043,692 | 1648,487 | 1163,439 | 3634,053 | 2945,158 | 3123,91 |
| ENSMUSG00000004270 | Lpcat3 | -1,00 | 3,30911E-08 | 2544,923 | 2138,395 | 2447,98 | 4625,338 | 5277,693 | 4364,221 |
| ENSMUSG00000020593 | Lpin1 | 1,46 | 3,90228E-08 | 2989,81 | 2225,361 | 2884,81 | 1084,466 | 747,0707 | 1106,848 |
| ENSMUSG00000024052 | Lpin2 | -1,01 | 0,000343321 | 5469,37 | 4095,128 | 3338,076 | 9513,367 | 7610,228 | 8795,172 |
| ENSMUSG00000015568 | Lpl | 1,77 | 0,035229956 | 6167,273 | 19034,9 | 12153,4 | 1740,697 | 1371,109 | 7848,479 |
| ENSMUSG00000024696 | Lpxn | 1,11 | 1,30717E-11 | 2130,608 | 1870,733 | 1960,114 | 927,8432 | 846,0037 | 986,732 |
| ENSMUSG00000040490 | Lrfn2 | 4,29 | 1,31302E-07 | 45,33209 | 186,4935 | 76,98594 | 7,930284 | 6,341857 | 1,779499 |
| ENSMUSG00000037095 | Lrg1 | -1,33 | 7,75781E-06 | 2044,161 | 2864,077 | 2217,887 | 4922,724 | 5202,859 | 7763,063 |
| ENSMUSG00000030029 | Lrig1 | -1,60 | 7,57135E-05 | 100,1523 | 45,41553 | 88,23108 | 214,1177 | 285,3836 | 211,7603 |
| ENSMUSG00000020105 | Lrig3 | -1,46 | 0,016704128 | 28,46434 | 15,4606 | 9,515116 | 45,59913 | 54,53997 | 45,37722 |
| ENSMUSG00000063458 | Lrmda | 1,14 | 0,010056683 | 321,5416 | 409,706 | 501,7061 | 202,2222 | 251,1375 | 109,4392 |
| ENSMUSG00000029103 | Lrpap1 | 1,08 | 4,57169E-09 | 4810,473 | 4414,003 | 5471,192 | 2153,072 | 2271,653 | 2546,463 |
| ENSMUSG00000052316 | Lrrc15 | -2,98 | 0,006735257 | 1,054235 | 0,966288 | 5,190064 | 24,78214 | 10,14697 | 23,13348 |
| ENSMUSG00000039883 | Lrrc17 | 5,98 | 2,17067E-05 | 12,65082 | 171,0329 | 58,82072 | 0 | 0 | 3,558997 |
| ENSMUSG00000041679 | Lrrc29 | -2,18 | 7,16492E-06 | 126,5082 | 153,6398 | 137,5367 | 417,3312 | 393,1951 | 1081,935 |
| ENSMUSG00000090958 | Lrrc32 | -3,77 | 0,003135763 | 209,7927 | 45,41553 | 231,8228 | 725,621 | 818,0995 | 5120,508 |
| ENSMUSG00000054320 | Lrrc36 | -1,62 | 0,011231776 | 18,97623 | 7,730302 | 8,650106 | 32,71242 | 32,97765 | 41,81822 |
| ENSMUSG00000022375 | Lrrc6 | -3,80 | 0,010725978 | 1,054235 | 0 | 1,730021 | 12,88671 | 21,56231 | 5,338496 |
| ENSMUSG00000046807 | Lrrc75b | -3,15 | 3,02969E-06 | 12,65082 | 8,69659 | 10,38013 | 90,20698 | 36,78277 | 153,9266 |
| ENSMUSG00000036295 | Lrrn3 | -2,90 | 1,37701E-05 | 56,92868 | 21,25833 | 62,28076 | 191,3181 | 206,7445 | 646,8478 |
| ENSMUSG00000018819 | Lsp1 | 1,05 | 1,31751E-08 | 47315,11 | 48706,7 | 52948,16 | 27671,73 | 22153,37 | 21979,48 |
| ENSMUSG00000001247 | Lsr | 1,14 | 0,001726609 | 94,88113 | 142,0443 | 125,4265 | 48,57299 | 67,22368 | 49,82596 |
| ENSMUSG00000073412 | Lst1 | 1,41 | 0,000805616 | 6187,304 | 9037,69 | 4789,564 | 3607,288 | 1794,745 | 2144,296 |
| ENSMUSG00000024402 | Lta | -1,86 | 0,000603387 | 26,35587 | 11,59545 | 14,70518 | 62,45098 | 46,92974 | 79,18769 |
| ENSMUSG00000024399 | Ltb | -1,50 | 0,012096226 | 1486,471 | 1599,206 | 700,6586 | 5876,34 | 1705,959 | 3161,28 |
| ENSMUSG00000046908 | Ltb4r1 | -1,17 | 1,67064E-05 | 2282,418 | 3058,301 | 2166,852 | 5999,26 | 6445,863 | 4464,762 |
| ENSMUSG00000040432 | Ltb4r2 | -3,18 | 0,000727999 | 7,379643 | 18,35947 | 5,190064 | 42,62527 | 35,5144 | 200,1936 |
| ENSMUSG00000001870 | Ltbp1 | -2,58 | 0,000880374 | 433,2905 | 286,0212 | 390,9848 | 656,231 | 1023,576 | 4959,463 |
| ENSMUSG00000002020 | Ltbp2 | -1,49 | 0,03229973 | 202,4131 | 151,7072 | 270,7483 | 211,1438 | 462,9555 | 1081,045 |
| ENSMUSG00000032496 | Ltf | -1,81 | 1,34971E-10 | 94974,95 | 106233,7 | 81492,65 | 382561,8 | 378624,1 | 232600,1 |
| ENSMUSG00000027297 | Ltk | -1,41 | 0,025065936 | 27,4101 | 16,42689 | 19,89524 | 87,23312 | 46,92974 | 34,70023 |
| ENSMUSG00000079018 | Ly6c1 | 1,06 | 0,0001245 | 300,4569 | 256,0663 | 343,4092 | 119,9455 | 166,1566 | 146,8086 |
| ENSMUSG00000022584 | Ly6c2 | 2,06 | 2,54184E-14 | 88033,87 | 73448,5 | 81548,01 | 21274,97 | 23195,98 | 13634,52 |
| ENSMUSG00000034634 | Ly6d | -2,03 | 0,00043566 | 1953,497 | 409,706 | 1016,387 | 5446,122 | 4380,955 | 4010,99 |
| ENSMUSG00000022582 | Ly6g | -1,87 | 5,72784E-17 | 1144,899 | 1660,082 | 1170,359 | 4876,133 | 4982,163 | 4722,79 |
| ENSMUSG00000043807 | Ly6g5b | 1,91 | 0,005903558 | 1131,194 | 2708,505 | 1161,709 | 839,6188 | 195,3292 | 295,3968 |
| ENSMUSG00000092586 | Ly6g6c | -4,45 | 0,000888037 | 10,54235 | 0 | 2,595032 | 21,80828 | 32,97765 | 227,7758 |
| ENSMUSG00000073413 | Ly6g6d | -4,02 | 0,000191329 | 7,379643 | 2,898863 | 3,460042 | 38,66013 | 13,95208 | 167,2729 |
| ENSMUSG00000013766 | Ly6g6e | -5,98 | 0,0034817 | 1,054235 | 0 | 0 | 13,878 | 6,341857 | 40,92847 |
| ENSMUSG00000034923 | Ly6g6f | -4,92 | 0,000951357 | 152,864 | 28,02235 | 46,71057 | 395,5229 | 593,5978 | 5907,936 |
| ENSMUSG00000044678 | Ly6k | -1,66 | 0,00534521 | 24,2474 | 13,52803 | 30,27537 | 40,6427 | 83,71251 | 91,64418 |
| ENSMUSG00000026980 | Ly75 | -1,38 | 7,45628E-07 | 1750,03 | 1833,048 | 1438,513 | 4915,785 | 5012,604 | 3151,492 |
| ENSMUSG00000021423 | Ly86 | 1,65 | 1,41276E-09 | 6534,147 | 6876,104 | 5993,658 | 1742,68 | 1686,934 | 2741,318 |
| ENSMUSG00000022594 | Lynx1 | -1,59 | 0,017655753 | 367,9279 | 85,99961 | 237,8779 | 927,8432 | 777,5116 | 371,0255 |
| ENSMUSG00000050447 | Lypd6 | -3,18 | 0,005624772 | 14,75929 | 1,932576 | 7,785095 | 13,878 | 43,12463 | 162,8241 |
| ENSMUSG00000026765 | Lypd6b | -1,55 | 0,005812228 | 24,2474 | 27,05606 | 57,95571 | 96,15469 | 147,1311 | 78,29794 |
| ENSMUSG00000039246 | Lyplal1 | -1,80 | 0,007795742 | 14,75929 | 7,730302 | 6,055074 | 28,74728 | 26,6358 | 42,70797 |
| ENSMUSG00000032184 | Lysmd2 | -1,88 | 0,036442902 | 25,30163 | 25,12348 | 12,11015 | 33,70371 | 35,5144 | 159,2651 |
| ENSMUSG00000030787 | Lyve1 | -3,09 | 1,86163E-14 | 35,84398 | 15,4606 | 17,30021 | 173,475 | 192,7924 | 213,5398 |
| ENSMUSG00000069515 | Lyz1 | -1,17 | 0,011355914 | 102,2608 | 58,94356 | 74,39091 | 159,597 | 121,7636 | 245,5708 |
| ENSMUSG00000068205 | Macrod2 | -1,54 | 0,040818705 | 50,60327 | 22,22462 | 21,62526 | 54,5207 | 57,07671 | 161,9344 |
| ENSMUSG00000036634 | Mag | 2,18 | 5,94225E-26 | 828,6285 | 820,3783 | 1036,283 | 185,3704 | 194,0608 | 213,5398 |
| ENSMUSG00000025151 | Maged1 | -1,40 | 0,011639277 | 395,338 | 216,4485 | 352,9243 | 541,2419 | 555,5466 | 1439,614 |
| ENSMUSG00000025268 | Maged2 | -1,99 | 0,015451895 | 283,5891 | 203,8867 | 288,0485 | 327,1242 | 436,3197 | 2317,797 |
| ENSMUSG00000031224 | Magee2 | -1,52 | 0,001012003 | 14,75929 | 24,1572 | 15,57019 | 55,51199 | 48,19811 | 52,49521 |
| ENSMUSG00000028164 | Manba | 1,14 | 1,41479E-13 | 3810,004 | 3755,961 | 4126,966 | 1892,364 | 1785,867 | 1618,454 |
| ENSMUSG00000032718 | Mansc1 | 3,09 | 6,02363E-23 | 1592,949 | 1900,688 | 1891,778 | 151,6667 | 181,3771 | 297,1763 |
| ENSMUSG00000027254 | Map1a | -1,98 | 0,000813229 | 22,13893 | 18,35947 | 19,03023 | 36,67756 | 86,24925 | 112,1084 |
| ENSMUSG00000052727 | Map1b | -1,21 | 0,010294035 | 30,57281 | 28,98863 | 32,00539 | 45,59913 | 87,51762 | 79,18769 |
| ENSMUSG00000033618 | Map3k13 | -2,10 | 0,013482226 | 16,86776 | 2,898863 | 8,650106 | 44,60785 | 54,53997 | 22,24373 |
| ENSMUSG00000028862 | Map3k6 | 1,66 | 0,000518258 | 1661,474 | 1147,95 | 1565,669 | 353,8889 | 763,5595 | 269,5941 |
| ENSMUSG00000034761 | Map4k5 | -1,58 | 4,13547E-08 | 1687,83 | 1074,512 | 1200,635 | 4481,602 | 4210,993 | 3130,138 |
| ENSMUSG00000067878 | Map7d3 | 1,20 | 0,003555684 | 157,081 | 201,9542 | 165,217 | 68,3987 | 50,73485 | 107,6597 |
| ENSMUSG00000053137 | Mapk11 | -1,20 | 1,2972E-05 | 171,8403 | 142,0443 | 154,8369 | 443,1046 | 325,9714 | 304,2943 |
| ENSMUSG00000022610 | Mapk12 | -1,34 | 2,54274E-05 | 49,54903 | 58,94356 | 47,57558 | 141,7538 | 106,5432 | 145,9189 |
| ENSMUSG00000004864 | Mapk13 | -1,37 | 6,47216E-05 | 1312,522 | 1763,475 | 1171,224 | 4893,976 | 3489,29 | 2628,32 |
| ENSMUSG00000024558 | Mapk4 | -1,04 | 0,01089626 | 118,0743 | 83,10075 | 108,1263 | 215,1089 | 280,3101 | 142,3599 |
| ENSMUSG00000032577 | Mapkapk3 | 1,03 | 1,47308E-06 | 14791,97 | 14132,93 | 15788,17 | 8585,523 | 7242,4 | 6035,17 |
| ENSMUSG00000078627 | March10 | 4,43 | 0,00035837 | 7,379643 | 40,58409 | 100,3412 | 0,991285 | 1,268371 | 4,448747 |
| ENSMUSG00000079557 | March2 | -2,36 | 9,64566E-36 | 3760,455 | 2900,796 | 3305,205 | 18723,4 | 15275 | 17155,26 |
| ENSMUSG00000032656 | March3 | -3,62 | 5,64427E-12 | 385,8499 | 134,314 | 189,4373 | 4399,325 | 1609,563 | 2698,61 |
| ENSMUSG00000023307 | March5 | -1,03 | 7,17036E-07 | 2684,082 | 2060,126 | 2274,113 | 5175,501 | 4954,258 | 4203,176 |
| ENSMUSG00000025702 | March8 | -2,00 | 8,45713E-13 | 3864,825 | 2348,079 | 2761,979 | 13535,01 | 12615,22 | 9861,982 |
| ENSMUSG00000040502 | March9 | -1,36 | 0,005841765 | 33,73551 | 18,35947 | 24,2203 | 65,42484 | 48,19811 | 80,96719 |
| ENSMUSG00000069662 | Marcks | -1,71 | 4,55623E-13 | 1379,993 | 1023,299 | 1414,292 | 4048,41 | 3604,711 | 4794,859 |
| ENSMUSG00000026390 | Marco | -3,91 | 0,002364221 | 1,054235 | 0 | 4,325053 | 11,89543 | 17,7572 | 53,38496 |
| ENSMUSG00000044345 | Marveld1 | -1,14 | 3,21694E-14 | 1002,577 | 1046,49 | 913,4512 | 2160,011 | 2253,896 | 2094,47 |
| ENSMUSG00000034751 | Mast4 | -1,96 | 1,20489E-05 | 435,3989 | 269,5943 | 275,0734 | 1038,867 | 797,8056 | 1977,913 |
| ENSMUSG00000022324 | Matn2 | 1,68 | 5,30901E-06 | 473,3514 | 457,0541 | 611,5625 | 109,0414 | 144,5943 | 227,7758 |
| ENSMUSG00000032344 | Mb21d1 | 1,09 | 4,06968E-06 | 2150,639 | 1814,688 | 1861,503 | 891,1656 | 1098,41 | 752,728 |
| ENSMUSG00000020646 | Mboat2 | -2,60 | 4,45685E-11 | 240,3655 | 175,8644 | 104,6663 | 991,2855 | 1362,231 | 804,3334 |
| ENSMUSG00000045569 | Mc2r | 2,79 | 0,000202343 | 33,73551 | 166,2015 | 109,8563 | 19,82571 | 7,610228 | 16,90524 |
| ENSMUSG00000007480 | Mc5r | -2,46 | 3,72034E-05 | 11,59658 | 22,22462 | 13,84017 | 52,53813 | 71,02879 | 138,8009 |
| ENSMUSG00000032135 | Mcam | -2,88 | 8,59827E-18 | 124,3997 | 94,6962 | 135,8067 | 1084,466 | 961,4255 | 576,5576 |
| ENSMUSG00000013974 | Mcemp1 | -1,38 | 7,54929E-06 | 4798,877 | 4721,282 | 4009,324 | 12813,36 | 14525,39 | 7864,495 |
| ENSMUSG00000031442 | Mcf2l | 1,63 | 3,42308E-11 | 4230,644 | 3960,814 | 4190,111 | 1241,089 | 1057,822 | 1694,083 |
| ENSMUSG00000011008 | Mcoln2 | -1,87 | 5,63053E-05 | 120,1828 | 57,01098 | 85,63605 | 272,6035 | 220,6966 | 463,5594 |
| ENSMUSG00000036853 | Mcoln3 | -1,71 | 0,018450739 | 21,08469 | 4,831439 | 17,30021 | 32,71242 | 57,07671 | 51,60546 |
| ENSMUSG00000022157 | Mcpt8 | -1,42 | 1,03118E-05 | 1440,085 | 1659,116 | 887,5009 | 4134,652 | 3274,935 | 3228,9 |
| ENSMUSG00000032776 | Mctp2 | -2,04 | 5,3121E-13 | 698,9576 | 389,414 | 482,6759 | 2033,126 | 2106,765 | 2299,112 |
| ENSMUSG00000027994 | Mcub | 2,49 | 5,34971E-17 | 1603,491 | 1833,048 | 2087,271 | 372,7233 | 393,1951 | 221,5476 |
| ENSMUSG00000056476 | Med12l | -2,27 | 0,004617157 | 220,3351 | 182,6284 | 227,4978 | 347,9412 | 388,1216 | 2303,561 |
| ENSMUSG00000022534 | Mefv | 1,70 | 2,80094E-06 | 6501,466 | 5305,886 | 5496,277 | 1980,588 | 2313,509 | 1035,668 |
| ENSMUSG00000021268 | Meg3 | 5,54 | 1,95553E-08 | 63,25408 | 945,9958 | 145,3218 | 7,930284 | 12,68371 | 4,448747 |
| ENSMUSG00000043289 | Mei4 | -2,15 | 0,000278907 | 16,86776 | 4,831439 | 17,30021 | 64,43356 | 58,34508 | 50,71571 |
| ENSMUSG00000020160 | Meis1 | -2,30 | 0,000585308 | 190,8165 | 166,2015 | 197,2224 | 405,4358 | 442,6616 | 1888,938 |
| ENSMUSG00000058704 | Memo1 | -1,26 | 3,19302E-20 | 1917,653 | 1803,093 | 1941,949 | 4640,207 | 4246,507 | 4639,153 |
| ENSMUSG00000014361 | Mertk | -2,56 | 3,18781E-06 | 225,6062 | 124,6511 | 278,5334 | 863,4096 | 697,6042 | 2152,304 |
| ENSMUSG00000051855 | Mest | -2,03 | 0,003725178 | 27,4101 | 49,28068 | 62,28076 | 121,9281 | 88,78599 | 356,7895 |
| ENSMUSG00000009376 | Met | 2,44 | 4,52163E-07 | 3947,055 | 2966,504 | 3688,405 | 769,2375 | 919,5692 | 264,2556 |
| ENSMUSG00000036112 | Metap2 | -1,35 | 4,72201E-11 | 8015,347 | 6421,949 | 7585,278 | 20934,96 | 19432,72 | 15938,97 |
| ENSMUSG00000040557 | Mettl27 | -2,52 | 1,91319E-48 | 160,2437 | 157,5049 | 156,5669 | 894,1395 | 977,9143 | 848,8209 |
| ENSMUSG00000030876 | Mettl9 | -1,01 | 1,21917E-12 | 2562,845 | 2626,37 | 2550,051 | 5178,475 | 5608,738 | 4850,024 |
| ENSMUSG00000074480 | Mex3a | -1,18 | 0,017844492 | 167,6233 | 112,0894 | 223,1727 | 338,0283 | 243,5273 | 559,6524 |
| ENSMUSG00000030605 | Mfge8 | 1,49 | 3,63786E-06 | 2073,68 | 1942,238 | 2586,382 | 642,353 | 624,0387 | 1079,266 |
| ENSMUSG00000025227 | Mfsd13a | 1,15 | 0,000166429 | 2328,805 | 2608,977 | 2164,256 | 1046,797 | 1390,135 | 765,1845 |
| ENSMUSG00000037336 | Mfsd2b | -3,12 | 8,61941E-14 | 1669,908 | 1055,186 | 967,0818 | 7570,447 | 7875,318 | 16629,42 |
| ENSMUSG00000029490 | Mfsd7a | 2,37 | 5,5909E-12 | 1501,23 | 2468,865 | 1940,219 | 471,8519 | 419,8309 | 252,6888 |
| ENSMUSG00000041945 | Mfsd9 | -1,15 | 5,45305E-05 | 154,9725 | 226,1113 | 144,4568 | 407,4183 | 399,537 | 359,4587 |
| ENSMUSG00000068587 | Mgam | 2,36 | 1,07644E-17 | 8874,548 | 11283,34 | 11969,15 | 2730,991 | 1828,991 | 1714,547 |
| ENSMUSG00000036155 | Mgat5 | 1,13 | 1,23665E-07 | 3686,659 | 2806,1 | 3459,177 | 1666,351 | 1551,218 | 1328,396 |
| ENSMUSG00000040950 | Mgl2 | -2,31 | 0,001145797 | 250,9079 | 70,53901 | 96,88119 | 674,0741 | 1136,461 | 269,5941 |
| ENSMUSG00000033174 | Mgll | -2,38 | 1,83054E-27 | 1184,96 | 1089,973 | 954,9717 | 6590,066 | 5641,716 | 4609,791 |
| ENSMUSG00000054612 | Mgmt | -1,80 | 0,023198238 | 139,159 | 172,9655 | 211,9276 | 261,6994 | 237,1854 | 1320,388 |
| ENSMUSG00000026688 | Mgst3 | -2,45 | 5,92654E-11 | 944,5943 | 406,8072 | 675,5733 | 3022,429 | 4506,523 | 3532,305 |
| ENSMUSG00000038244 | Mical2 | 4,72 | 7,65879E-32 | 5684,434 | 5746,514 | 4916,72 | 295,4031 | 97,66459 | 227,7758 |
| ENSMUSG00000051586 | Mical3 | -1,96 | 1,76408E-11 | 623,0527 | 397,1443 | 400,4999 | 2064,848 | 1489,068 | 1972,574 |
| ENSMUSG00000030771 | Micalcl | 4,13 | 2,37065E-17 | 131,7793 | 151,7072 | 109,8563 | 11,89543 | 3,805114 | 6,228246 |
| ENSMUSG00000036718 | Micall2 | -1,48 | 2,85255E-10 | 718,9881 | 568,1772 | 660,8681 | 1774,401 | 2161,305 | 1506,346 |
| ENSMUSG00000042570 | Mier2 | 1,07 | 9,25687E-06 | 716,8796 | 658,042 | 638,3778 | 359,8366 | 248,6008 | 346,1125 |
| ENSMUSG00000040987 | Mill2 | -1,87 | 0,036994215 | 13,70505 | 9,662878 | 12,97516 | 22,79957 | 20,29394 | 88,97494 |
| ENSMUSG00000040528 | Milr1 | 1,26 | 6,1311E-12 | 3446,293 | 4122,184 | 4261,042 | 1575,153 | 1562,633 | 1807,081 |
| ENSMUSG00000024896 | Minpp1 | -1,07 | 3,42677E-05 | 2582,875 | 1867,834 | 2108,031 | 4163,399 | 5569,419 | 4037,683 |
| ENSMUSG00000065401 | Mir144 | -4,68 | 0,000698225 | 0 | 0,966288 | 1,730021 | 46,59042 | 12,68371 | 12,45649 |
| ENSMUSG00000088015 | Mir1932 | 1,98 | 0,017293576 | 15,81352 | 26,08977 | 15,57019 | 4,956427 | 6,341857 | 3,558997 |
| ENSMUSG00000070065 | Mir451a | -3,52 | 3,17443E-05 | 39,00669 | 5,797727 | 4,325053 | 260,7081 | 152,2046 | 145,9189 |
| ENSMUSG00000099145 | Mir8116 | -3,42 | 3,79653E-13 | 24,2474 | 24,1572 | 22,49028 | 197,2658 | 158,5464 | 398,6077 |
| ENSMUSG00000097391 | Mirg | 9,83 | 0,003959101 | 6,325408 | 86,9659 | 11,24514 | 0 | 0 | 0 |
| ENSMUSG00000092920 | Mirt2 | -1,93 | 9,77E-08 | 147,5929 | 192,2913 | 102,9363 | 559,085 | 705,2145 | 419,072 |
| ENSMUSG00000035158 | Mitf | 1,90 | 1,45868E-15 | 908,7503 | 1190,467 | 1245,615 | 301,3508 | 253,6743 | 337,215 |
| ENSMUSG00000086212 | Mkln1os | -2,92 | 1,35645E-06 | 5,271174 | 5,797727 | 4,325053 | 42,62527 | 32,97765 | 40,03872 |
| ENSMUSG00000029922 | Mkrn1 | -2,04 | 1,3786E-21 | 10519,15 | 7162,125 | 8639,726 | 38538,21 | 34005,04 | 35806,18 |
| ENSMUSG00000012519 | Mlkl | 1,83 | 4,04852E-41 | 2541,76 | 2536,505 | 2732,568 | 709,7604 | 759,7544 | 724,256 |
| ENSMUSG00000003948 | Mmd | -1,50 | 0,018277844 | 959,3536 | 623,2556 | 897,881 | 1290,654 | 1239,199 | 4487,006 |
| ENSMUSG00000027820 | Mme | -2,18 | 0,004647988 | 26,35587 | 28,98863 | 76,98594 | 75,3377 | 166,1566 | 361,2382 |
| ENSMUSG00000000901 | Mmp11 | -1,49 | 3,90283E-06 | 55,87444 | 50,24697 | 70,06586 | 195,2832 | 163,6199 | 136,1317 |
| ENSMUSG00000049723 | Mmp12 | 2,24 | 0,000159905 | 106,4777 | 107,2579 | 268,1533 | 25,77342 | 25,36743 | 50,71571 |
| ENSMUSG00000000957 | Mmp14 | -2,06 | 6,03613E-05 | 135,9963 | 101,4602 | 196,3574 | 343,9761 | 481,9811 | 984,0628 |
| ENSMUSG00000029061 | Mmp23 | -1,95 | 0,000258179 | 32,68128 | 30,92121 | 25,95032 | 91,19826 | 68,49205 | 184,1781 |
| ENSMUSG00000023903 | Mmp25 | -2,46 | 8,0178E-29 | 1549,725 | 1078,377 | 1129,704 | 7280 | 7146,004 | 6290,528 |
| ENSMUSG00000020682 | Mmp28 | 2,95 | 5,90868E-11 | 663,1137 | 1085,141 | 887,5009 | 175,4575 | 107,8116 | 58,72346 |
| ENSMUSG00000005800 | Mmp8 | -2,11 | 1,48808E-13 | 12659,25 | 13240,08 | 12802,16 | 49092,42 | 76471,38 | 42063,79 |
| ENSMUSG00000017737 | Mmp9 | -1,51 | 4,38752E-05 | 19072,16 | 38326,84 | 22762,75 | 94941,36 | 63389,39 | 70272,41 |
| ENSMUSG00000054641 | Mmrn1 | -3,70 | 1,29746E-05 | 383,7414 | 182,6284 | 346,8692 | 973,4423 | 1409,161 | 9470,492 |
| ENSMUSG00000041445 | Mmrn2 | -1,63 | 0,002576013 | 137,0505 | 55,0784 | 141,8617 | 261,6994 | 260,0161 | 508,9366 |
| ENSMUSG00000090272 | Mndal | 1,09 | 0,001455625 | 3840,577 | 5643,121 | 3989,429 | 2574,368 | 1472,579 | 2291,105 |
| ENSMUSG00000032221 | Mns1 | -1,60 | 0,002069921 | 847,6047 | 245,4371 | 723,1488 | 2050,97 | 1532,193 | 1904,953 |
| ENSMUSG00000073910 | Mob3b | 1,29 | 2,1461E-08 | 767,4829 | 1049,389 | 866,7406 | 353,8889 | 331,0449 | 411,0642 |
| ENSMUSG00000056458 | Mok | 4,21 | 5,65452E-58 | 448,0498 | 459,953 | 451,5355 | 21,80828 | 22,83068 | 28,47198 |
| ENSMUSG00000028402 | Mpdz | -1,84 | 0,00384047 | 45,33209 | 40,58409 | 62,28076 | 79,30284 | 135,7157 | 316,7508 |
| ENSMUSG00000046805 | Mpeg1 | 1,43 | 1,60153E-13 | 85751,45 | 85503,91 | 91105,51 | 31136,28 | 28061,45 | 38337,52 |
| ENSMUSG00000006389 | Mpl | -3,29 | 9,99465E-05 | 356,3313 | 227,0776 | 275,0734 | 763,2898 | 885,3232 | 6754,087 |
| ENSMUSG00000017314 | Mpp2 | -3,43 | 1,74869E-11 | 230,8774 | 54,11212 | 116,7764 | 1667,342 | 1314,033 | 1350,64 |
| ENSMUSG00000021112 | Mpp5 | -1,08 | 1,58399E-05 | 735,8558 | 610,6939 | 661,7331 | 1172,691 | 1334,327 | 1745,688 |
| ENSMUSG00000038388 | Mpp6 | 1,06 | 1,60578E-12 | 2967,671 | 3114,346 | 3225,624 | 1368,965 | 1506,825 | 1580,195 |
| ENSMUSG00000026566 | Mpzl1 | -2,48 | 2,48651E-27 | 152,864 | 112,0894 | 160,027 | 831,6885 | 716,6298 | 822,1284 |
| ENSMUSG00000070305 | Mpzl3 | -1,73 | 5,94174E-06 | 309,945 | 343,9985 | 215,3876 | 1257,941 | 1008,355 | 608,5886 |
| ENSMUSG00000026471 | Mr1 | -1,17 | 0,02626395 | 88,55572 | 91,79734 | 125,4265 | 162,5708 | 153,4729 | 372,805 |
| ENSMUSG00000032470 | Mras | -2,21 | 0,003713045 | 93,82689 | 21,25833 | 96,88119 | 213,1264 | 181,3771 | 583,6756 |
| ENSMUSG00000093973 | Mrgpra2a | -1,91 | 2,65759E-05 | 30,57281 | 54,11212 | 23,35529 | 108,0501 | 162,3515 | 134,3522 |
| ENSMUSG00000096719 | Mrgpra2b | -1,78 | 3,71319E-06 | 219,2808 | 373,9534 | 175,5971 | 842,5926 | 1083,189 | 717,138 |
| ENSMUSG00000070547 | Mrgprb1 | -7,87 | 0,04131333 | 0 | 0 | 0 | 1,982571 | 1,268371 | 21,35398 |
| ENSMUSG00000048965 | Mrgpre | -1,33 | 0,000540286 | 119,1285 | 57,01098 | 80,44598 | 198,2571 | 200,4027 | 244,6811 |
| ENSMUSG00000018405 | Mrm1 | -1,08 | 3,25066E-08 | 949,8655 | 794,2886 | 762,9393 | 1949,859 | 1637,467 | 1707,429 |
| ENSMUSG00000037772 | Mrpl23 | 1,06 | 3,9532E-09 | 569,2868 | 601,9973 | 651,353 | 290,4466 | 309,4826 | 274,0428 |
| ENSMUSG00000005611 | Mrvi1 | -2,75 | 0,031784699 | 756,9405 | 476,3799 | 1174,684 | 1500,806 | 1681,86 | 12976,99 |
| ENSMUSG00000024673 | Ms4a1 | -2,40 | 1,86863E-05 | 1071,102 | 252,2011 | 658,2731 | 4494,488 | 3593,296 | 2386,308 |
| ENSMUSG00000024680 | Ms4a2 | -1,05 | 0,002392603 | 190,8165 | 207,7519 | 116,7764 | 397,5055 | 370,3644 | 301,625 |
| ENSMUSG00000024681 | Ms4a3 | 2,25 | 0,000167615 | 27711,61 | 25125,42 | 28592,92 | 5643,388 | 9710,651 | 1751,027 |
| ENSMUSG00000024675 | Ms4a4c | 2,39 | 1,53332E-35 | 6282,185 | 7581,494 | 6664,042 | 1518,649 | 1204,953 | 1201,162 |
| ENSMUSG00000024677 | Ms4a6b | 1,99 | 1,69205E-22 | 9220,337 | 7728,37 | 7781,635 | 2127,299 | 1727,522 | 2347,159 |
| ENSMUSG00000079419 | Ms4a6c | 2,38 | 1,81399E-19 | 22409,87 | 24682,86 | 26270,37 | 4824,586 | 5854,802 | 3388,166 |
| ENSMUSG00000069769 | Msi2 | -1,21 | 0,000201364 | 1112,218 | 739,2102 | 1215,34 | 2258,148 | 1882,263 | 2953,968 |
| ENSMUSG00000051236 | Msrb3 | -1,51 | 0,031152194 | 40,06092 | 29,95492 | 64,01078 | 51,54684 | 125,5688 | 203,7526 |
| ENSMUSG00000032591 | Mst1 | -2,12 | 5,33195E-12 | 56,92868 | 39,6178 | 37,19546 | 218,0828 | 185,1822 | 176,1704 |
| ENSMUSG00000048450 | Msx1 | 4,06 | 9,17947E-16 | 197,1419 | 145,9095 | 241,338 | 5,947713 | 8,878599 | 19,57449 |
| ENSMUSG00000031762 | Mt2 | -1,79 | 8,17949E-05 | 267,7756 | 148,8083 | 356,3844 | 569,9891 | 1071,774 | 1024,102 |
| ENSMUSG00000004748 | Mtfp1 | -1,11 | 8,35679E-07 | 218,2266 | 158,4712 | 181,6522 | 404,4445 | 394,4635 | 403,9462 |
| ENSMUSG00000027601 | Mtfr1 | -1,36 | 1,52111E-05 | 2366,757 | 1371,162 | 1970,494 | 5681,057 | 5148,319 | 3850,835 |
| ENSMUSG00000035078 | Mtmr9 | 1,10 | 0,000330826 | 3492,68 | 6505,049 | 4826,759 | 2368,181 | 2354,097 | 2172,768 |
| ENSMUSG00000033763 | Mtss1l | -1,99 | 0,019959777 | 95,93536 | 112,0894 | 271,6133 | 315,2288 | 225,7701 | 1364,876 |
| ENSMUSG00000038065 | Mturn | -1,49 | 4,18213E-05 | 133,8878 | 99,52764 | 132,3466 | 445,0872 | 352,6072 | 230,4451 |
| ENSMUSG00000059908 | Mug1 | -6,76 | 0,009709195 | 0 | 0 | 0,865011 | 8,921569 | 0 | 90,75444 |
| ENSMUSG00000042515 | Mum1l1 | -3,27 | 0,012752688 | 1,054235 | 2,898863 | 0,865011 | 10,90414 | 6,341857 | 28,47198 |
| ENSMUSG00000000386 | Mx1 | 2,70 | 2,14586E-07 | 1568,701 | 4574,406 | 2288,818 | 578,9107 | 240,9906 | 479,5749 |
| ENSMUSG00000023341 | Mx2 | 1,95 | 0,00037208 | 316,2704 | 783,6594 | 407,42 | 159,597 | 64,68694 | 164,6036 |
| ENSMUSG00000001156 | Mxd1 | -1,44 | 7,69059E-06 | 16956,31 | 13036,19 | 12764,96 | 40595,12 | 49070,75 | 26223,58 |
| ENSMUSG00000025141 | Myadml2 | 3,85 | 1,31914E-07 | 282,5349 | 989,4787 | 615,0225 | 20,81699 | 19,02557 | 89,86469 |
| ENSMUSG00000084822 | Myadml2os | 3,22 | 0,049295638 | 4,216939 | 16,42689 | 7,785095 | 0 | 1,268371 | 1,779499 |
| ENSMUSG00000068745 | Mybphl | 2,48 | 0,024871083 | 12,65082 | 14,49432 | 17,30021 | 4,956427 | 0 | 2,669248 |
| ENSMUSG00000046916 | Myct1 | -2,61 | 0,001770315 | 167,6233 | 191,325 | 153,9719 | 314,2375 | 364,0226 | 2449,48 |
| ENSMUSG00000020900 | Myh10 | -3,40 | 1,11242E-10 | 572,4495 | 126,5837 | 285,4535 | 3370,371 | 3937,025 | 3047,392 |
| ENSMUSG00000060180 | Myh13 | -3,82 | 8,87857E-05 | 3,162704 | 0,966288 | 0,865011 | 24,78214 | 24,09906 | 19,57449 |
| ENSMUSG00000030739 | Myh14 | -2,73 | 0,013102967 | 2,108469 | 1,932576 | 6,920085 | 13,878 | 12,68371 | 47,15672 |
| ENSMUSG00000061816 | Myl1 | 2,79 | 0,045529272 | 46,38633 | 80,20189 | 1,730021 | 6,938998 | 2,536743 | 8,897494 |
| ENSMUSG00000013936 | Myl2 | -2,68 | 1,64052E-09 | 10,54235 | 23,19091 | 17,30021 | 133,8235 | 110,3483 | 85,41594 |
| ENSMUSG00000067818 | Myl9 | -4,94 | 0,001758477 | 431,182 | 27,05606 | 121,1015 | 1404,652 | 2371,854 | 14055,37 |
| ENSMUSG00000038175 | Mylip | -1,20 | 5,91986E-06 | 2078,951 | 1492,915 | 1488,683 | 4541,079 | 3793,699 | 3254,703 |
| ENSMUSG00000022836 | Mylk | -2,39 | 0,00110837 | 632,5408 | 333,3693 | 560,5269 | 1048,78 | 1227,783 | 5734,435 |
| ENSMUSG00000031698 | Mylk3 | -3,68 | 2,92215E-07 | 404,8261 | 52,17954 | 128,8866 | 2978,813 | 3088,484 | 1428,048 |
| ENSMUSG00000030672 | Mylpf | -1,20 | 0,001152783 | 173,9487 | 167,1678 | 91,69112 | 382,6362 | 319,6296 | 290,0583 |
| ENSMUSG00000042678 | Myo15 | -1,51 | 0,016059748 | 13,70505 | 35,75265 | 18,16522 | 89,21569 | 59,61345 | 44,48747 |
| ENSMUSG00000072720 | Myo18b | 6,02 | 1,60279E-64 | 4473,118 | 4520,294 | 4169,351 | 67,40741 | 100,2013 | 36,47972 |
| ENSMUSG00000018417 | Myo1b | -1,83 | 4,67187E-07 | 123,3455 | 65,70757 | 120,2365 | 296,3944 | 358,9491 | 443,9849 |
| ENSMUSG00000035441 | Myo1d | -1,88 | 4,56182E-19 | 595,6426 | 553,6829 | 445,4805 | 2065,839 | 2083,934 | 1713,657 |
| ENSMUSG00000032220 | Myo1e | -1,50 | 9,21821E-08 | 455,4294 | 261,864 | 398,7699 | 1105,283 | 1010,892 | 1033,889 |
| ENSMUSG00000034593 | Myo5a | 1,05 | 1,26749E-08 | 13417,25 | 15871,28 | 15571,92 | 7716,166 | 7643,206 | 6350,141 |
| ENSMUSG00000025885 | Myo5b | -1,88 | 0,008137969 | 5,271174 | 9,662878 | 17,30021 | 25,77342 | 50,73485 | 43,59772 |
| ENSMUSG00000020067 | Mypn | -3,32 | 0,03541267 | 0 | 1,932576 | 0,865011 | 13,878 | 10,14697 | 4,448747 |
| ENSMUSG00000041361 | Myzap | -1,26 | 0,011129667 | 31,62704 | 16,42689 | 36,33044 | 76,32898 | 68,49205 | 57,83371 |
| ENSMUSG00000001053 | N4bp3 | -1,26 | 4,04136E-06 | 201,3588 | 175,8644 | 210,1976 | 521,4162 | 355,144 | 532,0701 |
| ENSMUSG00000097432 | NA | -3,47 | 7,77309E-05 | 3,162704 | 6,764015 | 0 | 36,67756 | 36,78277 | 34,70023 |
| ENSMUSG00000072855 | NA | -3,05 | 1,94588E-13 | 165,5149 | 74,40416 | 99,47622 | 683,987 | 830,7832 | 1292,806 |
| ENSMUSG00000094924 | NA | -2,97 | 0,000684297 | 10,54235 | 0,966288 | 2,595032 | 34,69499 | 31,70928 | 41,81822 |
| ENSMUSG00000086344 | NA | -2,41 | 0,011231235 | 3,162704 | 2,898863 | 4,325053 | 12,88671 | 12,68371 | 29,36173 |
| ENSMUSG00000093809 | NA | -2,38 | 2,50522E-05 | 15,81352 | 21,25833 | 16,4352 | 55,51199 | 73,56554 | 148,5881 |
| ENSMUSG00000078933 | NA | -2,10 | 0,010272954 | 73,79643 | 75,37045 | 86,50106 | 115,9804 | 140,7892 | 753,6177 |
| ENSMUSG00000073854 | NA | -2,06 | 2,59583E-05 | 12,65082 | 11,59545 | 15,57019 | 54,5207 | 69,76042 | 42,70797 |
| ENSMUSG00000003178 | NA | -2,02 | 3,06438E-09 | 267,7756 | 144,9432 | 137,5367 | 773,2027 | 681,1154 | 769,6332 |
| ENSMUSG00000099065 | NA | -1,90 | 0,002938236 | 1505,447 | 462,8519 | 1295,786 | 6191,569 | 4224,945 | 1794,624 |
| ENSMUSG00000062808 | NA | -1,80 | 0,000239079 | 15,81352 | 18,35947 | 24,2203 | 62,45098 | 49,46648 | 90,75444 |
| ENSMUSG00000096581 | NA | -1,60 | 0,030596951 | 44,27786 | 25,12348 | 29,41036 | 43,61656 | 71,02879 | 183,2884 |
| ENSMUSG00000029333 | NA | -1,42 | 0,011513482 | 295,1857 | 115,9545 | 197,2224 | 374,7059 | 394,4635 | 861,2774 |
| ENSMUSG00000037814 | NA | -1,03 | 3,23719E-09 | 760,1032 | 668,6712 | 731,799 | 1433,399 | 1628,589 | 1364,876 |
| ENSMUSG00000053664 | NA | 1,54 | 6,39857E-06 | 672,6018 | 418,4026 | 730,9339 | 175,4575 | 260,0161 | 193,0756 |
| ENSMUSG00000028269 | NA | 2,93 | 0,045372537 | 55,87444 | 7,730302 | 4,325053 | 2,973856 | 1,268371 | 4,448747 |
| ENSMUSG00000087349 | NA | 7,73 | 0,0274941 | 9,488113 | 9,662878 | 5,190064 | 0 | 0 | 0 |
| ENSMUSG00000029413 | Naaa | 2,12 | 9,13089E-31 | 8136,584 | 9161,375 | 7958,962 | 1762,506 | 2246,286 | 1820,427 |
| ENSMUSG00000022253 | Nadk2 | -1,30 | 4,7505E-08 | 2182,266 | 1638,824 | 1818,252 | 5172,528 | 4913,671 | 3762,75 |
| ENSMUSG00000022453 | Naga | 1,23 | 2,94316E-13 | 5871,033 | 7012,351 | 7125,957 | 2782,538 | 2735,877 | 3025,148 |
| ENSMUSG00000048217 | Nags | -4,37 | 3,66681E-07 | 4,216939 | 0 | 1,730021 | 43,61656 | 32,97765 | 42,70797 |
| ENSMUSG00000071203 | Naip5 | 1,42 | 2,90787E-07 | 4035,611 | 6307,927 | 4323,323 | 2113,421 | 1779,525 | 1599,769 |
| ENSMUSG00000078942 | Naip6/Naip6 | 1,76 | 4,25786E-12 | 3192,223 | 4754,136 | 3110,578 | 1184,586 | 1045,138 | 1045,456 |
| ENSMUSG00000072437 | Nanos1 | -1,47 | 7,97599E-06 | 175,003 | 197,1227 | 151,3769 | 577,9194 | 546,668 | 328,3175 |
| ENSMUSG00000027438 | Napb | 1,49 | 1,93961E-05 | 119,1285 | 138,1792 | 98,61121 | 46,59042 | 32,97765 | 46,26697 |
| ENSMUSG00000022574 | Naprt | -1,42 | 1,73295E-09 | 195,0334 | 209,6845 | 148,7818 | 524,39 | 451,5402 | 505,3776 |
| ENSMUSG00000002204 | Napsa | 1,19 | 0,000156291 | 12741,48 | 12600,39 | 14937 | 6212,386 | 7491,001 | 4003,872 |
| ENSMUSG00000068299 | Nat8f4 | -1,15 | 0,000905335 | 62,19985 | 62,80871 | 76,98594 | 111,024 | 167,425 | 170,8319 |
| ENSMUSG00000027799 | Nbea | -2,38 | 9,43888E-05 | 30,57281 | 16,42689 | 17,30021 | 51,54684 | 115,4218 | 166,3831 |
| ENSMUSG00000039542 | Ncam1 | -1,36 | 0,042935382 | 335,2466 | 494,7394 | 448,0755 | 1170,708 | 1775,72 | 338,1048 |
| ENSMUSG00000047586 | Nccrp1 | 1,85 | 0,012836837 | 25,30163 | 33,82007 | 83,90603 | 15,86057 | 11,41534 | 12,45649 |
| ENSMUSG00000066877 | Nck2 | -1,46 | 0,019184442 | 453,3209 | 293,7515 | 517,2763 | 704,804 | 622,7703 | 2158,532 |
| ENSMUSG00000027002 | Nckap1 | -2,37 | 0,000287024 | 395,338 | 201,9542 | 401,3649 | 786,0894 | 988,0613 | 3373,04 |
| ENSMUSG00000032598 | Nckipsd | 1,00 | 9,88632E-07 | 1860,724 | 1895,857 | 1774,137 | 972,451 | 1029,918 | 759,846 |
| ENSMUSG00000056234 | Ncoa4 | -1,10 | 1,27453E-09 | 2218,11 | 2206,035 | 1941,949 | 4445,915 | 5122,952 | 4097,296 |
| ENSMUSG00000029478 | Ncor2 | 1,21 | 1,05564E-07 | 8963,104 | 11832,19 | 10906,92 | 5397,549 | 4223,677 | 4120,429 |
| ENSMUSG00000033585 | Ndn | -2,36 | 0,029364118 | 4,216939 | 2,898863 | 7,785095 | 8,921569 | 17,7572 | 49,82596 |
| ENSMUSG00000049001 | Ndnf | 2,74 | 6,44621E-05 | 100,1523 | 396,178 | 196,3574 | 21,80828 | 22,83068 | 58,72346 |
| ENSMUSG00000026950 | Neb | 1,02 | 0,047759893 | 214,0097 | 175,8644 | 92,55613 | 61,4597 | 74,83391 | 101,4314 |
| ENSMUSG00000032012 | Nectin1 | 2,48 | 8,02705E-07 | 667,3306 | 461,8856 | 869,3356 | 137,7887 | 52,00322 | 168,1626 |
| ENSMUSG00000062300 | Nectin2 | 1,49 | 0,00130271 | 390,0669 | 336,2682 | 429,9103 | 91,19826 | 96,39622 | 222,4373 |
| ENSMUSG00000022656 | Nectin3 | -1,68 | 0,015794015 | 21,08469 | 7,730302 | 10,38013 | 25,77342 | 48,19811 | 50,71571 |
| ENSMUSG00000032216 | Nedd4 | -1,38 | 4,19141E-12 | 5413,495 | 5274,965 | 4934,02 | 11516,75 | 13226,58 | 15935,41 |
| ENSMUSG00000020396 | Nefh | -1,60 | 7,97477E-08 | 118,0743 | 95,66249 | 118,5065 | 314,2375 | 267,6264 | 426,1899 |
| ENSMUSG00000026749 | Nek6 | -1,29 | 1,55732E-07 | 409,0431 | 366,2231 | 366,7645 | 723,6384 | 990,598 | 1084,604 |
| ENSMUSG00000021215 | Net1 | -1,32 | 2,07216E-08 | 1936,629 | 1332,511 | 1746,456 | 4086,079 | 4708,194 | 3760,971 |
| ENSMUSG00000036902 | Neto2 | -1,35 | 0,018549416 | 242,474 | 227,0776 | 216,2526 | 683,987 | 846,0037 | 219,7681 |
| ENSMUSG00000035239 | Neu3 | -1,28 | 0,001245657 | 288,8603 | 155,5723 | 166,082 | 516,4597 | 367,8277 | 597,0218 |
| ENSMUSG00000006435 | Neurl1a | -3,90 | 9,99366E-05 | 25,30163 | 15,4606 | 20,76025 | 63,44227 | 71,02879 | 782,9794 |
| ENSMUSG00000047180 | Neurl3 | -1,04 | 0,000624132 | 2667,214 | 3131,739 | 2377,914 | 6229,238 | 3966,197 | 6600,161 |
| ENSMUSG00000048015 | Neurod4 | -7,87 | 0,026366595 | 0 | 0 | 0 | 13,878 | 6,341857 | 4,448747 |
| ENSMUSG00000028565 | Nfia | -1,70 | 2,37065E-17 | 177,1114 | 145,9095 | 189,4373 | 541,2419 | 577,109 | 552,5344 |
| ENSMUSG00000072889 | Nfxl1 | 1,11 | 2,98825E-06 | 1764,789 | 2275,608 | 1956,654 | 972,451 | 1050,211 | 754,5075 |
| ENSMUSG00000000120 | Ngfr | -2,37 | 7,35487E-08 | 17,92199 | 9,662878 | 12,11015 | 72,36384 | 64,68694 | 66,7312 |
| ENSMUSG00000032484 | Ngp | -1,06 | 0,00417884 | 202973,9 | 267990,3 | 205723,7 | 495860,8 | 620702,9 | 297999,3 |
| ENSMUSG00000090113 | Nhlrc4 | -4,00 | 8,37749E-10 | 28,46434 | 5,797727 | 5,190064 | 200,2397 | 182,6455 | 238,4528 |
| ENSMUSG00000005397 | Nid1 | -1,84 | 0,000178038 | 178,1657 | 74,40416 | 178,1922 | 318,2026 | 596,1345 | 622,8246 |
| ENSMUSG00000021806 | Nid2 | -1,97 | 1,42435E-05 | 20,03046 | 27,05606 | 45,84556 | 107,0588 | 106,5432 | 152,1471 |
| ENSMUSG00000068115 | Ninl | -2,78 | 7,47266E-12 | 196,0877 | 98,56136 | 97,7462 | 1041,841 | 1036,259 | 612,1476 |
| ENSMUSG00000047037 | Nipa1 | -2,34 | 1,99125E-10 | 228,7689 | 103,3928 | 126,2915 | 694,8911 | 736,9237 | 879,0724 |
| ENSMUSG00000020411 | Nipal4 | 4,74 | 0,030586397 | 14,75929 | 10,62917 | 3,460042 | 0 | 1,268371 | 0 |
| ENSMUSG00000059395 | Nkapl | 2,67 | 0,003932739 | 13,70505 | 40,58409 | 18,16522 | 4,956427 | 3,805114 | 2,669248 |
| ENSMUSG00000021567 | Nkd2 | -1,62 | 0,000851858 | 14,75929 | 12,56174 | 19,89524 | 54,5207 | 40,58788 | 49,82596 |
| ENSMUSG00000004612 | Nkg7 | -1,01 | 0,017021132 | 2389,95 | 1205,927 | 1191,985 | 2578,333 | 3902,779 | 3189,751 |
| ENSMUSG00000001496 | Nkx2-1 | 7,88 | 0,033435738 | 3,162704 | 21,25833 | 2,595032 | 0 | 0 | 0 |
| ENSMUSG00000039193 | Nlrc4 | 2,29 | 1,45486E-17 | 2755,77 | 2143,226 | 1764,622 | 544,2157 | 419,8309 | 401,277 |
| ENSMUSG00000049709 | Nlrp10 | 3,60 | 1,57009E-23 | 423,8024 | 836,8052 | 660,8681 | 48,57299 | 44,393 | 64,9517 |
| ENSMUSG00000078817 | Nlrp12 | -1,09 | 0,012370543 | 928,7808 | 1582,779 | 745,6391 | 2909,423 | 2365,513 | 1670,06 |
| ENSMUSG00000069830 | Nlrp1a | 1,84 | 9,42438E-08 | 1398,97 | 1423,342 | 1278,486 | 479,7822 | 440,1249 | 226,8861 |
| ENSMUSG00000070390 | Nlrp1b | 1,55 | 0,010991141 | 499,7073 | 827,1424 | 393,5798 | 193,3007 | 81,17577 | 310,5225 |
| ENSMUSG00000038745 | Nlrp6 | -6,25 | 3,43395E-08 | 6,325408 | 0,966288 | 4,325053 | 56,50327 | 65,95531 | 755,3972 |
| ENSMUSG00000032109 | Nlrx1 | -1,19 | 3,35974E-06 | 1800,633 | 2169,316 | 1583,834 | 3565,654 | 4000,443 | 5145,421 |
| ENSMUSG00000032456 | Nmnat3 | -1,22 | 0,000459602 | 423,8024 | 455,1216 | 629,7277 | 809,8802 | 1248,077 | 1452,961 |
| ENSMUSG00000034738 | Nostrin | 1,95 | 5,25536E-11 | 340,5178 | 547,8852 | 402,2299 | 95,1634 | 117,9585 | 121,8957 |
| ENSMUSG00000038146 | Notch3 | -1,30 | 0,037243856 | 108,5862 | 122,7186 | 211,0626 | 183,3878 | 290,457 | 618,3758 |
| ENSMUSG00000042988 | Notum | 3,97 | 0,000458287 | 10,54235 | 50,24697 | 32,00539 | 2,973856 | 0 | 2,669248 |
| ENSMUSG00000053613 | Notumos | 3,40 | 0,030808068 | 7,379643 | 20,29204 | 6,055074 | 0 | 2,536743 | 0,889749 |
| ENSMUSG00000030411 | Nova2 | -2,21 | 0,026033717 | 4,216939 | 1,932576 | 4,325053 | 8,921569 | 24,09906 | 16,01549 |
| ENSMUSG00000072919 | Noxred1 | -1,48 | 0,044296147 | 30,57281 | 18,35947 | 10,38013 | 43,61656 | 34,24603 | 86,30569 |
| ENSMUSG00000021010 | Npas3 | 6,06 | 7,2898E-23 | 232,9859 | 164,2689 | 223,1727 | 0 | 5,073485 | 4,448747 |
| ENSMUSG00000045903 | Npas4 | -6,34 | 2,20938E-08 | 5,271174 | 2,898863 | 0 | 242,8649 | 24,09906 | 376,364 |
| ENSMUSG00000021242 | Npc2 | 1,35 | 5,77403E-14 | 29442,67 | 24258,66 | 30723,45 | 11074,64 | 10354,98 | 11790,07 |
| ENSMUSG00000020289 | Nprl3 | -1,27 | 8,36192E-11 | 725,3135 | 902,5128 | 762,9393 | 1777,375 | 1842,944 | 2154,083 |
| ENSMUSG00000003849 | Nqo1 | -2,90 | 6,89728E-28 | 122,2912 | 71,5053 | 93,42114 | 665,1525 | 706,4828 | 774,0819 |
| ENSMUSG00000037583 | Nr0b2 | 1,99 | 0,000148086 | 56,92868 | 79,2356 | 46,71057 | 9,912855 | 16,48883 | 19,57449 |
| ENSMUSG00000002108 | Nr1h3 | -1,62 | 0,000724646 | 188,708 | 175,8644 | 304,4837 | 431,2092 | 556,815 | 1067,699 |
| ENSMUSG00000031618 | Nr3c2 | -1,94 | 0,013287012 | 39,00669 | 5,797727 | 13,84017 | 67,40741 | 64,68694 | 91,64418 |
| ENSMUSG00000065873 | n-R5s183 | -2,08 | 0,003985408 | 4,216939 | 4,831439 | 12,97516 | 31,72113 | 36,78277 | 26,69248 |
| ENSMUSG00000049134 | Nrap | -1,38 | 0,046868778 | 12,65082 | 11,59545 | 10,38013 | 19,82571 | 25,36743 | 44,48747 |
| ENSMUSG00000078202 | Nrarp | -1,78 | 4,11388E-07 | 79,06761 | 47,3481 | 55,36068 | 198,2571 | 168,6934 | 255,3581 |
| ENSMUSG00000060275 | Nrg2 | 2,65 | 3,196E-15 | 1286,166 | 1058,085 | 1286,271 | 205,1961 | 256,211 | 118,3367 |
| ENSMUSG00000032311 | Nrg4 | 1,68 | 0,044317728 | 140,2132 | 962,4227 | 233,5529 | 113,9978 | 188,9873 | 112,9982 |
| ENSMUSG00000053310 | Nrgn | -1,94 | 0,035598148 | 521,8462 | 629,0534 | 1207,555 | 1054,728 | 924,6427 | 7052,153 |
| ENSMUSG00000034825 | Nrip3 | -3,21 | 2,56011E-14 | 61,14562 | 19,32576 | 36,33044 | 363,8018 | 360,2175 | 353,2305 |
| ENSMUSG00000025810 | Nrp1 | 1,79 | 1,54821E-13 | 4991,802 | 5663,413 | 6330,147 | 1347,157 | 1533,461 | 2024,18 |
| ENSMUSG00000025969 | Nrp2 | -1,51 | 0,007253444 | 168,6776 | 146,8757 | 360,7094 | 370,7408 | 617,6968 | 938,6856 |
| ENSMUSG00000052384 | Nrros | 1,09 | 1,2002E-11 | 19930,31 | 18652,25 | 21384,79 | 9486,602 | 10108,92 | 8529,137 |
| ENSMUSG00000024109 | Nrxn1 | -2,82 | 5,16976E-08 | 14,75929 | 6,764015 | 22,49028 | 106,0675 | 125,5688 | 81,85694 |
| ENSMUSG00000033768 | Nrxn2 | -3,81 | 5,5909E-12 | 7,379643 | 5,797727 | 6,055074 | 61,4597 | 84,98088 | 122,7854 |
| ENSMUSG00000020297 | Nsg2 | -1,24 | 0,013987046 | 88,55572 | 74,40416 | 108,9913 | 222,0479 | 112,885 | 305,184 |
| ENSMUSG00000029780 | Nt5c3 | -1,74 | 7,27852E-19 | 3705,635 | 2685,314 | 3351,051 | 10256,83 | 11266,94 | 10952,81 |
| ENSMUSG00000020019 | Ntn4 | 1,16 | 8,99621E-05 | 664,1679 | 964,3552 | 1045,798 | 325,1416 | 436,3197 | 432,4182 |
| ENSMUSG00000020032 | Nuak1 | -1,93 | 4,12376E-06 | 176,0572 | 85,99961 | 168,6771 | 755,3595 | 455,3453 | 434,1977 |
| ENSMUSG00000024228 | Nudt12 | -1,47 | 9,18572E-05 | 46,38633 | 64,74128 | 59,68573 | 111,024 | 169,9618 | 193,9654 |
| ENSMUSG00000030717 | Nupr1 | 1,41 | 3,73406E-07 | 1007,848 | 791,3897 | 885,7708 | 253,7691 | 336,1184 | 416,4027 |
| ENSMUSG00000032028 | Nxpe2 | -3,17 | 1,29419E-07 | 1390,536 | 235,7742 | 561,3919 | 6530,589 | 6609,483 | 6547,666 |
| ENSMUSG00000075033 | Nxpe3 | 1,72 | 1,32253E-05 | 7190,935 | 3037,043 | 3845,837 | 1344,183 | 1300,081 | 1621,123 |
| ENSMUSG00000047592 | Nxpe5 | 2,14 | 0,000861809 | 446,9955 | 883,1871 | 1525,014 | 117,963 | 158,5464 | 371,0255 |
| ENSMUSG00000001166 | Oas1c | -1,39 | 0,000206647 | 85,39301 | 161,3701 | 112,4514 | 276,5686 | 270,1631 | 395,9385 |
| ENSMUSG00000066867 | Oas1e | -3,68 | 0,000210465 | 8,433878 | 2,898863 | 0 | 45,59913 | 27,90417 | 66,7312 |
| ENSMUSG00000061462 | Obscn | -3,16 | 9,94263E-11 | 46,38633 | 22,22462 | 21,62526 | 203,2135 | 201,671 | 395,0487 |
| ENSMUSG00000026211 | Obsl1 | -2,05 | 0,001245286 | 17,92199 | 34,78636 | 14,70518 | 70,38127 | 58,34508 | 149,4779 |
| ENSMUSG00000011179 | Odc1 | -1,85 | 3,13214E-18 | 5075,086 | 4027,488 | 4675,382 | 18907,78 | 16799,58 | 13814,25 |
| ENSMUSG00000021390 | Ogn | -2,73 | 0,000115962 | 29,51857 | 19,32576 | 27,68034 | 67,40741 | 105,2748 | 334,5458 |
| ENSMUSG00000009654 | Oit3 | -1,88 | 0,037131998 | 22,13893 | 3,865151 | 10,38013 | 29,73856 | 30,44091 | 72,0697 |
| ENSMUSG00000026833 | Olfm1 | 3,03 | 4,58601E-20 | 5195,269 | 8455,018 | 6738,432 | 823,7582 | 1093,336 | 578,3371 |
| ENSMUSG00000022026 | Olfm4 | -3,34 | 8,17648E-08 | 2221,273 | 1128,624 | 1211,88 | 16144,08 | 25558,95 | 4513,699 |
| ENSMUSG00000046618 | Olfml2a | -1,57 | 0,008700295 | 55,87444 | 51,21325 | 94,28615 | 102,1024 | 168,6934 | 325,6483 |
| ENSMUSG00000038463 | Olfml2b | -1,13 | 0,004221137 | 1654,094 | 2071,721 | 1519,824 | 4646,155 | 4604,188 | 2234,161 |
| ENSMUSG00000027848 | Olfml3 | -1,65 | 0,036847173 | 115,9658 | 58,94356 | 152,2419 | 127,8758 | 220,6966 | 677,0993 |
| ENSMUSG00000050742 | Olfr164 | -3,38 | 0,000409133 | 16,86776 | 5,797727 | 8,650106 | 60,46841 | 25,36743 | 237,5631 |
| ENSMUSG00000050788 | Olfr419 | -5,19 | 0,012250662 | 1,054235 | 0 | 0 | 5,947713 | 19,02557 | 10,67699 |
| ENSMUSG00000055033 | Olfr420 | -3,07 | 0,001931925 | 12,65082 | 0 | 4,325053 | 49,56427 | 54,53997 | 36,47972 |
| ENSMUSG00000091950 | Olfr421-ps1 | -3,92 | 0,005645035 | 3,162704 | 0 | 0 | 11,89543 | 19,02557 | 14,23599 |
| ENSMUSG00000074006 | Omp | -2,07 | 0,006803406 | 43,22362 | 8,69659 | 9,515116 | 79,30284 | 90,05436 | 86,30569 |
| ENSMUSG00000032346 | Ooep | 7,27 | 9,5747E-16 | 246,6909 | 139,1454 | 540,6316 | 0 | 1,268371 | 4,448747 |
| ENSMUSG00000041857 | Oosp1 | 1,02 | 0,011443464 | 85,39301 | 65,70757 | 95,15116 | 42,62527 | 46,92974 | 32,92073 |
| ENSMUSG00000031214 | Ophn1 | -1,66 | 0,049786774 | 220,3351 | 243,5045 | 237,0129 | 236,9172 | 315,8245 | 1659,383 |
| ENSMUSG00000026525 | Opn3 | 1,36 | 0,001138601 | 164,4606 | 288,9201 | 218,8477 | 76,32898 | 64,68694 | 119,2264 |
| ENSMUSG00000026672 | Optn | -2,03 | 1,03683E-21 | 972,0044 | 883,1871 | 724,8789 | 3207,8 | 4023,274 | 3304,529 |
| ENSMUSG00000028587 | Orc1 | -1,34 | 1,9311E-10 | 417,477 | 418,4026 | 418,6651 | 1113,214 | 1189,732 | 867,5056 |
| ENSMUSG00000028359 | Orm3 | 4,58 | 1,81401E-26 | 348,9517 | 532,4246 | 448,0755 | 10,90414 | 31,70928 | 14,23599 |
| ENSMUSG00000038150 | Ormdl3 | -1,37 | 8,30595E-06 | 1898,677 | 1171,141 | 1564,804 | 3536,907 | 3403,04 | 5054,666 |
| ENSMUSG00000024687 | Osbp | 1,64 | 3,79564E-15 | 16607,36 | 16705,18 | 18205,01 | 5907,07 | 6226,435 | 4417,606 |
| ENSMUSG00000042359 | Osbpl6 | 2,25 | 0,00109564 | 66,41679 | 325,639 | 127,1566 | 27,75599 | 36,78277 | 44,48747 |
| ENSMUSG00000054594 | Oscar | -2,41 | 0,039972369 | 3,162704 | 0,966288 | 7,785095 | 10,90414 | 13,95208 | 39,14897 |
| ENSMUSG00000022146 | Osmr | -1,05 | 0,010532469 | 60,09138 | 55,0784 | 50,17061 | 77,32027 | 143,326 | 121,8957 |
| ENSMUSG00000044055 | Otos | 1,94 | 8,14645E-06 | 127,5624 | 222,2462 | 192,8974 | 37,66885 | 35,5144 | 67,62095 |
| ENSMUSG00000021203 | Otub2 | -1,93 | 3,19378E-06 | 631,4866 | 329,5041 | 460,1856 | 2405,85 | 1906,362 | 1107,738 |
| ENSMUSG00000038495 | Otud7b | -1,76 | 2,72171E-07 | 1562,376 | 1133,456 | 1163,439 | 3491,307 | 3401,772 | 6203,333 |
| ENSMUSG00000076438 | Oxct2b | -7,85 | 0,02721005 | 0 | 0 | 0 | 4,956427 | 5,073485 | 14,23599 |
| ENSMUSG00000049112 | Oxtr | 3,15 | 0,000696895 | 13,70505 | 23,19091 | 26,81533 | 2,973856 | 2,536743 | 1,779499 |
| ENSMUSG00000029468 | P2rx7 | 1,20 | 9,23988E-05 | 1554,996 | 1840,778 | 2043,155 | 838,6275 | 554,2783 | 971,6063 |
| ENSMUSG00000050921 | P2ry10 | -1,58 | 0,009352699 | 588,263 | 132,3814 | 501,7061 | 969,4772 | 1352,084 | 1325,727 |
| ENSMUSG00000036353 | P2ry12 | -3,91 | 8,59503E-08 | 51,6575 | 34,78636 | 51,03562 | 249,8039 | 296,7989 | 1520,582 |
| ENSMUSG00000036362 | P2ry13 | -2,43 | 7,74128E-09 | 195,0334 | 132,3814 | 195,4924 | 652,2658 | 697,6042 | 1473,425 |
| ENSMUSG00000036381 | P2ry14 | -1,59 | 0,006718456 | 274,101 | 91,79734 | 138,4017 | 295,4031 | 487,0546 | 738,492 |
| ENSMUSG00000038168 | P3h2 | -2,57 | 0,010654882 | 27,4101 | 8,69659 | 39,79049 | 34,69499 | 83,71251 | 333,656 |
| ENSMUSG00000054582 | Pabpc1l | -1,71 | 2,40456E-08 | 100,1523 | 96,62878 | 85,63605 | 300,3595 | 389,39 | 232,2246 |
| ENSMUSG00000011257 | Pabpc4 | -1,20 | 1,39488E-08 | 3685,605 | 2645,696 | 2818,204 | 7012,353 | 7313,429 | 6674,9 |
| ENSMUSG00000040276 | Pacsin1 | -2,35 | 0,000516521 | 121,237 | 156,5386 | 92,55613 | 356,8628 | 258,7478 | 1270,562 |
| ENSMUSG00000028927 | Padi2 | 2,28 | 3,47263E-19 | 8426,498 | 11864,08 | 11031,48 | 2654,662 | 1837,87 | 1962,787 |
| ENSMUSG00000025330 | Padi4 | -1,11 | 0,005131243 | 4193,746 | 7598,887 | 3887,358 | 13991 | 10853,45 | 9046,082 |
| ENSMUSG00000020092 | Pald1 | -1,65 | 0,002805266 | 40,06092 | 34,78636 | 45,84556 | 74,34641 | 96,39622 | 207,3116 |
| ENSMUSG00000027188 | Pamr1 | -2,97 | 0,000880374 | 4,216939 | 4,831439 | 8,650106 | 19,82571 | 29,17254 | 89,86469 |
| ENSMUSG00000097814 | Panct2 | -2,22 | 3,5871E-07 | 219,2808 | 163,3026 | 123,6965 | 1058,693 | 866,2976 | 432,4182 |
| ENSMUSG00000025464 | Paox | 1,07 | 1,94568E-08 | 636,7578 | 647,4128 | 724,0139 | 293,4205 | 313,2877 | 351,451 |
| ENSMUSG00000021223 | Papln | -1,56 | 0,044124286 | 12,65082 | 5,797727 | 12,97516 | 16,85185 | 32,97765 | 42,70797 |
| ENSMUSG00000028370 | Pappa | -1,59 | 0,013176851 | 87,50148 | 34,78636 | 79,58097 | 125,8933 | 138,2525 | 343,4433 |
| ENSMUSG00000024899 | Papss2 | 2,81 | 1,18614E-10 | 5472,533 | 5192,831 | 6045,559 | 846,5578 | 1177,049 | 368,3562 |
| ENSMUSG00000032278 | Paqr5 | -2,17 | 3,5358E-07 | 53,76597 | 48,31439 | 41,52051 | 132,8323 | 202,9394 | 308,743 |
| ENSMUSG00000064225 | Paqr9 | -3,41 | 3,15731E-09 | 382,6872 | 73,43787 | 143,5918 | 2248,235 | 2133,401 | 1982,362 |
| ENSMUSG00000025812 | Pard3 | -1,47 | 0,001652237 | 18,97623 | 22,22462 | 32,00539 | 50,55556 | 77,37065 | 76,51845 |
| ENSMUSG00000052062 | Pard3b | -2,88 | 0,000138759 | 126,5082 | 65,70757 | 71,79588 | 259,7168 | 272,6998 | 1410,253 |
| ENSMUSG00000056214 | Pard6g | -1,06 | 0,009242943 | 72,7422 | 67,64015 | 103,8013 | 155,6318 | 129,3739 | 223,3271 |
| ENSMUSG00000034981 | Parm1 | -2,86 | 0,004647988 | 33,73551 | 18,35947 | 15,57019 | 57,49456 | 35,5144 | 397,718 |
| ENSMUSG00000032392 | Parp16 | -1,93 | 4,25695E-11 | 274,101 | 214,5159 | 181,6522 | 912,9739 | 995,6715 | 649,517 |
| ENSMUSG00000021725 | Parp8 | 1,47 | 1,8929E-09 | 5824,647 | 7480,034 | 5817,196 | 2641,776 | 2440,346 | 1840,002 |
| ENSMUSG00000030770 | Parva | -1,47 | 0,036010569 | 55,87444 | 31,8875 | 69,20085 | 55,51199 | 153,4729 | 225,1066 |
| ENSMUSG00000022438 | Parvb | -2,13 | 0,003628227 | 4574,325 | 2662,123 | 4074,2 | 6207,43 | 7979,324 | 35354,19 |
| ENSMUSG00000061859 | Patj | -1,25 | 7,16943E-05 | 114,9116 | 84,06704 | 125,4265 | 263,6819 | 206,7445 | 298,066 |
| ENSMUSG00000035873 | Pawr | -1,78 | 0,035742425 | 12,65082 | 9,662878 | 8,650106 | 17,84314 | 22,83068 | 64,9517 |
| ENSMUSG00000014030 | Pax5 | -1,85 | 0,003316514 | 643,0832 | 209,6845 | 506,8962 | 2433,606 | 1740,205 | 710,9097 |
| ENSMUSG00000026976 | Pax8 | -2,23 | 0,006835202 | 4,216939 | 4,831439 | 14,70518 | 43,61656 | 49,46648 | 20,46424 |
| ENSMUSG00000001497 | Pax9 | -9,38 | 0,003527273 | 0 | 0 | 0 | 27,75599 | 24,09906 | 18,68474 |
| ENSMUSG00000052534 | Pbx1 | -1,08 | 0,003603681 | 1485,417 | 1967,362 | 2001,634 | 2703,235 | 3403,04 | 5433,699 |
| ENSMUSG00000041650 | Pcca | 1,08 | 8,67636E-10 | 1700,481 | 1519,004 | 1772,407 | 744,4554 | 748,3391 | 866,6159 |
| ENSMUSG00000037892 | Pcdh18 | 2,28 | 0,000131837 | 52,71174 | 119,8197 | 164,352 | 14,86928 | 27,90417 | 26,69248 |
| ENSMUSG00000029108 | Pcdh7 | 4,05 | 1,13131E-24 | 1892,351 | 4006,229 | 2839,83 | 130,8497 | 145,8627 | 248,2401 |
| ENSMUSG00000047910 | Pcdhb16 | 1,43 | 0,014751215 | 35,84398 | 99,52764 | 67,47083 | 27,75599 | 25,36743 | 22,24373 |
| ENSMUSG00000024892 | Pcx | -2,45 | 3,39583E-18 | 413,26 | 284,0886 | 345,1392 | 2366,198 | 1859,432 | 1471,645 |
| ENSMUSG00000029998 | Pcyox1 | 1,08 | 9,66259E-12 | 8536,139 | 8408,636 | 8905,284 | 4559,913 | 3882,485 | 3824,143 |
| ENSMUSG00000024579 | Pcyox1l | 1,38 | 1,63936E-10 | 1873,375 | 1904,553 | 1674,66 | 724,6297 | 801,6107 | 566,7703 |
| ENSMUSG00000035246 | Pcyt1b | -2,34 | 1,41884E-13 | 412,2058 | 212,5833 | 288,0485 | 1403,66 | 1410,429 | 1808,86 |
| ENSMUSG00000026285 | Pdcd1 | 1,73 | 0,030116331 | 474,4056 | 63,77499 | 275,9384 | 55,51199 | 82,44414 | 106,7699 |
| ENSMUSG00000024975 | Pdcd4 | 1,73 | 6,79E-21 | 14825,7 | 12827,47 | 17008,7 | 4270,458 | 4474,814 | 4759,269 |
| ENSMUSG00000075270 | Pde11a | 2,88 | 0,000116607 | 25,30163 | 40,58409 | 30,27537 | 2,973856 | 2,536743 | 7,117995 |
| ENSMUSG00000041741 | Pde3a | -3,62 | 6,497E-05 | 103,315 | 29,95492 | 44,98055 | 199,2484 | 263,8212 | 1720,775 |
| ENSMUSG00000031842 | Pde4c | -1,57 | 0,044129635 | 62,19985 | 39,6178 | 75,25592 | 298,3769 | 178,8404 | 48,93622 |
| ENSMUSG00000053965 | Pde5a | -3,25 | 6,18383E-05 | 530,2801 | 335,3019 | 489,596 | 1181,612 | 1595,611 | 10110,22 |
| ENSMUSG00000019990 | Pde7b | -2,11 | 0,000319654 | 177,1114 | 77,30302 | 81,31099 | 343,9761 | 811,7577 | 288,2788 |
| ENSMUSG00000025584 | Pde8a | -1,41 | 0,000734441 | 446,9955 | 420,3352 | 517,2763 | 1160,795 | 1809,966 | 710,02 |
| ENSMUSG00000021684 | Pde8b | 1,82 | 6,09702E-05 | 201,3588 | 422,2678 | 275,0734 | 74,34641 | 60,88182 | 119,2264 |
| ENSMUSG00000041119 | Pde9a | 6,92 | 4,98996E-61 | 1900,785 | 2658,258 | 2705,753 | 24,78214 | 6,341857 | 27,58223 |
| ENSMUSG00000025856 | Pdgfa | -2,87 | 1,60849E-05 | 27,4101 | 32,85379 | 35,46543 | 114,9891 | 119,2269 | 464,4492 |
| ENSMUSG00000000489 | Pdgfb | -2,75 | 0,000446326 | 214,0097 | 155,5723 | 139,2667 | 426,2527 | 404,6105 | 2581,163 |
| ENSMUSG00000028019 | Pdgfc | -1,90 | 0,003098965 | 35,84398 | 20,29204 | 66,60582 | 100,1198 | 117,9585 | 240,2323 |
| ENSMUSG00000032006 | Pdgfd | -2,08 | 2,05079E-06 | 28,46434 | 14,49432 | 16,4352 | 76,32898 | 96,39622 | 76,51845 |
| ENSMUSG00000055044 | Pdlim1 | -2,01 | 1,15926E-05 | 586,1545 | 420,3352 | 647,0279 | 1478,998 | 1534,729 | 3664,878 |
| ENSMUSG00000032788 | Pdxk | 1,25 | 4,68207E-07 | 6133,538 | 4237,172 | 5178,818 | 2103,508 | 2543,085 | 1904,953 |
| ENSMUSG00000022197 | Pdzd2 | -1,64 | 0,006507539 | 146,5386 | 67,64015 | 103,8013 | 202,2222 | 213,0864 | 572,1088 |
| ENSMUSG00000032105 | Pdzd3 | -1,74 | 0,005085868 | 39,00669 | 34,78636 | 33,73541 | 108,0501 | 50,73485 | 200,1936 |
| ENSMUSG00000028716 | Pdzk1ip1 | -2,00 | 1,1266E-15 | 1390,536 | 924,7374 | 1132,299 | 3985,959 | 4429,153 | 5372,307 |
| ENSMUSG00000024227 | Pdzph1 | 2,70 | 0,000319477 | 176,0572 | 131,4151 | 80,44598 | 7,930284 | 10,14697 | 40,92847 |
| ENSMUSG00000028073 | Pear1 | -2,01 | 0,002419012 | 392,1753 | 396,178 | 425,5852 | 813,8454 | 735,6554 | 3350,796 |
| ENSMUSG00000045573 | Penk | 1,69 | 0,001857261 | 249,8536 | 229,0102 | 450,6705 | 100,1198 | 140,7892 | 48,04647 |
| ENSMUSG00000063931 | Pepd | 1,29 | 8,01425E-10 | 3120,535 | 3024,481 | 4014,514 | 1292,636 | 1345,742 | 1521,471 |
| ENSMUSG00000067825 | Pex26 | 1,12 | 2,25631E-06 | 1875,484 | 1299,657 | 1604,595 | 815,8279 | 677,3103 | 709,1302 |
| ENSMUSG00000033065 | Pfkm | -1,10 | 1,9892E-09 | 895,0453 | 920,8723 | 999,0872 | 1851,721 | 1900,02 | 2298,223 |
| ENSMUSG00000021196 | Pfkp | 1,32 | 1,08197E-13 | 8559,332 | 7147,631 | 8032,488 | 3019,456 | 3547,635 | 2955,747 |
| ENSMUSG00000020639 | Pfn4 | -2,20 | 0,032037383 | 4,216939 | 2,898863 | 4,325053 | 12,88671 | 8,878599 | 30,25148 |
| ENSMUSG00000011752 | Pgam1 | 1,25 | 3,78226E-07 | 4450,979 | 4012,993 | 5014,466 | 1882,451 | 2285,605 | 1514,353 |
| ENSMUSG00000020475 | Pgam2 | 1,46 | 1,74993E-11 | 617,7816 | 646,4465 | 712,7687 | 264,6732 | 196,5976 | 255,3581 |
| ENSMUSG00000030413 | Pglyrp1 | -1,26 | 1,07614E-05 | 9280,428 | 12435,16 | 9155,272 | 27489,34 | 28306,24 | 18306,59 |
| ENSMUSG00000079563 | Pglyrp2 | -1,18 | 0,037439032 | 173,9487 | 113,0557 | 193,7624 | 206,1874 | 282,8468 | 599,6911 |
| ENSMUSG00000042250 | Pglyrp4 | -1,46 | 0,032872328 | 12,65082 | 9,662878 | 12,97516 | 44,60785 | 34,24603 | 18,68474 |
| ENSMUSG00000056131 | Pgm3 | 1,03 | 6,78168E-07 | 780,1337 | 913,142 | 829,5452 | 416,3399 | 462,9555 | 354,1202 |
| ENSMUSG00000057457 | Phex | -1,35 | 0,044748306 | 20,03046 | 13,52803 | 36,33044 | 38,66013 | 55,80834 | 83,63644 |
| ENSMUSG00000091649 | Phf11b | 1,36 | 0,009999771 | 432,2362 | 858,0636 | 493,921 | 206,1874 | 131,9106 | 354,1202 |
| ENSMUSG00000068245 | Phf11d | 1,96 | 0,006366335 | 256,179 | 729,5473 | 330,434 | 79,30284 | 48,19811 | 209,0911 |
| ENSMUSG00000026873 | Phf19 | 1,00 | 2,32766E-05 | 1284,058 | 1336,376 | 1410,832 | 736,5251 | 754,6809 | 524,0624 |
| ENSMUSG00000036062 | Phf24 | 2,94 | 0,000228084 | 34,78975 | 52,17954 | 54,49567 | 3,965142 | 1,268371 | 12,45649 |
| ENSMUSG00000031295 | Phka2 | 1,27 | 3,26814E-08 | 5867,871 | 7697,449 | 5985,873 | 3060,098 | 2767,586 | 2293,774 |
| ENSMUSG00000025537 | Phkg1 | 2,81 | 0,000231082 | 21,08469 | 28,02235 | 38,92548 | 3,965142 | 6,341857 | 2,669248 |
| ENSMUSG00000020205 | Phlda1 | -1,02 | 0,011406613 | 442,7786 | 364,2905 | 410,015 | 531,329 | 748,3391 | 1195,823 |
| ENSMUSG00000010760 | Phlda2 | -2,71 | 0,037626686 | 2,108469 | 2,898863 | 1,730021 | 6,938998 | 6,341857 | 30,25148 |
| ENSMUSG00000050860 | Phospho1 | -2,76 | 5,84911E-17 | 18,97623 | 20,29204 | 16,4352 | 123,9107 | 126,8371 | 125,4547 |
| ENSMUSG00000036504 | Phpt1 | 1,01 | 0,015751138 | 91,71842 | 105,3254 | 92,55613 | 58,48584 | 54,53997 | 31,14123 |
| ENSMUSG00000003469 | Phyhip | -2,88 | 7,81925E-12 | 131,7793 | 82,13446 | 79,58097 | 404,4445 | 929,7162 | 816,7899 |
| ENSMUSG00000030329 | Pianp | -1,77 | 0,003128504 | 21,08469 | 18,35947 | 6,920085 | 52,53813 | 62,1502 | 42,70797 |
| ENSMUSG00000039361 | Picalm | -1,10 | 0,002371343 | 13962,28 | 7924,526 | 10296,22 | 21894,52 | 29615,2 | 17480,02 |
| ENSMUSG00000014444 | Piezo1 | 1,21 | 3,37359E-08 | 7988,991 | 9304,385 | 8183,865 | 4161,416 | 3872,338 | 2947,74 |
| ENSMUSG00000041482 | Piezo2 | 3,20 | 0,025063285 | 4,216939 | 42,51666 | 8,650106 | 1,982571 | 1,268371 | 2,669248 |
| ENSMUSG00000025728 | Pigq | -1,97 | 1,26681E-17 | 4656,555 | 3581,063 | 3642,56 | 16458,31 | 17358,93 | 12593,51 |
| ENSMUSG00000045625 | Pigz | -1,85 | 0,025697321 | 21,08469 | 18,35947 | 38,06047 | 62,45098 | 34,24603 | 182,3986 |
| ENSMUSG00000026447 | Pik3c2b | -2,25 | 1,47999E-14 | 500,7615 | 299,5492 | 396,1748 | 2118,377 | 1487,8 | 2063,329 |
| ENSMUSG00000024867 | Pip5k1b | -1,47 | 3,12067E-24 | 712,6627 | 716,0193 | 779,3745 | 2057,909 | 1932,998 | 2109,596 |
| ENSMUSG00000046634 | Pkd1l1 | -3,47 | 4,20988E-07 | 43,22362 | 7,730302 | 10,38013 | 267,6471 | 256,211 | 152,1471 |
| ENSMUSG00000048827 | Pkd1l3 | -1,81 | 0,008200022 | 196,0877 | 171,0329 | 177,3272 | 287,4728 | 323,4347 | 1303,483 |
| ENSMUSG00000024247 | Pkdcc | 2,78 | 1,68547E-19 | 1148,062 | 1337,342 | 1454,083 | 189,3355 | 129,3739 | 254,4683 |
| ENSMUSG00000038725 | Pkhd1l1 | -3,48 | 4,58844E-08 | 633,5951 | 131,4151 | 179,9222 | 3650,904 | 4632,092 | 2221,704 |
| ENSMUSG00000019876 | Pkib | 1,29 | 0,000518496 | 707,3915 | 875,4567 | 768,9944 | 259,7168 | 234,6487 | 468,0082 |
| ENSMUSG00000041237 | Pklr | -3,91 | 2,21022E-11 | 236,1486 | 51,21325 | 63,14577 | 1762,506 | 1718,643 | 1767,932 |
| ENSMUSG00000026785 | Pkn3 | 1,34 | 3,27675E-05 | 723,205 | 809,7492 | 642,7029 | 222,0479 | 243,5273 | 392,3795 |
| ENSMUSG00000041957 | Pkp2 | -1,87 | 0,009983827 | 8,433878 | 4,831439 | 6,055074 | 16,85185 | 26,6358 | 26,69248 |
| ENSMUSG00000002847 | Pla1a | -2,16 | 0,014974787 | 34,78975 | 9,662878 | 24,2203 | 24,78214 | 124,3004 | 156,5959 |
| ENSMUSG00000027999 | Pla2g12a | -1,92 | 6,08049E-14 | 365,8195 | 239,6394 | 378,8746 | 1210,36 | 1177,049 | 1344,411 |
| ENSMUSG00000060675 | Pla2g16 | -2,28 | 3,46281E-05 | 1487,525 | 370,0882 | 528,5215 | 3900,708 | 3793,699 | 3912,228 |
| ENSMUSG00000041202 | Pla2g2d | -3,79 | 0,000263077 | 4,216939 | 0 | 19,89524 | 108,0501 | 76,10228 | 152,1471 |
| ENSMUSG00000033847 | Pla2g4c | -1,62 | 0,003049883 | 164,4606 | 58,94356 | 58,82072 | 324,1503 | 314,5561 | 225,1066 |
| ENSMUSG00000023913 | Pla2g7 | -1,23 | 0,003555684 | 1300,926 | 1906,486 | 1162,574 | 4214,946 | 2004,027 | 4045,69 |
| ENSMUSG00000029322 | Plac8 | 1,64 | 8,2077E-20 | 53420,18 | 48416,82 | 51889,39 | 15442,24 | 19025,57 | 14735,14 |
| ENSMUSG00000097107 | Platr6 | -2,73 | 0,044127225 | 1,054235 | 1,932576 | 1,730021 | 18,83442 | 6,341857 | 6,228246 |
| ENSMUSG00000097076 | Platr7 | -1,68 | 0,002282314 | 32,68128 | 103,3928 | 64,01078 | 203,2135 | 150,9362 | 286,4993 |
| ENSMUSG00000030214 | Plbd1 | -1,42 | 2,68691E-07 | 6852,526 | 10042,63 | 6647,606 | 22228,59 | 23433,16 | 17233,56 |
| ENSMUSG00000051177 | Plcb1 | 2,77 | 1,83358E-23 | 1865,995 | 2138,395 | 2555,241 | 380,6536 | 351,3389 | 232,2246 |
| ENSMUSG00000003363 | Pld3 | -1,06 | 0,001053236 | 2692,516 | 2298,799 | 3279,255 | 4536,122 | 5053,191 | 7627,821 |
| ENSMUSG00000052160 | Pld4 | 2,37 | 3,9449E-22 | 40832,62 | 39418,74 | 43624,21 | 8314,902 | 5929,636 | 9733,858 |
| ENSMUSG00000021118 | Plek2 | -2,75 | 4,96126E-09 | 59,03715 | 17,39318 | 49,3056 | 274,5861 | 248,6008 | 322,979 |
| ENSMUSG00000040268 | Plekha1 | 1,11 | 3,43012E-07 | 3441,022 | 2659,224 | 3066,463 | 1565,24 | 1476,384 | 1204,721 |
| ENSMUSG00000002733 | Plekha3 | 1,77 | 3,71923E-19 | 3353,521 | 2954,908 | 3978,184 | 1004,172 | 1080,652 | 936,0163 |
| ENSMUSG00000041757 | Plekha6 | -2,05 | 1,38787E-05 | 54,82021 | 28,02235 | 37,19546 | 209,1612 | 98,93296 | 187,7371 |
| ENSMUSG00000026123 | Plekhb2 | 1,10 | 8,30933E-12 | 7041,234 | 5787,098 | 6204,721 | 2929,249 | 2884,276 | 3077,643 |
| ENSMUSG00000039713 | Plekhg5 | 1,55 | 1,01273E-06 | 688,4153 | 1019,434 | 1082,128 | 363,8018 | 235,9171 | 352,3407 |
| ENSMUSG00000040852 | Plekhh2 | 3,47 | 2,04648E-13 | 484,948 | 993,3439 | 853,7654 | 62,45098 | 38,05114 | 108,5494 |
| ENSMUSG00000035172 | Plekhh3 | 1,27 | 7,53595E-06 | 158,1352 | 168,1341 | 177,3272 | 74,34641 | 54,53997 | 78,29794 |
| ENSMUSG00000016495 | Plgrkt | 1,03 | 7,61079E-07 | 3103,667 | 3623,579 | 3160,749 | 1499,815 | 1447,212 | 1896,946 |
| ENSMUSG00000028494 | Plin2 | 1,24 | 0,001224927 | 14331,27 | 7040,373 | 11928,5 | 5357,898 | 5240,91 | 3480,7 |
| ENSMUSG00000038583 | Pln | -1,38 | 0,019246228 | 14,75929 | 20,29204 | 10,38013 | 29,73856 | 49,46648 | 39,14897 |
| ENSMUSG00000004846 | Plod3 | 1,27 | 0,000552567 | 8684,786 | 9468,654 | 8750,447 | 4576,765 | 4406,322 | 2141,627 |
| ENSMUSG00000031425 | Plp1 | -2,29 | 0,013036484 | 32,68128 | 61,84242 | 33,73541 | 61,4597 | 82,44414 | 482,2442 |
| ENSMUSG00000021759 | Plpp1 | -3,07 | 5,81072E-11 | 177,1114 | 48,31439 | 113,3164 | 921,8955 | 1052,748 | 859,4979 |
| ENSMUSG00000052151 | Plpp2 | 1,32 | 5,7394E-07 | 638,8663 | 463,8181 | 608,9675 | 207,1787 | 206,7445 | 272,2633 |
| ENSMUSG00000028517 | Plpp3 | -1,73 | 0,00818255 | 356,3313 | 109,1905 | 318,3239 | 385,61 | 970,3041 | 1243,87 |
| ENSMUSG00000035835 | Plppr3 | 1,97 | 1,51348E-31 | 4826,287 | 6111,77 | 5650,249 | 1448,268 | 1425,649 | 1353,309 |
| ENSMUSG00000044667 | Plppr4 | 4,37 | 0,001003008 | 10,54235 | 39,6178 | 13,84017 | 0,991285 | 1,268371 | 0,889749 |
| ENSMUSG00000049493 | Pls1 | -2,56 | 0,004436791 | 196,0877 | 120,786 | 257,7732 | 271,6122 | 431,2463 | 2687,043 |
| ENSMUSG00000032369 | Plscr1 | -1,03 | 0,001462075 | 1830,152 | 1325,747 | 1379,692 | 2673,497 | 4099,376 | 2487,739 |
| ENSMUSG00000032372 | Plscr2 | -1,95 | 0,005307371 | 15,81352 | 18,35947 | 13,84017 | 41,63399 | 32,97765 | 109,4392 |
| ENSMUSG00000032377 | Plscr4 | -1,33 | 3,84577E-08 | 87,50148 | 98,56136 | 76,12093 | 211,1438 | 221,965 | 223,3271 |
| ENSMUSG00000034845 | Plvap | -1,37 | 0,023694881 | 264,6129 | 145,9095 | 275,9384 | 335,0545 | 400,8053 | 1034,779 |
| ENSMUSG00000026640 | Plxna2 | -1,66 | 6,81582E-06 | 33,73551 | 60,87613 | 58,82072 | 132,8323 | 172,4985 | 182,3986 |
| ENSMUSG00000029765 | Plxna4 | -3,56 | 3,60097E-06 | 120,1828 | 49,28068 | 88,23108 | 370,7408 | 416,0258 | 2242,168 |
| ENSMUSG00000086763 | Plxna4os1 | -2,03 | 0,015768084 | 6,325408 | 6,764015 | 11,24514 | 24,78214 | 16,48883 | 57,83371 |
| ENSMUSG00000053646 | Plxnb1 | -1,71 | 0,023812012 | 13,70505 | 22,22462 | 17,30021 | 51,54684 | 22,83068 | 98,76218 |
| ENSMUSG00000036606 | Plxnb2 | 1,72 | 2,55256E-11 | 13599,63 | 15709,91 | 14117,84 | 4612,451 | 3235,615 | 5358,071 |
| ENSMUSG00000074785 | Plxnc1 | -1,11 | 1,07642E-05 | 3666,628 | 2822,527 | 2552,646 | 6271,863 | 7508,758 | 5672,152 |
| ENSMUSG00000030123 | Plxnd1 | 1,14 | 8,88861E-06 | 9619,892 | 10462 | 9259,938 | 5684,031 | 3952,245 | 3655,09 |
| ENSMUSG00000054659 | Pm20d2 | -2,89 | 0,003524378 | 2,108469 | 0,966288 | 3,460042 | 15,86057 | 20,29394 | 13,34624 |
| ENSMUSG00000038400 | Pmepa1 | -1,35 | 0,012843547 | 89,60995 | 39,6178 | 83,04102 | 107,0588 | 238,4538 | 195,7449 |
| ENSMUSG00000018217 | Pmp22 | -1,78 | 0,007188912 | 64,30832 | 49,28068 | 157,4319 | 147,7015 | 409,6839 | 372,805 |
| ENSMUSG00000002012 | Pnck | -3,44 | 1,25889E-05 | 23,19316 | 23,19091 | 31,14038 | 106,0675 | 107,8116 | 625,4938 |
| ENSMUSG00000054383 | Pnma1 | -2,39 | 9,47914E-11 | 20,03046 | 18,35947 | 15,57019 | 105,0763 | 93,85948 | 83,63644 |
| ENSMUSG00000018659 | Pnpo | -1,60 | 1,18003E-09 | 2518,567 | 1641,723 | 2009,42 | 6562,31 | 5114,073 | 6964,068 |
| ENSMUSG00000028600 | Podn | -2,29 | 0,000187812 | 11,59658 | 19,32576 | 22,49028 | 56,50327 | 60,88182 | 144,1394 |
| ENSMUSG00000022803 | Popdc2 | -2,57 | 1,3123E-11 | 60,09138 | 28,02235 | 55,36068 | 253,7691 | 347,5337 | 250,9093 |
| ENSMUSG00000019848 | Popdc3 | 4,81 | 8,66209E-05 | 20,03046 | 19,32576 | 46,71057 | 0 | 1,268371 | 1,779499 |
| ENSMUSG00000032053 | Pou2af1 | -1,75 | 0,014653946 | 2847,488 | 896,7151 | 2026,72 | 9362,691 | 8038,938 | 2029,518 |
| ENSMUSG00000009739 | Pou6f1 | -1,78 | 2,26131E-05 | 166,5691 | 75,37045 | 109,8563 | 375,6972 | 304,4091 | 523,1726 |
| ENSMUSG00000029167 | Ppargc1a | 3,47 | 6,00926E-18 | 238,2571 | 214,5159 | 364,1695 | 33,70371 | 21,56231 | 18,68474 |
| ENSMUSG00000029372 | Ppbp | -3,60 | 0,007795742 | 4535,318 | 2461,135 | 4179,731 | 7238,366 | 14814,58 | 113308,7 |
| ENSMUSG00000029410 | Ppef2 | 2,42 | 1,1803E-07 | 195,0334 | 120,786 | 88,23108 | 27,75599 | 25,36743 | 22,24373 |
| ENSMUSG00000026458 | Ppfia4 | 1,27 | 0,009887099 | 3612,862 | 5008,27 | 4398,579 | 1673,29 | 901,812 | 2806,27 |
| ENSMUSG00000024538 | Ppic | -1,85 | 0,013039113 | 123,3455 | 107,2579 | 173,8671 | 198,2571 | 243,5273 | 1020,543 |
| ENSMUSG00000039457 | Ppl | -1,87 | 0,031924104 | 9,488113 | 3,865151 | 6,055074 | 28,74728 | 30,44091 | 11,56674 |
| ENSMUSG00000046442 | Ppm1e | -1,49 | 9,67684E-05 | 395,338 | 268,628 | 324,379 | 1337,244 | 797,8056 | 647,7375 |
| ENSMUSG00000027784 | Ppm1l | -2,63 | 5,99295E-11 | 390,0669 | 143,9769 | 224,9028 | 1746,645 | 1565,17 | 1389,789 |
| ENSMUSG00000062729 | Ppox | -1,87 | 5,02103E-12 | 3021,437 | 2945,245 | 2176,367 | 12087,73 | 10015,06 | 7699,001 |
| ENSMUSG00000037166 | Ppp1r14a | -5,33 | 0,000355583 | 6,325408 | 0,966288 | 0,865011 | 15,86057 | 8,878599 | 297,1763 |
| ENSMUSG00000040435 | Ppp1r15a | -2,90 | 3,34552E-11 | 2415,252 | 769,1651 | 1459,273 | 13359,55 | 11416,61 | 9946,508 |
| ENSMUSG00000037754 | Ppp1r16b | -1,14 | 0,000127192 | 2308,774 | 2071,721 | 1797,492 | 5850,567 | 3392,893 | 4355,323 |
| ENSMUSG00000034709 | Ppp1r21 | 1,17 | 8,58074E-05 | 6009,138 | 6284,736 | 6517,855 | 3073,976 | 3378,941 | 1883,599 |
| ENSMUSG00000025916 | Ppp1r42 | -1,73 | 1,45336E-06 | 104,3692 | 105,3254 | 71,79588 | 350,9151 | 371,6328 | 212,6501 |
| ENSMUSG00000032827 | Ppp1r9a | -2,55 | 4,60126E-09 | 78,01337 | 44,44924 | 66,60582 | 318,2026 | 240,9906 | 548,0856 |
| ENSMUSG00000032058 | Ppp2r1b | -1,06 | 0,001042607 | 3536,958 | 2860,212 | 3067,328 | 7225,48 | 8126,455 | 4406,039 |
| ENSMUSG00000024777 | Ppp2r5b | -1,30 | 9,22464E-08 | 1133,302 | 728,581 | 843,3853 | 2147,124 | 2220,918 | 2282,207 |
| ENSMUSG00000021209 | Ppp4r4 | -2,41 | 0,002175584 | 4,216939 | 5,797727 | 2,595032 | 25,77342 | 24,09906 | 16,90524 |
| ENSMUSG00000039410 | Prdm16 | 1,55 | 0,001477819 | 223,4978 | 207,7519 | 436,8303 | 118,9543 | 62,1502 | 114,7777 |
| ENSMUSG00000029913 | Prdm5 | 1,52 | 0,0138514 | 100,1523 | 163,3026 | 125,4265 | 26,76471 | 27,90417 | 80,07744 |
| ENSMUSG00000005161 | Prdx2 | -2,53 | 1,18614E-10 | 7596,816 | 2838,954 | 4295,643 | 29578,97 | 27940,95 | 27604,47 |
| ENSMUSG00000041577 | Prelp | -1,75 | 0,015865583 | 83,28454 | 47,3481 | 149,6468 | 130,8497 | 266,358 | 545,4164 |
| ENSMUSG00000030020 | Prickle2 | -1,75 | 0,005534564 | 15,81352 | 13,52803 | 20,76025 | 39,65142 | 38,05114 | 90,75444 |
| ENSMUSG00000028518 | Prkaa2 | -3,27 | 0,000125942 | 27,4101 | 10,62917 | 6,920085 | 81,28541 | 57,07671 | 290,948 |
| ENSMUSG00000028944 | Prkag2 | 1,25 | 1,26958E-17 | 2529,109 | 2721,066 | 2854,535 | 1194,499 | 1102,215 | 1106,848 |
| ENSMUSG00000002997 | Prkar2b | -1,55 | 0,001090526 | 3455,781 | 2848,616 | 3903,793 | 6201,482 | 7019,167 | 16657,89 |
| ENSMUSG00000050965 | Prkca | -2,46 | 0,001383707 | 906,6419 | 476,3799 | 907,3961 | 1757,549 | 1561,365 | 9304,999 |
| ENSMUSG00000052889 | Prkcb | -1,42 | 8,1111E-07 | 2561,79 | 2018,575 | 1900,428 | 6649,543 | 6544,796 | 4183,602 |
| ENSMUSG00000045038 | Prkce | -1,02 | 2,47708E-07 | 474,4056 | 431,9306 | 545,8217 | 1060,675 | 908,1539 | 975,1653 |
| ENSMUSG00000021108 | Prkch | 1,34 | 0,00021021 | 2210,73 | 1752,846 | 2620,982 | 662,1787 | 709,0196 | 1225,185 |
| ENSMUSG00000026778 | Prkcq | -2,22 | 0,004373923 | 601,968 | 238,6731 | 349,4643 | 715,7081 | 862,4925 | 3971,841 |
| ENSMUSG00000024070 | Prkd3 | 1,19 | 1,65966E-05 | 11237,09 | 11490,13 | 10904,32 | 5673,127 | 5611,275 | 3445,11 |
| ENSMUSG00000002731 | Prkra | 1,28 | 0,000313581 | 1515,99 | 2560,663 | 1819,982 | 629,4663 | 731,8503 | 1066,809 |
| ENSMUSG00000079037 | Prnp | -2,20 | 1,88117E-13 | 514,4666 | 287,9538 | 400,4999 | 1949,859 | 2054,762 | 1507,235 |
| ENSMUSG00000044122 | Proca1 | -1,29 | 0,002952081 | 21,08469 | 30,92121 | 22,49028 | 70,38127 | 60,88182 | 51,60546 |
| ENSMUSG00000030069 | Prok2 | -1,99 | 0,000243025 | 26,35587 | 37,68522 | 45,84556 | 128,8671 | 79,90739 | 227,7758 |
| ENSMUSG00000049409 | Prokr1 | -2,90 | 5,71072E-15 | 110,6946 | 46,38181 | 96,88119 | 678,0393 | 539,0578 | 671,7608 |
| ENSMUSG00000050558 | Prokr2 | -4,16 | 0,044804511 | 1,054235 | 0,966288 | 0 | 0,991285 | 6,341857 | 27,58223 |
| ENSMUSG00000029086 | Prom1 | -1,20 | 0,010121993 | 927,7266 | 1234,916 | 894,4209 | 2531,743 | 3283,813 | 1199,382 |
| ENSMUSG00000045319 | Proser2 | -2,63 | 0,006186081 | 133,8878 | 59,90984 | 124,5615 | 145,719 | 219,4282 | 1597,99 |
| ENSMUSG00000045725 | Prr15 | 3,49 | 3,27362E-07 | 103,315 | 215,4822 | 105,5313 | 3,965142 | 10,14697 | 23,13348 |
| ENSMUSG00000047996 | Prrg1 | 1,28 | 1,86866E-05 | 259,3417 | 262,8303 | 232,6878 | 123,9107 | 111,6167 | 75,6287 |
| ENSMUSG00000027978 | Prss12 | -2,55 | 0,000966156 | 8,433878 | 4,831439 | 4,325053 | 54,5207 | 21,56231 | 25,80273 |
| ENSMUSG00000006179 | Prss16 | 1,94 | 1,39933E-08 | 1023,662 | 1308,354 | 983,517 | 283,5076 | 391,9267 | 189,5166 |
| ENSMUSG00000024124 | Prss30 | -1,68 | 0,022756511 | 39,00669 | 43,48295 | 50,17061 | 68,3987 | 73,56554 | 283,83 |
| ENSMUSG00000056399 | Prss34 | -1,98 | 1,23432E-06 | 2442,662 | 2535,539 | 1014,657 | 8875,97 | 6889,793 | 7848,479 |
| ENSMUSG00000048752 | Prss50 | -4,17 | 6,12588E-19 | 8,433878 | 3,865151 | 10,38013 | 148,6928 | 110,3483 | 150,3676 |
| ENSMUSG00000020323 | Prss57 | -1,15 | 0,038546082 | 943,5401 | 717,9518 | 740,4491 | 1866,591 | 2715,583 | 754,5075 |
| ENSMUSG00000039126 | Prune2 | 1,44 | 0,047874897 | 550,3105 | 673,5026 | 704,1186 | 94,17212 | 133,179 | 483,1339 |
| ENSMUSG00000004207 | Psap | 1,25 | 7,01843E-11 | 225919,3 | 223523,6 | 268238,9 | 95700,68 | 90727,87 | 114321,2 |
| ENSMUSG00000031897 | Psmb10 | 1,09 | 3,0793E-06 | 7927,845 | 5663,413 | 6209,046 | 2927,266 | 2838,615 | 3526,077 |
| ENSMUSG00000078652 | Psme3 | -1,02 | 2,91534E-07 | 8473,939 | 6987,227 | 7726,275 | 17070,93 | 16507,85 | 13389,84 |
| ENSMUSG00000032869 | Psmf1 | -1,19 | 2,11272E-11 | 2003,046 | 1556,69 | 1813,927 | 4186,199 | 4166,6 | 3903,33 |
| ENSMUSG00000025495 | Ptdss2 | -2,17 | 1,07873E-14 | 1542,345 | 925,7037 | 1045,798 | 5396,558 | 5915,684 | 4466,542 |
| ENSMUSG00000071489 | Ptgdr | 2,22 | 0,00281148 | 117,0201 | 456,0878 | 130,6166 | 46,59042 | 78,63902 | 26,69248 |
| ENSMUSG00000037759 | Ptger2 | 2,08 | 1,65698E-20 | 3103,667 | 2449,54 | 2768,034 | 674,0741 | 757,2177 | 532,9599 |
| ENSMUSG00000040016 | Ptger3 | -2,20 | 0,014342328 | 57,98291 | 39,6178 | 110,7214 | 110,0327 | 129,3739 | 718,0277 |
| ENSMUSG00000028036 | Ptgfr | -1,51 | 0,021120718 | 21,08469 | 19,32576 | 19,89524 | 26,76471 | 57,07671 | 87,19544 |
| ENSMUSG00000027864 | Ptgfrn | 1,72 | 0,00088159 | 336,3009 | 617,4579 | 1178,144 | 233,9434 | 191,5241 | 220,6578 |
| ENSMUSG00000097754 | Ptgs2os2 | -2,09 | 0,000109709 | 29,51857 | 14,49432 | 15,57019 | 96,15469 | 104,0064 | 52,49521 |
| ENSMUSG00000022607 | Ptk2 | -1,81 | 0,004140318 | 115,9658 | 132,3814 | 181,6522 | 273,5948 | 273,9682 | 963,5986 |
| ENSMUSG00000059895 | Ptp4a3 | -1,95 | 8,33504E-24 | 3389,365 | 3005,155 | 3333,751 | 14572,89 | 10697,44 | 12315,02 |
| ENSMUSG00000034573 | Ptpn13 | -2,10 | 8,64837E-14 | 98,04383 | 61,84242 | 76,12093 | 313,2462 | 384,3165 | 310,5225 |
| ENSMUSG00000026604 | Ptpn14 | -1,77 | 0,000114005 | 48,4948 | 53,14583 | 98,61121 | 189,3355 | 180,1087 | 314,9713 |
| ENSMUSG00000038764 | Ptpn3 | -2,32 | 0,000163179 | 121,237 | 109,1905 | 76,98594 | 306,3072 | 248,6008 | 980,5038 |
| ENSMUSG00000045826 | Ptprcap | -1,68 | 7,44627E-08 | 1225,021 | 694,7609 | 1204,095 | 3745,076 | 2803,101 | 3458,456 |
| ENSMUSG00000028399 | Ptprd | -1,29 | 0,002323029 | 94,88113 | 70,53901 | 105,5313 | 140,7625 | 227,0385 | 293,6173 |
| ENSMUSG00000021745 | Ptprg | -1,20 | 0,023339373 | 26,35587 | 28,98863 | 45,84556 | 51,54684 | 78,63902 | 103,2109 |
| ENSMUSG00000026204 | Ptprn | -3,50 | 0,004974117 | 1,054235 | 1,932576 | 15,57019 | 27,75599 | 27,90417 | 157,4856 |
| ENSMUSG00000030223 | Ptpro | 2,49 | 1,92728E-08 | 4461,521 | 7116,71 | 4965,161 | 1508,736 | 523,8374 | 904,8751 |
| ENSMUSG00000020151 | Ptprr | -2,66 | 0,007921277 | 14,75929 | 3,865151 | 2,595032 | 31,72113 | 81,17577 | 20,46424 |
| ENSMUSG00000040511 | Pvr | 1,30 | 6,78331E-05 | 883,4487 | 524,6943 | 647,0279 | 222,0479 | 284,1152 | 329,2073 |
| ENSMUSG00000097039 | Pvt1 | -1,59 | 2,57275E-06 | 50,60327 | 55,0784 | 42,38552 | 113,0065 | 159,8148 | 172,6114 |
| ENSMUSG00000021411 | Pxdc1 | -2,24 | 1,67538E-05 | 79,06761 | 28,02235 | 108,1263 | 333,0719 | 294,2622 | 387,041 |
| ENSMUSG00000043587 | Pxylp1 | 1,45 | 0,000816976 | 7943,659 | 6185,208 | 9265,993 | 3803,562 | 3269,861 | 1513,464 |
| ENSMUSG00000030793 | Pycard | 1,19 | 4,65511E-10 | 5809,888 | 4698,091 | 5619,974 | 2138,203 | 2617,918 | 2321,356 |
| ENSMUSG00000025140 | Pycr1 | 2,80 | 2,25501E-22 | 998,3603 | 1228,152 | 1198,905 | 112,0153 | 183,9138 | 197,5244 |
| ENSMUSG00000060224 | Pyroxd2 | 1,59 | 1,20083E-05 | 743,2355 | 1315,118 | 914,3162 | 298,3769 | 253,6743 | 435,0874 |
| ENSMUSG00000024084 | Qpct | 3,97 | 6,66504E-69 | 1275,624 | 1354,735 | 1530,204 | 99,12855 | 96,39622 | 70,2902 |
| ENSMUSG00000059277 | R74862 | -1,95 | 1,21501E-08 | 31,62704 | 23,19091 | 32,00539 | 104,085 | 102,7381 | 128,1239 |
| ENSMUSG00000031488 | Rab11fip1 | -1,04 | 0,000262525 | 2095,819 | 1836,913 | 1732,616 | 4694,728 | 4094,303 | 2824,064 |
| ENSMUSG00000031504 | Rab20 | -1,00 | 0,004279402 | 238,2571 | 200,0216 | 285,4535 | 346,9499 | 620,2336 | 486,6929 |
| ENSMUSG00000024511 | Rab27b | -3,41 | 0,008355029 | 421,6939 | 229,9765 | 428,1802 | 818,8018 | 1132,656 | 9510,531 |
| ENSMUSG00000030643 | Rab30 | -2,80 | 7,90238E-11 | 72,7422 | 28,02235 | 32,8704 | 352,8976 | 281,5784 | 289,1685 |
| ENSMUSG00000020732 | Rab37 | -1,57 | 0,000970402 | 560,8529 | 659,9746 | 656,543 | 1184,586 | 1255,688 | 3149,713 |
| ENSMUSG00000021700 | Rab3c | 9,26 | 0,005431397 | 6,325408 | 28,98863 | 34,60042 | 0 | 0 | 0 |
| ENSMUSG00000024663 | Rab3il1 | -2,94 | 5,52122E-09 | 1586,623 | 405,8409 | 736,124 | 7566,482 | 7501,148 | 5928,4 |
| ENSMUSG00000019478 | Rab4a | -2,37 | 1,61227E-10 | 49,54903 | 49,28068 | 78,71596 | 243,8562 | 267,6264 | 409,2847 |
| ENSMUSG00000032549 | Rab6b | -2,02 | 0,0024367 | 29,51857 | 14,49432 | 16,4352 | 45,59913 | 60,88182 | 137,0214 |
| ENSMUSG00000052688 | Rab7b | 1,34 | 0,000659984 | 1239,78 | 769,1651 | 1007,737 | 283,5076 | 569,4987 | 338,9945 |
| ENSMUSG00000003813 | Rad23a | -1,66 | 1,09531E-13 | 3288,158 | 2456,304 | 3009,372 | 10243,94 | 9789,29 | 7702,56 |
| ENSMUSG00000043518 | Rai2 | -2,76 | 0,001055396 | 20,03046 | 14,49432 | 17,30021 | 45,59913 | 48,19811 | 256,2478 |
| ENSMUSG00000026594 | Ralgps2 | -2,02 | 3,37018E-10 | 988,8722 | 522,7617 | 680,7633 | 3374,336 | 2395,953 | 3106,115 |
| ENSMUSG00000034353 | Ramp1 | 1,02 | 8,1714E-07 | 4516,342 | 4096,094 | 5269,644 | 2000,414 | 2316,046 | 2512,652 |
| ENSMUSG00000037415 | Ranbp10 | -2,10 | 2,34276E-15 | 5416,658 | 3597,489 | 3856,217 | 21536,67 | 18322,89 | 15225,39 |
| ENSMUSG00000049044 | Rapgef4 | 2,01 | 0,000819641 | 189,7623 | 450,2901 | 413,4751 | 102,1024 | 35,5144 | 123,6752 |
| ENSMUSG00000085439 | Rapgef4os1 | 7,68 | 0,032037383 | 3,162704 | 12,56174 | 7,785095 | 0 | 0 | 0 |
| ENSMUSG00000041992 | Rapgef5 | -1,06 | 0,03164863 | 90,66419 | 50,24697 | 102,9363 | 174,4662 | 220,6966 | 115,6674 |
| ENSMUSG00000026014 | Raph1 | 2,00 | 1,52791E-10 | 631,4866 | 936,3329 | 744,7741 | 247,8214 | 185,1822 | 144,1394 |
| ENSMUSG00000001288 | Rarg | 1,32 | 0,000622817 | 4096,756 | 4342,497 | 4334,568 | 1581,1 | 1034,991 | 2487,739 |
| ENSMUSG00000032413 | Rasa2 | -1,04 | 0,01778858 | 2232,869 | 1624,33 | 1605,46 | 3077,941 | 5683,572 | 2472,613 |
| ENSMUSG00000004952 | Rasa4 | 2,49 | 4,0984E-13 | 8461,288 | 10669,75 | 8390,603 | 1609,848 | 1028,649 | 2266,192 |
| ENSMUSG00000029602 | Rasal1 | -1,48 | 8,62009E-06 | 72,7422 | 54,11212 | 67,47083 | 229,9782 | 148,3994 | 164,6036 |
| ENSMUSG00000020374 | Rasgef1c | -2,04 | 0,013422398 | 7,379643 | 2,898863 | 3,460042 | 17,84314 | 20,29394 | 17,79499 |
| ENSMUSG00000032356 | Rasgrf1 | 2,63 | 2,6018E-05 | 41,11515 | 118,8534 | 57,0907 | 12,88671 | 11,41534 | 10,67699 |
| ENSMUSG00000021708 | Rasgrf2 | 2,38 | 5,53097E-08 | 425,9108 | 374,9197 | 474,0258 | 83,26798 | 39,31951 | 121,0059 |
| ENSMUSG00000027347 | Rasgrp1 | -1,45 | 0,000388805 | 659,9509 | 279,2572 | 367,6295 | 1361,035 | 1008,355 | 1202,051 |
| ENSMUSG00000044562 | Rasip1 | -1,47 | 0,000235288 | 76,95914 | 79,2356 | 63,14577 | 130,8497 | 275,2366 | 203,7526 |
| ENSMUSG00000034209 | Rasl10a | -4,28 | 6,80481E-06 | 13,70505 | 5,797727 | 3,460042 | 71,37255 | 40,58788 | 329,2073 |
| ENSMUSG00000029641 | Rasl11a | 3,58 | 3,91953E-09 | 202,4131 | 215,4822 | 229,2278 | 8,921569 | 7,610228 | 36,47972 |
| ENSMUSG00000049907 | Rasl11b | -1,22 | 0,002660574 | 417,477 | 193,2576 | 333,8941 | 740,4902 | 896,7385 | 570,3293 |
| ENSMUSG00000042129 | Rassf4 | 2,28 | 2,19606E-25 | 36881,35 | 37177,92 | 36412,62 | 8889,848 | 7945,078 | 5856,33 |
| ENSMUSG00000029370 | Rassf6 | 4,07 | 9,28484E-16 | 300,4569 | 807,8166 | 409,15 | 25,77342 | 20,29394 | 43,59772 |
| ENSMUSG00000022105 | Rb1 | -1,09 | 3,58453E-05 | 3056,227 | 3790,747 | 4050,845 | 8632,114 | 8552,628 | 5963,1 |
| ENSMUSG00000032940 | Rbm11 | 3,67 | 2,17929E-20 | 157,081 | 242,5382 | 228,3628 | 15,86057 | 11,41534 | 21,35398 |
| ENSMUSG00000027510 | Rbm38 | -1,32 | 6,41634E-11 | 5218,462 | 3986,903 | 4605,316 | 12811,37 | 10256,05 | 11472,43 |
| ENSMUSG00000032387 | Rbpms2 | -3,13 | 0,000487413 | 191,8707 | 182,6284 | 189,4373 | 367,7669 | 492,1281 | 4065,265 |
| ENSMUSG00000079575 | Rbpsuh-rs3 | 1,05 | 0,01778858 | 248,7994 | 311,1447 | 538,0366 | 152,658 | 218,1599 | 161,9344 |
| ENSMUSG00000024968 | Rcor2 | -2,29 | 0,00013634 | 79,06761 | 66,67386 | 86,50106 | 227,9957 | 190,2557 | 718,0277 |
| ENSMUSG00000021123 | Rdh12 | -1,52 | 0,004281999 | 1972,473 | 1380,825 | 1452,353 | 3821,405 | 7729,455 | 2214,586 |
| ENSMUSG00000028476 | Reck | -1,47 | 7,40425E-09 | 277,2637 | 241,572 | 248,258 | 867,3748 | 566,962 | 684,2173 |
| ENSMUSG00000096982 | Redrum | -3,02 | 0,030344763 | 286,7518 | 13,52803 | 44,98055 | 1023,998 | 972,8408 | 805,2232 |
| ENSMUSG00000052852 | Reep1 | -1,38 | 0,019961732 | 31,62704 | 22,22462 | 17,30021 | 48,57299 | 43,12463 | 91,64418 |
| ENSMUSG00000038555 | Reep2 | -3,60 | 2,21464E-05 | 50,60327 | 50,24697 | 36,33044 | 183,3878 | 154,7413 | 1318,609 |
| ENSMUSG00000035504 | Reep6 | -2,41 | 2,8258E-13 | 59,03715 | 44,44924 | 54,49567 | 247,8214 | 224,5017 | 367,4665 |
| ENSMUSG00000042453 | Reln | -2,49 | 0,000839671 | 467,026 | 113,0557 | 211,0626 | 2232,375 | 1747,816 | 452,8824 |
| ENSMUSG00000000359 | Rem1 | 2,46 | 3,34695E-07 | 218,2266 | 223,2125 | 329,569 | 71,37255 | 45,66137 | 23,13348 |
| ENSMUSG00000040855 | Reps2 | -1,03 | 0,010915865 | 197,1419 | 124,6511 | 151,3769 | 325,1416 | 421,0993 | 219,7681 |
| ENSMUSG00000012705 | Retn | -1,23 | 0,01966053 | 15,81352 | 28,98863 | 20,76025 | 60,46841 | 55,80834 | 38,25922 |
| ENSMUSG00000022651 | Retnlg | -2,40 | 8,46759E-22 | 17231,47 | 14503,98 | 12840,22 | 71528,19 | 98485,23 | 65584,32 |
| ENSMUSG00000022270 | Retreg1 | -1,12 | 6,53777E-07 | 775,9168 | 1097,703 | 847,7104 | 2128,29 | 1954,56 | 1838,222 |
| ENSMUSG00000020846 | Rflnb | -1,21 | 1,16117E-05 | 4258,054 | 5424,74 | 4172,811 | 12274,1 | 11727,36 | 7964,147 |
| ENSMUSG00000025978 | Rftn2 | -1,05 | 0,002150206 | 84,33878 | 89,86477 | 103,8013 | 247,8214 | 178,8404 | 150,3676 |
| ENSMUSG00000024206 | Rfx2 | -1,79 | 1,0062E-05 | 726,3677 | 384,5825 | 417,8001 | 2227,418 | 1879,726 | 1163,792 |
| ENSMUSG00000026482 | Rgl1 | -1,81 | 6,14391E-05 | 500,7615 | 359,4591 | 570,907 | 1128,083 | 1201,148 | 2675,476 |
| ENSMUSG00000048027 | Rgmb | -1,28 | 0,044362155 | 64,30832 | 20,29204 | 43,25053 | 94,17212 | 71,02879 | 143,2496 |
| ENSMUSG00000026358 | Rgs1 | -1,85 | 0,03118096 | 173,9487 | 84,06704 | 155,7019 | 97,14598 | 433,783 | 960,0396 |
| ENSMUSG00000029101 | Rgs12 | -1,63 | 8,76182E-17 | 1064,777 | 797,1874 | 901,341 | 2700,262 | 3113,852 | 2760,003 |
| ENSMUSG00000026475 | Rgs16 | 1,42 | 0,02642478 | 177,1114 | 61,84242 | 70,93087 | 27,75599 | 41,85625 | 46,26697 |
| ENSMUSG00000026527 | Rgs7 | -7,35 | 0,04549579 | 0 | 0 | 0 | 5,947713 | 8,878599 | 2,669248 |
| ENSMUSG00000042671 | Rgs8 | 4,30 | 4,10295E-23 | 1007,848 | 491,8405 | 658,2731 | 54,5207 | 24,09906 | 30,25148 |
| ENSMUSG00000056043 | Rgs9bp | -2,28 | 4,80547E-07 | 14,75929 | 20,29204 | 26,81533 | 105,0763 | 126,8371 | 71,17995 |
| ENSMUSG00000023926 | Rhag | -3,34 | 1,55732E-07 | 1816,446 | 284,0886 | 657,408 | 9207,059 | 11121,08 | 7533,508 |
| ENSMUSG00000030549 | Rhcg | -2,76 | 0,002142064 | 5,271174 | 8,69659 | 13,84017 | 57,49456 | 115,4218 | 16,90524 |
| ENSMUSG00000028825 | Rhd | -3,53 | 1,02675E-07 | 2763,149 | 396,178 | 862,4156 | 17095,71 | 16908,66 | 12375,52 |
| ENSMUSG00000021589 | Rhobtb3 | -2,92 | 1,00538E-06 | 63,25408 | 51,21325 | 30,27537 | 202,2222 | 224,5017 | 666,4223 |
| ENSMUSG00000002233 | Rhoc | -1,79 | 7,59702E-06 | 126,5082 | 114,022 | 173,8671 | 339,0196 | 398,2686 | 694,0045 |
| ENSMUSG00000041845 | Rhod | 1,44 | 0,038226651 | 68,52526 | 37,68522 | 63,14577 | 16,85185 | 10,14697 | 34,70023 |
| ENSMUSG00000029449 | Rhof | -1,57 | 7,60013E-05 | 914,0215 | 563,3458 | 554,4718 | 1825,948 | 1391,403 | 2812,498 |
| ENSMUSG00000029204 | Rhoh | -1,18 | 0,001146556 | 1173,363 | 658,042 | 1023,308 | 2737,93 | 2118,18 | 1627,352 |
| ENSMUSG00000097451 | Rian | 6,10 | 7,96978E-18 | 140,2132 | 601,031 | 288,9135 | 5,947713 | 2,536743 | 6,228246 |
| ENSMUSG00000022323 | Rida | -1,63 | 0,024711832 | 556,6359 | 415,5038 | 498,2461 | 713,7255 | 663,3582 | 3164,839 |
| ENSMUSG00000028139 | Riiad1 | 1,64 | 0,045529272 | 16,86776 | 43,48295 | 23,35529 | 8,921569 | 5,073485 | 12,45649 |
| ENSMUSG00000074577 | Ripor3 | -4,12 | 2,33932E-07 | 56,92868 | 42,51666 | 35,46543 | 351,9063 | 195,3292 | 1787,506 |
| ENSMUSG00000068407 | Rnase12 | -4,73 | 0,01744878 | 1,054235 | 0,966288 | 0 | 1,982571 | 5,073485 | 44,48747 |
| ENSMUSG00000021876 | Rnase4 | 1,15 | 0,012538435 | 843,3878 | 1589,543 | 1197,175 | 491,6776 | 356,4123 | 790,9872 |
| ENSMUSG00000041740 | Rnf10 | -1,02 | 6,98568E-07 | 15962,17 | 13181,13 | 15610,85 | 34396,61 | 30475,16 | 26060,76 |
| ENSMUSG00000028557 | Rnf11 | -1,46 | 1,41788E-14 | 2785,288 | 2328,754 | 2789,659 | 6791,297 | 6676,707 | 8208,828 |
| ENSMUSG00000041528 | Rnf123 | -1,78 | 3,35725E-14 | 3422,046 | 2514,281 | 2452,305 | 11015,16 | 9384,68 | 8474,863 |
| ENSMUSG00000033107 | Rnf125 | -1,02 | 0,023347228 | 315,2162 | 205,8193 | 271,6133 | 425,2615 | 801,6107 | 378,1435 |
| ENSMUSG00000047747 | Rnf150 | 1,98 | 0,000382639 | 1114,326 | 1703,565 | 1687,636 | 234,9347 | 211,818 | 696,6738 |
| ENSMUSG00000047496 | Rnf152 | 3,45 | 2,62862E-09 | 60,09138 | 179,7295 | 134,9417 | 8,921569 | 13,95208 | 11,56674 |
| ENSMUSG00000025427 | Rnf165 | -3,84 | 0,024926196 | 2,108469 | 0 | 7,785095 | 5,947713 | 7,610228 | 129,9034 |
| ENSMUSG00000021720 | Rnf180 | -1,88 | 0,048817123 | 29,51857 | 43,48295 | 35,46543 | 39,65142 | 53,2716 | 305,184 |
| ENSMUSG00000022280 | Rnf19a | -1,00 | 0,000294004 | 1911,328 | 1679,408 | 1582,969 | 3593,41 | 4198,309 | 2574,045 |
| ENSMUSG00000070327 | Rnf213 | 1,19 | 3,95437E-07 | 28944,01 | 37528,69 | 25343,95 | 14751,32 | 12701,47 | 12808,83 |
| ENSMUSG00000063760 | Rnf217 | 2,08 | 5,43448E-10 | 464,9175 | 484,1102 | 593,3973 | 139,7713 | 149,6678 | 77,40819 |
| ENSMUSG00000071573 | Rnls | 1,11 | 0,002267772 | 149,7013 | 238,6731 | 194,6274 | 70,38127 | 110,3483 | 90,75444 |
| ENSMUSG00000052516 | Robo2 | 1,83 | 0,019983039 | 16,86776 | 33,82007 | 32,00539 | 3,965142 | 7,610228 | 11,56674 |
| ENSMUSG00000032128 | Robo3 | -3,00 | 0,000158108 | 44,27786 | 41,55038 | 51,90064 | 144,7277 | 125,5688 | 827,4669 |
| ENSMUSG00000032125 | Robo4 | -1,65 | 0,000404052 | 101,2065 | 64,74128 | 91,69112 | 168,5185 | 229,5752 | 411,0642 |
| ENSMUSG00000035305 | Ror1 | -2,08 | 0,047883514 | 3,162704 | 1,932576 | 4,325053 | 7,930284 | 19,02557 | 13,34624 |
| ENSMUSG00000057132 | Rpgrip1 | -1,48 | 0,042687796 | 82,23031 | 140,1117 | 115,9114 | 205,1961 | 124,3004 | 612,1476 |
| ENSMUSG00000020847 | Rph3al | -2,03 | 3,83684E-09 | 67,47102 | 32,85379 | 53,63066 | 214,1177 | 219,4282 | 195,7449 |
| ENSMUSG00000002500 | Rpl3l | 1,93 | 0,006174946 | 83,28454 | 118,8534 | 308,8088 | 60,46841 | 22,83068 | 50,71571 |
| ENSMUSG00000047676 | Rpsa-ps10 | 4,03 | 0,02646093 | 13549,02 | 321,7738 | 441,1554 | 299,3682 | 249,8692 | 328,3175 |
| ENSMUSG00000028278 | Rragd | -1,90 | 2,47411E-09 | 147,5929 | 84,06704 | 139,2667 | 525,3813 | 479,4444 | 384,3717 |
| ENSMUSG00000055723 | Rras2 | -1,35 | 0,005028602 | 247,7452 | 119,8197 | 225,7678 | 519,4336 | 308,2142 | 685,107 |
| ENSMUSG00000020649 | Rrm2 | -1,09 | 0,000165602 | 8790,209 | 6572,69 | 8541,115 | 18922,65 | 19440,33 | 12473,4 |
| ENSMUSG00000020641 | Rsad2 | -2,92 | 7,50958E-17 | 4350,827 | 2263,046 | 2364,074 | 17367,32 | 25837,99 | 24857,82 |
| ENSMUSG00000032215 | Rsl24d1 | 1,23 | 2,09966E-11 | 2619,773 | 2504,618 | 2939,306 | 1003,181 | 1246,809 | 1180,697 |
| ENSMUSG00000024033 | Rsph1 | -1,48 | 0,008316193 | 75,9049 | 35,75265 | 57,0907 | 224,0305 | 159,8148 | 85,41594 |
| ENSMUSG00000075569 | Rsph10b | -2,56 | 4,11335E-05 | 18,97623 | 13,52803 | 4,325053 | 60,46841 | 60,88182 | 92,53393 |
| ENSMUSG00000028871 | Rspo1 | 2,11 | 1,95392E-09 | 95,93536 | 89,86477 | 95,15116 | 23,79085 | 17,7572 | 23,13348 |
| ENSMUSG00000051920 | Rspo2 | -5,32 | 0,000132688 | 0 | 2,898863 | 1,730021 | 19,82571 | 11,41534 | 156,5959 |
| ENSMUSG00000021087 | Rtn1 | -2,30 | 0,00016634 | 11,59658 | 4,831439 | 10,38013 | 58,48584 | 36,78277 | 36,47972 |
| ENSMUSG00000045287 | Rtn4rl1 | -1,05 | 0,027091324 | 146,5386 | 111,1231 | 131,4816 | 211,1438 | 178,8404 | 414,6232 |
| ENSMUSG00000061815 | Rufy4 | -1,30 | 2,09583E-08 | 120,1828 | 150,7409 | 117,6414 | 346,9499 | 304,4091 | 306,9635 |
| ENSMUSG00000006575 | Rundc3a | -1,89 | 3,76316E-13 | 95,93536 | 76,33674 | 89,9611 | 367,7669 | 339,9235 | 267,8146 |
| ENSMUSG00000006586 | Runx1t1 | -3,97 | 1,32248E-08 | 6,325408 | 4,831439 | 5,190064 | 122,9194 | 97,66459 | 35,58997 |
| ENSMUSG00000035969 | Rusc2 | 1,74 | 0,0024367 | 595,6426 | 620,3568 | 799,2698 | 102,1024 | 131,9106 | 369,246 |
| ENSMUSG00000032417 | Rwdd2a | 1,34 | 0,000333707 | 156,0267 | 168,1341 | 196,3574 | 89,21569 | 44,393 | 71,17995 |
| ENSMUSG00000034009 | Rxfp1 | -3,53 | 0,02524089 | 0 | 0,966288 | 1,730021 | 19,82571 | 6,341857 | 6,228246 |
| ENSMUSG00000032547 | Ryk | -2,00 | 4,04107E-10 | 93,82689 | 56,04469 | 83,90603 | 277,5599 | 277,7733 | 377,2537 |
| ENSMUSG00000021313 | Ryr2 | -3,05 | 0,000957476 | 4,216939 | 0 | 6,920085 | 32,71242 | 24,09906 | 36,47972 |
| ENSMUSG00000001020 | S100a4 | 1,83 | 3,38955E-07 | 7332,203 | 12395,54 | 13323,76 | 3753,998 | 3380,21 | 2184,335 |
| ENSMUSG00000056054 | S100a8 | -1,53 | 2,8994E-09 | 172371,6 | 255498,1 | 178540,8 | 572811,3 | 667641,5 | 507014,8 |
| ENSMUSG00000056071 | S100a9 | -1,56 | 1,95988E-09 | 219087,9 | 295277,3 | 214821,9 | 691768,6 | 863172,4 | 588095 |
| ENSMUSG00000045092 | S1pr1 | -2,18 | 7,4202E-08 | 426,9651 | 168,1341 | 382,3347 | 1742,68 | 1324,18 | 1372,883 |
| ENSMUSG00000043895 | S1pr2 | 1,24 | 1,08643E-10 | 1777,44 | 1943,205 | 1513,769 | 695,8824 | 724,24 | 798,1052 |
| ENSMUSG00000067586 | S1pr3 | 1,44 | 0,002712383 | 1585,569 | 1021,366 | 1820,847 | 313,2462 | 834,5883 | 483,1339 |
| ENSMUSG00000044199 | S1pr4 | -1,12 | 6,73433E-08 | 2011,48 | 2107,474 | 1794,897 | 4946,514 | 3633,884 | 4287,702 |
| ENSMUSG00000045087 | S1pr5 | -2,19 | 7,6562E-07 | 24,2474 | 17,39318 | 25,08531 | 135,8061 | 71,02879 | 97,87243 |
| ENSMUSG00000040026 | Saa3 | 3,48 | 4,33661E-05 | 347,8975 | 143,9769 | 429,9103 | 2,973856 | 34,24603 | 45,37722 |
| ENSMUSG00000096351 | Samd11 | -3,22 | 2,03345E-07 | 468,0802 | 102,4265 | 140,9967 | 2270,044 | 2907,107 | 1452,071 |
| ENSMUSG00000047181 | Samd14 | -3,21 | 3,29095E-12 | 1048,964 | 384,5825 | 614,1575 | 4884,063 | 4902,255 | 9136,836 |
| ENSMUSG00000021838 | Samd4 | -3,68 | 2,25269E-11 | 37,95245 | 7,730302 | 41,52051 | 425,2615 | 344,997 | 350,5613 |
| ENSMUSG00000051860 | Samd7 | -3,26 | 0,004650151 | 3,162704 | 0,966288 | 0,865011 | 18,83442 | 19,02557 | 8,897494 |
| ENSMUSG00000036185 | Sapcd1 | -1,30 | 0,017276759 | 13,70505 | 23,19091 | 19,03023 | 53,52942 | 31,70928 | 52,49521 |
| ENSMUSG00000009614 | Sardh | -3,04 | 0,000166145 | 27,4101 | 22,22462 | 33,73541 | 64,43356 | 121,7636 | 500,0391 |
| ENSMUSG00000050132 | Sarm1 | 1,63 | 0,005841576 | 39,00669 | 29,95492 | 44,11554 | 16,85185 | 7,610228 | 11,56674 |
| ENSMUSG00000015305 | Sash1 | 2,42 | 6,44907E-08 | 2552,302 | 4776,361 | 4222,117 | 549,1721 | 470,5658 | 1133,541 |
| ENSMUSG00000023927 | Satb1 | -1,39 | 2,24626E-06 | 1688,884 | 1009,771 | 1735,211 | 4186,199 | 3981,418 | 3485,148 |
| ENSMUSG00000038331 | Satb2 | 1,11 | 0,018331234 | 98,04383 | 208,7182 | 139,2667 | 82,27669 | 72,29717 | 52,49521 |
| ENSMUSG00000038371 | Sbf2 | -1,26 | 0,002850706 | 220,3351 | 161,3701 | 228,3628 | 386,6013 | 346,2654 | 724,256 |
| ENSMUSG00000042978 | Sbk1 | -1,22 | 0,012094175 | 1615,088 | 1076,445 | 1479,168 | 4897,941 | 3150,634 | 1685,185 |
| ENSMUSG00000040722 | Scamp5 | -3,73 | 1,79577E-06 | 88,55572 | 38,65151 | 79,58097 | 365,7843 | 318,3612 | 2063,329 |
| ENSMUSG00000034463 | Scara3 | -1,63 | 0,012553353 | 44,27786 | 42,51666 | 83,04102 | 77,32027 | 161,0832 | 288,2788 |
| ENSMUSG00000022032 | Scara5 | -2,79 | 0,000355583 | 24,2474 | 9,662878 | 30,27537 | 39,65142 | 200,4027 | 205,5321 |
| ENSMUSG00000037936 | Scarb1 | 1,01 | 1,68477E-05 | 7470,307 | 8361,288 | 8764,287 | 3689,565 | 3533,683 | 5011,068 |
| ENSMUSG00000037071 | Scd1 | -2,44 | 3,98512E-09 | 1393,698 | 584,6041 | 1175,549 | 6386,852 | 6769,298 | 3917,566 |
| ENSMUSG00000024653 | Scgb1a1 | 5,39 | 0,005658695 | 13,70505 | 9,662878 | 20,76025 | 0 | 1,268371 | 0 |
| ENSMUSG00000027777 | Schip1 | -1,80 | 0,002262099 | 47,44056 | 46,38181 | 70,93087 | 77,32027 | 223,2334 | 274,0428 |
| ENSMUSG00000057135 | Scimp | -1,34 | 0,015092432 | 128,6166 | 68,60643 | 58,82072 | 160,5882 | 158,5464 | 324,7585 |
| ENSMUSG00000002565 | Scin | -1,12 | 0,034445071 | 75,9049 | 90,83105 | 70,93087 | 124,902 | 112,885 | 278,4916 |
| ENSMUSG00000044770 | Scml4 | -1,64 | 0,000141574 | 216,1181 | 88,89848 | 203,2775 | 602,7016 | 422,3677 | 553,4241 |
| ENSMUSG00000019194 | Scn1b | -1,69 | 0,000476241 | 40,06092 | 26,08977 | 41,52051 | 91,19826 | 84,98088 | 169,9421 |
| ENSMUSG00000057182 | Scn3a | -4,66 | 0,006559759 | 1,054235 | 0 | 1,730021 | 2,973856 | 10,14697 | 57,83371 |
| ENSMUSG00000001027 | Scn4a | -2,04 | 0,000574028 | 96,9896 | 51,21325 | 90,82611 | 561,0676 | 265,0896 | 154,8164 |
| ENSMUSG00000046480 | Scn4b | -2,00 | 0,011279258 | 111,7489 | 29,95492 | 80,44598 | 497,6253 | 300,604 | 92,53393 |
| ENSMUSG00000030340 | Scnn1a | -1,04 | 0,006066284 | 669,4391 | 782,6931 | 630,5927 | 1905,251 | 1486,531 | 887,9699 |
| ENSMUSG00000063253 | Scoc | -1,20 | 3,38644E-07 | 467,026 | 337,2344 | 358,1144 | 800,9587 | 881,5181 | 977,8346 |
| ENSMUSG00000028603 | Scp2 | 1,45 | 1,73798E-12 | 12698,26 | 14415,08 | 15359,13 | 5286,525 | 5899,195 | 4339,308 |
| ENSMUSG00000058492 | Scp2-ps2 | 1,53 | 6,6514E-08 | 290,9688 | 274,4257 | 345,1392 | 95,1634 | 133,179 | 88,08519 |
| ENSMUSG00000000278 | Scpep1 | 1,23 | 9,02617E-07 | 6845,146 | 4740,608 | 4867,415 | 2297,8 | 2094,081 | 2636,327 |
| ENSMUSG00000031610 | Scrg1 | -3,22 | 6,08413E-38 | 57,98291 | 57,01098 | 61,41575 | 510,512 | 475,6393 | 653,076 |
| ENSMUSG00000008226 | Scrn3 | -1,61 | 6,49609E-09 | 769,5914 | 597,1659 | 553,6068 | 1981,58 | 2360,439 | 1507,235 |
| ENSMUSG00000016763 | Scube1 | -2,33 | 0,003329542 | 3,162704 | 4,831439 | 3,460042 | 20,81699 | 19,02557 | 17,79499 |
| ENSMUSG00000020592 | Sdc1 | 1,41 | 8,43418E-06 | 2599,743 | 4471,014 | 3284,445 | 1118,17 | 1612,1 | 1166,461 |
| ENSMUSG00000022261 | Sdc2 | -2,07 | 0,006135765 | 62,19985 | 42,51666 | 83,04102 | 74,34641 | 219,4282 | 492,0314 |
| ENSMUSG00000017009 | Sdc4 | -1,45 | 0,022649802 | 927,7266 | 232,8754 | 880,5808 | 2027,179 | 2359,171 | 1193,154 |
| ENSMUSG00000041592 | Sdk2 | -1,62 | 0,040544645 | 9,488113 | 11,59545 | 16,4352 | 16,85185 | 36,78277 | 62,28246 |
| ENSMUSG00000034308 | Sdr42e1 | 1,14 | 5,90947E-11 | 531,3343 | 574,9412 | 576,097 | 250,7952 | 270,1631 | 242,0118 |
| ENSMUSG00000029596 | Sdsl | -2,66 | 8,50175E-09 | 74,85067 | 56,04469 | 73,5259 | 284,4989 | 300,604 | 706,461 |
| ENSMUSG00000003585 | Sec14l2 | -3,52 | 9,04168E-07 | 331,0297 | 44,44924 | 70,93087 | 1587,048 | 1869,579 | 1643,367 |
| ENSMUSG00000091712 | Sec14l5 | -5,47 | 3,113E-06 | 12,65082 | 0,966288 | 0,865011 | 99,12855 | 49,46648 | 480,4647 |
| ENSMUSG00000030539 | Sema4b | -1,60 | 1,11816E-10 | 1658,311 | 1078,377 | 1437,648 | 4722,484 | 3717,596 | 4245,884 |
| ENSMUSG00000000627 | Sema4f | -2,43 | 2,88867E-14 | 119,1285 | 93,72992 | 109,8563 | 759,3247 | 408,4156 | 574,7781 |
| ENSMUSG00000022231 | Sema5a | -2,30 | 0,000183367 | 16,86776 | 9,662878 | 15,57019 | 34,69499 | 74,83391 | 97,87243 |
| ENSMUSG00000027200 | Sema6d | -2,19 | 0,000233654 | 191,8707 | 185,5273 | 254,3131 | 490,6863 | 551,7415 | 1848,899 |
| ENSMUSG00000000486 | Sept1 | -1,44 | 2,60264E-13 | 2378,354 | 1797,295 | 2145,226 | 6282,767 | 5257,399 | 5640,121 |
| ENSMUSG00000019917 | Sept10 | 1,20 | 1,64539E-06 | 451,2125 | 659,9746 | 585,6122 | 227,9957 | 249,8692 | 263,3658 |
| ENSMUSG00000020486 | Sept4 | -1,86 | 4,10947E-06 | 66,41679 | 61,84242 | 64,01078 | 178,4314 | 172,4985 | 344,333 |
| ENSMUSG00000072214 | Sept5 | -1,21 | 0,000709402 | 1773,223 | 2778,077 | 1828,632 | 5723,682 | 5616,348 | 3378,378 |
| ENSMUSG00000018398 | Sept8 | -2,55 | 2,5789E-18 | 1414,783 | 769,1651 | 870,2006 | 5771,264 | 6038,716 | 6017,375 |
| ENSMUSG00000059248 | Sept9 | 1,17 | 2,2112E-13 | 22885,33 | 24279,91 | 24753,14 | 10116,07 | 9911,054 | 11898,62 |
| ENSMUSG00000030839 | Sergef | 2,75 | 2,37065E-17 | 1868,104 | 1641,723 | 2212,697 | 222,0479 | 225,7701 | 403,9462 |
| ENSMUSG00000021703 | Serinc5 | -1,03 | 0,016885874 | 4146,305 | 2931,717 | 3537,028 | 9937,637 | 7686,33 | 4094,627 |
| ENSMUSG00000092572 | Serpinb10 | -2,74 | 4,26583E-06 | 30,57281 | 21,25833 | 12,97516 | 100,1198 | 90,05436 | 238,4528 |
| ENSMUSG00000026327 | Serpinb11 | 8,12 | 0,019383809 | 4,216939 | 16,42689 | 11,24514 | 0 | 0 | 0 |
| ENSMUSG00000060147 | Serpinb6a | -1,36 | 0,001267656 | 747,4524 | 847,4344 | 1147,004 | 1491,885 | 2068,714 | 3472,692 |
| ENSMUSG00000042842 | Serpinb6b | -1,02 | 0,004146126 | 374,2533 | 231,9091 | 355,5194 | 524,39 | 589,7927 | 834,5849 |
| ENSMUSG00000026249 | Serpine2 | -2,13 | 0,001536007 | 1548,671 | 848,4007 | 1871,018 | 2061,874 | 5169,882 | 11477,77 |
| ENSMUSG00000049800 | Sertad2 | -1,09 | 0,000594639 | 2524,892 | 1390,488 | 1883,128 | 4760,153 | 3898,973 | 3689,791 |
| ENSMUSG00000042272 | Sestd1 | 2,25 | 2,54184E-14 | 1501,23 | 2484,326 | 2778,414 | 467,8867 | 495,9332 | 458,2209 |
| ENSMUSG00000024548 | Setbp1 | -1,18 | 0,000561997 | 188,708 | 130,4489 | 107,2613 | 325,1416 | 268,8947 | 371,9152 |
| ENSMUSG00000061186 | Sfmbt2 | -1,28 | 0,000135713 | 123,3455 | 98,56136 | 145,3218 | 374,7059 | 227,0385 | 291,8378 |
| ENSMUSG00000047281 | Sfn | -1,16 | 1,60071E-05 | 312,0535 | 296,6504 | 287,1835 | 809,8802 | 506,0802 | 677,989 |
| ENSMUSG00000031548 | Sfrp1 | -1,82 | 0,011670507 | 36,89822 | 19,32576 | 39,79049 | 40,6427 | 114,1534 | 183,2884 |
| ENSMUSG00000027996 | Sfrp2 | 6,73 | 1,39853E-31 | 285,6976 | 417,4363 | 497,3811 | 0,991285 | 5,073485 | 5,338496 |
| ENSMUSG00000021319 | Sfrp4 | -2,23 | 0,003713045 | 201,3588 | 41,55038 | 186,8423 | 238,8998 | 852,3455 | 924,4496 |
| ENSMUSG00000056370 | Sftpb | -2,04 | 8,55806E-06 | 11,59658 | 19,32576 | 12,97516 | 54,5207 | 71,02879 | 56,05421 |
| ENSMUSG00000025212 | Sfxn3 | 1,05 | 0,001139664 | 1796,416 | 2320,057 | 2616,657 | 850,5229 | 982,9878 | 1418,26 |
| ENSMUSG00000004631 | Sgce | -3,24 | 2,55E-05 | 39,00669 | 30,92121 | 31,14038 | 95,1634 | 163,6199 | 695,784 |
| ENSMUSG00000028524 | Sgip1 | 2,93 | 1,86955E-12 | 251,9621 | 318,875 | 595,9923 | 49,56427 | 52,00322 | 51,60546 |
| ENSMUSG00000019970 | Sgk1 | -1,31 | 2,03345E-07 | 893,9911 | 595,2333 | 867,6056 | 2134,238 | 1925,388 | 1769,711 |
| ENSMUSG00000021054 | Sgpp1 | 1,03 | 1,86035E-05 | 6191,521 | 5501,076 | 6828,394 | 3547,811 | 3084,679 | 2418,339 |
| ENSMUSG00000053886 | Sh2d4a | -3,89 | 9,15837E-08 | 78,01337 | 10,62917 | 17,30021 | 603,6928 | 620,2336 | 336,3253 |
| ENSMUSG00000037833 | Sh2d4b | -1,56 | 0,030951039 | 28,46434 | 27,05606 | 19,89524 | 70,38127 | 27,90417 | 121,8957 |
| ENSMUSG00000045349 | Sh2d5 | 1,56 | 8,39078E-08 | 1897,623 | 3324,996 | 2582,057 | 759,3247 | 901,812 | 976,9448 |
| ENSMUSG00000052631 | Sh2d6 | 5,98 | 5,06856E-11 | 47,44056 | 225,1451 | 118,5065 | 1,982571 | 2,536743 | 1,779499 |
| ENSMUSG00000040666 | Sh3bgr | -1,75 | 0,034583381 | 6,325408 | 14,49432 | 8,650106 | 23,79085 | 19,02557 | 56,05421 |
| ENSMUSG00000032261 | Sh3bgrl2 | -3,72 | 1,88344E-06 | 99,09807 | 32,85379 | 88,23108 | 281,5251 | 550,4732 | 2073,116 |
| ENSMUSG00000053617 | Sh3pxd2a | -1,21 | 5,99209E-06 | 514,4666 | 384,5825 | 630,5927 | 1201,438 | 1090,799 | 1247,429 |
| ENSMUSG00000040711 | Sh3pxd2b | 1,43 | 8,58273E-05 | 1562,376 | 1237,815 | 1387,477 | 423,2789 | 758,4861 | 369,246 |
| ENSMUSG00000057719 | Sh3rf2 | 5,50 | 5,91304E-13 | 79,06761 | 78,26931 | 119,3715 | 0,991285 | 1,268371 | 3,558997 |
| ENSMUSG00000045629 | Sh3tc2 | -3,59 | 2,72977E-07 | 445,9413 | 60,87613 | 121,9665 | 2793,442 | 2993,356 | 1777,719 |
| ENSMUSG00000020669 | Sh3yl1 | -2,50 | 5,98853E-09 | 144,4302 | 63,77499 | 62,28076 | 606,6667 | 520,0322 | 402,1667 |
| ENSMUSG00000038738 | Shank1 | -1,91 | 1,32104E-05 | 14,75929 | 14,49432 | 19,03023 | 64,43356 | 49,46648 | 67,62095 |
| ENSMUSG00000037541 | Shank2 | -1,67 | 0,042935382 | 11,59658 | 7,730302 | 11,24514 | 44,60785 | 40,58788 | 12,45649 |
| ENSMUSG00000022623 | Shank3 | -2,19 | 1,98671E-05 | 154,9725 | 179,7295 | 121,9665 | 467,8867 | 403,3421 | 1204,721 |
| ENSMUSG00000035109 | Shc4 | -1,74 | 0,000492547 | 33,73551 | 21,25833 | 36,33044 | 112,0153 | 133,179 | 61,39271 |
| ENSMUSG00000033256 | Shf | -1,05 | 0,001818665 | 147,5929 | 108,2242 | 146,1868 | 292,4292 | 337,3868 | 206,4219 |
| ENSMUSG00000044461 | Shisa2 | 4,00 | 1,26958E-17 | 389,0126 | 514,0651 | 647,8929 | 34,69499 | 49,46648 | 14,23599 |
| ENSMUSG00000096883 | Shisa8 | -1,66 | 0,018786807 | 18,97623 | 9,662878 | 11,24514 | 60,46841 | 21,56231 | 42,70797 |
| ENSMUSG00000022696 | Sidt1 | -1,73 | 0,002811791 | 119,1285 | 57,97727 | 36,33044 | 212,1351 | 167,425 | 326,538 |
| ENSMUSG00000030474 | Siglece | -1,95 | 2,1832E-27 | 941,4316 | 1068,714 | 836,4652 | 3902,691 | 3679,545 | 3389,945 |
| ENSMUSG00000039013 | Siglecf | 1,10 | 0,019431459 | 1622,467 | 1664,914 | 1559,614 | 702,8214 | 1159,291 | 397,718 |
| ENSMUSG00000030468 | Siglecg | -1,10 | 0,037131998 | 2687,244 | 1378,893 | 1742,996 | 6130,109 | 4018,2 | 2260,853 |
| ENSMUSG00000051504 | Siglech | -2,18 | 4,64862E-05 | 1248,214 | 1163,411 | 865,8756 | 2444,51 | 3514,657 | 8939,312 |
| ENSMUSG00000036078 | Sigmar1 | 1,18 | 5,19568E-09 | 2236,032 | 2353,877 | 2412,515 | 844,5752 | 1103,483 | 1138,879 |
| ENSMUSG00000074677 | Sirpb1c | 1,05 | 0,018514072 | 924,5639 | 2161,586 | 1531,934 | 837,6362 | 549,2048 | 847,9311 |
| ENSMUSG00000028460 | Sit1 | -1,44 | 0,000911795 | 57,98291 | 28,98863 | 69,20085 | 149,6841 | 128,1055 | 145,9189 |
| ENSMUSG00000027636 | Sla2 | -2,09 | 0,007689125 | 292,023 | 113,0557 | 316,5939 | 404,4445 | 533,9843 | 2140,737 |
| ENSMUSG00000055717 | Slain1 | -1,40 | 0,00275111 | 265,6672 | 97,59507 | 173,8671 | 588,8236 | 395,7319 | 431,5284 |
| ENSMUSG00000089707 | Slain1os | -2,30 | 0,038008685 | 6,325408 | 1,932576 | 0,865011 | 11,89543 | 13,95208 | 17,79499 |
| ENSMUSG00000015316 | Slamf1 | -3,65 | 1,52606E-05 | 183,4368 | 70,53901 | 93,42114 | 442,1133 | 506,0802 | 3404,181 |
| ENSMUSG00000015314 | Slamf6 | -1,78 | 5,35964E-05 | 361,6025 | 145,9095 | 200,6825 | 1044,815 | 662,0898 | 715,3585 |
| ENSMUSG00000023030 | Slc11a2 | -1,55 | 5,83112E-09 | 1511,773 | 951,7935 | 1140,949 | 3877,909 | 3675,74 | 2986,889 |
| ENSMUSG00000037344 | Slc12a9 | 1,21 | 3,55593E-09 | 2746,282 | 3415,827 | 2622,712 | 1398,704 | 1170,707 | 1214,508 |
| ENSMUSG00000018459 | Slc13a3 | -3,15 | 0,00131605 | 5,271174 | 1,932576 | 17,30021 | 37,66885 | 41,85625 | 139,6907 |
| ENSMUSG00000020805 | Slc13a5 | 3,29 | 0,000219997 | 13,70505 | 58,94356 | 38,06047 | 3,965142 | 3,805114 | 3,558997 |
| ENSMUSG00000059336 | Slc14a1 | -3,19 | 2,45576E-11 | 1327,282 | 614,559 | 845,1153 | 5843,628 | 5740,649 | 13884,54 |
| ENSMUSG00000024552 | Slc14a2 | -3,85 | 0,002344101 | 3,162704 | 3,865151 | 0,865011 | 17,84314 | 7,610228 | 86,30569 |
| ENSMUSG00000024737 | Slc15a3 | -1,61 | 4,18917E-06 | 1740,542 | 1180,804 | 1476,573 | 4744,292 | 5788,847 | 2890,796 |
| ENSMUSG00000032902 | Slc16a1 | -1,58 | 8,92086E-07 | 4887,432 | 3065,065 | 3960,883 | 14204,13 | 12985,59 | 8426,816 |
| ENSMUSG00000019838 | Slc16a10 | -1,56 | 7,83468E-06 | 4079,888 | 2189,608 | 3158,154 | 11182,69 | 9890,76 | 6816,37 |
| ENSMUSG00000009378 | Slc16a12 | -3,20 | 0,001534902 | 3,162704 | 2,898863 | 3,460042 | 15,86057 | 13,95208 | 56,94396 |
| ENSMUSG00000044367 | Slc16a13 | 1,34 | 2,72348E-07 | 476,5141 | 570,1098 | 539,7666 | 215,1089 | 159,8148 | 248,2401 |
| ENSMUSG00000033965 | Slc16a2 | 1,32 | 0,000304697 | 187,6538 | 177,797 | 233,5529 | 56,50327 | 77,37065 | 105,8802 |
| ENSMUSG00000025161 | Slc16a3 | -1,29 | 0,000241378 | 5171,021 | 3865,151 | 4159,836 | 10784,19 | 14361,77 | 7039,697 |
| ENSMUSG00000020102 | Slc16a7 | 1,45 | 1,90104E-17 | 710,5542 | 803,9515 | 741,3141 | 274,5861 | 290,457 | 262,4761 |
| ENSMUSG00000037762 | Slc16a9 | -2,04 | 0,00324492 | 48,4948 | 14,49432 | 46,71057 | 113,0065 | 86,24925 | 251,7991 |
| ENSMUSG00000036330 | Slc18a1 | 1,70 | 9,26718E-06 | 68,52526 | 89,86477 | 93,42114 | 23,79085 | 22,83068 | 30,25148 |
| ENSMUSG00000025094 | Slc18a2 | -1,34 | 0,004891102 | 317,3247 | 345,931 | 368,4945 | 622,5273 | 550,4732 | 1439,614 |
| ENSMUSG00000037455 | Slc18b1 | -1,65 | 0,002078386 | 17,92199 | 19,32576 | 24,2203 | 57,49456 | 41,85625 | 92,53393 |
| ENSMUSG00000005360 | Slc1a3 | -2,06 | 0,008561166 | 40,06092 | 17,39318 | 72,66089 | 114,9891 | 93,85948 | 333,656 |
| ENSMUSG00000027397 | Slc20a1 | -1,28 | 1,90463E-09 | 2823,241 | 2224,395 | 2070,835 | 6275,828 | 5213,006 | 5828,748 |
| ENSMUSG00000061742 | Slc22a12 | -2,34 | 0,006306507 | 12,65082 | 2,898863 | 2,595032 | 25,77342 | 30,44091 | 33,81048 |
| ENSMUSG00000000154 | Slc22a18 | 1,21 | 0,008801922 | 78,01337 | 76,33674 | 88,23108 | 27,75599 | 25,36743 | 50,71571 |
| ENSMUSG00000037451 | Slc22a20 | -1,33 | 0,01357049 | 40,06092 | 112,0894 | 55,36068 | 197,2658 | 129,3739 | 193,0756 |
| ENSMUSG00000027340 | Slc23a2 | 1,11 | 2,10546E-07 | 3024,599 | 2606,078 | 2709,213 | 1137,004 | 1193,537 | 1535,707 |
| ENSMUSG00000063873 | Slc24a3 | -2,75 | 0,000319867 | 88,55572 | 97,59507 | 76,98594 | 200,2397 | 254,9426 | 1316,829 |
| ENSMUSG00000035183 | Slc24a5 | -2,47 | 0,00080944 | 94,88113 | 84,06704 | 77,85095 | 183,3878 | 213,0864 | 1024,991 |
| ENSMUSG00000035472 | Slc25a21 | -3,70 | 1,19315E-08 | 127,5624 | 20,29204 | 35,46543 | 764,2811 | 927,1794 | 677,989 |
| ENSMUSG00000069041 | Slc25a31 | -9,98 | 0,003426398 | 0 | 0 | 0 | 1,982571 | 59,61345 | 45,37722 |
| ENSMUSG00000028982 | Slc25a33 | -1,21 | 0,000816467 | 160,2437 | 105,3254 | 116,7764 | 260,7081 | 390,6584 | 235,7836 |
| ENSMUSG00000034248 | Slc25a37 | -2,48 | 1,72123E-08 | 17266,26 | 6872,239 | 8179,54 | 72858,49 | 64254,42 | 42854,78 |
| ENSMUSG00000032519 | Slc25a38 | -1,52 | 1,68904E-14 | 2049,432 | 1819,52 | 1612,38 | 5682,048 | 5464,144 | 4549,289 |
| ENSMUSG00000018677 | Slc25a39 | -1,78 | 1,73362E-16 | 4362,423 | 3373,311 | 4549,091 | 15812,99 | 12324,76 | 14117,65 |
| ENSMUSG00000002346 | Slc25a42 | -1,46 | 5,2452E-06 | 302,5654 | 348,8299 | 262,0982 | 976,4162 | 971,5724 | 569,4396 |
| ENSMUSG00000045973 | Slc25a51 | -1,46 | 4,63824E-06 | 7199,369 | 4510,631 | 5864,772 | 19649,26 | 16842,7 | 11759,82 |
| ENSMUSG00000044348 | Slc25a53 | -1,02 | 2,66224E-05 | 399,555 | 281,1898 | 396,1748 | 784,1068 | 692,5308 | 711,7995 |
| ENSMUSG00000046959 | Slc26a1 | -3,15 | 1,49095E-13 | 105,4235 | 102,4265 | 38,92548 | 734,5425 | 862,4925 | 593,4628 |
| ENSMUSG00000001225 | Slc26a3 | -2,80 | 0,009724626 | 3,162704 | 1,932576 | 0,865011 | 15,86057 | 13,95208 | 10,67699 |
| ENSMUSG00000040569 | Slc26a7 | -2,67 | 0,001752971 | 63,25408 | 20,29204 | 37,19546 | 69,38998 | 161,0832 | 533,8496 |
| ENSMUSG00000027359 | Slc27a2 | -3,10 | 2,50522E-05 | 6,325408 | 2,898863 | 3,460042 | 22,79957 | 38,05114 | 46,26697 |
| ENSMUSG00000027932 | Slc27a3 | 3,66 | 3,02778E-26 | 900,3165 | 1578,914 | 1518,094 | 137,7887 | 93,85948 | 83,63644 |
| ENSMUSG00000059316 | Slc27a4 | -1,45 | 0,002792017 | 4683,965 | 3031,245 | 3197,944 | 9069,271 | 15443,69 | 5264,647 |
| ENSMUSG00000025726 | Slc28a1 | -3,11 | 0,021349996 | 2,108469 | 2,898863 | 0 | 23,79085 | 11,41534 | 7,117995 |
| ENSMUSG00000027219 | Slc28a2 | -1,14 | 0,000158925 | 1469,603 | 1365,365 | 1262,915 | 3355,501 | 3597,101 | 2061,549 |
| ENSMUSG00000020100 | Slc29a3 | 1,32 | 1,3814E-11 | 5161,533 | 5478,852 | 5387,286 | 2173,889 | 1783,33 | 2453,929 |
| ENSMUSG00000050822 | Slc29a4 | 1,76 | 0,000867423 | 44,27786 | 87,93219 | 82,17601 | 19,82571 | 15,22046 | 27,58223 |
| ENSMUSG00000018566 | Slc2a4 | -3,44 | 9,96394E-13 | 177,1114 | 53,14583 | 67,47083 | 1177,647 | 1104,751 | 943,1343 |
| ENSMUSG00000028976 | Slc2a5 | 5,83 | 2,18045E-08 | 48,4948 | 49,28068 | 76,98594 | 0 | 1,268371 | 1,779499 |
| ENSMUSG00000026614 | Slc30a10 | -4,50 | 2,72977E-07 | 28,46434 | 0,966288 | 7,785095 | 327,1242 | 361,4858 | 145,9189 |
| ENSMUSG00000005802 | Slc30a4 | -1,04 | 0,013743056 | 372,1449 | 175,8644 | 326,109 | 686,9608 | 653,2112 | 454,6619 |
| ENSMUSG00000037771 | Slc32a1 | -1,89 | 0,020322059 | 8,433878 | 2,898863 | 5,190064 | 19,82571 | 25,36743 | 16,01549 |
| ENSMUSG00000021490 | Slc34a1 | 2,49 | 1,48689E-05 | 62,19985 | 59,90984 | 46,71057 | 14,86928 | 3,805114 | 10,67699 |
| ENSMUSG00000049922 | Slc35c1 | 1,29 | 2,52892E-07 | 10163,88 | 8701,422 | 10380,13 | 4694,728 | 4250,312 | 3043,833 |
| ENSMUSG00000050473 | Slc35d3 | -2,98 | 0,001548762 | 75,9049 | 91,79734 | 76,12093 | 176,4488 | 143,326 | 1607,777 |
| ENSMUSG00000048807 | Slc35e4 | 1,37 | 6,75401E-07 | 554,5275 | 608,7613 | 741,3141 | 190,3268 | 254,9426 | 290,948 |
| ENSMUSG00000042195 | Slc35f2 | -2,18 | 0,002098856 | 24,2474 | 14,49432 | 30,27537 | 51,54684 | 68,49205 | 193,0756 |
| ENSMUSG00000020261 | Slc36a1 | 1,36 | 1,06982E-08 | 6523,605 | 6083,748 | 5824,981 | 2925,283 | 2385,806 | 1888,938 |
| ENSMUSG00000020264 | Slc36a2 | 3,75 | 2,14905E-26 | 503,9242 | 344,9647 | 492,191 | 25,77342 | 26,6358 | 46,26697 |
| ENSMUSG00000032122 | Slc37a2 | -1,13 | 0,000184201 | 590,3715 | 471,5484 | 626,2677 | 1026,972 | 1052,748 | 1604,218 |
| ENSMUSG00000031170 | Slc38a5 | -3,72 | 1,99506E-10 | 517,6293 | 93,72992 | 189,4373 | 3418,944 | 3678,277 | 3427,315 |
| ENSMUSG00000063354 | Slc39a4 | -1,07 | 0,00445975 | 132,8336 | 159,4375 | 107,2613 | 257,7342 | 208,0129 | 371,9152 |
| ENSMUSG00000053897 | Slc39a8 | -1,40 | 3,75936E-09 | 286,7518 | 202,9204 | 284,5885 | 627,4837 | 735,6554 | 686,8865 |
| ENSMUSG00000025993 | Slc40a1 | -2,32 | 2,21837E-22 | 3116,318 | 3638,074 | 3413,332 | 15041,77 | 14201,95 | 21498,12 |
| ENSMUSG00000034591 | Slc41a2 | -2,27 | 0,000152683 | 160,2437 | 57,97727 | 88,23108 | 302,3421 | 331,0449 | 837,2542 |
| ENSMUSG00000027075 | Slc43a1 | -3,04 | 8,98187E-11 | 2303,503 | 707,3227 | 1092,508 | 13444,8 | 11269,48 | 9022,059 |
| ENSMUSG00000026435 | Slc45a3 | -2,59 | 1,87908E-05 | 71,68796 | 45,41553 | 108,1263 | 284,4989 | 253,6743 | 822,1284 |
| ENSMUSG00000020829 | Slc46a1 | 2,75 | 1,532E-09 | 218,2266 | 347,8636 | 359,8444 | 38,66013 | 26,6358 | 71,17995 |
| ENSMUSG00000029650 | Slc46a3 | -1,29 | 9,77E-08 | 312,0535 | 406,8072 | 383,1997 | 820,7844 | 802,8791 | 1072,148 |
| ENSMUSG00000006574 | Slc4a1 | -3,40 | 3,08624E-08 | 28355,75 | 4616,923 | 11316,93 | 169277,9 | 169082,8 | 128746,7 |
| ENSMUSG00000068323 | Slc4a5 | 3,23 | 7,58427E-09 | 62,19985 | 53,14583 | 84,77104 | 9,912855 | 3,805114 | 7,117995 |
| ENSMUSG00000021733 | Slc4a7 | 1,07 | 9,58587E-12 | 2853,813 | 3021,582 | 2967,851 | 1524,597 | 1268,371 | 1408,473 |
| ENSMUSG00000023032 | Slc4a8 | 1,31 | 0,009738223 | 467,026 | 178,7632 | 307,0788 | 90,20698 | 131,9106 | 161,9344 |
| ENSMUSG00000053862 | Slc51b | 3,17 | 0,040639234 | 8,433878 | 18,35947 | 24,2203 | 0 | 0 | 5,338496 |
| ENSMUSG00000027463 | Slc52a3 | -1,04 | 0,009961809 | 65,36255 | 65,70757 | 50,17061 | 88,22441 | 126,8371 | 155,7061 |
| ENSMUSG00000030769 | Slc5a11 | -2,10 | 3,27315E-08 | 32,68128 | 41,55038 | 38,92548 | 197,2658 | 180,1087 | 109,4392 |
| ENSMUSG00000030781 | Slc5a2 | -2,61 | 0,045351998 | 2,108469 | 0,966288 | 1,730021 | 13,878 | 10,14697 | 5,338496 |
| ENSMUSG00000030109 | Slc6a12 | -1,24 | 0,002077671 | 101,2065 | 159,4375 | 99,47622 | 315,2288 | 344,997 | 193,0756 |
| ENSMUSG00000030108 | Slc6a13 | -1,50 | 1,89198E-07 | 131,7793 | 79,2356 | 97,7462 | 272,6035 | 305,6775 | 293,6173 |
| ENSMUSG00000021565 | Slc6a19 | -2,65 | 0,00032136 | 6,325408 | 2,898863 | 4,325053 | 25,77342 | 21,56231 | 36,47972 |
| ENSMUSG00000036814 | Slc6a20a | -3,69 | 8,1881E-10 | 96,9896 | 28,02235 | 24,2203 | 746,438 | 776,2433 | 399,4975 |
| ENSMUSG00000021609 | Slc6a3 | -3,64 | 5,54424E-05 | 12,65082 | 20,29204 | 23,35529 | 76,32898 | 68,49205 | 556,0934 |
| ENSMUSG00000020838 | Slc6a4 | -4,58 | 0,00060933 | 321,5416 | 55,0784 | 128,8866 | 1025,98 | 1340,669 | 9718,732 |
| ENSMUSG00000019558 | Slc6a8 | -1,44 | 0,02009019 | 50,60327 | 50,24697 | 100,3412 | 87,23312 | 271,4315 | 187,7371 |
| ENSMUSG00000028542 | Slc6a9 | -3,17 | 1,11413E-07 | 606,185 | 112,0894 | 237,8779 | 3359,466 | 3007,308 | 2251,066 |
| ENSMUSG00000027737 | Slc7a11 | -2,44 | 0,008441313 | 751,6694 | 137,2129 | 208,4676 | 1760,523 | 3715,06 | 462,6697 |
| ENSMUSG00000031596 | Slc7a2 | -1,81 | 0,009532646 | 25,30163 | 12,56174 | 49,3056 | 60,46841 | 150,9362 | 96,98268 |
| ENSMUSG00000040010 | Slc7a5 | -1,18 | 3,66159E-11 | 3324,002 | 3213,873 | 3762,796 | 8077,985 | 8361,104 | 6858,188 |
| ENSMUSG00000032754 | Slc8b1 | 1,21 | 1,49279E-07 | 9546,096 | 10325,75 | 10292,76 | 5108,094 | 4554,721 | 3397,953 |
| ENSMUSG00000002504 | Slc9a3r2 | -1,39 | 0,00024399 | 101,2065 | 53,14583 | 102,0712 | 193,3007 | 256,211 | 222,4373 |
| ENSMUSG00000037341 | Slc9a7 | 1,52 | 2,32423E-10 | 1186,014 | 1169,208 | 1184,199 | 519,4336 | 372,9012 | 342,5535 |
| ENSMUSG00000030737 | Slco2b1 | -1,86 | 0,002998369 | 294,1315 | 150,7409 | 438,5604 | 787,0807 | 572,0355 | 1843,561 |
| ENSMUSG00000040693 | Slco4c1 | -2,27 | 5,79127E-08 | 370,0364 | 277,3246 | 182,5172 | 1507,745 | 1688,202 | 806,1129 |
| ENSMUSG00000025938 | Slco5a1 | -1,49 | 0,048209132 | 41,11515 | 9,662878 | 36,33044 | 118,9543 | 52,00322 | 72,95945 |
| ENSMUSG00000082101 | Slfn14 | -3,21 | 8,02199E-10 | 1108,001 | 256,0663 | 518,1413 | 5629,51 | 6530,844 | 5208,593 |
| ENSMUSG00000054404 | Slfn5 | 1,46 | 1,20412E-06 | 2930,773 | 4900,045 | 4915,855 | 1384,826 | 1471,311 | 1788,396 |
| ENSMUSG00000069793 | Slfn9 | 1,33 | 3,43794E-13 | 2248,683 | 2393,495 | 2480,85 | 813,8454 | 974,1092 | 1047,235 |
| ENSMUSG00000025020 | Slit1 | -1,51 | 0,003113689 | 42,16939 | 22,22462 | 28,54535 | 116,9717 | 90,05436 | 57,83371 |
| ENSMUSG00000056427 | Slit3 | -1,31 | 0,046456833 | 50,60327 | 27,05606 | 67,47083 | 58,48584 | 131,9106 | 169,0524 |
| ENSMUSG00000027796 | Smad9 | -7,49 | 0,042799095 | 0 | 0 | 0 | 1,982571 | 6,341857 | 10,67699 |
| ENSMUSG00000078350 | Smim1 | -3,08 | 4,07749E-08 | 245,6367 | 52,17954 | 90,82611 | 1069,597 | 1111,093 | 1099,73 |
| ENSMUSG00000021136 | Smoc1 | -1,25 | 0,002296787 | 56,92868 | 54,11212 | 84,77104 | 125,8933 | 133,179 | 206,4219 |
| ENSMUSG00000027333 | Smox | -2,55 | 1,22992E-12 | 2104,253 | 892,8499 | 1325,196 | 7828,181 | 8007,228 | 9538,113 |
| ENSMUSG00000071724 | Smpd5 | -1,61 | 5,81605E-08 | 130,7251 | 119,8197 | 87,36607 | 335,0545 | 412,2207 | 286,4993 |
| ENSMUSG00000028885 | Smpdl3b | -1,30 | 0,00909113 | 53,76597 | 45,41553 | 62,28076 | 118,9543 | 79,90739 | 196,6346 |
| ENSMUSG00000042821 | Snai1 | -1,76 | 0,042652544 | 62,19985 | 15,4606 | 54,49567 | 44,60785 | 232,112 | 170,8319 |
| ENSMUSG00000025889 | Snca | -3,97 | 1,20062E-17 | 1771,114 | 486,0428 | 928,1564 | 18371,49 | 15245,82 | 16294,87 |
| ENSMUSG00000037972 | Snn | -1,80 | 0,000296392 | 1267,19 | 642,5814 | 1453,218 | 6064,684 | 2993,356 | 2614,084 |
| ENSMUSG00000027457 | Snph | 2,75 | 0,015655779 | 18,97623 | 61,84242 | 12,97516 | 0,991285 | 8,878599 | 4,448747 |
| ENSMUSG00000020672 | Sntg2 | -3,12 | 0,006648739 | 3,162704 | 1,932576 | 1,730021 | 11,89543 | 8,878599 | 37,36947 |
| ENSMUSG00000024787 | Snx15 | -1,18 | 2,47102E-09 | 736,9101 | 782,6931 | 697,1985 | 1442,32 | 1662,835 | 1909,402 |
| ENSMUSG00000031662 | Snx20 | 1,03 | 3,88556E-13 | 4488,932 | 4848,832 | 4798,214 | 2444,51 | 2323,656 | 2176,327 |
| ENSMUSG00000039452 | Snx22 | -3,29 | 2,84008E-12 | 348,9517 | 113,0557 | 134,9417 | 2226,427 | 1921,583 | 1680,737 |
| ENSMUSG00000013611 | Snx31 | -4,41 | 0,00088159 | 0 | 0 | 3,460042 | 11,89543 | 40,58788 | 25,80273 |
| ENSMUSG00000028007 | Snx7 | 1,50 | 2,07929E-06 | 297,2942 | 281,1898 | 289,7785 | 72,36384 | 101,4697 | 133,4624 |
| ENSMUSG00000026600 | Soat1 | 1,13 | 0,003865543 | 10199,72 | 8107,155 | 9700,229 | 5251,83 | 5086,169 | 2431,685 |
| ENSMUSG00000025006 | Sorbs1 | -2,27 | 7,1307E-16 | 148,6471 | 85,03333 | 126,2915 | 591,7974 | 615,1601 | 524,0624 |
| ENSMUSG00000022091 | Sorbs3 | -1,39 | 0,01408397 | 143,3759 | 120,786 | 207,6025 | 263,6819 | 265,0896 | 706,461 |
| ENSMUSG00000044352 | Sowaha | -3,65 | 9,02661E-10 | 340,5178 | 68,60643 | 118,5065 | 2777,582 | 2293,215 | 1565,069 |
| ENSMUSG00000051817 | Sox12 | -1,27 | 0,000217457 | 234,0401 | 159,4375 | 156,5669 | 409,4009 | 347,5337 | 571,2191 |
| ENSMUSG00000046470 | Sox18 | -1,98 | 0,005902637 | 13,70505 | 39,6178 | 39,79049 | 84,25926 | 67,22368 | 216,2091 |
| ENSMUSG00000051910 | Sox6 | -3,17 | 3,61322E-09 | 1254,539 | 317,9087 | 512,0863 | 7274,053 | 7061,023 | 4437,18 |
| ENSMUSG00000030664 | Sox6os | -2,67 | 0,012482251 | 2,108469 | 1,932576 | 2,595032 | 20,81699 | 12,68371 | 8,897494 |
| ENSMUSG00000060284 | Sp7 | -2,40 | 0,005512125 | 6,325408 | 20,29204 | 7,785095 | 30,72985 | 36,78277 | 113,8879 |
| ENSMUSG00000028475 | Spaar | -1,98 | 0,042399598 | 3,162704 | 6,764015 | 6,920085 | 20,81699 | 8,878599 | 36,47972 |
| ENSMUSG00000026809 | Spaca9 | 1,79 | 2,58513E-08 | 175,003 | 204,853 | 241,338 | 44,60785 | 71,02879 | 64,9517 |
| ENSMUSG00000050876 | Spata31d1a | -3,32 | 0,02652918 | 0 | 0,966288 | 1,730021 | 9,912855 | 6,341857 | 11,56674 |
| ENSMUSG00000049653 | Spatc1 | -1,75 | 5,95912E-06 | 133,8878 | 159,4375 | 77,85095 | 414,3573 | 514,9588 | 320,3098 |
| ENSMUSG00000042331 | Specc1 | -1,90 | 6,69259E-14 | 4022,96 | 2864,077 | 3361,431 | 15624,64 | 12034,31 | 10630,73 |
| ENSMUSG00000061878 | Sphk1 | -3,71 | 3,57141E-13 | 326,8128 | 88,89848 | 113,3164 | 2389,989 | 2463,177 | 2051,762 |
| ENSMUSG00000008193 | Spib | -1,98 | 0,000395571 | 4344,501 | 1435,904 | 3084,628 | 17336,59 | 11393,78 | 6282,52 |
| ENSMUSG00000004359 | Spic | -3,19 | 3,7531E-07 | 109,6404 | 83,10075 | 230,0928 | 783,1155 | 688,7256 | 2387,198 |
| ENSMUSG00000053030 | Spink2 | 3,60 | 5,88584E-05 | 55,87444 | 34,78636 | 22,49028 | 5,947713 | 2,536743 | 0,889749 |
| ENSMUSG00000024533 | Spire1 | -3,09 | 5,80462E-11 | 1068,994 | 337,2344 | 463,6457 | 5892,201 | 5938,515 | 4139,114 |
| ENSMUSG00000010154 | Spire2 | -2,72 | 1,76338E-07 | 12,65082 | 8,69659 | 9,515116 | 65,42484 | 46,92974 | 88,97494 |
| ENSMUSG00000040447 | Spns2 | -1,99 | 8,08816E-05 | 401,6634 | 439,6609 | 429,9103 | 1131,057 | 942,3999 | 2978,881 |
| ENSMUSG00000037379 | Spon2 | -4,40 | 3,59676E-10 | 24,2474 | 3,865151 | 3,460042 | 229,9782 | 239,7222 | 185,9576 |
| ENSMUSG00000035206 | Sppl2b | -1,65 | 7,32406E-11 | 1809,067 | 1853,34 | 1536,259 | 6701,09 | 5427,361 | 4193,389 |
| ENSMUSG00000037211 | Spry1 | -1,72 | 0,04048923 | 186,5995 | 172,9655 | 219,7127 | 203,2135 | 280,3101 | 1429,827 |
| ENSMUSG00000026532 | Spta1 | -3,54 | 3,47035E-09 | 3208,036 | 580,739 | 1143,544 | 20737,69 | 21133,6 | 15591,08 |
| ENSMUSG00000021061 | Sptb | -3,37 | 8,31909E-09 | 6019,68 | 1131,523 | 2317,363 | 34871,44 | 35270,87 | 27482,58 |
| ENSMUSG00000027646 | Src | -4,28 | 9,44034E-08 | 217,1724 | 92,76363 | 118,5065 | 884,2266 | 969,0357 | 6481,824 |
| ENSMUSG00000027579 | Srms | -2,29 | 9,3833E-06 | 10,54235 | 8,69659 | 7,785095 | 50,55556 | 39,31951 | 41,81822 |
| ENSMUSG00000002007 | Srpk3 | -2,24 | 4,63703E-07 | 163,4064 | 81,16818 | 140,1317 | 902,0698 | 473,1025 | 436,8669 |
| ENSMUSG00000032802 | Srxn1 | -1,41 | 0,01966053 | 497,5988 | 464,7844 | 643,5679 | 653,2571 | 1019,771 | 2599,848 |
| ENSMUSG00000030255 | Sspn | -1,24 | 0,002403323 | 28,46434 | 31,8875 | 37,19546 | 63,44227 | 71,02879 | 96,09293 |
| ENSMUSG00000037014 | Sstr4 | -1,66 | 0,037111599 | 12,65082 | 18,35947 | 12,11015 | 67,40741 | 53,2716 | 16,01549 |
| ENSMUSG00000036825 | Ssx2ip | -1,04 | 0,000456605 | 1407,403 | 897,6814 | 1058,773 | 2060,882 | 2049,688 | 2784,916 |
| ENSMUSG00000033740 | St18 | -1,93 | 0,033029559 | 4,216939 | 4,831439 | 2,595032 | 11,89543 | 15,22046 | 16,90524 |
| ENSMUSG00000056091 | St3gal5 | -2,70 | 6,02025E-25 | 2650,346 | 1730,621 | 1813,927 | 15084,39 | 13826,52 | 11201,05 |
| ENSMUSG00000022747 | St3gal6 | -2,27 | 9,96394E-13 | 617,7816 | 306,3132 | 388,3898 | 2079,717 | 1985,001 | 2246,617 |
| ENSMUSG00000031024 | St5 | -2,32 | 4,73628E-08 | 625,1612 | 364,2905 | 343,4092 | 2573,377 | 2798,027 | 1272,342 |
| ENSMUSG00000022885 | St6gal1 | -1,59 | 0,000277479 | 1636,172 | 569,1435 | 980,057 | 2942,135 | 3491,826 | 3179,074 |
| ENSMUSG00000057286 | St6galnac2 | -1,62 | 0,000134946 | 16,86776 | 29,95492 | 23,35529 | 66,41613 | 65,95531 | 83,63644 |
| ENSMUSG00000052544 | St6galnac3 | -1,67 | 0,000192028 | 41,11515 | 23,19091 | 32,8704 | 82,27669 | 87,51762 | 138,8009 |
| ENSMUSG00000030283 | St8sia1 | -2,27 | 0,019332577 | 72,7422 | 466,717 | 144,4568 | 1680,229 | 208,0129 | 1411,142 |
| ENSMUSG00000025789 | St8sia2 | -2,52 | 0,022455536 | 4,216939 | 6,764015 | 6,055074 | 6,938998 | 22,83068 | 67,62095 |
| ENSMUSG00000040710 | St8sia4 | 1,25 | 1,70562E-08 | 9590,373 | 8988,409 | 8881,064 | 4192,146 | 3009,845 | 4331,3 |
| ENSMUSG00000003418 | St8sia6 | 1,21 | 0,010305685 | 681,0356 | 735,345 | 919,5063 | 207,1787 | 261,2845 | 537,4086 |
| ENSMUSG00000042286 | Stab1 | -1,69 | 3,30172E-11 | 296,24 | 205,8193 | 285,4535 | 725,621 | 849,8088 | 963,5986 |
| ENSMUSG00000035459 | Stab2 | -1,95 | 1,76651E-05 | 293,0773 | 261,864 | 396,1748 | 909,0088 | 781,3167 | 1975,244 |
| ENSMUSG00000017400 | Stac2 | -1,73 | 0,000106837 | 103,315 | 42,51666 | 46,71057 | 214,1177 | 219,4282 | 203,7526 |
| ENSMUSG00000038781 | Stap2 | -1,03 | 0,049786774 | 52,71174 | 60,87613 | 33,73541 | 73,35512 | 83,71251 | 143,2496 |
| ENSMUSG00000030688 | Stard10 | -1,56 | 0,00070733 | 1137,519 | 573,0087 | 797,5398 | 1401,678 | 2609,04 | 3403,291 |
| ENSMUSG00000003062 | Stard3nl | 1,72 | 3,57056E-37 | 2783,18 | 2941,38 | 3057,812 | 856,4706 | 900,5436 | 901,3161 |
| ENSMUSG00000033705 | Stard9 | 1,29 | 1,40759E-05 | 2615,556 | 3089,222 | 2365,804 | 1466,111 | 920,8376 | 922,6701 |
| ENSMUSG00000062939 | Stat4 | -1,22 | 0,003395843 | 1432,705 | 747,9068 | 1047,528 | 2175,872 | 3503,242 | 1848,899 |
| ENSMUSG00000025920 | Stau2 | -1,83 | 0,001422926 | 90,66419 | 57,01098 | 99,47622 | 170,5011 | 196,5976 | 513,3854 |
| ENSMUSG00000014813 | Stc1 | 2,34 | 0,044268556 | 15,81352 | 13,52803 | 51,90064 | 0,991285 | 10,14697 | 5,338496 |
| ENSMUSG00000026389 | Steap3 | -1,94 | 2,94944E-11 | 1559,213 | 1033,928 | 1117,594 | 5716,743 | 4922,549 | 3615,941 |
| ENSMUSG00000012428 | Steap4 | -1,44 | 0,005858412 | 263,5587 | 115,9545 | 316,5939 | 533,3116 | 460,4188 | 890,6391 |
| ENSMUSG00000026094 | Stk17b | -1,08 | 0,001042369 | 10204,99 | 5478,852 | 8405,308 | 17364,35 | 19332,52 | 14355,22 |
| ENSMUSG00000039954 | Stk32a | -4,73 | 0,027694658 | 0 | 0 | 0,865011 | 9,912855 | 8,878599 | 6,228246 |
| ENSMUSG00000027030 | Stk39 | -1,68 | 7,58427E-09 | 250,9079 | 147,842 | 241,338 | 751,3944 | 672,2368 | 626,3836 |
| ENSMUSG00000038143 | Stox2 | 1,80 | 2,63776E-08 | 708,4457 | 878,3556 | 938,5365 | 247,8214 | 162,3515 | 312,302 |
| ENSMUSG00000026027 | Stradb | -1,85 | 3,75889E-14 | 1642,498 | 1104,467 | 1204,095 | 4868,203 | 5276,425 | 4131,996 |
| ENSMUSG00000039232 | Stx11 | -1,45 | 8,65533E-08 | 2762,095 | 2064,957 | 2122,736 | 4940,567 | 6279,706 | 7726,584 |
| ENSMUSG00000029428 | Stx2 | -1,55 | 6,15788E-11 | 2327,75 | 1879,43 | 2108,896 | 6673,334 | 6982,384 | 4796,639 |
| ENSMUSG00000026797 | Stxbp1 | -1,11 | 0,006560774 | 346,8432 | 190,3587 | 234,4179 | 509,5207 | 413,4891 | 745,61 |
| ENSMUSG00000020546 | Stxbp4 | -1,20 | 1,5115E-08 | 316,2704 | 239,6394 | 256,0431 | 583,8671 | 621,502 | 663,753 |
| ENSMUSG00000046314 | Stxbp6 | 2,81 | 2,19214E-09 | 785,4049 | 1478,42 | 1898,698 | 252,7778 | 232,112 | 110,3289 |
| ENSMUSG00000006800 | Sulf2 | 2,04 | 9,54438E-14 | 3971,302 | 4606,294 | 6319,767 | 1357,07 | 1283,592 | 992,0705 |
| ENSMUSG00000049858 | Suox | -1,39 | 0,000166145 | 722,1508 | 384,5825 | 432,5053 | 1229,194 | 1685,666 | 1108,628 |
| ENSMUSG00000006342 | Susd2 | -1,73 | 0,002744496 | 109,6404 | 78,26931 | 65,7408 | 138,78 | 224,5017 | 477,7954 |
| ENSMUSG00000038576 | Susd4 | -4,94 | 0,024197777 | 1,054235 | 0 | 0 | 8,921569 | 3,805114 | 16,90524 |
| ENSMUSG00000086596 | Susd5 | 4,15 | 9,98787E-07 | 100,1523 | 298,5829 | 140,9967 | 3,965142 | 2,536743 | 23,13348 |
| ENSMUSG00000028643 | Svbp | -1,02 | 1,88651E-09 | 570,341 | 484,1102 | 525,9264 | 1021,024 | 1055,285 | 1137,1 |
| ENSMUSG00000028369 | Svep1 | -1,60 | 0,018611014 | 41,11515 | 27,05606 | 69,20085 | 65,42484 | 126,8371 | 223,3271 |
| ENSMUSG00000060445 | Sycp2 | -1,38 | 0,031340288 | 49,54903 | 141,078 | 62,28076 | 315,2288 | 121,7636 | 222,4373 |
| ENSMUSG00000032714 | Syde1 | -1,46 | 0,000245412 | 67,47102 | 85,99961 | 85,63605 | 155,6318 | 191,5241 | 312,302 |
| ENSMUSG00000036863 | Syde2 | -1,28 | 0,037685336 | 49,54903 | 47,3481 | 62,28076 | 74,34641 | 88,78599 | 221,5476 |
| ENSMUSG00000067629 | Syngap1 | -1,07 | 1,44732E-06 | 153,9183 | 144,9432 | 126,2915 | 292,4292 | 318,3612 | 278,4916 |
| ENSMUSG00000022415 | Syngr1 | -2,24 | 4,83258E-19 | 126,5082 | 131,4151 | 132,3466 | 543,2244 | 768,633 | 532,0701 |
| ENSMUSG00000043079 | Synpo | -1,17 | 0,022224892 | 62,19985 | 87,93219 | 96,01617 | 98,13726 | 249,8692 | 208,2014 |
| ENSMUSG00000050315 | Synpo2 | -2,48 | 3,1391E-06 | 32,68128 | 18,35947 | 25,95032 | 88,22441 | 112,885 | 225,9963 |
| ENSMUSG00000016200 | Syt14 | -3,04 | 0,000774908 | 8,433878 | 1,932576 | 0,865011 | 32,71242 | 29,17254 | 27,58223 |
| ENSMUSG00000030731 | Syt3 | 1,94 | 1,95566E-05 | 63,25408 | 55,0784 | 56,22569 | 15,86057 | 10,14697 | 18,68474 |
| ENSMUSG00000027849 | Syt6 | -1,58 | 0,037645085 | 12,65082 | 8,69659 | 9,515116 | 16,85185 | 49,46648 | 26,69248 |
| ENSMUSG00000028860 | Sytl1 | -1,02 | 3,37311E-10 | 348,9517 | 373,9534 | 346,8692 | 756,3508 | 688,7256 | 728,7047 |
| ENSMUSG00000035476 | Tab3 | -1,46 | 1,15844E-08 | 1023,662 | 996,2427 | 904,8011 | 2969,891 | 3096,094 | 1977,913 |
| ENSMUSG00000025400 | Tac2 | -2,30 | 5,72443E-14 | 50,60327 | 61,84242 | 44,11554 | 206,1874 | 306,9459 | 258,9171 |
| ENSMUSG00000030852 | Tacc2 | -1,69 | 1,01546E-05 | 117,0201 | 110,1568 | 201,5475 | 417,3312 | 597,4029 | 371,9152 |
| ENSMUSG00000028717 | Tal1 | -1,77 | 7,52707E-13 | 5243,764 | 3236,098 | 3758,471 | 14053,45 | 13684,46 | 14098,97 |
| ENSMUSG00000028417 | Tal2 | -1,89 | 0,003164855 | 4,216939 | 12,56174 | 10,38013 | 36,67756 | 35,5144 | 29,36173 |
| ENSMUSG00000035168 | Tanc1 | -1,65 | 3,29461E-10 | 354,2229 | 233,8416 | 310,5388 | 788,0719 | 1048,943 | 978,7243 |
| ENSMUSG00000038213 | Tapbpl | 1,01 | 0,001229954 | 3653,978 | 2133,563 | 2252,488 | 1303,54 | 1262,029 | 1428,048 |
| ENSMUSG00000046985 | Tapt1 | -1,29 | 1,20954E-06 | 3666,628 | 2321,99 | 3037,917 | 8099,794 | 7681,257 | 6255,828 |
| ENSMUSG00000053338 | Tarm1 | -1,73 | 0,000107279 | 405,8804 | 273,4594 | 230,0928 | 888,1918 | 1495,41 | 623,7143 |
| ENSMUSG00000030515 | Tarsl2 | -1,05 | 6,59498E-07 | 459,6463 | 379,7511 | 419,5301 | 766,2637 | 988,0613 | 853,2696 |
| ENSMUSG00000040247 | Tbc1d10c | -1,34 | 6,92831E-11 | 2435,282 | 2486,259 | 2141,766 | 7098,595 | 5483,169 | 5348,283 |
| ENSMUSG00000039976 | Tbc1d16 | -1,18 | 0,005358853 | 143,3759 | 158,4712 | 186,8423 | 282,5164 | 270,1631 | 556,0934 |
| ENSMUSG00000052302 | Tbc1d30 | 1,52 | 0,001559427 | 186,5995 | 134,314 | 241,338 | 41,63399 | 58,34508 | 96,09293 |
| ENSMUSG00000033083 | Tbc1d4 | 1,38 | 0,000263412 | 1356,8 | 1696,801 | 1783,652 | 408,4096 | 547,9364 | 895,9776 |
| ENSMUSG00000037287 | Tbcel | -1,48 | 9,22718E-08 | 2808,481 | 2123,901 | 2593,302 | 8285,164 | 7616,57 | 5127,626 |
| ENSMUSG00000030699 | Tbx6 | -1,07 | 0,025061772 | 196,0877 | 230,9428 | 164,352 | 626,4924 | 362,7542 | 248,2401 |
| ENSMUSG00000034881 | Tbxa2r | -2,87 | 0,000823925 | 108,5862 | 68,60643 | 194,6274 | 344,9673 | 289,1887 | 2079,344 |
| ENSMUSG00000020167 | Tcf3 | -1,01 | 0,000412443 | 5169,967 | 4218,813 | 4902,015 | 12527,87 | 8756,836 | 7588,672 |
| ENSMUSG00000024985 | Tcf7l2 | -1,19 | 0,003870242 | 163,4064 | 126,5837 | 126,2915 | 262,6906 | 224,5017 | 463,5594 |
| ENSMUSG00000028523 | Tctex1d1 | 6,51 | 1,82835E-29 | 245,6367 | 467,6833 | 422,1252 | 5,947713 | 5,073485 | 1,779499 |
| ENSMUSG00000029386 | Tctn2 | -2,51 | 0,003382735 | 10,54235 | 5,797727 | 12,11015 | 24,78214 | 30,44091 | 106,7699 |
| ENSMUSG00000050052 | Tdrp | -3,95 | 3,24133E-06 | 3,162704 | 2,898863 | 5,190064 | 68,3987 | 19,02557 | 87,19544 |
| ENSMUSG00000055320 | Tead1 | -1,67 | 0,036180475 | 43,22362 | 23,19091 | 25,08531 | 30,72985 | 169,9618 | 89,86469 |
| ENSMUSG00000030796 | Tead2 | -1,13 | 0,049649166 | 40,06092 | 72,47159 | 36,33044 | 94,17212 | 72,29717 | 159,2651 |
| ENSMUSG00000006386 | Tek | -1,75 | 0,006316055 | 87,50148 | 51,21325 | 76,98594 | 125,8933 | 150,9362 | 449,3234 |
| ENSMUSG00000029359 | Tesc | -3,20 | 0,000539005 | 157,081 | 37,68522 | 46,71057 | 222,0479 | 306,9459 | 1683,406 |
| ENSMUSG00000034833 | Tespa1 | 1,69 | 5,78635E-12 | 1946,117 | 2081,384 | 1566,534 | 513,4859 | 513,6904 | 705,5712 |
| ENSMUSG00000009670 | Tex11 | -3,24 | 0,041837946 | 4,216939 | 0 | 1,730021 | 36,67756 | 2,536743 | 16,01549 |
| ENSMUSG00000009628 | Tex15 | -2,69 | 0,000890882 | 225,6062 | 19,32576 | 166,947 | 851,5142 | 1229,052 | 583,6756 |
| ENSMUSG00000090626 | Tex9 | -1,05 | 2,68824E-07 | 389,0126 | 419,3689 | 417,8001 | 809,8802 | 748,3391 | 978,7243 |
| ENSMUSG00000032411 | Tfdp2 | -2,46 | 2,4138E-12 | 4143,143 | 1968,328 | 2651,257 | 17908,56 | 17613,87 | 12548,14 |
| ENSMUSG00000029553 | Tfec | 2,08 | 8,7098E-08 | 4632,307 | 3911,533 | 4613,101 | 1088,431 | 1457,359 | 564,9908 |
| ENSMUSG00000029716 | Tfr2 | -2,68 | 2,71469E-17 | 328,9212 | 268,628 | 190,3023 | 1473,05 | 1414,234 | 2167,429 |
| ENSMUSG00000022797 | Tfrc | -2,13 | 5,74827E-08 | 9978,332 | 4044,881 | 7420,061 | 36065,94 | 33023,32 | 24784,86 |
| ENSMUSG00000053469 | Tg | -1,42 | 2,80791E-05 | 79,06761 | 69,57272 | 92,55613 | 207,1787 | 275,2366 | 163,7139 |
| ENSMUSG00000030782 | Tgfb1i1 | -2,92 | 0,000591555 | 300,4569 | 193,2576 | 205,8725 | 452,0262 | 645,601 | 4179,153 |
| ENSMUSG00000029287 | Tgfbr3 | -2,11 | 0,000188965 | 245,6367 | 99,52764 | 237,8779 | 462,9303 | 689,994 | 1369,324 |
| ENSMUSG00000022218 | Tgm1 | 1,42 | 0,001555594 | 1013,12 | 1066,782 | 1160,844 | 206,1874 | 403,3421 | 598,8013 |
| ENSMUSG00000037820 | Tgm2 | -1,80 | 0,002225259 | 1417,946 | 351,7288 | 757,7493 | 2521,83 | 4100,645 | 2150,524 |
| ENSMUSG00000027401 | Tgm3 | 3,78 | 3,19379E-16 | 1133,302 | 3078,593 | 1648,71 | 193,3007 | 139,5208 | 95,20318 |
| ENSMUSG00000078921 | Tgtp2 | 1,52 | 0,040673589 | 1772,169 | 715,053 | 431,6403 | 229,9782 | 206,7445 | 580,1166 |
| ENSMUSG00000040152 | Thbs1 | -3,65 | 0,011322145 | 18819,14 | 943,0969 | 4281,802 | 44812,05 | 189853,6 | 66288,11 |
| ENSMUSG00000023885 | Thbs2 | -2,44 | 0,001206057 | 36,89822 | 21,25833 | 44,98055 | 52,53813 | 168,6934 | 339,8843 |
| ENSMUSG00000055312 | Them7 | -1,93 | 0,042916704 | 9,488113 | 1,932576 | 6,055074 | 13,878 | 19,02557 | 32,92073 |
| ENSMUSG00000021779 | Thrb | -1,31 | 0,010603238 | 95,93536 | 36,71894 | 76,12093 | 225,0218 | 158,5464 | 133,4624 |
| ENSMUSG00000032289 | Thsd4 | -2,58 | 0,00169101 | 25,30163 | 19,32576 | 37,19546 | 62,45098 | 73,56554 | 354,1202 |
| ENSMUSG00000056130 | Ticam2 | 1,64 | 1,69563E-08 | 1475,929 | 1463,926 | 1700,611 | 656,231 | 464,2239 | 365,687 |
| ENSMUSG00000049625 | Tifab | 2,60 | 1,00549E-15 | 26065,95 | 32111,68 | 28151,77 | 6431,46 | 4800,786 | 3052,73 |
| ENSMUSG00000038028 | Tigar | 1,04 | 0,004075656 | 1101,675 | 639,6825 | 987,8421 | 443,1046 | 547,9364 | 338,9945 |
| ENSMUSG00000055546 | Timd4 | -2,34 | 5,85443E-06 | 31,62704 | 17,39318 | 25,08531 | 127,8758 | 69,76042 | 176,1704 |
| ENSMUSG00000017466 | Timp2 | -1,07 | 5,58642E-05 | 2421,577 | 3052,503 | 2737,759 | 5159,641 | 7279,183 | 4798,418 |
| ENSMUSG00000020044 | Timp3 | -2,68 | 0,000739747 | 703,1746 | 258,9651 | 721,4188 | 862,4184 | 2649,628 | 7310,181 |
| ENSMUSG00000028776 | Tinagl1 | -1,47 | 1,01443E-09 | 756,9405 | 1083,209 | 743,0441 | 2421,71 | 2186,672 | 2529,557 |
| ENSMUSG00000019437 | Tlcd1 | -2,47 | 4,03827E-19 | 219,2808 | 152,6735 | 150,5118 | 1154,848 | 941,1315 | 798,1052 |
| ENSMUSG00000045322 | Tlr9 | 1,04 | 0,001735098 | 2389,95 | 4031,353 | 3164,209 | 1887,408 | 1204,953 | 1552,613 |
| ENSMUSG00000027800 | Tm4sf1 | -2,02 | 0,004650151 | 65,36255 | 34,78636 | 72,66089 | 66,41613 | 303,1407 | 330,9868 |
| ENSMUSG00000079625 | Tm4sf19 | -3,69 | 3,30293E-06 | 9,488113 | 2,898863 | 6,055074 | 36,67756 | 55,80834 | 143,2496 |
| ENSMUSG00000050106 | Tmc8 | -1,14 | 2,05005E-05 | 1012,065 | 1280,331 | 942,8615 | 2873,737 | 2291,947 | 1942,323 |
| ENSMUSG00000042066 | Tmcc2 | -3,11 | 3,86419E-11 | 8141,855 | 2921,088 | 4176,271 | 58571,09 | 44327,04 | 28317,16 |
| ENSMUSG00000078577 | Tmco2 | 3,15 | 0,002077671 | 22,13893 | 57,97727 | 34,60042 | 6,938998 | 6,341857 | 0 |
| ENSMUSG00000034659 | Tmem109 | 1,40 | 9,04304E-13 | 4624,928 | 4315,441 | 5293,865 | 1783,323 | 2029,394 | 1576,636 |
| ENSMUSG00000054675 | Tmem119 | -1,45 | 9,33722E-10 | 90,66419 | 77,30302 | 97,7462 | 232,9521 | 258,7478 | 234,0041 |
| ENSMUSG00000054434 | Tmem120b | -2,86 | 2,67965E-09 | 220,3351 | 71,5053 | 95,15116 | 1087,44 | 1010,892 | 704,6815 |
| ENSMUSG00000020701 | Tmem132e | -1,65 | 0,0406608 | 132,8336 | 70,53901 | 110,7214 | 542,2331 | 375,4379 | 71,17995 |
| ENSMUSG00000048503 | Tmem136 | -3,58 | 3,71577E-06 | 4,216939 | 1,932576 | 1,730021 | 30,72985 | 30,44091 | 31,14123 |
| ENSMUSG00000071506 | Tmem139 | 1,65 | 0,028120865 | 20,03046 | 21,25833 | 16,4352 | 5,947713 | 5,073485 | 7,117995 |
| ENSMUSG00000057137 | Tmem140 | -1,10 | 0,000145294 | 407,9888 | 293,7515 | 264,6932 | 764,2811 | 577,109 | 726,9252 |
| ENSMUSG00000027956 | Tmem144 | 1,18 | 0,00577731 | 61,14562 | 92,76363 | 109,8563 | 35,68628 | 36,78277 | 43,59772 |
| ENSMUSG00000046456 | Tmem150b | 2,55 | 7,79564E-28 | 1460,115 | 1632,06 | 1508,578 | 318,2026 | 263,8212 | 204,6424 |
| ENSMUSG00000096847 | Tmem151b | -2,84 | 0,01679995 | 13,70505 | 3,865151 | 43,25053 | 33,70371 | 63,41857 | 338,9945 |
| ENSMUSG00000056498 | Tmem154 | -1,05 | 0,000568091 | 2073,68 | 3515,355 | 2205,777 | 5959,608 | 5058,265 | 5158,767 |
| ENSMUSG00000037913 | Tmem156 | 1,34 | 3,06679E-15 | 1568,701 | 1441,701 | 1430,728 | 649,292 | 560,6201 | 541,8574 |
| ENSMUSG00000054871 | Tmem158 | -2,88 | 0,002818803 | 33,73551 | 27,05606 | 35,46543 | 52,53813 | 76,10228 | 577,4473 |
| ENSMUSG00000030917 | Tmem159 | 1,17 | 1,44125E-11 | 770,6456 | 867,7264 | 847,7104 | 341,9935 | 370,3644 | 393,2692 |
| ENSMUSG00000026188 | Tmem169 | -4,54 | 0,00033609 | 2,108469 | 4,831439 | 0 | 17,84314 | 21,56231 | 119,2264 |
| ENSMUSG00000024349 | Tmem173 | 1,46 | 1,69062E-07 | 8892,47 | 5304,92 | 6365,613 | 2189,75 | 2729,535 | 2578,494 |
| ENSMUSG00000024245 | Tmem178 | 5,57 | 2,95124E-56 | 1758,464 | 2772,28 | 3081,168 | 47,5817 | 38,05114 | 72,95945 |
| ENSMUSG00000093880 | Tmem181c-ps | 1,20 | 4,69955E-05 | 575,6122 | 834,8727 | 722,2838 | 262,6906 | 389,39 | 276,7121 |
| ENSMUSG00000036687 | Tmem184a | -3,15 | 0,000394818 | 5,271174 | 4,831439 | 5,190064 | 11,89543 | 55,80834 | 68,5107 |
| ENSMUSG00000049526 | Tmem202 | -1,15 | 0,047130491 | 11,59658 | 23,19091 | 19,03023 | 49,56427 | 35,5144 | 34,70023 |
| ENSMUSG00000024168 | Tmem204 | -1,36 | 0,007133584 | 68,52526 | 45,41553 | 49,3056 | 81,28541 | 134,4474 | 201,9731 |
| ENSMUSG00000046593 | Tmem215 | 2,36 | 3,99287E-05 | 59,03715 | 68,60643 | 92,55613 | 5,947713 | 16,48883 | 20,46424 |
| ENSMUSG00000032121 | Tmem218 | 1,11 | 1,49151E-05 | 342,6263 | 350,7625 | 360,7094 | 127,8758 | 171,2301 | 190,4064 |
| ENSMUSG00000046157 | Tmem229b | 1,32 | 5,41033E-07 | 12578,07 | 15065,39 | 11454,47 | 5374,75 | 3999,175 | 6254,938 |
| ENSMUSG00000031951 | Tmem231 | -2,59 | 1,74719E-09 | 40,06092 | 22,22462 | 22,49028 | 115,9804 | 205,4762 | 186,8474 |
| ENSMUSG00000061531 | Tmem236 | 4,16 | 7,32867E-10 | 48,4948 | 91,79734 | 59,68573 | 2,973856 | 2,536743 | 5,338496 |
| ENSMUSG00000030431 | Tmem238 | -1,07 | 0,014342328 | 37,95245 | 56,04469 | 57,95571 | 89,21569 | 87,51762 | 141,4701 |
| ENSMUSG00000039611 | Tmem246 | -2,63 | 0,000329377 | 33,73551 | 17,39318 | 31,14038 | 75,3377 | 92,59111 | 340,774 |
| ENSMUSG00000060044 | Tmem26 | -1,76 | 0,000504345 | 117,0201 | 99,52764 | 197,2224 | 337,0371 | 324,7031 | 743,8305 |
| ENSMUSG00000050777 | Tmem37 | -1,95 | 0,00024364 | 27,4101 | 17,39318 | 27,68034 | 59,47713 | 79,90739 | 139,6907 |
| ENSMUSG00000059900 | Tmem40 | -2,19 | 2,34404E-05 | 899,2622 | 869,659 | 847,7104 | 2259,14 | 2425,126 | 7244,339 |
| ENSMUSG00000022537 | Tmem44 | -1,80 | 0,00229278 | 10,54235 | 19,32576 | 19,03023 | 45,59913 | 40,58788 | 83,63644 |
| ENSMUSG00000028132 | Tmem56 | -3,73 | 7,34788E-10 | 270,9383 | 50,24697 | 83,90603 | 1996,449 | 1924,119 | 1460,968 |
| ENSMUSG00000043252 | Tmem64 | -1,45 | 5,07585E-05 | 778,0252 | 361,3916 | 674,7083 | 1815,044 | 1614,637 | 1531,259 |
| ENSMUSG00000045282 | Tmem86b | -1,08 | 0,000303244 | 585,1003 | 576,8738 | 394,4448 | 1296,601 | 1103,483 | 885,3006 |
| ENSMUSG00000026411 | Tmem9 | -1,36 | 1,10133E-07 | 764,3202 | 496,6719 | 665,1931 | 1426,46 | 1756,694 | 1774,16 |
| ENSMUSG00000035413 | Tmem98 | -1,58 | 0,024629952 | 74,85067 | 23,19091 | 62,28076 | 113,0065 | 96,39622 | 268,7043 |
| ENSMUSG00000049555 | Tmie | -3,06 | 0,00208746 | 6,325408 | 7,730302 | 5,190064 | 26,76471 | 15,22046 | 117,4469 |
| ENSMUSG00000028328 | Tmod1 | -3,08 | 6,15378E-10 | 445,9413 | 113,0557 | 211,0626 | 2288,878 | 2198,088 | 2007,275 |
| ENSMUSG00000079523 | Tmsb10 | 1,53 | 4,68678E-13 | 24443,49 | 17701,43 | 20955,75 | 8181,079 | 6888,525 | 6828,826 |
| ENSMUSG00000030306 | Tmtc1 | -1,72 | 0,002535995 | 17,92199 | 21,25833 | 23,35529 | 59,47713 | 40,58788 | 104,9904 |
| ENSMUSG00000028364 | Tnc | -1,17 | 0,041980114 | 741,127 | 603,9299 | 884,0408 | 774,1939 | 1431,991 | 2792,034 |
| ENSMUSG00000068105 | Tnfrsf13c | -2,26 | 0,000417452 | 572,4495 | 114,022 | 343,4092 | 2417,745 | 1471,311 | 1054,353 |
| ENSMUSG00000060548 | Tnfrsf19 | -1,62 | 0,002344101 | 334,1924 | 116,9208 | 232,6878 | 1005,163 | 635,454 | 459,1107 |
| ENSMUSG00000037613 | Tnfrsf23 | -1,42 | 0,000256838 | 103,315 | 125,6174 | 96,88119 | 309,2811 | 383,0481 | 183,2884 |
| ENSMUSG00000045362 | Tnfrsf26 | -1,12 | 2,95065E-06 | 279,3722 | 211,617 | 248,258 | 537,2767 | 602,4764 | 463,5594 |
| ENSMUSG00000028602 | Tnfrsf8 | 2,21 | 7,75261E-05 | 104,3692 | 323,7064 | 155,7019 | 55,51199 | 38,05114 | 32,92073 |
| ENSMUSG00000028965 | Tnfrsf9 | 1,11 | 0,049114319 | 150,7556 | 76,33674 | 108,1263 | 63,44227 | 27,90417 | 63,17221 |
| ENSMUSG00000097328 | Tnfsf12 | 2,07 | 5,1986E-17 | 771,6998 | 743,0753 | 982,652 | 181,4052 | 173,7669 | 236,6733 |
| ENSMUSG00000035678 | Tnfsf9 | 1,35 | 0,00026745 | 287,8061 | 152,6735 | 243,068 | 79,30284 | 87,51762 | 101,4314 |
| ENSMUSG00000027692 | Tnik | -2,26 | 0,001308837 | 571,3952 | 349,7962 | 461,0506 | 907,0262 | 1059,09 | 4652,499 |
| ENSMUSG00000044162 | Tnip3 | 3,34 | 0,000289541 | 227,7147 | 786,5583 | 373,6846 | 9,912855 | 19,02557 | 107,6597 |
| ENSMUSG00000001583 | Tnk1 | -2,47 | 8,64755E-09 | 21,08469 | 14,49432 | 12,97516 | 111,024 | 79,90739 | 76,51845 |
| ENSMUSG00000022791 | Tnk2 | -1,94 | 7,46734E-13 | 1776,386 | 1097,703 | 1126,244 | 5516,504 | 4738,635 | 5065,343 |
| ENSMUSG00000075265 | Tnk2os | -2,56 | 0,000526994 | 8,433878 | 6,764015 | 3,460042 | 53,52942 | 32,97765 | 22,24373 |
| ENSMUSG00000031097 | Tnni2 | 1,85 | 4,45208E-18 | 511,3038 | 537,256 | 484,4059 | 150,6754 | 116,6902 | 154,8164 |
| ENSMUSG00000061723 | Tnnt3 | 1,97 | 0,003363503 | 192,925 | 274,4257 | 98,61121 | 46,59042 | 19,02557 | 77,40819 |
| ENSMUSG00000037003 | Tns2 | -1,04 | 0,002005738 | 117,0201 | 127,55 | 158,2969 | 220,0654 | 252,4059 | 355,01 |
| ENSMUSG00000017607 | Tns4 | 3,09 | 1,05835E-44 | 937,2147 | 1085,141 | 1011,197 | 142,7451 | 112,885 | 100,5417 |
| ENSMUSG00000037573 | Tob1 | -1,09 | 0,001138601 | 934,052 | 658,042 | 754,2892 | 2050,97 | 1776,988 | 1154,005 |
| ENSMUSG00000020541 | Tom1l1 | -2,55 | 1,54639E-15 | 421,6939 | 241,572 | 313,9988 | 1607,865 | 1655,225 | 2455,708 |
| ENSMUSG00000060519 | Tor3a | 1,31 | 8,17434E-09 | 3360,9 | 3341,423 | 3509,348 | 1281,732 | 1144,071 | 1698,532 |
| ENSMUSG00000074607 | Tox2 | 4,13 | 5,23708E-11 | 402,7177 | 1321,882 | 610,6975 | 21,80828 | 31,70928 | 79,18769 |
| ENSMUSG00000035274 | Tpbg | -1,85 | 0,033384008 | 7,379643 | 3,865151 | 3,460042 | 12,88671 | 22,83068 | 16,90524 |
| ENSMUSG00000094594 | Tpd52-ps | 1,89 | 0,038971653 | 11,59658 | 19,32576 | 15,57019 | 2,973856 | 6,341857 | 3,558997 |
| ENSMUSG00000032366 | Tpm1 | -1,61 | 0,001969956 | 2265,55 | 2026,306 | 2365,804 | 3757,963 | 4561,063 | 12008,06 |
| ENSMUSG00000028464 | Tpm2 | -4,15 | 4,94973E-07 | 171,8403 | 108,2242 | 98,61121 | 554,1286 | 813,026 | 5335,827 |
| ENSMUSG00000021573 | Tppp | -2,70 | 2,18611E-07 | 65,36255 | 26,08977 | 27,68034 | 222,0479 | 371,6328 | 178,8396 |
| ENSMUSG00000033825 | Tpsb2 | -10,21 | 0,005086144 | 0 | 0 | 0 | 2,973856 | 1,268371 | 121,0059 |
| ENSMUSG00000017386 | Traf4 | -2,06 | 4,74349E-09 | 488,1107 | 213,5496 | 343,4092 | 1552,353 | 1330,522 | 1478,763 |
| ENSMUSG00000042726 | Trafd1 | 1,04 | 3,67081E-05 | 8699,545 | 8074,301 | 7235,814 | 3982,985 | 3004,772 | 4651,61 |
| ENSMUSG00000026028 | Trak2 | -1,93 | 4,125E-10 | 9239,313 | 5064,314 | 6669,232 | 30185,63 | 28450,84 | 21504,35 |
| ENSMUSG00000042265 | Trem1 | -1,66 | 1,93553E-05 | 2413,143 | 1593,409 | 1621,895 | 5317,255 | 8490,478 | 3938,92 |
| ENSMUSG00000023992 | Trem2 | 1,12 | 3,68001E-05 | 1284,058 | 1537,364 | 948,9166 | 548,1809 | 550,4732 | 631,7221 |
| ENSMUSG00000041754 | Trem3 | -1,43 | 7,24358E-06 | 2042,053 | 2129,698 | 1485,223 | 5360,872 | 6321,563 | 3575,903 |
| ENSMUSG00000023993 | Treml1 | -3,06 | 0,022897667 | 710,5542 | 487,0091 | 797,5398 | 1088,431 | 1826,455 | 13674,56 |
| ENSMUSG00000051682 | Treml4 | -2,25 | 5,3599E-09 | 245,6367 | 341,0996 | 209,3326 | 1214,325 | 809,2209 | 1773,27 |
| ENSMUSG00000032501 | Trib1 | -1,10 | 0,048205131 | 1774,277 | 765,2999 | 1151,329 | 2072,778 | 4151,379 | 1696,752 |
| ENSMUSG00000020601 | Trib2 | -1,99 | 0,017104746 | 1348,366 | 172,9655 | 567,4469 | 3636,035 | 3512,12 | 1145,997 |
| ENSMUSG00000043496 | Tril | -3,08 | 0,011895846 | 1,054235 | 2,898863 | 0,865011 | 8,921569 | 10,14697 | 21,35398 |
| ENSMUSG00000073400 | Trim10 | -3,51 | 8,50175E-09 | 2904,417 | 505,3685 | 1038,878 | 19014,84 | 18127,56 | 13541,99 |
| ENSMUSG00000050747 | Trim15 | -1,48 | 0,045959055 | 21,08469 | 12,56174 | 6,920085 | 51,54684 | 35,5144 | 24,91298 |
| ENSMUSG00000027993 | Trim2 | -2,62 | 1,18155E-08 | 137,0505 | 64,74128 | 84,77104 | 688,9434 | 748,3391 | 328,3175 |
| ENSMUSG00000032013 | Trim29 | -2,54 | 0,025345645 | 0 | 4,831439 | 12,97516 | 31,72113 | 50,73485 | 23,13348 |
| ENSMUSG00000078616 | Trim30c | 1,42 | 0,001044952 | 303,6196 | 238,6731 | 150,5118 | 60,46841 | 106,5432 | 91,64418 |
| ENSMUSG00000090693 | Trim43a | -3,59 | 0,038036608 | 0 | 0,966288 | 0,865011 | 9,912855 | 7,610228 | 5,338496 |
| ENSMUSG00000033233 | Trim45 | 1,27 | 0,001840011 | 824,4116 | 827,1424 | 848,5754 | 397,5055 | 454,0769 | 187,7371 |
| ENSMUSG00000020773 | Trim47 | -1,97 | 0,006302898 | 85,39301 | 78,26931 | 84,77104 | 126,8845 | 175,0352 | 672,6505 |
| ENSMUSG00000037124 | Trim58 | -3,43 | 1,04512E-10 | 377,416 | 87,93219 | 153,1069 | 2386,024 | 2387,075 | 1877,371 |
| ENSMUSG00000034317 | Trim59 | -1,30 | 0,000184152 | 3686,659 | 2047,564 | 2433,275 | 8205,861 | 6637,387 | 5231,726 |
| ENSMUSG00000041000 | Trim62 | 1,11 | 0,013942724 | 236,1486 | 209,6845 | 257,7732 | 92,18955 | 68,49205 | 164,6036 |
| ENSMUSG00000073968 | Trim68 | 1,15 | 0,007351349 | 569,2868 | 1126,692 | 562,2569 | 393,5403 | 260,0161 | 363,0177 |
| ENSMUSG00000040350 | Trim7 | -2,02 | 6,56948E-05 | 105,4235 | 82,13446 | 61,41575 | 364,7931 | 161,0832 | 479,5749 |
| ENSMUSG00000022263 | Trio | 1,52 | 1,70562E-08 | 3223,85 | 4673,934 | 5393,341 | 1702,037 | 1489,068 | 1444,063 |
| ENSMUSG00000028331 | Trmo | -1,34 | 0,000660872 | 775,9168 | 385,5488 | 623,6726 | 1976,623 | 1178,317 | 1360,427 |
| ENSMUSG00000018199 | Trove2 | -1,77 | 0,012190233 | 404,8261 | 311,1447 | 386,6597 | 616,5796 | 540,3262 | 2596,289 |
| ENSMUSG00000068735 | Trp53i11 | -1,48 | 0,001098311 | 1251,377 | 502,4697 | 1039,743 | 3297,015 | 2622,992 | 1865,804 |
| ENSMUSG00000038375 | Trp53inp2 | -2,31 | 3,10531E-11 | 2852,759 | 1885,228 | 2100,246 | 14774,12 | 11767,95 | 7255,016 |
| ENSMUSG00000032839 | Trpc1 | -2,93 | 0,009133513 | 22,13893 | 1,932576 | 8,650106 | 31,72113 | 34,24603 | 181,5089 |
| ENSMUSG00000031997 | Trpc6 | -2,11 | 0,028647762 | 217,1724 | 240,6057 | 260,3682 | 264,6732 | 281,5784 | 2551,801 |
| ENSMUSG00000038679 | Trps1 | 1,13 | 9,35408E-09 | 2660,888 | 2379,001 | 2516,316 | 1273,802 | 1208,758 | 961,8191 |
| ENSMUSG00000036899 | Trpv5 | -3,15 | 0,000406651 | 2,108469 | 1,932576 | 5,190064 | 29,73856 | 39,31951 | 15,12574 |
| ENSMUSG00000022010 | Tsc22d1 | -1,70 | 0,014609171 | 2669,322 | 2959,74 | 3028,402 | 4197,103 | 4419,006 | 19558,47 |
| ENSMUSG00000020963 | Tshr | -1,45 | 0,000335137 | 52,71174 | 74,40416 | 86,50106 | 213,1264 | 239,7222 | 131,6829 |
| ENSMUSG00000021217 | Tshz3 | -1,43 | 0,006687855 | 14,75929 | 11,59545 | 19,03023 | 33,70371 | 41,85625 | 47,15672 |
| ENSMUSG00000049580 | Tsku | -2,38 | 0,009898874 | 5,271174 | 9,662878 | 16,4352 | 21,80828 | 32,97765 | 109,4392 |
| ENSMUSG00000031893 | Tsnaxip1 | -1,40 | 0,01720818 | 11,59658 | 11,59545 | 11,24514 | 37,66885 | 25,36743 | 27,58223 |
| ENSMUSG00000029669 | Tspan12 | -2,01 | 0,025652456 | 9,488113 | 3,865151 | 12,97516 | 18,83442 | 24,09906 | 63,17221 |
| ENSMUSG00000020577 | Tspan13 | -1,66 | 1,32418E-06 | 2109,524 | 1090,939 | 1443,703 | 4431,046 | 4158,99 | 6097,452 |
| ENSMUSG00000037031 | Tspan15 | -2,09 | 7,16943E-05 | 56,92868 | 15,4606 | 44,11554 | 141,7538 | 188,9873 | 166,3831 |
| ENSMUSG00000025875 | Tspan17 | 1,61 | 0,001869227 | 844,442 | 983,681 | 698,0635 | 152,658 | 197,8659 | 474,2364 |
| ENSMUSG00000027217 | Tspan18 | -1,89 | 0,025610108 | 26,35587 | 20,29204 | 14,70518 | 38,66013 | 32,97765 | 154,8164 |
| ENSMUSG00000027858 | Tspan2 | -1,81 | 9,28086E-26 | 490,2192 | 441,5935 | 445,4805 | 1474,041 | 1577,854 | 1790,176 |
| ENSMUSG00000006736 | Tspan31 | 1,07 | 1,18417E-08 | 1905,002 | 1725,79 | 1859,773 | 759,3247 | 867,566 | 983,1731 |
| ENSMUSG00000000244 | Tspan32 | -1,58 | 1,49307E-05 | 1598,22 | 1695,835 | 1882,263 | 3553,758 | 4247,776 | 7639,388 |
| ENSMUSG00000052281 | Tspan32os | -1,39 | 0,004179359 | 31,62704 | 72,47159 | 36,33044 | 145,719 | 102,7381 | 118,3367 |
| ENSMUSG00000001763 | Tspan33 | -3,19 | 1,32096E-10 | 2435,282 | 634,8511 | 1127,974 | 14101,04 | 13456,15 | 10829,14 |
| ENSMUSG00000058254 | Tspan7 | -1,11 | 0,035634514 | 114,9116 | 58,94356 | 105,5313 | 120,9368 | 200,4027 | 282,9403 |
| ENSMUSG00000034127 | Tspan8 | -3,74 | 4,94233E-10 | 235,0943 | 42,51666 | 80,44598 | 1785,305 | 1728,79 | 1267,893 |
| ENSMUSG00000030352 | Tspan9 | -3,85 | 2,58047E-06 | 153,9183 | 37,68522 | 84,77104 | 408,4096 | 658,2847 | 2913,929 |
| ENSMUSG00000023995 | Tspo2 | -3,44 | 5,16466E-09 | 682,0899 | 171,9992 | 195,4924 | 5236,961 | 3093,558 | 3068,746 |
| ENSMUSG00000039485 | Tspyl4 | -1,02 | 0,005982368 | 154,9725 | 96,62878 | 107,2613 | 221,0567 | 197,8659 | 305,184 |
| ENSMUSG00000000411 | Tssk3 | -2,13 | 0,005916995 | 11,59658 | 3,865151 | 5,190064 | 42,62527 | 22,83068 | 24,02323 |
| ENSMUSG00000044986 | Tst | -1,07 | 1,72735E-05 | 636,7578 | 556,5818 | 570,907 | 1306,514 | 1449,748 | 960,9293 |
| ENSMUSG00000040219 | Ttc12 | 3,11 | 3,89544E-33 | 553,4732 | 787,5246 | 702,3886 | 73,35512 | 71,02879 | 91,64418 |
| ENSMUSG00000039021 | Ttc16 | -2,42 | 0,007920035 | 6,325408 | 8,69659 | 7,785095 | 27,75599 | 13,95208 | 80,07744 |
| ENSMUSG00000032514 | Ttc21a | -2,56 | 1,42244E-06 | 84,33878 | 64,74128 | 70,06586 | 536,2854 | 591,061 | 165,4934 |
| ENSMUSG00000034848 | Ttc21b | 1,19 | 5,37502E-11 | 729,5304 | 679,3003 | 727,4739 | 310,2724 | 281,5784 | 343,4433 |
| ENSMUSG00000028555 | Ttc39a | -2,35 | 1,13775E-10 | 57,98291 | 40,58409 | 30,27537 | 267,6471 | 192,7924 | 193,0756 |
| ENSMUSG00000085873 | Ttc39aos1 | -3,31 | 8,37622E-06 | 3,162704 | 10,62917 | 5,190064 | 102,1024 | 50,73485 | 35,58997 |
| ENSMUSG00000038172 | Ttc39b | -2,04 | 7,56509E-08 | 637,812 | 297,6166 | 407,42 | 1521,623 | 1629,857 | 2380,08 |
| ENSMUSG00000024424 | Ttc39c | 1,30 | 5,74406E-07 | 784,3506 | 642,5814 | 848,5754 | 358,8453 | 318,3612 | 244,6811 |
| ENSMUSG00000027394 | Ttl | 1,06 | 6,3727E-06 | 702,1203 | 541,1212 | 601,1824 | 270,6209 | 273,9682 | 339,8843 |
| ENSMUSG00000022442 | Ttll1 | -1,28 | 9,51057E-05 | 280,4264 | 211,617 | 261,2332 | 499,6079 | 502,275 | 819,4592 |
| ENSMUSG00000026885 | Ttll11 | -4,10 | 7,30159E-06 | 44,27786 | 17,39318 | 19,89524 | 120,9368 | 149,6678 | 1129,092 |
| ENSMUSG00000016757 | Ttll12 | -1,07 | 8,92036E-05 | 2087,385 | 1239,747 | 1673,795 | 3550,785 | 3640,226 | 3301,86 |
| ENSMUSG00000036745 | Ttll7 | -3,55 | 3,78923E-05 | 8,433878 | 8,69659 | 8,650106 | 27,75599 | 60,88182 | 212,6501 |
| ENSMUSG00000034714 | Ttyh2 | -1,97 | 7,33466E-06 | 131,7793 | 67,64015 | 172,1371 | 355,8715 | 508,6169 | 589,0141 |
| ENSMUSG00000016255 | Tubb1 | -4,80 | 0,001660396 | 713,7169 | 51,21325 | 153,9719 | 2280,948 | 3140,487 | 20110,12 |
| ENSMUSG00000045136 | Tubb2b | -1,58 | 0,024629952 | 137,0505 | 23,19091 | 62,28076 | 249,8039 | 204,2078 | 208,2014 |
| ENSMUSG00000023467 | Tulp2 | -1,50 | 0,008422904 | 20,03046 | 28,98863 | 21,62526 | 101,1111 | 54,53997 | 43,59772 |
| ENSMUSG00000097929 | Tunar | 7,67 | 0,030769955 | 4,216939 | 7,730302 | 11,24514 | 0 | 0 | 0 |
| ENSMUSG00000046275 | Tusc5 | -1,69 | 0,010670289 | 9,488113 | 11,59545 | 6,920085 | 21,80828 | 27,90417 | 40,03872 |
| ENSMUSG00000075704 | Txnrd2 | -1,93 | 2,41713E-20 | 1031,042 | 795,2549 | 901,341 | 3887,822 | 3543,83 | 2975,322 |
| ENSMUSG00000032175 | Tyk2 | 1,05 | 7,15303E-09 | 7521,965 | 9628,092 | 8181,27 | 4245,676 | 3860,922 | 4160,468 |
| ENSMUSG00000034485 | Uaca | -1,28 | 0,000334802 | 129,6709 | 73,43787 | 148,7818 | 279,5425 | 294,2622 | 282,9403 |
| ENSMUSG00000036352 | Ubac1 | -2,24 | 2,58804E-15 | 2757,878 | 1520,937 | 1954,924 | 10104,17 | 10424,74 | 8843,219 |
| ENSMUSG00000001403 | Ube2c | -1,09 | 4,53515E-07 | 4884,27 | 3526,95 | 4044,789 | 9024,663 | 9586,351 | 7961,477 |
| ENSMUSG00000058317 | Ube2e2 | -1,13 | 0,044472098 | 35,84398 | 57,01098 | 83,90603 | 90,20698 | 112,885 | 185,0679 |
| ENSMUSG00000027011 | Ube2e3 | -1,07 | 2,88745E-08 | 1542,345 | 1181,77 | 1399,587 | 2889,597 | 2688,947 | 3093,659 |
| ENSMUSG00000027078 | Ube2l6 | -3,20 | 6,06965E-16 | 4797,822 | 1728,689 | 2737,759 | 29386,66 | 27065,78 | 28436,39 |
| ENSMUSG00000020802 | Ube2o | -2,79 | 6,86058E-24 | 3445,239 | 1912,284 | 2305,253 | 18426,01 | 16685,42 | 17755,84 |
| ENSMUSG00000041231 | Ublcp1 | -1,43 | 1,54906E-11 | 578,7749 | 766,2662 | 654,813 | 1973,649 | 1841,675 | 1574,856 |
| ENSMUSG00000026558 | Uck2 | 1,53 | 7,31173E-08 | 5523,136 | 4219,779 | 5519,633 | 1473,05 | 2305,899 | 1511,684 |
| ENSMUSG00000032942 | Ucp3 | -1,93 | 0,005982368 | 6,325408 | 10,62917 | 28,54535 | 59,47713 | 67,22368 | 48,93622 |
| ENSMUSG00000051502 | Ufsp1 | -2,89 | 9,70976E-06 | 119,1285 | 183,5947 | 98,61121 | 600,719 | 397,0002 | 1969,015 |
| ENSMUSG00000037470 | Uggt1 | 1,01 | 4,68424E-05 | 17733,28 | 16974,78 | 16255,28 | 9359,717 | 9554,641 | 6317,221 |
| ENSMUSG00000054545 | Ugt1a6a | 1,68 | 3,74054E-06 | 168,6776 | 320,8076 | 205,8725 | 76,32898 | 69,76042 | 70,2902 |
| ENSMUSG00000090124 | Ugt1a7c | 1,87 | 1,75422E-17 | 2752,607 | 3761,758 | 3786,151 | 1039,858 | 868,8344 | 917,3316 |
| ENSMUSG00000032854 | Ugt8a | -3,28 | 2,41694E-06 | 30,57281 | 5,797727 | 10,38013 | 195,2832 | 175,0352 | 81,85694 |
| ENSMUSG00000054134 | Umodl1 | -5,00 | 0,036362855 | 0 | 0 | 0,865011 | 15,86057 | 1,268371 | 12,45649 |
| ENSMUSG00000043592 | Unc5cl | -2,03 | 2,3058E-06 | 104,3692 | 53,14583 | 47,57558 | 352,8976 | 252,4059 | 228,6656 |
| ENSMUSG00000006143 | Upk3bl | 1,79 | 0,028936356 | 21,08469 | 16,42689 | 20,76025 | 6,938998 | 7,610228 | 2,669248 |
| ENSMUSG00000026839 | Upp2 | 2,86 | 2,39003E-07 | 45,33209 | 60,87613 | 49,3056 | 4,956427 | 6,341857 | 9,787243 |
| ENSMUSG00000028684 | Urod | -1,98 | 9,17947E-16 | 3637,11 | 2233,091 | 2726,513 | 11821,08 | 11311,34 | 10733,05 |
| ENSMUSG00000030979 | Uros | -2,38 | 3,75889E-14 | 1178,634 | 690,8958 | 696,3335 | 4465,741 | 5234,569 | 3636,406 |
| ENSMUSG00000030838 | Ush1c | -2,88 | 0,022649802 | 0 | 3,865151 | 2,595032 | 6,938998 | 16,48883 | 24,91298 |
| ENSMUSG00000000804 | Usp32 | -1,48 | 4,81032E-11 | 4565,891 | 3372,344 | 3225,624 | 11144,03 | 10710,13 | 9377,069 |
| ENSMUSG00000054814 | Usp46 | -1,31 | 1,48656E-10 | 1559,213 | 1132,489 | 1441,973 | 3214,739 | 3423,334 | 3592,808 |
| ENSMUSG00000073002 | Vamp5 | -1,76 | 1,67934E-13 | 598,8053 | 642,5814 | 484,4059 | 1846,765 | 1660,298 | 2325,805 |
| ENSMUSG00000027860 | Vangl1 | -3,39 | 7,88566E-07 | 207,6842 | 25,12348 | 73,5259 | 1112,222 | 1202,416 | 887,9699 |
| ENSMUSG00000021256 | Vash1 | -2,31 | 0,005683608 | 22,13893 | 10,62917 | 15,57019 | 27,75599 | 53,2716 | 157,4856 |
| ENSMUSG00000027962 | Vcam1 | -2,03 | 1,23918E-05 | 3174,301 | 2066,89 | 4211,737 | 7895,589 | 10820,48 | 19995,34 |
| ENSMUSG00000031380 | Vegfd | -1,76 | 0,002724357 | 12,65082 | 18,35947 | 12,97516 | 69,38998 | 30,44091 | 48,04647 |
| ENSMUSG00000026728 | Vim | 1,06 | 1,27444E-05 | 58262,28 | 60519,57 | 62479,71 | 32284,19 | 32734,13 | 21883,39 |
| ENSMUSG00000032528 | Vipr1 | -1,42 | 0,000269039 | 46,38633 | 71,5053 | 59,68573 | 165,5447 | 111,6167 | 198,4141 |
| ENSMUSG00000011171 | Vipr2 | -1,40 | 0,023836586 | 18,97623 | 25,12348 | 25,95032 | 41,63399 | 43,12463 | 99,65193 |
| ENSMUSG00000096630 | Vmn2r26 | 9,98 | 1,48695E-74 | 3474,758 | 9771,102 | 5595,753 | 5,947713 | 7,610228 | 5,338496 |
| ENSMUSG00000070601 | Vmn2r84 | 1,39 | 0,005780113 | 95,93536 | 97,59507 | 55,36068 | 30,72985 | 21,56231 | 41,81822 |
| ENSMUSG00000020010 | Vnn3 | -2,23 | 3,23719E-09 | 146,5386 | 90,83105 | 115,9114 | 790,0545 | 443,93 | 425,3002 |
| ENSMUSG00000037788 | Vopp1 | -2,18 | 1,99218E-09 | 2155,91 | 1105,433 | 995,6272 | 6552,397 | 6931,649 | 5824,299 |
| ENSMUSG00000059305 | Vpreb1 | -1,52 | 0,02073556 | 439,6159 | 109,1905 | 351,1943 | 1200,447 | 889,1283 | 487,5827 |
| ENSMUSG00000059280 | Vpreb2 | -1,71 | 0,003954667 | 32,68128 | 9,662878 | 32,00539 | 97,14598 | 81,17577 | 65,84145 |
| ENSMUSG00000035284 | Vps13c | 2,02 | 8,99738E-24 | 12854,28 | 16997 | 13643,81 | 3718,312 | 3852,044 | 3183,523 |
| ENSMUSG00000001943 | Vsig2 | -3,33 | 4,95616E-06 | 8,433878 | 19,32576 | 19,89524 | 162,5708 | 48,19811 | 269,5941 |
| ENSMUSG00000044206 | Vsig4 | -3,84 | 0,001241374 | 2,108469 | 3,865151 | 2,595032 | 55,51199 | 3,805114 | 63,17221 |
| ENSMUSG00000050666 | Vstm4 | -2,58 | 0,000203434 | 28,46434 | 22,22462 | 47,57558 | 124,902 | 87,51762 | 375,4742 |
| ENSMUSG00000023186 | Vwa5a | 1,16 | 0,000372044 | 3618,134 | 2573,224 | 3765,391 | 1080,501 | 1501,752 | 1870,253 |
| ENSMUSG00000028753 | Vwa5b1 | -5,77 | 0,004650151 | 0 | 0 | 0,865011 | 28,74728 | 16,48883 | 6,228246 |
| ENSMUSG00000043789 | Vwce | -2,19 | 0,044429755 | 8,433878 | 0,966288 | 3,460042 | 20,81699 | 10,14697 | 26,69248 |
| ENSMUSG00000001930 | Vwf | -3,08 | 0,015251764 | 2016,751 | 1737,385 | 1555,289 | 4284,336 | 4293,437 | 36330,25 |
| ENSMUSG00000051721 | Wdcp | 1,23 | 0,000686713 | 746,3982 | 619,3905 | 583,8821 | 363,8018 | 291,7254 | 177,0601 |
| ENSMUSG00000028391 | Wdr31 | -1,39 | 0,031274398 | 10,54235 | 19,32576 | 25,95032 | 48,57299 | 31,70928 | 65,84145 |
| ENSMUSG00000066643 | Wdr35 | -1,23 | 7,19296E-05 | 74,85067 | 79,2356 | 111,5864 | 217,0915 | 177,572 | 230,4451 |
| ENSMUSG00000042050 | Wdr60 | -1,55 | 4,27983E-10 | 102,2608 | 91,79734 | 106,3963 | 244,8475 | 312,0193 | 323,8688 |
| ENSMUSG00000055235 | Wdr86 | -1,65 | 0,03257542 | 33,73551 | 51,21325 | 21,62526 | 113,9978 | 36,78277 | 182,3986 |
| ENSMUSG00000069792 | Wfdc17 | 2,85 | 2,83218E-09 | 7700,131 | 11926,89 | 9776,35 | 787,0807 | 924,6427 | 2353,387 |
| ENSMUSG00000000983 | Wfdc18 | 2,53 | 2,11105E-07 | 111,7489 | 90,83105 | 61,41575 | 11,89543 | 12,68371 | 20,46424 |
| ENSMUSG00000051748 | Wfdc21 | -1,81 | 6,53879E-18 | 4445,708 | 5540,694 | 5212,554 | 17302,89 | 20797,48 | 15257,42 |
| ENSMUSG00000028173 | Wls | 1,11 | 2,43583E-10 | 6165,165 | 5604,469 | 6525,64 | 2790,469 | 3141,756 | 2564,258 |
| ENSMUSG00000035112 | Wnk4 | -2,81 | 8,42603E-06 | 50,60327 | 59,90984 | 42,38552 | 180,414 | 188,9873 | 699,343 |
| ENSMUSG00000022996 | Wnt10b | -1,44 | 0,042737347 | 23,19316 | 5,797727 | 12,97516 | 40,6427 | 34,24603 | 38,25922 |
| ENSMUSG00000033227 | Wnt6 | 1,59 | 0,032891026 | 22,13893 | 27,05606 | 16,4352 | 4,956427 | 8,878599 | 8,007744 |
| ENSMUSG00000017677 | Wsb1 | 1,13 | 0,000143106 | 8998,948 | 12941,49 | 9293,674 | 5986,373 | 4321,341 | 3931,802 |
| ENSMUSG00000036459 | Wtip | -1,32 | 0,012283933 | 18,97623 | 17,39318 | 21,62526 | 45,59913 | 34,24603 | 64,06195 |
| ENSMUSG00000027803 | Wwtr1 | -1,66 | 0,007456514 | 51,6575 | 26,08977 | 54,49567 | 61,4597 | 171,2301 | 186,8474 |
| ENSMUSG00000015342 | Xk | -1,36 | 1,19617E-07 | 1142,79 | 807,8166 | 864,1456 | 2446,493 | 2767,586 | 1991,259 |
| ENSMUSG00000031258 | Xkrx | -1,46 | 0,006394048 | 91,71842 | 158,4712 | 124,5615 | 362,8105 | 163,6199 | 503,5981 |
| ENSMUSG00000022100 | Xpo7 | -2,15 | 1,91678E-10 | 10629,85 | 6098,242 | 6935,655 | 43027,74 | 37036,44 | 25252,87 |
| ENSMUSG00000026187 | Xrcc5 | -2,11 | 8,9042E-13 | 166,5691 | 114,9882 | 187,7073 | 624,5098 | 582,1824 | 813,2309 |
| ENSMUSG00000053110 | Yap1 | -1,89 | 0,000522565 | 40,06092 | 32,85379 | 37,19546 | 66,41613 | 130,6422 | 209,9809 |
| ENSMUSG00000014932 | Yes1 | -1,15 | 0,012155803 | 39,00669 | 28,98863 | 35,46543 | 51,54684 | 91,32274 | 86,30569 |
| ENSMUSG00000034059 | Ypel4 | -3,30 | 3,85824E-12 | 1011,011 | 355,5939 | 384,9297 | 7370,207 | 5313,208 | 4594,666 |
| ENSMUSG00000079173 | Zan | -2,11 | 0,007669603 | 9,488113 | 6,764015 | 4,325053 | 20,81699 | 20,29394 | 46,26697 |
| ENSMUSG00000027514 | Zbp1 | 1,95 | 1,78676E-07 | 3223,85 | 2768,415 | 2099,381 | 542,2331 | 527,6425 | 1028,55 |
| ENSMUSG00000063659 | Zbtb18 | 1,01 | 0,003783856 | 6852,526 | 7749,628 | 6559,375 | 4800,796 | 3326,938 | 2361,395 |
| ENSMUSG00000027583 | Zbtb46 | -2,48 | 2,06176E-06 | 207,6842 | 60,87613 | 72,66089 | 630,4576 | 684,9205 | 581,0063 |
| ENSMUSG00000043542 | Zc2hc1a | 1,14 | 4,11335E-05 | 253,0163 | 314,0435 | 241,338 | 104,085 | 119,2269 | 143,2496 |
| ENSMUSG00000034265 | Zdhhc14 | -2,27 | 1,46905E-15 | 274,101 | 149,7746 | 205,8725 | 1015,076 | 1074,311 | 939,5753 |
| ENSMUSG00000033906 | Zdhhc15 | -1,54 | 2,58665E-07 | 140,2132 | 126,5837 | 172,1371 | 319,1939 | 461,6872 | 500,9289 |
| ENSMUSG00000028403 | Zdhhc21 | 1,28 | 8,31305E-09 | 4866,348 | 3762,725 | 4914,125 | 1981,58 | 2015,442 | 1583,754 |
| ENSMUSG00000024238 | Zeb1 | -1,50 | 1,81401E-26 | 656,7882 | 660,9409 | 642,7029 | 1941,928 | 1867,043 | 1746,578 |
| ENSMUSG00000026872 | Zeb2 | 1,25 | 5,09612E-05 | 12068,88 | 14061,42 | 12528,81 | 5321,22 | 3772,136 | 7128,672 |
| ENSMUSG00000052248 | Zeb2os | 1,56 | 2,23085E-06 | 208,7385 | 267,6617 | 270,7483 | 71,37255 | 68,49205 | 112,1084 |
| ENSMUSG00000057895 | Zfp105 | 2,30 | 2,94903E-10 | 200,3046 | 186,4935 | 178,1922 | 34,69499 | 55,80834 | 25,80273 |
| ENSMUSG00000030486 | Zfp108 | 1,06 | 0,023371838 | 61,14562 | 57,97727 | 63,14577 | 22,79957 | 24,09906 | 40,03872 |
| ENSMUSG00000087598 | Zfp111 | 1,12 | 5,4367E-07 | 1038,421 | 895,7488 | 881,4458 | 355,8715 | 451,5402 | 485,8032 |
| ENSMUSG00000068134 | Zfp120 | 1,14 | 8,64351E-14 | 2356,215 | 2155,788 | 2346,774 | 1036,885 | 963,9622 | 1106,848 |
| ENSMUSG00000049321 | Zfp2 | -1,86 | 3,57912E-05 | 22,13893 | 18,35947 | 16,4352 | 52,53813 | 65,95531 | 86,30569 |
| ENSMUSG00000005267 | Zfp287 | 1,23 | 0,001938593 | 281,4807 | 225,1451 | 206,7375 | 80,29412 | 77,37065 | 145,0291 |
| ENSMUSG00000037855 | Zfp365 | 2,11 | 0,016025224 | 35,84398 | 51,21325 | 44,98055 | 5,947713 | 2,536743 | 21,35398 |
| ENSMUSG00000021127 | Zfp36l1 | -1,61 | 2,09506E-09 | 445,9413 | 299,5492 | 372,8196 | 1157,821 | 929,7162 | 1333,734 |
| ENSMUSG00000068130 | Zfp442 | 1,39 | 0,004974117 | 67,47102 | 49,28068 | 67,47083 | 18,83442 | 17,7572 | 32,92073 |
| ENSMUSG00000060206 | Zfp462 | 1,40 | 0,035680226 | 73,79643 | 117,8871 | 122,8315 | 16,85185 | 35,5144 | 66,7312 |
| ENSMUSG00000068551 | Zfp467 | 1,94 | 1,55189E-11 | 1148,062 | 1102,534 | 1026,768 | 312,2549 | 191,5241 | 347,0023 |
| ENSMUSG00000039081 | Zfp503 | 1,01 | 0,008964902 | 67,47102 | 94,6962 | 107,2613 | 45,59913 | 45,66137 | 42,70797 |
| ENSMUSG00000024420 | Zfp521 | -1,21 | 0,013036968 | 89,60995 | 73,43787 | 63,14577 | 123,9107 | 129,3739 | 267,8146 |
| ENSMUSG00000062794 | Zfp599 | -1,35 | 0,010335209 | 63,25408 | 41,55038 | 50,17061 | 86,24184 | 101,4697 | 205,5321 |
| ENSMUSG00000048897 | Zfp710 | 1,09 | 0,000159905 | 9067,473 | 10771,21 | 10265,08 | 6221,308 | 3696,034 | 4269,017 |
| ENSMUSG00000071064 | Zfp827 | -1,78 | 1,45175E-08 | 48,4948 | 42,51666 | 41,52051 | 183,3878 | 126,8371 | 144,1394 |
| ENSMUSG00000050600 | Zfp831 | -1,22 | 0,00861283 | 531,3343 | 784,6257 | 351,1943 | 1823,965 | 1037,528 | 1014,314 |
| ENSMUSG00000095325 | Zfp870 | 1,01 | 0,003186761 | 363,711 | 422,2678 | 456,7256 | 138,78 | 254,9426 | 224,2168 |
| ENSMUSG00000031907 | Zfp90 | 1,50 | 3,4299E-06 | 132,8336 | 172,9655 | 157,4319 | 50,55556 | 44,393 | 67,62095 |
| ENSMUSG00000030424 | Zfp939 | 1,12 | 0,002117306 | 114,9116 | 164,2689 | 115,9114 | 48,57299 | 71,02879 | 63,17221 |
| ENSMUSG00000063383 | Zfp947 | 2,09 | 3,38185E-17 | 218,2266 | 205,8193 | 238,7429 | 52,53813 | 49,46648 | 53,38496 |
| ENSMUSG00000074519 | Zfp971 | 1,60 | 7,31463E-10 | 880,286 | 1237,815 | 861,5505 | 309,2811 | 296,7989 | 378,1435 |
| ENSMUSG00000078546 | Zfp995 | 1,23 | 1,14237E-05 | 171,8403 | 178,7632 | 206,7375 | 93,18083 | 67,22368 | 77,40819 |
| ENSMUSG00000049577 | Zfpm1 | -2,85 | 1,93561E-11 | 2000,938 | 680,2666 | 1004,277 | 8782,789 | 8321,784 | 9458,036 |
| ENSMUSG00000021286 | Zfyve21 | 1,38 | 2,84079E-12 | 1039,475 | 1329,612 | 1257,725 | 501,5904 | 428,7095 | 460,0004 |
| ENSMUSG00000034557 | Zfyve9 | 2,08 | 6,87337E-07 | 594,5884 | 1445,567 | 1274,161 | 311,2636 | 201,671 | 272,2633 |
| ENSMUSG00000049350 | Zg16 | -3,34 | 2,34581E-06 | 6,325408 | 1,932576 | 2,595032 | 37,66885 | 34,24603 | 35,58997 |
| ENSMUSG00000071757 | Zhx2 | -1,31 | 0,004389817 | 455,4294 | 472,5147 | 387,5247 | 1033,911 | 1642,541 | 579,2268 |
| ENSMUSG00000054931 | Zkscan4 | 2,79 | 4,16184E-14 | 147,5929 | 212,5833 | 188,5723 | 22,79957 | 20,29394 | 35,58997 |
| ENSMUSG00000023902 | Zscan10 | -1,95 | 0,033882998 | 2,108469 | 4,831439 | 5,190064 | 10,90414 | 19,02557 | 17,79499 |
| ENSMUSG00000022228 | Zscan26 | 1,16 | 2,816E-08 | 2185,429 | 2470,798 | 1982,604 | 1079,51 | 843,4669 | 1050,794 |
|  |  |  |  |  |  |  |  |  |  |
|  |  |  |  |  |  |  |  |  |  |
