# Supplementary material for: CDX2 expression in the hematopoietic lineage promotes leukemogenesis via TGFβ inhibition
Source: Mol Oncol. 2021 Jun 26;15(9):2318–29. doi: 10.1002/1878-0261.12982 (PMC8410536; doi:10.1002/1878-0261.12982)
Supplement: Supplementary file 5 — Table S4. Differentially‐expressed genes for DNA binding/Transcription Regulatory Factors. [file MOL2-15-2318-s005.docx]

**Table S4**

Differentially-expressed genes for DNA binding / Transcription Regulatory Factors. Transcription factors genes involved in hematopoiesis and leukemogenesis are underlined.

AFF3

AHR

ALX4

ARHGAP22

ARRB1

ATF3

ATF7

ATOH8

BATF2

BCL11A

BEND5

BEND6

BHLHE40

BTG2

CARHSP1

CBFA2T3

CBX7

CCND1

CDX2

CEBPA

CHD5

CITED4

CITED4

COPRS

CR2

CREB3L2

CREB3L3

CSRP3

CTNND2

CUX2

CXXC5

DMRTB1

DPF3

DR1

E2F4

EAF2

EBF1

EBF3

EGR2

EHF

ELL2

ERBB2

ERN1

ESR1

ETS1

ETV5

EYA1

FHL2

FOSB

FOXC1

FOXO3

FOXQ1

FSTL3

GATA1

GATA2

GBX2

GFI1B

GLIS3

H2AFY2

HACE1

*HES1*

HES5

HES7

HESX1

HIF3A

HIP1

HIST1H1C

HIST1H1E

HIST1H2AC

HIST1H2BC

HIST1H2BE

HIST1H2BN

HIST1H2BP

HIST3H2BA

HIVEP2

HIVEP3

HLF

HMGA2

HMGB3

HNF4A

HOPX

HOXA10

HOXA7

HOXA9

HOXD3

HSF2

ID3

IFI204

IFI211

IKZF3

IRF5

IRF8

JUN

KCNIP3

KHDRBS3

KLF1

KLF15

KLF3

KLF4

KLF7

KLF8

L3MBTL1

LEF1

LHX1

LHX2

LIN28A

LMCD1

LPIN1

LPIN2

LPXN

MAPK11

MAPK12

MAPK13

MB21D1

MED12L

MEIS1

MGMT

MIER2

MITF

MSX1

MXD1

NCOR2

NDN

NEUROD4

NFIA

NKAPL

NKX2-1

NOSTRIN

NOTCH3

NPAS3

NPAS4

NR0B2

NR1H3

NR3C2

NUPR1

ORC1

OTUD7B

PADI4

PAWR

PAX5

PAX8

PAX9

PBX1

PCX

PHF11D

PHF19

POU2AF1

POU6F1

PPARGC1A

PRDM16

PRDM5

PRKAA2

PRKCB

PTPN14

PTPRN

RARG

RASL11A

RB1

RCOR2

RFX2

RNF10

RUNX1T1

SATB1

SATB2

SCML4

SEC14L2

SERTAD2

SETBP1

SMAD9

SNAI1

SOX12

SOX18

SOX6

SP7

SPHK1

SPIB

SPIC

ST18

STAT4

SYCP2

TAL1

TAL2

TBX6

TCF3

TCF7L2

TEAD1

TEAD2

TFDP2

TFEC

THRB

TRP53INP2

TRPS1

TSC22D1

TSHZ3

VOPP1

WTIP

WWTR1

XRCC5

YAP1

ZBP1

ZBTB18

ZBTB46

ZEB1

ZEB2

ZFP105

ZFP120

ZFP2

ZFP287

ZFP36L1

ZFP467

ZFP503

ZFP521

ZFP710

ZFP827

ZFP831

ZFP90

ZFPM1

ZHX2

ZKSCAN4

ZSCAN10

ZSCAN26
