# Supplementary material for: CDX2 expression in the hematopoietic lineage promotes leukemogenesis via TGFβ inhibition
Source: Mol Oncol. 2021 Jun 26;15(9):2318–29. doi: 10.1002/1878-0261.12982 (PMC8410536; doi:10.1002/1878-0261.12982)
Supplement: Supplementary file 6 — Table S5. Combined analysis of DNA‐binding sites in promoter of hematopoietic transcription factors and their respective deregulated expression. [file MOL2-15-2318-s006.docx]

**Table S5**

Combined analysis of DNA-binding sites in promoter of hematopoietic transcription factors and their respective deregulated expression.

| **⮫** | | **Promoters** | | | | | | | | | | | | | | | | | | | |
| --- | --- | --- | --- | --- | --- | --- | --- | --- | --- | --- | --- | --- | --- | --- | --- | --- | --- | --- | --- | --- | --- |
|  |  | **CDX2** | **Ahr** | **Cebp**  ***** | **Egr2**  ***** | **Irf8**  ***** | **Jun**  ***** | **Klf4**  ***** | **E2f4**  ***** | **Ebf1** | **Foxo3** | **Gata1**  ***** | **Gata2** | **Gfi1b**  ***** | **Klf1**  ***** | **Meis1** | **Pbx1** | **Sox6**  ***** | **Tal1**  ***** | **Zfpm1**  ***** |  |
| **Transcription Factors** | **CDX2** |  | **1** |  |  | **1** | **1** |  | **0** |  | **0** |  |  |  |  | **0** |  |  |  |  |  |
|  | **Ahr** |  |  | **1** |  | **1** |  | **1** | **0** | **0** | **0** | **0** | **0** |  |  | **0** |  |  |  |  |  |
|  | **Cebpa *** |  |  |  |  |  |  |  |  |  |  |  |  | **0** |  |  |  |  |  |  |  |
|  | **Egr2 *** |  |  |  |  |  |  |  |  |  | **0** |  |  |  |  |  |  |  |  | **0** |  |
|  | **Irf8 *** |  | **1** | **1** |  | **1** |  |  | **0** |  |  |  |  | **0** |  |  |  |  |  |  |  |
|  | **Jun *** | **1** | **1** | **1** | **1** | **1** | **1** | **1** | **0** | **0** | **0** | **0** | **0** | **0** | **0** | **0** | **0** | **0** | **0** | **0** |  |
|  | **Klf4 *** | **1** | **1** | **1** | **1** | **1** | **1** | **1** | **0** | **0** | **0** | **0** | **0** | **0** | **0** | **0** | **0** |  | **0** | **0** |  |
|  | **E2f4 *** |  |  |  | **0** |  |  |  | **1** |  |  | **1** | **1** | **1** |  | **1** |  |  |  |  |  |
|  | **Ebf1** | **0** |  | **0** |  |  |  |  |  |  |  | **1** | **1** |  |  |  |  | **1** |  |  |  |
|  | **Foxo3** | **0** |  | **0** | **0** |  |  | **0** |  |  | **1** |  |  |  |  | **1** | **1** | **1** |  |  |  |
|  | **Gata1 *** |  |  |  | **0** |  | **0** |  | **1** | **1** |  | **1** | **1** | **1** | **1** |  | **1** |  | **1** |  |  |
|  | **Gata2** |  |  |  | **0** |  | **0** |  | **1** | **1** |  | **1** | **1** | **1** | **1** |  | **1** |  | **1** |  |  |
|  | **Gfi1b *** | **0** |  | **0** |  | **0** |  |  |  |  |  |  |  |  | **1** | **1** | **1** |  |  |  |  |
|  | **Klf1 *** | **0** |  | **0** |  |  | **0** |  | **1** |  |  | **1** | **1** | **1** |  | **1** |  |  |  | **1** |  |
|  | **Meis1** | **0** |  | **0** | **0** | **0** |  |  | **1** | **1** | **1** | **1** | **1** | **1** | **1** | **1** |  | **1** |  | **1** |  |
|  | **Pbx1** |  |  |  |  |  |  |  |  |  |  |  |  |  |  |  | **1** |  |  | **1** |  |
|  | **Sox6 *** |  | **0** |  | **0** | **0** |  | **0** | **1** | **1** | **1** | **1** | **1** | **1** |  | **1** |  | **1** | **1** | **1** |  |
|  | **Tal1 *** |  |  |  |  |  |  |  |  |  |  |  |  | **1** |  |  |  |  |  |  |  |
|  | **Zfpm1 *** |  |  |  |  |  |  |  |  |  |  |  |  |  |  |  |  |  |  |  |  |

The lines correspond to transcription factors involved in hematopoiesis, and the columns correspond to their respective gene promoters (-2000bp to +50bp with respect to the transcription start site). Green and red colors indicate transcription factors / gene promoters whose mRNA levels are respectively upregulated and downregulated in the bone marrow by ectopic *CDX2* expression in *MxCDX2* mice. A value of 1 is attributed when a given transcription factor and its putative target promoter correspond to genes whose expression levels change in the same way (both are upregulated or both are downregulated) in *MxCDX2* mice; a value of 0 is given when they change in opposite way.

The Circos plot representation of a subgroup (*) of this Table is shown in Figure 4C.
