# Supplementary material for: CDX2 expression in the hematopoietic lineage promotes leukemogenesis via TGFβ inhibition
Source: Mol Oncol. 2021 Jun 26;15(9):2318–29. doi: 10.1002/1878-0261.12982 (PMC8410536; doi:10.1002/1878-0261.12982)
Supplement: Supplementary file 7 — Table S6. Recurrent nucleotide changes in the transcribed sequences of MxCDX2 mice compared to control littermates. [file MOL2-15-2318-s008.docx]

**Table S6**

Recurrent nucleotide changes in the transcribed sequences of *MxCDX2* mice compared to control littermates.

| **Chromosome** | **Chromosomal Coordinates** | **Reference:Altered** | **ensGeneID** | **Gene Symbol** |
| --- | --- | --- | --- | --- |
|  |  |  |  |  |
| chr1 | 138121809 | TG:T | ENSMUSG00000026395 | Ptprc |
| chr1 | 170288954 | A:G | ENSMUSG00000102418 | Sh2d1b1 |
| chr1 | 171701954 | A:G | ENSMUSG00000015355 | Cd48 |
| chr1 | 66708747 | C:T | ENSMUSG00000026005 | Rpe |
| chr1 | 67002256 | C:A | ENSMUSG00000026000 | Lancl1 |
| chr1 | 91291262 | A:C | ENSMUSG00000034343 | Ube2f |
| chr1 | 94038650 | A:AT | ENSMUSG00000026285 | Pdcd1 |
| chr1 | 99190350 | A:T | ENSMUSG00000087804 | Gm23434 |
| chr1 | 99790318 | T:C | ENSMUSG00000067028 | Cntnap5b |
| chr1 | 99790342 | G:A | ENSMUSG00000067028 | Cntnap5b |
| chr1 | 33583113 | A:T | ENSMUSG00000026134 | Prim2 |
| chr1 | 37443158 | T:C | ENSMUSG00000026110 | Mgat4a |
| chr1 | 43050804 | C:T | ENSMUSG00000070939 | Tgfbrap1 |
| chr2 | 110462591 | C:G | ENSMUSG00000083320 | Gm13935 |
| chr2 | 121543632 | A:G | ENSMUSG00000027242 | Wdr76 |
| chr2 | 155283614 | G:A | ENSMUSG00000038383 | Pigu |
| chr2 | 155291037 | A:C | ENSMUSG00000038383 | Pigu |
| chr2 | 155643238 | T:C | ENSMUSG00000038324 | Trpc4ap |
| chr2 | 156311032 | A:G | ENSMUSG00000046229 | Scand1 |
| chr2 | 156320378 | T:A | ENSMUSG00000038085 | Cnbd2 |
| chr2 | 156320426 | G:A | ENSMUSG00000038085 | Cnbd2 |
| chr2 | 156321430 | C:T | ENSMUSG00000038085 | Cnbd2 |
| chr2 | 156321713 | A:G | ENSMUSG00000038085 | Cnbd2 |
| chr2 | 156342480 | T:G | ENSMUSG00000038085 | Cnbd2 |
| chr2 | 156349504 | T:C | ENSMUSG00000038085 | Cnbd2 |
| chr2 | 156349758 | A:C | ENSMUSG00000038085 | Cnbd2 |
| chr2 | 156351013 | A:G | ENSMUSG00000038085 | Cnbd2 |
| chr2 | 156351018 | A:G | ENSMUSG00000038085 | Cnbd2 |
| chr2 | 156525188 | G:A | ENSMUSG00000027624 | Epb41l1 |
| chr2 | 157993884 | C:A | ENSMUSG00000027650 | Tti1 |
| chr2 | 158317769 | T:G | ENSMUSG00000016024 | Lbp |
| chr2 | 158317802 | A:G | ENSMUSG00000016024 | Lbp |
| chr2 | 158317960 | G:T | ENSMUSG00000016024 | Lbp |
| chr2 | 158318736 | T:G | ENSMUSG00000016024 | Lbp |
| chr2 | 158319100 | C:A | ENSMUSG00000016024 | Lbp |
| chr2 | 158327893 | A:G | ENSMUSG00000016024 | Lbp |
| chr2 | 158328097 | A:G | ENSMUSG00000016024 | Lbp |
| chr2 | 158328617 | G:T | ENSMUSG00000016024 | Lbp |
| chr2 | 158330319 | A:C | ENSMUSG00000016024 | Lbp |
| chr2 | 158637611 | G:T | ENSMUSG00000037761 | Actr5 |
| chr2 | 165784823 | G:C | ENSMUSG00000039671 | Zmynd8 |
| chr2 | 166075605 | T:G | ENSMUSG00000006800 | Sulf2 |
| chr2 | 166076786 | A:G | ENSMUSG00000006800 | Sulf2 |
| chr2 | 168634687 | T:A | ENSMUSG00000027546 | Atp9a |
| chr2 | 31615894 | A:G | ENSMUSG00000026843 | Fubp3 |
| chr2 | 3504036 | G:A | ENSMUSG00000109865 | Hspa14 |
| chr2 | 81510802 | A:AC | ENSMUSG00000088194 | Gm23900 |
| chr2 | 90739497 | A:G | ENSMUSG00000051329 | Nup160 |
| chr3 | 10322192 | T:A | ENSMUSG00000103392 | Gm38303 |
| chr3 | 116676877 | T:C | ENSMUSG00000027957 | Slc35a3 |
| chr3 | 116744163 | T:C | ENSMUSG00000033400 | Agl |
| chr3 | 153931979 | A:C | ENSMUSG00000062908 | Acadm |
| chr3 | 153932929 | G:C | ENSMUSG00000062908 | Acadm |
| chr3 | 32336903 | A:G | ENSMUSG00000027663 | Zmat3 |
| chr3 | 32339795 | G:A | ENSMUSG00000027663 | Zmat3 |
| chr3 | 32340860 | A:G | ENSMUSG00000027663 | Zmat3 |
| chr3 | 32340861 | T:C | ENSMUSG00000027663 | Zmat3 |
| chr3 | 33801010 | G:C | ENSMUSG00000027677 | Ttc14 |
| chr3 | 33804834 | G:A | ENSMUSG00000027677 | Ttc14 |
| chr3 | 65959007 | A:G | ENSMUSG00000103041 | Gm37305 |
| chr3 | 88411654 | A:AT | ENSMUSG00000050144 | Slc25a44 |
| chr3 | 96624315 | A:G | ENSMUSG00000049288 | Lix1l |
| chr4 | 107055235 | T:C | ENSMUSG00000028622 | Mrpl37 |
| chr4 | 140718569 | A:G | ENSMUSG00000040945 | Rcc2 |
| chr4 | 140918614 | T:C | ENSMUSG00000028927 | Padi2 |
| chr4 | 140918717 | A:G | ENSMUSG00000064443 | Gm26226 |
| chr4 | 140918940 | T:C | ENSMUSG00000028927 | Padi2 |
| chr4 | 140919154 | T:C | ENSMUSG00000028927 | Padi2 |
| chr4 | 140931899 | T:C | ENSMUSG00000028927 | Padi2 |
| chr4 | 140932914 | C:T | ENSMUSG00000028927 | Padi2 |
| chr4 | 140932955 | T:A | ENSMUSG00000028927 | Padi2 |
| chr4 | 140974101 | G:C | ENSMUSG00000009863 | Sdhb |
| chr4 | 141002272 | T:G | ENSMUSG00000036622 | Atp13a2 |
| chr4 | 141220516 | T:C | ENSMUSG00000073733 | Cplane2 |
| chr4 | 141458772 | A:C | ENSMUSG00000006215 | Zbtb17 |
| chr4 | 141691929 | C:T | ENSMUSG00000078515 | Ddi2 |
| chr4 | 141692756 | C:T | ENSMUSG00000078515 | Ddi2 |
| chr4 | 141693240 | A:C | ENSMUSG00000078515 | Ddi2 |
| chr4 | 141693282 | G:A | ENSMUSG00000078515 | Ddi2 |
| chr4 | 141695254 | G:A | ENSMUSG00000078515 | Ddi2 |
| chr4 | 141701984 | GT:G | ENSMUSG00000078515 | Ddi2 |
| chr4 | 141701989 | T:G | ENSMUSG00000078515 | Ddi2 |
| chr4 | 141705382 | C:T | ENSMUSG00000078515 | Ddi2 |
| chr4 | 144921113 | C:T | ENSMUSG00000066026 | Dhrs3 |
| chr4 | 145224184 | GCTTCCCC:G | ENSMUSG00000028599 | Tnfrsf1b |
| chr4 | 145224192 | AGT:A | ENSMUSG00000028599 | Tnfrsf1b |
| chr4 | 145292685 | A:C | ENSMUSG00000028602 | Tnfrsf8 |
| chr4 | 145292746 | T:C | ENSMUSG00000028602 | Tnfrsf8 |
| chr4 | 145294635 | T:C | ENSMUSG00000028602 | Tnfrsf8 |
| chr4 | 146270623 | C:T | ENSMUSG00000085525 | Gm13166 |
| chr4 | 146449070 | C:T | ENSMUSG00000070605 | Zfp992 |
| chr4 | 146454614 | T:C | ENSMUSG00000070605 | Zfp992 |
| chr4 | 146454621 | C:T | ENSMUSG00000070605 | Zfp992 |
| chr4 | 146456889 | GTC:G | ENSMUSG00000070605 | Zfp992 |
| chr4 | 146456894 | T:TTA | ENSMUSG00000070605 | Zfp992 |
| chr4 | 146910781 | G:A | ENSMUSG00000045699 | Gm21411 |
| chr4 | 146920723 | G:A | ENSMUSG00000045699 | Gm21411 |
| chr4 | 148016271 | GAA:G | ENSMUSG00000029016 | Clcn6 |
| chr4 | 148143664 | C:G | ENSMUSG00000029003 | Mad2l2 |
| chr4 | 148144812 | T:C | ENSMUSG00000029003 | Mad2l2 |
| chr4 | 148574509 | G:T | ENSMUSG00000017264 | Exosc10 |
| chr4 | 148595000 | A:G | ENSMUSG00000006442 | Srm |
| chr4 | 149653666 | A:G | ENSMUSG00000039936 | Pik3cd |
| chr4 | 149657626 | T:C | ENSMUSG00000039936 | Pik3cd |
| chr4 | 150241780 | C:T | ENSMUSG00000063524 | Eno1 |
| chr4 | 150902322 | A:G | ENSMUSG00000028964 | Park7 |
| chr4 | 151055494 | A:T | ENSMUSG00000014592 | Camta1 |
| chr4 | 151985433 | G:GT | ENSMUSG00000039759 | Thap3 |
| chr4 | 151987335 | A:G | ENSMUSG00000039759 | Thap3 |
| chr4 | 152023620 | C:T | ENSMUSG00000028952 | Zbtb48 |
| chr4 | 152032080 | T:G | ENSMUSG00000028950 | Tas1r1 |
| chr4 | 152112641 | A:G | ENSMUSG00000039713 | Plekhg5 |
| chr4 | 152270058 | AG:A | ENSMUSG00000028937 | Acot7 |
| chr4 | 152270095 | C:T | ENSMUSG00000028937 | Acot7 |
| chr4 | 152270109 | T:C | ENSMUSG00000028937 | Acot7 |
| chr4 | 152326124 | G:A | ENSMUSG00000028936 | Rpl22 |
| chr4 | 152398700 | C:T | ENSMUSG00000028931 | Kcnab2 |
| chr4 | 152399928 | T:C | ENSMUSG00000028931 | Kcnab2 |
| chr4 | 152399958 | G:A | ENSMUSG00000028931 | Kcnab2 |
| chr4 | 152406228 | C:T | ENSMUSG00000028931 | Kcnab2 |
| chr4 | 154320539 | T:C | ENSMUSG00000039410 | Prdm16 |
| chr4 | 154921951 | T:C | ENSMUSG00000042333 | Tnfrsf14 |
| chr4 | 154923854 | G:GC | ENSMUSG00000042333 | Tnfrsf14 |
| chr4 | 154932691 | C:T | ENSMUSG00000042333 | Tnfrsf14 |
| chr4 | 155252456 | A:G | ENSMUSG00000073684 | Faap20 |
| chr4 | 155805545 | T:C | ENSMUSG00000029066 | Mrpl20 |
| chr4 | 155809006 | T:C | ENSMUSG00000029066 | Mrpl20 |
| chr4 | 155809009 | A:C | ENSMUSG00000029066 | Mrpl20 |
| chr4 | 155812038 | T:A | ENSMUSG00000029068 | Ccnl2 |
| chr4 | 155889427 | C:T | ENSMUSG00000051557 | Pusl1 |
| chr4 | 155895172 | C:G | ENSMUSG00000029033 | Acap3 |
| chr4 | 155895728 | AC:A | ENSMUSG00000029033 | Acap3 |
| chr4 | 155908341 | C:T | ENSMUSG00000029033 | Acap3 |
| chr4 | 155908562 | A:G | ENSMUSG00000029033 | Acap3 |
| chr4 | 155909517 | G:A | ENSMUSG00000096174 | Gm22991 |
| chr4 | 155910747 | G:T | ENSMUSG00000096174 | Gm22991 |
| chr4 | 155928071 | G:T | ENSMUSG00000023286 | Ube2j2 |
| chr4 | 155928227 | A:G | ENSMUSG00000023286 | Ube2j2 |
| chr4 | 155928266 | C:T | ENSMUSG00000023286 | Ube2j2 |
| chr4 | 32022582 | A:G | ENSMUSG00000028284 | Map3k7 |
| chr4 | 3554421 | A:G | ENSMUSG00000028232 | Tmem68 |
| chr4 | 3677607 | T:C | ENSMUSG00000042228 | Lyn |
| chr4 | 3684160 | A:G | ENSMUSG00000042228 | Lyn |
| chr4 | 3798780 | G:T | ENSMUSG00000042228 | Lyn |
| chr4 | 3831839 | CAA:C | ENSMUSG00000028234 | Rps20 |
| chr4 | 46582584 | T:C | ENSMUSG00000028337 | Coro2a |
| chr4 | 55294764 | C:T | ENSMUSG00000077403 | Gm25419 |
| chr4 | 55392019 | A:G | ENSMUSG00000028426 | Rad23b |
| chr4 | 55531024 | C:T | ENSMUSG00000003032 | Klf4 |
| chr4 | 55531025 | A:C | ENSMUSG00000003032 | Klf4 |
| chr4 | 59025645 | C:T | ENSMUSG00000092345 | Gm20503 |
| chr4 | 59025646 | A:G | ENSMUSG00000092345 | Gm20503 |
| chr4 | 63356589 | C:T | ENSMUSG00000028359 | Orm3 |
| chr4 | 63356599 | T:C | ENSMUSG00000028359 | Orm3 |
| chr4 | 63356708 | C:T | ENSMUSG00000028359 | Orm3 |
| chr4 | 63356730 | G:T | ENSMUSG00000028359 | Orm3 |
| chr4 | 63357242 | A:C | ENSMUSG00000028359 | Orm3 |
| chr4 | 63359429 | C:T | ENSMUSG00000028359 | Orm3 |
| chr4 | 63359454 | C:T | ENSMUSG00000028359 | Orm3 |
| chr4 | 63551277 | C:T | ENSMUSG00000039105 | Atp6v1g1 |
| chr4 | 6741550 | G:A | ENSMUSG00000041272 | Tox |
| chr4 | 6741559 | C:A | ENSMUSG00000041272 | Tox |
| chr4 | 8145899 | T:C | ENSMUSG00000041261 | Car8 |
| chr4 | 8146020 | GCA:G | ENSMUSG00000041261 | Car8 |
| chr4 | 8146044 | A:C | ENSMUSG00000041261 | Car8 |
| chr4 | 8146190 | G:A | ENSMUSG00000041261 | Car8 |
| chr4 | 8146191 | G:A | ENSMUSG00000041261 | Car8 |
| chr4 | 8592223 | C:T | ENSMUSG00000047187 | Rab2a |
| chr4 | 8595089 | A:G | ENSMUSG00000047187 | Rab2a |
| chr4 | 8599006 | A:T | ENSMUSG00000047187 | Rab2a |
| chr4 | 8602367 | G:A | ENSMUSG00000047187 | Rab2a |
| chr4 | 94088821 | T:A | ENSMUSG00000085931 | Gm12648 |
| chr5 | 100566414 | G:A | ENSMUSG00000029322 | Plac8 |
| chr5 | 107902834 | A:G | ENSMUSG00000058558 | Rpl5 |
| chr5 | 108097005 | C:T | ENSMUSG00000029267 | Mtf2 |
| chr5 | 108128001 | T:C | ENSMUSG00000063406 | Tmed5 |
| chr5 | 108129672 | A:G | ENSMUSG00000063406 | Tmed5 |
| chr5 | 108571611 | C:T | ENSMUSG00000062234 | Gak |
| chr5 | 108631957 | A:T | ENSMUSG00000013495 | Tmem175 |
| chr5 | 108635349 | C:T | ENSMUSG00000013495 | Tmem175 |
| chr5 | 108653568 | C:G | ENSMUSG00000004815 | Dgkq |
| chr5 | 108653865 | A:G | ENSMUSG00000004815 | Dgkq |
| chr5 | 108681188 | T:C | ENSMUSG00000033540 | Idua |
| chr5 | 109549976 | T:G | ENSMUSG00000106028 | Gm8493 |
| chr5 | 109550413 | A:G | ENSMUSG00000106028 | Gm8493 |
| chr5 | 109550430 | T:C | ENSMUSG00000106028 | Gm8493 |
| chr5 | 109553083 | C:A | ENSMUSG00000106028 | Gm8493 |
| chr5 | 109558102 | A:G | ENSMUSG00000033467 | Crlf2 |
| chr5 | 109558144 | T:C | ENSMUSG00000033467 | Crlf2 |
| chr5 | 109736203 | G:C | ENSMUSG00000072762 | 4930522L14Rik |
| chr5 | 109888137 | T:C | ENSMUSG00000097140 | Gm26779 |
| chr5 | 109888141 | C:G | ENSMUSG00000097140 | Gm26779 |
| chr5 | 109938534 | G:T | ENSMUSG00000090015 | Gm15446 |
| chr5 | 110206505 | G:C | ENSMUSG00000029502 | Golga3 |
| chr5 | 110223382 | C:CATAA | ENSMUSG00000029502 | Golga3 |
| chr5 | 110252610 | T:C | ENSMUSG00000029501 | Ankle2 |
| chr5 | 110256681 | C:T | ENSMUSG00000029501 | Ankle2 |
| chr5 | 110256682 | C:G | ENSMUSG00000029501 | Ankle2 |
| chr5 | 110256956 | G:C | ENSMUSG00000029501 | Ankle2 |
| chr5 | 110259129 | C:CT | ENSMUSG00000029500 | Pgam5 |
| chr5 | 110371988 | A:G | ENSMUSG00000043323 | Fbrsl1 |
| chr5 | 110387039 | C:A | ENSMUSG00000043323 | Fbrsl1 |
| chr5 | 112304966 | G:GC | ENSMUSG00000029344 | Tpst2 |
| chr5 | 112689017 | GTC:G | ENSMUSG00000072720 | Myo18b |
| chr5 | 112770067 | T:C | ENSMUSG00000072720 | Myo18b |
| chr5 | 112809682 | T:C | ENSMUSG00000072720 | Myo18b |
| chr5 | 112817677 | T:G | ENSMUSG00000072720 | Myo18b |
| chr5 | 113847912 | C:T | ENSMUSG00000004530 | Coro1c |
| chr5 | 114372651 | C:T | ENSMUSG00000001098 | Kctd10 |
| chr5 | 125020237 | G:A | ENSMUSG00000029478 | Ncor2 |
| chr5 | 125286080 | T:G | ENSMUSG00000037936 | Scarb1 |
| chr5 | 125456906 | A:G | ENSMUSG00000037905 | Bri3bp |
| chr5 | 125456927 | A:G | ENSMUSG00000037905 | Bri3bp |
| chr5 | 127579029 | T:A | ENSMUSG00000106015 | Gm42980 |
| chr5 | 127590131 | A:C | ENSMUSG00000029416 | Slc15a4 |
| chr5 | 127601606 | C:G | ENSMUSG00000029416 | Slc15a4 |
| chr5 | 127607631 | T:C | ENSMUSG00000029416 | Slc15a4 |
| chr5 | 127610580 | C:T | ENSMUSG00000029416 | Slc15a4 |
| chr5 | 147306965 | A:G | ENSMUSG00000029646 | Cdx2 |
| chr5 | 38122706 | A:G | ENSMUSG00000029125 | Stx18 |
| chr5 | 38125356 | C:T | ENSMUSG00000029125 | Stx18 |
| chr5 | 38211303 | T:G | ENSMUSG00000029127 | Zbtb49 |
| chr5 | 38268113 | G:A | ENSMUSG00000067365 | Tmem128 |
| chr5 | 38268141 | G:A | ENSMUSG00000067365 | Tmem128 |
| chr5 | 38351828 | C:T | ENSMUSG00000005107 | Slc2a9 |
| chr5 | 38531909 | G:A | ENSMUSG00000005103 | Wdr1 |
| chr5 | 38531979 | C:CA | ENSMUSG00000005103 | Wdr1 |
| chr5 | 49960118 | G:A | ENSMUSG00000029090 | Adgra3 |
| chr5 | 52666677 | C:T | ENSMUSG00000029173 | Sepsecs |
| chr5 | 52757098 | G:A | ENSMUSG00000029186 | Pi4k2b |
| chr5 | 52758728 | T:G | ENSMUSG00000029186 | Pi4k2b |
| chr5 | 52759544 | T:A | ENSMUSG00000029186 | Pi4k2b |
| chr5 | 53662296 | G:A | ENSMUSG00000039191 | Rbpj |
| chr5 | 53662329 | A:T | ENSMUSG00000039191 | Rbpj |
| chr5 | 57729016 | A:G | ENSMUSG00000029108 | Pcdh7 |
| chr5 | 58129507 | C:T | ENSMUSG00000029108 | Pcdh7 |
| chr5 | 58130500 | T:G | ENSMUSG00000029108 | Pcdh7 |
| chr5 | 58132252 | C:CAG | ENSMUSG00000029108 | Pcdh7 |
| chr5 | 58132655 | A:G | ENSMUSG00000029108 | Pcdh7 |
| chr5 | 64338294 | A:C | ENSMUSG00000029174 | Tbc1d1 |
| chr5 | 64338295 | A:C | ENSMUSG00000029174 | Tbc1d1 |
| chr5 | 64339486 | G:T | ENSMUSG00000029174 | Tbc1d1 |
| chr5 | 64349714 | C:T | ENSMUSG00000029174 | Tbc1d1 |
| chr5 | 64929067 | G:A | ENSMUSG00000044827 | Tlr1 |
| chr5 | 65019083 | A:T | ENSMUSG00000029185 | Fam114a1 |
| chr5 | 65039522 | C:T | ENSMUSG00000029185 | Fam114a1 |
| chr5 | 65039757 | C:A | ENSMUSG00000029185 | Fam114a1 |
| chr5 | 65040336 | C:T | ENSMUSG00000029185 | Fam114a1 |
| chr5 | 65041850 | G:T | ENSMUSG00000029185 | Fam114a1 |
| chr5 | 65041865 | A:G | ENSMUSG00000029185 | Fam114a1 |
| chr5 | 65156051 | C:T | ENSMUSG00000054920 | Klhl5 |
| chr5 | 65156491 | C:T | ENSMUSG00000054920 | Klhl5 |
| chr5 | 65157240 | CAAA:C | ENSMUSG00000054920 | Klhl5 |
| chr5 | 65279005 | A:G | ENSMUSG00000029191 | Rfc1 |
| chr5 | 65422523 | TCAAA:T | ENSMUSG00000029201 | Ugdh |
| chr5 | 65461285 | G:T | ENSMUSG00000037822 | Smim14 |
| chr5 | 65490511 | T:G | ENSMUSG00000037822 | Smim14 |
| chr5 | 65490512 | T:A | ENSMUSG00000037822 | Smim14 |
| chr5 | 65606089 | G:A | ENSMUSG00000029202 | Pds5a |
| chr5 | 65779987 | G:A | ENSMUSG00000106682 | Gm42648 |
| chr5 | 66687170 | C:T | ENSMUSG00000029223 | Uchl1 |
| chr5 | 67260253 | A:G | ENSMUSG00000107319 | Gm42670 |
| chr5 | 67666732 | A:G | ENSMUSG00000037685 | Atp8a1 |
| chr5 | 73310750 | GT:G | ENSMUSG00000029152 | Ociad1 |
| chr5 | 74204987 | A:G | ENSMUSG00000062110 | Scfd2 |
| chr5 | 74204989 | G:C | ENSMUSG00000062110 | Scfd2 |
| chr5 | 74205098 | T:C | ENSMUSG00000062110 | Scfd2 |
| chr5 | 74205903 | T:TG | ENSMUSG00000062110 | Scfd2 |
| chr5 | 74441623 | A:G | ENSMUSG00000062110 | Scfd2 |
| chr5 | 74451818 | C:T | ENSMUSG00000062110 | Scfd2 |
| chr5 | 76150708 | A:T | ENSMUSG00000029233 | Srd5a3 |
| chr5 | 76151525 | G:GA | ENSMUSG00000029233 | Srd5a3 |
| chr5 | 76156115 | T:C | ENSMUSG00000029233 | Srd5a3 |
| chr5 | 76185655 | A:C | ENSMUSG00000029234 | Tmem165 |
| chr5 | 76202347 | T:C | ENSMUSG00000029234 | Tmem165 |
| chr5 | 76210856 | T:C | ENSMUSG00000029238 | Clock |
| chr5 | 86093305 | A:C | ENSMUSG00000029254 | Stap1 |
| chr5 | 86109123 | A:G | ENSMUSG00000035898 | Uba6 |
| chr5 | 89676466 | GCACAAA:G | ENSMUSG00000043635 | Adamts3 |
| chr5 | 89697618 | T:G | ENSMUSG00000043635 | Adamts3 |
| chr5 | 89708675 | G:A | ENSMUSG00000043635 | Adamts3 |
| chr5 | 90216217 | C:A | ENSMUSG00000035505 | Cox18 |
| chr5 | 90217273 | G:A | ENSMUSG00000035505 | Cox18 |
| chr5 | 90229878 | T:G | ENSMUSG00000055204 | Ankrd17 |
| chr5 | 90239675 | G:A | ENSMUSG00000055204 | Ankrd17 |
| chr5 | 90608943 | C:T | ENSMUSG00000029370 | Rassf6 |
| chr5 | 90608945 | G:C | ENSMUSG00000029370 | Rassf6 |
| chr5 | 90640640 | T:A | ENSMUSG00000029370 | Rassf6 |
| chr5 | 91947161 | A:AT | ENSMUSG00000029397 | Rchy1 |
| chr5 | 91952694 | A:G | ENSMUSG00000029397 | Rchy1 |
| chr5 | 91972566 | G:T | ENSMUSG00000102644 | Thap6 |
| chr5 | 92006271 | C:T | ENSMUSG00000029403 | Cdkl2 |
| chr5 | 92006881 | T:C | ENSMUSG00000029403 | Cdkl2 |
| chr5 | 92245584 | C:G | ENSMUSG00000029410 | Ppef2 |
| chr5 | 92258968 | C:T | ENSMUSG00000029413 | Naaa |
| chr5 | 92264236 | A:G | ENSMUSG00000029413 | Naaa |
| chr5 | 92291342 | T:A | ENSMUSG00000106698 | Gm43599 |
| chr5 | 92295842 | T:G | ENSMUSG00000106698 | Gm43599 |
| chr5 | 92300340 | G:T | ENSMUSG00000106698 | Gm43599 |
| chr5 | 92605942 | T:A | ENSMUSG00000047963 | Stbd1 |
| chr5 | 93454688 | G:A | ENSMUSG00000096044 | Gm16427 |
| chr5 | 93454694 | A:G | ENSMUSG00000096044 | Gm16427 |
| chr5 | 93455633 | A:T | ENSMUSG00000096044 | Gm16427 |
| chr5 | 95512859 | G:A | ENSMUSG00000079423 | Gm3286 |
| chr5 | 95512860 | T:C | ENSMUSG00000079423 | Gm3286 |
| chr5 | 96936580 | C:T | ENSMUSG00000105377 | Gm43148 |
| chr5 | 97083040 | C:T | ENSMUSG00000055725 | Paqr3 |
| chr6 | 120905615 | G:C | ENSMUSG00000004446 | Bid |
| chr6 | 128406115 | T:G | ENSMUSG00000001520 | Nrip2 |
| chr6 | 128407798 | T:C | ENSMUSG00000001520 | Nrip2 |
| chr6 | 129007865 | C:T | ENSMUSG00000087150 | BC064078 |
| chr6 | 129056293 | A:G | ENSMUSG00000030154 | Klrb1f |
| chr6 | 129057550 | T:C | ENSMUSG00000030154 | Klrb1f |
| chr6 | 129063538 | C:T | ENSMUSG00000030154 | Klrb1f |
| chr6 | 129649575 | T:C | ENSMUSG00000052736 | Klrc2 |
| chr6 | 129874907 | T:C | ENSMUSG00000014543 | Klra17 |
| chr6 | 129874909 | A:G | ENSMUSG00000014543 | Klra17 |
| chr6 | 129972403 | G:T | ENSMUSG00000083120 | Gm15854 |
| chr6 | 130377839 | T:C | ENSMUSG00000079853 | Klra1 |
| chr6 | 134933456 | T:C | ENSMUSG00000098318 | Lockd |
| chr6 | 134984732 | T:C | ENSMUSG00000090698 | Apold1 |
| chr6 | 135021780 | T:G | ENSMUSG00000030204 | Ddx47 |
| chr6 | 136815967 | T:C | ENSMUSG00000030216 | Wbp11 |
| chr6 | 136820052 | A:T | ENSMUSG00000030216 | Wbp11 |
| chr6 | 137419769 | A:C | ENSMUSG00000030223 | Ptpro |
| chr6 | 137419770 | G:GC | ENSMUSG00000030223 | Ptpro |
| chr6 | 137420641 | A:C | ENSMUSG00000030223 | Ptpro |
| chr6 | 137420642 | A:C | ENSMUSG00000030223 | Ptpro |
| chr6 | 137422397 | T:C | ENSMUSG00000030223 | Ptpro |
| chr6 | 137422713 | G:A | ENSMUSG00000030223 | Ptpro |
| chr6 | 137423088 | G:C | ENSMUSG00000030223 | Ptpro |
| chr6 | 137423107 | T:C | ENSMUSG00000030223 | Ptpro |
| chr6 | 137423131 | G:T | ENSMUSG00000030223 | Ptpro |
| chr6 | 137423173 | C:T | ENSMUSG00000030223 | Ptpro |
| chr6 | 137423198 | G:A | ENSMUSG00000030223 | Ptpro |
| chr6 | 137424005 | A:C | ENSMUSG00000030223 | Ptpro |
| chr6 | 137424288 | A:G | ENSMUSG00000030223 | Ptpro |
| chr6 | 137425748 | A:G | ENSMUSG00000030223 | Ptpro |
| chr6 | 137425755 | G:T | ENSMUSG00000030223 | Ptpro |
| chr6 | 137425859 | T:C | ENSMUSG00000030223 | Ptpro |
| chr6 | 137425866 | A:C | ENSMUSG00000030223 | Ptpro |
| chr6 | 137426834 | C:G | ENSMUSG00000030223 | Ptpro |
| chr6 | 137456368 | G:C | ENSMUSG00000030223 | Ptpro |
| chr6 | 137591460 | A:G | ENSMUSG00000015766 | Eps8 |
| chr6 | 49060548 | A:G | ENSMUSG00000029816 | Gpnmb |
| chr6 | 49060650 | A:G | ENSMUSG00000029816 | Gpnmb |
| chr7 | 104422219 | GTT:G | ENSMUSG00000030921 | Trim30a |
| chr7 | 131560495 | C:T | ENSMUSG00000030849 | Fgfr2 |
| chr7 | 131563071 | C:G | ENSMUSG00000030849 | Fgfr2 |
| chr7 | 131563086 | C:T | ENSMUSG00000030849 | Fgfr2 |
| chr7 | 131573423 | G:A | ENSMUSG00000030849 | Fgfr2 |
| chr7 | 139426540 | C:T | ENSMUSG00000025477 | Inpp5a |
| chr7 | 142577095 | T:C | ENSMUSG00000076275 | Mir675 |
| chr7 | 144921119 | G:A | ENSMUSG00000031072 | LTO1 |
| chr7 | 144921596 | T:C | ENSMUSG00000031072 | LTO1 |
| chr7 | 145248398 | G:A | ENSMUSG00000048677 | Tpcn2 |
| chr7 | 145250874 | T:C | ENSMUSG00000048677 | Tpcn2 |
| chr7 | 145260582 | G:A | ENSMUSG00000048677 | Tpcn2 |
| chr7 | 45491353 | T:C | ENSMUSG00000023467 | Tulp2 |
| chr7 | 53887319 | A:G | ENSMUSG00000099176 | Mir6238 |
| chr7 | 66088512 | C:T | ENSMUSG00000075701 | Selenos |
| chr7 | 66261453 | C:T | ENSMUSG00000015133 | Lrrk1 |
| chr7 | 68739750 | G:C | ENSMUSG00000042659 | Arrdc4 |
| chr7 | 78792010 | CTG:C | ENSMUSG00000030611 | Mrps11 |
| chr7 | 80241632 | G:A | ENSMUSG00000050973 | Gdpgp1 |
| chr7 | 80288609 | T:A | ENSMUSG00000030534 | Vps33b |
| chr7 | 80289898 | G:A | ENSMUSG00000030534 | Vps33b |
| chr7 | 80301724 | T:G | ENSMUSG00000038943 | Prc1 |
| chr7 | 80327136 | A:G | ENSMUSG00000030533 | Unc45a |
| chr7 | 80360231 | A:G | ENSMUSG00000038886 | Man2a2 |
| chr7 | 80360723 | T:C | ENSMUSG00000038886 | Man2a2 |
| chr7 | 80401951 | T:C | ENSMUSG00000030530 | Furin |
| chr7 | 81038812 | T:G | ENSMUSG00000005621 | Zfp592 |
| chr7 | 81038900 | T:G | ENSMUSG00000005621 | Zfp592 |
| chr7 | 81524392 | A:G | ENSMUSG00000097277 | 2900076A07Rik |
| chr7 | 81524449 | C:T | ENSMUSG00000097277 | 2900076A07Rik |
| chr7 | 81525518 | A:G | ENSMUSG00000097277 | 2900076A07Rik |
| chr7 | 81527992 | G:T | ENSMUSG00000097277 | 2900076A07Rik |
| chr7 | 81776376 | A:C | ENSMUSG00000025102 | 3110040N11Rik |
| chr7 | 83892036 | G:C | ENSMUSG00000038503 | Mesd |
| chr7 | 88305590 | A:G | ENSMUSG00000030560 | Ctsc |
| chr7 | 88309033 | A:G | ENSMUSG00000030560 | Ctsc |
| chr7 | 88311773 | A:AG | ENSMUSG00000109244 | Gm44751 |
| chr7 | 88313804 | A:G | ENSMUSG00000109244 | Gm44751 |
| chr7 | 88313930 | C:CAG | ENSMUSG00000109244 | Gm44751 |
| chr7 | 88313936 | G:A | ENSMUSG00000109244 | Gm44751 |
| chr7 | 89940119 | G:A | ENSMUSG00000062797 | Hikeshi |
| chr7 | 89940121 | G:T | ENSMUSG00000062797 | Hikeshi |
| chr7 | 90040668 | C:A | ENSMUSG00000097585 | E230029C05Rik |
| chr7 | 90047503 | TG:T | ENSMUSG00000097585 | E230029C05Rik |
| chr7 | 90047505 | C:T | ENSMUSG00000097585 | E230029C05Rik |
| chr7 | 90131090 | A:T | ENSMUSG00000039361 | Picalm |
| chr7 | 90409592 | A:G | ENSMUSG00000030616 | Sytl2 |
| chr8 | 13043993 | G:GAAA | ENSMUSG00000031444 | F10 |
| chr8 | 19703115 | T:G | ENSMUSG00000087153 | Gm6483 |
| chr8 | 19703206 | A:C | ENSMUSG00000087153 | Gm6483 |
| chr8 | 19703254 | T:A | ENSMUSG00000087153 | Gm6483 |
| chr8 | 19703435 | C:T | ENSMUSG00000087153 | Gm6483 |
| chr8 | 19703470 | C:T | ENSMUSG00000087153 | Gm6483 |
| chr8 | 19706957 | T:C | ENSMUSG00000110531 | Gm7760 |
| chr8 | 25814414 | T:C | ENSMUSG00000031574 | Star |
| chr8 | 25814415 | T:C | ENSMUSG00000031574 | Star |
| chr8 | 31736728 | A:C | ENSMUSG00000093862 | Gm5117 |
| chr8 | 80993071 | G:C | ENSMUSG00000038250 | Usp38 |
| chr8 | 82765534 | G:C | ENSMUSG00000031711 | Zfp330 |
| chr8 | 82766065 | A:G | ENSMUSG00000031711 | Zfp330 |
| chr8 | 82771699 | G:T | ENSMUSG00000031711 | Zfp330 |
| chr8 | 83042682 | T:A | ENSMUSG00000047747 | Rnf150 |
| chr8 | 83083939 | T:C | ENSMUSG00000109876 | Gm45449 |
| chr8 | 83084158 | C:T | ENSMUSG00000109876 | Gm45449 |
| chr8 | 83437455 | T:C | ENSMUSG00000063253 | Scoc |
| chr8 | 83674087 | G:A | ENSMUSG00000057672 | Pkn1 |
| chr8 | 83690240 | T:C | ENSMUSG00000057672 | Pkn1 |
| chr8 | 84092367 | C:T | ENSMUSG00000031706 | Rfx1 |
| chr8 | 84092473 | T:C | ENSMUSG00000031706 | Rfx1 |
| chr8 | 84097026 | T:C | ENSMUSG00000031706 | Rfx1 |
| chr8 | 84130633 | T:C | ENSMUSG00000012889 | Podnl1 |
| chr8 | 84130645 | C:T | ENSMUSG00000012889 | Podnl1 |
| chr8 | 84132272 | G:A | ENSMUSG00000012889 | Podnl1 |
| chr8 | 84209682 | A:G | ENSMUSG00000097296 | Gm26532 |
| chr8 | 84246365 | C:T | ENSMUSG00000019362 | D8Ertd738e |
| chr8 | 84252728 | C:A | ENSMUSG00000004996 | Mri1 |
| chr8 | 84253561 | G:A | ENSMUSG00000004996 | Mri1 |
| chr8 | 84670106 | T:C | ENSMUSG00000001910 | Nacc1 |
| chr8 | 84670148 | T:C | ENSMUSG00000001910 | Nacc1 |
| chr8 | 84670264 | T:C | ENSMUSG00000001910 | Nacc1 |
| chr8 | 84671758 | CCA:C | ENSMUSG00000001910 | Nacc1 |
| chr8 | 84679280 | A:G | ENSMUSG00000001910 | Nacc1 |
| chr8 | 84686016 | G:A | ENSMUSG00000001910 | Nacc1 |
| chr8 | 84690021 | A:G | ENSMUSG00000001909 | Trmt1 |
| chr8 | 84698861 | C:A | ENSMUSG00000001909 | Trmt1 |
| chr8 | 84724951 | G:A | ENSMUSG00000074203 | G430095P16Rik |
| chr8 | 84725920 | C:T | ENSMUSG00000074203 | G430095P16Rik |
| chr8 | 84726317 | C:T | ENSMUSG00000074203 | G430095P16Rik |
| chr8 | 84861268 | A:G | ENSMUSG00000003808 | Farsa |
| chr8 | 84864676 | G:A | ENSMUSG00000003808 | Farsa |
| chr8 | 84887651 | C:T | ENSMUSG00000003824 | Syce2 |
| chr8 | 84997169 | A:T | ENSMUSG00000052566 | Hook2 |
| chr8 | 85020883 | C:T | ENSMUSG00000052456 | Asna1 |
| chr8 | 85024997 | T:C | ENSMUSG00000052456 | Asna1 |
| chr8 | 85034159 | C:T | ENSMUSG00000031691 | Tnpo2 |
| chr8 | 85066997 | T:C | ENSMUSG00000008167 | Fbxw9 |
| chr8 | 85067076 | TC:T | ENSMUSG00000008167 | Fbxw9 |
| chr8 | 85067953 | T:C | ENSMUSG00000095845 | Gm5741 |
| chr8 | 85070601 | ACCTTTT:A | ENSMUSG00000060038 | Dhps |
| chr8 | 85070850 | C:A | ENSMUSG00000060038 | Dhps |
| chr8 | 85071737 | G:A | ENSMUSG00000060038 | Dhps |
| chr8 | 85072796 | T:G | ENSMUSG00000060038 | Dhps |
| chr8 | 85073894 | A:G | ENSMUSG00000060038 | Dhps |
| chr8 | 85076315 | A:ATT | ENSMUSG00000005150 | Wdr83 |
| chr8 | 85076316 | C:G | ENSMUSG00000005150 | Wdr83 |
| chr8 | 85078582 | G:A | ENSMUSG00000005150 | Wdr83 |
| chr8 | 85086501 | T:A | ENSMUSG00000005142 | Man2b1 |
| chr8 | 85087476 | T:C | ENSMUSG00000005142 | Man2b1 |
| chr8 | 85091632 | A:T | ENSMUSG00000005142 | Man2b1 |
| chr8 | 85094652 | GCTCCCC:G | ENSMUSG00000005142 | Man2b1 |
| chr8 | 85303054 | A:G | ENSMUSG00000031697 | Orc6 |
| chr8 | 85518021 | G:C | ENSMUSG00000031700 | Gpt2 |
| chr8 | 85521350 | C:T | ENSMUSG00000031700 | Gpt2 |
| chr8 | 85526930 | T:C | ENSMUSG00000031700 | Gpt2 |
| chr8 | 85540615 | C:G | ENSMUSG00000031701 | Dnaja2 |
| chr8 | 85541016 | T:C | ENSMUSG00000031701 | Dnaja2 |
| chr8 | 85541636 | T:C | ENSMUSG00000031701 | Dnaja2 |
| chr8 | 85541961 | A:G | ENSMUSG00000031701 | Dnaja2 |
| chr8 | 85542118 | T:C | ENSMUSG00000031701 | Dnaja2 |
| chr8 | 85542597 | C:T | ENSMUSG00000031701 | Dnaja2 |
| chr8 | 85544503 | C:CA | ENSMUSG00000031701 | Dnaja2 |
| chr8 | 85546289 | G:A | ENSMUSG00000031701 | Dnaja2 |
| chr8 | 85554241 | C:G | ENSMUSG00000031701 | Dnaja2 |
| chr8 | 85841291 | T:C | ENSMUSG00000036879 | Phkb |
| chr8 | 85842275 | G:A | ENSMUSG00000036879 | Phkb |
| chr8 | 85937416 | G:A | ENSMUSG00000036879 | Phkb |
| chr8 | 88163759 | G:C | ENSMUSG00000031657 | Heatr3 |
| chr8 | 88255035 | C:T | ENSMUSG00000036779 | Tent4b |
| chr8 | 97164648 | A:G | ENSMUSG00000110715 | Gm5131 |
| chr9 | 35070069 | A:T | ENSMUSG00000032038 | St3gal4 |
| chr9 | 35098214 | A:G | ENSMUSG00000032038 | St3gal4 |
| chr9 | 35098326 | A:AG | ENSMUSG00000032038 | St3gal4 |
| chr9 | 36738780 | G:A | ENSMUSG00000032116 | Stt3a |
| chr9 | 37348134 | T:G | ENSMUSG00000034303 | Ccdc15 |
| chr9 | 37651591 | A:AG | ENSMUSG00000011114 | Tbrg1 |
| chr9 | 65902572 | A:G | ENSMUSG00000040204 | Pclaf |
| chr10 | 112925433 | C:T | ENSMUSG00000074748 | Atxn7l3b |
| chr10 | 4154510 | T:A | ENSMUSG00000040675 | Mthfd1l |
| chr10 | 4398164 | T:G | ENSMUSG00000095123 | Gm21781 |
| chr10 | 4398168 | A:G | ENSMUSG00000095123 | Gm21781 |
| chr10 | 4432327 | G:T | ENSMUSG00000019763 | Rmnd1 |
| chr10 | 77916969 | A:AT | ENSMUSG00000009292 | Trpm2 |
| chr10 | 13178391 | G:A | ENSMUSG00000019814 | Ltv1 |
| chr10 | 14403831 | A:G | ENSMUSG00000039116 | Adgrg6 |
| chr10 | 14403851 | T:A | ENSMUSG00000039116 | Adgrg6 |
| chr10 | 14404135 | T:A | ENSMUSG00000039116 | Adgrg6 |
| chr10 | 14404145 | T:C | ENSMUSG00000039116 | Adgrg6 |
| chr10 | 14404569 | T:A | ENSMUSG00000039116 | Adgrg6 |
| chr10 | 14404863 | A:G | ENSMUSG00000039116 | Adgrg6 |
| chr10 | 18019258 | T:C | ENSMUSG00000078453 | Abracl |
| chr11 | 101578871 | A:G | ENSMUSG00000017119 | Nbr1 |
| chr11 | 116123864 | T:C | ENSMUSG00000020773 | Trim47 |
| chr11 | 17944548 | C:A | ENSMUSG00000016984 | Etaa1 |
| chr11 | 3920136 | T:C | ENSMUSG00000020432 | Tcn2 |
| chr11 | 53399900 | C:CA | ENSMUSG00000049470 | Aff4 |
| chr11 | 79944351 | G:T | ENSMUSG00000035575 | Utp6 |
| chr12 | 113879380 | C:T | ENSMUSG00000096638 | Ighv2-9 |
| chr12 | 113896566 | C:T | ENSMUSG00000076665 | Ighv7-1 |
| chr12 | 115848145 | C:T | ENSMUSG00000093896 | Ighv1-76 |
| chr12 | 26326424 | G:T | ENSMUSG00000020642 | Rnf144a |
| chr12 | 26471162 | T:G | ENSMUSG00000020638 | Cmpk2 |
| chr12 | 28634415 | G:A | ENSMUSG00000061477 | Rps7 |
| chr12 | 28658700 | T:C | ENSMUSG00000020630 | Rnaseh1 |
| chr12 | 28856099 | A:G | ENSMUSG00000036613 | Eipr1 |
| chr12 | 32033121 | T:G | ENSMUSG00000002997 | Prkar2b |
| chr12 | 32847944 | T:G | ENSMUSG00000020572 | Nampt |
| chr12 | 33365082 | C:T | ENSMUSG00000020564 | Atxn7l1 |
| chr12 | 34047933 | TA:T | ENSMUSG00000004698 | Hdac9 |
| chr12 | 34048381 | TC:T | ENSMUSG00000004698 | Hdac9 |
| chr12 | 35139784 | A:C | ENSMUSG00000020590 | Snx13 |
| chr12 | 35499737 | C:T | ENSMUSG00000019256 | Ahr |
| chr12 | 40321187 | A:G | ENSMUSG00000055917 | Zfp277 |
| chr12 | 40321264 | T:C | ENSMUSG00000055917 | Zfp277 |
| chr12 | 40321604 | T:C | ENSMUSG00000055917 | Zfp277 |
| chr12 | 40321609 | T:C | ENSMUSG00000055917 | Zfp277 |
| chr12 | 40321801 | C:T | ENSMUSG00000055917 | Zfp277 |
| chr12 | 40322472 | C:T | ENSMUSG00000055917 | Zfp277 |
| chr12 | 40672844 | A:G | ENSMUSG00000035954 | Dock4 |
| chr12 | 4239002 | TC:T | ENSMUSG00000096199 | Ptrhd1 |
| chr12 | 51364684 | T:A | ENSMUSG00000035293 | G2e3 |
| chr12 | 51365873 | A:G | ENSMUSG00000035293 | G2e3 |
| chr12 | 51373038 | ATTT:A | ENSMUSG00000035293 | G2e3 |
| chr12 | 51444090 | C:T | ENSMUSG00000020952 | Scfd1 |
| chr12 | 51593770 | T:C | ENSMUSG00000020953 | Coch |
| chr12 | 51595483 | T:A | ENSMUSG00000020953 | Coch |
| chr12 | 51598285 | T:C | ENSMUSG00000020953 | Coch |
| chr12 | 51603510 | T:C | ENSMUSG00000020953 | Coch |
| chr12 | 51605034 | C:T | ENSMUSG00000020953 | Coch |
| chr12 | 51605135 | C:T | ENSMUSG00000020953 | Coch |
| chr12 | 51605406 | C:A | ENSMUSG00000020953 | Coch |
| chr12 | 51605503 | A:G | ENSMUSG00000020953 | Coch |
| chr12 | 51605691 | C:G | ENSMUSG00000020953 | Coch |
| chr12 | 51833358 | C:T | ENSMUSG00000035247 | Hectd1 |
| chr12 | 51885286 | T:C | ENSMUSG00000035181 | Heatr5a |
| chr12 | 51886298 | T:C | ENSMUSG00000035181 | Heatr5a |
| chr12 | 51886682 | T:C | ENSMUSG00000035181 | Heatr5a |
| chr12 | 51889316 | C:T | ENSMUSG00000035181 | Heatr5a |
| chr12 | 51959102 | A:G | ENSMUSG00000035181 | Heatr5a |
| chr12 | 51959303 | T:TA | ENSMUSG00000035181 | Heatr5a |
| chr12 | 51959402 | T:G | ENSMUSG00000035181 | Heatr5a |
| chr12 | 53450295 | G:A | ENSMUSG00000021010 | Npas3 |
| chr12 | 53501122 | C:T | ENSMUSG00000021010 | Npas3 |
| chr12 | 54048844 | A:G | ENSMUSG00000021010 | Npas3 |
| chr12 | 54067745 | T:C | ENSMUSG00000021010 | Npas3 |
| chr12 | 54067760 | C:A | ENSMUSG00000021010 | Npas3 |
| chr12 | 54068897 | C:T | ENSMUSG00000021010 | Npas3 |
| chr12 | 54069940 | A:T | ENSMUSG00000021010 | Npas3 |
| chr12 | 54070676 | T:C | ENSMUSG00000021010 | Npas3 |
| chr12 | 54071422 | A:AGAT | ENSMUSG00000021010 | Npas3 |
| chr13 | 90289706 | A:C | ENSMUSG00000102756 | Gm37054 |
| chr14 | 31381794 | T:C | ENSMUSG00000021892 | Sh3bp5 |
| chr14 | 31381816 | G:A | ENSMUSG00000021892 | Sh3bp5 |
| chr14 | 32182861 | G:A | ENSMUSG00000013701 | Timm23 |
| chr14 | 32298865 | T:C | ENSMUSG00000021911 | Parg |
| chr14 | 32752735 | T:G | ENSMUSG00000041707 | Tmem273 |
| chr14 | 32752788 | C:T | ENSMUSG00000041707 | Tmem273 |
| chr14 | 33021384 | G:T | ENSMUSG00000051506 | Wdfy4 |
| chr14 | 33380478 | A:G | ENSMUSG00000021936 | Mapk8 |
| chr14 | 34096546 | C:T | ENSMUSG00000095493 | A630023A22Rik |
| chr14 | 34097883 | G:A | ENSMUSG00000095493 | A630023A22Rik |
| chr14 | 34231585 | A:G | ENSMUSG00000115124 | Gm49201 |
| chr14 | 34234983 | C:A | ENSMUSG00000041471 | Shld2 |
| chr14 | 34236575 | T:C | ENSMUSG00000041471 | Shld2 |
| chr14 | 34314356 | A:T | ENSMUSG00000021794 | Glud1 |
| chr14 | 34315349 | A:G | ENSMUSG00000021794 | Glud1 |
| chr14 | 34315354 | CT:C | ENSMUSG00000021794 | Glud1 |
| chr14 | 34329528 | G:GA | ENSMUSG00000021794 | Glud1 |
| chr14 | 34330683 | A:G | ENSMUSG00000021794 | Glud1 |
| chr14 | 34330701 | T:C | ENSMUSG00000021794 | Glud1 |
| chr14 | 34333204 | GGTCT:G | ENSMUSG00000021794 | Glud1 |
| chr14 | 34333348 | T:C | ENSMUSG00000021794 | Glud1 |
| chr14 | 34338732 | T:C | ENSMUSG00000021794 | Glud1 |
| chr14 | 34339286 | A:G | ENSMUSG00000021794 | Glud1 |
| chr14 | 34341492 | C:T | ENSMUSG00000021794 | Glud1 |
| chr14 | 34341532 | T:C | ENSMUSG00000021794 | Glud1 |
| chr14 | 36941260 | A:G | ENSMUSG00000058690 | Ccser2 |
| chr14 | 45587622 | T:C | ENSMUSG00000085913 | Gm15601 |
| chr14 | 45587813 | C:CT | ENSMUSG00000085913 | Gm15601 |
| chr14 | 45589789 | C:T | ENSMUSG00000037697 | Ddhd1 |
| chr14 | 45589802 | A:G | ENSMUSG00000037697 | Ddhd1 |
| chr14 | 45597307 | T:C | ENSMUSG00000037697 | Ddhd1 |
| chr14 | 45598329 | C:T | ENSMUSG00000037697 | Ddhd1 |
| chr14 | 45598482 | T:C | ENSMUSG00000037697 | Ddhd1 |
| chr14 | 45613904 | A:T | ENSMUSG00000115148 | Gm49125 |
| chr14 | 45623741 | T:TTAAG | ENSMUSG00000037697 | Ddhd1 |
| chr14 | 45627494 | G:A | ENSMUSG00000037697 | Ddhd1 |
| chr14 | 45629468 | C:A | ENSMUSG00000037697 | Ddhd1 |
| chr14 | 46778715 | T:C | ENSMUSG00000015759 | Cnih1 |
| chr14 | 46814046 | TC:T | ENSMUSG00000062014 | Gmfb |
| chr14 | 46840418 | T:G | ENSMUSG00000055128 | Cgrrf1 |
| chr14 | 46840583 | G:A | ENSMUSG00000055128 | Cgrrf1 |
| chr14 | 46842494 | C:T | ENSMUSG00000055128 | Cgrrf1 |
| chr14 | 46846780 | T:C | ENSMUSG00000055128 | Cgrrf1 |
| chr14 | 46847939 | C:T | ENSMUSG00000055128 | Cgrrf1 |
| chr14 | 47246358 | T:C | ENSMUSG00000037572 | Wdhd1 |
| chr14 | 47251360 | G:C | ENSMUSG00000037572 | Wdhd1 |
| chr14 | 47268030 | C:G | ENSMUSG00000037572 | Wdhd1 |
| chr14 | 47322285 | T:G | ENSMUSG00000021840 | Mapk1ip1l |
| chr14 | 47543560 | C:G | ENSMUSG00000115823 | Gm49004 |
| chr14 | 47719654 | T:C | ENSMUSG00000021843 | Ktn1 |
| chr14 | 47729295 | T:C | ENSMUSG00000021843 | Ktn1 |
| chr14 | 47737476 | T:C | ENSMUSG00000021843 | Ktn1 |
| chr14 | 47739415 | T:C | ENSMUSG00000021843 | Ktn1 |
| chr14 | 48241292 | G:A | ENSMUSG00000021846 | Peli2 |
| chr14 | 48501176 | A:G | ENSMUSG00000036339 | Tmem260 |
| chr14 | 48511581 | G:C | ENSMUSG00000036339 | Tmem260 |
| chr14 | 49014430 | C:T | ENSMUSG00000061244 | Exoc5 |
| chr14 | 49081637 | G:A | ENSMUSG00000036291 | Ap5m1 |
| chr14 | 49081920 | A:G | ENSMUSG00000036291 | Ap5m1 |
| chr14 | 49173664 | T:C | ENSMUSG00000036282 | Naa30 |
| chr14 | 49173716 | T:C | ENSMUSG00000036282 | Naa30 |
| chr14 | 49174669 | A:G | ENSMUSG00000036282 | Naa30 |
| chr14 | 49176189 | G:A | ENSMUSG00000036282 | Naa30 |
| chr14 | 50774017 | T:A | ENSMUSG00000006288 | Ttc5 |
| chr14 | 50818433 | A:G | ENSMUSG00000036023 | Parp2 |
| chr14 | 50818851 | G:T | ENSMUSG00000036023 | Parp2 |
| chr14 | 50819902 | A:T | ENSMUSG00000036023 | Parp2 |
| chr14 | 50834793 | A:G | ENSMUSG00000006281 | Tep1 |
| chr14 | 50872593 | T:C | ENSMUSG00000097431 | Gm26782 |
| chr14 | 50889348 | A:G | ENSMUSG00000090799 | Klhl33 |
| chr14 | 50889350 | A:G | ENSMUSG00000090799 | Klhl33 |
| chr14 | 50912689 | G:C | ENSMUSG00000006289 | Osgep |
| chr14 | 50914661 | T:G | ENSMUSG00000006289 | Osgep |
| chr14 | 50920311 | C:A | ENSMUSG00000006289 | Osgep |
| chr14 | 50920634 | T:C | ENSMUSG00000006289 | Osgep |
| chr14 | 50920793 | T:A | ENSMUSG00000006289 | Osgep |
| chr14 | 50923664 | C:G | ENSMUSG00000006289 | Osgep |
| chr14 | 50930449 | T:C | ENSMUSG00000035953 | Pip4p1 |
| chr14 | 50930469 | A:G | ENSMUSG00000035953 | Pip4p1 |
| chr14 | 50948729 | C:A | ENSMUSG00000021871 | Gm49342 |
| chr14 | 50948734 | G:A | ENSMUSG00000021871 | Gm49342 |
| chr14 | 51096436 | G:A | ENSMUSG00000072115 | Ang |
| chr14 | 51101915 | T:G | ENSMUSG00000072115 | Ang |
| chr14 | 52016344 | A:G | ENSMUSG00000072571 | Tmem253 |
| chr14 | 52272390 | G:A | ENSMUSG00000022159 | Rab2b |
| chr14 | 52273706 | A:G | ENSMUSG00000022159 | Rab2b |
| chr14 | 52297251 | T:C | ENSMUSG00000022160 | Mettl3 |
| chr14 | 52311921 | C:A | ENSMUSG00000049532 | Sall2 |
| chr14 | 53797738 | T:C | ENSMUSG00000087666 | Trav13-5 |
| chr14 | 54139709 | G:A | ENSMUSG00000106112 | Gm43434 |
| chr14 | 54236847 | T:C | ENSMUSG00000022174 | Dad1 |
| chr14 | 54507977 | A:C | ENSMUSG00000023110 | Prmt5 |
| chr14 | 54546803 | T:A | ENSMUSG00000022177 | Haus4 |
| chr14 | 54546817 | GC:G | ENSMUSG00000022177 | Haus4 |
| chr14 | 54874388 | A:G | ENSMUSG00000072494 | Ppp1r3e |
| chr14 | 54888907 | G:C | ENSMUSG00000092232 | Gm20521 |
| chr14 | 54890393 | T:C | ENSMUSG00000092232 | Gm20521 |
| chr14 | 54890870 | T:C | ENSMUSG00000092232 | Gm20521 |
| chr14 | 54891731 | T:C | ENSMUSG00000092232 | Gm20521 |
| chr14 | 54891843 | G:A | ENSMUSG00000092232 | Gm20521 |
| chr14 | 55017172 | C:A | ENSMUSG00000022204 | Ngdn |
| chr14 | 55018244 | C:CT | ENSMUSG00000022204 | Ngdn |
| chr14 | 55022532 | C:T | ENSMUSG00000022204 | Ngdn |
| chr14 | 55067573 | A:C | ENSMUSG00000040721 | Zfhx2 |
| chr14 | 55068120 | C:T | ENSMUSG00000040721 | Zfhx2 |
| chr14 | 61612683 | C:T | ENSMUSG00000097589 | Dleu2 |
| chr14 | 62693850 | T:C | ENSMUSG00000035161 | Ints6 |
| chr14 | 63144541 | A:G | ENSMUSG00000021939 | Ctsb |
| chr14 | 63524649 | A:T | ENSMUSG00000035078 | Mtmr9 |
| chr14 | 65394668 | T:C | ENSMUSG00000034522 | Zfp395 |
| chr14 | 69243134 | TGGG:T | ENSMUSG00000034248 | Slc25a37 |
| chr14 | 69697818 | A:G | ENSMUSG00000034194 | R3hcc1 |
| chr14 | 7943204 | T:C | ENSMUSG00000025278 | Flnb |
| chr14 | 7944021 | C:T | ENSMUSG00000025278 | Flnb |
| chr14 | 7944065 | C:T | ENSMUSG00000025278 | Flnb |
| chr14 | 7945611 | T:C | ENSMUSG00000025278 | Flnb |
| chr14 | 7945624 | A:G | ENSMUSG00000025278 | Flnb |
| chr14 | 8056653 | C:T | ENSMUSG00000025277 | Abhd6 |
| chr14 | 8162296 | C:T | ENSMUSG00000033885 | Pxk |
| chr14 | 8165329 | G:T | ENSMUSG00000033885 | Pxk |
| chr14 | 8214985 | A:G | ENSMUSG00000021752 | Kctd6 |
| chr14 | 8217551 | G:A | ENSMUSG00000021752 | Kctd6 |
| chr14 | 13957841 | C:T | ENSMUSG00000053453 | Thoc7 |
| chr14 | 13960372 | C:G | ENSMUSG00000053453 | Thoc7 |
| chr14 | 14098198 | A:T | ENSMUSG00000021738 | Atxn7 |
| chr14 | 16295267 | C:T | ENSMUSG00000021785 | Ngly1 |
| chr14 | 16300908 | A:G | ENSMUSG00000021785 | Ngly1 |
| chr14 | 18214035 | T:C | ENSMUSG00000021775 | Nr1d2 |
| chr14 | 18283470 | G:A | ENSMUSG00000021774 | Ube2e1 |
| chr14 | 19879762 | A:G | ENSMUSG00000043004 | Gng2 |
| chr14 | 19884101 | C:T | ENSMUSG00000043004 | Gng2 |
| chr14 | 27000870 | G:A | ENSMUSG00000040726 | Hesx1 |
| chr14 | 27001445 | T:C | ENSMUSG00000040726 | Hesx1 |
| chr14 | 27001511 | G:A | ENSMUSG00000040726 | Hesx1 |
| chr14 | 27001896 | G:A | ENSMUSG00000040726 | Hesx1 |
| chr14 | 27002071 | G:A | ENSMUSG00000040726 | Hesx1 |
| chr14 | 27002073 | A:G | ENSMUSG00000040726 | Hesx1 |
| chr14 | 27002801 | T:G | ENSMUSG00000040726 | Hesx1 |
| chr14 | 27003084 | A:C | ENSMUSG00000040726 | Hesx1 |
| chr14 | 31366680 | C:T | ENSMUSG00000021892 | Sh3bp5 |
| chr14 | 31377107 | A:G | ENSMUSG00000021892 | Sh3bp5 |
| chr15 | 55255684 | A:G | ENSMUSG00000022419 | Deptor |
| chr15 | 85371021 | T:C | ENSMUSG00000016541 | Atxn10 |
| chr15 | 95941990 | T:G | ENSMUSG00000064210 | Ano6 |
| chr15 | 96584229 | A:G | ENSMUSG00000023169 | Slc38a1 |
| chr15 | 96589941 | T:G | ENSMUSG00000023169 | Slc38a1 |
| chr15 | 96638321 | G:A | ENSMUSG00000023169 | Slc38a1 |
| chr15 | 96643369 | G:A | ENSMUSG00000023169 | Slc38a1 |
| chr15 | 96694872 | C:T | ENSMUSG00000022462 | Slc38a2 |
| chr15 | 97801560 | A:G | ENSMUSG00000022475 | Hdac7 |
| chr15 | 97854782 | C:G | ENSMUSG00000022479 | Vdr |
| chr15 | 97856825 | T:C | ENSMUSG00000022479 | Vdr |
| chr15 | 97856826 | G:A | ENSMUSG00000022479 | Vdr |
| chr15 | 98136655 | T:C | ENSMUSG00000048175 | Asb8 |
| chr15 | 99405992 | T:C | ENSMUSG00000023010 | Tmbim6 |
| chr15 | 99422483 | A:G | ENSMUSG00000023009 | Nckap5l |
| chr15 | 99425633 | A:G | ENSMUSG00000023009 | Nckap5l |
| chr15 | 99425858 | C:T | ENSMUSG00000023009 | Nckap5l |
| chr16 | 11203472 | G:T | ENSMUSG00000097537 | 2610020C07Rik |
| chr16 | 55865709 | A:G | ENSMUSG00000075033 | Nxpe3 |
| chr16 | 57391298 | G:GAAAAAA | ENSMUSG00000022748 | Cmss1 |
| chr16 | 64762269 | T:C | ENSMUSG00000059920 | 4930453N24Rik |
| chr16 | 75601406 | A:G | ENSMUSG00000032940 | Rbm11 |
| chr16 | 97463249 | A:G | ENSMUSG00000000386 | Mx1 |
| chr17 | 14099574 | TC:T | ENSMUSG00000099476 | 4930488N24Rik |
| chr17 | 31433176 | A:G | ENSMUSG00000118157 | Gm9902 |
| chr17 | 31467198 | G:A | ENSMUSG00000041119 | Pde9a |
| chr17 | 31467564 | T:C | ENSMUSG00000041119 | Pde9a |
| chr17 | 31469366 | C:A | ENSMUSG00000041119 | Pde9a |
| chr17 | 31469381 | A:G | ENSMUSG00000041119 | Pde9a |
| chr17 | 31469437 | T:C | ENSMUSG00000041119 | Pde9a |
| chr17 | 31469755 | G:A | ENSMUSG00000041119 | Pde9a |
| chr17 | 31471362 | A:G | ENSMUSG00000041119 | Pde9a |
| chr17 | 3310923 | G:A | ENSMUSG00000116858 | Gm49797 |
| chr17 | 33304468 | C:T | ENSMUSG00000096910 | Zfp955b |
| chr17 | 33304481 | G:C | ENSMUSG00000096910 | Zfp955b |
| chr17 | 39845013 | T:G | ENSMUSG00000106106 | CT010467.1 |
| chr17 | 39848110 | T:C | ENSMUSG00000118642 | CT010467.2 |
| chr17 | 56176004 | T:C | ENSMUSG00000019579 | Mydgf |
| chr17 | 71228688 | G:A | ENSMUSG00000024052 | Lpin2 |
| chr17 | 71254664 | G:A | ENSMUSG00000024053 | Emilin2 |
| chr17 | 71265477 | G:A | ENSMUSG00000024053 | Emilin2 |
| chr17 | 71365890 | T:C | ENSMUSG00000024054 | Smchd1 |
| chr18 | 10002708 | C:T | ENSMUSG00000047879 | Usp14 |
| chr18 | 10010102 | T:C | ENSMUSG00000047879 | Usp14 |
| chr18 | 10069709 | G:T | ENSMUSG00000024290 | Rock1 |
| chr18 | 10070111 | G:T | ENSMUSG00000024290 | Rock1 |
| chr18 | 10557120 | A:G | ENSMUSG00000042942 | Greb1l |
| chr18 | 10558734 | G:A | ENSMUSG00000042942 | Greb1l |
| chr18 | 10623820 | C:T | ENSMUSG00000002477 | Snrpd1 |
| chr18 | 10623872 | C:T | ENSMUSG00000002477 | Snrpd1 |
| chr18 | 10625942 | C:T | ENSMUSG00000002477 | Snrpd1 |
| chr18 | 10625945 | A:G | ENSMUSG00000002477 | Snrpd1 |
| chr18 | 10626144 | T:C | ENSMUSG00000002477 | Snrpd1 |
| chr18 | 10647772 | A:C | ENSMUSG00000002475 | Abhd3 |
| chr18 | 11982459 | T:C | ENSMUSG00000049411 | Tmem241 |
| chr18 | 12137616 | A:C | ENSMUSG00000024404 | Riok3 |
| chr18 | 12138764 | A:G | ENSMUSG00000024404 | Riok3 |
| chr18 | 12140183 | T:C | ENSMUSG00000024404 | Riok3 |
| chr18 | 12140543 | T:G | ENSMUSG00000024404 | Riok3 |
| chr18 | 12140550 | C:T | ENSMUSG00000024404 | Riok3 |
| chr18 | 12140862 | T:A | ENSMUSG00000024404 | Riok3 |
| chr18 | 12141838 | G:T | ENSMUSG00000024404 | Riok3 |
| chr18 | 12146058 | G:A | ENSMUSG00000024404 | Riok3 |
| chr18 | 12146084 | TTGA:T | ENSMUSG00000024404 | Riok3 |
| chr18 | 12146375 | T:G | ENSMUSG00000024404 | Riok3 |
| chr18 | 12153465 | C:T | ENSMUSG00000024404 | Riok3 |
| chr18 | 12154284 | T:C | ENSMUSG00000024404 | Riok3 |
| chr18 | 12154744 | GC:G | ENSMUSG00000024404 | Riok3 |
| chr18 | 12154773 | A:T | ENSMUSG00000024404 | Riok3 |
| chr18 | 12180588 | A:G | ENSMUSG00000024410 | Rmc1 |
| chr18 | 12189967 | CA:C | ENSMUSG00000024413 | Npc1 |
| chr18 | 12190114 | A:G | ENSMUSG00000024413 | Npc1 |
| chr18 | 12210061 | C:T | ENSMUSG00000024413 | Npc1 |
| chr18 | 12643527 | G:A | ENSMUSG00000117418 | Gm50063 |
| chr18 | 12686954 | C:T | ENSMUSG00000024424 | Ttc39c |
| chr18 | 12728664 | C:T | ENSMUSG00000024424 | Ttc39c |
| chr18 | 12926462 | A:C | ENSMUSG00000044252 | Osbpl1a |
| chr18 | 12927590 | C:T | ENSMUSG00000044252 | Osbpl1a |
| chr18 | 12927971 | A:C | ENSMUSG00000044252 | Osbpl1a |
| chr18 | 12941650 | G:C | ENSMUSG00000044252 | Osbpl1a |
| chr18 | 12957810 | G:A | ENSMUSG00000024423 | Impact |
| chr18 | 12959499 | T:C | ENSMUSG00000024423 | Impact |
| chr18 | 12965624 | T:C | ENSMUSG00000024423 | Impact |
| chr18 | 12967918 | G:T | ENSMUSG00000024423 | Impact |
| chr18 | 12971989 | T:C | ENSMUSG00000024423 | Impact |
| chr18 | 12979939 | G:A | ENSMUSG00000024423 | Impact |
| chr18 | 14571023 | C:T | ENSMUSG00000053740 | Gm6457 |
| chr18 | 14762131 | T:C | ENSMUSG00000036743 | Psma8 |
| chr18 | 20699319 | T:C | ENSMUSG00000056124 | B4galt6 |
| chr18 | 20829356 | A:G | ENSMUSG00000033382 | Trappc8 |
| chr18 | 30342741 | A:G | ENSMUSG00000033628 | Pik3c3 |
| chr18 | 31597312 | G:A | ENSMUSG00000024259 | Slc25a46 |
| chr18 | 31618829 | C:G | ENSMUSG00000092124 | B930094E09Rik |
| chr18 | 31865295 | C:T | ENSMUSG00000024400 | Wdr33 |
| chr18 | 31907862 | T:C | ENSMUSG00000024400 | Wdr33 |
| chr18 | 33795620 | C:T | ENSMUSG00000087590 | Epb41l4aos |
| chr18 | 33795621 | T:C | ENSMUSG00000087590 | Epb41l4aos |
| chr18 | 34333007 | G:A | ENSMUSG00000014504 | Srp19 |
| chr18 | 34635017 | A:AT | ENSMUSG00000024370 | Cdc23 |
| chr18 | 34764161 | A:G | ENSMUSG00000034300 | Fam53c |
| chr18 | 34813368 | T:C | ENSMUSG00000038773 | Kdm3b |
| chr18 | 34917804 | G:T | ENSMUSG00000024360 | Etf1 |
| chr18 | 34932802 | AT:A | ENSMUSG00000024360 | Etf1 |
| chr18 | 34933786 | G:A | ENSMUSG00000024360 | Etf1 |
| chr18 | 34933978 | A:G | ENSMUSG00000024360 | Etf1 |
| chr18 | 35650677 | T:C | ENSMUSG00000117874 | Gm50318 |
| chr18 | 35671710 | T:TTTCC | ENSMUSG00000024350 | Dnajc18 |
| chr18 | 35671925 | C:G | ENSMUSG00000024350 | Dnajc18 |
| chr18 | 35673317 | TA:T | ENSMUSG00000024350 | Dnajc18 |
| chr18 | 35673421 | C:T | ENSMUSG00000024350 | Dnajc18 |
| chr18 | 35735360 | C:T | ENSMUSG00000024349 | Sting1 |
| chr18 | 35736698 | C:T | ENSMUSG00000024349 | Sting1 |
| chr18 | 35737513 | T:C | ENSMUSG00000024349 | Sting1 |
| chr18 | 35737668 | T:C | ENSMUSG00000024349 | Sting1 |
| chr18 | 35738026 | T:A | ENSMUSG00000024349 | Sting1 |
| chr18 | 35738465 | G:GA | ENSMUSG00000024349 | Sting1 |
| chr18 | 36015804 | G:A | ENSMUSG00000024347 | Psd2 |
| chr18 | 36015821 | G:A | ENSMUSG00000024347 | Psd2 |
| chr18 | 36015860 | A:G | ENSMUSG00000024347 | Psd2 |
| chr18 | 36016006 | T:C | ENSMUSG00000024347 | Psd2 |
| chr18 | 36033743 | C:A | ENSMUSG00000060275 | Nrg2 |
| chr18 | 36660759 | C:T | ENSMUSG00000117942 | Maskbp3 |
| chr18 | 36679731 | A:C | ENSMUSG00000033272 | Slc35a4 |
| chr18 | 36747459 | A:C | ENSMUSG00000024474 | Ik |
| chr18 | 36767035 | T:C | ENSMUSG00000001380 | Hars |
| chr18 | 37846857 | A:G | ENSMUSG00000024456 | Diaph1 |
| chr18 | 37846996 | C:A | ENSMUSG00000024456 | Diaph1 |
| chr18 | 37851507 | GC:G | ENSMUSG00000024456 | Diaph1 |
| chr18 | 37854141 | G:A | ENSMUSG00000024456 | Diaph1 |
| chr18 | 37974779 | C:A | ENSMUSG00000024451 | Arap3 |
| chr18 | 37990624 | A:G | ENSMUSG00000024451 | Arap3 |
| chr18 | 38259100 | C:G | ENSMUSG00000024442 | Dele1 |
| chr18 | 38259241 | T:G | ENSMUSG00000024442 | Dele1 |
| chr18 | 38260773 | G:A | ENSMUSG00000024442 | Dele1 |
| chr18 | 38307632 | A:G | ENSMUSG00000060450 | Rnf14 |
| chr18 | 38318748 | G:A | ENSMUSG00000060450 | Rnf14 |
| chr18 | 38318782 | G:T | ENSMUSG00000060450 | Rnf14 |
| chr18 | 38331776 | A:G | ENSMUSG00000052102 | Gnpda1 |
| chr18 | 38332792 | A:G | ENSMUSG00000052102 | Gnpda1 |
| chr18 | 38332795 | C:A | ENSMUSG00000052102 | Gnpda1 |
| chr18 | 38334603 | G:T | ENSMUSG00000052102 | Gnpda1 |
| chr18 | 38334608 | T:C | ENSMUSG00000052102 | Gnpda1 |
| chr18 | 38336113 | T:G | ENSMUSG00000052102 | Gnpda1 |
| chr18 | 38359699 | G:A | ENSMUSG00000118140 | Gm4949 |
| chr18 | 38359706 | C:T | ENSMUSG00000118140 | Gm4949 |
| chr18 | 38449000 | A:G | ENSMUSG00000024425 | Ndfip1 |
| chr18 | 38449005 | T:C | ENSMUSG00000024425 | Ndfip1 |
| chr18 | 38450570 | C:T | ENSMUSG00000024425 | Ndfip1 |
| chr18 | 38451072 | T:A | ENSMUSG00000024425 | Ndfip1 |
| chr18 | 38460474 | A:G | ENSMUSG00000024425 | Ndfip1 |
| chr18 | 38461008 | T:C | ENSMUSG00000024425 | Ndfip1 |
| chr18 | 39357853 | C:T | ENSMUSG00000036452 | Arhgap26 |
| chr18 | 50057718 | G:A | ENSMUSG00000062210 | Tnfaip8 |
| chr18 | 50058271 | A:G | ENSMUSG00000062210 | Tnfaip8 |
| chr18 | 50063188 | C:G | ENSMUSG00000062210 | Tnfaip8 |
| chr18 | 50063216 | T:G | ENSMUSG00000062210 | Tnfaip8 |
| chr18 | 50067473 | G:A | ENSMUSG00000062210 | Tnfaip8 |
| chr18 | 50067474 | C:T | ENSMUSG00000062210 | Tnfaip8 |
| chr18 | 5063594 | T:C | ENSMUSG00000024236 | Svil |
| chr18 | 53383253 | G:A | ENSMUSG00000024535 | Snx24 |
| chr18 | 53386456 | C:T | ENSMUSG00000024535 | Snx24 |
| chr18 | 53387902 | A:G | ENSMUSG00000024535 | Snx24 |
| chr18 | 57384338 | A:C | ENSMUSG00000086607 | 4930511M06Rik |
| chr18 | 57388230 | C:T | ENSMUSG00000086607 | 4930511M06Rik |
| chr18 | 57391477 | G:GCA | ENSMUSG00000086607 | 4930511M06Rik |
| chr18 | 60392083 | T:C | ENSMUSG00000054072 | Iigp1 |
| chr18 | 60817338 | C:T | ENSMUSG00000024613 | Tcof1 |
| chr18 | 60918366 | G:A | ENSMUSG00000036412 | Arsi |
| chr18 | 61297721 | T:C | ENSMUSG00000033871 | Ppargc1b |
| chr18 | 61503254 | G:A | ENSMUSG00000045094 | Arhgef37 |
| chr18 | 6207128 | C:A | ENSMUSG00000006740 | Kif5b |
| chr18 | 62187348 | G:A | ENSMUSG00000054589 | Gm9949 |
| chr18 | 62187841 | T:C | ENSMUSG00000054589 | Gm9949 |
| chr18 | 62542607 | C:T | ENSMUSG00000042211 | Fbxo38 |
| chr18 | 63666386 | G:A | ENSMUSG00000024583 | Txnl1 |
| chr18 | 6445660 | T:C | ENSMUSG00000024240 | Epc1 |
| chr18 | 64501060 | A:G | ENSMUSG00000024587 | Nars |
| chr18 | 64501156 | T:C | ENSMUSG00000024587 | Nars |
| chr18 | 65444344 | T:A | ENSMUSG00000032688 | Malt1 |
| chr18 | 65455244 | A:G | ENSMUSG00000032688 | Malt1 |
| chr18 | 67647086 | A:AC | ENSMUSG00000024537 | Psmg2 |
| chr18 | 67666980 | A:T | ENSMUSG00000024539 | Ptpn2 |
| chr18 | 67669025 | A:G | ENSMUSG00000024539 | Ptpn2 |
| chr18 | 67671918 | T:A | ENSMUSG00000024539 | Ptpn2 |
| chr18 | 67789839 | T:A | ENSMUSG00000079614 | Seh1l |
| chr18 | 68449187 | A:G | ENSMUSG00000118288 | Gm50258 |
| chr18 | 69680109 | A:C | ENSMUSG00000053477 | Tcf4 |
| chr18 | 69681869 | C:A | ENSMUSG00000053477 | Tcf4 |
| chr18 | 69682469 | A:G | ENSMUSG00000053477 | Tcf4 |
| chr18 | 69683864 | AT:A | ENSMUSG00000053477 | Tcf4 |
| chr18 | 69684302 | T:A | ENSMUSG00000053477 | Tcf4 |
| chr18 | 69687560 | T:A | ENSMUSG00000053477 | Tcf4 |
| chr18 | 73643301 | C:T | ENSMUSG00000024515 | Smad4 |
| chr18 | 73735843 | T:C | ENSMUSG00000036941 | Elac1 |
| chr18 | 73736329 | G:T | ENSMUSG00000036941 | Elac1 |
| chr18 | 73739359 | T:C | ENSMUSG00000036941 | Elac1 |
| chr18 | 73740753 | A:G | ENSMUSG00000036941 | Elac1 |
| chr18 | 73769700 | CT:C | ENSMUSG00000024556 | Me2 |
| chr18 | 73812072 | C:T | ENSMUSG00000024556 | Me2 |
| chr18 | 74201291 | A:G | ENSMUSG00000036223 | Ska1 |
| chr18 | 75000596 | T:C | ENSMUSG00000062328 | Rpl17 |
| chr18 | 75000637 | A:G | ENSMUSG00000062328 | Rpl17 |
| chr18 | 75002881 | T:A | ENSMUSG00000062328 | Rpl17 |
| chr18 | 75009156 | G:A | ENSMUSG00000036299 | BC031181 |
| chr18 | 77874445 | A:C | ENSMUSG00000025429 | Pstpip2 |
| chr18 | 9848423 | C:T | ENSMUSG00000036103 | Colec12 |
| chr18 | 9848456 | A:G | ENSMUSG00000036103 | Colec12 |
| chr18 | 9959908 | A:G | ENSMUSG00000024287 | Thoc1 |
| chr18 | 9992291 | C:A | ENSMUSG00000024287 | Thoc1 |
| chr19 | 11426832 | A:G | ENSMUSG00000024675 | Ms4a4c |
| chr19 | 17221071 | A:G | ENSMUSG00000039126 | Prune2 |
| chr19 | 17221139 | A:G | ENSMUSG00000039126 | Prune2 |
| chr19 | 17221282 | A:G | ENSMUSG00000039126 | Prune2 |
| chr19 | 17221840 | G:A | ENSMUSG00000039126 | Prune2 |
| chr19 | 17223452 | G:C | ENSMUSG00000039126 | Prune2 |
| chr19 | 18591177 | T:C | ENSMUSG00000024725 | Ostf1 |
| chr19 | 21408893 | G:T | ENSMUSG00000058624 | Gda |
| chr19 | 25168535 | T:C | ENSMUSG00000052085 | Dock8 |
| chr19 | 25191955 | A:G | ENSMUSG00000052085 | Dock8 |
| chr19 | 27764914 | C:T | ENSMUSG00000040929 | Rfx3 |
| chr19 | 29351367 | G:A | ENSMUSG00000016495 | Plgrkt |
| chr19 | 29352302 | G:A | ENSMUSG00000016495 | Plgrkt |
| chr19 | 29353297 | G:GCT | ENSMUSG00000016495 | Plgrkt |
| chr19 | 29357614 | T:C | ENSMUSG00000016495 | Plgrkt |
| chr19 | 29383648 | G:A | ENSMUSG00000016496 | Cd274 |
| chr19 | 32014247 | A:G | ENSMUSG00000024887 | Asah2 |
| chr19 | 34593600 | A:G | ENSMUSG00000079339 | Ifit1bl1 |
| chr19 | 37986003 | G:A | ENSMUSG00000048612 | Myof |
| chr19 | 38055168 | A:G | ENSMUSG00000024989 | Cep55 |
| chr19 | 38055181 | A:G | ENSMUSG00000024989 | Cep55 |
| chr19 | 38070719 | A:G | ENSMUSG00000024989 | Cep55 |
| chr19 | 38222774 | A:C | ENSMUSG00000054237 | Fra10ac1 |
| chr19 | 38223278 | T:A | ENSMUSG00000054237 | Fra10ac1 |
| chr19 | 41926471 | G:A | ENSMUSG00000034321 | Exosc1 |
| chr19 | 41928593 | C:T | ENSMUSG00000034321 | Exosc1 |
| chr19 | 41929818 | G:A | ENSMUSG00000034321 | Exosc1 |
| chr19 | 41931440 | A:G | ENSMUSG00000034321 | Exosc1 |
| chr19 | 41932827 | C:T | ENSMUSG00000034321 | Exosc1 |
| chr19 | 42013958 | G:A | ENSMUSG00000025171 | Ubtd1 |
| chr19 | 42593841 | G:A | ENSMUSG00000025185 | Loxl4 |
| chr19 | 42594458 | A:G | ENSMUSG00000025185 | Loxl4 |
| chr19 | 42594485 | A:G | ENSMUSG00000025185 | Loxl4 |
| chr19 | 42602183 | G:A | ENSMUSG00000025185 | Loxl4 |
| chr19 | 42606704 | C:T | ENSMUSG00000025185 | Loxl4 |
| chr19 | 42607655 | C:T | ENSMUSG00000025185 | Loxl4 |
| chr19 | 42608346 | G:A | ENSMUSG00000025185 | Loxl4 |
| chr19 | 42608417 | T:C | ENSMUSG00000025185 | Loxl4 |
| chr19 | 42608463 | G:A | ENSMUSG00000025185 | Loxl4 |
| chr19 | 42737219 | TG:T | ENSMUSG00000060224 | Pyroxd2 |
| chr19 | 42737321 | GCA:G | ENSMUSG00000060224 | Pyroxd2 |
| chr19 | 42737392 | C:T | ENSMUSG00000060224 | Pyroxd2 |
| chr19 | 42737485 | CA:C | ENSMUSG00000060224 | Pyroxd2 |
| chr19 | 42737487 | CA:C | ENSMUSG00000060224 | Pyroxd2 |
| chr19 | 42748073 | A:T | ENSMUSG00000118354 | Gm19437 |
| chr19 | 42748182 | A:G | ENSMUSG00000118354 | Gm19437 |
| chr19 | 42748187 | C:A | ENSMUSG00000118354 | Gm19437 |
| chr19 | 42749079 | G:T | ENSMUSG00000118354 | Gm19437 |
| chr19 | 42749506 | C:A | ENSMUSG00000118354 | Gm19437 |
| chr19 | 42749532 | A:G | ENSMUSG00000118354 | Gm19437 |
| chr19 | 42749562 | C:T | ENSMUSG00000118354 | Gm19437 |
| chr19 | 42749608 | C:T | ENSMUSG00000060224 | Pyroxd2 |
| chr19 | 42750161 | T:C | ENSMUSG00000060224 | Pyroxd2 |
| chr19 | 42752591 | G:C | ENSMUSG00000060224 | Pyroxd2 |
| chr19 | 42758175 | C:T | ENSMUSG00000025188 | Hps1 |
| chr19 | 42758196 | A:C | ENSMUSG00000025188 | Hps1 |
| chr19 | 42758663 | C:T | ENSMUSG00000025188 | Hps1 |
| chr19 | 42759162 | C:T | ENSMUSG00000025188 | Hps1 |
| chr19 | 42759200 | C:A | ENSMUSG00000025188 | Hps1 |
| chr19 | 42766725 | ATCC:A | ENSMUSG00000025188 | Hps1 |
| chr19 | 43505812 | G:A | ENSMUSG00000025190 | Got1 |
| chr19 | 43836746 | A:G | ENSMUSG00000025194 | Abcc2 |
| chr19 | 43838137 | T:C | ENSMUSG00000025194 | Abcc2 |
| chr19 | 44069465 | G:A | ENSMUSG00000025198 | Erlin1 |
| chr19 | 44079558 | T:G | ENSMUSG00000025199 | Chuk |
| chr19 | 44109425 | A:G | ENSMUSG00000025200 | Cwf19l1 |
| chr19 | 45021411 | A:G | ENSMUSG00000035342 | Lzts2 |
| chr19 | 45055658 | ACC:A | ENSMUSG00000025212 | Sfxn3 |
| chr19 | 46360749 | A:G | ENSMUSG00000025227 | Mfsd13a |
| chr19 | 46596493 | A:G | ENSMUSG00000025036 | Sfxn2 |
| chr19 | 47113437 | AGAGGAG:A | ENSMUSG00000025047 | Pdcd11 |
| chr19 | 47128794 | G:T | ENSMUSG00000025047 | Pdcd11 |
| chr19 | 47129328 | A:G | ENSMUSG00000025047 | Pdcd11 |
| chr19 | 47129339 | C:T | ENSMUSG00000025047 | Pdcd11 |
| chr19 | 47860859 | A:G | ENSMUSG00000044948 | Cfap43 |
| chr19 | 47862736 | C:A | ENSMUSG00000044948 | Cfap43 |
| chr19 | 53387469 | T:C | ENSMUSG00000025024 | Smndc1 |
| chr19 | 57034895 | T:C | ENSMUSG00000025085 | Ablim1 |
| chr19 | 57034896 | TTGTTTGTTTC:T | ENSMUSG00000025085 | Ablim1 |
| chr19 | 59904597 | A:G | ENSMUSG00000040022 | Rab11fip2 |
| chr19 | 60532827 | G:A | ENSMUSG00000033417 | Cacul1 |
| chr19 | 60582908 | A:G | ENSMUSG00000033417 | Cacul1 |
| chr19 | 60830708 | G:A | ENSMUSG00000024993 | Fam45a |
| chr19 | 60836073 | T:C | ENSMUSG00000024993 | Fam45a |
| chr19 | 60836074 | C:T | ENSMUSG00000024993 | Fam45a |
| chr19 | 60865590 | T:A | ENSMUSG00000024997 | Prdx3 |
| chr19 | 61126674 | T:C | ENSMUSG00000074733 | Zfp950 |
| chrGL456221.1 | 113467 | A:G | ENSMUSG00000062783 | Csprs |
| chrJH584304.1 | 48591 | C:T | ENSMUSG00000095041 | AC149090.1 |
| chrX | 152910095 | C:A | ENSMUSG00000083440 | Rpl7a-ps12 |
| chrX | 155504004 | A:G | ENSMUSG00000083362 | Gm8606 |
| chrX | 155504008 | A:C | ENSMUSG00000083362 | Gm8606 |
| chrX | 74988109 | T:C | ENSMUSG00000032750 | Gab3 |
| chrY | 1016750 | GT:G | ENSMUSG00000069049 | Eif2s3y |
| chrY | 4389605 | ACT:A | ENSMUSG00000102088 | Gm21064 |
