# Supplementary material for: CDX2 expression in the hematopoietic lineage promotes leukemogenesis via TGFβ inhibition
Source: Mol Oncol. 2021 Jun 26;15(9):2318–29. doi: 10.1002/1878-0261.12982 (PMC8410536; doi:10.1002/1878-0261.12982)
Supplement: Supplementary file 8 — Table S7. Prediction of the consequences of the nucleotide changes in MxCDX2 mice. [file MOL2-15-2318-s004.pdf]

Table S6

Prediction of the consequences of the nucleotide changes in *MxCDX2* mice

Locus	Consequence	Impact	Gene symbole	Gene identifier	Feature	Feature identifier	Biotype	Mutation identifier
1:138121809-138121810	downstream_gene_variant	MODIFIER	Ptprc	ENSMUSG00000026395	Transcript	ENSMUST00000182138	retained_intron	rs254298739
1:138121809-138121810	intron_variant	MODIFIER	Ptprc	ENSMUSG00000026395	Transcript	ENSMUST00000182283	protein_coding	rs254298739
1:138121809-138121810	intron_variant	MODIFIER	Ptprc	ENSMUSG00000026395	Transcript	ENSMUST00000182755	protein_coding	rs254298739
1:138121809-138121810	downstream_gene_variant	MODIFIER	Ptprc	ENSMUSG00000026395	Transcript	ENSMUST00000183229	retained_intron	rs254298739
1:138121809-138121810	intron_variant,non_coding_trans	MODIFIER	Ptprc	ENSMUSG00000026395	Transcript	ENSMUST00000183262	retained_intron	rs254298739
1:138121809-138121810	intron_variant	MODIFIER	Ptprc	ENSMUSG00000026395	Transcript	ENSMUST00000183301	protein_coding	rs254298739
1:138121809-138121810	upstream_gene_variant	MODIFIER	Ptprc	ENSMUSG00000026395	Transcript	ENSMUST00000187586	retained_intron	rs254298739
1:138121809-138121810	intron_variant	MODIFIER	Ptprc	ENSMUSG00000026395	Transcript	ENSMUST00000193650	protein_coding	rs254298739
1:138121809-138121810	intron_variant	MODIFIER	Ptprc	ENSMUSG00000026395	Transcript	ENSMUST00000195533	protein_coding	rs254298739
1:170288954-170288954	downstream_gene_variant	MODIFIER	Sh2d1b1	ENSMUSG00000102418	Transcript	ENSMUST00000179976	protein_coding	-
1:171701954-171701954	intron_variant	MODIFIER	Cd48	ENSMUSG00000015355	Transcript	ENSMUST00000015499	protein_coding	rs864281451
1:171701954-171701954	intron_variant	MODIFIER	Cd48	ENSMUSG00000015355	Transcript	ENSMUST00000068584	protein_coding	rs864281451
1:66708747-66708747	intron_variant	MODIFIER	Rpe	ENSMUSG00000026005	Transcript	ENSMUST00000027157	protein_coding	rs32055350
1:66708747-66708747	intron_variant	MODIFIER	Rpe	ENSMUSG00000026005	Transcript	ENSMUST00000113995	protein_coding	rs32055350
1:66708747-66708747	intron_variant,NMD_transcript_v	MODIFIER	Rpe	ENSMUSG00000026005	Transcript	ENSMUST00000142920	nonsense_mediated_decay	rs32055350
1:66708747-66708747	intron_variant,NMD_transcript_v	MODIFIER	Rpe	ENSMUSG00000026005	Transcript	ENSMUST00000151016	nonsense_mediated_decay	rs32055350
1:66708747-66708747	intron_variant,NMD_transcript_v	MODIFIER	Rpe	ENSMUSG00000026005	Transcript	ENSMUST00000190404	nonsense_mediated_decay	rs32055350
1:67002256-67002256	3_prime_UTR_variant	MODIFIER	Lancl1	ENSMUSG00000026000	Transcript	ENSMUST00000027149	protein_coding	rs30339595
1:67002256-67002256	3_prime_UTR_variant	MODIFIER	Lancl1	ENSMUSG00000026000	Transcript	ENSMUST00000113979	protein_coding	rs30339595
1:67002256-67002256	downstream_gene_variant	MODIFIER	Lancl1	ENSMUSG00000026000	Transcript	ENSMUST00000119559	protein_coding	rs30339595
1:67002256-67002256	downstream_gene_variant	MODIFIER	Lancl1	ENSMUSG00000026000	Transcript	ENSMUST00000133508	processed_transcript	rs30339595
1:67002256-67002256	regulatory_region_variant	MODIFIER	-	-	RegulatoryFeature	ENSMUSR00000481899	enhancer	rs30339595
1:91291262-91291262	upstream_gene_variant	MODIFIER	Gm7785	ENSMUSG00000083064	Transcript	ENSMUST00000117121	processed_pseudogene	rs50517452
1:91291262-91291262	downstream_gene_variant	MODIFIER	Ube2f	ENSMUSG00000034343	Transcript	ENSMUST00000171165	protein_coding	rs50517452
1:94038650-94038650	3_prime_UTR_variant	MODIFIER	Pdcd1	ENSMUSG00000026285	Transcript	ENSMUST00000027507	protein_coding	rs246171206
1:99190350-99190350	intergenic_variant	MODIFIER	-	-	-	-	-	-
1:99790318-99790318	intron_variant	MODIFIER	Cntnap5b	ENSMUSG00000067028	Transcript	ENSMUST00000086738	protein_coding	rs48936094
1:99790318-99790318	downstream_gene_variant	MODIFIER	Tdpx-ps1	ENSMUSG00000082431	Transcript	ENSMUST00000117372	processed_pseudogene	rs48936094
1:99790342-99790342	intron_variant	MODIFIER	Cntnap5b	ENSMUSG00000067028	Transcript	ENSMUST00000086738	protein_coding	-
1:99790342-99790342	downstream_gene_variant	MODIFIER	Tdpx-ps1	ENSMUSG00000082431	Transcript	ENSMUST00000117372	processed_pseudogene	-
1:33583113-33583113	intron_variant	MODIFIER	Prim2	ENSMUSG00000026134	Transcript	ENSMUST00000027312	protein_coding	rs32088204
1:33583113-33583113	intron_variant,non_coding_trans	MODIFIER	Prim2	ENSMUSG00000026134	Transcript	ENSMUST00000189552	retained_intron	rs32088204
1:37443158-37443158	downstream_gene_variant	MODIFIER	Unc50	ENSMUSG00000026111	Transcript	ENSMUST00000027285	protein_coding	-
1:37443158-37443158	3_prime_UTR_variant	MODIFIER	Mgat4a	ENSMUSG00000026110	Transcript	ENSMUST00000042161	protein_coding	-
1:37443158-37443158	downstream_gene_variant	MODIFIER	Unc50	ENSMUSG00000026111	Transcript	ENSMUST00000114925	protein_coding	-
1:37443158-37443158	downstream_gene_variant	MODIFIER	Unc50	ENSMUSG00000026111	Transcript	ENSMUST00000118059	protein_coding	-
1:37443158-37443158	downstream_gene_variant	MODIFIER	Mgat4a	ENSMUSG00000026110	Transcript	ENSMUST00000143636	protein_coding	-
1:37443158-37443158	3_prime_UTR_variant	MODIFIER	Mgat4a	ENSMUSG00000026110	Transcript	ENSMUST00000151952	protein_coding	-
1:37443158-37443158	downstream_gene_variant	MODIFIER	Mgat4a	ENSMUSG00000026110	Transcript	ENSMUST00000154819	protein_coding	-
1:43050804-43050804	intron_variant	MODIFIER	Tgfbrap1	ENSMUSG00000070939	Transcript	ENSMUST00000095014	protein_coding	rs48773819
1:43050804-43050804	intron_variant	MODIFIER	Tgfbrap1	ENSMUSG00000070939	Transcript	ENSMUST00000186694	protein_coding	rs48773819
1:43050804-43050804	downstream_gene_variant	MODIFIER	Tgfbrap1	ENSMUSG00000070939	Transcript	ENSMUST00000188598	retained_intron	rs48773819
2:110462591-110462591	intergenic_variant	MODIFIER	-	-	-	-	-	-
2:121543632-121543632	downstream_gene_variant	MODIFIER	Wdr76	ENSMUSG00000027242	Transcript	ENSMUST00000028676	protein_coding	-
2:121543632-121543632	downstream_gene_variant	MODIFIER	Frmd5	ENSMUSG00000027238	Transcript	ENSMUST00000110592	protein_coding	-
2:121543632-121543632	downstream_gene_variant	MODIFIER	Frmd5	ENSMUSG00000027238	Transcript	ENSMUST00000110593	protein_coding	-
2:121543632-121543632	3_prime_UTR_variant	MODIFIER	Wdr76	ENSMUSG00000027242	Transcript	ENSMUST00000110602	protein_coding	-
2:121543632-121543632	downstream_gene_variant	MODIFIER	Wdr76	ENSMUSG00000027242	Transcript	ENSMUST00000110603	protein_coding	-
2:121543632-121543632	downstream_gene_variant	MODIFIER	Frmd5	ENSMUSG00000027238	Transcript	ENSMUST00000121219	protein_coding	-
2:121543632-121543632	downstream_gene_variant	MODIFIER	Frmd5	ENSMUSG00000027238	Transcript	ENSMUST00000128428	nonsense_mediated_decay	-
2:121543632-121543632	downstream_gene_variant	MODIFIER	Frmd5	ENSMUSG00000027238	Transcript	ENSMUST00000131092	protein_coding	-
2:121543632-121543632	downstream_gene_variant	MODIFIER	Frmd5	ENSMUSG00000027238	Transcript	ENSMUST00000133898	protein_coding	-
2:121543632-121543632	downstream_gene_variant	MODIFIER	Frmd5	ENSMUSG00000027238	Transcript	ENSMUST00000138157	protein_coding	-
2:121543632-121543632	downstream_gene_variant	MODIFIER	Frmd5	ENSMUSG00000027238	Transcript	ENSMUST00000155570	protein_coding	-
2:121543632-121543632	downstream_gene_variant	MODIFIER	Frmd5	ENSMUSG00000027238	Transcript	ENSMUST00000212518	protein_coding	-
2:155283614-155283614	intron_variant	MODIFIER	Pigu	ENSMUSG00000038383	Transcript	ENSMUST00000077626	protein_coding	rs27310833
2:155283614-155283614	intron_variant,non_coding_trans	MODIFIER	Pigu	ENSMUSG00000038383	Transcript	ENSMUST00000139854	processed_transcript	rs27310833
2:155283614-155283614	intron_variant	MODIFIER	Pigu	ENSMUSG00000038383	Transcript	ENSMUST00000165234	protein_coding	rs27310833
2:155283614-155283614	regulatory_region_variant	MODIFIER	-	-	RegulatoryFeature	ENSMUSR00000166044	promoter_flanking_region	rs27310833
2:155291037-155291037	intron_variant	MODIFIER	Pigu	ENSMUSG00000038383	Transcript	ENSMUST00000077626	protein_coding	rs47443090
2:155291037-155291037	intron_variant,non_coding_trans	MODIFIER	Pigu	ENSMUSG00000038383	Transcript	ENSMUST00000139854	processed_transcript	rs47443090
2:155291037-155291037	intron_variant	MODIFIER	Pigu	ENSMUSG00000038383	Transcript	ENSMUST00000165234	protein_coding	rs47443090
2:155643238-155643238	intron_variant	MODIFIER	Trpc4ap	ENSMUSG00000038324	Transcript	ENSMUST00000041059	protein_coding	rs27308652
2:155643238-155643238	intron_variant	MODIFIER	Trpc4ap	ENSMUSG00000038324	Transcript	ENSMUST00000103140	protein_coding	rs27308652

2:155643238-155643238	downstream_gene_variant	MODIFIER	Trpc4ap	ENSMUSG00000038324	Transcript	ENSMUST00000140370	retained_intron	rs27308652
2:155643238-155643238	upstream_gene_variant	MODIFIER	Trpc4ap	ENSMUSG00000038324	Transcript	ENSMUST00000146484	retained_intron	rs27308652
2:155643238-155643238	intron_variant,non_coding_trans	MODIFIER	Trpc4ap	ENSMUSG00000038324	Transcript	ENSMUST00000152548	retained_intron	rs27308652
2:156311032-156311032	upstream_gene_variant	MODIFIER	Cnbd2	ENSMUSG00000038085	Transcript	ENSMUST00000037096	protein_coding	rs27355691
2:156311032-156311032	downstream_gene_variant	MODIFIER	Phf20	ENSMUSG00000038116	Transcript	ENSMUST00000037401	protein_coding	rs27355691
2:156311032-156311032	upstream_gene_variant	MODIFIER	Cnbd2	ENSMUSG00000038085	Transcript	ENSMUST00000073942	protein_coding	rs27355691
2:156311032-156311032	downstream_gene_variant	MODIFIER	Scand1	ENSMUSG00000046229	Transcript	ENSMUST00000079125	protein_coding	rs27355691
2:156311032-156311032	upstream_gene_variant	MODIFIER	Cnbd2	ENSMUSG00000038085	Transcript	ENSMUST00000109580	protein_coding	rs27355691
2:156311032-156311032	upstream_gene_variant	MODIFIER	Cnbd2	ENSMUSG00000038085	Transcript	ENSMUST00000125395	processed_transcript	rs27355691
2:156311032-156311032	upstream_gene_variant	MODIFIER	Cnbd2	ENSMUSG00000038085	Transcript	ENSMUST00000133056	retained_intron	rs27355691
2:156311032-156311032	downstream_gene_variant	MODIFIER	Phf20	ENSMUSG00000038116	Transcript	ENSMUST00000152617	processed_transcript	rs27355691
2:156320378-156320378	intron_variant	MODIFIER	Cnbd2	ENSMUSG00000038085	Transcript	ENSMUST00000037096	protein_coding	rs234111515
2:156320378-156320378	intron_variant	MODIFIER	Cnbd2	ENSMUSG00000038085	Transcript	ENSMUST00000073942	protein_coding	rs234111515
2:156320378-156320378	upstream_gene_variant	MODIFIER	Gm22759	ENSMUSG00000076231	Transcript	ENSMUST00000102276	miRNA	rs234111515
2:156320378-156320378	intron_variant	MODIFIER	Cnbd2	ENSMUSG00000038085	Transcript	ENSMUST00000109580	protein_coding	rs234111515
2:156320378-156320378	upstream_gene_variant	MODIFIER	Gm14251	ENSMUSG00000081751	Transcript	ENSMUST00000122133	processed_pseudogene	rs234111515
2:156320378-156320378	intron_variant,non_coding_trans	MODIFIER	Cnbd2	ENSMUSG00000038085	Transcript	ENSMUST00000125395	processed_transcript	rs234111515
2:156320378-156320378	non_coding_transcript_exon_va	MODIFIER	Cnbd2	ENSMUSG00000038085	Transcript	ENSMUST00000133056	retained_intron	rs234111515
2:156320426-156320426	intron_variant	MODIFIER	Cnbd2	ENSMUSG00000038085	Transcript	ENSMUST00000037096	protein_coding	rs245864004
2:156320426-156320426	intron_variant	MODIFIER	Cnbd2	ENSMUSG00000038085	Transcript	ENSMUST00000073942	protein_coding	rs245864004
2:156320426-156320426	upstream_gene_variant	MODIFIER	Gm22759	ENSMUSG00000076231	Transcript	ENSMUST00000102276	miRNA	rs245864004
2:156320426-156320426	intron_variant	MODIFIER	Cnbd2	ENSMUSG00000038085	Transcript	ENSMUST00000109580	protein_coding	rs245864004
2:156320426-156320426	upstream_gene_variant	MODIFIER	Gm14251	ENSMUSG00000081751	Transcript	ENSMUST00000122133	processed_pseudogene	rs245864004
2:156320426-156320426	intron_variant,non_coding_trans	MODIFIER	Cnbd2	ENSMUSG00000038085	Transcript	ENSMUST00000125395	processed_transcript	rs245864004
2:156320426-156320426	non_coding_transcript_exon_va	MODIFIER	Cnbd2	ENSMUSG00000038085	Transcript	ENSMUST00000133056	retained_intron	rs245864004
2:156321430-156321430	intron_variant	MODIFIER	Cnbd2	ENSMUSG00000038085	Transcript	ENSMUST00000037096	protein_coding	rs259295972
2:156321430-156321430	intron_variant	MODIFIER	Cnbd2	ENSMUSG00000038085	Transcript	ENSMUST00000073942	protein_coding	rs259295972
2:156321430-156321430	upstream_gene_variant	MODIFIER	Gm22759	ENSMUSG00000076231	Transcript	ENSMUST00000102276	miRNA	rs259295972
2:156321430-156321430	intron_variant	MODIFIER	Cnbd2	ENSMUSG00000038085	Transcript	ENSMUST00000109580	protein_coding	rs259295972
2:156321430-156321430	upstream_gene_variant	MODIFIER	Gm14251	ENSMUSG00000081751	Transcript	ENSMUST00000122133	processed_pseudogene	rs259295972
2:156321430-156321430	intron_variant,non_coding_trans	MODIFIER	Cnbd2	ENSMUSG00000038085	Transcript	ENSMUST00000125395	processed_transcript	rs259295972
2:156321430-156321430	downstream_gene_variant	MODIFIER	Cnbd2	ENSMUSG00000038085	Transcript	ENSMUST00000133056	retained_intron	rs259295972
2:156321713-156321713	intron_variant	MODIFIER	Cnbd2	ENSMUSG00000038085	Transcript	ENSMUST00000037096	protein_coding	rs224022414
2:156321713-156321713	intron_variant	MODIFIER	Cnbd2	ENSMUSG00000038085	Transcript	ENSMUST00000073942	protein_coding	rs224022414
2:156321713-156321713	upstream_gene_variant	MODIFIER	Gm22759	ENSMUSG00000076231	Transcript	ENSMUST00000102276	miRNA	rs224022414
2:156321713-156321713	intron_variant	MODIFIER	Cnbd2	ENSMUSG00000038085	Transcript	ENSMUST00000109580	protein_coding	rs224022414
2:156321713-156321713	upstream_gene_variant	MODIFIER	Gm14251	ENSMUSG00000081751	Transcript	ENSMUST00000122133	processed_pseudogene	rs224022414
2:156321713-156321713	intron_variant,non_coding_trans	MODIFIER	Cnbd2	ENSMUSG00000038085	Transcript	ENSMUST00000125395	processed_transcript	rs224022414
2:156321713-156321713	downstream_gene_variant	MODIFIER	Cnbd2	ENSMUSG00000038085	Transcript	ENSMUST00000133056	retained_intron	rs224022414
2:156342480-156342480	intron_variant	MODIFIER	Cnbd2	ENSMUSG00000038085	Transcript	ENSMUST00000037096	protein_coding	rs33205837
2:156342480-156342480	intron_variant	MODIFIER	Cnbd2	ENSMUSG00000038085	Transcript	ENSMUST00000073942	protein_coding	rs33205837
2:156342480-156342480	intron_variant	MODIFIER	Cnbd2	ENSMUSG00000038085	Transcript	ENSMUST00000109580	protein_coding	rs33205837
2:156342480-156342480	downstream_gene_variant	MODIFIER	Gm14253	ENSMUSG00000082321	Transcript	ENSMUST00000122112	processed_pseudogene	rs33205837
2:156342480-156342480	downstream_gene_variant	MODIFIER	Cnbd2	ENSMUSG00000038085	Transcript	ENSMUST00000125395	processed_transcript	rs33205837
2:156342480-156342480	upstream_gene_variant	MODIFIER	Gm14252	ENSMUSG00000085546	Transcript	ENSMUST00000131801	antisense	rs33205837
2:156342480-156342480	downstream_gene_variant	MODIFIER	Cnbd2	ENSMUSG00000038085	Transcript	ENSMUST00000154227	processed_transcript	rs33205837
2:156349504-156349504	intron_variant	MODIFIER	Cnbd2	ENSMUSG00000038085	Transcript	ENSMUST00000037096	protein_coding	rs27336925
2:156349504-156349504	intron_variant	MODIFIER	Cnbd2	ENSMUSG00000038085	Transcript	ENSMUST00000073942	protein_coding	rs27336925
2:156349504-156349504	intron_variant	MODIFIER	Cnbd2	ENSMUSG00000038085	Transcript	ENSMUST00000109580	protein_coding	rs27336925
2:156349504-156349504	upstream_gene_variant	MODIFIER	Gm14253	ENSMUSG00000082321	Transcript	ENSMUST00000122112	processed_pseudogene	rs27336925
2:156349758-156349758	intron_variant	MODIFIER	Cnbd2	ENSMUSG00000038085	Transcript	ENSMUST00000037096	protein_coding	rs27336923
2:156349758-156349758	intron_variant	MODIFIER	Cnbd2	ENSMUSG00000038085	Transcript	ENSMUST00000073942	protein_coding	rs27336923
2:156349758-156349758	intron_variant	MODIFIER	Cnbd2	ENSMUSG00000038085	Transcript	ENSMUST00000109580	protein_coding	rs27336923
2:156349758-156349758	upstream_gene_variant	MODIFIER	Gm14253	ENSMUSG00000082321	Transcript	ENSMUST00000122112	processed_pseudogene	rs27336923
2:156351013-156351013	intron_variant	MODIFIER	Cnbd2	ENSMUSG00000038085	Transcript	ENSMUST00000037096	protein_coding	rs29577843
2:156351013-156351013	intron_variant	MODIFIER	Cnbd2	ENSMUSG00000038085	Transcript	ENSMUST00000073942	protein_coding	rs29577843
2:156351013-156351013	intron_variant	MODIFIER	Cnbd2	ENSMUSG00000038085	Transcript	ENSMUST00000109580	protein_coding	rs29577843
2:156351013-156351013	upstream_gene_variant	MODIFIER	Gm14253	ENSMUSG00000082321	Transcript	ENSMUST00000122112	processed_pseudogene	rs29577843
2:156351018-156351018	intron_variant	MODIFIER	Cnbd2	ENSMUSG00000038085	Transcript	ENSMUST00000037096	protein_coding	rs33517821
2:156351018-156351018	intron_variant	MODIFIER	Cnbd2	ENSMUSG00000038085	Transcript	ENSMUST00000073942	protein_coding	rs33517821
2:156351018-156351018	intron_variant	MODIFIER	Cnbd2	ENSMUSG00000038085	Transcript	ENSMUST00000109580	protein_coding	rs33517821
2:156351018-156351018	upstream_gene_variant	MODIFIER	Gm14253	ENSMUSG00000082321	Transcript	ENSMUST00000122112	processed_pseudogene	rs33517821
2:156525188-156525188	missense_variant	MODERATE	Epb41l1	ENSMUSG00000027624	Transcript	ENSMUST00000029155	protein_coding	rs27306095
2:156525188-156525188	missense_variant	MODERATE	Epb41l1	ENSMUSG00000027624	Transcript	ENSMUST00000103136	protein_coding	rs27306095
2:156525188-156525188	missense_variant	MODERATE	Epb41l1	ENSMUSG00000027624	Transcript	ENSMUST00000103137	protein_coding	rs27306095
2:156525188-156525188	intron_variant	MODIFIER	Epb41l1	ENSMUSG00000027624	Transcript	ENSMUST00000109574	protein_coding	rs27306095
2:156525188-156525188	missense_variant	MODERATE	Epb41l1	ENSMUSG00000027624	Transcript	ENSMUST00000109577	protein_coding	rs27306095
2:156525188-156525188	downstream_gene_variant	MODIFIER	Epb41l1	ENSMUSG00000027624	Transcript	ENSMUST00000125153	protein_coding	rs27306095
2:157993884-157993884	intron_variant	MODIFIER	Tti1	ENSMUSG00000027650	Transcript	ENSMUST00000029179	protein_coding	rs256796174

2:157993884-157993884	intron_variant	MODIFIER	Tti1	ENSMUSG00000027650	Transcript	ENSMUST00000109522	protein_coding	rs256796174
2:157993884-157993884	downstream_gene_variant	MODIFIER	Tti1	ENSMUSG00000027650	Transcript	ENSMUST00000126541	processed_transcript	rs256796174
2:158317769-158317769	intron_variant	MODIFIER	Lbp	ENSMUSG00000016024	Transcript	ENSMUST00000016168	protein_coding	rs46179339
2:158317769-158317769	intron_variant	MODIFIER	Lbp	ENSMUSG00000016024	Transcript	ENSMUST00000129811	protein_coding	rs46179339
2:158317769-158317769	non_coding_transcript_exon_var	MODIFIER	Lbp	ENSMUSG00000016024	Transcript	ENSMUST00000146600	retained_intron	rs46179339
2:158317769-158317769	intron_variant,non_coding_trans	MODIFIER	Lbp	ENSMUSG00000016024	Transcript	ENSMUST00000152541	processed_transcript	rs46179339
2:158317802-158317802	intron_variant	MODIFIER	Lbp	ENSMUSG00000016024	Transcript	ENSMUST00000016168	protein_coding	rs247533409
2:158317802-158317802	intron_variant	MODIFIER	Lbp	ENSMUSG00000016024	Transcript	ENSMUST00000129811	protein_coding	rs247533409
2:158317802-158317802	non_coding_transcript_exon_var	MODIFIER	Lbp	ENSMUSG00000016024	Transcript	ENSMUST00000146600	retained_intron	rs247533409
2:158317802-158317802	intron_variant,non_coding_trans	MODIFIER	Lbp	ENSMUSG00000016024	Transcript	ENSMUST00000152541	processed_transcript	rs247533409
2:158317960-158317960	intron_variant	MODIFIER	Lbp	ENSMUSG00000016024	Transcript	ENSMUST00000016168	protein_coding	rs259708555
2:158317960-158317960	intron_variant	MODIFIER	Lbp	ENSMUSG00000016024	Transcript	ENSMUST00000129811	protein_coding	rs259708555
2:158317960-158317960	non_coding_transcript_exon_var	MODIFIER	Lbp	ENSMUSG00000016024	Transcript	ENSMUST00000146600	retained_intron	rs259708555
2:158317960-158317960	intron_variant,non_coding_trans	MODIFIER	Lbp	ENSMUSG00000016024	Transcript	ENSMUST00000152541	processed_transcript	rs259708555
2:158318736-158318736	intron_variant	MODIFIER	Lbp	ENSMUSG00000016024	Transcript	ENSMUST00000016168	protein_coding	rs27303850
2:158318736-158318736	intron_variant	MODIFIER	Lbp	ENSMUSG00000016024	Transcript	ENSMUST00000129811	protein_coding	rs27303850
2:158318736-158318736	downstream_gene_variant	MODIFIER	Lbp	ENSMUSG00000016024	Transcript	ENSMUST00000146600	retained_intron	rs27303850
2:158318736-158318736	intron_variant,non_coding_trans	MODIFIER	Lbp	ENSMUSG00000016024	Transcript	ENSMUST00000152541	processed_transcript	rs27303850
2:158319100-158319100	intron_variant	MODIFIER	Lbp	ENSMUSG00000016024	Transcript	ENSMUST00000016168	protein_coding	rs259485455
2:158319100-158319100	intron_variant	MODIFIER	Lbp	ENSMUSG00000016024	Transcript	ENSMUST00000129811	protein_coding	rs259485455
2:158319100-158319100	downstream_gene_variant	MODIFIER	Lbp	ENSMUSG00000016024	Transcript	ENSMUST00000146600	retained_intron	rs259485455
2:158319100-158319100	intron_variant,non_coding_trans	MODIFIER	Lbp	ENSMUSG00000016024	Transcript	ENSMUST00000152541	processed_transcript	rs259485455
2:158327893-158327893	intron_variant	MODIFIER	Lbp	ENSMUSG00000016024	Transcript	ENSMUST00000016168	protein_coding	rs27353956
2:158327893-158327893	downstream_gene_variant	MODIFIER	Lbp	ENSMUSG00000016024	Transcript	ENSMUST00000129811	protein_coding	rs27353956
2:158328097-158328097	intron_variant	MODIFIER	Lbp	ENSMUSG00000016024	Transcript	ENSMUST00000016168	protein_coding	rs27353953
2:158328097-158328097	downstream_gene_variant	MODIFIER	Lbp	ENSMUSG00000016024	Transcript	ENSMUST00000129811	protein_coding	rs27353953
2:158328617-158328617	intron_variant	MODIFIER	Lbp	ENSMUSG00000016024	Transcript	ENSMUST00000016168	protein_coding	rs27353950
2:158328617-158328617	downstream_gene_variant	MODIFIER	Lbp	ENSMUSG00000016024	Transcript	ENSMUST00000129811	protein_coding	rs27353950
2:158330319-158330319	intron_variant	MODIFIER	Lbp	ENSMUSG00000016024	Transcript	ENSMUST00000016168	protein_coding	rs27353941
2:158637611-158637611	intron_variant	MODIFIER	Actr5	ENSMUSG00000037761	Transcript	ENSMUST00000045644	protein_coding	rs51319236
2:158637611-158637611	intron_variant,non_coding_trans	MODIFIER	Actr5	ENSMUSG00000037761	Transcript	ENSMUST00000142531	retained_intron	rs51319236
2:158637611-158637611	upstream_gene_variant	MODIFIER	Mir3474	ENSMUSG00000093120	Transcript	ENSMUST00000175379	miRNA	rs51319236
2:158637611-158637611	downstream_gene_variant	MODIFIER	Actr5	ENSMUSG00000037761	Transcript	ENSMUST00000183586	processed_transcript	rs51319236
2:158637611-158637611	intron_variant,NMD_transcript_v	MODIFIER	Actr5	ENSMUSG00000037761	Transcript	ENSMUST00000183731	nonsense_mediated_decay	rs51319236
2:165784823-165784823	downstream_gene_variant	MODIFIER	Zmynd8	ENSMUSG00000039671	Transcript	ENSMUST00000018050	protein_coding	-
2:165784823-165784823	downstream_gene_variant	MODIFIER	Zmynd8	ENSMUSG00000039671	Transcript	ENSMUST00000088113	protein_coding	-
2:165784823-165784823	3_prime_UTR_variant	MODIFIER	Zmynd8	ENSMUSG00000039671	Transcript	ENSMUST00000099084	protein_coding	-
2:165784823-165784823	3_prime_UTR_variant	MODIFIER	Zmynd8	ENSMUSG00000039671	Transcript	ENSMUST00000109266	protein_coding	-
2:165784823-165784823	downstream_gene_variant	MODIFIER	Zmynd8	ENSMUSG00000039671	Transcript	ENSMUST00000109269	protein_coding	-
2:165784823-165784823	downstream_gene_variant	MODIFIER	Zmynd8	ENSMUSG00000039671	Transcript	ENSMUST00000152682	processed_transcript	-
2:165784823-165784823	3_prime_UTR_variant	MODIFIER	Zmynd8	ENSMUSG00000039671	Transcript	ENSMUST00000170272	protein_coding	-
2:165784823-165784823	3_prime_UTR_variant	MODIFIER	Zmynd8	ENSMUSG00000039671	Transcript	ENSMUST00000177633	protein_coding	-
2:165784823-165784823	regulatory_region_variant	MODIFIER	-	-	RegulatoryFeature	ENSMUSR00000638290	open_chromatin_region	-
2:166075605-166075605	intron_variant	MODIFIER	Sulf2	ENSMUSG00000006800	Transcript	ENSMUST00000088086	protein_coding	rs6362627
2:166075605-166075605	downstream_gene_variant	MODIFIER	Ncoa3	ENSMUSG00000027678	Transcript	ENSMUST00000088095	protein_coding	rs6362627
2:166075605-166075605	intron_variant	MODIFIER	Sulf2	ENSMUSG00000006800	Transcript	ENSMUST00000109249	protein_coding	rs6362627
2:166075605-166075605	downstream_gene_variant	MODIFIER	Ncoa3	ENSMUSG00000027678	Transcript	ENSMUST00000109252	protein_coding	rs6362627
2:166075605-166075605	intron_variant,non_coding_trans	MODIFIER	Sulf2	ENSMUSG00000006800	Transcript	ENSMUST00000125503	retained_intron	rs6362627
2:166075605-166075605	intron_variant,non_coding_trans	MODIFIER	Sulf2	ENSMUSG00000006800	Transcript	ENSMUST00000143996	retained_intron	rs6362627
2:166075605-166075605	intron_variant	MODIFIER	Sulf2	ENSMUSG00000006800	Transcript	ENSMUST00000146497	protein_coding	rs6362627
2:166076786-166076786	intron_variant	MODIFIER	Sulf2	ENSMUSG00000006800	Transcript	ENSMUST00000088086	protein_coding	rs29961095
2:166076786-166076786	downstream_gene_variant	MODIFIER	Ncoa3	ENSMUSG00000027678	Transcript	ENSMUST00000088095	protein_coding	rs29961095
2:166076786-166076786	intron_variant	MODIFIER	Sulf2	ENSMUSG00000006800	Transcript	ENSMUST00000109249	protein_coding	rs29961095
2:166076786-166076786	upstream_gene_variant	MODIFIER	Sulf2	ENSMUSG00000006800	Transcript	ENSMUST00000125503	retained_intron	rs29961095
2:166076786-166076786	intron_variant,non_coding_trans	MODIFIER	Sulf2	ENSMUSG00000006800	Transcript	ENSMUST00000143996	retained_intron	rs29961095
2:166076786-166076786	intron_variant	MODIFIER	Sulf2	ENSMUSG00000006800	Transcript	ENSMUST00000146497	protein_coding	rs29961095
2:168634687-168634687	3_prime_UTR_variant	MODIFIER	Atp9a	ENSMUSG00000027546	Transcript	ENSMUST00000029060	protein_coding	rs27288812
2:168634687-168634687	3_prime_UTR_variant	MODIFIER	Atp9a	ENSMUSG00000027546	Transcript	ENSMUST00000109175	protein_coding	rs27288812
2:168634687-168634687	3_prime_UTR_variant	MODIFIER	Atp9a	ENSMUSG00000027546	Transcript	ENSMUST00000109176	protein_coding	rs27288812
2:168634687-168634687	3_prime_UTR_variant	MODIFIER	Atp9a	ENSMUSG00000027546	Transcript	ENSMUST00000109177	protein_coding	rs27288812
2:168634687-168634687	3_prime_UTR_variant	MODIFIER	Atp9a	ENSMUSG00000027546	Transcript	ENSMUST00000178504	protein_coding	rs27288812
2:31615894-31615894	3_prime_UTR_variant	MODIFIER	Fubp3	ENSMUSG00000026843	Transcript	ENSMUST00000055244	protein_coding	rs864259738
2:31615894-31615894	intron_variant	MODIFIER	Fubp3	ENSMUSG00000026843	Transcript	ENSMUST00000113482	protein_coding	rs864259738
2:31615894-31615894	3_prime_UTR_variant	MODIFIER	Fubp3	ENSMUSG00000026843	Transcript	ENSMUST00000129903	protein_coding	rs864259738
2:31615894-31615894	intron_variant	MODIFIER	Fubp3	ENSMUSG00000026843	Transcript	ENSMUST00000130578	protein_coding	rs864259738
2:31615894-31615894	non_coding_transcript_exon_var	MODIFIER	Fubp3	ENSMUSG00000026843	Transcript	ENSMUST00000132534	retained_intron	rs864259738
2:31615894-31615894	downstream_gene_variant	MODIFIER	Fubp3	ENSMUSG00000026843	Transcript	ENSMUST00000134553	nonsense_mediated_decay	rs864259738
2:31615894-31615894	intron_variant	MODIFIER	Fubp3	ENSMUSG00000026843	Transcript	ENSMUST00000150473	protein_coding	rs864259738
2:31615894-31615894	downstream_gene_variant	MODIFIER	Mir6998	ENSMUSG00000098904	Transcript	ENSMUST00000183343	miRNA	rs864259738

2:3504036-3504036	intron_variant	MODIFIER	Hspa14	ENSMUSG00000109865	Transcript	ENSMUST00000027961	protein_coding	rs27110587
2:3504036-3504036	downstream_gene_variant	MODIFIER	Gm45902	ENSMUSG00000051396	Transcript	ENSMUST00000124331	protein_coding	rs27110587
2:3504036-3504036	upstream_gene_variant	MODIFIER	Hspa14	ENSMUSG00000109865	Transcript	ENSMUST00000135157	retained_intron	rs27110587
2:3504036-3504036	downstream_gene_variant	MODIFIER	Gm45902	ENSMUSG00000051396	Transcript	ENSMUST00000140494	protein_coding	rs27110587
2:81510802-81510802	intergenic_variant	MODIFIER	-	-	-	-	-	-
2:90739497-90739497	downstream_gene_variant	MODIFIER	Nup160	ENSMUSG00000051329	Transcript	ENSMUST00000057481	protein_coding	rs864286220
2:90739497-90739497	downstream_gene_variant	MODIFIER	Nup160	ENSMUSG00000051329	Transcript	ENSMUST00000126503	processed_transcript	rs864286220
3:10322192-10322192	intron_variant	MODIFIER	Impa1	ENSMUSG00000027531	Transcript	ENSMUST00000065938	protein_coding	rs108627359
3:10322192-10322192	intron_variant	MODIFIER	Impa1	ENSMUSG00000027531	Transcript	ENSMUST00000118410	protein_coding	rs108627359
3:10322192-10322192	downstream_gene_variant	MODIFIER	Impa1	ENSMUSG00000027531	Transcript	ENSMUST00000128912	protein_coding	rs108627359
3:10322192-10322192	intron_variant	MODIFIER	Impa1	ENSMUSG00000027531	Transcript	ENSMUST00000191670	protein_coding	rs108627359
3:10322192-10322192	intron_variant,NMD_transcript_v	MODIFIER	Gm38303	ENSMUSG00000103392	Transcript	ENSMUST00000192603	nonsense_mediated_decay	rs108627359
3:116676877-116676877	intron_variant	MODIFIER	Slc35a3	ENSMUSG00000027957	Transcript	ENSMUST00000029569	protein_coding	-
3:116676877-116676877	intron_variant	MODIFIER	Slc35a3	ENSMUSG00000027957	Transcript	ENSMUST00000120120	protein_coding	-
3:116676877-116676877	intron_variant	MODIFIER	Gm43191	ENSMUSG00000105103	Transcript	ENSMUST00000140672	protein_coding	-
3:116676877-116676877	downstream_gene_variant	MODIFIER	Slc35a3	ENSMUSG00000027957	Transcript	ENSMUST00000153108	protein_coding	-
3:116676877-116676877	upstream_gene_variant	MODIFIER	Slc35a3	ENSMUSG00000027957	Transcript	ENSMUST00000196331	processed_transcript	-
3:116676877-116676877	downstream_gene_variant	MODIFIER	Slc35a3	ENSMUSG00000027957	Transcript	ENSMUST00000196335	protein_coding	-
3:116744163-116744163	3_prime_UTR_variant	MODIFIER	Agl	ENSMUSG00000033400	Transcript	ENSMUST00000040603	protein_coding	-
3:116744163-116744163	3_prime_UTR_variant	MODIFIER	Agl	ENSMUSG00000033400	Transcript	ENSMUST00000160484	protein_coding	-
3:116744163-116744163	downstream_gene_variant	MODIFIER	Agl	ENSMUSG00000033400	Transcript	ENSMUST00000161336	nonsense_mediated_decay	-
3:116744163-116744163	3_prime_UTR_variant	MODIFIER	Agl	ENSMUSG00000033400	Transcript	ENSMUST00000162792	protein_coding	-
3:153931979-153931979	intron_variant	MODIFIER	Acadm	ENSMUSG00000062908	Transcript	ENSMUST00000072697	protein_coding	rs30604906
3:153931979-153931979	downstream_gene_variant	MODIFIER	Acadm	ENSMUSG00000062908	Transcript	ENSMUST00000150070	protein_coding	rs30604906
3:153931979-153931979	downstream_gene_variant	MODIFIER	Acadm	ENSMUSG00000062908	Transcript	ENSMUST00000156310	nonsense_mediated_decay	rs30604906
3:153931979-153931979	downstream_gene_variant	MODIFIER	Acadm	ENSMUSG00000062908	Transcript	ENSMUST00000196188	retained_intron	rs30604906
3:153931979-153931979	upstream_gene_variant	MODIFIER	Acadm	ENSMUSG00000062908	Transcript	ENSMUST00000196688	retained_intron	rs30604906
3:153931979-153931979	intron_variant,non_coding_trans	MODIFIER	Acadm	ENSMUSG00000062908	Transcript	ENSMUST00000199342	retained_intron	rs30604906
3:153931979-153931979	intron_variant,non_coding_trans	MODIFIER	Acadm	ENSMUSG00000062908	Transcript	ENSMUST00000200250	retained_intron	rs30604906
3:153932929-153932929	intron_variant	MODIFIER	Acadm	ENSMUSG00000062908	Transcript	ENSMUST00000072697	protein_coding	rs30453927
3:153932929-153932929	downstream_gene_variant	MODIFIER	Acadm	ENSMUSG00000062908	Transcript	ENSMUST00000150070	protein_coding	rs30453927
3:153932929-153932929	downstream_gene_variant	MODIFIER	Acadm	ENSMUSG00000062908	Transcript	ENSMUST00000156310	nonsense_mediated_decay	rs30453927
3:153932929-153932929	downstream_gene_variant	MODIFIER	Acadm	ENSMUSG00000062908	Transcript	ENSMUST00000196188	retained_intron	rs30453927
3:153932929-153932929	upstream_gene_variant	MODIFIER	Acadm	ENSMUSG00000062908	Transcript	ENSMUST00000196688	retained_intron	rs30453927
3:153932929-153932929	intron_variant,non_coding_trans	MODIFIER	Acadm	ENSMUSG00000062908	Transcript	ENSMUST00000199342	retained_intron	rs30453927
3:153932929-153932929	intron_variant,non_coding_trans	MODIFIER	Acadm	ENSMUSG00000062908	Transcript	ENSMUST00000200250	retained_intron	rs30453927
3:153932929-153932929	regulatory_region_variant	MODIFIER	-	-	RegulatoryFeature	ENSMUSR00000656346	open_chromatin_region	rs30453927
3:32336903-32336903	3_prime_UTR_variant	MODIFIER	Zmat3	ENSMUSG00000027663	Transcript	ENSMUST00000029199	protein_coding	rs29864994
3:32336903-32336903	3_prime_UTR_variant	MODIFIER	Zmat3	ENSMUSG00000027663	Transcript	ENSMUST00000168566	protein_coding	rs29864994
3:32339795-32339795	3_prime_UTR_variant	MODIFIER	Zmat3	ENSMUSG00000027663	Transcript	ENSMUST00000029199	protein_coding	rs29863219
3:32339795-32339795	3_prime_UTR_variant	MODIFIER	Zmat3	ENSMUSG00000027663	Transcript	ENSMUST00000168566	protein_coding	rs29863219
3:32340860-32340860	3_prime_UTR_variant	MODIFIER	Zmat3	ENSMUSG00000027663	Transcript	ENSMUST00000029199	protein_coding	rs30558420
3:32340860-32340860	3_prime_UTR_variant	MODIFIER	Zmat3	ENSMUSG00000027663	Transcript	ENSMUST00000168566	protein_coding	rs30558420
3:32340861-32340861	3_prime_UTR_variant	MODIFIER	Zmat3	ENSMUSG00000027663	Transcript	ENSMUST00000029199	protein_coding	rs30006568
3:32340861-32340861	3_prime_UTR_variant	MODIFIER	Zmat3	ENSMUSG00000027663	Transcript	ENSMUST00000168566	protein_coding	rs30006568
3:33801010-33801010	synonymous_variant	LOW	Ttc14	ENSMUSG00000027677	Transcript	ENSMUST00000099153	protein_coding	rs50656174
3:33801010-33801010	synonymous_variant	LOW	Ttc14	ENSMUSG00000027677	Transcript	ENSMUST00000108210	protein_coding	rs50656174
3:33801010-33801010	synonymous_variant	LOW	Ttc14	ENSMUSG00000027677	Transcript	ENSMUST00000117915	protein_coding	rs50656174
3:33801010-33801010	synonymous_variant	LOW	Ttc14	ENSMUSG00000027677	Transcript	ENSMUST00000196139	protein_coding	rs50656174
3:33801010-33801010	upstream_gene_variant	MODIFIER	Ttc14	ENSMUSG00000027677	Transcript	ENSMUST00000196369	nonsense_mediated_decay	rs50656174
3:33801010-33801010	synonymous_variant	LOW	Ttc14	ENSMUSG00000027677	Transcript	ENSMUST00000196975	protein_coding	rs50656174
3:33801010-33801010	upstream_gene_variant	MODIFIER	Ttc14	ENSMUSG00000027677	Transcript	ENSMUST00000197592	retained_intron	rs50656174
3:33801010-33801010	synonymous_variant	LOW	Ttc14	ENSMUSG00000027677	Transcript	ENSMUST00000198529	protein_coding	rs50656174
3:33801010-33801010	synonymous_variant,NMD_trans	LOW	Ttc14	ENSMUSG00000027677	Transcript	ENSMUST00000199222	nonsense_mediated_decay	rs50656174
3:33801010-33801010	downstream_gene_variant	MODIFIER	Ttc14	ENSMUSG00000027677	Transcript	ENSMUST00000199523	retained_intron	rs50656174
3:33801010-33801010	synonymous_variant	LOW	Ttc14	ENSMUSG00000027677	Transcript	ENSMUST00000200271	protein_coding	rs50656174
3:33801010-33801010	non_coding_transcript_exon_var	MODIFIER	Ttc14	ENSMUSG00000027677	Transcript	ENSMUST00000200559	retained_intron	rs50656174
3:33801010-33801010	regulatory_region_variant	MODIFIER	-	-	RegulatoryFeature	ENSMUSR00000172538	promoter	rs50656174
3:33804834-33804834	intron_variant	MODIFIER	Ttc14	ENSMUSG00000027677	Transcript	ENSMUST00000099153	protein_coding	rs30304375
3:33804834-33804834	intron_variant	MODIFIER	Ttc14	ENSMUSG00000027677	Transcript	ENSMUST00000108210	protein_coding	rs30304375
3:33804834-33804834	intron_variant	MODIFIER	Ttc14	ENSMUSG00000027677	Transcript	ENSMUST00000117915	protein_coding	rs30304375
3:33804834-33804834	downstream_gene_variant	MODIFIER	Ttc14	ENSMUSG00000027677	Transcript	ENSMUST00000196139	protein_coding	rs30304375
3:33804834-33804834	intron_variant,NMD_transcript_v	MODIFIER	Ttc14	ENSMUSG00000027677	Transcript	ENSMUST00000196369	nonsense_mediated_decay	rs30304375
3:33804834-33804834	intron_variant	MODIFIER	Ttc14	ENSMUSG00000027677	Transcript	ENSMUST00000196975	protein_coding	rs30304375
3:33804834-33804834	downstream_gene_variant	MODIFIER	Ttc14	ENSMUSG00000027677	Transcript	ENSMUST00000197592	retained_intron	rs30304375
3:33804834-33804834	intron_variant	MODIFIER	Ttc14	ENSMUSG00000027677	Transcript	ENSMUST00000198529	protein_coding	rs30304375
3:33804834-33804834	intron_variant,NMD_transcript_v	MODIFIER	Ttc14	ENSMUSG00000027677	Transcript	ENSMUST00000199222	nonsense_mediated_decay	rs30304375
3:33804834-33804834	downstream_gene_variant	MODIFIER	Ttc14	ENSMUSG00000027677	Transcript	ENSMUST00000199523	retained_intron	rs30304375
3:33804834-33804834	intron_variant	MODIFIER	Ttc14	ENSMUSG00000027677	Transcript	ENSMUST00000200271	protein_coding	rs30304375

3:33804834-33804834	downstream gene variant	MODIFIER	Ttc14	ENSMUSG00000027677	Transcript	ENSMUST00000200559	retained intron	rs30304375
3:65959007-65959007	upstream gene variant	MODIFIER	Ccnl1	ENSMUSG00000027829	Transcript	ENSMUST00000029416	protein coding	rs262950115
3:65959007-65959007	upstream_gene_variant	MODIFIER	Ccnl1	ENSMUSG00000027829	Transcript	ENSMUST00000122919	retained_intron	rs262950115
3:65959007-65959007	upstream_gene_variant	MODIFIER	Ccnl1	ENSMUSG00000027829	Transcript	ENSMUST00000129002	nonsense_mediated_decay	rs262950115
3:65959007-65959007	upstream gene variant	MODIFIER	Ccnl1	ENSMUSG00000027829	Transcript	ENSMUST00000135719	nonsense mediated decay	rs262950115
3:65959007-65959007	upstream gene variant	MODIFIER	Ccnl1	ENSMUSG00000027829	Transcript	ENSMUST00000142153	retained intron	rs262950115
3:65959007-65959007	upstream_gene_variant	MODIFIER	Ccnl1	ENSMUSG00000027829	Transcript	ENSMUST00000144810	nonsense_mediated_decay	rs262950115
3:65959007-65959007	upstream gene variant	MODIFIER	Ccnl1	ENSMUSG00000027829	Transcript	ENSMUST00000145186	nonsense mediated decay	rs262950115
3:65959007-65959007	upstream_gene_variant	MODIFIER	Ccnl1	ENSMUSG00000027829	Transcript	ENSMUST00000148623	retained intron	rs262950115
3:65959007-65959007	upstream_gene_variant	MODIFIER	Ccnl1	ENSMUSG00000027829	Transcript	ENSMUST00000149160	retained_intron	rs262950115
3:65959007-65959007	upstream gene variant	MODIFIER	Ccnl1	ENSMUSG00000027829	Transcript	ENSMUST00000154585	nonsense mediated decay	rs262950115
3:65959007-65959007	non coding transcript exon va	MODIFIER	Gm37305	ENSMUSG00000103041	Transcript	ENSMUST00000195714	antisense	rs262950115
3:65959007-65959007	regulatory_region_variant	MODIFIER	-	-	RegulatoryFeature	ENSMUSR00000175578	promoter	rs262950115
3:88411654-88411654	upstream gene variant	MODIFIER	Pmf1	ENSMUSG00000028066	Transcript	ENSMUST00000056370	protein coding	-
3:88411654-88411654	3 prime UTR variant	MODIFIER	Slc25a44	ENSMUSG00000050144	Transcript	ENSMUST00000057935	protein coding	-
3:88411654-88411654	3_prime_UTR_variant	MODIFIER	Slc25a44	ENSMUSG00000050144	Transcript	ENSMUST00000168755	protein coding	-
3:88411654-88411654	upstream_gene_variant	MODIFIER	Pmf1	ENSMUSG00000028066	Transcript	ENSMUST00000191622	retained_intron	-
3:88411654-88411654	upstream gene variant	MODIFIER	Pmf1	ENSMUSG00000028066	Transcript	ENSMUST00000192365	processed transcript	-
3:88411654-88411654	upstream gene variant	MODIFIER	Pmf1	ENSMUSG00000028066	Transcript	ENSMUST00000192909	nonsense mediated decay	-
3:88411654-88411654	upstream_gene_variant	MODIFIER	Pmf1	ENSMUSG00000028066	Transcript	ENSMUST00000193338	protein_coding	-
3:88411654-88411654	3 prime UTR variant	MODIFIER	Slc25a44	ENSMUSG00000050144	Transcript	ENSMUST00000193433	protein coding	-
3:88411654-88411654	3 prime UTR variant	MODIFIER	Slc25a44	ENSMUSG00000050144	Transcript	ENSMUST00000195657	protein coding	-
3:96624315-96624315	intron_variant,non_coding_trans	MODIFIER	6330549D23Rik	ENSMUSG00000045327	Transcript	ENSMUST00000059190	processed_transcript	rs864292968
3:96624315-96624315	3 prime UTR variant	MODIFIER	Lix1l	ENSMUSG00000049288	Transcript	ENSMUST00000062058	protein coding	rs864292968
3:96624315-96624315	intron variant,non coding trans	MODIFIER	6330549D23Rik	ENSMUSG00000045327	Transcript	ENSMUST00000160818	processed transcript	rs864292968
3:96624315-96624315	downstream_gene_variant	MODIFIER	Lix1l	ENSMUSG00000049288	Transcript	ENSMUST00000199166	retained_intron	rs864292968
4:107055235-107055235	downstream gene variant	MODIFIER	Mrpl37	ENSMUSG00000028622	Transcript	ENSMUST00000030365	protein coding	rs864257304
4:107055235-107055235	downstream gene variant	MODIFIER	Mrpl37	ENSMUSG00000028622	Transcript	ENSMUST00000125157	processed transcript	rs864257304
4:107055235-107055235	downstream_gene_variant	MODIFIER	Mrpl37	ENSMUSG00000028622	Transcript	ENSMUST00000125397	processed_transcript	rs864257304
4:107055235-107055235	downstream_gene_variant	MODIFIER	Mrpl37	ENSMUSG00000028622	Transcript	ENSMUST00000141150	retained_intron	rs864257304
4:140718569-140718569	intron variant	MODIFIER	Rcc2	ENSMUSG00000040945	Transcript	ENSMUST00000038893	protein coding	rs27611298
4:140718569-140718569	intron variant	MODIFIER	Rcc2	ENSMUSG00000040945	Transcript	ENSMUST00000071169	protein coding	rs27611298
4:140718569-140718569	downstream_gene_variant	MODIFIER	Rcc2	ENSMUSG00000040945	Transcript	ENSMUST00000129838	processed_transcript	rs27611298
4:140718569-140718569	downstream gene variant	MODIFIER	Rcc2	ENSMUSG00000040945	Transcript	ENSMUST00000138682	processed transcript	rs27611298
4:140718569-140718569	downstream gene variant	MODIFIER	Gm25951	ENSMUSG00000093071	Transcript	ENSMUST00000175330	miRNA	rs27611298
4:140918614-140918614	intron_variant	MODIFIER	Padi2	ENSMUSG00000028927	Transcript	ENSMUST00000030765	protein_coding	rs48530552
4:140918614-140918614	upstream gene variant	MODIFIER	Gm26226	ENSMUSG00000064443	Transcript	ENSMUST00000082509	snRNA	rs48530552
4:140918717-140918717	intron variant	MODIFIER	Padi2	ENSMUSG00000028927	Transcript	ENSMUST00000030765	protein coding	rs47649325
4:140918717-140918717	non_coding_transcript_exon_va	MODIFIER	Gm26226	ENSMUSG00000064443	Transcript	ENSMUST00000082509	snRNA	rs47649325
4:140918940-140918940	intron variant	MODIFIER	Padi2	ENSMUSG00000028927	Transcript	ENSMUST00000030765	protein coding	rs49698867
4:140918940-140918940	downstream gene variant	MODIFIER	Gm26226	ENSMUSG00000064443	Transcript	ENSMUST00000082509	snRNA	rs49698867
4:140919154-140919154	intron_variant	MODIFIER	Padi2	ENSMUSG00000028927	Transcript	ENSMUST00000030765	protein_coding	rs47519700
4:140919154-140919154	downstream_gene_variant	MODIFIER	Gm26226	ENSMUSG00000064443	Transcript	ENSMUST00000082509	snRNA	rs47519700
4:140931899-140931899	intron variant	MODIFIER	Padi2	ENSMUSG00000028927	Transcript	ENSMUST00000030765	protein coding	rs28276094
4:140931899-140931899	intron variant,non coding trans	MODIFIER	Padi2	ENSMUSG00000028927	Transcript	ENSMUST00000148160	processed transcript	rs28276094
4:140932914-140932914	intron_variant	MODIFIER	Padi2	ENSMUSG00000028927	Transcript	ENSMUST00000030765	protein_coding	rs47403939
4:140932914-140932914	downstream gene variant	MODIFIER	Padi2	ENSMUSG00000028927	Transcript	ENSMUST00000148160	processed transcript	rs47403939
4:140932955-140932955	intron variant	MODIFIER	Padi2	ENSMUSG00000028927	Transcript	ENSMUST00000030765	protein coding	rs47135910
4:140932955-140932955	downstream_gene_variant	MODIFIER	Padi2	ENSMUSG00000028927	Transcript	ENSMUST00000148160	processed_transcript	rs47135910
4:140974101-140974101	intron variant	MODIFIER	Sdhb	ENSMUSG00000009863	Transcript	ENSMUST00000010007	protein coding	rs260421764
4:140974101-140974101	non coding transcript exon va	MODIFIER	Sdhb	ENSMUSG00000009863	Transcript	ENSMUST00000125780	processed transcript	rs260421764
4:140974101-140974101	intron_variant,non_coding_trans	MODIFIER	Sdhb	ENSMUSG00000009863	Transcript	ENSMUST00000129181	processed_transcript	rs260421764
4:141002272-141002272	intron variant	MODIFIER	Atp13a2	ENSMUSG00000036622	Transcript	ENSMUST00000037055	protein coding	rs32601055
4:141002272-141002272	downstream gene variant	MODIFIER	Atp13a2	ENSMUSG00000036622	Transcript	ENSMUST00000125797	retained intron	rs32601055
4:141002272-141002272	intron_variant	MODIFIER	Atp13a2	ENSMUSG00000036622	Transcript	ENSMUST00000127833	protein_coding	rs32601055
4:141002272-141002272	downstream_gene_variant	MODIFIER	Atp13a2	ENSMUSG00000036622	Transcript	ENSMUST00000135117	processed_transcript	rs32601055
4:141002272-141002272	upstream gene variant	MODIFIER	Atp13a2	ENSMUSG00000036622	Transcript	ENSMUST00000137630	retained intron	rs32601055
4:141002272-141002272	intron variant,non coding trans	MODIFIER	Atp13a2	ENSMUSG00000036622	Transcript	ENSMUST00000151517	retained intron	rs32601055
4:141002272-141002272	downstream_gene_variant	MODIFIER	Atp13a2	ENSMUSG00000036622	Transcript	ENSMUST00000156995	processed_transcript	rs32601055
4:141002272-141002272	intron variant	MODIFIER	Atp13a2	ENSMUSG00000036622	Transcript	ENSMUST00000168047	protein coding	rs32601055
4:141002272-141002272	downstream gene variant	MODIFIER	Atp13a2	ENSMUSG00000036622	Transcript	ENSMUST00000170797	processed transcript	rs32601055
4:141220516-141220516	downstream_gene_variant	MODIFIER	Rsg1	ENSMUSG00000073733	Transcript	ENSMUST00000097813	protein_coding	rs258503513
4:141220516-141220516	downstream gene variant	MODIFIER	Rsg1	ENSMUSG00000073733	Transcript	ENSMUST00000125175	processed transcript	rs258503513
4:141220516-141220516	intron variant,non coding trans	MODIFIER	Rsg1	ENSMUSG00000073733	Transcript	ENSMUST00000151475	processed transcript	rs258503513
4:141458772-141458772	intron_variant	MODIFIER	Zbtb17	ENSMUSG00000006215	Transcript	ENSMUST00000006377	protein_coding	rs27604143
4:141458772-141458772	intron variant,non coding trans	MODIFIER	Zbtb17	ENSMUSG00000006215	Transcript	ENSMUST00000142020	processed transcript	rs27604143
4:141458772-141458772	intron variant,non coding trans	MODIFIER	Zbtb17	ENSMUSG00000006215	Transcript	ENSMUST00000142438	processed transcript	rs27604143
4:141458772-141458772	upstream_gene_variant	MODIFIER	Zbtb17	ENSMUSG00000006215	Transcript	ENSMUST00000142695	processed_transcript	rs27604143
4:141458772-141458772	intron_variant,non_coding_trans	MODIFIER	Zbtb17	ENSMUSG00000006215	Transcript	ENSMUST00000144899	processed_transcript	rs27604143

4:141458772-141458772	regulatory_region_variant	MODIFIER	-	-	RegulatoryFeature	ENSMUSR00000672503	promoter_flanking_region	rs27604143
4:141691929-141691929	intron_variant	MODIFIER	Ddi2	ENSMUSG00000078515	Transcript	ENSMUST00000102484	protein_coding	rs27628361
4:141691929-141691929	intron_variant	MODIFIER	Ddi2	ENSMUSG00000078515	Transcript	ENSMUST00000177592	protein_coding	rs27628361
4:141692756-141692756	intron_variant	MODIFIER	Ddi2	ENSMUSG00000078515	Transcript	ENSMUST00000102484	protein_coding	rs3723417
4:141692756-141692756	intron_variant	MODIFIER	Ddi2	ENSMUSG00000078515	Transcript	ENSMUST00000177592	protein_coding	rs3723417
4:141693240-141693240	intron_variant	MODIFIER	Ddi2	ENSMUSG00000078515	Transcript	ENSMUST00000102484	protein_coding	rs27628354
4:141693240-141693240	intron_variant	MODIFIER	Ddi2	ENSMUSG00000078515	Transcript	ENSMUST00000177592	protein_coding	rs27628354
4:141693282-141693282	intron_variant	MODIFIER	Ddi2	ENSMUSG00000078515	Transcript	ENSMUST00000102484	protein_coding	rs27628353
4:141693282-141693282	intron_variant	MODIFIER	Ddi2	ENSMUSG00000078515	Transcript	ENSMUST00000177592	protein_coding	rs27628353
4:141695254-141695254	intron_variant	MODIFIER	Ddi2	ENSMUSG00000078515	Transcript	ENSMUST00000102484	protein_coding	rs33025839
4:141695254-141695254	intron_variant	MODIFIER	Ddi2	ENSMUSG00000078515	Transcript	ENSMUST00000177592	protein_coding	rs33025839
4:141701984-141701985	intron_variant	MODIFIER	Ddi2	ENSMUSG00000078515	Transcript	ENSMUST00000102484	protein_coding	rs252056708
4:141701984-141701985	intron_variant	MODIFIER	Ddi2	ENSMUSG00000078515	Transcript	ENSMUST00000177592	protein_coding	rs252056708
4:141701989-141701989	intron_variant	MODIFIER	Ddi2	ENSMUSG00000078515	Transcript	ENSMUST00000102484	protein_coding	rs255958891
4:141701989-141701989	intron_variant	MODIFIER	Ddi2	ENSMUSG00000078515	Transcript	ENSMUST00000177592	protein_coding	rs255958891
4:141705382-141705382	intron_variant	MODIFIER	Ddi2	ENSMUSG00000078515	Transcript	ENSMUST00000102484	protein_coding	rs47015841
4:141705382-141705382	intron_variant	MODIFIER	Ddi2	ENSMUSG00000078515	Transcript	ENSMUST00000177592	protein_coding	rs47015841
4:141705382-141705382	regulatory_region_variant	MODIFIER	-	-	RegulatoryFeature	ENSMUSR00000672563	enhancer	rs47015841
4:144921113-144921113	intron_variant,NMD_transcript_v	MODIFIER	Dhrs3	ENSMUSG00000066026	Transcript	ENSMUST00000084184	nonsense_mediated_decay	rs50406955
4:144921113-144921113	intron_variant	MODIFIER	Dhrs3	ENSMUSG00000066026	Transcript	ENSMUST00000105744	protein_coding	rs50406955
4:144921113-144921113	downstream_gene_variant	MODIFIER	Dhrs3	ENSMUSG00000066026	Transcript	ENSMUST00000128926	processed_transcript	rs50406955
4:144921113-144921113	downstream_gene_variant	MODIFIER	Dhrs3	ENSMUSG00000066026	Transcript	ENSMUST00000133265	retained_intron	rs50406955
4:144921113-144921113	downstream_gene_variant	MODIFIER	Dhrs3	ENSMUSG00000066026	Transcript	ENSMUST00000142808	protein_coding	rs50406955
4:144921113-144921113	intron_variant	MODIFIER	Dhrs3	ENSMUSG00000066026	Transcript	ENSMUST00000154208	protein_coding	rs50406955
4:144921113-144921113	intron_variant	MODIFIER	Dhrs3	ENSMUSG00000066026	Transcript	ENSMUST00000171001	protein_coding	rs50406955
4:145224184-145224191	intron_variant	MODIFIER	Tnfrsf1b	ENSMUSG00000028599	Transcript	ENSMUST00000030336	protein_coding	-
4:145224184-145224191	downstream_gene_variant	MODIFIER	Tnfrsf1b	ENSMUSG00000028599	Transcript	ENSMUST00000143055	nonsense_mediated_decay	-
4:145224192-145224194	intron_variant	MODIFIER	Tnfrsf1b	ENSMUSG00000028599	Transcript	ENSMUST00000030336	protein_coding	-
4:145224192-145224194	downstream_gene_variant	MODIFIER	Tnfrsf1b	ENSMUSG00000028599	Transcript	ENSMUST00000143055	nonsense_mediated_decay	-
4:145292685-145292685	synonymous_variant	LOW	Tnfrsf8	ENSMUSG00000028602	Transcript	ENSMUST00000030339	protein_coding	rs27627532
4:145292685-145292685	synonymous_variant,NMD_trans	LOW	Tnfrsf8	ENSMUSG00000028602	Transcript	ENSMUST00000123027	nonsense_mediated_decay	rs27627532
4:145292746-145292746	missense_variant	MODERATE	Tnfrsf8	ENSMUSG00000028602	Transcript	ENSMUST00000030339	protein_coding	rs27627530
4:145292746-145292746	missense_variant,NMD_transcri	MODERATE	Tnfrsf8	ENSMUSG00000028602	Transcript	ENSMUST00000123027	nonsense_mediated_decay	rs27627530
4:145294635-145294635	intron_variant	MODIFIER	Tnfrsf8	ENSMUSG00000028602	Transcript	ENSMUST00000030339	protein_coding	rs27627519
4:145294635-145294635	intron_variant,NMD_transcript_v	MODIFIER	Tnfrsf8	ENSMUSG00000028602	Transcript	ENSMUST00000123027	nonsense_mediated_decay	rs27627519
4:146270623-146270623	intron_variant,non_coding_trans	MODIFIER	Gm13166	ENSMUSG00000085525	Transcript	ENSMUST00000124975	lincRNA	-
4:146270623-146270623	intron_variant,non_coding_trans	MODIFIER	Gm13166	ENSMUSG00000085525	Transcript	ENSMUST00000144166	lincRNA	-
4:146270623-146270623	intron_variant,non_coding_trans	MODIFIER	Gm13166	ENSMUSG00000085525	Transcript	ENSMUST00000146671	lincRNA	-
4:146270623-146270623	intron_variant,non_coding_trans	MODIFIER	Gm13166	ENSMUSG00000085525	Transcript	ENSMUST00000154592	lincRNA	-
4:146270623-146270623	intron_variant,non_coding_trans	MODIFIER	Gm26573	ENSMUSG00000096958	Transcript	ENSMUST00000181199	lincRNA	-
4:146270623-146270623	regulatory_region_variant	MODIFIER	-	-	RegulatoryFeature	ENSMUSR00000202689	promoter	-
4:146449070-146449070	5_prime_UTR_variant	MODIFIER	Zfp992	ENSMUSG00000070605	Transcript	ENSMUST00000105733	protein_coding	rs581062421
4:146449070-146449070	intron_variant,non_coding_trans	MODIFIER	Gm26573	ENSMUSG00000096958	Transcript	ENSMUST00000181199	lincRNA	rs581062421
4:146449070-146449070	regulatory_region_variant	MODIFIER	-	-	RegulatoryFeature	ENSMUSR00000673138	open_chromatin_region	rs581062421
4:146454614-146454614	intron_variant	MODIFIER	Zfp992	ENSMUSG00000070605	Transcript	ENSMUST00000105733	protein_coding	rs27631291
4:146454614-146454614	intron_variant,non_coding_trans	MODIFIER	Gm26573	ENSMUSG00000096958	Transcript	ENSMUST00000181199	lincRNA	rs27631291
4:146454621-146454621	intron_variant	MODIFIER	Zfp992	ENSMUSG00000070605	Transcript	ENSMUST00000105733	protein_coding	rs51742464
4:146454621-146454621	intron_variant,non_coding_trans	MODIFIER	Gm26573	ENSMUSG00000096958	Transcript	ENSMUST00000181199	lincRNA	rs51742464
4:146456889-146456891	intron_variant	MODIFIER	Zfp992	ENSMUSG00000070605	Transcript	ENSMUST00000105733	protein_coding	-
4:146456889-146456891	intron_variant,non_coding_trans	MODIFIER	Gm26573	ENSMUSG00000096958	Transcript	ENSMUST00000181199	lincRNA	-
4:146456894-146456894	intron_variant	MODIFIER	Zfp992	ENSMUSG00000070605	Transcript	ENSMUST00000105733	protein_coding	-
4:146456894-146456894	intron_variant,non_coding_trans	MODIFIER	Gm26573	ENSMUSG00000096958	Transcript	ENSMUST00000181199	lincRNA	-
4:146910781-146910781	upstream_gene_variant	MODIFIER	Gm21411	ENSMUSG00000045699	Transcript	ENSMUST00000049821	protein_coding	rs581273409
4:146910781-146910781	intron_variant,non_coding_trans	MODIFIER	Gm26573	ENSMUSG00000096958	Transcript	ENSMUST00000181199	lincRNA	rs581273409
4:146920723-146920723	intron_variant,non_coding_trans	MODIFIER	Gm26573	ENSMUSG00000096958	Transcript	ENSMUST00000181199	lincRNA	rs46653047
4:148016271-148016273	intron_variant	MODIFIER	Clcn6	ENSMUSG00000029016	Transcript	ENSMUST00000030879	protein_coding	rs223740081
4:148016271-148016273	intron_variant,NMD_transcript_v	MODIFIER	Clcn6	ENSMUSG00000029016	Transcript	ENSMUST00000105711	nonsense_mediated_decay	rs223740081
4:148016271-148016273	intron_variant	MODIFIER	Clcn6	ENSMUSG00000029016	Transcript	ENSMUST00000137724	protein_coding	rs223740081
4:148143664-148143664	downstream_gene_variant	MODIFIER	Fbxo6	ENSMUSG00000055401	Transcript	ENSMUST00000030858	protein_coding	rs31808429
4:148143664-148143664	intron_variant	MODIFIER	Mad2l2	ENSMUSG00000029003	Transcript	ENSMUST00000030860	protein_coding	rs31808429
4:148143664-148143664	downstream_gene_variant	MODIFIER	Fbxo6	ENSMUSG00000055401	Transcript	ENSMUST00000056965	protein_coding	rs31808429
4:148143664-148143664	intron_variant	MODIFIER	Mad2l2	ENSMUSG00000029003	Transcript	ENSMUST00000084129	protein_coding	rs31808429
4:148143664-148143664	downstream_gene_variant	MODIFIER	Fbxo6	ENSMUSG00000055401	Transcript	ENSMUST00000105706	protein_coding	rs31808429
4:148143664-148143664	intron_variant	MODIFIER	Mad2l2	ENSMUSG00000029003	Transcript	ENSMUST00000105707	protein_coding	rs31808429
4:148143664-148143664	upstream_gene_variant	MODIFIER	Mad2l2	ENSMUSG00000029003	Transcript	ENSMUST00000105708	retained_intron	rs31808429
4:148143664-148143664	downstream_gene_variant	MODIFIER	Mad2l2	ENSMUSG00000029003	Transcript	ENSMUST00000126261	processed_transcript	rs31808429
4:148143664-148143664	downstream_gene_variant	MODIFIER	Fbxo6	ENSMUSG00000055401	Transcript	ENSMUST00000126615	protein_coding	rs31808429
4:148143664-148143664	intron_variant	MODIFIER	Mad2l2	ENSMUSG00000029003	Transcript	ENSMUST00000132698	protein_coding	rs31808429
4:148143664-148143664	downstream_gene_variant	MODIFIER	Fbxo6	ENSMUSG00000055401	Transcript	ENSMUST00000134261	protein_coding	rs31808429

4:148143664-148143664	intron_variant	MODIFIER	Mad2l2	ENSMUSG00000029003	Transcript	ENSMUST00000140049	protein_coding	rs31808429
4:148143664-148143664	downstream_gene_variant	MODIFIER	Mad2l2	ENSMUSG00000029003	Transcript	ENSMUST00000148742	retained_intron	rs31808429
4:148143664-148143664	downstream_gene_variant	MODIFIER	Fbxo6	ENSMUSG00000055401	Transcript	ENSMUST00000152098	protein_coding	rs31808429
4:148143664-148143664	downstream_gene_variant	MODIFIER	Fbxo6	ENSMUSG00000055401	Transcript	ENSMUST00000168503	protein_coding	rs31808429
4:148143664-148143664	downstream_gene_variant	MODIFIER	Mir7022	ENSMUSG00000099043	Transcript	ENSMUST00000183749	miRNA	rs31808429
4:148144812-148144812	downstream_gene_variant	MODIFIER	Fbxo6	ENSMUSG00000055401	Transcript	ENSMUST00000030858	protein_coding	rs27602010
4:148144812-148144812	intron_variant	MODIFIER	Mad2l2	ENSMUSG00000029003	Transcript	ENSMUST00000030860	protein_coding	rs27602010
4:148144812-148144812	downstream_gene_variant	MODIFIER	Fbxo6	ENSMUSG00000055401	Transcript	ENSMUST00000056965	protein_coding	rs27602010
4:148144812-148144812	intron_variant	MODIFIER	Mad2l2	ENSMUSG00000029003	Transcript	ENSMUST00000084129	protein_coding	rs27602010
4:148144812-148144812	downstream_gene_variant	MODIFIER	Fbxo6	ENSMUSG00000055401	Transcript	ENSMUST00000105706	protein_coding	rs27602010
4:148144812-148144812	intron_variant	MODIFIER	Mad2l2	ENSMUSG00000029003	Transcript	ENSMUST00000105707	protein_coding	rs27602010
4:148144812-148144812	non_coding_transcript_exon_va	MODIFIER	Mad2l2	ENSMUSG00000029003	Transcript	ENSMUST00000105708	retained_intron	rs27602010
4:148144812-148144812	downstream_gene_variant	MODIFIER	Mad2l2	ENSMUSG00000029003	Transcript	ENSMUST00000126261	processed_transcript	rs27602010
4:148144812-148144812	downstream_gene_variant	MODIFIER	Fbxo6	ENSMUSG00000055401	Transcript	ENSMUST00000126615	protein_coding	rs27602010
4:148144812-148144812	downstream_gene_variant	MODIFIER	Fbxo6	ENSMUSG00000055401	Transcript	ENSMUST00000132083	protein_coding	rs27602010
4:148144812-148144812	intron_variant	MODIFIER	Mad2l2	ENSMUSG00000029003	Transcript	ENSMUST00000132698	protein_coding	rs27602010
4:148144812-148144812	downstream_gene_variant	MODIFIER	Fbxo6	ENSMUSG00000055401	Transcript	ENSMUST00000134261	protein_coding	rs27602010
4:148144812-148144812	intron_variant	MODIFIER	Mad2l2	ENSMUSG00000029003	Transcript	ENSMUST00000140049	protein_coding	rs27602010
4:148144812-148144812	downstream_gene_variant	MODIFIER	Mad2l2	ENSMUSG00000029003	Transcript	ENSMUST00000148742	retained_intron	rs27602010
4:148144812-148144812	downstream_gene_variant	MODIFIER	Fbxo6	ENSMUSG00000055401	Transcript	ENSMUST00000152098	protein_coding	rs27602010
4:148144812-148144812	downstream_gene_variant	MODIFIER	Fbxo6	ENSMUSG00000055401	Transcript	ENSMUST00000168503	protein_coding	rs27602010
4:148144812-148144812	downstream_gene_variant	MODIFIER	Mir7022	ENSMUSG00000099043	Transcript	ENSMUST00000183749	miRNA	rs27602010
4:148574509-148574509	intron_variant	MODIFIER	Exosc10	ENSMUSG00000017264	Transcript	ENSMUST00000017408	protein_coding	rs3167265
4:148574509-148574509	intron_variant	MODIFIER	Exosc10	ENSMUSG00000017264	Transcript	ENSMUST00000076022	protein_coding	rs3167265
4:148574509-148574509	intron_variant,NMD_transcript_v	MODIFIER	Exosc10	ENSMUSG00000017264	Transcript	ENSMUST00000097781	nonsense_mediated_decay	rs3167265
4:148574509-148574509	upstream_gene_variant	MODIFIER	Gm13204	ENSMUSG00000082860	Transcript	ENSMUST00000120110	processed_pseudogene	rs3167265
4:148574509-148574509	intron_variant,non_coding_trans	MODIFIER	Exosc10	ENSMUSG00000017264	Transcript	ENSMUST00000126897	retained_intron	rs3167265
4:148574509-148574509	intron_variant,non_coding_trans	MODIFIER	Exosc10	ENSMUSG00000017264	Transcript	ENSMUST00000173154	processed_transcript	rs3167265
4:148574509-148574509	intron_variant,non_coding_trans	MODIFIER	Exosc10	ENSMUSG00000017264	Transcript	ENSMUST00000173767	retained_intron	rs3167265
4:148574509-148574509	upstream_gene_variant	MODIFIER	Exosc10	ENSMUSG00000017264	Transcript	ENSMUST00000173892	processed_transcript	rs3167265
4:148595000-148595000	downstream_gene_variant	MODIFIER	Srm	ENSMUSG00000006442	Transcript	ENSMUST00000006611	protein_coding	rs3165450
4:148595000-148595000	downstream_gene_variant	MODIFIER	Srm	ENSMUSG00000006442	Transcript	ENSMUST00000140129	processed_transcript	rs3165450
4:148595000-148595000	downstream_gene_variant	MODIFIER	Srm	ENSMUSG00000006442	Transcript	ENSMUST00000152701	retained_intron	rs3165450
4:148595000-148595000	regulatory_region_variant	MODIFIER	-	-	RegulatoryFeature	ENSMUSR00000673487	open_chromatin_region	rs3165450
4:149653666-149653666	intron_variant	MODIFIER	Pik3cd	ENSMUSG00000039936	Transcript	ENSMUST00000038859	protein_coding	rs49908256
4:149653666-149653666	intron_variant	MODIFIER	Pik3cd	ENSMUSG00000039936	Transcript	ENSMUST00000105688	protein_coding	rs49908256
4:149653666-149653666	intron_variant	MODIFIER	Pik3cd	ENSMUSG00000039936	Transcript	ENSMUST00000105689	protein_coding	rs49908256
4:149653666-149653666	intron_variant	MODIFIER	Pik3cd	ENSMUSG00000039936	Transcript	ENSMUST00000105690	protein_coding	rs49908256
4:149653666-149653666	downstream_gene_variant	MODIFIER	Clstn1	ENSMUSG00000039953	Transcript	ENSMUST00000105691	protein_coding	rs49908256
4:149653666-149653666	intron_variant	MODIFIER	Pik3cd	ENSMUSG00000039936	Transcript	ENSMUST00000118704	protein_coding	rs49908256
4:149653666-149653666	intron_variant	MODIFIER	Pik3cd	ENSMUSG00000039936	Transcript	ENSMUST00000122059	protein_coding	rs49908256
4:149653666-149653666	intron_variant	MODIFIER	Pik3cd	ENSMUSG00000039936	Transcript	ENSMUST00000177654	protein_coding	rs49908256
4:149653666-149653666	downstream_gene_variant	MODIFIER	Mir7023	ENSMUSG00000098580	Transcript	ENSMUST00000185093	miRNA	rs49908256
4:149653666-149653666	regulatory_region_variant	MODIFIER	-	-	RegulatoryFeature	ENSMUSR00000673786	open_chromatin_region	rs49908256
4:149657626-149657626	intron_variant	MODIFIER	Pik3cd	ENSMUSG00000039936	Transcript	ENSMUST00000038859	protein_coding	rs578548868
4:149657626-149657626	intron_variant	MODIFIER	Pik3cd	ENSMUSG00000039936	Transcript	ENSMUST00000105688	protein_coding	rs578548868
4:149657626-149657626	intron_variant	MODIFIER	Pik3cd	ENSMUSG00000039936	Transcript	ENSMUST00000105689	protein_coding	rs578548868
4:149657626-149657626	intron_variant	MODIFIER	Pik3cd	ENSMUSG00000039936	Transcript	ENSMUST00000105690	protein_coding	rs578548868
4:149657626-149657626	intron_variant	MODIFIER	Pik3cd	ENSMUSG00000039936	Transcript	ENSMUST00000118704	protein_coding	rs578548868
4:149657626-149657626	intron_variant	MODIFIER	Pik3cd	ENSMUSG00000039936	Transcript	ENSMUST00000122059	protein_coding	rs578548868
4:149657626-149657626	downstream_gene_variant	MODIFIER	Pik3cd	ENSMUSG00000039936	Transcript	ENSMUST00000127273	protein_coding	rs578548868
4:149657626-149657626	downstream_gene_variant	MODIFIER	Pik3cd	ENSMUSG00000039936	Transcript	ENSMUST00000131224	protein_coding	rs578548868
4:149657626-149657626	downstream_gene_variant	MODIFIER	Pik3cd	ENSMUSG00000039936	Transcript	ENSMUST00000134534	protein_coding	rs578548868
4:149657626-149657626	downstream_gene_variant	MODIFIER	Pik3cd	ENSMUSG00000039936	Transcript	ENSMUST00000146612	protein_coding	rs578548868
4:149657626-149657626	intron_variant	MODIFIER	Pik3cd	ENSMUSG00000039936	Transcript	ENSMUST00000177654	protein_coding	rs578548868
4:149657626-149657626	upstream_gene_variant	MODIFIER	Mir7023	ENSMUSG00000098580	Transcript	ENSMUST00000185093	miRNA	rs578548868
4:150241780-150241780	upstream_gene_variant	MODIFIER	Eno1	ENSMUSG00000063524	Transcript	ENSMUST00000080149	protein_coding	rs32669891
4:150241780-150241780	intron_variant	MODIFIER	Eno1	ENSMUSG00000063524	Transcript	ENSMUST00000080926	protein_coding	rs32669891
4:150241780-150241780	non_coding_transcript_exon_va	MODIFIER	Eno1	ENSMUSG00000063524	Transcript	ENSMUST00000130632	retained_intron	rs32669891
4:150241780-150241780	upstream_gene_variant	MODIFIER	Eno1	ENSMUSG00000063524	Transcript	ENSMUST00000133789	retained_intron	rs32669891
4:150241780-150241780	synonymous_variant	LOW	Eno1	ENSMUSG00000063524	Transcript	ENSMUST00000133839	protein_coding	rs32669891
4:150241780-150241780	upstream_gene_variant	MODIFIER	Eno1	ENSMUSG00000063524	Transcript	ENSMUST00000135063	retained_intron	rs32669891
4:150241780-150241780	intron_variant	MODIFIER	Eno1	ENSMUSG00000063524	Transcript	ENSMUST00000141931	protein_coding	rs32669891
4:150241780-150241780	downstream_gene_variant	MODIFIER	Eno1	ENSMUSG00000063524	Transcript	ENSMUST00000148605	retained_intron	rs32669891
4:150241780-150241780	intron_variant	MODIFIER	Eno1	ENSMUSG00000063524	Transcript	ENSMUST00000150175	protein_coding	rs32669891
4:150902322-150902322	intron_variant	MODIFIER	Park7	ENSMUSG00000028964	Transcript	ENSMUST00000030805	protein_coding	rs32718258
4:150902322-150902322	intron_variant	MODIFIER	Park7	ENSMUSG00000028964	Transcript	ENSMUST00000105673	protein_coding	rs32718258
4:150902322-150902322	intron_variant	MODIFIER	Park7	ENSMUSG00000028964	Transcript	ENSMUST00000105674	protein_coding	rs32718258
4:150902322-150902322	intron_variant	MODIFIER	Park7	ENSMUSG00000028964	Transcript	ENSMUST00000105675	protein_coding	rs32718258

4:150902322-150902322	intron_variant	MODIFIER	Park7	ENSMUSG00000028964	Transcript	ENSMUST00000105676	protein_coding	rs32718258
4:150902322-150902322	intron_variant	MODIFIER	Park7	ENSMUSG00000028964	Transcript	ENSMUST00000128075	protein_coding	rs32718258
4:150902322-150902322	upstream_gene_variant	MODIFIER	Park7	ENSMUSG00000028964	Transcript	ENSMUST00000132265	processed_transcript	rs32718258
4:150902322-150902322	downstream_gene_variant	MODIFIER	Park7	ENSMUSG00000028964	Transcript	ENSMUST00000134751	protein_coding	rs32718258
4:150902322-150902322	downstream_gene_variant	MODIFIER	Park7	ENSMUSG00000028964	Transcript	ENSMUST00000146184	protein_coding	rs32718258
4:150902322-150902322	downstream_gene_variant	MODIFIER	Park7	ENSMUSG00000028964	Transcript	ENSMUST00000148626	retained_intron	rs32718258
4:150902322-150902322	regulatory_region_variant	MODIFIER	-	-	RegulatoryFeature	ENSMUSR00000419371	promoter_flanking_region	rs32718258
4:151055494-151055494	intron_variant	MODIFIER	Vamp3	ENSMUSG00000028955	Transcript	ENSMUST00000030797	protein_coding	rs32739749
4:151055494-151055494	downstream_gene_variant	MODIFIER	Camta1	ENSMUSG00000014592	Transcript	ENSMUST00000049790	protein_coding	rs32739749
4:151055494-151055494	downstream_gene_variant	MODIFIER	Camta1	ENSMUSG00000014592	Transcript	ENSMUST00000097774	protein_coding	rs32739749
4:151055494-151055494	downstream_gene_variant	MODIFIER	Camta1	ENSMUSG00000014592	Transcript	ENSMUST00000105668	protein_coding	rs32739749
4:151055494-151055494	downstream_gene_variant	MODIFIER	Camta1	ENSMUSG00000014592	Transcript	ENSMUST00000105670	protein_coding	rs32739749
4:151055494-151055494	downstream_gene_variant	MODIFIER	Vamp3	ENSMUSG00000028955	Transcript	ENSMUST00000125205	retained_intron	rs32739749
4:151055494-151055494	downstream_gene_variant	MODIFIER	Camta1	ENSMUSG00000014592	Transcript	ENSMUST00000140030	protein_coding	rs32739749
4:151055494-151055494	intron_variant,non_coding_trans	MODIFIER	Vamp3	ENSMUSG00000028955	Transcript	ENSMUST00000155446	retained_intron	rs32739749
4:151055494-151055494	intron_variant	MODIFIER	Camta1	ENSMUSG00000014592	Transcript	ENSMUST00000169423	protein_coding	rs32739749
4:151055494-151055494	regulatory_region_variant	MODIFIER	-	-	RegulatoryFeature	ENSMUSR00000674161	enhancer	rs32739749
4:151985433-151985433	intron_variant	MODIFIER	Thap3	ENSMUSG00000039759	Transcript	ENSMUST00000036680	protein_coding	rs227156016
4:151985433-151985433	downstream_gene_variant	MODIFIER	Phf13	ENSMUSG00000047777	Transcript	ENSMUST00000055688	protein_coding	rs227156016
4:151985433-151985433	downstream_gene_variant	MODIFIER	Dnajc11	ENSMUSG00000039768	Transcript	ENSMUST00000062904	protein_coding	rs227156016
4:151985433-151985433	intron_variant	MODIFIER	Thap3	ENSMUSG00000039759	Transcript	ENSMUST00000105665	protein_coding	rs227156016
4:151985433-151985433	downstream_gene_variant	MODIFIER	Dnajc11	ENSMUSG00000039768	Transcript	ENSMUST00000124955	retained_intron	rs227156016
4:151985433-151985433	downstream_gene_variant	MODIFIER	Dnajc11	ENSMUSG00000039768	Transcript	ENSMUST00000128582	retained_intron	rs227156016
4:151985433-151985433	downstream_gene_variant	MODIFIER	Dnajc11	ENSMUSG00000039768	Transcript	ENSMUST00000139069	nonsense_mediated_decay	rs227156016
4:151985433-151985433	downstream_gene_variant	MODIFIER	Thap3	ENSMUSG00000039759	Transcript	ENSMUST00000146471	retained_intron	rs227156016
4:151985433-151985433	downstream_gene_variant	MODIFIER	Dnajc11	ENSMUSG00000039768	Transcript	ENSMUST00000149337	retained_intron	rs227156016
4:151987335-151987335	intron_variant	MODIFIER	Thap3	ENSMUSG00000039759	Transcript	ENSMUST00000036680	protein_coding	rs228896315
4:151987335-151987335	downstream_gene_variant	MODIFIER	Phf13	ENSMUSG00000047777	Transcript	ENSMUST00000055688	protein_coding	rs228896315
4:151987335-151987335	intron_variant	MODIFIER	Thap3	ENSMUSG00000039759	Transcript	ENSMUST00000105665	protein_coding	rs228896315
4:151987335-151987335	intron_variant,non_coding_trans	MODIFIER	Thap3	ENSMUSG00000039759	Transcript	ENSMUST00000146471	retained_intron	rs228896315
4:152023620-152023620	downstream_gene_variant	MODIFIER	Tas1r1	ENSMUSG00000028950	Transcript	ENSMUST00000030792	protein_coding	rs47862982
4:152023620-152023620	intron_variant	MODIFIER	Zbtb48	ENSMUSG00000028952	Transcript	ENSMUST00000066715	protein_coding	rs47862982
4:152023620-152023620	intron_variant,non_coding_trans	MODIFIER	Zbtb48	ENSMUSG00000028952	Transcript	ENSMUST00000123696	retained_intron	rs47862982
4:152023620-152023620	downstream_gene_variant	MODIFIER	Zbtb48	ENSMUSG00000028952	Transcript	ENSMUST00000131935	protein_coding	rs47862982
4:152023620-152023620	upstream_gene_variant	MODIFIER	Zbtb48	ENSMUSG00000028952	Transcript	ENSMUST00000136212	processed_transcript	rs47862982
4:152023620-152023620	upstream_gene_variant	MODIFIER	Zbtb48	ENSMUSG00000028952	Transcript	ENSMUST00000147895	processed_transcript	rs47862982
4:152023620-152023620	intron_variant,NMD_transcript_v	MODIFIER	Zbtb48	ENSMUSG00000028952	Transcript	ENSMUST00000155389	nonsense_mediated_decay	rs47862982
4:152023620-152023620	intron_variant,non_coding_trans	MODIFIER	Zbtb48	ENSMUSG00000028952	Transcript	ENSMUST00000155441	retained_intron	rs47862982
4:152023620-152023620	intron_variant,NMD_transcript_v	MODIFIER	Zbtb48	ENSMUSG00000028952	Transcript	ENSMUST00000156748	nonsense_mediated_decay	rs47862982
4:152032080-152032080	synonymous_variant	LOW	Tas1r1	ENSMUSG00000028950	Transcript	ENSMUST00000030792	protein_coding	rs31834980
4:152032080-152032080	upstream_gene_variant	MODIFIER	Zbtb48	ENSMUSG00000028952	Transcript	ENSMUST00000066715	protein_coding	rs31834980
4:152032080-152032080	upstream_gene_variant	MODIFIER	Zbtb48	ENSMUSG00000028952	Transcript	ENSMUST00000123696	retained_intron	rs31834980
4:152032080-152032080	upstream_gene_variant	MODIFIER	Zbtb48	ENSMUSG00000028952	Transcript	ENSMUST00000131935	protein_coding	rs31834980
4:152032080-152032080	upstream_gene_variant	MODIFIER	Zbtb48	ENSMUSG00000028952	Transcript	ENSMUST00000155389	nonsense_mediated_decay	rs31834980
4:152112641-152112641	upstream_gene_variant	MODIFIER	Tnfrsf25	ENSMUSG00000024793	Transcript	ENSMUST00000025706	protein_coding	rs32693222
4:152112641-152112641	upstream_gene_variant	MODIFIER	Tnfrsf25	ENSMUSG00000024793	Transcript	ENSMUST00000035275	protein_coding	rs32693222
4:152112641-152112641	synonymous_variant	LOW	Plekhg5	ENSMUSG00000039713	Transcript	ENSMUST00000084115	protein_coding	rs32693222
4:152112641-152112641	synonymous_variant	LOW	Plekhg5	ENSMUSG00000039713	Transcript	ENSMUST00000105661	protein_coding	rs32693222
4:152112641-152112641	synonymous_variant	LOW	Plekhg5	ENSMUSG00000039713	Transcript	ENSMUST00000105662	protein_coding	rs32693222
4:152112641-152112641	synonymous_variant	LOW	Plekhg5	ENSMUSG00000039713	Transcript	ENSMUST00000118648	protein_coding	rs32693222
4:152112641-152112641	upstream_gene_variant	MODIFIER	Tnfrsf25	ENSMUSG00000024793	Transcript	ENSMUST00000127111	processed_transcript	rs32693222
4:152112641-152112641	non_coding_transcript_exon_var	MODIFIER	Plekhg5	ENSMUSG00000039713	Transcript	ENSMUST00000140085	retained_intron	rs32693222
4:152112641-152112641	downstream_gene_variant	MODIFIER	Plekhg5	ENSMUSG00000039713	Transcript	ENSMUST00000142412	retained_intron	rs32693222
4:152270058-152270059	intron_variant	MODIFIER	Acot7	ENSMUSG00000028937	Transcript	ENSMUST00000030779	protein_coding	rs214782755
4:152270058-152270059	intron_variant	MODIFIER	Acot7	ENSMUSG00000028937	Transcript	ENSMUST00000075363	protein_coding	rs214782755
4:152270058-152270059	upstream_gene_variant	MODIFIER	Gpr153	ENSMUSG00000042804	Transcript	ENSMUST00000105650	protein_coding	rs214782755
4:152270058-152270059	upstream_gene_variant	MODIFIER	Gpr153	ENSMUSG00000042804	Transcript	ENSMUST00000105651	protein_coding	rs214782755
4:152270058-152270059	intron_variant	MODIFIER	Acot7	ENSMUSG00000028937	Transcript	ENSMUST00000105652	protein_coding	rs214782755
4:152270058-152270059	intron_variant	MODIFIER	Acot7	ENSMUSG00000028937	Transcript	ENSMUST00000167926	protein_coding	rs214782755
4:152270095-152270095	intron_variant	MODIFIER	Acot7	ENSMUSG00000028937	Transcript	ENSMUST00000030779	protein_coding	rs32261663
4:152270095-152270095	intron_variant	MODIFIER	Acot7	ENSMUSG00000028937	Transcript	ENSMUST00000075363	protein_coding	rs32261663
4:152270095-152270095	upstream_gene_variant	MODIFIER	Gpr153	ENSMUSG00000042804	Transcript	ENSMUST00000105650	protein_coding	rs32261663
4:152270095-152270095	upstream_gene_variant	MODIFIER	Gpr153	ENSMUSG00000042804	Transcript	ENSMUST00000105651	protein_coding	rs32261663
4:152270095-152270095	intron_variant	MODIFIER	Acot7	ENSMUSG00000028937	Transcript	ENSMUST00000105652	protein_coding	rs32261663
4:152270095-152270095	intron_variant	MODIFIER	Acot7	ENSMUSG00000028937	Transcript	ENSMUST00000167926	protein_coding	rs32261663
4:152270109-152270109	intron_variant	MODIFIER	Acot7	ENSMUSG00000028937	Transcript	ENSMUST00000030779	protein_coding	rs32146192
4:152270109-152270109	intron_variant	MODIFIER	Acot7	ENSMUSG00000028937	Transcript	ENSMUST00000075363	protein_coding	rs32146192
4:152270109-152270109	upstream_gene_variant	MODIFIER	Gpr153	ENSMUSG00000042804	Transcript	ENSMUST00000105650	protein_coding	rs32146192
4:152270109-152270109	upstream_gene_variant	MODIFIER	Gpr153	ENSMUSG00000042804	Transcript	ENSMUST00000105651	protein_coding	rs32146192

4:152270109-152270109	intron_variant	MODIFIER	Acot7	ENSMUSG00000028937	Transcript	ENSMUST00000105652	protein_coding	rs32146192
4:152270109-152270109	intron_variant	MODIFIER	Acot7	ENSMUSG00000028937	Transcript	ENSMUST00000167926	protein_coding	rs32146192
4:152326124-152326124	intron_variant	MODIFIER	Rpl22	ENSMUSG00000028936	Transcript	ENSMUST00000103191	protein_coding	rs48946340
4:152326124-152326124	upstream_gene_variant	MODIFIER	Rpl22	ENSMUSG00000028936	Transcript	ENSMUST00000126519	processed_transcript	rs48946340
4:152326124-152326124	upstream_gene_variant	MODIFIER	Rpl22	ENSMUSG00000028936	Transcript	ENSMUST00000127659	processed_transcript	rs48946340
4:152326124-152326124	intron_variant	MODIFIER	Rpl22	ENSMUSG00000028936	Transcript	ENSMUST00000139685	protein_coding	rs48946340
4:152326124-152326124	intron_variant,non_coding_trans	MODIFIER	Rpl22	ENSMUSG00000028936	Transcript	ENSMUST00000142735	processed_transcript	rs48946340
4:152326124-152326124	upstream_gene_variant	MODIFIER	Rpl22	ENSMUSG00000028936	Transcript	ENSMUST00000150485	processed_transcript	rs48946340
4:152326124-152326124	upstream_gene_variant	MODIFIER	Rpl22	ENSMUSG00000028936	Transcript	ENSMUST00000156445	processed_transcript	rs48946340
4:152326124-152326124	intron_variant	MODIFIER	Rpl22	ENSMUSG00000028936	Transcript	ENSMUST00000188151	protein_coding	rs48946340
4:152326124-152326124	regulatory_region_variant	MODIFIER	-	-	RegulatoryFeature	ENSMUSR00000419573	promoter	rs48946340
4:152398700-152398700	intron_variant	MODIFIER	Kcnab2	ENSMUSG00000028931	Transcript	ENSMUST00000030768	protein_coding	rs32687531
4:152398700-152398700	intron_variant	MODIFIER	Kcnab2	ENSMUSG00000028931	Transcript	ENSMUST00000105648	protein_coding	rs32687531
4:152398700-152398700	intron_variant	MODIFIER	Kcnab2	ENSMUSG00000028931	Transcript	ENSMUST00000159186	protein_coding	rs32687531
4:152398700-152398700	downstream_gene_variant	MODIFIER	Kcnab2	ENSMUSG00000028931	Transcript	ENSMUST00000159435	processed_transcript	rs32687531
4:152398700-152398700	intron_variant	MODIFIER	Kcnab2	ENSMUSG00000028931	Transcript	ENSMUST00000159840	protein_coding	rs32687531
4:152398700-152398700	intron_variant,non_coding_trans	MODIFIER	Kcnab2	ENSMUSG00000028931	Transcript	ENSMUST00000159844	processed_transcript	rs32687531
4:152398700-152398700	intron_variant	MODIFIER	Kcnab2	ENSMUSG00000028931	Transcript	ENSMUST00000160884	protein_coding	rs32687531
4:152398700-152398700	downstream_gene_variant	MODIFIER	Kcnab2	ENSMUSG00000028931	Transcript	ENSMUST00000161236	protein_coding	rs32687531
4:152398700-152398700	downstream_gene_variant	MODIFIER	Kcnab2	ENSMUSG00000028931	Transcript	ENSMUST00000161496	processed_transcript	rs32687531
4:152398700-152398700	regulatory_region_variant	MODIFIER	-	-	RegulatoryFeature	ENSMUSR00000674524	CTCF_binding_site	rs32687531
4:152399928-152399928	intron_variant	MODIFIER	Kcnab2	ENSMUSG00000028931	Transcript	ENSMUST00000030768	protein_coding	rs32529839
4:152399928-152399928	intron_variant	MODIFIER	Kcnab2	ENSMUSG00000028931	Transcript	ENSMUST00000105648	protein_coding	rs32529839
4:152399928-152399928	intron_variant	MODIFIER	Kcnab2	ENSMUSG00000028931	Transcript	ENSMUST00000159186	protein_coding	rs32529839
4:152399928-152399928	downstream_gene_variant	MODIFIER	Kcnab2	ENSMUSG00000028931	Transcript	ENSMUST00000159435	processed_transcript	rs32529839
4:152399928-152399928	intron_variant	MODIFIER	Kcnab2	ENSMUSG00000028931	Transcript	ENSMUST00000159840	protein_coding	rs32529839
4:152399928-152399928	intron_variant,non_coding_trans	MODIFIER	Kcnab2	ENSMUSG00000028931	Transcript	ENSMUST00000159844	processed_transcript	rs32529839
4:152399928-152399928	intron_variant	MODIFIER	Kcnab2	ENSMUSG00000028931	Transcript	ENSMUST00000160884	protein_coding	rs32529839
4:152399928-152399928	downstream_gene_variant	MODIFIER	Kcnab2	ENSMUSG00000028931	Transcript	ENSMUST00000161236	protein_coding	rs32529839
4:152399928-152399928	downstream_gene_variant	MODIFIER	Kcnab2	ENSMUSG00000028931	Transcript	ENSMUST00000161496	processed_transcript	rs32529839
4:152399958-152399958	intron_variant	MODIFIER	Kcnab2	ENSMUSG00000028931	Transcript	ENSMUST00000030768	protein_coding	rs32078249
4:152399958-152399958	intron_variant	MODIFIER	Kcnab2	ENSMUSG00000028931	Transcript	ENSMUST00000105648	protein_coding	rs32078249
4:152399958-152399958	intron_variant	MODIFIER	Kcnab2	ENSMUSG00000028931	Transcript	ENSMUST00000159186	protein_coding	rs32078249
4:152399958-152399958	downstream_gene_variant	MODIFIER	Kcnab2	ENSMUSG00000028931	Transcript	ENSMUST00000159435	processed_transcript	rs32078249
4:152399958-152399958	intron_variant	MODIFIER	Kcnab2	ENSMUSG00000028931	Transcript	ENSMUST00000159840	protein_coding	rs32078249
4:152399958-152399958	intron_variant,non_coding_trans	MODIFIER	Kcnab2	ENSMUSG00000028931	Transcript	ENSMUST00000159844	processed_transcript	rs32078249
4:152399958-152399958	intron_variant	MODIFIER	Kcnab2	ENSMUSG00000028931	Transcript	ENSMUST00000160884	protein_coding	rs32078249
4:152399958-152399958	downstream_gene_variant	MODIFIER	Kcnab2	ENSMUSG00000028931	Transcript	ENSMUST00000161236	protein_coding	rs32078249
4:152399958-152399958	downstream_gene_variant	MODIFIER	Kcnab2	ENSMUSG00000028931	Transcript	ENSMUST00000161496	processed_transcript	rs32078249
4:152406228-152406228	intron_variant	MODIFIER	Kcnab2	ENSMUSG00000028931	Transcript	ENSMUST00000030768	protein_coding	rs48120185
4:152406228-152406228	intron_variant	MODIFIER	Kcnab2	ENSMUSG00000028931	Transcript	ENSMUST00000105648	protein_coding	rs48120185
4:152406228-152406228	intron_variant	MODIFIER	Kcnab2	ENSMUSG00000028931	Transcript	ENSMUST00000159186	protein_coding	rs48120185
4:152406228-152406228	intron_variant,non_coding_trans	MODIFIER	Kcnab2	ENSMUSG00000028931	Transcript	ENSMUST00000159435	processed_transcript	rs48120185
4:152406228-152406228	intron_variant	MODIFIER	Kcnab2	ENSMUSG00000028931	Transcript	ENSMUST00000159840	protein_coding	rs48120185
4:152406228-152406228	intron_variant,non_coding_trans	MODIFIER	Kcnab2	ENSMUSG00000028931	Transcript	ENSMUST00000159844	processed_transcript	rs48120185
4:152406228-152406228	intron_variant	MODIFIER	Kcnab2	ENSMUSG00000028931	Transcript	ENSMUST00000160884	protein_coding	rs48120185
4:152406228-152406228	intron_variant	MODIFIER	Kcnab2	ENSMUSG00000028931	Transcript	ENSMUST00000161236	protein_coding	rs48120185
4:152406228-152406228	upstream_gene_variant	MODIFIER	Gm16333	ENSMUSG00000090057	Transcript	ENSMUST00000161416	antisense	rs48120185
4:152406228-152406228	intron_variant,non_coding_trans	MODIFIER	Kcnab2	ENSMUSG00000028931	Transcript	ENSMUST00000161496	processed_transcript	rs48120185
4:152406228-152406228	regulatory_region_variant	MODIFIER	-	-	RegulatoryFeature	ENSMUSR00000674526	enhancer	rs48120185
4:154320539-154320539	3_prime_UTR_variant	MODIFIER	Prdm16	ENSMUSG00000039410	Transcript	ENSMUST00000030902	protein_coding	rs33057013
4:154320539-154320539	3_prime_UTR_variant	MODIFIER	Prdm16	ENSMUSG00000039410	Transcript	ENSMUST00000070313	protein_coding	rs33057013
4:154320539-154320539	3_prime_UTR_variant	MODIFIER	Prdm16	ENSMUSG00000039410	Transcript	ENSMUST00000097759	protein_coding	rs33057013
4:154320539-154320539	3_prime_UTR_variant	MODIFIER	Prdm16	ENSMUSG00000039410	Transcript	ENSMUST00000105636	protein_coding	rs33057013
4:154320539-154320539	3_prime_UTR_variant	MODIFIER	Prdm16	ENSMUSG00000039410	Transcript	ENSMUST00000105637	protein_coding	rs33057013
4:154320539-154320539	3_prime_UTR_variant	MODIFIER	Prdm16	ENSMUSG00000039410	Transcript	ENSMUST00000105638	protein_coding	rs33057013
4:154320539-154320539	non_coding_transcript_exon_va	MODIFIER	Prdm16	ENSMUSG00000039410	Transcript	ENSMUST00000141164	processed_transcript	rs33057013
4:154320539-154320539	downstream_gene_variant	MODIFIER	Prdm16	ENSMUSG00000039410	Transcript	ENSMUST00000145217	processed_transcript	rs33057013
4:154921951-154921951	downstream_gene_variant	MODIFIER	Tnfrsf14	ENSMUSG00000042333	Transcript	ENSMUST00000123514	protein_coding	-
4:154921951-154921951	downstream_gene_variant	MODIFIER	Tnfrsf14	ENSMUSG00000042333	Transcript	ENSMUST00000137803	protein_coding	-
4:154921951-154921951	downstream_gene_variant	MODIFIER	Tnfrsf14	ENSMUSG00000042333	Transcript	ENSMUST00000145296	protein_coding	-
4:154921951-154921951	downstream_gene_variant	MODIFIER	Tnfrsf14	ENSMUSG00000042333	Transcript	ENSMUST00000152687	protein_coding	-
4:154921951-154921951	3_prime_UTR_variant	MODIFIER	Tnfrsf14	ENSMUSG00000042333	Transcript	ENSMUST00000219534	protein_coding	-
4:154923854-154923854	intron_variant	MODIFIER	Tnfrsf14	ENSMUSG00000042333	Transcript	ENSMUST00000123514	protein_coding	rs249064527
4:154923854-154923854	downstream_gene_variant	MODIFIER	Tnfrsf14	ENSMUSG00000042333	Transcript	ENSMUST00000137803	protein_coding	rs249064527
4:154923854-154923854	downstream_gene_variant	MODIFIER	Tnfrsf14	ENSMUSG00000042333	Transcript	ENSMUST00000145296	protein_coding	rs249064527
4:154923854-154923854	intron_variant	MODIFIER	Tnfrsf14	ENSMUSG00000042333	Transcript	ENSMUST00000152687	protein_coding	rs249064527
4:154923854-154923854	intron_variant	MODIFIER	Tnfrsf14	ENSMUSG00000042333	Transcript	ENSMUST00000219534	protein_coding	rs249064527
4:154932691-154932691	upstream_gene_variant	MODIFIER	Tnfrsf14	ENSMUSG00000042333	Transcript	ENSMUST00000123514	protein_coding	rs32199916

4:154932691-154932691	upstream_gene_variant	MODIFIER	Tnfrsf14	ENSMUSG00000042333	Transcript	ENSMUST00000137803	protein_coding	rs32199916
4:154932691-154932691	upstream_gene_variant	MODIFIER	Tnfrsf14	ENSMUSG00000042333	Transcript	ENSMUST00000145296	protein_coding	rs32199916
4:154932691-154932691	upstream_gene_variant	MODIFIER	Tnfrsf14	ENSMUSG00000042333	Transcript	ENSMUST00000152687	protein_coding	rs32199916
4:154932691-154932691	upstream_gene_variant	MODIFIER	Tnfrsf14	ENSMUSG00000042333	Transcript	ENSMUST00000219534	protein_coding	rs32199916
4:155252456-155252456	intron_variant	MODIFIER	Faap20	ENSMUSG00000073684	Transcript	ENSMUST00000097747	protein_coding	rs49915416
4:155252456-155252456	intron_variant	MODIFIER	Faap20	ENSMUSG00000073684	Transcript	ENSMUST00000105627	protein_coding	rs49915416
4:155252456-155252456	downstream_gene_variant	MODIFIER	Faap20	ENSMUSG00000073684	Transcript	ENSMUST00000122957	processed_transcript	rs49915416
4:155252456-155252456	downstream_gene_variant	MODIFIER	Faap20	ENSMUSG00000073684	Transcript	ENSMUST00000126713	processed_transcript	rs49915416
4:155252456-155252456	intron_variant,non_coding_trans	MODIFIER	Faap20	ENSMUSG00000073684	Transcript	ENSMUST00000126803	processed_transcript	rs49915416
4:155252456-155252456	intron_variant	MODIFIER	Faap20	ENSMUSG00000073684	Transcript	ENSMUST00000143709	protein_coding	rs49915416
4:155252456-155252456	intron_variant,NMD_transcript_v	MODIFIER	Faap20	ENSMUSG00000073684	Transcript	ENSMUST00000148406	nonsense_mediated_decay	rs49915416
4:155252456-155252456	intron_variant,non_coding_trans	MODIFIER	Faap20	ENSMUSG00000073684	Transcript	ENSMUST00000149468	processed_transcript	rs49915416
4:155252456-155252456	intron_variant,non_coding_trans	MODIFIER	Faap20	ENSMUSG00000073684	Transcript	ENSMUST00000156404	retained_intron	rs49915416
4:155252456-155252456	intron_variant	MODIFIER	Faap20	ENSMUSG00000073684	Transcript	ENSMUST00000178473	protein_coding	rs49915416
4:155805545-155805545	intron_variant	MODIFIER	Mrpl20	ENSMUSG00000029066	Transcript	ENSMUST00000030942	protein_coding	rs49908734
4:155805545-155805545	intron_variant	MODIFIER	Mrpl20	ENSMUSG00000029066	Transcript	ENSMUST00000130188	protein_coding	rs49908734
4:155805545-155805545	intron_variant,NMD_transcript_v	MODIFIER	Mrpl20	ENSMUSG00000029066	Transcript	ENSMUST00000137487	nonsense_mediated_decay	rs49908734
4:155805545-155805545	intron_variant,non_coding_trans	MODIFIER	Mrpl20	ENSMUSG00000029066	Transcript	ENSMUST00000143503	processed_transcript	rs49908734
4:155805545-155805545	downstream_gene_variant	MODIFIER	Mrpl20	ENSMUSG00000029066	Transcript	ENSMUST00000184339	processed_transcript	rs49908734
4:155805545-155805545	intron_variant	MODIFIER	Mrpl20	ENSMUSG00000029066	Transcript	ENSMUST00000185148	protein_coding	rs49908734
4:155809006-155809006	downstream_gene_variant	MODIFIER	Mrpl20	ENSMUSG00000029066	Transcript	ENSMUST00000030942	protein_coding	rs3666643
4:155809006-155809006	upstream_gene_variant	MODIFIER	Ccnl2	ENSMUSG00000029068	Transcript	ENSMUST00000030944	protein_coding	rs3666643
4:155809006-155809006	upstream_gene_variant	MODIFIER	Ccnl2	ENSMUSG00000029068	Transcript	ENSMUST00000126346	nonsense_mediated_decay	rs3666643
4:155809006-155809006	upstream_gene_variant	MODIFIER	Ccnl2	ENSMUSG00000029068	Transcript	ENSMUST00000129850	retained_intron	rs3666643
4:155809006-155809006	downstream_gene_variant	MODIFIER	Mrpl20	ENSMUSG00000029066	Transcript	ENSMUST00000130188	protein_coding	rs3666643
4:155809006-155809006	upstream_gene_variant	MODIFIER	Ccnl2	ENSMUSG00000029068	Transcript	ENSMUST00000136370	retained_intron	rs3666643
4:155809006-155809006	intron_variant,NMD_transcript_v	MODIFIER	Mrpl20	ENSMUSG00000029066	Transcript	ENSMUST00000137487	nonsense_mediated_decay	rs3666643
4:155809006-155809006	upstream_gene_variant	MODIFIER	Ccnl2	ENSMUSG00000029068	Transcript	ENSMUST00000139066	retained_intron	rs3666643
4:155809006-155809006	downstream_gene_variant	MODIFIER	Mrpl20	ENSMUSG00000029066	Transcript	ENSMUST00000143503	processed_transcript	rs3666643
4:155809006-155809006	downstream_gene_variant	MODIFIER	Mrpl20	ENSMUSG00000029066	Transcript	ENSMUST00000185148	protein_coding	rs3666643
4:155809009-155809009	downstream_gene_variant	MODIFIER	Mrpl20	ENSMUSG00000029066	Transcript	ENSMUST00000030942	protein_coding	rs3666646
4:155809009-155809009	upstream_gene_variant	MODIFIER	Ccnl2	ENSMUSG00000029068	Transcript	ENSMUST00000030944	protein_coding	rs3666646
4:155809009-155809009	upstream_gene_variant	MODIFIER	Ccnl2	ENSMUSG00000029068	Transcript	ENSMUST00000126346	nonsense_mediated_decay	rs3666646
4:155809009-155809009	upstream_gene_variant	MODIFIER	Ccnl2	ENSMUSG00000029068	Transcript	ENSMUST00000129850	retained_intron	rs3666646
4:155809009-155809009	downstream_gene_variant	MODIFIER	Mrpl20	ENSMUSG00000029066	Transcript	ENSMUST00000130188	protein_coding	rs3666646
4:155809009-155809009	upstream_gene_variant	MODIFIER	Ccnl2	ENSMUSG00000029068	Transcript	ENSMUST00000136370	retained_intron	rs3666646
4:155809009-155809009	intron_variant,NMD_transcript_v	MODIFIER	Mrpl20	ENSMUSG00000029066	Transcript	ENSMUST00000137487	nonsense_mediated_decay	rs3666646
4:155809009-155809009	upstream_gene_variant	MODIFIER	Ccnl2	ENSMUSG00000029068	Transcript	ENSMUST00000139066	retained_intron	rs3666646
4:155809009-155809009	downstream_gene_variant	MODIFIER	Mrpl20	ENSMUSG00000029066	Transcript	ENSMUST00000143503	processed_transcript	rs3666646
4:155809009-155809009	downstream_gene_variant	MODIFIER	Mrpl20	ENSMUSG00000029066	Transcript	ENSMUST00000185148	protein_coding	rs3666646
4:155812038-155812038	downstream_gene_variant	MODIFIER	Mrpl20	ENSMUSG00000029066	Transcript	ENSMUST00000030942	protein_coding	rs3690855
4:155812038-155812038	upstream_gene_variant	MODIFIER	Ccnl2	ENSMUSG00000029068	Transcript	ENSMUST00000030944	protein_coding	rs3690855
4:155812038-155812038	upstream_gene_variant	MODIFIER	Ccnl2	ENSMUSG00000029068	Transcript	ENSMUST00000126346	nonsense_mediated_decay	rs3690855
4:155812038-155812038	upstream_gene_variant	MODIFIER	Ccnl2	ENSMUSG00000029068	Transcript	ENSMUST00000129850	retained_intron	rs3690855
4:155812038-155812038	downstream_gene_variant	MODIFIER	Mrpl20	ENSMUSG00000029066	Transcript	ENSMUST00000130188	protein_coding	rs3690855
4:155812038-155812038	upstream_gene_variant	MODIFIER	Ccnl2	ENSMUSG00000029068	Transcript	ENSMUST00000136370	retained_intron	rs3690855
4:155812038-155812038	downstream_gene_variant	MODIFIER	Mrpl20	ENSMUSG00000029066	Transcript	ENSMUST00000137487	nonsense_mediated_decay	rs3690855
4:155812038-155812038	upstream_gene_variant	MODIFIER	Ccnl2	ENSMUSG00000029068	Transcript	ENSMUST00000139066	retained_intron	rs3690855
4:155812038-155812038	downstream_gene_variant	MODIFIER	Mrpl20	ENSMUSG00000029066	Transcript	ENSMUST00000143503	processed_transcript	rs3690855
4:155812038-155812038	downstream_gene_variant	MODIFIER	Mrpl20	ENSMUSG00000029066	Transcript	ENSMUST00000185148	protein_coding	rs3690855
4:155812038-155812038	regulatory_region_variant	MODIFIER	-	-	RegulatoryFeature	ENSMUSR00000204472	promoter	rs3690855
4:155889427-155889427	downstream_gene_variant	MODIFIER	Ints11	ENSMUSG00000029034	Transcript	ENSMUST00000030901	protein_coding	rs51760569
4:155889427-155889427	upstream_gene_variant	MODIFIER	Acap3	ENSMUSG00000029033	Transcript	ENSMUST00000079031	protein_coding	rs51760569
4:155889427-155889427	splice_region_variant,intron_vari	LOW	Pusl1	ENSMUSG00000051557	Transcript	ENSMUST00000097737	protein_coding	rs51760569
4:155889427-155889427	upstream_gene_variant	MODIFIER	Acap3	ENSMUSG00000029033	Transcript	ENSMUST00000105584	protein_coding	rs51760569
4:155889427-155889427	downstream_gene_variant	MODIFIER	Ints11	ENSMUSG00000029034	Transcript	ENSMUST00000120794	protein_coding	rs51760569
4:155889427-155889427	splice_region_variant,intron_vari	LOW	Pusl1	ENSMUSG00000051557	Transcript	ENSMUST00000123504	processed_transcript	rs51760569
4:155889427-155889427	upstream_gene_variant	MODIFIER	Acap3	ENSMUSG00000029033	Transcript	ENSMUST00000126582	processed_transcript	rs51760569
4:155889427-155889427	downstream_gene_variant	MODIFIER	Pusl1	ENSMUSG00000051557	Transcript	ENSMUST00000129637	retained_intron	rs51760569
4:155889427-155889427	downstream_gene_variant	MODIFIER	Pusl1	ENSMUSG00000051557	Transcript	ENSMUST00000130146	retained_intron	rs51760569
4:155889427-155889427	upstream_gene_variant	MODIFIER	Acap3	ENSMUSG00000029033	Transcript	ENSMUST00000131168	processed_transcript	rs51760569
4:155889427-155889427	downstream_gene_variant	MODIFIER	Ints11	ENSMUSG00000029034	Transcript	ENSMUST00000132632	retained_intron	rs51760569
4:155889427-155889427	downstream_gene_variant	MODIFIER	Ints11	ENSMUSG00000029034	Transcript	ENSMUST00000134678	processed_transcript	rs51760569
4:155889427-155889427	downstream_gene_variant	MODIFIER	Ints11	ENSMUSG00000029034	Transcript	ENSMUST00000135844	retained_intron	rs51760569
4:155889427-155889427	upstream_gene_variant	MODIFIER	Acap3	ENSMUSG00000029033	Transcript	ENSMUST00000137726	processed_transcript	rs51760569
4:155889427-155889427	downstream_gene_variant	MODIFIER	Pusl1	ENSMUSG00000051557	Transcript	ENSMUST00000142566	processed_transcript	rs51760569
4:155889427-155889427	downstream_gene_variant	MODIFIER	Pusl1	ENSMUSG00000051557	Transcript	ENSMUST00000142724	retained_intron	rs51760569
4:155889427-155889427	downstream_gene_variant	MODIFIER	Pusl1	ENSMUSG00000051557	Transcript	ENSMUST00000150446	retained_intron	rs51760569
4:155889427-155889427	downstream_gene_variant	MODIFIER	Ints11	ENSMUSG00000029034	Transcript	ENSMUST00000156460	nonsense_mediated_decay	rs51760569

4:155895172-155895172	intron_variant	MODIFIER	Acap3	ENSMUSG00000029033	Transcript	ENSMUST00000079031	protein_coding	rs47536647
4:155895172-155895172	upstream_gene_variant	MODIFIER	Pusl1	ENSMUSG00000051557	Transcript	ENSMUST00000097737	protein_coding	rs47536647
4:155895172-155895172	intron_variant	MODIFIER	Acap3	ENSMUSG00000029033	Transcript	ENSMUST00000105584	protein_coding	rs47536647
4:155895172-155895172	intron_variant,non_coding_trans	MODIFIER	Acap3	ENSMUSG00000029033	Transcript	ENSMUST00000126582	processed_transcript	rs47536647
4:155895172-155895172	upstream_gene_variant	MODIFIER	Pusl1	ENSMUSG00000051557	Transcript	ENSMUST00000129637	retained_intron	rs47536647
4:155895172-155895172	upstream_gene_variant	MODIFIER	Pusl1	ENSMUSG00000051557	Transcript	ENSMUST00000130146	retained_intron	rs47536647
4:155895172-155895172	intron_variant,non_coding_trans	MODIFIER	Acap3	ENSMUSG00000029033	Transcript	ENSMUST00000131168	processed_transcript	rs47536647
4:155895172-155895172	intron_variant,non_coding_trans	MODIFIER	Acap3	ENSMUSG00000029033	Transcript	ENSMUST00000137726	processed_transcript	rs47536647
4:155895172-155895172	upstream_gene_variant	MODIFIER	Pusl1	ENSMUSG00000051557	Transcript	ENSMUST00000142566	processed_transcript	rs47536647
4:155895172-155895172	upstream_gene_variant	MODIFIER	Pusl1	ENSMUSG00000051557	Transcript	ENSMUST00000142724	retained_intron	rs47536647
4:155895172-155895172	upstream_gene_variant	MODIFIER	Pusl1	ENSMUSG00000051557	Transcript	ENSMUST00000150446	retained_intron	rs47536647
4:155895172-155895172	regulatory_region_variant	MODIFIER	-	-	RegulatoryFeature	ENSMUSR00000204486	promoter	rs47536647
4:155895728-155895729	intron_variant	MODIFIER	Acap3	ENSMUSG00000029033	Transcript	ENSMUST00000079031	protein_coding	rs248031195
4:155895728-155895729	upstream_gene_variant	MODIFIER	Pusl1	ENSMUSG00000051557	Transcript	ENSMUST00000097737	protein_coding	rs248031195
4:155895728-155895729	intron_variant	MODIFIER	Acap3	ENSMUSG00000029033	Transcript	ENSMUST00000105584	protein_coding	rs248031195
4:155895728-155895729	intron_variant,non_coding_trans	MODIFIER	Acap3	ENSMUSG00000029033	Transcript	ENSMUST00000126582	processed_transcript	rs248031195
4:155895728-155895729	upstream_gene_variant	MODIFIER	Pusl1	ENSMUSG00000051557	Transcript	ENSMUST00000129637	retained_intron	rs248031195
4:155895728-155895729	upstream_gene_variant	MODIFIER	Pusl1	ENSMUSG00000051557	Transcript	ENSMUST00000130146	retained_intron	rs248031195
4:155895728-155895729	intron_variant,non_coding_trans	MODIFIER	Acap3	ENSMUSG00000029033	Transcript	ENSMUST00000131168	processed_transcript	rs248031195
4:155895728-155895729	intron_variant,non_coding_trans	MODIFIER	Acap3	ENSMUSG00000029033	Transcript	ENSMUST00000137726	processed_transcript	rs248031195
4:155895728-155895729	upstream_gene_variant	MODIFIER	Pusl1	ENSMUSG00000051557	Transcript	ENSMUST00000142566	processed_transcript	rs248031195
4:155895728-155895729	upstream_gene_variant	MODIFIER	Pusl1	ENSMUSG00000051557	Transcript	ENSMUST00000142724	retained_intron	rs248031195
4:155895728-155895729	upstream_gene_variant	MODIFIER	Pusl1	ENSMUSG00000051557	Transcript	ENSMUST00000150446	retained_intron	rs248031195
4:155908341-155908341	downstream_gene_variant	MODIFIER	Acap3	ENSMUSG00000029033	Transcript	ENSMUST00000079031	protein_coding	rs3725431
4:155908341-155908341	downstream_gene_variant	MODIFIER	Acap3	ENSMUSG00000029033	Transcript	ENSMUST00000105584	protein_coding	rs3725431
4:155908341-155908341	downstream_gene_variant	MODIFIER	Acap3	ENSMUSG00000029033	Transcript	ENSMUST00000131372	processed_transcript	rs3725431
4:155908341-155908341	downstream_gene_variant	MODIFIER	Acap3	ENSMUSG00000029033	Transcript	ENSMUST00000137726	processed_transcript	rs3725431
4:155908341-155908341	downstream_gene_variant	MODIFIER	Acap3	ENSMUSG00000029033	Transcript	ENSMUST00000140855	processed_transcript	rs3725431
4:155908341-155908341	downstream_gene_variant	MODIFIER	Acap3	ENSMUSG00000029033	Transcript	ENSMUST00000145654	retained_intron	rs3725431
4:155908341-155908341	upstream_gene_variant	MODIFIER	Gm22991	ENSMUSG00000096174	Transcript	ENSMUST00000178126	miRNA	rs3725431
4:155908562-155908562	downstream_gene_variant	MODIFIER	Acap3	ENSMUSG00000029033	Transcript	ENSMUST00000079031	protein_coding	rs32410455
4:155908562-155908562	downstream_gene_variant	MODIFIER	Acap3	ENSMUSG00000029033	Transcript	ENSMUST00000105584	protein_coding	rs32410455
4:155908562-155908562	downstream_gene_variant	MODIFIER	Acap3	ENSMUSG00000029033	Transcript	ENSMUST00000131372	processed_transcript	rs32410455
4:155908562-155908562	downstream_gene_variant	MODIFIER	Acap3	ENSMUSG00000029033	Transcript	ENSMUST00000137726	processed_transcript	rs32410455
4:155908562-155908562	downstream_gene_variant	MODIFIER	Acap3	ENSMUSG00000029033	Transcript	ENSMUST00000140855	processed_transcript	rs32410455
4:155908562-155908562	downstream_gene_variant	MODIFIER	Acap3	ENSMUSG00000029033	Transcript	ENSMUST00000145654	retained_intron	rs32410455
4:155908562-155908562	upstream_gene_variant	MODIFIER	Gm22991	ENSMUSG00000096174	Transcript	ENSMUST00000178126	miRNA	rs32410455
4:155909517-155909517	downstream_gene_variant	MODIFIER	Acap3	ENSMUSG00000029033	Transcript	ENSMUST00000079031	protein_coding	rs32231309
4:155909517-155909517	downstream_gene_variant	MODIFIER	Acap3	ENSMUSG00000029033	Transcript	ENSMUST00000105584	protein_coding	rs32231309
4:155909517-155909517	downstream_gene_variant	MODIFIER	Acap3	ENSMUSG00000029033	Transcript	ENSMUST00000131372	processed_transcript	rs32231309
4:155909517-155909517	downstream_gene_variant	MODIFIER	Acap3	ENSMUSG00000029033	Transcript	ENSMUST00000137726	processed_transcript	rs32231309
4:155909517-155909517	downstream_gene_variant	MODIFIER	Acap3	ENSMUSG00000029033	Transcript	ENSMUST00000140855	processed_transcript	rs32231309
4:155909517-155909517	downstream_gene_variant	MODIFIER	Acap3	ENSMUSG00000029033	Transcript	ENSMUST00000145654	retained_intron	rs32231309
4:155909517-155909517	upstream_gene_variant	MODIFIER	Gm22991	ENSMUSG00000096174	Transcript	ENSMUST00000178126	miRNA	rs32231309
4:155910747-155910747	downstream_gene_variant	MODIFIER	Acap3	ENSMUSG00000029033	Transcript	ENSMUST00000079031	protein_coding	rs46399013
4:155910747-155910747	downstream_gene_variant	MODIFIER	Acap3	ENSMUSG00000029033	Transcript	ENSMUST00000105584	protein_coding	rs46399013
4:155910747-155910747	downstream_gene_variant	MODIFIER	Acap3	ENSMUSG00000029033	Transcript	ENSMUST00000137726	processed_transcript	rs46399013
4:155910747-155910747	downstream_gene_variant	MODIFIER	Acap3	ENSMUSG00000029033	Transcript	ENSMUST00000140855	processed_transcript	rs46399013
4:155910747-155910747	upstream_gene_variant	MODIFIER	Gm22991	ENSMUSG00000096174	Transcript	ENSMUST00000178126	miRNA	rs46399013
4:155928071-155928071	intergenic_variant	MODIFIER	-	-	-	-	-	rs227265312
4:155928227-155928227	regulatory_region_variant	MODIFIER	-	-	RegulatoryFeature	ENSMUSR00000675286	promoter_flanking_region	rs48323324
4:155928227-155928227	regulatory_region_variant	MODIFIER	-	-	RegulatoryFeature	ENSMUSR00000675287	CTCF_binding_site	rs48323324
4:155928227-155928227	intergenic_variant	MODIFIER	-	-	-	-	-	rs48323324
4:155928266-155928266	regulatory_region_variant	MODIFIER	-	-	RegulatoryFeature	ENSMUSR00000675286	promoter_flanking_region	rs46724433
4:155928266-155928266	regulatory_region_variant	MODIFIER	-	-	RegulatoryFeature	ENSMUSR00000675287	CTCF_binding_site	rs46724433
4:155928266-155928266	intergenic_variant	MODIFIER	-	-	-	-	-	rs46724433
4:32022582-32022582	3_prime_UTR_variant	MODIFIER	Map3k7	ENSMUSG00000028284	Transcript	ENSMUST00000037607	protein_coding	-
4:32022582-32022582	3_prime_UTR_variant	MODIFIER	Map3k7	ENSMUSG00000028284	Transcript	ENSMUST00000080933	protein_coding	-
4:32022582-32022582	3_prime_UTR_variant	MODIFIER	Map3k7	ENSMUSG00000028284	Transcript	ENSMUST00000108183	protein_coding	-
4:32022582-32022582	3_prime_UTR_variant	MODIFIER	Map3k7	ENSMUSG00000028284	Transcript	ENSMUST00000108184	protein_coding	-
4:32022582-32022582	downstream_gene_variant	MODIFIER	Map3k7	ENSMUSG00000028284	Transcript	ENSMUST00000131310	processed_transcript	-
4:3554421-3554421	splice_region_variant,intron_vari	LOW	Tmem68	ENSMUSG00000028232	Transcript	ENSMUST00000029891	protein_coding	rs221598772
4:3554421-3554421	upstream_gene_variant	MODIFIER	Tmem68	ENSMUSG00000028232	Transcript	ENSMUST00000134806	retained_intron	rs221598772
4:3554421-3554421	splice_region_variant,intron_vari	LOW	Tmem68	ENSMUSG00000028232	Transcript	ENSMUST00000154922	nonsense_mediated_decay	rs221598772
4:3677607-3677607	upstream_gene_variant	MODIFIER	Lyn	ENSMUSG00000042228	Transcript	ENSMUST00000041377	protein_coding	rs32577200
4:3677607-3677607	upstream_gene_variant	MODIFIER	Lyn	ENSMUSG00000042228	Transcript	ENSMUST00000103010	protein_coding	rs32577200
4:3677607-3677607	upstream_gene_variant	MODIFIER	Lyn	ENSMUSG00000042228	Transcript	ENSMUST00000145083	processed_transcript	rs32577200
4:3677607-3677607	downstream_gene_variant	MODIFIER	Gm26857	ENSMUSG00000097860	Transcript	ENSMUST00000181580	lincRNA	rs32577200
4:3677607-3677607	regulatory_region_variant	MODIFIER	-	-	RegulatoryFeature	ENSMUSR00000186649	promoter	rs32577200

4:3684160-3684160	intron_variant	MODIFIER	Lyn	ENSMUSG00000042228	Transcript	ENSMUST00000041377	protein_coding	rs32350600
4:3684160-3684160	intron_variant	MODIFIER	Lyn	ENSMUSG00000042228	Transcript	ENSMUST00000103010	protein_coding	rs32350600
4:3684160-3684160	intron_variant,non_coding_trans	MODIFIER	Lyn	ENSMUSG00000042228	Transcript	ENSMUST00000145083	processed_transcript	rs32350600
4:3684160-3684160	intron_variant,non_coding_trans	MODIFIER	Gm26857	ENSMUSG00000097860	Transcript	ENSMUST00000181580	lincRNA	rs32350600
4:3798780-3798780	upstream_gene_variant	MODIFIER	Gm22781	ENSMUSG00000077271	Transcript	ENSMUST00000104083	snoRNA	rs27722533
4:3798780-3798780	intron_variant,non_coding_trans	MODIFIER	Lyn	ENSMUSG00000042228	Transcript	ENSMUST00000137943	processed_transcript	rs27722533
4:3798780-3798780	regulatory_region_variant	MODIFIER	-	-	RegulatoryFeature	ENSMUSR00000656831	promoter_flanking_region	rs27722533
4:3831839-3831841	downstream_gene_variant	MODIFIER	Rps20	ENSMUSG00000028234	Transcript	ENSMUST00000103009	retained_intron	rs387841377
4:3831839-3831841	downstream_gene_variant	MODIFIER	Rps20	ENSMUSG00000028234	Transcript	ENSMUST00000130128	retained_intron	rs387841377
4:3831839-3831841	3_prime_UTR_variant	MODIFIER	Rps20	ENSMUSG00000028234	Transcript	ENSMUST00000138502	protein_coding	rs387841377
4:3831839-3831841	downstream_gene_variant	MODIFIER	Gm24016	ENSMUSG00000088351	Transcript	ENSMUST00000157726	snoRNA	rs387841377
4:46582584-46582584	intron_variant	MODIFIER	Coro2a	ENSMUSG00000028337	Transcript	ENSMUST00000107756	protein_coding	rs108527415
4:46582584-46582584	intron_variant,non_coding_trans	MODIFIER	Coro2a	ENSMUSG00000028337	Transcript	ENSMUST00000127304	processed_transcript	rs108527415
4:46582584-46582584	intron_variant,non_coding_trans	MODIFIER	Coro2a	ENSMUSG00000028337	Transcript	ENSMUST00000139179	retained_intron	rs108527415
4:46582584-46582584	regulatory_region_variant	MODIFIER	-	-	RegulatoryFeature	ENSMUSR00000190314	promoter_flanking_region	rs108527415
4:55294764-55294764	downstream_gene_variant	MODIFIER	Gm25419	ENSMUSG00000077403	Transcript	ENSMUST00000104215	snoRNA	rs27791710
4:55294764-55294764	regulatory_region_variant	MODIFIER	-	-	RegulatoryFeature	ENSMUSR00000191218	promoter_flanking_region	rs27791710
4:55392019-55392019	3_prime_UTR_variant	MODIFIER	Rad23b	ENSMUSG00000028426	Transcript	ENSMUST00000030134	protein_coding	rs27774136
4:55392019-55392019	downstream_gene_variant	MODIFIER	Gm12516	ENSMUSG00000084144	Transcript	ENSMUST00000120819	processed_pseudogene	rs27774136
4:55531024-55531024	intron_variant	MODIFIER	Klf4	ENSMUSG00000003032	Transcript	ENSMUST00000107619	protein_coding	rs33024037
4:55531024-55531024	intron_variant	MODIFIER	Klf4	ENSMUSG00000003032	Transcript	ENSMUST00000129250	protein_coding	rs33024037
4:55531024-55531024	intron_variant	MODIFIER	Klf4	ENSMUSG00000003032	Transcript	ENSMUST00000132746	protein_coding	rs33024037
4:55531024-55531024	regulatory_region_variant	MODIFIER	-	-	RegulatoryFeature	ENSMUSR00000191275	promoter	rs33024037
4:55531025-55531025	intron_variant	MODIFIER	Klf4	ENSMUSG00000003032	Transcript	ENSMUST00000107619	protein_coding	rs32258067
4:55531025-55531025	intron_variant	MODIFIER	Klf4	ENSMUSG00000003032	Transcript	ENSMUST00000129250	protein_coding	rs32258067
4:55531025-55531025	intron_variant	MODIFIER	Klf4	ENSMUSG00000003032	Transcript	ENSMUST00000132746	protein_coding	rs32258067
4:55531025-55531025	regulatory_region_variant	MODIFIER	-	-	RegulatoryFeature	ENSMUSR00000191275	promoter	rs32258067
4:59025645-59025645	downstream_gene_variant	MODIFIER	Dnajc25	ENSMUSG00000070972	Transcript	ENSMUST00000095070	protein_coding	rs32856947
4:59025645-59025645	downstream_gene_variant	MODIFIER	Dnajc25	ENSMUSG00000070972	Transcript	ENSMUST00000150309	nonsense_mediated_decay	rs32856947
4:59025645-59025645	downstream_gene_variant	MODIFIER	Dnajc25	ENSMUSG00000070972	Transcript	ENSMUST00000152199	protein_coding	rs32856947
4:59025645-59025645	downstream_gene_variant	MODIFIER	Dnajc25	ENSMUSG00000070972	Transcript	ENSMUST00000153467	nonsense_mediated_decay	rs32856947
4:59025645-59025645	intron_variant	MODIFIER	Gm20503	ENSMUSG00000092345	Transcript	ENSMUST00000174664	protein_coding	rs32856947
4:59025646-59025646	downstream_gene_variant	MODIFIER	Dnajc25	ENSMUSG00000070972	Transcript	ENSMUST00000095070	protein_coding	rs32659270
4:59025646-59025646	downstream_gene_variant	MODIFIER	Dnajc25	ENSMUSG00000070972	Transcript	ENSMUST00000150309	nonsense_mediated_decay	rs32659270
4:59025646-59025646	downstream_gene_variant	MODIFIER	Dnajc25	ENSMUSG00000070972	Transcript	ENSMUST00000152199	protein_coding	rs32659270
4:59025646-59025646	downstream_gene_variant	MODIFIER	Dnajc25	ENSMUSG00000070972	Transcript	ENSMUST00000153467	nonsense_mediated_decay	rs32659270
4:59025646-59025646	intron_variant	MODIFIER	Gm20503	ENSMUSG00000092345	Transcript	ENSMUST00000174664	protein_coding	rs32659270
4:63356589-63356589	missense_variant	MODERATE	Orm3	ENSMUSG00000028359	Transcript	ENSMUST00000006687	protein_coding	rs27884279
4:63356589-63356589	downstream_gene_variant	MODIFIER	Gm11212	ENSMUSG00000082141	Transcript	ENSMUST00000118337	unprocessed_pseudogene	rs27884279
4:63356589-63356589	regulatory_region_variant	MODIFIER	-	-	RegulatoryFeature	ENSMUSR00000662357	enhancer	rs27884279
4:63356599-63356599	missense_variant	MODERATE	Orm3	ENSMUSG00000028359	Transcript	ENSMUST00000006687	protein_coding	rs27884278
4:63356599-63356599	downstream_gene_variant	MODIFIER	Gm11212	ENSMUSG00000082141	Transcript	ENSMUST00000118337	unprocessed_pseudogene	rs27884278
4:63356599-63356599	regulatory_region_variant	MODIFIER	-	-	RegulatoryFeature	ENSMUSR00000662357	enhancer	rs27884278
4:63356708-63356708	synonymous_variant	LOW	Orm3	ENSMUSG00000028359	Transcript	ENSMUST00000006687	protein_coding	rs27884277
4:63356708-63356708	downstream_gene_variant	MODIFIER	Gm11212	ENSMUSG00000082141	Transcript	ENSMUST00000118337	unprocessed_pseudogene	rs27884277
4:63356708-63356708	regulatory_region_variant	MODIFIER	-	-	RegulatoryFeature	ENSMUSR00000662357	enhancer	rs27884277
4:63356730-63356730	intron_variant	MODIFIER	Orm3	ENSMUSG00000028359	Transcript	ENSMUST00000006687	protein_coding	rs46330627
4:63356730-63356730	downstream_gene_variant	MODIFIER	Gm11212	ENSMUSG00000082141	Transcript	ENSMUST00000118337	unprocessed_pseudogene	rs46330627
4:63356730-63356730	regulatory_region_variant	MODIFIER	-	-	RegulatoryFeature	ENSMUSR00000662357	enhancer	rs46330627
4:63357242-63357242	intron_variant	MODIFIER	Orm3	ENSMUSG00000028359	Transcript	ENSMUST00000006687	protein_coding	rs27884272
4:63357242-63357242	downstream_gene_variant	MODIFIER	Gm11212	ENSMUSG00000082141	Transcript	ENSMUST00000118337	unprocessed_pseudogene	rs27884272
4:63359429-63359429	3_prime_UTR_variant	MODIFIER	Orm3	ENSMUSG00000028359	Transcript	ENSMUST00000006687	protein_coding	rs46823976
4:63359429-63359429	upstream_gene_variant	MODIFIER	Orm2	ENSMUSG00000061540	Transcript	ENSMUST00000075341	protein_coding	rs46823976
4:63359454-63359454	3_prime_UTR_variant	MODIFIER	Orm3	ENSMUSG00000028359	Transcript	ENSMUST00000006687	protein_coding	rs49938250
4:63359454-63359454	upstream_gene_variant	MODIFIER	Orm2	ENSMUSG00000061540	Transcript	ENSMUST00000075341	protein_coding	rs49938250
4:63551277-63551277	downstream_gene_variant	MODIFIER	Atp6v1g1	ENSMUSG00000039105	Transcript	ENSMUST00000035301	protein_coding	rs32587139
4:6741550-6741550	synonymous_variant	LOW	Tox	ENSMUSG00000041272	Transcript	ENSMUST00000039987	protein_coding	rs27657701
4:6741550-6741550	non_coding_transcript_exon_var	MODIFIER	Tox	ENSMUSG00000041272	Transcript	ENSMUST00000147657	retained_intron	rs27657701
4:6741559-6741559	missense_variant	MODERATE	Tox	ENSMUSG00000041272	Transcript	ENSMUST00000039987	protein_coding	rs27657700
4:6741559-6741559	non_coding_transcript_exon_var	MODIFIER	Tox	ENSMUSG00000041272	Transcript	ENSMUST00000147657	retained_intron	rs27657700
4:8145899-8145899	3_prime_UTR_variant	MODIFIER	Car8	ENSMUSG00000041261	Transcript	ENSMUST00000066674	protein_coding	rs3022975
4:8146020-8146022	3_prime_UTR_variant	MODIFIER	Car8	ENSMUSG00000041261	Transcript	ENSMUST00000066674	protein_coding	rs234209929
4:8146044-8146044	3_prime_UTR_variant	MODIFIER	Car8	ENSMUSG00000041261	Transcript	ENSMUST00000066674	protein_coding	rs27660680
4:8146190-8146190	3_prime_UTR_variant	MODIFIER	Car8	ENSMUSG00000041261	Transcript	ENSMUST00000066674	protein_coding	rs46508305
4:8146191-8146191	3_prime_UTR_variant	MODIFIER	Car8	ENSMUSG00000041261	Transcript	ENSMUST00000066674	protein_coding	rs27660679
4:8592223-8592223	intron_variant	MODIFIER	Rab2a	ENSMUSG00000047187	Transcript	ENSMUST00000060232	protein_coding	rs49216954
4:8595089-8595089	intron_variant	MODIFIER	Rab2a	ENSMUSG00000047187	Transcript	ENSMUST00000060232	protein_coding	rs27676358
4:8599006-8599006	intron_variant	MODIFIER	Rab2a	ENSMUSG00000047187	Transcript	ENSMUST00000060232	protein_coding	rs32049485
4:8602367-8602367	intron_variant	MODIFIER	Rab2a	ENSMUSG00000047187	Transcript	ENSMUST00000060232	protein_coding	rs27676328

4:94088821-94088821	downstream_gene_variant	MODIFIER	Gm12648	ENSMUSG00000085931	Transcript	ENSMUST00000126270	lincRNA	rs32008015
5:100566414-100566414	intron_variant	MODIFIER	Plac8	ENSMUSG00000029322	Transcript	ENSMUST00000031264	protein_coding	rs33151758
5:100566414-100566414	upstream_gene_variant	MODIFIER	Plac8	ENSMUSG00000029322	Transcript	ENSMUST00000097437	protein_coding	rs33151758
5:100566414-100566414	upstream_gene_variant	MODIFIER	Plac8	ENSMUSG00000029322	Transcript	ENSMUST00000112910	protein_coding	rs33151758
5:100566414-100566414	intron_variant,non_coding_trans	MODIFIER	Plac8	ENSMUSG00000029322	Transcript	ENSMUST00000144595	retained_intron	rs33151758
5:107902834-107902834	intron_variant	MODIFIER	Rpl5	ENSMUSG00000058558	Transcript	ENSMUST00000082223	protein_coding	rs33691132
5:107902834-107902834	upstream_gene_variant	MODIFIER	Snord21	ENSMUSG00000064453	Transcript	ENSMUST00000082519	snoRNA	rs33691132
5:107902834-107902834	upstream_gene_variant	MODIFIER	Gm22270	ENSMUSG00000077222	Transcript	ENSMUST00000104034	snoRNA	rs33691132
5:107902834-107902834	upstream_gene_variant	MODIFIER	Gm26387	ENSMUSG00000077426	Transcript	ENSMUST00000104238	snoRNA	rs33691132
5:107902834-107902834	intron_variant,non_coding_trans	MODIFIER	Rpl5	ENSMUSG00000058558	Transcript	ENSMUST00000123183	retained_intron	rs33691132
5:107902834-107902834	intron_variant,non_coding_trans	MODIFIER	Rpl5	ENSMUSG00000058558	Transcript	ENSMUST00000129944	retained_intron	rs33691132
5:107902834-107902834	upstream_gene_variant	MODIFIER	Rpl5	ENSMUSG00000058558	Transcript	ENSMUST00000140659	retained_intron	rs33691132
5:107902834-107902834	upstream_gene_variant	MODIFIER	Rpl5	ENSMUSG00000058558	Transcript	ENSMUST00000151767	retained_intron	rs33691132
5:107902834-107902834	intron_variant	MODIFIER	Rpl5	ENSMUSG00000058558	Transcript	ENSMUST00000153590	protein_coding	rs33691132
5:108097005-108097005	intron_variant	MODIFIER	Mtf2	ENSMUSG00000029267	Transcript	ENSMUST00000081567	protein_coding	rs33649355
5:108097005-108097005	intron_variant	MODIFIER	Mtf2	ENSMUSG00000029267	Transcript	ENSMUST00000112626	protein_coding	rs33649355
5:108097005-108097005	intron_variant,NMD_transcript_v	MODIFIER	Mtf2	ENSMUSG00000029267	Transcript	ENSMUST00000124195	nonsense_mediated_decay	rs33649355
5:108097005-108097005	intron_variant,NMD_transcript_v	MODIFIER	Mtf2	ENSMUSG00000029267	Transcript	ENSMUST00000129921	nonsense_mediated_decay	rs33649355
5:108097005-108097005	intron_variant,non_coding_trans	MODIFIER	Mtf2	ENSMUSG00000029267	Transcript	ENSMUST00000131264	processed_transcript	rs33649355
5:108097005-108097005	intron_variant,non_coding_trans	MODIFIER	Mtf2	ENSMUSG00000029267	Transcript	ENSMUST00000131291	processed_transcript	rs33649355
5:108097005-108097005	intron_variant,NMD_transcript_v	MODIFIER	Mtf2	ENSMUSG00000029267	Transcript	ENSMUST00000134026	nonsense_mediated_decay	rs33649355
5:108097005-108097005	intron_variant	MODIFIER	Mtf2	ENSMUSG00000029267	Transcript	ENSMUST00000137996	protein_coding	rs33649355
5:108097005-108097005	intron_variant,non_coding_trans	MODIFIER	Mtf2	ENSMUSG00000029267	Transcript	ENSMUST00000141592	processed_transcript	rs33649355
5:108097005-108097005	intron_variant,NMD_transcript_v	MODIFIER	Mtf2	ENSMUSG00000029267	Transcript	ENSMUST00000143412	nonsense_mediated_decay	rs33649355
5:108097005-108097005	downstream_gene_variant	MODIFIER	Mtf2	ENSMUSG00000029267	Transcript	ENSMUST00000170319	protein_coding	rs33649355
5:108097005-108097005	intron_variant,non_coding_trans	MODIFIER	Mtf2	ENSMUSG00000029267	Transcript	ENSMUST00000198662	processed_transcript	rs33649355
5:108128001-108128001	intron_variant	MODIFIER	Tmed5	ENSMUSG00000063406	Transcript	ENSMUST00000002837	protein_coding	rs259364096
5:108128001-108128001	upstream_gene_variant	MODIFIER	Ccdc18	ENSMUSG00000056531	Transcript	ENSMUST00000047677	protein_coding	rs259364096
5:108128001-108128001	intron_variant	MODIFIER	Tmed5	ENSMUSG00000063406	Transcript	ENSMUST00000061203	protein_coding	rs259364096
5:108128001-108128001	downstream_gene_variant	MODIFIER	Tmed5	ENSMUSG00000063406	Transcript	ENSMUST00000117759	protein_coding	rs259364096
5:108128001-108128001	intron_variant	MODIFIER	Tmed5	ENSMUSG00000063406	Transcript	ENSMUST00000118036	protein_coding	rs259364096
5:108128001-108128001	intron_variant	MODIFIER	Tmed5	ENSMUSG00000063406	Transcript	ENSMUST00000119437	protein_coding	rs259364096
5:108128001-108128001	downstream_gene_variant	MODIFIER	Tmed5	ENSMUSG00000063406	Transcript	ENSMUST00000119784	protein_coding	rs259364096
5:108128001-108128001	upstream_gene_variant	MODIFIER	Ccdc18	ENSMUSG00000056531	Transcript	ENSMUST00000197718	nonsense_mediated_decay	rs259364096
5:108128001-108128001	upstream_gene_variant	MODIFIER	Ccdc18	ENSMUSG00000056531	Transcript	ENSMUST00000200463	retained_intron	rs259364096
5:108129672-108129672	intron_variant	MODIFIER	Tmed5	ENSMUSG00000063406	Transcript	ENSMUST00000002837	protein_coding	rs29624612
5:108129672-108129672	upstream_gene_variant	MODIFIER	Ccdc18	ENSMUSG00000056531	Transcript	ENSMUST00000047677	protein_coding	rs29624612
5:108129672-108129672	intron_variant	MODIFIER	Tmed5	ENSMUSG00000063406	Transcript	ENSMUST00000061203	protein_coding	rs29624612
5:108129672-108129672	intron_variant	MODIFIER	Tmed5	ENSMUSG00000063406	Transcript	ENSMUST00000117759	protein_coding	rs29624612
5:108129672-108129672	intron_variant	MODIFIER	Tmed5	ENSMUSG00000063406	Transcript	ENSMUST00000118036	protein_coding	rs29624612
5:108129672-108129672	intron_variant	MODIFIER	Tmed5	ENSMUSG00000063406	Transcript	ENSMUST00000119437	protein_coding	rs29624612
5:108129672-108129672	downstream_gene_variant	MODIFIER	Tmed5	ENSMUSG00000063406	Transcript	ENSMUST00000119784	protein_coding	rs29624612
5:108129672-108129672	upstream_gene_variant	MODIFIER	Ccdc18	ENSMUSG00000056531	Transcript	ENSMUST00000197718	nonsense_mediated_decay	rs29624612
5:108129672-108129672	upstream_gene_variant	MODIFIER	Ccdc18	ENSMUSG00000056531	Transcript	ENSMUST00000200463	retained_intron	rs29624612
5:108571611-108571611	intron_variant	MODIFIER	Gak	ENSMUSG00000062234	Transcript	ENSMUST00000046603	protein_coding	rs33626156
5:108571611-108571611	non_coding_transcript_exon_var	MODIFIER	Gak	ENSMUSG00000062234	Transcript	ENSMUST00000133745	retained_intron	rs33626156
5:108571611-108571611	intron_variant,NMD_transcript_v	MODIFIER	Gak	ENSMUSG00000062234	Transcript	ENSMUST00000135225	nonsense_mediated_decay	rs33626156
5:108571611-108571611	intron_variant,NMD_transcript_v	MODIFIER	Gak	ENSMUSG00000062234	Transcript	ENSMUST00000145467	nonsense_mediated_decay	rs33626156
5:108571611-108571611	intron_variant	MODIFIER	Gak	ENSMUSG00000062234	Transcript	ENSMUST00000156110	protein_coding	rs33626156
5:108571611-108571611	downstream_gene_variant	MODIFIER	Gak	ENSMUSG00000062234	Transcript	ENSMUST00000199010	processed_transcript	rs33626156
5:108571611-108571611	intron_variant,NMD_transcript_v	MODIFIER	Gak	ENSMUSG00000062234	Transcript	ENSMUST00000199048	nonsense_mediated_decay	rs33626156
5:108631957-108631957	upstream_gene_variant	MODIFIER	Gak	ENSMUSG00000062234	Transcript	ENSMUST00000046603	protein_coding	rs29764584
5:108631957-108631957	intron_variant	MODIFIER	Tmem175	ENSMUSG00000013495	Transcript	ENSMUST00000063272	protein_coding	rs29764584
5:108631957-108631957	intron_variant	MODIFIER	Tmem175	ENSMUSG00000013495	Transcript	ENSMUST00000078323	protein_coding	rs29764584
5:108631957-108631957	intron_variant	MODIFIER	Tmem175	ENSMUSG00000013495	Transcript	ENSMUST00000120327	protein_coding	rs29764584
5:108631957-108631957	upstream_gene_variant	MODIFIER	Gak	ENSMUSG00000062234	Transcript	ENSMUST00000135225	nonsense_mediated_decay	rs29764584
5:108631957-108631957	upstream_gene_variant	MODIFIER	Gak	ENSMUSG00000062234	Transcript	ENSMUST00000145467	nonsense_mediated_decay	rs29764584
5:108631957-108631957	upstream_gene_variant	MODIFIER	Gak	ENSMUSG00000062234	Transcript	ENSMUST00000145935	processed_transcript	rs29764584
5:108631957-108631957	intron_variant	MODIFIER	Tmem175	ENSMUSG00000013495	Transcript	ENSMUST00000146207	protein_coding	rs29764584
5:108631957-108631957	upstream_gene_variant	MODIFIER	Gak	ENSMUSG00000062234	Transcript	ENSMUST00000199048	nonsense_mediated_decay	rs29764584
5:108631957-108631957	upstream_gene_variant	MODIFIER	Gak	ENSMUSG00000062234	Transcript	ENSMUST00000199662	nonsense_mediated_decay	rs29764584
5:108635349-108635349	intron_variant	MODIFIER	Tmem175	ENSMUSG00000013495	Transcript	ENSMUST00000063272	protein_coding	rs3709503
5:108635349-108635349	intron_variant	MODIFIER	Tmem175	ENSMUSG00000013495	Transcript	ENSMUST00000078323	protein_coding	rs3709503
5:108635349-108635349	intron_variant	MODIFIER	Tmem175	ENSMUSG00000013495	Transcript	ENSMUST00000120327	protein_coding	rs3709503
5:108635349-108635349	intron_variant	MODIFIER	Tmem175	ENSMUSG00000013495	Transcript	ENSMUST00000146207	protein_coding	rs3709503
5:108653568-108653568	intron_variant	MODIFIER	Dgkq	ENSMUSG00000004815	Transcript	ENSMUST00000053913	protein_coding	rs33313275
5:108653568-108653568	downstream_gene_variant	MODIFIER	Tmem175	ENSMUSG00000013495	Transcript	ENSMUST00000063272	protein_coding	rs33313275
5:108653568-108653568	intron_variant,non_coding_trans	MODIFIER	Dgkq	ENSMUSG00000004815	Transcript	ENSMUST00000123669	retained_intron	rs33313275
5:108653568-108653568	intron_variant,NMD_transcript_v	MODIFIER	Dgkq	ENSMUSG00000004815	Transcript	ENSMUST00000132179	nonsense_mediated_decay	rs33313275

5:108653568-108653568	intron_variant,NMD_transcript_v	MODIFIER	Dgkq	ENSMUSG00000004815	Transcript	ENSMUST00000132708	nonsense_mediated_decay	rs33313275
5:108653568-108653568	upstream_gene_variant	MODIFIER	Dgkq	ENSMUSG00000004815	Transcript	ENSMUST00000139169	retained_intron	rs33313275
5:108653568-108653568	downstream_gene_variant	MODIFIER	Dgkq	ENSMUSG00000004815	Transcript	ENSMUST00000139598	retained_intron	rs33313275
5:108653568-108653568	intron_variant,non_coding_trans	MODIFIER	Dgkq	ENSMUSG00000004815	Transcript	ENSMUST00000144624	retained_intron	rs33313275
5:108653568-108653568	upstream_gene_variant	MODIFIER	Dgkq	ENSMUSG00000004815	Transcript	ENSMUST00000145917	retained_intron	rs33313275
5:108653568-108653568	intron_variant,NMD_transcript_v	MODIFIER	Dgkq	ENSMUSG00000004815	Transcript	ENSMUST00000153238	nonsense_mediated_decay	rs33313275
5:108653568-108653568	intron_variant,non_coding_trans	MODIFIER	Dgkq	ENSMUSG00000004815	Transcript	ENSMUST00000153365	retained_intron	rs33313275
5:108653568-108653568	non_coding_transcript_exon_var	MODIFIER	Dgkq	ENSMUSG00000004815	Transcript	ENSMUST00000156964	retained_intron	rs33313275
5:108653865-108653865	intron_variant	MODIFIER	Dgkq	ENSMUSG00000004815	Transcript	ENSMUST00000053913	protein_coding	rs29717159
5:108653865-108653865	upstream_gene_variant	MODIFIER	Dgkq	ENSMUSG00000004815	Transcript	ENSMUST00000123669	retained_intron	rs29717159
5:108653865-108653865	intron_variant,NMD_transcript_v	MODIFIER	Dgkq	ENSMUSG00000004815	Transcript	ENSMUST00000132179	nonsense_mediated_decay	rs29717159
5:108653865-108653865	intron_variant,NMD_transcript_v	MODIFIER	Dgkq	ENSMUSG00000004815	Transcript	ENSMUST00000132708	nonsense_mediated_decay	rs29717159
5:108653865-108653865	upstream_gene_variant	MODIFIER	Dgkq	ENSMUSG00000004815	Transcript	ENSMUST00000139169	retained_intron	rs29717159
5:108653865-108653865	downstream_gene_variant	MODIFIER	Dgkq	ENSMUSG00000004815	Transcript	ENSMUST00000139598	retained_intron	rs29717159
5:108653865-108653865	intron_variant,non_coding_trans	MODIFIER	Dgkq	ENSMUSG00000004815	Transcript	ENSMUST00000144624	retained_intron	rs29717159
5:108653865-108653865	upstream_gene_variant	MODIFIER	Dgkq	ENSMUSG00000004815	Transcript	ENSMUST00000145917	retained_intron	rs29717159
5:108653865-108653865	intron_variant,NMD_transcript_v	MODIFIER	Dgkq	ENSMUSG00000004815	Transcript	ENSMUST00000153238	nonsense_mediated_decay	rs29717159
5:108653865-108653865	intron_variant,non_coding_trans	MODIFIER	Dgkq	ENSMUSG00000004815	Transcript	ENSMUST00000153365	retained_intron	rs29717159
5:108653865-108653865	intron_variant,non_coding_trans	MODIFIER	Dgkq	ENSMUSG00000004815	Transcript	ENSMUST00000156964	retained_intron	rs29717159
5:108681188-108681188	intron_variant	MODIFIER	Idua	ENSMUSG00000033540	Transcript	ENSMUST00000071650	protein_coding	rs45680768
5:108681188-108681188	intron_variant	MODIFIER	Idua	ENSMUSG00000033540	Transcript	ENSMUST00000112563	protein_coding	rs45680768
5:108681188-108681188	intron_variant	MODIFIER	Idua	ENSMUSG00000033540	Transcript	ENSMUST00000119212	protein_coding	rs45680768
5:108681188-108681188	upstream_gene_variant	MODIFIER	Idua	ENSMUSG00000033540	Transcript	ENSMUST00000133482	retained_intron	rs45680768
5:108681188-108681188	intron_variant,NMD_transcript_v	MODIFIER	Idua	ENSMUSG00000033540	Transcript	ENSMUST00000139734	nonsense_mediated_decay	rs45680768
5:108681188-108681188	3_prime_UTR_variant,NMD_trar	MODIFIER	Idua	ENSMUSG00000033540	Transcript	ENSMUST00000140620	nonsense_mediated_decay	rs45680768
5:108681188-108681188	intron_variant,non_coding_trans	MODIFIER	Idua	ENSMUSG00000033540	Transcript	ENSMUST00000151445	retained_intron	rs45680768
5:108681188-108681188	downstream_gene_variant	MODIFIER	Idua	ENSMUSG00000033540	Transcript	ENSMUST00000159464	processed_transcript	rs45680768
5:109549976-109549976	downstream_gene_variant	MODIFIER	Crlf2	ENSMUSG00000033467	Transcript	ENSMUST00000044579	protein_coding	rs29506578
5:109549976-109549976	downstream_gene_variant	MODIFIER	Gm8493	ENSMUSG00000106028	Transcript	ENSMUST00000196881	unprocessed_pseudogene	rs29506578
5:109549976-109549976	downstream_gene_variant	MODIFIER	Crlf2	ENSMUSG00000033467	Transcript	ENSMUST00000198960	protein_coding	rs29506578
5:109549976-109549976	downstream_gene_variant	MODIFIER	Crlf2	ENSMUSG00000033467	Transcript	ENSMUST00000200284	protein_coding	rs29506578
5:109550413-109550413	downstream_gene_variant	MODIFIER	Crlf2	ENSMUSG00000033467	Transcript	ENSMUST00000044579	protein_coding	rs241696506
5:109550413-109550413	downstream_gene_variant	MODIFIER	Gm8493	ENSMUSG00000106028	Transcript	ENSMUST00000196881	unprocessed_pseudogene	rs241696506
5:109550413-109550413	downstream_gene_variant	MODIFIER	Crlf2	ENSMUSG00000033467	Transcript	ENSMUST00000198960	protein_coding	rs241696506
5:109550413-109550413	downstream_gene_variant	MODIFIER	Crlf2	ENSMUSG00000033467	Transcript	ENSMUST00000200284	protein_coding	rs241696506
5:109550430-109550430	downstream_gene_variant	MODIFIER	Crlf2	ENSMUSG00000033467	Transcript	ENSMUST00000044579	protein_coding	rs47639059
5:109550430-109550430	downstream_gene_variant	MODIFIER	Gm8493	ENSMUSG00000106028	Transcript	ENSMUST00000196881	unprocessed_pseudogene	rs47639059
5:109550430-109550430	downstream_gene_variant	MODIFIER	Crlf2	ENSMUSG00000033467	Transcript	ENSMUST00000198960	protein_coding	rs47639059
5:109550430-109550430	downstream_gene_variant	MODIFIER	Crlf2	ENSMUSG00000033467	Transcript	ENSMUST00000200284	protein_coding	rs47639059
5:109553083-109553083	downstream_gene_variant	MODIFIER	Crlf2	ENSMUSG00000033467	Transcript	ENSMUST00000044579	protein_coding	rs226152806
5:109553083-109553083	non_coding_transcript_exon_var	MODIFIER	Gm8493	ENSMUSG00000106028	Transcript	ENSMUST00000196881	unprocessed_pseudogene	rs226152806
5:109553083-109553083	downstream_gene_variant	MODIFIER	Crlf2	ENSMUSG00000033467	Transcript	ENSMUST00000198960	protein_coding	rs226152806
5:109553083-109553083	downstream_gene_variant	MODIFIER	Crlf2	ENSMUSG00000033467	Transcript	ENSMUST00000200284	protein_coding	rs226152806
5:109558102-109558102	intron_variant	MODIFIER	Crlf2	ENSMUSG00000033467	Transcript	ENSMUST00000044579	protein_coding	rs108144015
5:109558102-109558102	upstream_gene_variant	MODIFIER	Gm8493	ENSMUSG00000106028	Transcript	ENSMUST00000196881	unprocessed_pseudogene	rs108144015
5:109558102-109558102	upstream_gene_variant	MODIFIER	Crlf2	ENSMUSG00000033467	Transcript	ENSMUST00000198960	protein_coding	rs108144015
5:109558102-109558102	intron_variant	MODIFIER	Crlf2	ENSMUSG00000033467	Transcript	ENSMUST00000200284	protein_coding	rs108144015
5:109558102-109558102	regulatory_region_variant	MODIFIER	-	-	RegulatoryFeature	ENSMUSR00000215036	promoter	rs108144015
5:109558144-109558144	intron_variant	MODIFIER	Crlf2	ENSMUSG00000033467	Transcript	ENSMUST00000044579	protein_coding	rs107637773
5:109558144-109558144	upstream_gene_variant	MODIFIER	Gm8493	ENSMUSG00000106028	Transcript	ENSMUST00000196881	unprocessed_pseudogene	rs107637773
5:109558144-109558144	upstream_gene_variant	MODIFIER	Crlf2	ENSMUSG00000033467	Transcript	ENSMUST00000198960	protein_coding	rs107637773
5:109558144-109558144	intron_variant	MODIFIER	Crlf2	ENSMUSG00000033467	Transcript	ENSMUST00000200284	protein_coding	rs107637773
5:109558144-109558144	regulatory_region_variant	MODIFIER	-	-	RegulatoryFeature	ENSMUSR00000215036	promoter	rs107637773
5:109736203-109736203	downstream_gene_variant	MODIFIER	4930522L14Rik	ENSMUSG00000072762	Transcript	ENSMUST00000100937	protein_coding	rs33578475
5:109736203-109736203	missense_variant	MODERATE	4930522L14Rik	ENSMUSG00000072762	Transcript	ENSMUST00000112547	protein_coding	rs33578475
5:109888137-109888137	intron_variant,non_coding_trans	MODIFIER	Gm26779	ENSMUSG00000097140	Transcript	ENSMUST00000181101	lincRNA	rs33547875
5:109888141-109888141	intron_variant,non_coding_trans	MODIFIER	Gm26779	ENSMUSG00000097140	Transcript	ENSMUST00000181101	lincRNA	rs33054373
5:109938534-109938534	intron_variant	MODIFIER	Gm15446	ENSMUSG00000090015	Transcript	ENSMUST00000112544	protein_coding	rs29549894
5:109938534-109938534	intron_variant,non_coding_trans	MODIFIER	Gm15446	ENSMUSG00000090015	Transcript	ENSMUST00000146564	retained_intron	rs29549894
5:109938534-109938534	upstream_gene_variant	MODIFIER	Gm15446	ENSMUSG00000090015	Transcript	ENSMUST00000170826	protein_coding	rs29549894
5:110206505-110206505	intron_variant	MODIFIER	Golga3	ENSMUSG00000029502	Transcript	ENSMUST00000031477	protein_coding	rs33145160
5:110206505-110206505	intron_variant	MODIFIER	Golga3	ENSMUSG00000029502	Transcript	ENSMUST00000112512	protein_coding	rs33145160
5:110206505-110206505	upstream_gene_variant	MODIFIER	Gm43138	ENSMUSG00000104720	Transcript	ENSMUST00000199123	sense_intronic	rs33145160
5:110223382-110223382	downstream_gene_variant	MODIFIER	Golga3	ENSMUSG00000029502	Transcript	ENSMUST00000031477	protein_coding	rs228789455
5:110223382-110223382	3_prime_UTR_variant	MODIFIER	Golga3	ENSMUSG00000029502	Transcript	ENSMUST00000112512	protein_coding	rs228789455
5:110252610-110252610	intron_variant	MODIFIER	Ankle2	ENSMUSG00000029501	Transcript	ENSMUST00000031474	protein_coding	rs45815461
5:110252610-110252610	intron_variant	MODIFIER	Ankle2	ENSMUSG00000029501	Transcript	ENSMUST00000086674	protein_coding	rs45815461
5:110252610-110252610	intron_variant,non_coding_trans	MODIFIER	Ankle2	ENSMUSG00000029501	Transcript	ENSMUST00000196821	processed_transcript	rs45815461
5:110252610-110252610	intron_variant	MODIFIER	Ankle2	ENSMUSG00000029501	Transcript	ENSMUST00000197188	protein_coding	rs45815461

5:110256681-110256681	downstream_gene_variant	MODIFIER	Ankle2	ENSMUSG00000029501	Transcript	ENSMUST00000031474	protein_coding	rs33244440
5:110256681-110256681	downstream_gene_variant	MODIFIER	Pgam5	ENSMUSG00000029500	Transcript	ENSMUST00000059229	protein_coding	rs33244440
5:110256681-110256681	downstream_gene_variant	MODIFIER	Ankle2	ENSMUSG00000029501	Transcript	ENSMUST00000086674	protein_coding	rs33244440
5:110256681-110256681	downstream_gene_variant	MODIFIER	Pgam5	ENSMUSG00000029500	Transcript	ENSMUST00000112505	protein_coding	rs33244440
5:110256681-110256681	upstream_gene_variant	MODIFIER	Gm26718	ENSMUSG00000097367	Transcript	ENSMUST00000181048	lincRNA	rs33244440
5:110256681-110256681	downstream_gene_variant	MODIFIER	Ankle2	ENSMUSG00000029501	Transcript	ENSMUST00000196821	processed_transcript	rs33244440
5:110256681-110256681	downstream_gene_variant	MODIFIER	Ankle2	ENSMUSG00000029501	Transcript	ENSMUST00000197188	protein_coding	rs33244440
5:110256682-110256682	downstream_gene_variant	MODIFIER	Ankle2	ENSMUSG00000029501	Transcript	ENSMUST00000031474	protein_coding	rs33559112
5:110256682-110256682	downstream_gene_variant	MODIFIER	Pgam5	ENSMUSG00000029500	Transcript	ENSMUST00000059229	protein_coding	rs33559112
5:110256682-110256682	downstream_gene_variant	MODIFIER	Ankle2	ENSMUSG00000029501	Transcript	ENSMUST00000086674	protein_coding	rs33559112
5:110256682-110256682	downstream_gene_variant	MODIFIER	Pgam5	ENSMUSG00000029500	Transcript	ENSMUST00000112505	protein_coding	rs33559112
5:110256682-110256682	upstream_gene_variant	MODIFIER	Gm26718	ENSMUSG00000097367	Transcript	ENSMUST00000181048	lincRNA	rs33559112
5:110256682-110256682	downstream_gene_variant	MODIFIER	Ankle2	ENSMUSG00000029501	Transcript	ENSMUST00000196821	processed_transcript	rs33559112
5:110256682-110256682	downstream_gene_variant	MODIFIER	Ankle2	ENSMUSG00000029501	Transcript	ENSMUST00000197188	protein_coding	rs33559112
5:110256956-110256956	downstream_gene_variant	MODIFIER	Ankle2	ENSMUSG00000029501	Transcript	ENSMUST00000031474	protein_coding	rs259706371
5:110256956-110256956	downstream_gene_variant	MODIFIER	Pgam5	ENSMUSG00000029500	Transcript	ENSMUST00000059229	protein_coding	rs259706371
5:110256956-110256956	downstream_gene_variant	MODIFIER	Ankle2	ENSMUSG00000029501	Transcript	ENSMUST00000086674	protein_coding	rs259706371
5:110256956-110256956	downstream_gene_variant	MODIFIER	Pgam5	ENSMUSG00000029500	Transcript	ENSMUST00000112505	protein_coding	rs259706371
5:110256956-110256956	upstream_gene_variant	MODIFIER	Gm26718	ENSMUSG00000097367	Transcript	ENSMUST00000181048	lincRNA	rs259706371
5:110256956-110256956	downstream_gene_variant	MODIFIER	Ankle2	ENSMUSG00000029501	Transcript	ENSMUST00000196821	processed_transcript	rs259706371
5:110256956-110256956	downstream_gene_variant	MODIFIER	Ankle2	ENSMUSG00000029501	Transcript	ENSMUST00000197188	protein_coding	rs259706371
5:110259129-110259129	downstream_gene_variant	MODIFIER	Ankle2	ENSMUSG00000029501	Transcript	ENSMUST00000031474	protein_coding	rs220792105
5:110259129-110259129	downstream_gene_variant	MODIFIER	Pgam5	ENSMUSG00000029500	Transcript	ENSMUST00000059229	protein_coding	rs220792105
5:110259129-110259129	downstream_gene_variant	MODIFIER	Ankle2	ENSMUSG00000029501	Transcript	ENSMUST00000086674	protein_coding	rs220792105
5:110259129-110259129	downstream_gene_variant	MODIFIER	Pgam5	ENSMUSG00000029500	Transcript	ENSMUST00000112505	protein_coding	rs220792105
5:110259129-110259129	upstream_gene_variant	MODIFIER	Gm15788	ENSMUSG00000087439	Transcript	ENSMUST00000130123	antisense	rs220792105
5:110259129-110259129	upstream_gene_variant	MODIFIER	Gm15788	ENSMUSG00000087439	Transcript	ENSMUST00000140087	antisense	rs220792105
5:110259129-110259129	upstream_gene_variant	MODIFIER	Gm26718	ENSMUSG00000097367	Transcript	ENSMUST00000181048	lincRNA	rs220792105
5:110259129-110259129	downstream_gene_variant	MODIFIER	Ankle2	ENSMUSG00000029501	Transcript	ENSMUST00000197188	protein_coding	rs220792105
5:110371988-110371988	intron_variant	MODIFIER	Fbrsl1	ENSMUSG00000043323	Transcript	ENSMUST00000056124	protein_coding	rs33483783
5:110371988-110371988	intron_variant	MODIFIER	Fbrsl1	ENSMUSG00000043323	Transcript	ENSMUST00000069483	protein_coding	rs33483783
5:110371988-110371988	intron_variant,NMD_transcript_v	MODIFIER	Fbrsl1	ENSMUSG00000043323	Transcript	ENSMUST00000196801	nonsense_mediated_decay	rs33483783
5:110371988-110371988	intron_variant	MODIFIER	Fbrsl1	ENSMUSG00000043323	Transcript	ENSMUST00000198834	protein_coding	rs33483783
5:110387039-110387039	5_prime_UTR_variant	MODIFIER	Fbrsl1	ENSMUSG00000043323	Transcript	ENSMUST00000056124	protein_coding	rs33753644
5:110387039-110387039	intron_variant	MODIFIER	Fbrsl1	ENSMUSG00000043323	Transcript	ENSMUST00000069483	protein_coding	rs33753644
5:110387039-110387039	non_coding_transcript_exon_var	MODIFIER	Gm26711	ENSMUSG00000097904	Transcript	ENSMUST00000180866	lincRNA	rs33753644
5:110387039-110387039	intron_variant,NMD_transcript_v	MODIFIER	Fbrsl1	ENSMUSG00000043323	Transcript	ENSMUST00000196801	nonsense_mediated_decay	rs33753644
5:110387039-110387039	intron_variant	MODIFIER	Fbrsl1	ENSMUSG00000043323	Transcript	ENSMUST00000198768	protein_coding	rs33753644
5:110387039-110387039	upstream_gene_variant	MODIFIER	Fbrsl1	ENSMUSG00000043323	Transcript	ENSMUST00000198834	protein_coding	rs33753644
5:110387039-110387039	regulatory_region_variant	MODIFIER	-	-	RegulatoryFeature	ENSMUSR00000215125	promoter	rs33753644
5:112304966-112304966	intron_variant	MODIFIER	Tpst2	ENSMUSG00000029344	Transcript	ENSMUST00000031287	protein_coding	rs211954909
5:112304966-112304966	intron_variant	MODIFIER	Tpst2	ENSMUSG00000029344	Transcript	ENSMUST00000071455	protein_coding	rs211954909
5:112304966-112304966	intron_variant,non_coding_trans	MODIFIER	Tpst2	ENSMUSG00000029344	Transcript	ENSMUST00000134071	retained_intron	rs211954909
5:112304966-112304966	upstream_gene_variant	MODIFIER	Tpst2	ENSMUSG00000029344	Transcript	ENSMUST00000140262	nonsense_mediated_decay	rs211954909
5:112304966-112304966	intron_variant	MODIFIER	Tpst2	ENSMUSG00000029344	Transcript	ENSMUST00000151947	protein_coding	rs211954909
5:112304966-112304966	intron_variant	MODIFIER	Tpst2	ENSMUSG00000029344	Transcript	ENSMUST00000198502	protein_coding	rs211954909
5:112689017-112689019	3_prime_UTR_variant	MODIFIER	Myo18b	ENSMUSG00000072720	Transcript	ENSMUST00000086617	protein_coding	rs387518323
5:112689017-112689019	non_coding_transcript_exon_var	MODIFIER	Myo18b	ENSMUSG00000072720	Transcript	ENSMUST00000182189	processed_transcript	rs387518323
5:112770067-112770067	intron_variant	MODIFIER	Myo18b	ENSMUSG00000072720	Transcript	ENSMUST00000086617	protein_coding	rs29563158
5:112770067-112770067	upstream_gene_variant	MODIFIER	Myo18b	ENSMUSG00000072720	Transcript	ENSMUST00000182189	processed_transcript	rs29563158
5:112770067-112770067	downstream_gene_variant	MODIFIER	Myo18b	ENSMUSG00000072720	Transcript	ENSMUST00000183029	processed_transcript	rs29563158
5:112770067-112770067	regulatory_region_variant	MODIFIER	-	-	RegulatoryFeature	ENSMUSR00000686922	promoter_flanking_region	rs29563158
5:112809682-112809682	missense_variant	MODERATE	Myo18b	ENSMUSG00000072720	Transcript	ENSMUST00000086617	protein_coding	rs46076017
5:112809682-112809682	non_coding_transcript_exon_var	MODIFIER	Myo18b	ENSMUSG00000072720	Transcript	ENSMUST00000183029	processed_transcript	rs46076017
5:112817677-112817677	missense_variant	MODERATE	Myo18b	ENSMUSG00000072720	Transcript	ENSMUST00000086617	protein_coding	rs45870467
5:112817677-112817677	non_coding_transcript_exon_var	MODIFIER	Myo18b	ENSMUSG00000072720	Transcript	ENSMUST00000183029	processed_transcript	rs45870467
5:112817677-112817677	3_prime_UTR_variant,NMD_tran	MODIFIER	Myo18b	ENSMUSG00000072720	Transcript	ENSMUST00000183273	nonsense_mediated_decay	rs45870467
5:113847912-113847912	intron_variant	MODIFIER	Coro1c	ENSMUSG00000004530	Transcript	ENSMUST00000004646	protein_coding	rs29813178
5:113847912-113847912	downstream_gene_variant	MODIFIER	Coro1c	ENSMUSG00000004530	Transcript	ENSMUST00000111283	retained_intron	rs29813178
5:113847912-113847912	intron_variant,non_coding_trans	MODIFIER	Coro1c	ENSMUSG00000004530	Transcript	ENSMUST00000163995	retained_intron	rs29813178
5:113847912-113847912	intron_variant,NMD_transcript_v	MODIFIER	Coro1c	ENSMUSG00000004530	Transcript	ENSMUST00000164980	nonsense_mediated_decay	rs29813178
5:113847912-113847912	downstream_gene_variant	MODIFIER	Gm17122	ENSMUSG00000090458	Transcript	ENSMUST00000166047	antisense	rs29813178
5:113847912-113847912	downstream_gene_variant	MODIFIER	Coro1c	ENSMUSG00000004530	Transcript	ENSMUST00000166647	processed_transcript	rs29813178
5:113847912-113847912	downstream_gene_variant	MODIFIER	Coro1c	ENSMUSG00000004530	Transcript	ENSMUST00000168493	processed_transcript	rs29813178
5:113847912-113847912	intron_variant,non_coding_trans	MODIFIER	Coro1c	ENSMUSG00000004530	Transcript	ENSMUST00000168634	retained_intron	rs29813178
5:113847912-113847912	upstream_gene_variant	MODIFIER	Coro1c	ENSMUSG00000004530	Transcript	ENSMUST00000172016	retained_intron	rs29813178
5:114372651-114372651	intron_variant	MODIFIER	Kctd10	ENSMUSG00000001098	Transcript	ENSMUST00000001125	protein_coding	rs29778625
5:114372651-114372651	intron_variant	MODIFIER	Kctd10	ENSMUSG00000001098	Transcript	ENSMUST00000102581	protein_coding	rs29778625
5:114372651-114372651	upstream_gene_variant	MODIFIER	Kctd10	ENSMUSG00000001098	Transcript	ENSMUST00000123538	retained_intron	rs29778625

5:114372651-114372651	upstream_gene_variant	MODIFIER	Kctd10	ENSMUSG00000001098	Transcript	ENSMUST00000132646	retained_intron	rs29778625
5:114372651-114372651	upstream_gene_variant	MODIFIER	Kctd10	ENSMUSG00000001098	Transcript	ENSMUST00000134173	processed_transcript	rs29778625
5:114372651-114372651	intron_variant,NMD_transcript_v	MODIFIER	Kctd10	ENSMUSG00000001098	Transcript	ENSMUST00000134532	nonsense_mediated_decay	rs29778625
5:114372651-114372651	upstream_gene_variant	MODIFIER	Kctd10	ENSMUSG00000001098	Transcript	ENSMUST00000135170	retained_intron	rs29778625
5:125020237-125020237	intron_variant	MODIFIER	Ncor2	ENSMUSG00000029478	Transcript	ENSMUST00000055256	protein_coding	rs33273829
5:125020237-125020237	intron_variant	MODIFIER	Ncor2	ENSMUSG00000029478	Transcript	ENSMUST00000086083	protein_coding	rs33273829
5:125020237-125020237	intron_variant	MODIFIER	Ncor2	ENSMUSG00000029478	Transcript	ENSMUST00000111393	protein_coding	rs33273829
5:125020237-125020237	intron_variant	MODIFIER	Ncor2	ENSMUSG00000029478	Transcript	ENSMUST00000111394	protein_coding	rs33273829
5:125020237-125020237	intron_variant	MODIFIER	Ncor2	ENSMUSG00000029478	Transcript	ENSMUST00000111398	protein_coding	rs33273829
5:125020237-125020237	intron_variant	MODIFIER	Ncor2	ENSMUSG00000029478	Transcript	ENSMUST00000111402	protein_coding	rs33273829
5:125020237-125020237	intron_variant	MODIFIER	Ncor2	ENSMUSG00000029478	Transcript	ENSMUST00000125053	protein_coding	rs33273829
5:125020237-125020237	downstream_gene_variant	MODIFIER	Ncor2	ENSMUSG00000029478	Transcript	ENSMUST00000130742	retained_intron	rs33273829
5:125020237-125020237	downstream_gene_variant	MODIFIER	Ncor2	ENSMUSG00000029478	Transcript	ENSMUST00000134819	protein_coding	rs33273829
5:125020237-125020237	intron_variant	MODIFIER	Ncor2	ENSMUSG00000029478	Transcript	ENSMUST00000138890	protein_coding	rs33273829
5:125020237-125020237	intron_variant,non_coding_trans	MODIFIER	Ncor2	ENSMUSG00000029478	Transcript	ENSMUST00000144354	retained_intron	rs33273829
5:125020237-125020237	intron_variant,NMD_transcript_v	MODIFIER	Ncor2	ENSMUSG00000029478	Transcript	ENSMUST00000200297	nonsense_mediated_decay	rs33273829
5:125286080-125286080	intron_variant	MODIFIER	Scarb1	ENSMUSG00000037936	Transcript	ENSMUST00000086075	protein_coding	rs3656810
5:125286080-125286080	intron_variant	MODIFIER	Scarb1	ENSMUSG00000037936	Transcript	ENSMUST00000111390	protein_coding	rs3656810
5:125286080-125286080	upstream_gene_variant	MODIFIER	Scarb1	ENSMUSG00000037936	Transcript	ENSMUST00000124582	processed_transcript	rs3656810
5:125286080-125286080	intron_variant	MODIFIER	Scarb1	ENSMUSG00000037936	Transcript	ENSMUST00000127148	protein_coding	rs3656810
5:125286080-125286080	non_coding_transcript_exon_va	MODIFIER	Scarb1	ENSMUSG00000037936	Transcript	ENSMUST00000135736	retained_intron	rs3656810
5:125286080-125286080	intron_variant,non_coding_trans	MODIFIER	Scarb1	ENSMUSG00000037936	Transcript	ENSMUST00000148373	retained_intron	rs3656810
5:125286080-125286080	regulatory_region_variant	MODIFIER	-	-	RegulatoryFeature	ENSMUSR00000218027	open_chromatin_region	rs3656810
5:125456906-125456906	3_prime_UTR_variant	MODIFIER	Bri3bp	ENSMUSG00000037905	Transcript	ENSMUST00000049040	protein_coding	-
5:125456906-125456906	downstream_gene_variant	MODIFIER	Bri3bp	ENSMUSG00000037905	Transcript	ENSMUST00000198811	protein_coding	-
5:125456927-125456927	3_prime_UTR_variant	MODIFIER	Bri3bp	ENSMUSG00000037905	Transcript	ENSMUST00000049040	protein_coding	-
5:125456927-125456927	downstream_gene_variant	MODIFIER	Bri3bp	ENSMUSG00000037905	Transcript	ENSMUST00000198811	protein_coding	-
5:127579029-127579029	non_coding_transcript_exon_va	MODIFIER	Gm42980	ENSMUSG00000106015	Transcript	ENSMUST00000200582	lincRNA	rs29515128
5:127590131-127590131	intergenic_variant	MODIFIER	-	-	-	-	-	rs29774442
5:127601606-127601606	intron_variant	MODIFIER	Slc15a4	ENSMUSG00000029416	Transcript	ENSMUST00000031367	protein_coding	rs33164285
5:127601606-127601606	intron_variant,NMD_transcript_v	MODIFIER	Slc15a4	ENSMUSG00000029416	Transcript	ENSMUST00000124569	nonsense_mediated_decay	rs33164285
5:127601606-127601606	downstream_gene_variant	MODIFIER	Slc15a4	ENSMUSG00000029416	Transcript	ENSMUST00000144603	nonsense_mediated_decay	rs33164285
5:127601606-127601606	downstream_gene_variant	MODIFIER	Slc15a4	ENSMUSG00000029416	Transcript	ENSMUST00000152727	protein_coding	rs33164285
5:127601606-127601606	intron_variant,NMD_transcript_v	MODIFIER	Slc15a4	ENSMUSG00000029416	Transcript	ENSMUST00000153832	nonsense_mediated_decay	rs33164285
5:127601606-127601606	intron_variant	MODIFIER	Slc15a4	ENSMUSG00000029416	Transcript	ENSMUST00000198486	protein_coding	rs33164285
5:127601606-127601606	downstream_gene_variant	MODIFIER	Slc15a4	ENSMUSG00000029416	Transcript	ENSMUST00000198727	processed_transcript	rs33164285
5:127601606-127601606	downstream_gene_variant	MODIFIER	Slc15a4	ENSMUSG00000029416	Transcript	ENSMUST00000199810	processed_transcript	rs33164285
5:127601606-127601606	downstream_gene_variant	MODIFIER	Slc15a4	ENSMUSG00000029416	Transcript	ENSMUST00000200212	processed_transcript	rs33164285
5:127607631-127607631	intron_variant	MODIFIER	Slc15a4	ENSMUSG00000029416	Transcript	ENSMUST00000031367	protein_coding	rs29522873
5:127607631-127607631	intron_variant,NMD_transcript_v	MODIFIER	Slc15a4	ENSMUSG00000029416	Transcript	ENSMUST00000124569	nonsense_mediated_decay	rs29522873
5:127607631-127607631	intron_variant,NMD_transcript_v	MODIFIER	Slc15a4	ENSMUSG00000029416	Transcript	ENSMUST00000144603	nonsense_mediated_decay	rs29522873
5:127607631-127607631	intron_variant	MODIFIER	Slc15a4	ENSMUSG00000029416	Transcript	ENSMUST00000152727	protein_coding	rs29522873
5:127607631-127607631	intron_variant,NMD_transcript_v	MODIFIER	Slc15a4	ENSMUSG00000029416	Transcript	ENSMUST00000153832	nonsense_mediated_decay	rs29522873
5:127607631-127607631	downstream_gene_variant	MODIFIER	Slc15a4	ENSMUSG00000029416	Transcript	ENSMUST00000155321	protein_coding	rs29522873
5:127607631-127607631	upstream_gene_variant	MODIFIER	Slc15a4	ENSMUSG00000029416	Transcript	ENSMUST00000198486	protein_coding	rs29522873
5:127607631-127607631	intron_variant,non_coding_trans	MODIFIER	Slc15a4	ENSMUSG00000029416	Transcript	ENSMUST00000198727	processed_transcript	rs29522873
5:127607631-127607631	upstream_gene_variant	MODIFIER	Slc15a4	ENSMUSG00000029416	Transcript	ENSMUST00000199810	processed_transcript	rs29522873
5:127607631-127607631	upstream_gene_variant	MODIFIER	Slc15a4	ENSMUSG00000029416	Transcript	ENSMUST00000200212	processed_transcript	rs29522873
5:127610580-127610580	intron_variant	MODIFIER	Slc15a4	ENSMUSG00000029416	Transcript	ENSMUST00000031367	protein_coding	rs29678741
5:127610580-127610580	intron_variant,NMD_transcript_v	MODIFIER	Slc15a4	ENSMUSG00000029416	Transcript	ENSMUST00000124569	nonsense_mediated_decay	rs29678741
5:127610580-127610580	intron_variant,NMD_transcript_v	MODIFIER	Slc15a4	ENSMUSG00000029416	Transcript	ENSMUST00000144603	nonsense_mediated_decay	rs29678741
5:127610580-127610580	upstream_gene_variant	MODIFIER	Slc15a4	ENSMUSG00000029416	Transcript	ENSMUST00000152727	protein_coding	rs29678741
5:127610580-127610580	intron_variant,NMD_transcript_v	MODIFIER	Slc15a4	ENSMUSG00000029416	Transcript	ENSMUST00000153832	nonsense_mediated_decay	rs29678741
5:127610580-127610580	intron_variant	MODIFIER	Slc15a4	ENSMUSG00000029416	Transcript	ENSMUST00000155321	protein_coding	rs29678741
5:127610580-127610580	downstream_gene_variant	MODIFIER	Slc15a4	ENSMUSG00000029416	Transcript	ENSMUST00000182841	retained_intron	rs29678741
5:127610580-127610580	intron_variant,non_coding_trans	MODIFIER	Slc15a4	ENSMUSG00000029416	Transcript	ENSMUST00000198727	processed_transcript	rs29678741
5:147306965-147306965	synonymous_variant	LOW	Cdx2	ENSMUSG00000029646	Transcript	ENSMUST00000031650	protein_coding	-
5:147306965-147306965	regulatory_region_variant	MODIFIER	-	-	RegulatoryFeature	ENSMUSR00000693977	open_chromatin_region	-
5:38122706-38122706	intron_variant	MODIFIER	Stx18	ENSMUSG00000029125	Transcript	ENSMUST00000031008	protein_coding	rs29681339
5:38122706-38122706	intron_variant	MODIFIER	Stx18	ENSMUSG00000029125	Transcript	ENSMUST00000042146	protein_coding	rs29681339
5:38122706-38122706	intron_variant	MODIFIER	Stx18	ENSMUSG00000029125	Transcript	ENSMUST00000114126	protein_coding	rs29681339
5:38122706-38122706	intron_variant	MODIFIER	Stx18	ENSMUSG00000029125	Transcript	ENSMUST00000146864	protein_coding	rs29681339
5:38122706-38122706	intron_variant,non_coding_trans	MODIFIER	Stx18	ENSMUSG00000029125	Transcript	ENSMUST00000147667	retained_intron	rs29681339
5:38122706-38122706	intron_variant	MODIFIER	Stx18	ENSMUSG00000029125	Transcript	ENSMUST00000154929	protein_coding	rs29681339
5:38122706-38122706	downstream_gene_variant	MODIFIER	Stx18	ENSMUSG00000029125	Transcript	ENSMUST00000201285	processed_transcript	rs29681339
5:38125356-38125356	intron_variant	MODIFIER	Stx18	ENSMUSG00000029125	Transcript	ENSMUST00000031008	protein_coding	rs33375845
5:38125356-38125356	intron_variant	MODIFIER	Stx18	ENSMUSG00000029125	Transcript	ENSMUST00000042146	protein_coding	rs33375845
5:38125356-38125356	intron_variant	MODIFIER	Stx18	ENSMUSG00000029125	Transcript	ENSMUST00000114126	protein_coding	rs33375845
5:38125356-38125356	intron_variant	MODIFIER	Stx18	ENSMUSG00000029125	Transcript	ENSMUST00000146864	protein_coding	rs33375845

5:38125356-38125356	intron_variant,non_coding_trans	MODIFIER	Stx18	ENSMUSG00000029125	Transcript	ENSMUST00000147667	retained_intron	rs33375845
5:38125356-38125356	intron_variant	MODIFIER	Stx18	ENSMUSG00000029125	Transcript	ENSMUST00000154929	protein_coding	rs33375845
5:38125356-38125356	downstream_gene_variant	MODIFIER	Stx18	ENSMUSG00000029125	Transcript	ENSMUST00000201285	processed_transcript	rs33375845
5:38211303-38211303	intron_variant	MODIFIER	Zbtb49	ENSMUSG00000029127	Transcript	ENSMUST00000094833	protein_coding	rs29542903
5:38211303-38211303	downstream_gene_variant	MODIFIER	Zbtb49	ENSMUSG00000029127	Transcript	ENSMUST00000114113	protein_coding	rs29542903
5:38211303-38211303	intron_variant,NMD_transcript_v	MODIFIER	Zbtb49	ENSMUSG00000029127	Transcript	ENSMUST00000123106	nonsense_mediated_decay	rs29542903
5:38211303-38211303	intron_variant,NMD_transcript_v	MODIFIER	Zbtb49	ENSMUSG00000029127	Transcript	ENSMUST00000126267	nonsense_mediated_decay	rs29542903
5:38211303-38211303	intron_variant,NMD_transcript_v	MODIFIER	Zbtb49	ENSMUSG00000029127	Transcript	ENSMUST00000129161	nonsense_mediated_decay	rs29542903
5:38211303-38211303	intron_variant,NMD_transcript_v	MODIFIER	Zbtb49	ENSMUSG00000029127	Transcript	ENSMUST00000136475	nonsense_mediated_decay	rs29542903
5:38211303-38211303	downstream_gene_variant	MODIFIER	Zbtb49	ENSMUSG00000029127	Transcript	ENSMUST00000137577	retained_intron	rs29542903
5:38211303-38211303	intron_variant,NMD_transcript_v	MODIFIER	Zbtb49	ENSMUSG00000029127	Transcript	ENSMUST00000138820	nonsense_mediated_decay	rs29542903
5:38211303-38211303	intron_variant,NMD_transcript_v	MODIFIER	Zbtb49	ENSMUSG00000029127	Transcript	ENSMUST00000143436	nonsense_mediated_decay	rs29542903
5:38268113-38268113	intron_variant	MODIFIER	Tmem128	ENSMUSG00000067365	Transcript	ENSMUST00000087511	protein_coding	rs37556015
5:38268113-38268113	downstream_gene_variant	MODIFIER	Tmem128	ENSMUSG00000067365	Transcript	ENSMUST00000119047	protein_coding	rs37556015
5:38268113-38268113	downstream_gene_variant	MODIFIER	Tmem128	ENSMUSG00000067365	Transcript	ENSMUST00000128025	retained_intron	rs37556015
5:38268113-38268113	downstream_gene_variant	MODIFIER	Tmem128	ENSMUSG00000067365	Transcript	ENSMUST00000142277	protein_coding	rs37556015
5:38268141-38268141	intron_variant	MODIFIER	Tmem128	ENSMUSG00000067365	Transcript	ENSMUST00000087511	protein_coding	rs46052468
5:38268141-38268141	downstream_gene_variant	MODIFIER	Tmem128	ENSMUSG00000067365	Transcript	ENSMUST00000119047	protein_coding	rs46052468
5:38268141-38268141	downstream_gene_variant	MODIFIER	Tmem128	ENSMUSG00000067365	Transcript	ENSMUST00000128025	retained_intron	rs46052468
5:38268141-38268141	downstream_gene_variant	MODIFIER	Tmem128	ENSMUSG00000067365	Transcript	ENSMUST00000142277	protein_coding	rs46052468
5:38351828-38351828	intron_variant	MODIFIER	Slc2a9	ENSMUSG00000005107	Transcript	ENSMUST00000005238	protein_coding	rs36839087
5:38351828-38351828	intron_variant	MODIFIER	Slc2a9	ENSMUSG00000005107	Transcript	ENSMUST00000067872	protein_coding	rs36839087
5:38351828-38351828	intron_variant	MODIFIER	Slc2a9	ENSMUSG00000005107	Transcript	ENSMUST00000067886	protein_coding	rs36839087
5:38351828-38351828	intron_variant,NMD_transcript_v	MODIFIER	Slc2a9	ENSMUSG00000005107	Transcript	ENSMUST00000122970	nonsense_mediated_decay	rs36839087
5:38351828-38351828	intron_variant	MODIFIER	Slc2a9	ENSMUSG00000005107	Transcript	ENSMUST00000129099	protein_coding	rs36839087
5:38351828-38351828	intron_variant	MODIFIER	Slc2a9	ENSMUSG00000005107	Transcript	ENSMUST00000143758	protein_coding	rs36839087
5:38351828-38351828	intron_variant	MODIFIER	Slc2a9	ENSMUSG00000005107	Transcript	ENSMUST00000155634	protein_coding	rs36839087
5:38351828-38351828	downstream_gene_variant	MODIFIER	4930487D11Rik	ENSMUSG00000106932	Transcript	ENSMUST00000201983	lincRNA	rs36839087
5:38531909-38531909	intron_variant	MODIFIER	Wdr1	ENSMUSG00000005103	Transcript	ENSMUST00000005234	protein_coding	-
5:38531909-38531909	upstream_gene_variant	MODIFIER	Wdr1	ENSMUSG00000005103	Transcript	ENSMUST00000200909	retained_intron	-
5:38531909-38531909	upstream_gene_variant	MODIFIER	Wdr1	ENSMUSG00000005103	Transcript	ENSMUST00000201059	retained_intron	-
5:38531909-38531909	intron_variant	MODIFIER	Wdr1	ENSMUSG00000005103	Transcript	ENSMUST00000201260	protein_coding	-
5:38531909-38531909	upstream_gene_variant	MODIFIER	Wdr1	ENSMUSG00000005103	Transcript	ENSMUST00000201538	retained_intron	-
5:38531909-38531909	intron_variant,non_coding_trans	MODIFIER	Wdr1	ENSMUSG00000005103	Transcript	ENSMUST00000202496	processed_transcript	-
5:38531979-38531979	intron_variant	MODIFIER	Wdr1	ENSMUSG00000005103	Transcript	ENSMUST00000005234	protein_coding	rs243749148
5:38531979-38531979	upstream_gene_variant	MODIFIER	Wdr1	ENSMUSG00000005103	Transcript	ENSMUST00000200909	retained_intron	rs243749148
5:38531979-38531979	upstream_gene_variant	MODIFIER	Wdr1	ENSMUSG00000005103	Transcript	ENSMUST00000201059	retained_intron	rs243749148
5:38531979-38531979	intron_variant	MODIFIER	Wdr1	ENSMUSG00000005103	Transcript	ENSMUST00000201260	protein_coding	rs243749148
5:38531979-38531979	upstream_gene_variant	MODIFIER	Wdr1	ENSMUSG00000005103	Transcript	ENSMUST00000201538	retained_intron	rs243749148
5:38531979-38531979	non_coding_transcript_exon_vai	MODIFIER	Wdr1	ENSMUSG00000005103	Transcript	ENSMUST00000202496	processed_transcript	rs243749148
5:49960118-49960118	3_prime_UTR_variant	MODIFIER	Adgra3	ENSMUSG00000029090	Transcript	ENSMUST00000030971	protein_coding	rs33640639
5:52666677-52666677	intron_variant	MODIFIER	Sepsecs	ENSMUSG00000029173	Transcript	ENSMUST00000031069	protein_coding	-
5:52666677-52666677	intron_variant,NMD_transcript_v	MODIFIER	Sepsecs	ENSMUSG00000029173	Transcript	ENSMUST00000123856	nonsense_mediated_decay	-
5:52666677-52666677	upstream_gene_variant	MODIFIER	Sepsecs	ENSMUSG00000029173	Transcript	ENSMUST00000126574	nonsense_mediated_decay	-
5:52666677-52666677	intron_variant,NMD_transcript_v	MODIFIER	Sepsecs	ENSMUSG00000029173	Transcript	ENSMUST00000150709	nonsense_mediated_decay	-
5:52757098-52757098	intron_variant	MODIFIER	Pi4k2b	ENSMUSG00000029186	Transcript	ENSMUST00000031081	protein_coding	rs32953577
5:52757098-52757098	intron_variant	MODIFIER	Pi4k2b	ENSMUSG00000029186	Transcript	ENSMUST00000031082	protein_coding	rs32953577
5:52757098-52757098	non_coding_transcript_exon_vai	MODIFIER	Pi4k2b	ENSMUSG00000029186	Transcript	ENSMUST00000145825	retained_intron	rs32953577
5:52758728-52758728	intron_variant	MODIFIER	Pi4k2b	ENSMUSG00000029186	Transcript	ENSMUST00000031081	protein_coding	rs51448018
5:52758728-52758728	intron_variant	MODIFIER	Pi4k2b	ENSMUSG00000029186	Transcript	ENSMUST00000031082	protein_coding	rs51448018
5:52758728-52758728	downstream_gene_variant	MODIFIER	Pi4k2b	ENSMUSG00000029186	Transcript	ENSMUST00000145825	retained_intron	rs51448018
5:52759544-52759544	intron_variant	MODIFIER	Pi4k2b	ENSMUSG00000029186	Transcript	ENSMUST00000031081	protein_coding	rs46981522
5:52759544-52759544	intron_variant	MODIFIER	Pi4k2b	ENSMUSG00000029186	Transcript	ENSMUST00000031082	protein_coding	rs46981522
5:52759544-52759544	downstream_gene_variant	MODIFIER	Pi4k2b	ENSMUSG00000029186	Transcript	ENSMUST00000145825	retained_intron	rs46981522
5:53662296-53662296	downstream_gene_variant	MODIFIER	Rbpj	ENSMUSG00000039191	Transcript	ENSMUST00000037618	protein_coding	rs33570940
5:53662296-53662296	downstream_gene_variant	MODIFIER	Rbpj	ENSMUSG00000039191	Transcript	ENSMUST00000087360	protein_coding	rs33570940
5:53662296-53662296	downstream_gene_variant	MODIFIER	Rbpj	ENSMUSG00000039191	Transcript	ENSMUST00000113865	protein_coding	rs33570940
5:53662296-53662296	downstream_gene_variant	MODIFIER	Rbpj	ENSMUSG00000039191	Transcript	ENSMUST00000201912	protein_coding	rs33570940
5:53662329-53662329	downstream_gene_variant	MODIFIER	Rbpj	ENSMUSG00000039191	Transcript	ENSMUST00000037618	protein_coding	rs29730820
5:53662329-53662329	downstream_gene_variant	MODIFIER	Rbpj	ENSMUSG00000039191	Transcript	ENSMUST00000087360	protein_coding	rs29730820
5:53662329-53662329	downstream_gene_variant	MODIFIER	Rbpj	ENSMUSG00000039191	Transcript	ENSMUST00000113865	protein_coding	rs29730820
5:53662329-53662329	downstream_gene_variant	MODIFIER	Rbpj	ENSMUSG00000039191	Transcript	ENSMUST00000201912	protein_coding	rs29730820
5:57729016-57729016	3_prime_UTR_variant	MODIFIER	Pcdh7	ENSMUSG00000029108	Transcript	ENSMUST00000068110	protein_coding	rs33452154
5:57729016-57729016	intron_variant	MODIFIER	Pcdh7	ENSMUSG00000029108	Transcript	ENSMUST00000094783	protein_coding	rs33452154
5:57729016-57729016	intron_variant	MODIFIER	Pcdh7	ENSMUSG00000029108	Transcript	ENSMUST00000191837	protein_coding	rs33452154
5:57729016-57729016	intron_variant	MODIFIER	Pcdh7	ENSMUSG00000029108	Transcript	ENSMUST00000192048	protein_coding	rs33452154
5:57729016-57729016	intron_variant	MODIFIER	Pcdh7	ENSMUSG00000029108	Transcript	ENSMUST00000192287	protein_coding	rs33452154
5:57729016-57729016	intron_variant,NMD_transcript_v	MODIFIER	Pcdh7	ENSMUSG00000029108	Transcript	ENSMUST00000195156	nonsense_mediated_decay	rs33452154
5:57729016-57729016	intron_variant	MODIFIER	Pcdh7	ENSMUSG00000029108	Transcript	ENSMUST00000199310	protein_coding	rs33452154

5:57729016-57729016	downstream_gene_variant	MODIFIER	Gm42635	ENSMUSG00000105940	Transcript	ENSMUST00000200266	TEC	rs33452154
5:58129507-58129507	downstream_gene_variant	MODIFIER	Pcdh7	ENSMUSG00000029108	Transcript	ENSMUST00000094783	protein_coding	rs33645796
5:58129507-58129507	3_prime_UTR_variant	MODIFIER	Pcdh7	ENSMUSG00000029108	Transcript	ENSMUST00000191837	protein_coding	rs33645796
5:58129507-58129507	downstream_gene_variant	MODIFIER	Pcdh7	ENSMUSG00000029108	Transcript	ENSMUST00000192048	protein_coding	rs33645796
5:58129507-58129507	3_prime_UTR_variant,NMD_trar	MODIFIER	Pcdh7	ENSMUSG00000029108	Transcript	ENSMUST00000195156	nonsense_mediated_decay	rs33645796
5:58129507-58129507	downstream_gene_variant	MODIFIER	Gm42486	ENSMUSG00000105049	Transcript	ENSMUST00000200369	TEC	rs33645796
5:58130500-58130500	downstream_gene_variant	MODIFIER	Pcdh7	ENSMUSG00000029108	Transcript	ENSMUST00000094783	protein_coding	rs37407901
5:58130500-58130500	3_prime_UTR_variant	MODIFIER	Pcdh7	ENSMUSG00000029108	Transcript	ENSMUST00000191837	protein_coding	rs37407901
5:58130500-58130500	downstream_gene_variant	MODIFIER	Pcdh7	ENSMUSG00000029108	Transcript	ENSMUST00000192048	protein_coding	rs37407901
5:58130500-58130500	downstream_gene_variant	MODIFIER	Pcdh7	ENSMUSG00000029108	Transcript	ENSMUST00000195156	nonsense_mediated_decay	rs37407901
5:58132252-58132252	downstream_gene_variant	MODIFIER	Pcdh7	ENSMUSG00000029108	Transcript	ENSMUST00000094783	protein_coding	rs244488738
5:58132252-58132252	3_prime_UTR_variant	MODIFIER	Pcdh7	ENSMUSG00000029108	Transcript	ENSMUST00000191837	protein_coding	rs244488738
5:58132252-58132252	downstream_gene_variant	MODIFIER	Pcdh7	ENSMUSG00000029108	Transcript	ENSMUST00000192048	protein_coding	rs244488738
5:58132252-58132252	downstream_gene_variant	MODIFIER	Pcdh7	ENSMUSG00000029108	Transcript	ENSMUST00000195156	nonsense_mediated_decay	rs244488738
5:58132655-58132655	downstream_gene_variant	MODIFIER	Pcdh7	ENSMUSG00000029108	Transcript	ENSMUST00000094783	protein_coding	rs33729500
5:58132655-58132655	3_prime_UTR_variant	MODIFIER	Pcdh7	ENSMUSG00000029108	Transcript	ENSMUST00000191837	protein_coding	rs33729500
5:58132655-58132655	downstream_gene_variant	MODIFIER	Pcdh7	ENSMUSG00000029108	Transcript	ENSMUST00000192048	protein_coding	rs33729500
5:58132655-58132655	downstream_gene_variant	MODIFIER	Pcdh7	ENSMUSG00000029108	Transcript	ENSMUST00000195156	nonsense_mediated_decay	rs33729500
5:64338294-64338294	intron_variant	MODIFIER	Tbc1d1	ENSMUSG00000029174	Transcript	ENSMUST00000043893	protein_coding	rs243905008
5:64338294-64338294	intron_variant	MODIFIER	Tbc1d1	ENSMUSG00000029174	Transcript	ENSMUST00000101195	protein_coding	rs243905008
5:64338294-64338294	intron_variant	MODIFIER	Tbc1d1	ENSMUSG00000029174	Transcript	ENSMUST00000119756	protein_coding	rs243905008
5:64338294-64338294	intron_variant	MODIFIER	Tbc1d1	ENSMUSG00000029174	Transcript	ENSMUST00000121370	protein_coding	rs243905008
5:64338294-64338294	intron_variant,non_coding_trans	MODIFIER	Tbc1d1	ENSMUSG00000029174	Transcript	ENSMUST00000140960	processed_transcript	rs243905008
5:64338294-64338294	regulatory_region_variant	MODIFIER	-	-	RegulatoryFeature	ENSMUSR00000209865	promoter_flanking_region	rs243905008
5:64338295-64338295	intron_variant	MODIFIER	Tbc1d1	ENSMUSG00000029174	Transcript	ENSMUST00000043893	protein_coding	-
5:64338295-64338295	intron_variant	MODIFIER	Tbc1d1	ENSMUSG00000029174	Transcript	ENSMUST00000101195	protein_coding	-
5:64338295-64338295	intron_variant	MODIFIER	Tbc1d1	ENSMUSG00000029174	Transcript	ENSMUST00000119756	protein_coding	-
5:64338295-64338295	intron_variant	MODIFIER	Tbc1d1	ENSMUSG00000029174	Transcript	ENSMUST00000121370	protein_coding	-
5:64338295-64338295	intron_variant,non_coding_trans	MODIFIER	Tbc1d1	ENSMUSG00000029174	Transcript	ENSMUST00000140960	processed_transcript	-
5:64338295-64338295	regulatory_region_variant	MODIFIER	-	-	RegulatoryFeature	ENSMUSR00000209865	promoter_flanking_region	-
5:64339486-64339486	intron_variant	MODIFIER	Tbc1d1	ENSMUSG00000029174	Transcript	ENSMUST00000043893	protein_coding	rs30578598
5:64339486-64339486	intron_variant	MODIFIER	Tbc1d1	ENSMUSG00000029174	Transcript	ENSMUST00000101195	protein_coding	rs30578598
5:64339486-64339486	intron_variant	MODIFIER	Tbc1d1	ENSMUSG00000029174	Transcript	ENSMUST00000119756	protein_coding	rs30578598
5:64339486-64339486	intron_variant	MODIFIER	Tbc1d1	ENSMUSG00000029174	Transcript	ENSMUST00000121370	protein_coding	rs30578598
5:64339486-64339486	intron_variant,non_coding_trans	MODIFIER	Tbc1d1	ENSMUSG00000029174	Transcript	ENSMUST00000140960	processed_transcript	rs30578598
5:64339486-64339486	regulatory_region_variant	MODIFIER	-	-	RegulatoryFeature	ENSMUSR00000209865	promoter_flanking_region	rs30578598
5:64349714-64349714	intron_variant	MODIFIER	Tbc1d1	ENSMUSG00000029174	Transcript	ENSMUST00000043893	protein_coding	rs48297543
5:64349714-64349714	intron_variant	MODIFIER	Tbc1d1	ENSMUSG00000029174	Transcript	ENSMUST00000101195	protein_coding	rs48297543
5:64349714-64349714	intron_variant	MODIFIER	Tbc1d1	ENSMUSG00000029174	Transcript	ENSMUST00000119756	protein_coding	rs48297543
5:64349714-64349714	intron_variant	MODIFIER	Tbc1d1	ENSMUSG00000029174	Transcript	ENSMUST00000121370	protein_coding	rs48297543
5:64349714-64349714	intron_variant,non_coding_trans	MODIFIER	Tbc1d1	ENSMUSG00000029174	Transcript	ENSMUST00000140960	processed_transcript	rs48297543
5:64929067-64929067	intron_variant	MODIFIER	Tlr1	ENSMUSG00000044827	Transcript	ENSMUST00000059349	protein_coding	rs226042033
5:64929067-64929067	intron_variant	MODIFIER	Tlr1	ENSMUSG00000044827	Transcript	ENSMUST00000197315	protein_coding	rs226042033
5:64929067-64929067	downstream_gene_variant	MODIFIER	Tlr1	ENSMUSG00000044827	Transcript	ENSMUST00000197886	processed_transcript	rs226042033
5:65019083-65019083	intron_variant	MODIFIER	Fam114a1	ENSMUSG00000029185	Transcript	ENSMUST00000031080	protein_coding	rs31447051
5:65019083-65019083	regulatory_region_variant	MODIFIER	-	-	RegulatoryFeature	ENSMUSR00000210050	promoter_flanking_region	rs31447051
5:65039522-65039522	intron_variant	MODIFIER	Fam114a1	ENSMUSG00000029185	Transcript	ENSMUST00000031080	protein_coding	rs31451154
5:65039522-65039522	regulatory_region_variant	MODIFIER	-	-	RegulatoryFeature	ENSMUSR00000210055	promoter_flanking_region	rs31451154
5:65039757-65039757	intron_variant	MODIFIER	Fam114a1	ENSMUSG00000029185	Transcript	ENSMUST00000031080	protein_coding	rs31451156
5:65039757-65039757	regulatory_region_variant	MODIFIER	-	-	RegulatoryFeature	ENSMUSR00000210055	promoter_flanking_region	rs31451156
5:65040336-65040336	intron_variant	MODIFIER	Fam114a1	ENSMUSG00000029185	Transcript	ENSMUST00000031080	protein_coding	rs46234798
5:65041850-65041850	3_prime_UTR_variant	MODIFIER	Fam114a1	ENSMUSG00000029185	Transcript	ENSMUST00000031080	protein_coding	rs31451161
5:65041865-65041865	3_prime_UTR_variant	MODIFIER	Fam114a1	ENSMUSG00000029185	Transcript	ENSMUST00000031080	protein_coding	rs31451162
5:65156051-65156051	splice_region_variant,intron_vari	LOW	Klhl5	ENSMUSG00000054920	Transcript	ENSMUST00000101191	protein_coding	rs31448857
5:65156051-65156051	upstream_gene_variant	MODIFIER	Klhl5	ENSMUSG00000054920	Transcript	ENSMUST00000203538	nonsense_mediated_decay	rs31448857
5:65156051-65156051	downstream_gene_variant	MODIFIER	Klhl5	ENSMUSG00000054920	Transcript	ENSMUST00000203561	processed_transcript	rs31448857
5:65156051-65156051	splice_region_variant,intron_vari	LOW	Klhl5	ENSMUSG00000054920	Transcript	ENSMUST00000204097	protein_coding	rs31448857
5:65156051-65156051	splice_region_variant,intron_vari	LOW	Klhl5	ENSMUSG00000054920	Transcript	ENSMUST00000204348	protein_coding	rs31448857
5:65156491-65156491	intron_variant	MODIFIER	Klhl5	ENSMUSG00000054920	Transcript	ENSMUST00000101191	protein_coding	rs29732267
5:65156491-65156491	intron_variant,NMD_transcript_v	MODIFIER	Klhl5	ENSMUSG00000054920	Transcript	ENSMUST00000203538	nonsense_mediated_decay	rs29732267
5:65156491-65156491	downstream_gene_variant	MODIFIER	Klhl5	ENSMUSG00000054920	Transcript	ENSMUST00000203561	processed_transcript	rs29732267
5:65156491-65156491	intron_variant	MODIFIER	Klhl5	ENSMUSG00000054920	Transcript	ENSMUST00000204097	protein_coding	rs29732267
5:65156491-65156491	intron_variant	MODIFIER	Klhl5	ENSMUSG00000054920	Transcript	ENSMUST00000204348	protein_coding	rs29732267
5:65157240-65157243	intron_variant	MODIFIER	Klhl5	ENSMUSG00000054920	Transcript	ENSMUST00000101191	protein_coding	rs387788566
5:65157240-65157243	intron_variant,NMD_transcript_v	MODIFIER	Klhl5	ENSMUSG00000054920	Transcript	ENSMUST00000203538	nonsense_mediated_decay	rs387788566
5:65157240-65157243	downstream_gene_variant	MODIFIER	Klhl5	ENSMUSG00000054920	Transcript	ENSMUST00000203561	processed_transcript	rs387788566
5:65157240-65157243	intron_variant	MODIFIER	Klhl5	ENSMUSG00000054920	Transcript	ENSMUST00000204097	protein_coding	rs387788566
5:65157240-65157243	intron_variant	MODIFIER	Klhl5	ENSMUSG00000054920	Transcript	ENSMUST00000204348	protein_coding	rs387788566
5:65157240-65157243	regulatory_region_variant	MODIFIER	-	-	RegulatoryFeature	ENSMUSR00000210091	promoter_flanking_region	rs387788566

5:65279005-65279005	intron_variant	MODIFIER	Rfc1	ENSMUSG00000029191	Transcript	ENSMUST00000172732	protein_coding	rs31456859
5:65279005-65279005	intron_variant	MODIFIER	Rfc1	ENSMUSG00000029191	Transcript	ENSMUST00000203471	protein_coding	rs31456859
5:65279005-65279005	intron_variant	MODIFIER	Rfc1	ENSMUSG00000029191	Transcript	ENSMUST00000203581	protein_coding	rs31456859
5:65279005-65279005	intron_variant	MODIFIER	Rfc1	ENSMUSG00000029191	Transcript	ENSMUST00000204965	protein_coding	rs31456859
5:65422523-65422527	intron_variant	MODIFIER	Ugdh	ENSMUSG00000029201	Transcript	ENSMUST00000031103	protein_coding	rs232450249
5:65422523-65422527	non_coding_transcript_exon_vai	MODIFIER	Ugdh	ENSMUSG00000029201	Transcript	ENSMUST00000125375	retained_intron	rs232450249
5:65422523-65422527	downstream_gene_variant	MODIFIER	Ugdh	ENSMUSG00000029201	Transcript	ENSMUST00000131263	protein_coding	rs232450249
5:65422523-65422527	intron_variant,non_coding_trans	MODIFIER	Ugdh	ENSMUSG00000029201	Transcript	ENSMUST00000139402	retained_intron	rs232450249
5:65422523-65422527	downstream_gene_variant	MODIFIER	Gm43552	ENSMUSG00000105835	Transcript	ENSMUST00000196121	protein_coding	rs232450249
5:65461285-654461285	intron_variant,NMD_transcript_v	MODIFIER	Smim14	ENSMUSG00000037822	Transcript	ENSMUST00000040532	nonsense_mediated_decay	rs244398578
5:65461285-65461285	intron_variant	MODIFIER	Smim14	ENSMUSG00000037822	Transcript	ENSMUST00000121661	protein_coding	rs244398578
5:65461285-65461285	non_coding_transcript_exon_vai	MODIFIER	Smim14	ENSMUSG00000037822	Transcript	ENSMUST00000135258	retained_intron	rs244398578
5:65461285-65461285	intron_variant	MODIFIER	Smim14	ENSMUSG00000037822	Transcript	ENSMUST00000139122	protein_coding	rs244398578
5:65461285-65461285	intron_variant	MODIFIER	Smim14	ENSMUSG00000037822	Transcript	ENSMUST00000149167	protein_coding	rs244398578
5:65461285-65461285	intron_variant	MODIFIER	Gm43552	ENSMUSG00000105835	Transcript	ENSMUST00000196121	protein_coding	rs244398578
5:65461285-65461285	upstream_gene_variant	MODIFIER	Smim14	ENSMUSG00000037822	Transcript	ENSMUST00000200111	retained_intron	rs244398578
5:65490511-65490511	intron_variant,NMD_transcript_v	MODIFIER	Smim14	ENSMUSG00000037822	Transcript	ENSMUST00000040532	nonsense_mediated_decay	rs33154886
5:65490511-65490511	intron_variant	MODIFIER	Smim14	ENSMUSG00000037822	Transcript	ENSMUST00000121661	protein_coding	rs33154886
5:65490511-65490511	intron_variant,non_coding_trans	MODIFIER	Smim14	ENSMUSG00000037822	Transcript	ENSMUST00000125954	retained_intron	rs33154886
5:65490511-65490511	intron_variant	MODIFIER	Smim14	ENSMUSG00000037822	Transcript	ENSMUST00000139122	protein_coding	rs33154886
5:65490511-65490511	intron_variant,non_coding_trans	MODIFIER	Smim14	ENSMUSG00000037822	Transcript	ENSMUST00000144562	retained_intron	rs33154886
5:65490511-65490511	intron_variant	MODIFIER	Smim14	ENSMUSG00000037822	Transcript	ENSMUST00000149167	protein_coding	rs33154886
5:65490511-65490511	intron_variant	MODIFIER	Gm43552	ENSMUSG00000105835	Transcript	ENSMUST00000196121	protein_coding	rs33154886
5:65490511-65490511	non_coding_transcript_exon_vai	MODIFIER	Smim14	ENSMUSG00000037822	Transcript	ENSMUST00000198981	retained_intron	rs33154886
5:65490511-65490511	upstream_gene_variant	MODIFIER	4930589O11Rik	ENSMUSG00000107195	Transcript	ENSMUST00000200768	TEC	rs33154886
5:65490511-65490511	regulatory_region_variant	MODIFIER	-	-	RegulatoryFeature	ENSMUSR00000681560	promoter	rs33154886
5:65490512-65490512	intron_variant,NMD_transcript_v	MODIFIER	Smim14	ENSMUSG00000037822	Transcript	ENSMUST00000040532	nonsense_mediated_decay	rs33417098
5:65490512-65490512	intron_variant	MODIFIER	Smim14	ENSMUSG00000037822	Transcript	ENSMUST00000121661	protein_coding	rs33417098
5:65490512-65490512	intron_variant,non_coding_trans	MODIFIER	Smim14	ENSMUSG00000037822	Transcript	ENSMUST00000125954	retained_intron	rs33417098
5:65490512-65490512	intron_variant	MODIFIER	Smim14	ENSMUSG00000037822	Transcript	ENSMUST00000139122	protein_coding	rs33417098
5:65490512-65490512	intron_variant,non_coding_trans	MODIFIER	Smim14	ENSMUSG00000037822	Transcript	ENSMUST00000144562	retained_intron	rs33417098
5:65490512-65490512	intron_variant	MODIFIER	Smim14	ENSMUSG00000037822	Transcript	ENSMUST00000149167	protein_coding	rs33417098
5:65490512-65490512	intron_variant	MODIFIER	Gm43552	ENSMUSG00000105835	Transcript	ENSMUST00000196121	protein_coding	rs33417098
5:65490512-65490512	non_coding_transcript_exon_vai	MODIFIER	Smim14	ENSMUSG00000037822	Transcript	ENSMUST00000198981	retained_intron	rs33417098
5:65490512-65490512	upstream_gene_variant	MODIFIER	4930589O11Rik	ENSMUSG00000107195	Transcript	ENSMUST00000200768	TEC	rs33417098
5:65490512-65490512	regulatory_region_variant	MODIFIER	-	-	RegulatoryFeature	ENSMUSR00000681560	promoter	rs33417098
5:65606089-65606089	non_coding_transcript_exon_vai	MODIFIER	Pds5a	ENSMUSG00000029202	Transcript	ENSMUST00000201673	retained_intron	rs31458533
5:65606089-65606089	3_prime_UTR_variant	MODIFIER	Pds5a	ENSMUSG00000029202	Transcript	ENSMUST00000201948	protein_coding	rs31458533
5:65606089-65606089	downstream_gene_variant	MODIFIER	Pds5a	ENSMUSG00000029202	Transcript	ENSMUST00000202648	protein_coding	rs31458533
5:65779987-65779987	intron_variant	MODIFIER	N4bp2	ENSMUSG00000037795	Transcript	ENSMUST00000087264	protein_coding	rs33590837
5:65779987-65779987	downstream_gene_variant	MODIFIER	Gm24346	ENSMUSG00000089337	Transcript	ENSMUST00000158712	snoRNA	rs33590837
5:65779987-65779987	non_coding_transcript_exon_vai	MODIFIER	Gm42648	ENSMUSG00000106682	Transcript	ENSMUST00000200774	TEC	rs33590837
5:65779987-65779987	intron_variant	MODIFIER	N4bp2	ENSMUSG00000037795	Transcript	ENSMUST00000201489	protein_coding	rs33590837
5:65779987-65779987	downstream_gene_variant	MODIFIER	N4bp2	ENSMUSG00000037795	Transcript	ENSMUST00000201503	retained_intron	rs33590837
5:65779987-65779987	intron_variant	MODIFIER	N4bp2	ENSMUSG00000037795	Transcript	ENSMUST00000201615	protein_coding	rs33590837
5:65779987-65779987	upstream_gene_variant	MODIFIER	N4bp2	ENSMUSG00000037795	Transcript	ENSMUST00000202817	retained_intron	rs33590837
5:65779987-65779987	intron_variant,non_coding_trans	MODIFIER	N4bp2	ENSMUSG00000037795	Transcript	ENSMUST00000202934	processed_transcript	rs33590837
5:66687170-66687170	3_prime_UTR_variant	MODIFIER	Uchl1	ENSMUSG00000029223	Transcript	ENSMUST00000031131	protein_coding	rs13459521
5:66687170-66687170	non_coding_transcript_exon_vai	MODIFIER	Uchl1	ENSMUSG00000029223	Transcript	ENSMUST00000176015	retained_intron	rs13459521
5:66687170-66687170	downstream_gene_variant	MODIFIER	Uchl1	ENSMUSG00000029223	Transcript	ENSMUST00000202339	retained_intron	rs13459521
5:66687170-66687170	upstream_gene_variant	MODIFIER	Gm43279	ENSMUSG00000107300	Transcript	ENSMUST00000202376	TEC	rs13459521
5:66687170-66687170	regulatory_region_variant	MODIFIER	-	-	RegulatoryFeature	ENSMUSR00000423720	CTCF_binding_site	rs13459521
5:67260253-67260253	upstream_gene_variant	MODIFIER	Tmem33	ENSMUSG00000037720	Transcript	ENSMUST00000037918	protein_coding	rs29529097
5:67260253-67260253	upstream_gene_variant	MODIFIER	Tmem33	ENSMUSG00000037720	Transcript	ENSMUST00000160352	protein_coding	rs29529097
5:67260253-67260253	upstream_gene_variant	MODIFIER	Tmem33	ENSMUSG00000037720	Transcript	ENSMUST00000161233	protein_coding	rs29529097
5:67260253-67260253	upstream_gene_variant	MODIFIER	Tmem33	ENSMUSG00000037720	Transcript	ENSMUST00000161369	protein_coding	rs29529097
5:67260253-67260253	upstream_gene_variant	MODIFIER	Tmem33	ENSMUSG00000037720	Transcript	ENSMUST00000162074	nonsense_mediated_decay	rs29529097
5:67260253-67260253	upstream_gene_variant	MODIFIER	Tmem33	ENSMUSG00000037720	Transcript	ENSMUST00000162543	protein_coding	rs29529097
5:67260253-67260253	upstream_gene_variant	MODIFIER	Tmem33	ENSMUSG00000037720	Transcript	ENSMUST00000163028	retained_intron	rs29529097
5:67260253-67260253	non_coding_transcript_exon_vai	MODIFIER	Gm42670	ENSMUSG00000107319	Transcript	ENSMUST00000201816	antisense	rs29529097
5:67260253-67260253	upstream_gene_variant	MODIFIER	Tmem33	ENSMUSG00000037720	Transcript	ENSMUST00000201979	nonsense_mediated_decay	rs29529097
5:67260253-67260253	upstream_gene_variant	MODIFIER	Tmem33	ENSMUSG00000037720	Transcript	ENSMUST00000202324	retained_intron	rs29529097
5:67260253-67260253	regulatory_region_variant	MODIFIER	-	-	RegulatoryFeature	ENSMUSR00000210570	promoter	rs29529097
5:67666732-67666732	intron_variant	MODIFIER	Atp8a1	ENSMUSG00000037685	Transcript	ENSMUST00000037380	protein_coding	rs48890369
5:67666732-67666732	intron_variant	MODIFIER	Atp8a1	ENSMUSG00000037685	Transcript	ENSMUST00000072971	protein_coding	rs48890369
5:67666732-67666732	intron_variant	MODIFIER	Atp8a1	ENSMUSG00000037685	Transcript	ENSMUST00000135930	protein_coding	rs48890369
5:67666732-67666732	intron_variant,NMD_transcript_v	MODIFIER	Atp8a1	ENSMUSG00000037685	Transcript	ENSMUST00000200955	nonsense_mediated_decay	rs48890369
5:73310750-73310751	intron_variant	MODIFIER	Ociad1	ENSMUSG00000029152	Transcript	ENSMUST00000031038	protein_coding	rs233566073
5:73310750-73310751	intron_variant	MODIFIER	Ociad1	ENSMUSG00000029152	Transcript	ENSMUST00000071081	protein_coding	rs233566073

5:73310750-73310751	downstream_gene_variant	MODIFIER	Ociad1	ENSMUSG00000029152	Transcript	ENSMUST00000166823	protein_coding	rs233566073
5:73310750-73310751	intron_variant	MODIFIER	Ociad1	ENSMUSG00000029152	Transcript	ENSMUST00000200935	protein_coding	rs233566073
5:73310750-73310751	upstream_gene_variant	MODIFIER	Gm42733	ENSMUSG00000107292	Transcript	ENSMUST00000201272	TEC	rs233566073
5:73310750-73310751	intron_variant	MODIFIER	Ociad1	ENSMUSG00000029152	Transcript	ENSMUST00000201556	protein_coding	rs233566073
5:73310750-73310751	intron_variant,NMD_transcript_v	MODIFIER	Ociad1	ENSMUSG00000029152	Transcript	ENSMUST00000201739	nonsense_mediated_decay	rs233566073
5:73310750-73310751	downstream_gene_variant	MODIFIER	Ociad1	ENSMUSG00000029152	Transcript	ENSMUST00000202237	protein_coding	rs233566073
5:73310750-73310751	downstream_gene_variant	MODIFIER	Ociad1	ENSMUSG00000029152	Transcript	ENSMUST00000202250	protein_coding	rs233566073
5:73310750-73310751	upstream_gene_variant	MODIFIER	Gm42732	ENSMUSG00000107331	Transcript	ENSMUST00000202887	TEC	rs233566073
5:73310750-73310751	regulatory_region_variant	MODIFIER	-	-	RegulatoryFeature	ENSMUSR00000682380	TF_binding_site	rs233566073
5:74204987-74204987	3_prime_UTR_variant	MODIFIER	Scfd2	ENSMUSG00000062110	Transcript	ENSMUST00000072857	protein_coding	rs33759100
5:74204987-74204987	downstream_gene_variant	MODIFIER	Scfd2	ENSMUSG00000062110	Transcript	ENSMUST00000113542	protein_coding	rs33759100
5:74204987-74204987	downstream_gene_variant	MODIFIER	Scfd2	ENSMUSG00000062110	Transcript	ENSMUST00000146015	retained_intron	rs33759100
5:74204987-74204987	downstream_gene_variant	MODIFIER	Scfd2	ENSMUSG00000062110	Transcript	ENSMUST00000202465	processed_transcript	rs33759100
5:74204987-74204987	regulatory_region_variant	MODIFIER	-	-	RegulatoryFeature	ENSMUSR00000424182	promoter_flanking_region	rs33759100
5:74204989-74204989	3_prime_UTR_variant	MODIFIER	Scfd2	ENSMUSG00000062110	Transcript	ENSMUST00000072857	protein_coding	rs33662599
5:74204989-74204989	downstream_gene_variant	MODIFIER	Scfd2	ENSMUSG00000062110	Transcript	ENSMUST00000113542	protein_coding	rs33662599
5:74204989-74204989	downstream_gene_variant	MODIFIER	Scfd2	ENSMUSG00000062110	Transcript	ENSMUST00000146015	retained_intron	rs33662599
5:74204989-74204989	downstream_gene_variant	MODIFIER	Scfd2	ENSMUSG00000062110	Transcript	ENSMUST00000202465	processed_transcript	rs33662599
5:74204989-74204989	regulatory_region_variant	MODIFIER	-	-	RegulatoryFeature	ENSMUSR00000424182	promoter_flanking_region	rs33662599
5:74205098-74205098	3_prime_UTR_variant	MODIFIER	Scfd2	ENSMUSG00000062110	Transcript	ENSMUST00000072857	protein_coding	rs29562162
5:74205098-74205098	downstream_gene_variant	MODIFIER	Scfd2	ENSMUSG00000062110	Transcript	ENSMUST00000113542	protein_coding	rs29562162
5:74205098-74205098	downstream_gene_variant	MODIFIER	Scfd2	ENSMUSG00000062110	Transcript	ENSMUST00000146015	retained_intron	rs29562162
5:74205098-74205098	non_coding_transcript_exon_va	MODIFIER	Scfd2	ENSMUSG00000062110	Transcript	ENSMUST00000202465	processed_transcript	rs29562162
5:74205098-74205098	regulatory_region_variant	MODIFIER	-	-	RegulatoryFeature	ENSMUSR00000424182	promoter_flanking_region	rs29562162
5:74205903-74205903	3_prime_UTR_variant	MODIFIER	Scfd2	ENSMUSG00000062110	Transcript	ENSMUST00000072857	protein_coding	rs227051270
5:74205903-74205903	downstream_gene_variant	MODIFIER	Scfd2	ENSMUSG00000062110	Transcript	ENSMUST00000113542	protein_coding	rs227051270
5:74205903-74205903	downstream_gene_variant	MODIFIER	Scfd2	ENSMUSG00000062110	Transcript	ENSMUST00000146015	retained_intron	rs227051270
5:74205903-74205903	intron_variant,non_coding_trans	MODIFIER	Scfd2	ENSMUSG00000062110	Transcript	ENSMUST00000202465	processed_transcript	rs227051270
5:74205903-74205903	regulatory_region_variant	MODIFIER	-	-	RegulatoryFeature	ENSMUSR00000424182	promoter_flanking_region	rs227051270
5:74441623-74441623	intron_variant	MODIFIER	Scfd2	ENSMUSG00000062110	Transcript	ENSMUST00000072857	protein_coding	rs29624941
5:74441623-74441623	intron_variant	MODIFIER	Scfd2	ENSMUSG00000062110	Transcript	ENSMUST00000113542	protein_coding	rs29624941
5:74451818-74451818	intron_variant	MODIFIER	Scfd2	ENSMUSG00000062110	Transcript	ENSMUST00000072857	protein_coding	rs33318651
5:74451818-74451818	intron_variant,NMD_transcript_v	MODIFIER	Scfd2	ENSMUSG00000062110	Transcript	ENSMUST00000075848	nonsense_mediated_decay	rs33318651
5:74451818-74451818	intron_variant	MODIFIER	Scfd2	ENSMUSG00000062110	Transcript	ENSMUST00000113542	protein_coding	rs33318651
5:74451818-74451818	downstream_gene_variant	MODIFIER	Scfd2	ENSMUSG00000062110	Transcript	ENSMUST00000151474	protein_coding	rs33318651
5:76150708-76150708	intron_variant	MODIFIER	Srd5a3	ENSMUSG00000029233	Transcript	ENSMUST00000031143	protein_coding	rs3155664
5:76150708-76150708	intron_variant	MODIFIER	Srd5a3	ENSMUSG00000029233	Transcript	ENSMUST00000113506	protein_coding	rs3155664
5:76150708-76150708	intron_variant	MODIFIER	Srd5a3	ENSMUSG00000029233	Transcript	ENSMUST00000113507	protein_coding	rs3155664
5:76150708-76150708	upstream_gene_variant	MODIFIER	Srd5a3	ENSMUSG00000029233	Transcript	ENSMUST00000124217	retained_intron	rs3155664
5:76150708-76150708	downstream_gene_variant	MODIFIER	Srd5a3	ENSMUSG00000029233	Transcript	ENSMUST00000127278	protein_coding	rs3155664
5:76150708-76150708	non_coding_transcript_exon_va	MODIFIER	Srd5a3	ENSMUSG00000029233	Transcript	ENSMUST00000138699	retained_intron	rs3155664
5:76150708-76150708	downstream_gene_variant	MODIFIER	Srd5a3	ENSMUSG00000029233	Transcript	ENSMUST00000152642	protein_coding	rs3155664
5:76151525-76151525	intron_variant	MODIFIER	Srd5a3	ENSMUSG00000029233	Transcript	ENSMUST00000031143	protein_coding	rs255004139
5:76151525-76151525	intron_variant	MODIFIER	Srd5a3	ENSMUSG00000029233	Transcript	ENSMUST00000113506	protein_coding	rs255004139
5:76151525-76151525	intron_variant	MODIFIER	Srd5a3	ENSMUSG00000029233	Transcript	ENSMUST00000113507	protein_coding	rs255004139
5:76151525-76151525	upstream_gene_variant	MODIFIER	Srd5a3	ENSMUSG00000029233	Transcript	ENSMUST00000124217	retained_intron	rs255004139
5:76151525-76151525	downstream_gene_variant	MODIFIER	Srd5a3	ENSMUSG00000029233	Transcript	ENSMUST00000127278	protein_coding	rs255004139
5:76151525-76151525	downstream_gene_variant	MODIFIER	Srd5a3	ENSMUSG00000029233	Transcript	ENSMUST00000138699	retained_intron	rs255004139
5:76151525-76151525	downstream_gene_variant	MODIFIER	Srd5a3	ENSMUSG00000029233	Transcript	ENSMUST00000152642	protein_coding	rs255004139
5:76156115-76156115	downstream_gene_variant	MODIFIER	Srd5a3	ENSMUSG00000029233	Transcript	ENSMUST00000031143	protein_coding	rs3155653
5:76156115-76156115	downstream_gene_variant	MODIFIER	Srd5a3	ENSMUSG00000029233	Transcript	ENSMUST00000113506	protein_coding	rs3155653
5:76156115-76156115	downstream_gene_variant	MODIFIER	Srd5a3	ENSMUSG00000029233	Transcript	ENSMUST00000113507	protein_coding	rs3155653
5:76156115-76156115	downstream_gene_variant	MODIFIER	Srd5a3	ENSMUSG00000029233	Transcript	ENSMUST00000124217	retained_intron	rs3155653
5:76156115-76156115	downstream_gene_variant	MODIFIER	Srd5a3	ENSMUSG00000029233	Transcript	ENSMUST00000138699	retained_intron	rs3155653
5:76185655-76185655	intron_variant	MODIFIER	Tmem165	ENSMUSG00000029234	Transcript	ENSMUST00000031144	protein_coding	rs3153698
5:76185655-76185655	intron_variant,non_coding_trans	MODIFIER	Tmem165	ENSMUSG00000029234	Transcript	ENSMUST00000136424	processed_transcript	rs3153698
5:76185655-76185655	regulatory_region_variant	MODIFIER	-	-	RegulatoryFeature	ENSMUSR00000211520	promoter	rs3153698
5:76202347-76202347	intron_variant	MODIFIER	Tmem165	ENSMUSG00000029234	Transcript	ENSMUST00000031144	protein_coding	rs3153685
5:76202347-76202347	intron_variant	MODIFIER	Tmem165	ENSMUSG00000029234	Transcript	ENSMUST00000130842	protein_coding	rs3153685
5:76202347-76202347	intron_variant,non_coding_trans	MODIFIER	Tmem165	ENSMUSG00000029234	Transcript	ENSMUST00000138544	retained_intron	rs3153685
5:76202347-76202347	upstream_gene_variant	MODIFIER	Tmem165	ENSMUSG00000029234	Transcript	ENSMUST00000153633	processed_transcript	rs3153685
5:76210856-76210856	downstream_gene_variant	MODIFIER	Tmem165	ENSMUSG00000029234	Transcript	ENSMUST00000031144	protein_coding	rs3155605
5:76210856-76210856	downstream_gene_variant	MODIFIER	Clock	ENSMUSG00000029238	Transcript	ENSMUST00000075159	protein_coding	rs3155605
5:76210856-76210856	downstream_gene_variant	MODIFIER	Tmem165	ENSMUSG00000029234	Transcript	ENSMUST00000153633	processed_transcript	rs3155605
5:76210856-76210856	downstream_gene_variant	MODIFIER	Clock	ENSMUSG00000029238	Transcript	ENSMUST00000202122	protein_coding	rs3155605
5:76210856-76210856	3_prime_UTR_variant	MODIFIER	Clock	ENSMUSG00000029238	Transcript	ENSMUST00000202651	protein_coding	rs3155605
5:86093305-86093305	intron_variant	MODIFIER	Stap1	ENSMUSG00000029254	Transcript	ENSMUST00000031171	protein_coding	rs33398007
5:86093305-86093305	intron_variant,non_coding_trans	MODIFIER	Stap1	ENSMUSG00000029254	Transcript	ENSMUST00000123393	retained_intron	rs33398007
5:86093305-86093305	intron_variant,non_coding_trans	MODIFIER	Stap1	ENSMUSG00000029254	Transcript	ENSMUST00000138076	retained_intron	rs33398007

5:86093305-86093305	intron_variant	MODIFIER	Stap1	ENSMUSG00000029254	Transcript	ENSMUST00000198435	protein_coding	rs33398007
5:86109123-86109123	downstream_gene_variant	MODIFIER	Uba6	ENSMUSG00000035898	Transcript	ENSMUST00000039373	protein_coding	rs31656666
5:86109123-86109123	downstream_gene_variant	MODIFIER	Uba6	ENSMUSG00000035898	Transcript	ENSMUST00000113373	protein_coding	rs31656666
5:86109123-86109123	downstream_gene_variant	MODIFIER	Stap1	ENSMUSG00000029254	Transcript	ENSMUST00000198435	protein_coding	rs31656666
5:89676466-89676472	downstream_gene_variant	MODIFIER	Adamts3	ENSMUSG00000043635	Transcript	ENSMUST00000061427	protein_coding	rs214336150
5:89676466-89676472	downstream_gene_variant	MODIFIER	Adamts3	ENSMUSG00000043635	Transcript	ENSMUST00000163159	protein_coding	rs214336150
5:89697618-89697618	intron_variant	MODIFIER	Adamts3	ENSMUSG00000043635	Transcript	ENSMUST00000061427	protein_coding	rs33254711
5:89697618-89697618	intron_variant	MODIFIER	Adamts3	ENSMUSG00000043635	Transcript	ENSMUST00000163159	protein_coding	rs33254711
5:89708675-89708675	synonymous_variant	LOW	Adamts3	ENSMUSG00000043635	Transcript	ENSMUST00000061427	protein_coding	rs33109821
5:89708675-89708675	synonymous_variant	LOW	Adamts3	ENSMUSG00000043635	Transcript	ENSMUST00000163159	protein_coding	rs33109821
5:90216217-90216217	intron_variant	MODIFIER	Cox18	ENSMUSG00000035505	Transcript	ENSMUST00000048363	protein_coding	rs31699010
5:90216217-90216217	intron_variant	MODIFIER	Cox18	ENSMUSG00000035505	Transcript	ENSMUST00000118816	protein_coding	rs31699010
5:90216217-90216217	downstream_gene_variant	MODIFIER	Cox18	ENSMUSG00000035505	Transcript	ENSMUST00000131068	retained_intron	rs31699010
5:90216217-90216217	upstream_gene_variant	MODIFIER	Cox18	ENSMUSG00000035505	Transcript	ENSMUST00000133905	processed_transcript	rs31699010
5:90216217-90216217	intron_variant,non_coding_trans	MODIFIER	Cox18	ENSMUSG00000035505	Transcript	ENSMUST00000144898	retained_intron	rs31699010
5:90216217-90216217	downstream_gene_variant	MODIFIER	Cox18	ENSMUSG00000035505	Transcript	ENSMUST00000148480	nonsense_mediated_decay	rs31699010
5:90216217-90216217	upstream_gene_variant	MODIFIER	Cox18	ENSMUSG00000035505	Transcript	ENSMUST00000200475	retained_intron	rs31699010
5:90217273-90217273	intron_variant	MODIFIER	Cox18	ENSMUSG00000035505	Transcript	ENSMUST00000048363	protein_coding	rs31699985
5:90217273-90217273	intron_variant	MODIFIER	Cox18	ENSMUSG00000035505	Transcript	ENSMUST00000118816	protein_coding	rs31699985
5:90217273-90217273	downstream_gene_variant	MODIFIER	Cox18	ENSMUSG00000035505	Transcript	ENSMUST00000131068	retained_intron	rs31699985
5:90217273-90217273	upstream_gene_variant	MODIFIER	Cox18	ENSMUSG00000035505	Transcript	ENSMUST00000133905	processed_transcript	rs31699985
5:90217273-90217273	intron_variant,non_coding_trans	MODIFIER	Cox18	ENSMUSG00000035505	Transcript	ENSMUST00000144898	retained_intron	rs31699985
5:90217273-90217273	downstream_gene_variant	MODIFIER	Cox18	ENSMUSG00000035505	Transcript	ENSMUST00000148480	nonsense_mediated_decay	rs31699985
5:90217273-90217273	upstream_gene_variant	MODIFIER	Cox18	ENSMUSG00000035505	Transcript	ENSMUST00000200475	retained_intron	rs31699985
5:90229878-90229878	intron_variant	MODIFIER	Ankrd17	ENSMUSG00000055204	Transcript	ENSMUST00000014421	protein_coding	rs50590842
5:90229878-90229878	intron_variant	MODIFIER	Ankrd17	ENSMUSG00000055204	Transcript	ENSMUST00000081914	protein_coding	rs50590842
5:90229878-90229878	intron_variant	MODIFIER	Ankrd17	ENSMUSG00000055204	Transcript	ENSMUST00000168058	protein_coding	rs50590842
5:90229878-90229878	intron_variant,non_coding_trans	MODIFIER	Ankrd17	ENSMUSG00000055204	Transcript	ENSMUST00000196919	retained_intron	rs50590842
5:90229878-90229878	intron_variant	MODIFIER	Ankrd17	ENSMUSG00000055204	Transcript	ENSMUST00000197021	protein_coding	rs50590842
5:90229878-90229878	regulatory_region_variant	MODIFIER	-	-	RegulatoryFeature	ENSMUSR00000212408	open_chromatin_region	rs50590842
5:90239675-90239675	intron_variant	MODIFIER	Ankrd17	ENSMUSG00000055204	Transcript	ENSMUST00000014421	protein_coding	rs45775342
5:90239675-90239675	intron_variant	MODIFIER	Ankrd17	ENSMUSG00000055204	Transcript	ENSMUST00000081914	protein_coding	rs45775342
5:90239675-90239675	intron_variant	MODIFIER	Ankrd17	ENSMUSG00000055204	Transcript	ENSMUST00000168058	protein_coding	rs45775342
5:90239675-90239675	intron_variant	MODIFIER	Ankrd17	ENSMUSG00000055204	Transcript	ENSMUST00000197021	protein_coding	rs45775342
5:90239675-90239675	non_coding_transcript_exon_va	MODIFIER	Ankrd17	ENSMUSG00000055204	Transcript	ENSMUST00000197327	retained_intron	rs45775342
5:90608943-90608943	synonymous_variant	LOW	Rassf6	ENSMUSG00000029370	Transcript	ENSMUST00000031317	protein_coding	-
5:90608943-90608943	synonymous_variant	LOW	Rassf6	ENSMUSG00000029370	Transcript	ENSMUST00000202704	protein_coding	-
5:90608943-90608943	synonymous_variant	LOW	Rassf6	ENSMUSG00000029370	Transcript	ENSMUST00000202784	protein_coding	-
5:90608943-90608943	3_prime_UTR_variant,NMD_tra	MODIFIER	Rassf6	ENSMUSG00000029370	Transcript	ENSMUST00000202807	nonsense_mediated_decay	-
5:90608943-90608943	regulatory_region_variant	MODIFIER	-	-	RegulatoryFeature	ENSMUSR00000683906	promoter_flanking_region	-
5:90608945-90608945	missense_variant	MODERATE	Rassf6	ENSMUSG00000029370	Transcript	ENSMUST00000031317	protein_coding	-
5:90608945-90608945	missense_variant	MODERATE	Rassf6	ENSMUSG00000029370	Transcript	ENSMUST00000202704	protein_coding	-
5:90608945-90608945	missense_variant	MODERATE	Rassf6	ENSMUSG00000029370	Transcript	ENSMUST00000202784	protein_coding	-
5:90608945-90608945	3_prime_UTR_variant,NMD_tra	MODIFIER	Rassf6	ENSMUSG00000029370	Transcript	ENSMUST00000202807	nonsense_mediated_decay	-
5:90608945-90608945	regulatory_region_variant	MODIFIER	-	-	RegulatoryFeature	ENSMUSR00000683906	promoter_flanking_region	-
5:90640640-90640640	upstream_gene_variant	MODIFIER	Rassf6	ENSMUSG00000029370	Transcript	ENSMUST00000031317	protein_coding	rs47653411
5:90640640-90640640	upstream_gene_variant	MODIFIER	Rassf6	ENSMUSG00000029370	Transcript	ENSMUST00000201121	nonsense_mediated_decay	rs47653411
5:90640640-90640640	upstream_gene_variant	MODIFIER	Rassf6	ENSMUSG00000029370	Transcript	ENSMUST00000201370	protein_coding	rs47653411
5:90640640-90640640	non_coding_transcript_exon_va	MODIFIER	Rassf6	ENSMUSG00000029370	Transcript	ENSMUST00000201436	processed_transcript	rs47653411
5:90640640-90640640	5_prime_UTR_variant	MODIFIER	Rassf6	ENSMUSG00000029370	Transcript	ENSMUST00000202704	protein_coding	rs47653411
5:90640640-90640640	upstream_gene_variant	MODIFIER	Rassf6	ENSMUSG00000029370	Transcript	ENSMUST00000202784	protein_coding	rs47653411
5:90640640-90640640	regulatory_region_variant	MODIFIER	-	-	RegulatoryFeature	ENSMUSR00000212483	promoter	rs47653411
5:91947161-91947161	downstream_gene_variant	MODIFIER	Rchy1	ENSMUSG00000029397	Transcript	ENSMUST00000031345	protein_coding	rs230764789
5:91947161-91947161	downstream_gene_variant	MODIFIER	Rchy1	ENSMUSG00000029397	Transcript	ENSMUST00000138351	retained_intron	rs230764789
5:91947161-91947161	downstream_gene_variant	MODIFIER	Rchy1	ENSMUSG00000029397	Transcript	ENSMUST00000140670	processed_transcript	rs230764789
5:91947161-91947161	downstream_gene_variant	MODIFIER	Rchy1	ENSMUSG00000029397	Transcript	ENSMUST00000169948	protein_coding	rs230764789
5:91952694-91952694	intron_variant	MODIFIER	Rchy1	ENSMUSG00000029397	Transcript	ENSMUST00000031345	protein_coding	rs29545223
5:91952694-91952694	intron_variant,non_coding_trans	MODIFIER	Rchy1	ENSMUSG00000029397	Transcript	ENSMUST00000138351	retained_intron	rs29545223
5:91952694-91952694	intron_variant,non_coding_trans	MODIFIER	Rchy1	ENSMUSG00000029397	Transcript	ENSMUST00000140670	processed_transcript	rs29545223
5:91952694-91952694	intron_variant	MODIFIER	Rchy1	ENSMUSG00000029397	Transcript	ENSMUST00000169948	protein_coding	rs29545223
5:91972566-91972566	downstream_gene_variant	MODIFIER	Thap6	ENSMUSG00000102644	Transcript	ENSMUST00000191860	transcribed_unitary_pseudogene	rs33043111
5:91972566-91972566	downstream_gene_variant	MODIFIER	Thap6	ENSMUSG00000102644	Transcript	ENSMUST00000192174	processed_transcript	rs33043111
5:91972566-91972566	downstream_gene_variant	MODIFIER	Thap6	ENSMUSG00000102644	Transcript	ENSMUST00000192939	processed_transcript	rs33043111
5:92006271-92006271	3_prime_UTR_variant	MODIFIER	Cdkl2	ENSMUSG00000029403	Transcript	ENSMUST00000069937	protein_coding	rs29563926
5:92006271-92006271	3_prime_UTR_variant	MODIFIER	Cdkl2	ENSMUSG00000029403	Transcript	ENSMUST00000086978	protein_coding	rs29563926
5:92006271-92006271	downstream_gene_variant	MODIFIER	Cdkl2	ENSMUSG00000029403	Transcript	ENSMUST00000113140	protein_coding	rs29563926
5:92006271-92006271	downstream_gene_variant	MODIFIER	Cdkl2	ENSMUSG00000029403	Transcript	ENSMUST00000113143	protein_coding	rs29563926
5:92006271-92006271	downstream_gene_variant	MODIFIER	Cdkl2	ENSMUSG00000029403	Transcript	ENSMUST00000136037	retained_intron	rs29563926
5:92006881-92006881	3_prime_UTR_variant	MODIFIER	Cdkl2	ENSMUSG00000029403	Transcript	ENSMUST00000069937	protein_coding	rs31762827

5:92006881-92006881	3_prime_UTR_variant	MODIFIER	Cdkl2	ENSMUSG00000029403	Transcript	ENSMUST00000086978	protein_coding	rs31762827
5:92006881-92006881	downstream_gene_variant	MODIFIER	Cdkl2	ENSMUSG00000029403	Transcript	ENSMUST00000113140	protein_coding	rs31762827
5:92006881-92006881	downstream_gene_variant	MODIFIER	Cdkl2	ENSMUSG00000029403	Transcript	ENSMUST00000113143	protein_coding	rs31762827
5:92006881-92006881	downstream_gene_variant	MODIFIER	Cdkl2	ENSMUSG00000029403	Transcript	ENSMUST00000136037	retained_intron	rs31762827
5:92245584-92245584	intron_variant	MODIFIER	Ppef2	ENSMUSG00000029410	Transcript	ENSMUST00000031359	protein_coding	rs29779648
5:92245584-92245584	intron_variant	MODIFIER	Ppef2	ENSMUSG00000029410	Transcript	ENSMUST00000201130	protein_coding	rs29779648
5:92245584-92245584	regulatory_region_variant	MODIFIER	-	-	RegulatoryFeature	ENSMUSR00000684142	TF_binding_site	rs29779648
5:92258968-92258968	intron_variant	MODIFIER	Naaa	ENSMUSG00000029413	Transcript	ENSMUST00000113102	protein_coding	rs29677929
5:92258968-92258968	intron_variant	MODIFIER	Naaa	ENSMUSG00000029413	Transcript	ENSMUST00000159345	protein_coding	rs29677929
5:92258968-92258968	intron_variant,non_coding_trans	MODIFIER	Naaa	ENSMUSG00000029413	Transcript	ENSMUST00000159732	retained_intron	rs29677929
5:92258968-92258968	downstream_gene_variant	MODIFIER	Naaa	ENSMUSG00000029413	Transcript	ENSMUST00000175656	protein_coding	rs29677929
5:92258968-92258968	upstream_gene_variant	MODIFIER	Ppef2	ENSMUSG00000029410	Transcript	ENSMUST00000201130	protein_coding	rs29677929
5:92264236-92264236	intron_variant	MODIFIER	Naaa	ENSMUSG00000029413	Transcript	ENSMUST00000113102	protein_coding	rs31777069
5:92264236-92264236	intron_variant	MODIFIER	Naaa	ENSMUSG00000029413	Transcript	ENSMUST00000159345	protein_coding	rs31777069
5:92264236-92264236	upstream_gene_variant	MODIFIER	Naaa	ENSMUSG00000029413	Transcript	ENSMUST00000159732	retained_intron	rs31777069
5:92264236-92264236	intron_variant	MODIFIER	Naaa	ENSMUSG00000029413	Transcript	ENSMUST00000175656	protein_coding	rs31777069
5:92264236-92264236	regulatory_region_variant	MODIFIER	-	-	RegulatoryFeature	ENSMUSR00000425112	promoter_flanking_region	rs31777069
5:92291342-92291342	intron_variant	MODIFIER	Sdad1	ENSMUSG00000029415	Transcript	ENSMUST00000031364	protein_coding	rs33500095
5:92291342-92291342	downstream_gene_variant	MODIFIER	Sdad1	ENSMUSG00000029415	Transcript	ENSMUST00000201084	retained_intron	rs33500095
5:92291342-92291342	intron_variant	MODIFIER	Sdad1	ENSMUSG00000029415	Transcript	ENSMUST00000201143	protein_coding	rs33500095
5:92291342-92291342	intron_variant,non_coding_trans	MODIFIER	Gm43599	ENSMUSG00000106698	Transcript	ENSMUST00000202680	antisense	rs33500095
5:92291342-92291342	intron_variant,non_coding_trans	MODIFIER	Sdad1	ENSMUSG00000029415	Transcript	ENSMUST00000202903	retained_intron	rs33500095
5:92295842-92295842	intron_variant	MODIFIER	Sdad1	ENSMUSG00000029415	Transcript	ENSMUST00000031364	protein_coding	rs33590216
5:92295842-92295842	intron_variant,non_coding_trans	MODIFIER	Sdad1	ENSMUSG00000029415	Transcript	ENSMUST00000201084	retained_intron	rs33590216
5:92295842-92295842	intron_variant	MODIFIER	Sdad1	ENSMUSG00000029415	Transcript	ENSMUST00000201143	protein_coding	rs33590216
5:92295842-92295842	intron_variant,non_coding_trans	MODIFIER	Gm43599	ENSMUSG00000106698	Transcript	ENSMUST00000202680	antisense	rs33590216
5:92295842-92295842	intron_variant,non_coding_trans	MODIFIER	Sdad1	ENSMUSG00000029415	Transcript	ENSMUST00000202903	retained_intron	rs33590216
5:92300340-92300340	intron_variant	MODIFIER	Sdad1	ENSMUSG00000029415	Transcript	ENSMUST00000031364	protein_coding	rs31777314
5:92300340-92300340	upstream_gene_variant	MODIFIER	Gm23031	ENSMUSG00000088623	Transcript	ENSMUST00000157998	snRNA	rs31777314
5:92300340-92300340	upstream_gene_variant	MODIFIER	Sdad1	ENSMUSG00000029415	Transcript	ENSMUST00000201084	retained_intron	rs31777314
5:92300340-92300340	intron_variant	MODIFIER	Sdad1	ENSMUSG00000029415	Transcript	ENSMUST00000201143	protein_coding	rs31777314
5:92300340-92300340	downstream_gene_variant	MODIFIER	Sdad1	ENSMUSG00000029415	Transcript	ENSMUST00000201532	retained_intron	rs31777314
5:92300340-92300340	downstream_gene_variant	MODIFIER	Sdad1	ENSMUSG00000029415	Transcript	ENSMUST00000202604	retained_intron	rs31777314
5:92300340-92300340	intron_variant,non_coding_trans	MODIFIER	Gm43599	ENSMUSG00000106698	Transcript	ENSMUST00000202680	antisense	rs31777314
5:92300340-92300340	upstream_gene_variant	MODIFIER	Sdad1	ENSMUSG00000029415	Transcript	ENSMUST00000202903	retained_intron	rs31777314
5:92605942-92605942	3_prime_UTR_variant	MODIFIER	Stbd1	ENSMUSG00000047963	Transcript	ENSMUST00000050952	protein_coding	rs33292414
5:92605942-92605942	downstream_gene_variant	MODIFIER	Ccdc158	ENSMUSG00000050050	Transcript	ENSMUST00000060930	protein_coding	rs33292414
5:92605942-92605942	downstream_gene_variant	MODIFIER	Ccdc158	ENSMUSG00000050050	Transcript	ENSMUST00000136004	retained_intron	rs33292414
5:92605942-92605942	downstream_gene_variant	MODIFIER	Stbd1	ENSMUSG00000047963	Transcript	ENSMUST00000200941	protein_coding	rs33292414
5:92605942-92605942	3_prime_UTR_variant,NMD_trar	MODIFIER	Stbd1	ENSMUSG00000047963	Transcript	ENSMUST00000202332	nonsense_mediated_decay	rs33292414
5:93454688-93454688	intergenic_variant	MODIFIER	-	-	-	-	-	rs33148943
5:93454694-93454694	intergenic_variant	MODIFIER	-	-	-	-	-	rs232102783
5:93455633-93455633	intergenic_variant	MODIFIER	-	-	-	-	-	rs238535280
5:95512859-95512859	upstream_gene_variant	MODIFIER	Gm3286	ENSMUSG00000079423	Transcript	ENSMUST00000200932	protein_coding	rs587503537
5:95512859-95512859	intron_variant	MODIFIER	Gm3286	ENSMUSG00000079423	Transcript	ENSMUST00000201629	protein_coding	rs587503537
5:95512860-95512860	upstream_gene_variant	MODIFIER	Gm3286	ENSMUSG00000079423	Transcript	ENSMUST00000200932	protein_coding	rs580584335
5:95512860-95512860	intron_variant	MODIFIER	Gm3286	ENSMUSG00000079423	Transcript	ENSMUST00000201629	protein_coding	rs580584335
5:96936580-96936580	upstream_gene_variant	MODIFIER	Gm43148	ENSMUSG00000105377	Transcript	ENSMUST00000198237	TEC	rs52285004
5:96936580-96936580	upstream_gene_variant	MODIFIER	Gm43144	ENSMUSG00000105257	Transcript	ENSMUST00000198256	TEC	rs52285004
5:96936580-96936580	downstream_gene_variant	MODIFIER	Gm8013	ENSMUSG00000106357	Transcript	ENSMUST00000200139	lincRNA	rs52285004
5:97083040-97083040	intron_variant	MODIFIER	Bmp2k	ENSMUSG00000034663	Transcript	ENSMUST00000035635	protein_coding	rs33075055
5:97083040-97083040	intron_variant	MODIFIER	Paqr3	ENSMUSG00000055725	Transcript	ENSMUST00000069453	protein_coding	rs33075055
5:97083040-97083040	downstream_gene_variant	MODIFIER	Paqr3	ENSMUSG00000055725	Transcript	ENSMUST00000112969	protein_coding	rs33075055
6:120905615-120905615	intron_variant	MODIFIER	Bid	ENSMUSG00000004446	Transcript	ENSMUST00000004560	protein_coding	rs47662882
6:120905615-120905615	downstream_gene_variant	MODIFIER	Bid	ENSMUSG00000004446	Transcript	ENSMUST00000125915	processed_transcript	rs47662882
6:120905615-120905615	intron_variant	MODIFIER	Bid	ENSMUSG00000004446	Transcript	ENSMUST00000145948	protein_coding	rs47662882
6:120905615-120905615	intron_variant,NMD_transcript_v	MODIFIER	Bid	ENSMUSG00000004446	Transcript	ENSMUST00000160684	nonsense_mediated_decay	rs47662882
6:120905615-120905615	upstream_gene_variant	MODIFIER	Gm17812	ENSMUSG00000108125	Transcript	ENSMUST00000204935	processed_pseudogene	rs47662882
6:128406115-128406115	downstream_gene_variant	MODIFIER	Itfg2	ENSMUSG00000001518	Transcript	ENSMUST00000001559	protein_coding	rs29825836
6:128406115-128406115	intron_variant	MODIFIER	Nrip2	ENSMUSG00000001520	Transcript	ENSMUST00000001561	protein_coding	rs29825836
6:128406115-128406115	intron_variant	MODIFIER	Nrip2	ENSMUSG00000001520	Transcript	ENSMUST00000120405	protein_coding	rs29825836
6:128406115-128406115	intron_variant	MODIFIER	Nrip2	ENSMUSG00000001520	Transcript	ENSMUST00000123867	protein_coding	rs29825836
6:128406115-128406115	non_coding_transcript_exon_vai	MODIFIER	Nrip2	ENSMUSG00000001520	Transcript	ENSMUST00000136631	retained_intron	rs29825836
6:128406115-128406115	downstream_gene_variant	MODIFIER	Itfg2	ENSMUSG00000001518	Transcript	ENSMUST00000142615	protein_coding	rs29825836
6:128406115-128406115	intron_variant,NMD_transcript_v	MODIFIER	Nrip2	ENSMUSG00000001520	Transcript	ENSMUST00000147155	nonsense_mediated_decay	rs29825836
6:128406115-128406115	downstream_gene_variant	MODIFIER	Itfg2	ENSMUSG00000001518	Transcript	ENSMUST00000203374	protein_coding	rs29825836
6:128406115-128406115	downstream_gene_variant	MODIFIER	Itfg2	ENSMUSG00000001518	Transcript	ENSMUST00000203984	protein_coding	rs29825836
6:128406115-128406115	intron_variant	MODIFIER	Gm44596	ENSMUSG00000108011	Transcript	ENSMUST00000204223	protein_coding	rs29825836
6:128406115-128406115	downstream_gene_variant	MODIFIER	Itfg2	ENSMUSG00000001518	Transcript	ENSMUST00000204362	retained_intron	rs29825836

6:128406115-128406115	intron_variant	MODIFIER	Nrip2	ENSMUSG00000001520	Transcript	ENSMUST00000204836	non_stop_decay	rs29825836
6:128407798-128407798	downstream_gene_variant	MODIFIER	Itfg2	ENSMUSG00000001518	Transcript	ENSMUST00000001559	protein_coding	rs226579891
6:128407798-128407798	intron_variant	MODIFIER	Nrip2	ENSMUSG00000001520	Transcript	ENSMUST00000001561	protein_coding	rs226579891
6:128407798-128407798	intron_variant	MODIFIER	Nrip2	ENSMUSG00000001520	Transcript	ENSMUST00000120405	protein_coding	rs226579891
6:128407798-128407798	intron_variant	MODIFIER	Nrip2	ENSMUSG00000001520	Transcript	ENSMUST00000123867	protein_coding	rs226579891
6:128407798-128407798	non_coding_transcript_exon_va	MODIFIER	Nrip2	ENSMUSG00000001520	Transcript	ENSMUST00000136631	retained_intron	rs226579891
6:128407798-128407798	downstream_gene_variant	MODIFIER	Itfg2	ENSMUSG00000001518	Transcript	ENSMUST00000142615	protein_coding	rs226579891
6:128407798-128407798	intron_variant,NMD_transcript_v	MODIFIER	Nrip2	ENSMUSG00000001520	Transcript	ENSMUST00000147155	nonsense_mediated_decay	rs226579891
6:128407798-128407798	downstream_gene_variant	MODIFIER	Itfg2	ENSMUSG00000001518	Transcript	ENSMUST00000203195	retained_intron	rs226579891
6:128407798-128407798	downstream_gene_variant	MODIFIER	Itfg2	ENSMUSG00000001518	Transcript	ENSMUST00000203374	protein_coding	rs226579891
6:128407798-128407798	downstream_gene_variant	MODIFIER	Itfg2	ENSMUSG00000001518	Transcript	ENSMUST00000203984	protein_coding	rs226579891
6:128407798-128407798	downstream_gene_variant	MODIFIER	Gm44596	ENSMUSG00000108011	Transcript	ENSMUST00000204223	protein_coding	rs226579891
6:128407798-128407798	downstream_gene_variant	MODIFIER	Itfg2	ENSMUSG00000001518	Transcript	ENSMUST00000204362	retained_intron	rs226579891
6:128407798-128407798	intron_variant	MODIFIER	Nrip2	ENSMUSG00000001520	Transcript	ENSMUST00000204836	non_stop_decay	rs226579891
6:129007865-129007865	splice_acceptor_variant,non_co	HIGH	BC064078	ENSMUSG00000087150	Transcript	ENSMUST00000122939	transcribed_unprocessed_pseu	rs50369298
6:129007865-129007865	non_coding_transcript_exon_va	MODIFIER	BC064078	ENSMUSG00000087150	Transcript	ENSMUST00000131176	processed_transcript	rs50369298
6:129056293-129056293	intron_variant	MODIFIER	Klrb1f	ENSMUSG00000030154	Transcript	ENSMUST00000032257	protein_coding	rs30747537
6:129056293-129056293	intron_variant,NMD_transcript_v	MODIFIER	Klrb1f	ENSMUSG00000030154	Transcript	ENSMUST00000203059	nonsense_mediated_decay	rs30747537
6:129056293-129056293	downstream_gene_variant	MODIFIER	Klrb1f	ENSMUSG00000030154	Transcript	ENSMUST00000204320	protein_coding	rs30747537
6:129056293-129056293	downstream_gene_variant	MODIFIER	Klrb1f	ENSMUSG00000030154	Transcript	ENSMUST00000204413	retained_intron	rs30747537
6:129056293-129056293	intron_variant,NMD_transcript_v	MODIFIER	Klrb1f	ENSMUSG00000030154	Transcript	ENSMUST00000204508	nonsense_mediated_decay	rs30747537
6:129056293-129056293	downstream_gene_variant	MODIFIER	Klrb1f	ENSMUSG00000030154	Transcript	ENSMUST00000204784	processed_transcript	rs30747537
6:129057550-129057550	downstream_gene_variant	MODIFIER	Klrb1f	ENSMUSG00000030154	Transcript	ENSMUST00000032257	protein_coding	rs37293589
6:129057550-129057550	downstream_gene_variant	MODIFIER	Klrb1f	ENSMUSG00000030154	Transcript	ENSMUST00000203059	nonsense_mediated_decay	rs37293589
6:129057550-129057550	downstream_gene_variant	MODIFIER	Klrb1f	ENSMUSG00000030154	Transcript	ENSMUST00000204320	protein_coding	rs37293589
6:129057550-129057550	downstream_gene_variant	MODIFIER	Klrb1f	ENSMUSG00000030154	Transcript	ENSMUST00000204413	retained_intron	rs37293589
6:129057550-129057550	downstream_gene_variant	MODIFIER	Klrb1f	ENSMUSG00000030154	Transcript	ENSMUST00000204508	nonsense_mediated_decay	rs37293589
6:129057550-129057550	downstream_gene_variant	MODIFIER	Klrb1f	ENSMUSG00000030154	Transcript	ENSMUST00000204784	processed_transcript	rs37293589
6:129063538-129063538	intergenic_variant	MODIFIER	-	-	-	-	-	rs1132095552
6:129649575-129649575	3_prime_UTR_variant	MODIFIER	Klrc2	ENSMUSG00000052736	Transcript	ENSMUST00000071920	protein_coding	rs387543454
6:129649575-129649575	3_prime_UTR_variant	MODIFIER	Klrc2	ENSMUSG00000052736	Transcript	ENSMUST00000112057	protein_coding	rs387543454
6:129649575-129649575	3_prime_UTR_variant	MODIFIER	Klrc2	ENSMUSG00000052736	Transcript	ENSMUST00000145984	protein_coding	rs387543454
6:129874907-129874907	start_lost	HIGH	Klra17	ENSMUSG00000014543	Transcript	ENSMUST00000014687	protein_coding	rs1133423676
6:129874907-129874907	start_lost	HIGH	Klra17	ENSMUSG00000014543	Transcript	ENSMUST00000122219	protein_coding	rs1133423676
6:129874909-129874909	5_prime_UTR_variant	MODIFIER	Klra17	ENSMUSG00000014543	Transcript	ENSMUST00000014687	protein_coding	rs243351661
6:129874909-129874909	5_prime_UTR_variant	MODIFIER	Klra17	ENSMUSG00000014543	Transcript	ENSMUST00000122219	protein_coding	rs243351661
6:129972403-129972403	non_coding_transcript_exon_va	MODIFIER	Gm15854	ENSMUSG00000083120	Transcript	ENSMUST00000118879	unprocessed_pseudogene	rs30944791
6:130377839-130377839	missense_variant	MODERATE	Klra1	ENSMUSG00000079853	Transcript	ENSMUST00000032288	protein_coding	rs1132962812
6:130377839-130377839	upstream_gene_variant	MODIFIER	Klra1	ENSMUSG00000079853	Transcript	ENSMUST00000127570	retained_intron	rs1132962812
6:134933456-134933456	intron_variant,non_coding_trans	MODIFIER	Lockd	ENSMUSG00000098318	Transcript	ENSMUST00000183867	lincRNA	rs47535441
6:134933456-134933456	intron_variant,non_coding_trans	MODIFIER	Lockd	ENSMUSG00000098318	Transcript	ENSMUST00000183905	lincRNA	rs47535441
6:134933456-134933456	intron_variant,non_coding_trans	MODIFIER	Lockd	ENSMUSG00000098318	Transcript	ENSMUST00000184504	lincRNA	rs47535441
6:134933456-134933456	intron_variant,non_coding_trans	MODIFIER	Lockd	ENSMUSG00000098318	Transcript	ENSMUST00000184991	lincRNA	rs47535441
6:134933456-134933456	intron_variant,non_coding_trans	MODIFIER	Lockd	ENSMUSG00000098318	Transcript	ENSMUST00000185152	lincRNA	rs47535441
6:134933456-134933456	upstream_gene_variant	MODIFIER	Gm44238	ENSMUSG00000107730	Transcript	ENSMUST00000203300	lincRNA	rs47535441
6:134984732-134984732	3_prime_UTR_variant	MODIFIER	Apold1	ENSMUSG00000090698	Transcript	ENSMUST00000167323	protein_coding	rs30745732
6:134984732-134984732	regulatory_region_variant	MODIFIER	-	-	RegulatoryFeature	ENSMUSR00000236726	promoter	rs30745732
6:135021780-135021780	intron_variant	MODIFIER	Ddx47	ENSMUSG00000030204	Transcript	ENSMUST00000032326	protein_coding	rs37083956
6:135021780-135021780	downstream_gene_variant	MODIFIER	Ddx47	ENSMUSG00000030204	Transcript	ENSMUST00000123022	processed_transcript	rs37083956
6:135021780-135021780	downstream_gene_variant	MODIFIER	Ddx47	ENSMUSG00000030204	Transcript	ENSMUST00000130851	protein_coding	rs37083956
6:135021780-135021780	downstream_gene_variant	MODIFIER	Ddx47	ENSMUSG00000030204	Transcript	ENSMUST00000134549	retained_intron	rs37083956
6:135021780-135021780	downstream_gene_variant	MODIFIER	Ddx47	ENSMUSG00000030204	Transcript	ENSMUST00000154558	protein_coding	rs37083956
6:135021780-135021780	3_prime_UTR_variant	MODIFIER	Ddx47	ENSMUSG00000030204	Transcript	ENSMUST00000155022	protein_coding	rs37083956
6:135021780-135021780	intron_variant,non_coding_trans	MODIFIER	Ddx47	ENSMUSG00000030204	Transcript	ENSMUST00000155277	retained_intron	rs37083956
6:135021780-135021780	downstream_gene_variant	MODIFIER	Ddx47	ENSMUSG00000030204	Transcript	ENSMUST00000204646	protein_coding	rs37083956
6:135021780-135021780	downstream_gene_variant	MODIFIER	Ddx47	ENSMUSG00000030204	Transcript	ENSMUST00000205055	protein_coding	rs37083956
6:135021780-135021780	downstream_gene_variant	MODIFIER	Ddx47	ENSMUSG00000030204	Transcript	ENSMUST00000205244	protein_coding	rs37083956
6:136815967-136815967	intron_variant	MODIFIER	Wbp11	ENSMUSG00000030216	Transcript	ENSMUST00000116514	protein_coding	rs36710304
6:136815967-136815967	downstream_gene_variant	MODIFIER	Wbp11	ENSMUSG00000030216	Transcript	ENSMUST00000151333	retained_intron	rs36710304
6:136815967-136815967	downstream_gene_variant	MODIFIER	Wbp11	ENSMUSG00000030216	Transcript	ENSMUST00000204129	protein_coding	rs36710304
6:136815967-136815967	downstream_gene_variant	MODIFIER	Wbp11	ENSMUSG00000030216	Transcript	ENSMUST00000204272	nonsense_mediated_decay	rs36710304
6:136820052-136820052	intron_variant	MODIFIER	Wbp11	ENSMUSG00000030216	Transcript	ENSMUST00000116514	protein_coding	rs30564926
6:136820052-136820052	downstream_gene_variant	MODIFIER	Wbp11	ENSMUSG00000030216	Transcript	ENSMUST00000141598	retained_intron	rs30564926
6:136820052-136820052	non_coding_transcript_exon_va	MODIFIER	Wbp11	ENSMUSG00000030216	Transcript	ENSMUST00000151333	retained_intron	rs30564926
6:136820052-136820052	intron_variant	MODIFIER	Wbp11	ENSMUSG00000030216	Transcript	ENSMUST00000204129	protein_coding	rs30564926
6:136820052-136820052	intron_variant,NMD_transcript_v	MODIFIER	Wbp11	ENSMUSG00000030216	Transcript	ENSMUST00000204272	nonsense_mediated_decay	rs30564926
6:137419769-137419769	intron_variant	MODIFIER	Ptpro	ENSMUSG00000030223	Transcript	ENSMUST00000077115	protein_coding	rs579307928
6:137419769-137419769	intron_variant	MODIFIER	Ptpro	ENSMUSG00000030223	Transcript	ENSMUST00000167002	protein_coding	rs579307928
6:137419769-137419769	intron_variant	MODIFIER	Ptpro	ENSMUSG00000030223	Transcript	ENSMUST00000167679	protein_coding	rs579307928

6:137419769-137419769	intron_variant	MODIFIER	Ptpro	ENSMUSG00000030223	Transcript	ENSMUST00000203914	protein_coding	rs579307928
6:137419770-137419770	intron_variant	MODIFIER	Ptpro	ENSMUSG00000030223	Transcript	ENSMUST00000077115	protein_coding	rs249172707
6:137419770-137419770	intron_variant	MODIFIER	Ptpro	ENSMUSG00000030223	Transcript	ENSMUST00000167002	protein_coding	rs249172707
6:137419770-137419770	intron_variant	MODIFIER	Ptpro	ENSMUSG00000030223	Transcript	ENSMUST00000167679	protein_coding	rs249172707
6:137419770-137419770	intron_variant	MODIFIER	Ptpro	ENSMUSG00000030223	Transcript	ENSMUST00000203914	protein_coding	rs249172707
6:137420641-137420641	intron_variant	MODIFIER	Ptpro	ENSMUSG00000030223	Transcript	ENSMUST00000077115	protein_coding	rs30949757
6:137420641-137420641	intron_variant	MODIFIER	Ptpro	ENSMUSG00000030223	Transcript	ENSMUST00000167002	protein_coding	rs30949757
6:137420641-137420641	intron_variant	MODIFIER	Ptpro	ENSMUSG00000030223	Transcript	ENSMUST00000167679	protein_coding	rs30949757
6:137420641-137420641	intron_variant	MODIFIER	Ptpro	ENSMUSG00000030223	Transcript	ENSMUST00000203914	protein_coding	rs30949757
6:137420642-137420642	intron_variant	MODIFIER	Ptpro	ENSMUSG00000030223	Transcript	ENSMUST00000077115	protein_coding	rs30271137
6:137420642-137420642	intron_variant	MODIFIER	Ptpro	ENSMUSG00000030223	Transcript	ENSMUST00000167002	protein_coding	rs30271137
6:137420642-137420642	intron_variant	MODIFIER	Ptpro	ENSMUSG00000030223	Transcript	ENSMUST00000167679	protein_coding	rs30271137
6:137420642-137420642	intron_variant	MODIFIER	Ptpro	ENSMUSG00000030223	Transcript	ENSMUST00000203914	protein_coding	rs30271137
6:137422397-137422397	intron_variant	MODIFIER	Ptpro	ENSMUSG00000030223	Transcript	ENSMUST00000077115	protein_coding	rs31941522
6:137422397-137422397	intron_variant	MODIFIER	Ptpro	ENSMUSG00000030223	Transcript	ENSMUST00000167002	protein_coding	rs31941522
6:137422397-137422397	intron_variant	MODIFIER	Ptpro	ENSMUSG00000030223	Transcript	ENSMUST00000167679	protein_coding	rs31941522
6:137422397-137422397	intron_variant	MODIFIER	Ptpro	ENSMUSG00000030223	Transcript	ENSMUST00000203914	protein_coding	rs31941522
6:137422713-137422713	intron_variant	MODIFIER	Ptpro	ENSMUSG00000030223	Transcript	ENSMUST00000077115	protein_coding	rs587022977
6:137422713-137422713	intron_variant	MODIFIER	Ptpro	ENSMUSG00000030223	Transcript	ENSMUST00000167002	protein_coding	rs587022977
6:137422713-137422713	intron_variant	MODIFIER	Ptpro	ENSMUSG00000030223	Transcript	ENSMUST00000167679	protein_coding	rs587022977
6:137422713-137422713	intron_variant	MODIFIER	Ptpro	ENSMUSG00000030223	Transcript	ENSMUST00000203914	protein_coding	rs587022977
6:137423088-137423088	intron_variant	MODIFIER	Ptpro	ENSMUSG00000030223	Transcript	ENSMUST00000077115	protein_coding	rs31943984
6:137423088-137423088	intron_variant	MODIFIER	Ptpro	ENSMUSG00000030223	Transcript	ENSMUST00000167002	protein_coding	rs31943984
6:137423088-137423088	intron_variant	MODIFIER	Ptpro	ENSMUSG00000030223	Transcript	ENSMUST00000167679	protein_coding	rs31943984
6:137423088-137423088	intron_variant	MODIFIER	Ptpro	ENSMUSG00000030223	Transcript	ENSMUST00000203914	protein_coding	rs31943984
6:137423107-137423107	intron_variant	MODIFIER	Ptpro	ENSMUSG00000030223	Transcript	ENSMUST00000077115	protein_coding	rs31943986
6:137423107-137423107	intron_variant	MODIFIER	Ptpro	ENSMUSG00000030223	Transcript	ENSMUST00000167002	protein_coding	rs31943986
6:137423107-137423107	intron_variant	MODIFIER	Ptpro	ENSMUSG00000030223	Transcript	ENSMUST00000167679	protein_coding	rs31943986
6:137423107-137423107	intron_variant	MODIFIER	Ptpro	ENSMUSG00000030223	Transcript	ENSMUST00000203914	protein_coding	rs31943986
6:137423131-137423131	intron_variant	MODIFIER	Ptpro	ENSMUSG00000030223	Transcript	ENSMUST00000077115	protein_coding	rs252820037
6:137423131-137423131	intron_variant	MODIFIER	Ptpro	ENSMUSG00000030223	Transcript	ENSMUST00000167002	protein_coding	rs252820037
6:137423131-137423131	intron_variant	MODIFIER	Ptpro	ENSMUSG00000030223	Transcript	ENSMUST00000167679	protein_coding	rs252820037
6:137423131-137423131	intron_variant	MODIFIER	Ptpro	ENSMUSG00000030223	Transcript	ENSMUST00000203914	protein_coding	rs252820037
6:137423173-137423173	intron_variant	MODIFIER	Ptpro	ENSMUSG00000030223	Transcript	ENSMUST00000077115	protein_coding	rs212810127
6:137423173-137423173	intron_variant	MODIFIER	Ptpro	ENSMUSG00000030223	Transcript	ENSMUST00000167002	protein_coding	rs212810127
6:137423173-137423173	intron_variant	MODIFIER	Ptpro	ENSMUSG00000030223	Transcript	ENSMUST00000167679	protein_coding	rs212810127
6:137423173-137423173	intron_variant	MODIFIER	Ptpro	ENSMUSG00000030223	Transcript	ENSMUST00000203914	protein_coding	rs212810127
6:137423198-137423198	intron_variant	MODIFIER	Ptpro	ENSMUSG00000030223	Transcript	ENSMUST00000077115	protein_coding	rs31943988
6:137423198-137423198	intron_variant	MODIFIER	Ptpro	ENSMUSG00000030223	Transcript	ENSMUST00000167002	protein_coding	rs31943988
6:137423198-137423198	intron_variant	MODIFIER	Ptpro	ENSMUSG00000030223	Transcript	ENSMUST00000167679	protein_coding	rs31943988
6:137423198-137423198	intron_variant	MODIFIER	Ptpro	ENSMUSG00000030223	Transcript	ENSMUST00000203914	protein_coding	rs31943988
6:137424005-137424005	intron_variant	MODIFIER	Ptpro	ENSMUSG00000030223	Transcript	ENSMUST00000077115	protein_coding	rs31947614
6:137424005-137424005	intron_variant	MODIFIER	Ptpro	ENSMUSG00000030223	Transcript	ENSMUST00000167002	protein_coding	rs31947614
6:137424005-137424005	intron_variant	MODIFIER	Ptpro	ENSMUSG00000030223	Transcript	ENSMUST00000167679	protein_coding	rs31947614
6:137424005-137424005	intron_variant	MODIFIER	Ptpro	ENSMUSG00000030223	Transcript	ENSMUST00000203914	protein_coding	rs31947614
6:137424288-137424288	intron_variant	MODIFIER	Ptpro	ENSMUSG00000030223	Transcript	ENSMUST00000077115	protein_coding	rs49800102
6:137424288-137424288	intron_variant	MODIFIER	Ptpro	ENSMUSG00000030223	Transcript	ENSMUST00000167002	protein_coding	rs49800102
6:137424288-137424288	intron_variant	MODIFIER	Ptpro	ENSMUSG00000030223	Transcript	ENSMUST00000167679	protein_coding	rs49800102
6:137424288-137424288	intron_variant	MODIFIER	Ptpro	ENSMUSG00000030223	Transcript	ENSMUST00000203914	protein_coding	rs49800102
6:137425748-137425748	intron_variant	MODIFIER	Ptpro	ENSMUSG00000030223	Transcript	ENSMUST00000077115	protein_coding	rs51677648
6:137425748-137425748	intron_variant	MODIFIER	Ptpro	ENSMUSG00000030223	Transcript	ENSMUST00000167002	protein_coding	rs51677648
6:137425748-137425748	intron_variant	MODIFIER	Ptpro	ENSMUSG00000030223	Transcript	ENSMUST00000167679	protein_coding	rs51677648
6:137425748-137425748	intron_variant	MODIFIER	Ptpro	ENSMUSG00000030223	Transcript	ENSMUST00000203914	protein_coding	rs51677648
6:137425748-137425748	regulatory_region_variant	MODIFIER	-	-	RegulatoryFeature	ENSMUSR00000709366	promoter_flanking_region	rs51677648
6:137425755-137425755	intron_variant	MODIFIER	Ptpro	ENSMUSG00000030223	Transcript	ENSMUST00000077115	protein_coding	rs51547743
6:137425755-137425755	intron_variant	MODIFIER	Ptpro	ENSMUSG00000030223	Transcript	ENSMUST00000167002	protein_coding	rs51547743
6:137425755-137425755	intron_variant	MODIFIER	Ptpro	ENSMUSG00000030223	Transcript	ENSMUST00000167679	protein_coding	rs51547743
6:137425755-137425755	intron_variant	MODIFIER	Ptpro	ENSMUSG00000030223	Transcript	ENSMUST00000203914	protein_coding	rs51547743
6:137425755-137425755	regulatory_region_variant	MODIFIER	-	-	RegulatoryFeature	ENSMUSR00000709366	promoter_flanking_region	rs51547743
6:137425859-137425859	intron_variant	MODIFIER	Ptpro	ENSMUSG00000030223	Transcript	ENSMUST00000077115	protein_coding	rs31943146
6:137425859-137425859	intron_variant	MODIFIER	Ptpro	ENSMUSG00000030223	Transcript	ENSMUST00000167002	protein_coding	rs31943146
6:137425859-137425859	intron_variant	MODIFIER	Ptpro	ENSMUSG00000030223	Transcript	ENSMUST00000167679	protein_coding	rs31943146
6:137425859-137425859	intron_variant	MODIFIER	Ptpro	ENSMUSG00000030223	Transcript	ENSMUST00000203914	protein_coding	rs31943146
6:137425859-137425859	regulatory_region_variant	MODIFIER	-	-	RegulatoryFeature	ENSMUSR00000709366	promoter_flanking_region	rs31943146
6:137425866-137425866	intron_variant	MODIFIER	Ptpro	ENSMUSG00000030223	Transcript	ENSMUST00000077115	protein_coding	rs48228779
6:137425866-137425866	intron_variant	MODIFIER	Ptpro	ENSMUSG00000030223	Transcript	ENSMUST00000167002	protein_coding	rs48228779
6:137425866-137425866	intron_variant	MODIFIER	Ptpro	ENSMUSG00000030223	Transcript	ENSMUST00000167679	protein_coding	rs48228779
6:137425866-137425866	intron_variant	MODIFIER	Ptpro	ENSMUSG00000030223	Transcript	ENSMUST00000203914	protein_coding	rs48228779
6:137425866-137425866	regulatory_region_variant	MODIFIER	-	-	RegulatoryFeature	ENSMUSR00000709366	promoter_flanking_region	rs48228779

6:137426834-137426834	intron_variant	MODIFIER	Ptpro	ENSMUSG00000030223	Transcript	ENSMUST00000077115	protein_coding	rs31945198
6:137426834-137426834	intron_variant	MODIFIER	Ptpro	ENSMUSG00000030223	Transcript	ENSMUST00000167002	protein_coding	rs31945198
6:137426834-137426834	intron_variant	MODIFIER	Ptpro	ENSMUSG00000030223	Transcript	ENSMUST00000167679	protein_coding	rs31945198
6:137426834-137426834	intron_variant	MODIFIER	Ptpro	ENSMUSG00000030223	Transcript	ENSMUST00000203914	protein_coding	rs31945198
6:137426834-137426834	upstream_gene_variant	MODIFIER	Gm44258	ENSMUSG00000107759	Transcript	ENSMUST00000204925	sense_intronic	rs31945198
6:137456368-137456368	intron_variant	MODIFIER	Ptpro	ENSMUSG00000030223	Transcript	ENSMUST00000077115	protein_coding	rs263957631
6:137456368-137456368	intron_variant	MODIFIER	Ptpro	ENSMUSG00000030223	Transcript	ENSMUST00000167002	protein_coding	rs263957631
6:137456368-137456368	intron_variant	MODIFIER	Ptpro	ENSMUSG00000030223	Transcript	ENSMUST00000167679	protein_coding	rs263957631
6:137456368-137456368	intron_variant,non_coding_trans	MODIFIER	Ptpro	ENSMUSG00000030223	Transcript	ENSMUST00000203127	processed_transcript	rs263957631
6:137456368-137456368	intron_variant,non_coding_trans	MODIFIER	Ptpro	ENSMUSG00000030223	Transcript	ENSMUST00000203255	processed_transcript	rs263957631
6:137456368-137456368	intron_variant	MODIFIER	Ptpro	ENSMUSG00000030223	Transcript	ENSMUST00000203914	protein_coding	rs263957631
6:137456368-137456368	regulatory_region_variant	MODIFIER	-	-	RegulatoryFeature	ENSMUSR00000237069	promoter_flanking_region	rs263957631
6:137456368-137456368	regulatory_region_variant	MODIFIER	-	-	RegulatoryFeature	ENSMUSR00000440728	CTCF_binding_site	rs263957631
6:137591460-137591460	5_prime_UTR_variant	MODIFIER	Eps8	ENSMUSG00000015766	Transcript	ENSMUST00000058210	protein_coding	rs31961149
6:137591460-137591460	intron_variant	MODIFIER	Eps8	ENSMUSG00000015766	Transcript	ENSMUST00000111878	protein_coding	rs31961149
6:137591460-137591460	5_prime_UTR_variant	MODIFIER	Eps8	ENSMUSG00000015766	Transcript	ENSMUST00000132920	protein_coding	rs31961149
6:137591460-137591460	5_prime_UTR_variant	MODIFIER	Eps8	ENSMUSG00000015766	Transcript	ENSMUST00000134630	protein_coding	rs31961149
6:137591460-137591460	5_prime_UTR_variant	MODIFIER	Eps8	ENSMUSG00000015766	Transcript	ENSMUST00000139753	protein_coding	rs31961149
6:137591460-137591460	upstream_gene_variant	MODIFIER	Eps8	ENSMUSG00000015766	Transcript	ENSMUST00000146442	protein_coding	rs31961149
6:137591460-137591460	5_prime_UTR_variant	MODIFIER	Eps8	ENSMUSG00000015766	Transcript	ENSMUST00000147526	protein_coding	rs31961149
6:137591460-137591460	non_coding_transcript_exon_va	MODIFIER	Eps8	ENSMUSG00000015766	Transcript	ENSMUST00000153933	processed_transcript	rs31961149
6:137591460-137591460	downstream_gene_variant	MODIFIER	Gm44106	ENSMUSG00000107878	Transcript	ENSMUST00000203153	TEC	rs31961149
6:49060548-49060548	downstream_gene_variant	MODIFIER	Gpnmb	ENSMUSG00000029816	Transcript	ENSMUST00000031840	protein_coding	-
6:49060548-49060548	intron_variant	MODIFIER	Gpnmb	ENSMUSG00000029816	Transcript	ENSMUST00000204260	protein_coding	-
6:49060650-49060650	downstream_gene_variant	MODIFIER	Gpnmb	ENSMUSG00000029816	Transcript	ENSMUST00000031840	protein_coding	-
6:49060650-49060650	intron_variant	MODIFIER	Gpnmb	ENSMUSG00000029816	Transcript	ENSMUST00000204260	protein_coding	-
7:104422219-104422221	intron_variant	MODIFIER	Trim30a	ENSMUSG00000030921	Transcript	ENSMUST00000076922	protein_coding	rs240281407
7:104422219-104422221	upstream_gene_variant	MODIFIER	Gm25405	ENSMUSG00000064907	Transcript	ENSMUST00000082973	snRNA	rs240281407
7:131560495-131560495	downstream_gene_variant	MODIFIER	Hmx2	ENSMUSG00000050100	Transcript	ENSMUST00000051997	protein_coding	rs220532262
7:131560495-131560495	intron_variant	MODIFIER	Bub3	ENSMUSG00000066979	Transcript	ENSMUST00000084502	protein_coding	rs220532262
7:131560495-131560495	intron_variant	MODIFIER	Fgfr2	ENSMUSG00000030849	Transcript	ENSMUST00000124096	protein_coding	rs220532262
7:131560495-131560495	downstream_gene_variant	MODIFIER	Hmx2	ENSMUSG00000050100	Transcript	ENSMUST00000183219	protein_coding	rs220532262
7:131560495-131560495	intron_variant	MODIFIER	Bub3	ENSMUSG00000066979	Transcript	ENSMUST00000207231	protein_coding	rs220532262
7:131560495-131560495	upstream_gene_variant	MODIFIER	Bub3	ENSMUSG00000066979	Transcript	ENSMUST00000207442	protein_coding	rs220532262
7:131560495-131560495	intron_variant,NMD_transcript_v	MODIFIER	Bub3	ENSMUSG00000066979	Transcript	ENSMUST00000207736	nonsense_mediated_decay	rs220532262
7:131560495-131560495	upstream_gene_variant	MODIFIER	Bub3	ENSMUSG00000066979	Transcript	ENSMUST00000208571	protein_coding	rs220532262
7:131560495-131560495	intron_variant,non_coding_trans	MODIFIER	Bub3	ENSMUSG00000066979	Transcript	ENSMUST00000208848	retained_intron	rs220532262
7:131560495-131560495	non_coding_transcript_exon_va	MODIFIER	Bub3	ENSMUSG00000066979	Transcript	ENSMUST00000209131	retained_intron	rs220532262
7:131560495-131560495	regulatory_region_variant	MODIFIER	-	-	RegulatoryFeature	ENSMUSR00000252764	promoter	rs220532262
7:131563071-131563071	intron_variant	MODIFIER	Bub3	ENSMUSG00000066979	Transcript	ENSMUST00000084502	protein_coding	rs107706529
7:131563071-131563071	intron_variant	MODIFIER	Fgfr2	ENSMUSG00000030849	Transcript	ENSMUST00000124096	protein_coding	rs107706529
7:131563071-131563071	intron_variant	MODIFIER	Bub3	ENSMUSG00000066979	Transcript	ENSMUST00000207231	protein_coding	rs107706529
7:131563071-131563071	intron_variant	MODIFIER	Bub3	ENSMUSG00000066979	Transcript	ENSMUST00000207442	protein_coding	rs107706529
7:131563071-131563071	intron_variant,NMD_transcript_v	MODIFIER	Bub3	ENSMUSG00000066979	Transcript	ENSMUST00000207736	nonsense_mediated_decay	rs107706529
7:131563071-131563071	intron_variant	MODIFIER	Bub3	ENSMUSG00000066979	Transcript	ENSMUST00000208571	protein_coding	rs107706529
7:131563071-131563071	downstream_gene_variant	MODIFIER	Bub3	ENSMUSG00000066979	Transcript	ENSMUST00000208848	retained_intron	rs107706529
7:131563071-131563071	non_coding_transcript_exon_va	MODIFIER	Bub3	ENSMUSG00000066979	Transcript	ENSMUST00000209131	retained_intron	rs107706529
7:131563086-131563086	intron_variant	MODIFIER	Bub3	ENSMUSG00000066979	Transcript	ENSMUST00000084502	protein_coding	rs31508888
7:131563086-131563086	intron_variant	MODIFIER	Fgfr2	ENSMUSG00000030849	Transcript	ENSMUST00000124096	protein_coding	rs31508888
7:131563086-131563086	intron_variant	MODIFIER	Bub3	ENSMUSG00000066979	Transcript	ENSMUST00000207231	protein_coding	rs31508888
7:131563086-131563086	intron_variant	MODIFIER	Bub3	ENSMUSG00000066979	Transcript	ENSMUST00000207442	protein_coding	rs31508888
7:131563086-131563086	intron_variant,NMD_transcript_v	MODIFIER	Bub3	ENSMUSG00000066979	Transcript	ENSMUST00000207736	nonsense_mediated_decay	rs31508888
7:131563086-131563086	intron_variant	MODIFIER	Bub3	ENSMUSG00000066979	Transcript	ENSMUST00000208571	protein_coding	rs31508888
7:131563086-131563086	downstream_gene_variant	MODIFIER	Bub3	ENSMUSG00000066979	Transcript	ENSMUST00000208848	retained_intron	rs31508888
7:131563086-131563086	non_coding_transcript_exon_va	MODIFIER	Bub3	ENSMUSG00000066979	Transcript	ENSMUST00000209131	retained_intron	rs31508888
7:131573423-131573423	downstream_gene_variant	MODIFIER	Bub3	ENSMUSG00000066979	Transcript	ENSMUST00000084502	protein_coding	rs47203548
7:131573423-131573423	intron_variant	MODIFIER	Fgfr2	ENSMUSG00000030849	Transcript	ENSMUST00000124096	protein_coding	rs47203548
7:131573423-131573423	downstream_gene_variant	MODIFIER	Bub3	ENSMUSG00000066979	Transcript	ENSMUST00000207442	protein_coding	rs47203548
7:131573423-131573423	downstream_gene_variant	MODIFIER	Bub3	ENSMUSG00000066979	Transcript	ENSMUST00000207736	nonsense_mediated_decay	rs47203548
7:131573423-131573423	downstream_gene_variant	MODIFIER	Bub3	ENSMUSG00000066979	Transcript	ENSMUST00000208571	protein_coding	rs47203548
7:131573423-131573423	downstream_gene_variant	MODIFIER	Bub3	ENSMUSG00000066979	Transcript	ENSMUST00000209131	retained_intron	rs47203548
7:139426540-139426540	intron_variant	MODIFIER	Inpp5a	ENSMUSG00000025477	Transcript	ENSMUST00000026550	protein_coding	-
7:139426540-139426540	intron_variant	MODIFIER	Inpp5a	ENSMUSG00000025477	Transcript	ENSMUST00000097975	protein_coding	-
7:139426540-139426540	intron_variant	MODIFIER	Inpp5a	ENSMUSG00000025477	Transcript	ENSMUST00000106098	protein_coding	-
7:142577095-142577095	non_coding_transcript_exon_va	MODIFIER	Mir675	ENSMUSG00000076275	Transcript	ENSMUST00000102320	miRNA	-
7:142577095-142577095	upstream_gene_variant	MODIFIER	H19	ENSMUSG00000000031	Transcript	ENSMUST00000132294	lincRNA	-
7:142577095-142577095	non_coding_transcript_exon_va	MODIFIER	H19	ENSMUSG00000000031	Transcript	ENSMUST00000136359	lincRNA	-
7:142577095-142577095	upstream_gene_variant	MODIFIER	H19	ENSMUSG00000000031	Transcript	ENSMUST00000140716	lincRNA	-
7:142577095-142577095	intron_variant,non_coding_trans	MODIFIER	H19	ENSMUSG00000000031	Transcript	ENSMUST00000149974	lincRNA	-

7:142577095-142577095	non_coding_transcript_exon_var	MODIFIER	H19	ENSMUSG00000000031	Transcript	ENSMUST00000152754	lincRNA	-
7:142577095-142577095	upstream_gene_variant	MODIFIER	Gm27786	ENSMUSG00000098816	Transcript	ENSMUST00000184337	misc_RNA	-
7:142577095-142577095	downstream_gene_variant	MODIFIER	Gm27483	ENSMUSG00000099143	Transcript	ENSMUST00000184983	misc_RNA	-
7:142577095-142577095	non_coding_transcript_exon_var	MODIFIER	H19	ENSMUSG00000000031	Transcript	ENSMUST00000228259	lincRNA	-
7:142577095-142577095	upstream_gene_variant	MODIFIER	H19	ENSMUSG00000000031	Transcript	ENSMUST00000228514	lincRNA	-
7:142577095-142577095	regulatory_region_variant	MODIFIER	-	-	RegulatoryFeature	ENSMUSR00000726612	promoter	-
7:144921119-144921119	3_prime_UTR_variant	MODIFIER	Oraov1	ENSMUSG00000031072	Transcript	ENSMUST00000033388	protein_coding	rs32195639
7:144921119-144921119	downstream_gene_variant	MODIFIER	Oraov1	ENSMUSG00000031072	Transcript	ENSMUST00000093964	retained_intron	rs32195639
7:144921119-144921119	intron_variant	MODIFIER	Oraov1	ENSMUSG00000031072	Transcript	ENSMUST00000105895	protein_coding	rs32195639
7:144921119-144921119	intron_variant	MODIFIER	Oraov1	ENSMUSG00000031072	Transcript	ENSMUST00000128057	protein_coding	rs32195639
7:144921119-144921119	downstream_gene_variant	MODIFIER	Oraov1	ENSMUSG00000031072	Transcript	ENSMUST00000141737	protein_coding	rs32195639
7:144921119-144921119	downstream_gene_variant	MODIFIER	Oraov1	ENSMUSG00000031072	Transcript	ENSMUST00000147460	retained_intron	rs32195639
7:144921119-144921119	non_coding_transcript_exon_var	MODIFIER	Oraov1	ENSMUSG00000031072	Transcript	ENSMUST00000207094	retained_intron	rs32195639
7:144921119-144921119	intron_variant	MODIFIER	Oraov1	ENSMUSG00000031072	Transcript	ENSMUST00000207453	protein_coding	rs32195639
7:144921596-144921596	downstream_gene_variant	MODIFIER	Oraov1	ENSMUSG00000031072	Transcript	ENSMUST00000033388	protein_coding	rs31055079
7:144921596-144921596	downstream_gene_variant	MODIFIER	Oraov1	ENSMUSG00000031072	Transcript	ENSMUST00000093964	retained_intron	rs31055079
7:144921596-144921596	intron_variant	MODIFIER	Oraov1	ENSMUSG00000031072	Transcript	ENSMUST00000105895	protein_coding	rs31055079
7:144921596-144921596	intron_variant	MODIFIER	Oraov1	ENSMUSG00000031072	Transcript	ENSMUST00000128057	protein_coding	rs31055079
7:144921596-144921596	downstream_gene_variant	MODIFIER	Oraov1	ENSMUSG00000031072	Transcript	ENSMUST00000147460	retained_intron	rs31055079
7:144921596-144921596	non_coding_transcript_exon_var	MODIFIER	Oraov1	ENSMUSG00000031072	Transcript	ENSMUST00000207094	retained_intron	rs31055079
7:144921596-144921596	intron_variant	MODIFIER	Oraov1	ENSMUSG00000031072	Transcript	ENSMUST00000207453	protein_coding	rs31055079
7:145248398-145248398	intron_variant,NMD_transcript_v	MODIFIER	Tpcn2	ENSMUSG00000048677	Transcript	ENSMUST00000208328	nonsense_mediated_decay	rs238736247
7:145248398-145248398	intron_variant,NMD_transcript_v	MODIFIER	Tpcn2	ENSMUSG00000048677	Transcript	ENSMUST00000209047	nonsense_mediated_decay	rs238736247
7:145250874-145250874	downstream_gene_variant	MODIFIER	Tpcn2	ENSMUSG00000048677	Transcript	ENSMUST00000058022	protein_coding	rs48206549
7:145250874-145250874	downstream_gene_variant	MODIFIER	Tpcn2	ENSMUSG00000048677	Transcript	ENSMUST00000208148	nonsense_mediated_decay	rs48206549
7:145250874-145250874	intron_variant,NMD_transcript_v	MODIFIER	Tpcn2	ENSMUSG00000048677	Transcript	ENSMUST00000208328	nonsense_mediated_decay	rs48206549
7:145250874-145250874	downstream_gene_variant	MODIFIER	Tpcn2	ENSMUSG00000048677	Transcript	ENSMUST00000208841	nonsense_mediated_decay	rs48206549
7:145250874-145250874	downstream_gene_variant	MODIFIER	Tpcn2	ENSMUSG00000048677	Transcript	ENSMUST00000208867	retained_intron	rs48206549
7:145250874-145250874	intron_variant,NMD_transcript_v	MODIFIER	Tpcn2	ENSMUSG00000048677	Transcript	ENSMUST00000209047	nonsense_mediated_decay	rs48206549
7:145260582-145260582	intron_variant	MODIFIER	Tpcn2	ENSMUSG00000048677	Transcript	ENSMUST00000058022	protein_coding	rs31433618
7:145260582-145260582	intron_variant,NMD_transcript_v	MODIFIER	Tpcn2	ENSMUSG00000048677	Transcript	ENSMUST00000208148	nonsense_mediated_decay	rs31433618
7:145260582-145260582	downstream_gene_variant	MODIFIER	Tpcn2	ENSMUSG00000048677	Transcript	ENSMUST00000208281	retained_intron	rs31433618
7:145260582-145260582	intron_variant,NMD_transcript_v	MODIFIER	Tpcn2	ENSMUSG00000048677	Transcript	ENSMUST00000208328	nonsense_mediated_decay	rs31433618
7:145260582-145260582	intron_variant,NMD_transcript_v	MODIFIER	Tpcn2	ENSMUSG00000048677	Transcript	ENSMUST00000208841	nonsense_mediated_decay	rs31433618
7:145260582-145260582	upstream_gene_variant	MODIFIER	Tpcn2	ENSMUSG00000048677	Transcript	ENSMUST00000208867	retained_intron	rs31433618
7:145260582-145260582	intron_variant,NMD_transcript_v	MODIFIER	Tpcn2	ENSMUSG00000048677	Transcript	ENSMUST00000209047	nonsense_mediated_decay	rs31433618
7:45491353-45491353	upstream_gene_variant	MODIFIER	Dhdh	ENSMUSG00000011382	Transcript	ENSMUST00000011526	protein_coding	-
7:45491353-45491353	downstream_gene_variant	MODIFIER	Nucb1	ENSMUSG00000030824	Transcript	ENSMUST00000033096	protein_coding	-
7:45491353-45491353	intron_variant	MODIFIER	Tulp2	ENSMUSG00000023467	Transcript	ENSMUST00000107758	protein_coding	-
7:45491353-45491353	intron_variant	MODIFIER	Tulp2	ENSMUSG00000023467	Transcript	ENSMUST00000107759	protein_coding	-
7:45491353-45491353	downstream_gene_variant	MODIFIER	Nucb1	ENSMUSG00000030824	Transcript	ENSMUST00000210394	retained_intron	-
7:45491353-45491353	upstream_gene_variant	MODIFIER	Dhdh	ENSMUSG00000011382	Transcript	ENSMUST00000210440	retained_intron	-
7:45491353-45491353	downstream_gene_variant	MODIFIER	Nucb1	ENSMUSG00000030824	Transcript	ENSMUST00000210449	protein_coding	-
7:45491353-45491353	upstream_gene_variant	MODIFIER	Gm45808	ENSMUSG00000109926	Transcript	ENSMUST00000210701	protein_coding	-
7:45491353-45491353	intron_variant	MODIFIER	Tulp2	ENSMUSG00000023467	Transcript	ENSMUST00000210813	protein_coding	-
7:45491353-45491353	intron_variant,non_coding_trans	MODIFIER	Tulp2	ENSMUSG00000023467	Transcript	ENSMUST00000211413	processed_transcript	-
7:45491353-45491353	3_prime_UTR_variant	MODIFIER	Nucb1	ENSMUSG00000030824	Transcript	ENSMUST00000211765	protein_coding	-
7:53887319-53887319	downstream_gene_variant	MODIFIER	Mir6238	ENSMUSG00000099176	Transcript	ENSMUST00000184140	miRNA	-
7:66088512-66088512	intron_variant	MODIFIER	Selenos	ENSMUSG00000075701	Transcript	ENSMUST00000101801	protein_coding	rs51677780
7:66088512-66088512	intron_variant,NMD_transcript_v	MODIFIER	Selenos	ENSMUSG00000075701	Transcript	ENSMUST00000205279	nonsense_mediated_decay	rs51677780
7:66088512-66088512	downstream_gene_variant	MODIFIER	Selenos	ENSMUSG00000075701	Transcript	ENSMUST00000205965	retained_intron	rs51677780
7:66088512-66088512	intron_variant	MODIFIER	Selenos	ENSMUSG00000075701	Transcript	ENSMUST00000206044	protein_coding	rs51677780
7:66088512-66088512	intron_variant	MODIFIER	Selenos	ENSMUSG00000075701	Transcript	ENSMUST00000206575	protein_coding	rs51677780
7:66261453-66261453	intron_variant	MODIFIER	Lrrk1	ENSMUSG00000015133	Transcript	ENSMUST00000015277	protein_coding	rs33075151
7:66261453-66261453	downstream_gene_variant	MODIFIER	Lrrk1	ENSMUSG00000015133	Transcript	ENSMUST00000131239	retained_intron	rs33075151
7:66261453-66261453	intron_variant,non_coding_trans	MODIFIER	Lrrk1	ENSMUSG00000015133	Transcript	ENSMUST00000137181	retained_intron	rs33075151
7:66261453-66261453	intron_variant,NMD_transcript_v	MODIFIER	Lrrk1	ENSMUSG00000015133	Transcript	ENSMUST00000145954	nonsense_mediated_decay	rs33075151
7:66261453-66261453	intron_variant,non_coding_trans	MODIFIER	Lrrk1	ENSMUSG00000015133	Transcript	ENSMUST00000167705	processed_transcript	rs33075151
7:66261453-66261453	regulatory_region_variant	MODIFIER	-	-	RegulatoryFeature	ENSMUSR00000244361	enhancer	rs33075151
7:68739750-68739750	intron_variant	MODIFIER	Arrdc4	ENSMUSG00000042659	Transcript	ENSMUST00000048068	protein_coding	rs31139207
7:68739750-68739750	downstream_gene_variant	MODIFIER	Arrdc4	ENSMUSG00000042659	Transcript	ENSMUST00000118110	protein_coding	rs31139207
7:78792010-78792012	intron_variant	MODIFIER	Mrps11	ENSMUSG00000030611	Transcript	ENSMUST00000032840	protein_coding	rs237955066
7:78792010-78792012	downstream_gene_variant	MODIFIER	Mrps11	ENSMUSG00000030611	Transcript	ENSMUST00000123706	retained_intron	rs237955066
7:78792010-78792012	downstream_gene_variant	MODIFIER	Mrps11	ENSMUSG00000030611	Transcript	ENSMUST00000132922	retained_intron	rs237955066
7:78792010-78792012	downstream_gene_variant	MODIFIER	Mrps11	ENSMUSG00000030611	Transcript	ENSMUST00000133304	retained_intron	rs237955066
7:78792010-78792012	intron_variant,NMD_transcript_v	MODIFIER	Mrps11	ENSMUSG00000030611	Transcript	ENSMUST00000133553	nonsense_mediated_decay	rs237955066
7:78792010-78792012	downstream_gene_variant	MODIFIER	Mrps11	ENSMUSG00000030611	Transcript	ENSMUST00000136001	retained_intron	rs237955066
7:80241632-80241632	upstream_gene_variant	MODIFIER	Ttll13	ENSMUSG00000045467	Transcript	ENSMUST00000058266	protein_coding	rs50789410
7:80241632-80241632	3_prime_UTR_variant	MODIFIER	Gdpgp1	ENSMUSG00000050973	Transcript	ENSMUST00000062915	protein_coding	rs50789410

7:80241632-80241632	upstream_gene_variant	MODIFIER	Ttll13	ENSMUSG00000045467	Transcript	ENSMUST00000107381	retained_intron	rs50789410
7:80241632-80241632	upstream_gene_variant	MODIFIER	Gm15504	ENSMUSG00000082954	Transcript	ENSMUST00000119674	processed_pseudogene	rs50789410
7:80241632-80241632	upstream_gene_variant	MODIFIER	Ttll13	ENSMUSG00000045467	Transcript	ENSMUST00000206240	retained_intron	rs50789410
7:80241632-80241632	regulatory_region_variant	MODIFIER	-	-	RegulatoryFeature	ENSMUSR00000718478	enhancer	rs50789410
7:80288609-80288609	intron_variant	MODIFIER	Vps33b	ENSMUSG00000030534	Transcript	ENSMUST00000032749	protein_coding	rs31052201
7:80288609-80288609	downstream_gene_variant	MODIFIER	Vps33b	ENSMUSG00000030534	Transcript	ENSMUST00000128254	retained_intron	rs31052201
7:80288609-80288609	downstream_gene_variant	MODIFIER	Rccd1	ENSMUSG00000038930	Transcript	ENSMUST00000130888	retained_intron	rs31052201
7:80288609-80288609	intron_variant,NMD_transcript_v	MODIFIER	Vps33b	ENSMUSG00000030534	Transcript	ENSMUST00000135053	nonsense_mediated_decay	rs31052201
7:80288609-80288609	upstream_gene_variant	MODIFIER	Vps33b	ENSMUSG00000030534	Transcript	ENSMUST00000135470	retained_intron	rs31052201
7:80288609-80288609	downstream_gene_variant	MODIFIER	Vps33b	ENSMUSG00000030534	Transcript	ENSMUST00000145594	processed_transcript	rs31052201
7:80288609-80288609	intron_variant,NMD_transcript_v	MODIFIER	Vps33b	ENSMUSG00000030534	Transcript	ENSMUST00000150585	nonsense_mediated_decay	rs31052201
7:80288609-80288609	upstream_gene_variant	MODIFIER	Vps33b	ENSMUSG00000030534	Transcript	ENSMUST00000205864	protein_coding	rs31052201
7:80289898-80289898	intron_variant	MODIFIER	Vps33b	ENSMUSG00000030534	Transcript	ENSMUST00000032749	protein_coding	rs3697747
7:80289898-80289898	upstream_gene_variant	MODIFIER	Prc1	ENSMUSG00000038943	Transcript	ENSMUST00000047558	protein_coding	rs3697747
7:80289898-80289898	downstream_gene_variant	MODIFIER	Vps33b	ENSMUSG00000030534	Transcript	ENSMUST00000128254	retained_intron	rs3697747
7:80289898-80289898	downstream_gene_variant	MODIFIER	Rccd1	ENSMUSG00000038930	Transcript	ENSMUST00000130888	retained_intron	rs3697747
7:80289898-80289898	intron_variant,NMD_transcript_v	MODIFIER	Vps33b	ENSMUSG00000030534	Transcript	ENSMUST00000135053	nonsense_mediated_decay	rs3697747
7:80289898-80289898	upstream_gene_variant	MODIFIER	Vps33b	ENSMUSG00000030534	Transcript	ENSMUST00000135470	retained_intron	rs3697747
7:80289898-80289898	intron_variant,NMD_transcript_v	MODIFIER	Vps33b	ENSMUSG00000030534	Transcript	ENSMUST00000150585	nonsense_mediated_decay	rs3697747
7:80289898-80289898	upstream_gene_variant	MODIFIER	Prc1	ENSMUSG00000038943	Transcript	ENSMUST00000163812	protein_coding	rs3697747
7:80289898-80289898	upstream_gene_variant	MODIFIER	Prc1	ENSMUSG00000038943	Transcript	ENSMUST00000172911	retained_intron	rs3697747
7:80289898-80289898	upstream_gene_variant	MODIFIER	Prc1	ENSMUSG00000038943	Transcript	ENSMUST00000173824	protein_coding	rs3697747
7:80289898-80289898	upstream_gene_variant	MODIFIER	Prc1	ENSMUSG00000038943	Transcript	ENSMUST00000173914	retained_intron	rs3697747
7:80289898-80289898	upstream_gene_variant	MODIFIER	Prc1	ENSMUSG00000038943	Transcript	ENSMUST00000174172	protein_coding	rs3697747
7:80289898-80289898	upstream_gene_variant	MODIFIER	Prc1	ENSMUSG00000038943	Transcript	ENSMUST00000174199	protein_coding	rs3697747
7:80289898-80289898	upstream_gene_variant	MODIFIER	Vps33b	ENSMUSG00000030534	Transcript	ENSMUST00000205864	protein_coding	rs3697747
7:80289898-80289898	upstream_gene_variant	MODIFIER	Prc1	ENSMUSG00000038943	Transcript	ENSMUST00000206178	retained_intron	rs3697747
7:80301724-80301724	intron_variant	MODIFIER	Prc1	ENSMUSG00000038943	Transcript	ENSMUST00000047558	protein_coding	rs48150123
7:80301724-80301724	intron_variant,non_coding_trans	MODIFIER	Rccd1	ENSMUSG00000038930	Transcript	ENSMUST00000130888	retained_intron	rs48150123
7:80301724-80301724	intron_variant	MODIFIER	Prc1	ENSMUSG00000038943	Transcript	ENSMUST00000163812	protein_coding	rs48150123
7:80301724-80301724	intron_variant,non_coding_trans	MODIFIER	Prc1	ENSMUSG00000038943	Transcript	ENSMUST00000172911	retained_intron	rs48150123
7:80301724-80301724	upstream_gene_variant	MODIFIER	Prc1	ENSMUSG00000038943	Transcript	ENSMUST00000173170	protein_coding	rs48150123
7:80301724-80301724	intron_variant	MODIFIER	Prc1	ENSMUSG00000038943	Transcript	ENSMUST00000173824	protein_coding	rs48150123
7:80301724-80301724	non_coding_transcript_exon_va	MODIFIER	Prc1	ENSMUSG00000038943	Transcript	ENSMUST00000173914	retained_intron	rs48150123
7:80301724-80301724	intron_variant	MODIFIER	Prc1	ENSMUSG00000038943	Transcript	ENSMUST00000174172	protein_coding	rs48150123
7:80301724-80301724	intron_variant	MODIFIER	Prc1	ENSMUSG00000038943	Transcript	ENSMUST00000174199	protein_coding	rs48150123
7:80301724-80301724	upstream_gene_variant	MODIFIER	Prc1	ENSMUSG00000038943	Transcript	ENSMUST00000174254	retained_intron	rs48150123
7:80301724-80301724	upstream_gene_variant	MODIFIER	6330403N20Rik	ENSMUSG00000097912	Transcript	ENSMUST00000180467	lincRNA	rs48150123
7:80301724-80301724	intron_variant,non_coding_trans	MODIFIER	Prc1	ENSMUSG00000038943	Transcript	ENSMUST00000206178	retained_intron	rs48150123
7:80301724-80301724	regulatory_region_variant	MODIFIER	-	-	RegulatoryFeature	ENSMUSR00000718489	open_chromatin_region	rs48150123
7:80301724-80301724	regulatory_region_variant	MODIFIER	-	-	RegulatoryFeature	ENSMUSR00000718490	CTCF_binding_site	rs48150123
7:80327136-80327136	intron_variant	MODIFIER	Unc45a	ENSMUSG00000030533	Transcript	ENSMUST00000032748	protein_coding	rs51330426
7:80327136-80327136	upstream_gene_variant	MODIFIER	Rccd1	ENSMUSG00000038930	Transcript	ENSMUST00000047362	protein_coding	rs51330426
7:80327136-80327136	intron_variant	MODIFIER	Unc45a	ENSMUSG00000030533	Transcript	ENSMUST00000107368	protein_coding	rs51330426
7:80327136-80327136	upstream_gene_variant	MODIFIER	Rccd1	ENSMUSG00000038930	Transcript	ENSMUST00000121882	protein_coding	rs51330426
7:80327136-80327136	upstream_gene_variant	MODIFIER	Rccd1	ENSMUSG00000038930	Transcript	ENSMUST00000123109	retained_intron	rs51330426
7:80327136-80327136	upstream_gene_variant	MODIFIER	Rccd1	ENSMUSG00000038930	Transcript	ENSMUST00000123189	protein_coding	rs51330426
7:80327136-80327136	upstream_gene_variant	MODIFIER	Rccd1	ENSMUSG00000038930	Transcript	ENSMUST00000134288	retained_intron	rs51330426
7:80327136-80327136	upstream_gene_variant	MODIFIER	Rccd1	ENSMUSG00000038930	Transcript	ENSMUST00000148001	retained_intron	rs51330426
7:80327136-80327136	intron_variant,NMD_transcript_v	MODIFIER	Unc45a	ENSMUSG00000030533	Transcript	ENSMUST00000154428	nonsense_mediated_decay	rs51330426
7:80327136-80327136	upstream_gene_variant	MODIFIER	Rccd1	ENSMUSG00000038930	Transcript	ENSMUST00000155124	processed_transcript	rs51330426
7:80327136-80327136	upstream_gene_variant	MODIFIER	Rccd1	ENSMUSG00000038930	Transcript	ENSMUST00000205863	retained_intron	rs51330426
7:80327136-80327136	intron_variant	MODIFIER	Unc45a	ENSMUSG00000030533	Transcript	ENSMUST00000206363	protein_coding	rs51330426
7:80360231-80360231	intron_variant	MODIFIER	Man2a2	ENSMUSG00000038886	Transcript	ENSMUST00000098346	protein_coding	rs31674024
7:80360231-80360231	intron_variant,non_coding_trans	MODIFIER	Man2a2	ENSMUSG00000038886	Transcript	ENSMUST00000205318	retained_intron	rs31674024
7:80360231-80360231	downstream_gene_variant	MODIFIER	Man2a2	ENSMUSG00000038886	Transcript	ENSMUST00000205535	retained_intron	rs31674024
7:80360231-80360231	intron_variant,NMD_transcript_v	MODIFIER	Man2a2	ENSMUSG00000038886	Transcript	ENSMUST00000205853	nonsense_mediated_decay	rs31674024
7:80360231-80360231	intron_variant,non_coding_trans	MODIFIER	Man2a2	ENSMUSG00000038886	Transcript	ENSMUST00000206066	retained_intron	rs31674024
7:80360231-80360231	downstream_gene_variant	MODIFIER	Man2a2	ENSMUSG00000038886	Transcript	ENSMUST00000206301	nonsense_mediated_decay	rs31674024
7:80360231-80360231	upstream_gene_variant	MODIFIER	Man2a2	ENSMUSG00000038886	Transcript	ENSMUST00000206807	nonsense_mediated_decay	rs31674024
7:80360231-80360231	downstream_gene_variant	MODIFIER	Man2a2	ENSMUSG00000038886	Transcript	ENSMUST00000206917	retained_intron	rs31674024
7:80360231-80360231	upstream_gene_variant	MODIFIER	Man2a2	ENSMUSG00000038886	Transcript	ENSMUST00000206973	retained_intron	rs31674024
7:80360723-80360723	intron_variant	MODIFIER	Man2a2	ENSMUSG00000038886	Transcript	ENSMUST00000098346	protein_coding	rs49282041
7:80360723-80360723	intron_variant,non_coding_trans	MODIFIER	Man2a2	ENSMUSG00000038886	Transcript	ENSMUST00000205318	retained_intron	rs49282041
7:80360723-80360723	non_coding_transcript_exon_va	MODIFIER	Man2a2	ENSMUSG00000038886	Transcript	ENSMUST00000205535	retained_intron	rs49282041
7:80360723-80360723	intron_variant,NMD_transcript_v	MODIFIER	Man2a2	ENSMUSG00000038886	Transcript	ENSMUST00000205853	nonsense_mediated_decay	rs49282041
7:80360723-80360723	intron_variant,non_coding_trans	MODIFIER	Man2a2	ENSMUSG00000038886	Transcript	ENSMUST00000206066	retained_intron	rs49282041
7:80360723-80360723	3_prime_UTR_variant,NMD_trar	MODIFIER	Man2a2	ENSMUSG00000038886	Transcript	ENSMUST00000206301	nonsense_mediated_decay	rs49282041
7:80360723-80360723	upstream_gene_variant	MODIFIER	Man2a2	ENSMUSG00000038886	Transcript	ENSMUST00000206807	nonsense_mediated_decay	rs49282041

7:80360723-80360723	downstream_gene_variant	MODIFIER	Man2a2	ENSMUSG00000038886	Transcript	ENSMUST00000206917	retained_intron	rs49282041
7:80360723-80360723	upstream_gene_variant	MODIFIER	Man2a2	ENSMUSG00000038886	Transcript	ENSMUST00000206973	retained_intron	rs49282041
7:80401951-80401951	intron_variant	MODIFIER	Furin	ENSMUSG00000030530	Transcript	ENSMUST00000107362	protein_coding	rs217652626
7:80401951-80401951	upstream_gene_variant	MODIFIER	Furin	ENSMUSG00000030530	Transcript	ENSMUST00000120753	protein_coding	rs217652626
7:80401951-80401951	intron_variant	MODIFIER	Furin	ENSMUSG00000030530	Transcript	ENSMUST00000122232	protein_coding	rs217652626
7:80401951-80401951	intron_variant	MODIFIER	Furin	ENSMUSG00000030530	Transcript	ENSMUST00000135306	protein_coding	rs217652626
7:80401951-80401951	intron_variant	MODIFIER	Furin	ENSMUSG00000030530	Transcript	ENSMUST00000147150	protein_coding	rs217652626
7:80401951-80401951	upstream_gene_variant	MODIFIER	Furin	ENSMUSG00000030530	Transcript	ENSMUST00000153446	retained_intron	rs217652626
7:80401951-80401951	upstream_gene_variant	MODIFIER	Gm44851	ENSMUSG00000108436	Transcript	ENSMUST00000205735	antisense	rs217652626
7:80401951-80401951	intron_variant,NMD_transcript_v	MODIFIER	Furin	ENSMUSG00000030530	Transcript	ENSMUST00000206352	nonsense_mediated_decay	rs217652626
7:80401951-80401951	regulatory_region_variant	MODIFIER	-	-	RegulatoryFeature	ENSMUSR00000246075	promoter	rs217652626
7:81038812-81038812	intron_variant	MODIFIER	Zfp592	ENSMUSG00000005621	Transcript	ENSMUST00000107353	protein_coding	rs46924364
7:81038812-81038812	intron_variant,non_coding_trans	MODIFIER	Zfp592	ENSMUSG00000005621	Transcript	ENSMUST00000125137	retained_intron	rs46924364
7:81038812-81038812	downstream_gene_variant	MODIFIER	Zfp592	ENSMUSG00000005621	Transcript	ENSMUST00000149508	retained_intron	rs46924364
7:81038900-81038900	intron_variant	MODIFIER	Zfp592	ENSMUSG00000005621	Transcript	ENSMUST00000107353	protein_coding	rs47507695
7:81038900-81038900	intron_variant,non_coding_trans	MODIFIER	Zfp592	ENSMUSG00000005621	Transcript	ENSMUST00000125137	retained_intron	rs47507695
7:81038900-81038900	downstream_gene_variant	MODIFIER	Zfp592	ENSMUSG00000005621	Transcript	ENSMUST00000149508	retained_intron	rs47507695
7:81524392-81524392	intron_variant,non_coding_trans	MODIFIER	2900076A07Rik	ENSMUSG00000097277	Transcript	ENSMUST00000180385	lincRNA	rs46097822
7:81524392-81524392	upstream_gene_variant	MODIFIER	2900076A07Rik	ENSMUSG00000097277	Transcript	ENSMUST00000180705	lincRNA	rs46097822
7:81524392-81524392	intron_variant,non_coding_trans	MODIFIER	2900076A07Rik	ENSMUSG00000097277	Transcript	ENSMUST00000180879	lincRNA	rs46097822
7:81524392-81524392	upstream_gene_variant	MODIFIER	2900076A07Rik	ENSMUSG00000097277	Transcript	ENSMUST00000180983	lincRNA	rs46097822
7:81524392-81524392	intron_variant,non_coding_trans	MODIFIER	2900076A07Rik	ENSMUSG00000097277	Transcript	ENSMUST00000181164	lincRNA	rs46097822
7:81524392-81524392	intron_variant,non_coding_trans	MODIFIER	2900076A07Rik	ENSMUSG00000097277	Transcript	ENSMUST00000181264	lincRNA	rs46097822
7:81524392-81524392	upstream_gene_variant	MODIFIER	2900076A07Rik	ENSMUSG00000097277	Transcript	ENSMUST00000181492	lincRNA	rs46097822
7:81524392-81524392	intron_variant,non_coding_trans	MODIFIER	2900076A07Rik	ENSMUSG00000097277	Transcript	ENSMUST00000181903	lincRNA	rs46097822
7:81524392-81524392	regulatory_region_variant	MODIFIER	-	-	RegulatoryFeature	ENSMUSR00000246354	promoter	rs46097822
7:81524449-81524449	intron_variant,non_coding_trans	MODIFIER	2900076A07Rik	ENSMUSG00000097277	Transcript	ENSMUST00000180385	lincRNA	rs47445492
7:81524449-81524449	upstream_gene_variant	MODIFIER	2900076A07Rik	ENSMUSG00000097277	Transcript	ENSMUST00000180705	lincRNA	rs47445492
7:81524449-81524449	intron_variant,non_coding_trans	MODIFIER	2900076A07Rik	ENSMUSG00000097277	Transcript	ENSMUST00000180879	lincRNA	rs47445492
7:81524449-81524449	upstream_gene_variant	MODIFIER	2900076A07Rik	ENSMUSG00000097277	Transcript	ENSMUST00000180983	lincRNA	rs47445492
7:81524449-81524449	intron_variant,non_coding_trans	MODIFIER	2900076A07Rik	ENSMUSG00000097277	Transcript	ENSMUST00000181164	lincRNA	rs47445492
7:81524449-81524449	intron_variant,non_coding_trans	MODIFIER	2900076A07Rik	ENSMUSG00000097277	Transcript	ENSMUST00000181264	lincRNA	rs47445492
7:81524449-81524449	upstream_gene_variant	MODIFIER	2900076A07Rik	ENSMUSG00000097277	Transcript	ENSMUST00000181492	lincRNA	rs47445492
7:81524449-81524449	intron_variant,non_coding_trans	MODIFIER	2900076A07Rik	ENSMUSG00000097277	Transcript	ENSMUST00000181903	lincRNA	rs47445492
7:81524449-81524449	regulatory_region_variant	MODIFIER	-	-	RegulatoryFeature	ENSMUSR00000246354	promoter	rs47445492
7:81525518-81525518	upstream_gene_variant	MODIFIER	Mir1839	ENSMUSG00000093107	Transcript	ENSMUST00000175366	scaRNA	rs230247527
7:81525518-81525518	intron_variant,non_coding_trans	MODIFIER	2900076A07Rik	ENSMUSG00000097277	Transcript	ENSMUST00000180385	lincRNA	rs230247527
7:81525518-81525518	upstream_gene_variant	MODIFIER	2900076A07Rik	ENSMUSG00000097277	Transcript	ENSMUST00000180705	lincRNA	rs230247527
7:81525518-81525518	intron_variant,non_coding_trans	MODIFIER	2900076A07Rik	ENSMUSG00000097277	Transcript	ENSMUST00000180879	lincRNA	rs230247527
7:81525518-81525518	upstream_gene_variant	MODIFIER	2900076A07Rik	ENSMUSG00000097277	Transcript	ENSMUST00000180983	lincRNA	rs230247527
7:81525518-81525518	intron_variant,non_coding_trans	MODIFIER	2900076A07Rik	ENSMUSG00000097277	Transcript	ENSMUST00000181164	lincRNA	rs230247527
7:81525518-81525518	intron_variant,non_coding_trans	MODIFIER	2900076A07Rik	ENSMUSG00000097277	Transcript	ENSMUST00000181264	lincRNA	rs230247527
7:81525518-81525518	upstream_gene_variant	MODIFIER	2900076A07Rik	ENSMUSG00000097277	Transcript	ENSMUST00000181492	lincRNA	rs230247527
7:81525518-81525518	intron_variant,non_coding_trans	MODIFIER	2900076A07Rik	ENSMUSG00000097277	Transcript	ENSMUST00000181903	lincRNA	rs230247527
7:81525518-81525518	downstream_gene_variant	MODIFIER	Gm37829	ENSMUSG00000104453	Transcript	ENSMUST00000193864	TEC	rs230247527
7:81525518-81525518	upstream_gene_variant	MODIFIER	Mir1839	ENSMUSG00000104618	Transcript	ENSMUST00000196359	miRNA	rs230247527
7:81527992-81527992	upstream_gene_variant	MODIFIER	Mir1839	ENSMUSG00000093107	Transcript	ENSMUST00000175366	scaRNA	rs32267114
7:81527992-81527992	intron_variant,non_coding_trans	MODIFIER	2900076A07Rik	ENSMUSG00000097277	Transcript	ENSMUST00000180385	lincRNA	rs32267114
7:81527992-81527992	upstream_gene_variant	MODIFIER	2900076A07Rik	ENSMUSG00000097277	Transcript	ENSMUST00000180705	lincRNA	rs32267114
7:81527992-81527992	intron_variant,non_coding_trans	MODIFIER	2900076A07Rik	ENSMUSG00000097277	Transcript	ENSMUST00000180879	lincRNA	rs32267114
7:81527992-81527992	upstream_gene_variant	MODIFIER	2900076A07Rik	ENSMUSG00000097277	Transcript	ENSMUST00000180983	lincRNA	rs32267114
7:81527992-81527992	intron_variant,non_coding_trans	MODIFIER	2900076A07Rik	ENSMUSG00000097277	Transcript	ENSMUST00000181164	lincRNA	rs32267114
7:81527992-81527992	intron_variant,non_coding_trans	MODIFIER	2900076A07Rik	ENSMUSG00000097277	Transcript	ENSMUST00000181264	lincRNA	rs32267114
7:81527992-81527992	upstream_gene_variant	MODIFIER	2900076A07Rik	ENSMUSG00000097277	Transcript	ENSMUST00000181492	lincRNA	rs32267114
7:81527992-81527992	intron_variant,non_coding_trans	MODIFIER	2900076A07Rik	ENSMUSG00000097277	Transcript	ENSMUST00000181903	lincRNA	rs32267114
7:81527992-81527992	downstream_gene_variant	MODIFIER	Gm37829	ENSMUSG00000104453	Transcript	ENSMUST00000193864	TEC	rs32267114
7:81527992-81527992	upstream_gene_variant	MODIFIER	Mir1839	ENSMUSG00000104618	Transcript	ENSMUST00000196359	miRNA	rs32267114
7:81776376-81776376	intergenic_variant	MODIFIER	-	-	-	-	-	rs31485795
7:83892036-83892036	5_prime_UTR_variant	MODIFIER	Mesd	ENSMUSG00000038503	Transcript	ENSMUST00000094215	protein_coding	rs36537392
7:83892036-83892036	missense_variant	MODERATE	Mesd	ENSMUSG00000038503	Transcript	ENSMUST00000130103	protein_coding	rs36537392
7:83892036-83892036	intron_variant,non_coding_trans	MODIFIER	Mesd	ENSMUSG00000038503	Transcript	ENSMUST00000138803	processed_transcript	rs36537392
7:83892036-83892036	upstream_gene_variant	MODIFIER	Mesd	ENSMUSG00000038503	Transcript	ENSMUST00000153377	nonsense_mediated_decay	rs36537392
7:83892036-83892036	upstream_gene_variant	MODIFIER	Mesd	ENSMUSG00000038503	Transcript	ENSMUST00000207373	retained_intron	rs36537392
7:83892036-83892036	upstream_gene_variant	MODIFIER	Gm49493	ENSMUSG00000108878	Transcript	ENSMUST00000208403	TEC	rs36537392
7:83892036-83892036	regulatory_region_variant	MODIFIER	-	-	RegulatoryFeature	ENSMUSR00000246704	promoter	rs36537392
7:88305590-88305590	intron_variant	MODIFIER	Ctsc	ENSMUSG00000030560	Transcript	ENSMUST00000032779	protein_coding	rs32423389
7:88305590-88305590	intron_variant	MODIFIER	Ctsc	ENSMUSG00000030560	Transcript	ENSMUST00000128791	protein_coding	rs32423389
7:88305590-88305590	downstream_gene_variant	MODIFIER	Ctsc	ENSMUSG00000030560	Transcript	ENSMUST00000152834	retained_intron	rs32423389
7:88309033-88309033	intron_variant	MODIFIER	Ctsc	ENSMUSG00000030560	Transcript	ENSMUST00000032779	protein_coding	rs32000003

7:88309033-88309033	downstream_gene_variant	MODIFIER	Ctsc	ENSMUSG00000030560	Transcript	ENSMUST00000128791	protein_coding	rs32000003
7:88309033-88309033	upstream_gene_variant	MODIFIER	Gm44751	ENSMUSG00000109244	Transcript	ENSMUST00000208302	lincRNA	rs32000003
7:88311773-88311773	downstream_gene_variant	MODIFIER	Ctsc	ENSMUSG00000030560	Transcript	ENSMUST00000032779	protein_coding	-
7:88311773-88311773	downstream_gene_variant	MODIFIER	Ctsc	ENSMUSG00000030560	Transcript	ENSMUST00000128791	protein_coding	-
7:88311773-88311773	non_coding_transcript_exon_va	MODIFIER	Gm44751	ENSMUSG00000109244	Transcript	ENSMUST00000208302	lincRNA	-
7:88313804-88313804	downstream_gene_variant	MODIFIER	Ctsc	ENSMUSG00000030560	Transcript	ENSMUST00000032779	protein_coding	rs31526453
7:88313804-88313804	non_coding_transcript_exon_va	MODIFIER	Gm44751	ENSMUSG00000109244	Transcript	ENSMUST00000208302	lincRNA	rs31526453
7:88313930-88313930	downstream_gene_variant	MODIFIER	Ctsc	ENSMUSG00000030560	Transcript	ENSMUST00000032779	protein_coding	rs255045063
7:88313930-88313930	non_coding_transcript_exon_va	MODIFIER	Gm44751	ENSMUSG00000109244	Transcript	ENSMUST00000208302	lincRNA	rs255045063
7:88313936-88313936	downstream_gene_variant	MODIFIER	Ctsc	ENSMUSG00000030560	Transcript	ENSMUST00000032779	protein_coding	rs31643413
7:88313936-88313936	non_coding_transcript_exon_va	MODIFIER	Gm44751	ENSMUSG00000109244	Transcript	ENSMUST00000208302	lincRNA	rs31643413
7:89940119-89940119	intron_variant	MODIFIER	Hikeshi	ENSMUSG00000062797	Transcript	ENSMUST00000075010	protein_coding	rs31729940
7:89940119-89940119	intron_variant,NMD_transcript_v	MODIFIER	Hikeshi	ENSMUSG00000062797	Transcript	ENSMUST00000078918	nonsense_mediated_decay	rs31729940
7:89940119-89940119	intron_variant	MODIFIER	Hikeshi	ENSMUSG00000062797	Transcript	ENSMUST00000130609	protein_coding	rs31729940
7:89940119-89940119	intron_variant	MODIFIER	Hikeshi	ENSMUSG00000062797	Transcript	ENSMUST00000153470	protein_coding	rs31729940
7:89940119-89940119	intron_variant	MODIFIER	Hikeshi	ENSMUSG00000062797	Transcript	ENSMUST00000207309	protein_coding	rs31729940
7:89940119-89940119	non_coding_transcript_exon_va	MODIFIER	Hikeshi	ENSMUSG00000062797	Transcript	ENSMUST00000207695	retained_intron	rs31729940
7:89940119-89940119	intron_variant,non_coding_trans	MODIFIER	Hikeshi	ENSMUSG00000062797	Transcript	ENSMUST00000208357	processed_transcript	rs31729940
7:89940119-89940119	regulatory_region_variant	MODIFIER	-	-	RegulatoryFeature	ENSMUSR00000247145	promoter	rs31729940
7:89940121-89940121	intron_variant	MODIFIER	Hikeshi	ENSMUSG00000062797	Transcript	ENSMUST00000075010	protein_coding	rs31533631
7:89940121-89940121	intron_variant,NMD_transcript_v	MODIFIER	Hikeshi	ENSMUSG00000062797	Transcript	ENSMUST00000078918	nonsense_mediated_decay	rs31533631
7:89940121-89940121	intron_variant	MODIFIER	Hikeshi	ENSMUSG00000062797	Transcript	ENSMUST00000130609	protein_coding	rs31533631
7:89940121-89940121	intron_variant	MODIFIER	Hikeshi	ENSMUSG00000062797	Transcript	ENSMUST00000153470	protein_coding	rs31533631
7:89940121-89940121	intron_variant	MODIFIER	Hikeshi	ENSMUSG00000062797	Transcript	ENSMUST00000207309	protein_coding	rs31533631
7:89940121-89940121	non_coding_transcript_exon_va	MODIFIER	Hikeshi	ENSMUSG00000062797	Transcript	ENSMUST00000207695	retained_intron	rs31533631
7:89940121-89940121	intron_variant,non_coding_trans	MODIFIER	Hikeshi	ENSMUSG00000062797	Transcript	ENSMUST00000208357	processed_transcript	rs31533631
7:89940121-89940121	regulatory_region_variant	MODIFIER	-	-	RegulatoryFeature	ENSMUSR00000247145	promoter	rs31533631
7:90040668-90040668	downstream_gene_variant	MODIFIER	E230029C05Rik	ENSMUSG00000097585	Transcript	ENSMUST00000207458	antisense	rs32173563
7:90040668-90040668	intron_variant,non_coding_trans	MODIFIER	E230029C05Rik	ENSMUSG00000097585	Transcript	ENSMUST00000208266	antisense	rs32173563
7:90040668-90040668	upstream_gene_variant	MODIFIER	Gm44861	ENSMUSG00000108897	Transcript	ENSMUST00000208325	TEC	rs32173563
7:90040668-90040668	downstream_gene_variant	MODIFIER	E230029C05Rik	ENSMUSG00000097585	Transcript	ENSMUST00000208362	antisense	rs32173563
7:90040668-90040668	intron_variant,non_coding_trans	MODIFIER	E230029C05Rik	ENSMUSG00000097585	Transcript	ENSMUST00000208547	antisense	rs32173563
7:90047503-90047504	intron_variant,non_coding_trans	MODIFIER	E230029C05Rik	ENSMUSG00000097585	Transcript	ENSMUST00000208266	antisense	rs252744467
7:90047503-90047504	downstream_gene_variant	MODIFIER	Gm44861	ENSMUSG00000108897	Transcript	ENSMUST00000208325	TEC	rs252744467
7:90047503-90047504	intron_variant,non_coding_trans	MODIFIER	E230029C05Rik	ENSMUSG00000097585	Transcript	ENSMUST00000208547	antisense	rs252744467
7:90047505-90047505	intron_variant,non_coding_trans	MODIFIER	E230029C05Rik	ENSMUSG00000097585	Transcript	ENSMUST00000208266	antisense	rs6254275
7:90047505-90047505	downstream_gene_variant	MODIFIER	Gm44861	ENSMUSG00000108897	Transcript	ENSMUST00000208325	TEC	rs6254275
7:90047505-90047505	intron_variant,non_coding_trans	MODIFIER	E230029C05Rik	ENSMUSG00000097585	Transcript	ENSMUST00000208547	antisense	rs6254275
7:90131090-90131090	intron_variant	MODIFIER	Picalm	ENSMUSG00000039361	Transcript	ENSMUST00000049537	protein_coding	rs31657057
7:90131090-90131090	upstream_gene_variant	MODIFIER	2310010J17Rik	ENSMUSG00000097162	Transcript	ENSMUST00000181189	bidirectional_promoter_lncRNA	rs31657057
7:90131090-90131090	upstream_gene_variant	MODIFIER	Gm45223	ENSMUSG00000109429	Transcript	ENSMUST00000207037	TEC	rs31657057
7:90131090-90131090	intron_variant,non_coding_trans	MODIFIER	Picalm	ENSMUSG00000039361	Transcript	ENSMUST00000207197	retained_intron	rs31657057
7:90131090-90131090	intron_variant	MODIFIER	Picalm	ENSMUSG00000039361	Transcript	ENSMUST00000207225	protein_coding	rs31657057
7:90131090-90131090	intron_variant	MODIFIER	Picalm	ENSMUSG00000039361	Transcript	ENSMUST00000207484	protein_coding	rs31657057
7:90131090-90131090	non_coding_transcript_exon_va	MODIFIER	Picalm	ENSMUSG00000039361	Transcript	ENSMUST00000207596	retained_intron	rs31657057
7:90131090-90131090	intron_variant,non_coding_trans	MODIFIER	Picalm	ENSMUSG00000039361	Transcript	ENSMUST00000207949	processed_transcript	rs31657057
7:90131090-90131090	upstream_gene_variant	MODIFIER	Picalm	ENSMUSG00000039361	Transcript	ENSMUST00000208684	protein_coding	rs31657057
7:90131090-90131090	intron_variant	MODIFIER	Picalm	ENSMUSG00000039361	Transcript	ENSMUST00000208730	protein_coding	rs31657057
7:90131090-90131090	intron_variant	MODIFIER	Picalm	ENSMUSG00000039361	Transcript	ENSMUST00000208742	protein_coding	rs31657057
7:90131090-90131090	intron_variant	MODIFIER	Picalm	ENSMUSG00000039361	Transcript	ENSMUST00000209068	protein_coding	rs31657057
7:90131090-90131090	upstream_gene_variant	MODIFIER	2310010J17Rik	ENSMUSG00000097162	Transcript	ENSMUST00000209164	bidirectional_promoter_lncRNA	rs31657057
7:90131090-90131090	upstream_gene_variant	MODIFIER	2310010J17Rik	ENSMUSG00000097162	Transcript	ENSMUST00000209166	bidirectional_promoter_lncRNA	rs31657057
7:90131090-90131090	regulatory_region_variant	MODIFIER	-	-	RegulatoryFeature	ENSMUSR00000447023	promoter	rs31657057
7:90409592-90409592	non_coding_transcript_exon_va	MODIFIER	Sytl2	ENSMUSG00000030616	Transcript	ENSMUST00000098310	processed_transcript	rs31647158
7:90409592-90409592	3_prime_UTR_variant	MODIFIER	Sytl2	ENSMUSG00000030616	Transcript	ENSMUST00000107210	protein_coding	rs31647158
7:90409592-90409592	3_prime_UTR_variant	MODIFIER	Sytl2	ENSMUSG00000030616	Transcript	ENSMUST00000107211	protein_coding	rs31647158
7:90409592-90409592	non_coding_transcript_exon_va	MODIFIER	Sytl2	ENSMUSG00000030616	Transcript	ENSMUST00000189194	retained_intron	rs31647158
7:90409592-90409592	non_coding_transcript_exon_va	MODIFIER	Sytl2	ENSMUSG00000030616	Transcript	ENSMUST00000190365	processed_transcript	rs31647158
7:90409592-90409592	3_prime_UTR_variant	MODIFIER	Sytl2	ENSMUSG00000030616	Transcript	ENSMUST00000190731	protein_coding	rs31647158
7:90409592-90409592	3_prime_UTR_variant	MODIFIER	Sytl2	ENSMUSG00000030616	Transcript	ENSMUST00000190837	protein_coding	rs31647158
7:90409592-90409592	non_coding_transcript_exon_va	MODIFIER	Sytl2	ENSMUSG00000030616	Transcript	ENSMUST00000208486	retained_intron	rs31647158
7:90409592-90409592	non_coding_transcript_exon_va	MODIFIER	Sytl2	ENSMUSG00000030616	Transcript	ENSMUST00000208580	retained_intron	rs31647158
7:90409592-90409592	non_coding_transcript_exon_va	MODIFIER	Sytl2	ENSMUSG00000030616	Transcript	ENSMUST00000208809	retained_intron	rs31647158
7:90409592-90409592	non_coding_transcript_exon_va	MODIFIER	Sytl2	ENSMUSG00000030616	Transcript	ENSMUST00000209188	retained_intron	rs31647158
8:13043993-13043993	intron_variant	MODIFIER	F10	ENSMUSG00000031444	Transcript	ENSMUST00000033821	protein_coding	-
8:13043993-13043993	intron_variant	MODIFIER	F10	ENSMUSG00000031444	Transcript	ENSMUST00000063820	protein_coding	-
8:13043993-13043993	intron_variant	MODIFIER	F10	ENSMUSG00000031444	Transcript	ENSMUST00000123768	protein_coding	-
8:13043993-13043993	intron_variant	MODIFIER	F10	ENSMUSG00000031444	Transcript	ENSMUST00000128418	protein_coding	-
8:13043993-13043993	intron_variant	MODIFIER	F10	ENSMUSG00000031444	Transcript	ENSMUST00000152034	protein_coding	-

8:19703115-19703115	regulatory_region_variant	MODIFIER	-	-	RegulatoryFeature	ENSMUSR00000256130	promoter_flanking_region	rs265142024
8:19703115-19703115	regulatory_region_variant	MODIFIER	-	-	RegulatoryFeature	ENSMUSR00000452790	CTCF_binding_site	rs265142024
8:19703115-19703115	intergenic_variant	MODIFIER	-	-	-	-	-	rs265142024
8:19703206-19703206	regulatory_region_variant	MODIFIER	-	-	RegulatoryFeature	ENSMUSR00000256130	promoter_flanking_region	rs50511458
8:19703206-19703206	regulatory_region_variant	MODIFIER	-	-	RegulatoryFeature	ENSMUSR00000452790	CTCF_binding_site	rs50511458
8:19703206-19703206	intergenic_variant	MODIFIER	-	-	-	-	-	rs50511458
8:19703254-19703254	regulatory_region_variant	MODIFIER	-	-	RegulatoryFeature	ENSMUSR00000256130	promoter_flanking_region	rs1134654442
8:19703254-19703254	regulatory_region_variant	MODIFIER	-	-	RegulatoryFeature	ENSMUSR00000452790	CTCF_binding_site	rs1134654442
8:19703254-19703254	intergenic_variant	MODIFIER	-	-	-	-	-	rs1134654442
8:19703435-19703435	regulatory_region_variant	MODIFIER	-	-	RegulatoryFeature	ENSMUSR00000256130	promoter_flanking_region	rs1133593914
8:19703435-19703435	regulatory_region_variant	MODIFIER	-	-	RegulatoryFeature	ENSMUSR00000452790	CTCF_binding_site	rs1133593914
8:19703435-19703435	intergenic_variant	MODIFIER	-	-	-	-	-	rs1133593914
8:19703470-19703470	regulatory_region_variant	MODIFIER	-	-	RegulatoryFeature	ENSMUSR00000256130	promoter_flanking_region	rs1135368266
8:19703470-19703470	regulatory_region_variant	MODIFIER	-	-	RegulatoryFeature	ENSMUSR00000452790	CTCF_binding_site	rs1135368266
8:19703470-19703470	intergenic_variant	MODIFIER	-	-	-	-	-	rs1135368266
8:19706957-19706957	downstream_gene_variant	MODIFIER	Gm7760	ENSMUSG00000110531	Transcript	ENSMUST00000212265	processed_pseudogene	rs51782372
8:19706957-19706957	regulatory_region_variant	MODIFIER	-	-	RegulatoryFeature	ENSMUSR00000256132	promoter_flanking_region	rs51782372
8:19706957-19706957	regulatory_region_variant	MODIFIER	-	-	RegulatoryFeature	ENSMUSR00000452792	CTCF_binding_site	rs51782372
8:25814414-25814414	3_prime_UTR_variant	MODIFIER	Star	ENSMUSG00000031574	Transcript	ENSMUST00000033979	protein_coding	-
8:25814414-25814414	downstream_gene_variant	MODIFIER	Ash2l	ENSMUSG00000031575	Transcript	ENSMUST00000068892	protein_coding	-
8:25814414-25814414	downstream_gene_variant	MODIFIER	Ash2l	ENSMUSG00000031575	Transcript	ENSMUST00000110608	protein_coding	-
8:25814414-25814414	downstream_gene_variant	MODIFIER	Ash2l	ENSMUSG00000031575	Transcript	ENSMUST00000110609	protein_coding	-
8:25814414-25814414	downstream_gene_variant	MODIFIER	Ash2l	ENSMUSG00000031575	Transcript	ENSMUST00000110610	protein_coding	-
8:25814414-25814414	downstream_gene_variant	MODIFIER	Ash2l	ENSMUSG00000031575	Transcript	ENSMUST00000139946	nonsense_mediated_decay	-
8:25814414-25814414	downstream_gene_variant	MODIFIER	Ash2l	ENSMUSG00000031575	Transcript	ENSMUST00000151856	nonsense_mediated_decay	-
8:25814414-25814414	downstream_gene_variant	MODIFIER	Ash2l	ENSMUSG00000031575	Transcript	ENSMUST00000166078	protein_coding	-
8:25814414-25814414	downstream_gene_variant	MODIFIER	Star	ENSMUSG00000031574	Transcript	ENSMUST00000210565	protein_coding	-
8:25814415-25814415	3_prime_UTR_variant	MODIFIER	Star	ENSMUSG00000031574	Transcript	ENSMUST00000033979	protein_coding	-
8:25814415-25814415	downstream_gene_variant	MODIFIER	Ash2l	ENSMUSG00000031575	Transcript	ENSMUST00000068892	protein_coding	-
8:25814415-25814415	downstream_gene_variant	MODIFIER	Ash2l	ENSMUSG00000031575	Transcript	ENSMUST00000110608	protein_coding	-
8:25814415-25814415	downstream_gene_variant	MODIFIER	Ash2l	ENSMUSG00000031575	Transcript	ENSMUST00000110609	protein_coding	-
8:25814415-25814415	downstream_gene_variant	MODIFIER	Ash2l	ENSMUSG00000031575	Transcript	ENSMUST00000110610	protein_coding	-
8:25814415-25814415	downstream_gene_variant	MODIFIER	Ash2l	ENSMUSG00000031575	Transcript	ENSMUST00000139946	nonsense_mediated_decay	-
8:25814415-25814415	downstream_gene_variant	MODIFIER	Ash2l	ENSMUSG00000031575	Transcript	ENSMUST00000151856	nonsense_mediated_decay	-
8:25814415-25814415	downstream_gene_variant	MODIFIER	Ash2l	ENSMUSG00000031575	Transcript	ENSMUST00000166078	protein_coding	-
8:25814415-25814415	downstream_gene_variant	MODIFIER	Star	ENSMUSG00000031574	Transcript	ENSMUST00000210565	protein_coding	-
8:31736728-31736728	downstream_gene_variant	MODIFIER	Gm5117	ENSMUSG00000093862	Transcript	ENSMUST00000178878	processed_pseudogene	rs245441837
8:80993071-80993071	intron_variant	MODIFIER	Usp38	ENSMUSG00000038250	Transcript	ENSMUST00000042724	protein_coding	rs33574826
8:82765534-82765534	intron_variant	MODIFIER	Zfp330	ENSMUSG00000031711	Transcript	ENSMUST00000034147	protein_coding	rs37868158
8:82765534-82765534	intron_variant,non_coding_trans	MODIFIER	Zfp330	ENSMUSG00000031711	Transcript	ENSMUST00000209587	retained_intron	rs37868158
8:82765534-82765534	intron_variant,non_coding_trans	MODIFIER	Zfp330	ENSMUSG00000031711	Transcript	ENSMUST00000209601	processed_transcript	rs37868158
8:82765534-82765534	intron_variant,non_coding_trans	MODIFIER	Zfp330	ENSMUSG00000031711	Transcript	ENSMUST00000209727	retained_intron	rs37868158
8:82765534-82765534	non_coding_transcript_exon_var	MODIFIER	Zfp330	ENSMUSG00000031711	Transcript	ENSMUST00000211451	retained_intron	rs37868158
8:82765534-82765534	intron_variant,NMD_transcript_v	MODIFIER	Zfp330	ENSMUSG00000031711	Transcript	ENSMUST00000211462	nonsense_mediated_decay	rs37868158
8:82766065-82766065	splice_region_variant,intron_vari	LOW	Zfp330	ENSMUSG00000031711	Transcript	ENSMUST00000034147	protein_coding	rs37115436
8:82766065-82766065	splice_region_variant,intron_vari	LOW	Zfp330	ENSMUSG00000031711	Transcript	ENSMUST00000209587	retained_intron	rs37115436
8:82766065-82766065	splice_region_variant,intron_vari	LOW	Zfp330	ENSMUSG00000031711	Transcript	ENSMUST00000209601	processed_transcript	rs37115436
8:82766065-82766065	splice_region_variant,intron_vari	LOW	Zfp330	ENSMUSG00000031711	Transcript	ENSMUST00000209727	retained_intron	rs37115436
8:82766065-82766065	non_coding_transcript_exon_var	MODIFIER	Zfp330	ENSMUSG00000031711	Transcript	ENSMUST00000211451	retained_intron	rs37115436
8:82766065-82766065	splice_region_variant,intron_vari	LOW	Zfp330	ENSMUSG00000031711	Transcript	ENSMUST00000211462	nonsense_mediated_decay	rs37115436
8:82771699-82771699	intron_variant	MODIFIER	Zfp330	ENSMUSG00000031711	Transcript	ENSMUST00000034147	protein_coding	rs49793225
8:82771699-82771699	upstream_gene_variant	MODIFIER	Zfp330	ENSMUSG00000031711	Transcript	ENSMUST00000209587	retained_intron	rs49793225
8:82771699-82771699	upstream_gene_variant	MODIFIER	Zfp330	ENSMUSG00000031711	Transcript	ENSMUST00000209601	processed_transcript	rs49793225
8:82771699-82771699	upstream_gene_variant	MODIFIER	Zfp330	ENSMUSG00000031711	Transcript	ENSMUST00000209727	retained_intron	rs49793225
8:82771699-82771699	downstream_gene_variant	MODIFIER	Zfp330	ENSMUSG00000031711	Transcript	ENSMUST00000210958	retained_intron	rs49793225
8:82771699-82771699	upstream_gene_variant	MODIFIER	Zfp330	ENSMUSG00000031711	Transcript	ENSMUST00000211451	retained_intron	rs49793225
8:82771699-82771699	intron_variant,NMD_transcript_v	MODIFIER	Zfp330	ENSMUSG00000031711	Transcript	ENSMUST00000211462	nonsense_mediated_decay	rs49793225
8:83042682-83042682	synonymous_variant	LOW	Rnf150	ENSMUSG00000047747	Transcript	ENSMUST00000078525	protein_coding	rs50554552
8:83083939-83083939	3_prime_UTR_variant	MODIFIER	Rnf150	ENSMUSG00000047747	Transcript	ENSMUST00000078525	protein_coding	rs46981236
8:83083939-83083939	non_coding_transcript_exon_var	MODIFIER	Gm45449	ENSMUSG00000109876	Transcript	ENSMUST00000211485	antisense	rs46981236
8:83084158-83084158	3_prime_UTR_variant	MODIFIER	Rnf150	ENSMUSG00000047747	Transcript	ENSMUST00000078525	protein_coding	rs32635874
8:83084158-83084158	intron_variant,non_coding_trans	MODIFIER	Gm45449	ENSMUSG00000109876	Transcript	ENSMUST00000211485	antisense	rs32635874
8:83437455-83437455	intron_variant	MODIFIER	Scoc	ENSMUSG00000063253	Transcript	ENSMUST00000081506	protein_coding	rs49403180
8:83437455-83437455	intron_variant	MODIFIER	Scoc	ENSMUSG00000063253	Transcript	ENSMUST00000167525	protein_coding	rs49403180
8:83437455-83437455	intron_variant	MODIFIER	Scoc	ENSMUSG00000063253	Transcript	ENSMUST00000212031	protein_coding	rs49403180
8:83437455-83437455	intron_variant	MODIFIER	Scoc	ENSMUSG00000063253	Transcript	ENSMUST00000212449	protein_coding	rs49403180
8:83437455-83437455	intron_variant,non_coding_trans	MODIFIER	Scoc	ENSMUSG00000063253	Transcript	ENSMUST00000212473	processed_transcript	rs49403180
8:83437455-83437455	downstream_gene_variant	MODIFIER	Scoc	ENSMUSG00000063253	Transcript	ENSMUST00000212500	protein_coding	rs49403180
8:83437455-83437455	downstream_gene_variant	MODIFIER	Scoc	ENSMUSG00000063253	Transcript	ENSMUST00000212849	retained_intron	rs49403180

8:83437455-83437455	non_coding_transcript_exon_va	MODIFIER	Scoc	ENSMUSG00000063253	Transcript	ENSMUST00000212871	retained_intron	rs49403180
8:83437455-83437455	intron_variant	MODIFIER	Scoc	ENSMUSG00000063253	Transcript	ENSMUST00000212905	protein_coding	rs49403180
8:83437455-83437455	intron_variant,non_coding_trans	MODIFIER	Scoc	ENSMUSG00000063253	Transcript	ENSMUST00000212947	retained_intron	rs49403180
8:83674087-83674087	intron_variant	MODIFIER	Pkn1	ENSMUSG00000057672	Transcript	ENSMUST00000005616	protein_coding	rs32983962
8:83674087-83674087	downstream_gene_variant	MODIFIER	Ptger1	ENSMUSG00000019464	Transcript	ENSMUST00000019608	protein_coding	rs32983962
8:83674087-83674087	upstream_gene_variant	MODIFIER	Pkn1	ENSMUSG00000057672	Transcript	ENSMUST00000124946	retained_intron	rs32983962
8:83674087-83674087	non_coding_transcript_exon_va	MODIFIER	Pkn1	ENSMUSG00000057672	Transcript	ENSMUST00000128523	retained_intron	rs32983962
8:83674087-83674087	intron_variant,NMD_transcript_v	MODIFIER	Pkn1	ENSMUSG00000057672	Transcript	ENSMUST00000132945	nonsense_mediated_decay	rs32983962
8:83674087-83674087	downstream_gene_variant	MODIFIER	Pkn1	ENSMUSG00000057672	Transcript	ENSMUST00000133195	retained_intron	rs32983962
8:83674087-83674087	upstream_gene_variant	MODIFIER	Pkn1	ENSMUSG00000057672	Transcript	ENSMUST00000138898	processed_transcript	rs32983962
8:83674087-83674087	intron_variant	MODIFIER	Pkn1	ENSMUSG00000057672	Transcript	ENSMUST00000144258	protein_coding	rs32983962
8:83674087-83674087	intron_variant,non_coding_trans	MODIFIER	Pkn1	ENSMUSG00000057672	Transcript	ENSMUST00000146057	processed_transcript	rs32983962
8:83674087-83674087	downstream_gene_variant	MODIFIER	Ptger1	ENSMUSG00000019464	Transcript	ENSMUST00000159935	retained_intron	rs32983962
8:83674087-83674087	downstream_gene_variant	MODIFIER	Ptger1	ENSMUSG00000019464	Transcript	ENSMUST00000212519	processed_transcript	rs32983962
8:83690240-83690240	intron_variant	MODIFIER	Pkn1	ENSMUSG00000057672	Transcript	ENSMUST00000005616	protein_coding	rs33540472
8:83690240-83690240	intron_variant,NMD_transcript_v	MODIFIER	Pkn1	ENSMUSG00000057672	Transcript	ENSMUST00000132945	nonsense_mediated_decay	rs33540472
8:83690240-83690240	non_coding_transcript_exon_va	MODIFIER	Pkn1	ENSMUSG00000057672	Transcript	ENSMUST00000135356	retained_intron	rs33540472
8:83690240-83690240	intron_variant	MODIFIER	Pkn1	ENSMUSG00000057672	Transcript	ENSMUST00000144258	protein_coding	rs33540472
8:84092367-84092367	intron_variant	MODIFIER	Rfx1	ENSMUSG00000031706	Transcript	ENSMUST00000005600	protein_coding	rs50176008
8:84092367-84092367	downstream_gene_variant	MODIFIER	Dcaf15	ENSMUSG00000037103	Transcript	ENSMUST00000041367	protein_coding	rs50176008
8:84092367-84092367	downstream_gene_variant	MODIFIER	Dcaf15	ENSMUSG00000037103	Transcript	ENSMUST00000210279	protein_coding	rs50176008
8:84092367-84092367	downstream_gene_variant	MODIFIER	Rfx1	ENSMUSG00000031706	Transcript	ENSMUST00000210660	retained_intron	rs50176008
8:84092367-84092367	intron_variant	MODIFIER	Rfx1	ENSMUSG00000031706	Transcript	ENSMUST00000211046	protein_coding	rs50176008
8:84092473-84092473	intron_variant	MODIFIER	Rfx1	ENSMUSG00000031706	Transcript	ENSMUST00000005600	protein_coding	rs50320591
8:84092473-84092473	downstream_gene_variant	MODIFIER	Dcaf15	ENSMUSG00000037103	Transcript	ENSMUST00000041367	protein_coding	rs50320591
8:84092473-84092473	downstream_gene_variant	MODIFIER	Dcaf15	ENSMUSG00000037103	Transcript	ENSMUST00000210279	protein_coding	rs50320591
8:84092473-84092473	downstream_gene_variant	MODIFIER	Rfx1	ENSMUSG00000031706	Transcript	ENSMUST00000210660	retained_intron	rs50320591
8:84092473-84092473	intron_variant	MODIFIER	Rfx1	ENSMUSG00000031706	Transcript	ENSMUST00000211046	protein_coding	rs50320591
8:84097026-84097026	downstream_gene_variant	MODIFIER	Rfx1	ENSMUSG00000031706	Transcript	ENSMUST00000005600	protein_coding	rs48419810
8:84097026-84097026	downstream_gene_variant	MODIFIER	Dcaf15	ENSMUSG00000037103	Transcript	ENSMUST00000041367	protein_coding	rs48419810
8:84097026-84097026	downstream_gene_variant	MODIFIER	Dcaf15	ENSMUSG00000037103	Transcript	ENSMUST00000210279	protein_coding	rs48419810
8:84097026-84097026	downstream_gene_variant	MODIFIER	Rfx1	ENSMUSG00000031706	Transcript	ENSMUST00000211046	protein_coding	rs48419810
8:84130633-84130633	downstream_gene_variant	MODIFIER	Cc2d1a	ENSMUSG00000036686	Transcript	ENSMUST00000040383	protein_coding	rs33475878
8:84130633-84130633	synonymous_variant	LOW	Podnl1	ENSMUSG00000012889	Transcript	ENSMUST00000093380	protein_coding	rs33475878
8:84130633-84130633	downstream_gene_variant	MODIFIER	Cc2d1a	ENSMUSG00000036686	Transcript	ENSMUST00000117424	protein_coding	rs33475878
8:84130633-84130633	non_coding_transcript_exon_va	MODIFIER	Podnl1	ENSMUSG00000012889	Transcript	ENSMUST00000147175	retained_intron	rs33475878
8:84130633-84130633	downstream_gene_variant	MODIFIER	Cc2d1a	ENSMUSG00000036686	Transcript	ENSMUST00000154029	retained_intron	rs33475878
8:84130633-84130633	regulatory_region_variant	MODIFIER	-	-	RegulatoryFeature	ENSMUSR00000735601	promoter_flanking_region	rs33475878
8:84130645-84130645	downstream_gene_variant	MODIFIER	Cc2d1a	ENSMUSG00000036686	Transcript	ENSMUST00000040383	protein_coding	rs33045917
8:84130645-84130645	synonymous_variant	LOW	Podnl1	ENSMUSG00000012889	Transcript	ENSMUST00000093380	protein_coding	rs33045917
8:84130645-84130645	downstream_gene_variant	MODIFIER	Cc2d1a	ENSMUSG00000036686	Transcript	ENSMUST00000117424	protein_coding	rs33045917
8:84130645-84130645	non_coding_transcript_exon_va	MODIFIER	Podnl1	ENSMUSG00000012889	Transcript	ENSMUST00000147175	retained_intron	rs33045917
8:84130645-84130645	downstream_gene_variant	MODIFIER	Cc2d1a	ENSMUSG00000036686	Transcript	ENSMUST00000154029	retained_intron	rs33045917
8:84130645-84130645	regulatory_region_variant	MODIFIER	-	-	RegulatoryFeature	ENSMUSR00000735601	promoter_flanking_region	rs33045917
8:84132272-84132272	downstream_gene_variant	MODIFIER	Cc2d1a	ENSMUSG00000036686	Transcript	ENSMUST00000040383	protein_coding	rs51761684
8:84132272-84132272	3_prime_UTR_variant	MODIFIER	Podnl1	ENSMUSG00000012889	Transcript	ENSMUST00000093380	protein_coding	rs51761684
8:84132272-84132272	downstream_gene_variant	MODIFIER	Cc2d1a	ENSMUSG00000036686	Transcript	ENSMUST00000117424	protein_coding	rs51761684
8:84132272-84132272	non_coding_transcript_exon_va	MODIFIER	Podnl1	ENSMUSG00000012889	Transcript	ENSMUST00000147175	retained_intron	rs51761684
8:84132272-84132272	downstream_gene_variant	MODIFIER	Cc2d1a	ENSMUSG00000036686	Transcript	ENSMUST00000154029	retained_intron	rs51761684
8:84209682-84209682	downstream_gene_variant	MODIFIER	Zswim4	ENSMUSG00000035671	Transcript	ENSMUST00000039480	protein_coding	rs253385939
8:84209682-84209682	downstream_gene_variant	MODIFIER	Mir27a	ENSMUSG00000065444	Transcript	ENSMUST00000083510	miRNA	rs253385939
8:84209682-84209682	downstream_gene_variant	MODIFIER	Mir24-2	ENSMUSG00000065541	Transcript	ENSMUST00000083607	miRNA	rs253385939
8:84209682-84209682	downstream_gene_variant	MODIFIER	Mir23a	ENSMUSG00000065611	Transcript	ENSMUST00000083677	miRNA	rs253385939
8:84209682-84209682	downstream_gene_variant	MODIFIER	Gm26532	ENSMUSG00000097296	Transcript	ENSMUST00000180630	lincRNA	rs253385939
8:84209682-84209682	upstream_gene_variant	MODIFIER	Mir3074-2	ENSMUSG00000105458	Transcript	ENSMUST00000199621	miRNA	rs253385939
8:84246365-84246365	3_prime_UTR_variant	MODIFIER	D8Ertd738e	ENSMUSG00000019362	Transcript	ENSMUST00000019506	protein_coding	rs13459774
8:84246365-84246365	downstream_gene_variant	MODIFIER	Mri1	ENSMUSG00000004996	Transcript	ENSMUST00000125498	protein_coding	rs13459774
8:84246365-84246365	downstream_gene_variant	MODIFIER	Mri1	ENSMUSG00000004996	Transcript	ENSMUST00000126435	protein_coding	rs13459774
8:84252728-84252728	downstream_gene_variant	MODIFIER	Mri1	ENSMUSG00000004996	Transcript	ENSMUST00000005122	nonsense_mediated_decay	rs232814241
8:84252728-84252728	upstream_gene_variant	MODIFIER	D8Ertd738e	ENSMUSG00000019362	Transcript	ENSMUST00000019506	protein_coding	rs232814241
8:84252728-84252728	intron_variant	MODIFIER	Mri1	ENSMUSG00000004996	Transcript	ENSMUST00000125498	protein_coding	rs232814241
8:84252728-84252728	intron_variant	MODIFIER	Mri1	ENSMUSG00000004996	Transcript	ENSMUST00000126435	protein_coding	rs232814241
8:84252728-84252728	downstream_gene_variant	MODIFIER	Mri1	ENSMUSG00000004996	Transcript	ENSMUST00000127747	retained_intron	rs232814241
8:84252728-84252728	downstream_gene_variant	MODIFIER	Mri1	ENSMUSG00000004996	Transcript	ENSMUST00000140850	processed_transcript	rs232814241
8:84253561-84253561	downstream_gene_variant	MODIFIER	Ccdc130	ENSMUSG00000004994	Transcript	ENSMUST00000005120	protein_coding	rs47671269
8:84253561-84253561	downstream_gene_variant	MODIFIER	Mri1	ENSMUSG00000004996	Transcript	ENSMUST00000005122	nonsense_mediated_decay	rs47671269
8:84253561-84253561	upstream_gene_variant	MODIFIER	D8Ertd738e	ENSMUSG00000019362	Transcript	ENSMUST00000019506	protein_coding	rs47671269
8:84253561-84253561	downstream_gene_variant	MODIFIER	Ccdc130	ENSMUSG00000004994	Transcript	ENSMUST00000098578	protein_coding	rs47671269
8:84253561-84253561	intron_variant	MODIFIER	Mri1	ENSMUSG00000004996	Transcript	ENSMUST00000125498	protein_coding	rs47671269

8:84253561-84253561	intron_variant	MODIFIER	Mri1	ENSMUSG00000004996	Transcript	ENSMUST00000126435	protein_coding	rs47671269
8:84253561-84253561	downstream_gene_variant	MODIFIER	Mri1	ENSMUSG00000004996	Transcript	ENSMUST00000127747	retained_intron	rs47671269
8:84253561-84253561	downstream_gene_variant	MODIFIER	Mri1	ENSMUSG00000004996	Transcript	ENSMUST00000140850	processed_transcript	rs47671269
8:84253561-84253561	downstream_gene_variant	MODIFIER	Ccdc130	ENSMUSG00000004994	Transcript	ENSMUST00000164476	retained_intron	rs47671269
8:84670106-84670106	downstream_gene_variant	MODIFIER	Nacc1	ENSMUSG00000001910	Transcript	ENSMUST00000001975	protein_coding	rs32999775
8:84670106-84670106	downstream_gene_variant	MODIFIER	Gm26664	ENSMUSG00000097193	Transcript	ENSMUST00000181140	lincRNA	rs32999775
8:84670148-84670148	downstream_gene_variant	MODIFIER	Nacc1	ENSMUSG00000001910	Transcript	ENSMUST00000001975	protein_coding	rs50663194
8:84670148-84670148	downstream_gene_variant	MODIFIER	Gm26664	ENSMUSG00000097193	Transcript	ENSMUST00000181140	lincRNA	rs50663194
8:84670264-84670264	downstream_gene_variant	MODIFIER	Nacc1	ENSMUSG00000001910	Transcript	ENSMUST00000001975	protein_coding	rs47760041
8:84670264-84670264	downstream_gene_variant	MODIFIER	Gm26664	ENSMUSG00000097193	Transcript	ENSMUST00000181140	lincRNA	rs47760041
8:84671758-84671760	3_prime_UTR_variant	MODIFIER	Nacc1	ENSMUSG00000001910	Transcript	ENSMUST00000001975	protein_coding	rs248225127
8:84671758-84671760	downstream_gene_variant	MODIFIER	Gm26664	ENSMUSG00000097193	Transcript	ENSMUST00000181140	lincRNA	rs248225127
8:84679280-84679280	intron_variant	MODIFIER	Nacc1	ENSMUSG00000001910	Transcript	ENSMUST00000001975	protein_coding	rs33404735
8:84686016-84686016	upstream_gene_variant	MODIFIER	Trmt1	ENSMUSG00000001909	Transcript	ENSMUST00000001974	protein_coding	rs226109670
8:84686016-84686016	intron_variant	MODIFIER	Nacc1	ENSMUSG00000001910	Transcript	ENSMUST00000001975	protein_coding	rs226109670
8:84686016-84686016	upstream_gene_variant	MODIFIER	Trmt1	ENSMUSG00000001909	Transcript	ENSMUST00000077990	processed_transcript	rs226109670
8:84686016-84686016	upstream_gene_variant	MODIFIER	Trmt1	ENSMUSG00000001909	Transcript	ENSMUST00000109767	protein_coding	rs226109670
8:84686016-84686016	upstream_gene_variant	MODIFIER	Trmt1	ENSMUSG00000001909	Transcript	ENSMUST00000109768	protein_coding	rs226109670
8:84686016-84686016	upstream_gene_variant	MODIFIER	Trmt1	ENSMUSG00000001909	Transcript	ENSMUST00000125370	protein_coding	rs226109670
8:84686016-84686016	upstream_gene_variant	MODIFIER	Trmt1	ENSMUSG00000001909	Transcript	ENSMUST00000127915	retained_intron	rs226109670
8:84686016-84686016	upstream_gene_variant	MODIFIER	Trmt1	ENSMUSG00000001909	Transcript	ENSMUST00000128022	processed_transcript	rs226109670
8:84686016-84686016	upstream_gene_variant	MODIFIER	Trmt1	ENSMUSG00000001909	Transcript	ENSMUST00000128537	retained_intron	rs226109670
8:84686016-84686016	upstream_gene_variant	MODIFIER	Trmt1	ENSMUSG00000001909	Transcript	ENSMUST00000131700	nonsense_mediated_decay	rs226109670
8:84686016-84686016	upstream_gene_variant	MODIFIER	Trmt1	ENSMUSG00000001909	Transcript	ENSMUST00000137953	retained_intron	rs226109670
8:84686016-84686016	upstream_gene_variant	MODIFIER	Trmt1	ENSMUSG00000001909	Transcript	ENSMUST00000143427	nonsense_mediated_decay	rs226109670
8:84686016-84686016	upstream_gene_variant	MODIFIER	Trmt1	ENSMUSG00000001909	Transcript	ENSMUST00000150085	retained_intron	rs226109670
8:84686016-84686016	upstream_gene_variant	MODIFIER	Trmt1	ENSMUSG00000001909	Transcript	ENSMUST00000152301	protein_coding	rs226109670
8:84686016-84686016	upstream_gene_variant	MODIFIER	Trmt1	ENSMUSG00000001909	Transcript	ENSMUST00000175784	protein_coding	rs226109670
8:84686016-84686016	upstream_gene_variant	MODIFIER	Trmt1	ENSMUSG00000001909	Transcript	ENSMUST00000175870	processed_transcript	rs226109670
8:84686016-84686016	upstream_gene_variant	MODIFIER	Trmt1	ENSMUSG00000001909	Transcript	ENSMUST00000175980	retained_intron	rs226109670
8:84686016-84686016	upstream_gene_variant	MODIFIER	Trmt1	ENSMUSG00000001909	Transcript	ENSMUST00000176053	processed_transcript	rs226109670
8:84686016-84686016	upstream_gene_variant	MODIFIER	Trmt1	ENSMUSG00000001909	Transcript	ENSMUST00000176914	processed_transcript	rs226109670
8:84686016-84686016	upstream_gene_variant	MODIFIER	Trmt1	ENSMUSG00000001909	Transcript	ENSMUST00000177084	protein_coding	rs226109670
8:84686016-84686016	upstream_gene_variant	MODIFIER	Trmt1	ENSMUSG00000001909	Transcript	ENSMUST00000177286	processed_transcript	rs226109670
8:84686016-84686016	upstream_gene_variant	MODIFIER	Trmt1	ENSMUSG00000001909	Transcript	ENSMUST00000177364	retained_intron	rs226109670
8:84686016-84686016	upstream_gene_variant	MODIFIER	Trmt1	ENSMUSG00000001909	Transcript	ENSMUST00000177423	protein_coding	rs226109670
8:84686016-84686016	upstream_gene_variant	MODIFIER	Trmt1	ENSMUSG00000001909	Transcript	ENSMUST00000177531	protein_coding	rs226109670
8:84686016-84686016	regulatory_region_variant	MODIFIER	-	-	RegulatoryFeature	ENSMUSR00000262209	promoter	rs226109670
8:84690021-84690021	intron_variant	MODIFIER	Trmt1	ENSMUSG00000001909	Transcript	ENSMUST00000001974	protein_coding	rs47975902
8:84690021-84690021	upstream_gene_variant	MODIFIER	Nacc1	ENSMUSG00000001910	Transcript	ENSMUST00000001975	protein_coding	rs47975902
8:84690021-84690021	intron_variant,non_coding_trans	MODIFIER	Trmt1	ENSMUSG00000001909	Transcript	ENSMUST00000077990	processed_transcript	rs47975902
8:84690021-84690021	intron_variant	MODIFIER	Trmt1	ENSMUSG00000001909	Transcript	ENSMUST00000109767	protein_coding	rs47975902
8:84690021-84690021	intron_variant	MODIFIER	Trmt1	ENSMUSG00000001909	Transcript	ENSMUST00000109768	protein_coding	rs47975902
8:84690021-84690021	intron_variant	MODIFIER	Trmt1	ENSMUSG00000001909	Transcript	ENSMUST00000125370	protein_coding	rs47975902
8:84690021-84690021	intron_variant,non_coding_trans	MODIFIER	Trmt1	ENSMUSG00000001909	Transcript	ENSMUST00000127915	retained_intron	rs47975902
8:84690021-84690021	upstream_gene_variant	MODIFIER	Trmt1	ENSMUSG00000001909	Transcript	ENSMUST00000128022	processed_transcript	rs47975902
8:84690021-84690021	intron_variant,non_coding_trans	MODIFIER	Trmt1	ENSMUSG00000001909	Transcript	ENSMUST00000128537	retained_intron	rs47975902
8:84690021-84690021	intron_variant,NMD_transcript_v	MODIFIER	Trmt1	ENSMUSG00000001909	Transcript	ENSMUST00000131700	nonsense_mediated_decay	rs47975902
8:84690021-84690021	intron_variant,non_coding_trans	MODIFIER	Trmt1	ENSMUSG00000001909	Transcript	ENSMUST00000137953	retained_intron	rs47975902
8:84690021-84690021	intron_variant,NMD_transcript_v	MODIFIER	Trmt1	ENSMUSG00000001909	Transcript	ENSMUST00000143427	nonsense_mediated_decay	rs47975902
8:84690021-84690021	upstream_gene_variant	MODIFIER	Trmt1	ENSMUSG00000001909	Transcript	ENSMUST00000148118	retained_intron	rs47975902
8:84690021-84690021	intron_variant,non_coding_trans	MODIFIER	Trmt1	ENSMUSG00000001909	Transcript	ENSMUST00000150085	retained_intron	rs47975902
8:84690021-84690021	intron_variant	MODIFIER	Trmt1	ENSMUSG00000001909	Transcript	ENSMUST00000152301	protein_coding	rs47975902
8:84690021-84690021	intron_variant	MODIFIER	Trmt1	ENSMUSG00000001909	Transcript	ENSMUST00000175784	protein_coding	rs47975902
8:84690021-84690021	intron_variant,non_coding_trans	MODIFIER	Trmt1	ENSMUSG00000001909	Transcript	ENSMUST00000175870	processed_transcript	rs47975902
8:84690021-84690021	intron_variant,non_coding_trans	MODIFIER	Trmt1	ENSMUSG00000001909	Transcript	ENSMUST00000175980	retained_intron	rs47975902
8:84690021-84690021	intron_variant,non_coding_trans	MODIFIER	Trmt1	ENSMUSG00000001909	Transcript	ENSMUST00000176053	processed_transcript	rs47975902
8:84690021-84690021	downstream_gene_variant	MODIFIER	Trmt1	ENSMUSG00000001909	Transcript	ENSMUST00000176914	processed_transcript	rs47975902
8:84690021-84690021	intron_variant	MODIFIER	Trmt1	ENSMUSG00000001909	Transcript	ENSMUST00000177084	protein_coding	rs47975902
8:84690021-84690021	intron_variant,non_coding_trans	MODIFIER	Trmt1	ENSMUSG00000001909	Transcript	ENSMUST00000177286	processed_transcript	rs47975902
8:84690021-84690021	downstream_gene_variant	MODIFIER	Trmt1	ENSMUSG00000001909	Transcript	ENSMUST00000177364	retained_intron	rs47975902
8:84690021-84690021	intron_variant	MODIFIER	Trmt1	ENSMUSG00000001909	Transcript	ENSMUST00000177423	protein_coding	rs47975902
8:84690021-84690021	intron_variant	MODIFIER	Trmt1	ENSMUSG00000001909	Transcript	ENSMUST00000177531	protein_coding	rs47975902
8:84690021-84690021	regulatory_region_variant	MODIFIER	-	-	RegulatoryFeature	ENSMUSR00000262209	promoter	rs47975902
8:84698861-84698861	intron_variant	MODIFIER	Trmt1	ENSMUSG00000001909	Transcript	ENSMUST00000001974	protein_coding	rs32923228
8:84698861-84698861	upstream_gene_variant	MODIFIER	Lyl1	ENSMUSG00000034041	Transcript	ENSMUST00000037165	protein_coding	rs32923228
8:84698861-84698861	intron_variant	MODIFIER	Trmt1	ENSMUSG00000001909	Transcript	ENSMUST00000109767	protein_coding	rs32923228
8:84698861-84698861	intron_variant	MODIFIER	Trmt1	ENSMUSG00000001909	Transcript	ENSMUST00000109768	protein_coding	rs32923228
8:84698861-84698861	intron_variant	MODIFIER	Trmt1	ENSMUSG00000001909	Transcript	ENSMUST00000125370	protein_coding	rs32923228

8:84698861-84698861	downstream_gene_variant	MODIFIER	Trmt1	ENSMUSG00000001909	Transcript	ENSMUST00000128022	processed_transcript	rs32923228
8:84698861-84698861	downstream_gene_variant	MODIFIER	Trmt1	ENSMUSG00000001909	Transcript	ENSMUST00000131700	nonsense_mediated_decay	rs32923228
8:84698861-84698861	intron_variant	MODIFIER	Trmt1	ENSMUSG00000001909	Transcript	ENSMUST00000136423	protein_coding	rs32923228
8:84698861-84698861	intron_variant,non_coding_trans	MODIFIER	Trmt1	ENSMUSG00000001909	Transcript	ENSMUST00000137953	retained_intron	rs32923228
8:84698861-84698861	downstream_gene_variant	MODIFIER	Trmt1	ENSMUSG00000001909	Transcript	ENSMUST00000138300	retained_intron	rs32923228
8:84698861-84698861	downstream_gene_variant	MODIFIER	Trmt1	ENSMUSG00000001909	Transcript	ENSMUST00000143427	nonsense_mediated_decay	rs32923228
8:84698861-84698861	downstream_gene_variant	MODIFIER	Trmt1	ENSMUSG00000001909	Transcript	ENSMUST00000148118	retained_intron	rs32923228
8:84698861-84698861	downstream_gene_variant	MODIFIER	Nfix	ENSMUSG00000001911	Transcript	ENSMUST00000148644	processed_transcript	rs32923228
8:84698861-84698861	downstream_gene_variant	MODIFIER	Trmt1	ENSMUSG00000001909	Transcript	ENSMUST00000152301	protein_coding	rs32923228
8:84698861-84698861	downstream_gene_variant	MODIFIER	Trmt1	ENSMUSG00000001909	Transcript	ENSMUST00000175704	retained_intron	rs32923228
8:84698861-84698861	upstream_gene_variant	MODIFIER	Trmt1	ENSMUSG00000001909	Transcript	ENSMUST00000175767	retained_intron	rs32923228
8:84698861-84698861	downstream_gene_variant	MODIFIER	Trmt1	ENSMUSG00000001909	Transcript	ENSMUST00000175784	protein_coding	rs32923228
8:84698861-84698861	downstream_gene_variant	MODIFIER	Trmt1	ENSMUSG00000001909	Transcript	ENSMUST00000175884	nonsense_mediated_decay	rs32923228
8:84698861-84698861	downstream_gene_variant	MODIFIER	Trmt1	ENSMUSG00000001909	Transcript	ENSMUST00000177084	protein_coding	rs32923228
8:84698861-84698861	upstream_gene_variant	MODIFIER	Trmt1	ENSMUSG00000001909	Transcript	ENSMUST00000177260	retained_intron	rs32923228
8:84698861-84698861	downstream_gene_variant	MODIFIER	Trmt1	ENSMUSG00000001909	Transcript	ENSMUST00000177286	processed_transcript	rs32923228
8:84698861-84698861	downstream_gene_variant	MODIFIER	Trmt1	ENSMUSG00000001909	Transcript	ENSMUST00000177423	protein_coding	rs32923228
8:84724951-84724951	intron_variant	MODIFIER	Nfix	ENSMUSG00000001911	Transcript	ENSMUST00000076715	protein_coding	rs33450101
8:84724951-84724951	intron_variant	MODIFIER	G430095P16Rik	ENSMUSG00000074203	Transcript	ENSMUST00000098571	protein_coding	rs33450101
8:84724951-84724951	intron_variant	MODIFIER	Nfix	ENSMUSG00000001911	Transcript	ENSMUST00000099070	protein_coding	rs33450101
8:84724951-84724951	intron_variant	MODIFIER	Nfix	ENSMUSG00000001911	Transcript	ENSMUST00000109762	protein_coding	rs33450101
8:84724951-84724951	intron_variant	MODIFIER	Nfix	ENSMUSG00000001911	Transcript	ENSMUST00000109764	protein_coding	rs33450101
8:84724951-84724951	intron_variant	MODIFIER	Nfix	ENSMUSG00000001911	Transcript	ENSMUST00000126806	protein_coding	rs33450101
8:84724951-84724951	regulatory_region_variant	MODIFIER	-	-	RegulatoryFeature	ENSMUSR00000735712	promoter	rs33450101
8:84725920-84725920	intron_variant	MODIFIER	Nfix	ENSMUSG00000001911	Transcript	ENSMUST00000076715	protein_coding	rs32765689
8:84725920-84725920	intron_variant	MODIFIER	G430095P16Rik	ENSMUSG00000074203	Transcript	ENSMUST00000098571	protein_coding	rs32765689
8:84725920-84725920	intron_variant	MODIFIER	Nfix	ENSMUSG00000001911	Transcript	ENSMUST00000099070	protein_coding	rs32765689
8:84725920-84725920	intron_variant	MODIFIER	Nfix	ENSMUSG00000001911	Transcript	ENSMUST00000109762	protein_coding	rs32765689
8:84725920-84725920	intron_variant	MODIFIER	Nfix	ENSMUSG00000001911	Transcript	ENSMUST00000109764	protein_coding	rs32765689
8:84725920-84725920	intron_variant	MODIFIER	Nfix	ENSMUSG00000001911	Transcript	ENSMUST00000126806	protein_coding	rs32765689
8:84725920-84725920	regulatory_region_variant	MODIFIER	-	-	RegulatoryFeature	ENSMUSR00000735712	promoter	rs32765689
8:84725920-84725920	regulatory_region_variant	MODIFIER	-	-	RegulatoryFeature	ENSMUSR00000735715	CTCF_binding_site	rs32765689
8:84726317-84726317	intron_variant	MODIFIER	Nfix	ENSMUSG00000001911	Transcript	ENSMUST00000076715	protein_coding	rs32539411
8:84726317-84726317	intron_variant	MODIFIER	G430095P16Rik	ENSMUSG00000074203	Transcript	ENSMUST00000098571	protein_coding	rs32539411
8:84726317-84726317	intron_variant	MODIFIER	Nfix	ENSMUSG00000001911	Transcript	ENSMUST00000099070	protein_coding	rs32539411
8:84726317-84726317	intron_variant	MODIFIER	Nfix	ENSMUSG00000001911	Transcript	ENSMUST00000109762	protein_coding	rs32539411
8:84726317-84726317	intron_variant	MODIFIER	Nfix	ENSMUSG00000001911	Transcript	ENSMUST00000109764	protein_coding	rs32539411
8:84726317-84726317	intron_variant	MODIFIER	Nfix	ENSMUSG00000001911	Transcript	ENSMUST00000126806	protein_coding	rs32539411
8:84726317-84726317	regulatory_region_variant	MODIFIER	-	-	RegulatoryFeature	ENSMUSR00000735715	CTCF_binding_site	rs32539411
8:84861268-84861268	intron_variant	MODIFIER	Farsa	ENSMUSG00000003808	Transcript	ENSMUST00000003906	protein_coding	rs33315097
8:84861268-84861268	intron_variant	MODIFIER	Farsa	ENSMUSG00000003808	Transcript	ENSMUST00000109754	protein_coding	rs33315097
8:84861268-84861268	intron_variant,non_coding_trans	MODIFIER	Farsa	ENSMUSG00000003808	Transcript	ENSMUST00000129595	retained_intron	rs33315097
8:84861268-84861268	intron_variant,non_coding_trans	MODIFIER	Farsa	ENSMUSG00000003808	Transcript	ENSMUST00000144404	retained_intron	rs33315097
8:84861268-84861268	intron_variant,NMD_transcript_v	MODIFIER	Farsa	ENSMUSG00000003808	Transcript	ENSMUST00000156970	nonsense_mediated_decay	rs33315097
8:84864676-84864676	intron_variant	MODIFIER	Farsa	ENSMUSG00000003808	Transcript	ENSMUST00000003906	protein_coding	rs33236304
8:84864676-84864676	intron_variant	MODIFIER	Farsa	ENSMUSG00000003808	Transcript	ENSMUST00000109754	protein_coding	rs33236304
8:84864676-84864676	intron_variant,non_coding_trans	MODIFIER	Farsa	ENSMUSG00000003808	Transcript	ENSMUST00000129595	retained_intron	rs33236304
8:84864676-84864676	upstream_gene_variant	MODIFIER	Farsa	ENSMUSG00000003808	Transcript	ENSMUST00000141480	retained_intron	rs33236304
8:84864676-84864676	non_coding_transcript_exon_va	MODIFIER	Farsa	ENSMUSG00000003808	Transcript	ENSMUST00000144404	retained_intron	rs33236304
8:84864676-84864676	intron_variant,NMD_transcript_v	MODIFIER	Farsa	ENSMUSG00000003808	Transcript	ENSMUST00000156970	nonsense_mediated_decay	rs33236304
8:84864676-84864676	upstream_gene_variant	MODIFIER	Mir7069	ENSMUSG00000098331	Transcript	ENSMUST00000184765	miRNA	rs33236304
8:84887651-84887651	intron_variant	MODIFIER	Gcdh	ENSMUSG00000003809	Transcript	ENSMUST00000003907	protein_coding	rs51308166
8:84887651-84887651	downstream_gene_variant	MODIFIER	Syce2	ENSMUSG00000003824	Transcript	ENSMUST00000003922	nonsense_mediated_decay	rs51308166
8:84887651-84887651	intron_variant	MODIFIER	Gcdh	ENSMUSG00000003809	Transcript	ENSMUST00000109745	protein_coding	rs51308166
8:84887651-84887651	downstream_gene_variant	MODIFIER	Gcdh	ENSMUSG00000003809	Transcript	ENSMUST00000128023	retained_intron	rs51308166
8:84887651-84887651	3_prime_UTR_variant	MODIFIER	Syce2	ENSMUSG00000003824	Transcript	ENSMUST00000136026	protein_coding	rs51308166
8:84887651-84887651	downstream_gene_variant	MODIFIER	Gcdh	ENSMUSG00000003809	Transcript	ENSMUST00000136462	retained_intron	rs51308166
8:84887651-84887651	downstream_gene_variant	MODIFIER	Gcdh	ENSMUSG00000003809	Transcript	ENSMUST00000139180	retained_intron	rs51308166
8:84887651-84887651	downstream_gene_variant	MODIFIER	Syce2	ENSMUSG00000003824	Transcript	ENSMUST00000144466	retained_intron	rs51308166
8:84887651-84887651	downstream_gene_variant	MODIFIER	Syce2	ENSMUSG00000003824	Transcript	ENSMUST00000170296	protein_coding	rs51308166
8:84997169-84997169	synonymous_variant	LOW	Hook2	ENSMUSG00000052566	Transcript	ENSMUST00000064495	protein_coding	rs50198640
8:84997169-84997169	upstream_gene_variant	MODIFIER	Hook2	ENSMUSG00000052566	Transcript	ENSMUST00000209652	processed_transcript	rs50198640
8:84997169-84997169	synonymous_variant	LOW	Hook2	ENSMUSG00000052566	Transcript	ENSMUST00000209764	protein_coding	rs50198640
8:84997169-84997169	synonymous_variant	LOW	Hook2	ENSMUSG00000052566	Transcript	ENSMUST00000210326	protein_coding	rs50198640
8:85020883-85020883	intron_variant	MODIFIER	Asna1	ENSMUSG00000052456	Transcript	ENSMUST00000064314	protein_coding	rs46505680
8:85020883-85020883	intron_variant,NMD_transcript_v	MODIFIER	Asna1	ENSMUSG00000052456	Transcript	ENSMUST00000209834	nonsense_mediated_decay	rs46505680
8:85020883-85020883	downstream_gene_variant	MODIFIER	Asna1	ENSMUSG00000052456	Transcript	ENSMUST00000211221	retained_intron	rs46505680
8:85020883-85020883	intron_variant,non_coding_trans	MODIFIER	Asna1	ENSMUSG00000052456	Transcript	ENSMUST00000211702	processed_transcript	rs46505680
8:85024997-85024997	upstream_gene_variant	MODIFIER	Trir	ENSMUSG00000041203	Transcript	ENSMUST00000047281	protein_coding	rs48781676

8:85024997-85024997	intron_variant	MODIFIER	Asna1	ENSMUSG00000052456	Transcript	ENSMUST00000064314	protein_coding	rs48781676
8:85024997-85024997	intron_variant,NMD_transcript_v	MODIFIER	Asna1	ENSMUSG00000052456	Transcript	ENSMUST00000209834	nonsense_mediated_decay	rs48781676
8:85024997-85024997	non_coding_transcript_exon_var	MODIFIER	Asna1	ENSMUSG00000052456	Transcript	ENSMUST00000211221	retained_intron	rs48781676
8:85024997-85024997	upstream_gene_variant	MODIFIER	Asna1	ENSMUSG00000052456	Transcript	ENSMUST00000211702	processed_transcript	rs48781676
8:85024997-85024997	regulatory_region_variant	MODIFIER	-	-	RegulatoryFeature	ENSMUSR00000262305	promoter	rs48781676
8:85034159-85034159	downstream_gene_variant	MODIFIER	Trir	ENSMUSG00000041203	Transcript	ENSMUST00000047281	protein_coding	rs52493912
8:85034159-85034159	upstream_gene_variant	MODIFIER	Tnpo2	ENSMUSG00000031691	Transcript	ENSMUST00000093360	protein_coding	rs52493912
8:85034159-85034159	upstream_gene_variant	MODIFIER	Tnpo2	ENSMUSG00000031691	Transcript	ENSMUST00000166592	protein_coding	rs52493912
8:85034159-85034159	upstream_gene_variant	MODIFIER	Tnpo2	ENSMUSG00000031691	Transcript	ENSMUST00000210576	retained_intron	rs52493912
8:85034159-85034159	upstream_gene_variant	MODIFIER	Tnpo2	ENSMUSG00000031691	Transcript	ENSMUST00000210945	protein_coding	rs52493912
8:85034159-85034159	upstream_gene_variant	MODIFIER	Tnpo2	ENSMUSG00000031691	Transcript	ENSMUST00000211601	protein_coding	rs52493912
8:85066997-85066997	upstream_gene_variant	MODIFIER	Dhps	ENSMUSG00000060038	Transcript	ENSMUST00000078665	protein_coding	rs32900093
8:85066997-85066997	3_prime_UTR_variant	MODIFIER	Fbxw9	ENSMUSG00000008167	Transcript	ENSMUST00000095220	protein_coding	rs32900093
8:85066997-85066997	downstream_gene_variant	MODIFIER	Fbxw9	ENSMUSG00000008167	Transcript	ENSMUST00000125109	retained_intron	rs32900093
8:85066997-85066997	upstream_gene_variant	MODIFIER	Dhps	ENSMUSG00000060038	Transcript	ENSMUST00000129826	retained_intron	rs32900093
8:85066997-85066997	non_coding_transcript_exon_var	MODIFIER	Fbxw9	ENSMUSG00000008167	Transcript	ENSMUST00000139721	retained_intron	rs32900093
8:85066997-85066997	downstream_gene_variant	MODIFIER	Fbxw9	ENSMUSG00000008167	Transcript	ENSMUST00000141183	retained_intron	rs32900093
8:85066997-85066997	downstream_gene_variant	MODIFIER	Fbxw9	ENSMUSG00000008167	Transcript	ENSMUST00000142036	processed_transcript	rs32900093
8:85066997-85066997	downstream_gene_variant	MODIFIER	Fbxw9	ENSMUSG00000008167	Transcript	ENSMUST00000143763	processed_transcript	rs32900093
8:85066997-85066997	downstream_gene_variant	MODIFIER	Fbxw9	ENSMUSG00000008167	Transcript	ENSMUST00000145479	retained_intron	rs32900093
8:85066997-85066997	downstream_gene_variant	MODIFIER	Fbxw9	ENSMUSG00000008167	Transcript	ENSMUST00000151962	retained_intron	rs32900093
8:85066997-85066997	upstream_gene_variant	MODIFIER	Dhps	ENSMUSG00000060038	Transcript	ENSMUST00000152871	nonsense_mediated_decay	rs32900093
8:85066997-85066997	non_coding_transcript_exon_var	MODIFIER	Fbxw9	ENSMUSG00000008167	Transcript	ENSMUST00000152884	retained_intron	rs32900093
8:85066997-85066997	downstream_gene_variant	MODIFIER	Gm5741	ENSMUSG00000095845	Transcript	ENSMUST00000177563	protein_coding	rs32900093
8:85066997-85066997	downstream_gene_variant	MODIFIER	Mir7070	ENSMUSG00000099076	Transcript	ENSMUST00000183657	miRNA	rs32900093
8:85067076-85067077	upstream_gene_variant	MODIFIER	Dhps	ENSMUSG00000060038	Transcript	ENSMUST00000078665	protein_coding	rs258571054
8:85067076-85067077	3_prime_UTR_variant	MODIFIER	Fbxw9	ENSMUSG00000008167	Transcript	ENSMUST00000095220	protein_coding	rs258571054
8:85067076-85067077	downstream_gene_variant	MODIFIER	Fbxw9	ENSMUSG00000008167	Transcript	ENSMUST00000125109	retained_intron	rs258571054
8:85067076-85067077	upstream_gene_variant	MODIFIER	Dhps	ENSMUSG00000060038	Transcript	ENSMUST00000129826	retained_intron	rs258571054
8:85067076-85067077	non_coding_transcript_exon_var	MODIFIER	Fbxw9	ENSMUSG00000008167	Transcript	ENSMUST00000139721	retained_intron	rs258571054
8:85067076-85067077	downstream_gene_variant	MODIFIER	Fbxw9	ENSMUSG00000008167	Transcript	ENSMUST00000141183	retained_intron	rs258571054
8:85067076-85067077	downstream_gene_variant	MODIFIER	Fbxw9	ENSMUSG00000008167	Transcript	ENSMUST00000142036	processed_transcript	rs258571054
8:85067076-85067077	downstream_gene_variant	MODIFIER	Fbxw9	ENSMUSG00000008167	Transcript	ENSMUST00000143763	processed_transcript	rs258571054
8:85067076-85067077	downstream_gene_variant	MODIFIER	Fbxw9	ENSMUSG00000008167	Transcript	ENSMUST00000145479	retained_intron	rs258571054
8:85067076-85067077	downstream_gene_variant	MODIFIER	Fbxw9	ENSMUSG00000008167	Transcript	ENSMUST00000151962	retained_intron	rs258571054
8:85067076-85067077	upstream_gene_variant	MODIFIER	Dhps	ENSMUSG00000060038	Transcript	ENSMUST00000152871	nonsense_mediated_decay	rs258571054
8:85067076-85067077	non_coding_transcript_exon_var	MODIFIER	Fbxw9	ENSMUSG00000008167	Transcript	ENSMUST00000152884	retained_intron	rs258571054
8:85067076-85067077	downstream_gene_variant	MODIFIER	Gm5741	ENSMUSG00000095845	Transcript	ENSMUST00000177563	protein_coding	rs258571054
8:85067076-85067077	downstream_gene_variant	MODIFIER	Mir7070	ENSMUSG00000099076	Transcript	ENSMUST00000183657	miRNA	rs258571054
8:85067953-85067953	upstream_gene_variant	MODIFIER	Dhps	ENSMUSG00000060038	Transcript	ENSMUST00000078665	protein_coding	rs32578096
8:85067953-85067953	downstream_gene_variant	MODIFIER	Fbxw9	ENSMUSG00000008167	Transcript	ENSMUST00000095220	protein_coding	rs32578096
8:85067953-85067953	downstream_gene_variant	MODIFIER	Fbxw9	ENSMUSG00000008167	Transcript	ENSMUST00000125109	retained_intron	rs32578096
8:85067953-85067953	upstream_gene_variant	MODIFIER	Dhps	ENSMUSG00000060038	Transcript	ENSMUST00000129826	retained_intron	rs32578096
8:85067953-85067953	downstream_gene_variant	MODIFIER	Fbxw9	ENSMUSG00000008167	Transcript	ENSMUST00000139721	retained_intron	rs32578096
8:85067953-85067953	downstream_gene_variant	MODIFIER	Fbxw9	ENSMUSG00000008167	Transcript	ENSMUST00000141183	retained_intron	rs32578096
8:85067953-85067953	downstream_gene_variant	MODIFIER	Fbxw9	ENSMUSG00000008167	Transcript	ENSMUST00000142036	processed_transcript	rs32578096
8:85067953-85067953	upstream_gene_variant	MODIFIER	Dhps	ENSMUSG00000060038	Transcript	ENSMUST00000142210	retained_intron	rs32578096
8:85067953-85067953	downstream_gene_variant	MODIFIER	Fbxw9	ENSMUSG00000008167	Transcript	ENSMUST00000143763	processed_transcript	rs32578096
8:85067953-85067953	downstream_gene_variant	MODIFIER	Fbxw9	ENSMUSG00000008167	Transcript	ENSMUST00000145479	retained_intron	rs32578096
8:85067953-85067953	downstream_gene_variant	MODIFIER	Fbxw9	ENSMUSG00000008167	Transcript	ENSMUST00000151962	retained_intron	rs32578096
8:85067953-85067953	upstream_gene_variant	MODIFIER	Dhps	ENSMUSG00000060038	Transcript	ENSMUST00000152871	nonsense_mediated_decay	rs32578096
8:85067953-85067953	downstream_gene_variant	MODIFIER	Fbxw9	ENSMUSG00000008167	Transcript	ENSMUST00000152884	retained_intron	rs32578096
8:85067953-85067953	5_prime_UTR_variant	MODIFIER	Gm5741	ENSMUSG00000095845	Transcript	ENSMUST00000177563	protein_coding	rs32578096
8:85070601-85070607	upstream_gene_variant	MODIFIER	Dhps	ENSMUSG00000060038	Transcript	ENSMUST00000078665	protein_coding	rs257787839
8:85070601-85070607	downstream_gene_variant	MODIFIER	Wdr83	ENSMUSG00000005150	Transcript	ENSMUST00000093357	protein_coding	rs257787839
8:85070601-85070607	downstream_gene_variant	MODIFIER	Fbxw9	ENSMUSG00000008167	Transcript	ENSMUST00000095220	protein_coding	rs257787839
8:85070601-85070607	downstream_gene_variant	MODIFIER	Fbxw9	ENSMUSG00000008167	Transcript	ENSMUST00000125109	retained_intron	rs257787839
8:85070601-85070607	upstream_gene_variant	MODIFIER	Dhps	ENSMUSG00000060038	Transcript	ENSMUST00000129826	retained_intron	rs257787839
8:85070601-85070607	downstream_gene_variant	MODIFIER	Wdr83	ENSMUSG00000005150	Transcript	ENSMUST00000131909	retained_intron	rs257787839
8:85070601-85070607	upstream_gene_variant	MODIFIER	Dhps	ENSMUSG00000060038	Transcript	ENSMUST00000132356	retained_intron	rs257787839
8:85070601-85070607	upstream_gene_variant	MODIFIER	Dhps	ENSMUSG00000060038	Transcript	ENSMUST00000132766	retained_intron	rs257787839
8:85070601-85070607	downstream_gene_variant	MODIFIER	Wdr83	ENSMUSG00000005150	Transcript	ENSMUST00000136696	retained_intron	rs257787839
8:85070601-85070607	downstream_gene_variant	MODIFIER	Fbxw9	ENSMUSG00000008167	Transcript	ENSMUST00000139721	retained_intron	rs257787839
8:85070601-85070607	downstream_gene_variant	MODIFIER	Fbxw9	ENSMUSG00000008167	Transcript	ENSMUST00000141183	retained_intron	rs257787839
8:85070601-85070607	downstream_gene_variant	MODIFIER	Fbxw9	ENSMUSG00000008167	Transcript	ENSMUST00000142036	processed_transcript	rs257787839
8:85070601-85070607	downstream_gene_variant	MODIFIER	Wdr83	ENSMUSG00000005150	Transcript	ENSMUST00000142201	protein_coding	rs257787839
8:85070601-85070607	upstream_gene_variant	MODIFIER	Dhps	ENSMUSG00000060038	Transcript	ENSMUST00000142210	retained_intron	rs257787839
8:85070601-85070607	upstream_gene_variant	MODIFIER	Dhps	ENSMUSG00000060038	Transcript	ENSMUST00000142422	retained_intron	rs257787839
8:85070601-85070607	downstream_gene_variant	MODIFIER	Fbxw9	ENSMUSG00000008167	Transcript	ENSMUST00000143763	processed_transcript	rs257787839

8:85070601-85070607	downstream_gene_variant	MODIFIER	Wdr83	ENSMUSG00000005150	Transcript	ENSMUST00000145880	retained_intron	rs257787839
8:85070601-85070607	downstream_gene_variant	MODIFIER	Wdr83	ENSMUSG00000005150	Transcript	ENSMUST00000146900	retained_intron	rs257787839
8:85070601-85070607	downstream_gene_variant	MODIFIER	Fbxw9	ENSMUSG00000008167	Transcript	ENSMUST00000151962	retained_intron	rs257787839
8:85070601-85070607	downstream_gene_variant	MODIFIER	Wdr83	ENSMUSG00000005150	Transcript	ENSMUST00000152186	retained_intron	rs257787839
8:85070601-85070607	upstream_gene_variant	MODIFIER	Dhps	ENSMUSG00000060038	Transcript	ENSMUST00000152871	nonsense_mediated_decay	rs257787839
8:85070601-85070607	downstream_gene_variant	MODIFIER	Fbxw9	ENSMUSG00000008167	Transcript	ENSMUST00000152884	retained_intron	rs257787839
8:85070601-85070607	upstream_gene_variant	MODIFIER	Dhps	ENSMUSG00000060038	Transcript	ENSMUST00000154575	retained_intron	rs257787839
8:85070601-85070607	upstream_gene_variant	MODIFIER	Gm5741	ENSMUSG00000095845	Transcript	ENSMUST00000177563	protein_coding	rs257787839
8:85070850-85070850	upstream_gene_variant	MODIFIER	Dhps	ENSMUSG00000060038	Transcript	ENSMUST00000078665	protein_coding	rs47708877
8:85070850-85070850	downstream_gene_variant	MODIFIER	Wdr83	ENSMUSG00000005150	Transcript	ENSMUST00000093357	protein_coding	rs47708877
8:85070850-85070850	downstream_gene_variant	MODIFIER	Fbxw9	ENSMUSG00000008167	Transcript	ENSMUST00000095220	protein_coding	rs47708877
8:85070850-85070850	downstream_gene_variant	MODIFIER	Fbxw9	ENSMUSG00000008167	Transcript	ENSMUST00000125109	retained_intron	rs47708877
8:85070850-85070850	upstream_gene_variant	MODIFIER	Dhps	ENSMUSG00000060038	Transcript	ENSMUST00000129826	retained_intron	rs47708877
8:85070850-85070850	downstream_gene_variant	MODIFIER	Wdr83	ENSMUSG00000005150	Transcript	ENSMUST00000131909	retained_intron	rs47708877
8:85070850-85070850	upstream_gene_variant	MODIFIER	Dhps	ENSMUSG00000060038	Transcript	ENSMUST00000132356	retained_intron	rs47708877
8:85070850-85070850	upstream_gene_variant	MODIFIER	Dhps	ENSMUSG00000060038	Transcript	ENSMUST00000132766	retained_intron	rs47708877
8:85070850-85070850	downstream_gene_variant	MODIFIER	Wdr83	ENSMUSG00000005150	Transcript	ENSMUST00000136696	retained_intron	rs47708877
8:85070850-85070850	downstream_gene_variant	MODIFIER	Fbxw9	ENSMUSG00000008167	Transcript	ENSMUST00000139721	retained_intron	rs47708877
8:85070850-85070850	downstream_gene_variant	MODIFIER	Fbxw9	ENSMUSG00000008167	Transcript	ENSMUST00000141183	retained_intron	rs47708877
8:85070850-85070850	downstream_gene_variant	MODIFIER	Fbxw9	ENSMUSG00000008167	Transcript	ENSMUST00000142036	processed_transcript	rs47708877
8:85070850-85070850	downstream_gene_variant	MODIFIER	Wdr83	ENSMUSG00000005150	Transcript	ENSMUST00000142201	protein_coding	rs47708877
8:85070850-85070850	upstream_gene_variant	MODIFIER	Dhps	ENSMUSG00000060038	Transcript	ENSMUST00000142210	retained_intron	rs47708877
8:85070850-85070850	upstream_gene_variant	MODIFIER	Dhps	ENSMUSG00000060038	Transcript	ENSMUST00000142422	retained_intron	rs47708877
8:85070850-85070850	downstream_gene_variant	MODIFIER	Fbxw9	ENSMUSG00000008167	Transcript	ENSMUST00000143763	processed_transcript	rs47708877
8:85070850-85070850	downstream_gene_variant	MODIFIER	Wdr83	ENSMUSG00000005150	Transcript	ENSMUST00000145880	retained_intron	rs47708877
8:85070850-85070850	downstream_gene_variant	MODIFIER	Wdr83	ENSMUSG00000005150	Transcript	ENSMUST00000146900	retained_intron	rs47708877
8:85070850-85070850	downstream_gene_variant	MODIFIER	Fbxw9	ENSMUSG00000008167	Transcript	ENSMUST00000151962	retained_intron	rs47708877
8:85070850-85070850	downstream_gene_variant	MODIFIER	Wdr83	ENSMUSG00000005150	Transcript	ENSMUST00000152186	retained_intron	rs47708877
8:85070850-85070850	downstream_gene_variant	MODIFIER	Wdr83	ENSMUSG00000005150	Transcript	ENSMUST00000152785	protein_coding	rs47708877
8:85070850-85070850	upstream_gene_variant	MODIFIER	Dhps	ENSMUSG00000060038	Transcript	ENSMUST00000152871	nonsense_mediated_decay	rs47708877
8:85070850-85070850	downstream_gene_variant	MODIFIER	Fbxw9	ENSMUSG00000008167	Transcript	ENSMUST00000152884	retained_intron	rs47708877
8:85070850-85070850	upstream_gene_variant	MODIFIER	Dhps	ENSMUSG00000060038	Transcript	ENSMUST00000154575	retained_intron	rs47708877
8:85070850-85070850	upstream_gene_variant	MODIFIER	Gm5741	ENSMUSG00000095845	Transcript	ENSMUST00000177563	protein_coding	rs47708877
8:85070850-85070850	regulatory_region_variant	MODIFIER	-	-	RegulatoryFeature	ENSMUSR00000262313	promoter	rs47708877
8:85071737-85071737	upstream_gene_variant	MODIFIER	Dhps	ENSMUSG00000060038	Transcript	ENSMUST00000078665	protein_coding	rs3676934
8:85071737-85071737	downstream_gene_variant	MODIFIER	Wdr83	ENSMUSG00000005150	Transcript	ENSMUST00000093357	protein_coding	rs3676934
8:85071737-85071737	downstream_gene_variant	MODIFIER	Fbxw9	ENSMUSG00000008167	Transcript	ENSMUST00000095220	protein_coding	rs3676934
8:85071737-85071737	upstream_gene_variant	MODIFIER	Dhps	ENSMUSG00000060038	Transcript	ENSMUST00000129826	retained_intron	rs3676934
8:85071737-85071737	downstream_gene_variant	MODIFIER	Wdr83	ENSMUSG00000005150	Transcript	ENSMUST00000131909	retained_intron	rs3676934
8:85071737-85071737	upstream_gene_variant	MODIFIER	Dhps	ENSMUSG00000060038	Transcript	ENSMUST00000132356	retained_intron	rs3676934
8:85071737-85071737	upstream_gene_variant	MODIFIER	Dhps	ENSMUSG00000060038	Transcript	ENSMUST00000132766	retained_intron	rs3676934
8:85071737-85071737	downstream_gene_variant	MODIFIER	Wdr83	ENSMUSG00000005150	Transcript	ENSMUST00000136696	retained_intron	rs3676934
8:85071737-85071737	downstream_gene_variant	MODIFIER	Fbxw9	ENSMUSG00000008167	Transcript	ENSMUST00000139721	retained_intron	rs3676934
8:85071737-85071737	downstream_gene_variant	MODIFIER	Fbxw9	ENSMUSG00000008167	Transcript	ENSMUST00000141183	retained_intron	rs3676934
8:85071737-85071737	downstream_gene_variant	MODIFIER	Fbxw9	ENSMUSG00000008167	Transcript	ENSMUST00000142036	processed_transcript	rs3676934
8:85071737-85071737	downstream_gene_variant	MODIFIER	Wdr83	ENSMUSG00000005150	Transcript	ENSMUST00000142201	protein_coding	rs3676934
8:85071737-85071737	upstream_gene_variant	MODIFIER	Dhps	ENSMUSG00000060038	Transcript	ENSMUST00000142210	retained_intron	rs3676934
8:85071737-85071737	upstream_gene_variant	MODIFIER	Dhps	ENSMUSG00000060038	Transcript	ENSMUST00000142422	retained_intron	rs3676934
8:85071737-85071737	downstream_gene_variant	MODIFIER	Wdr83	ENSMUSG00000005150	Transcript	ENSMUST00000145880	retained_intron	rs3676934
8:85071737-85071737	downstream_gene_variant	MODIFIER	Wdr83	ENSMUSG00000005150	Transcript	ENSMUST00000146900	retained_intron	rs3676934
8:85071737-85071737	downstream_gene_variant	MODIFIER	Wdr83	ENSMUSG00000005150	Transcript	ENSMUST00000149050	nonsense_mediated_decay	rs3676934
8:85071737-85071737	downstream_gene_variant	MODIFIER	Wdr83	ENSMUSG00000005150	Transcript	ENSMUST00000152186	retained_intron	rs3676934
8:85071737-85071737	downstream_gene_variant	MODIFIER	Wdr83	ENSMUSG00000005150	Transcript	ENSMUST00000152785	protein_coding	rs3676934
8:85071737-85071737	upstream_gene_variant	MODIFIER	Dhps	ENSMUSG00000060038	Transcript	ENSMUST00000152871	nonsense_mediated_decay	rs3676934
8:85071737-85071737	downstream_gene_variant	MODIFIER	Fbxw9	ENSMUSG00000008167	Transcript	ENSMUST00000152884	retained_intron	rs3676934
8:85071737-85071737	upstream_gene_variant	MODIFIER	Dhps	ENSMUSG00000060038	Transcript	ENSMUST00000154575	retained_intron	rs3676934
8:85071737-85071737	upstream_gene_variant	MODIFIER	Gm5741	ENSMUSG00000095845	Transcript	ENSMUST00000177563	protein_coding	rs3676934
8:85071737-85071737	regulatory_region_variant	MODIFIER	-	-	RegulatoryFeature	ENSMUSR00000262313	promoter	rs3676934
8:85072796-85072796	intron_variant	MODIFIER	Dhps	ENSMUSG00000060038	Transcript	ENSMUST00000078665	protein_coding	rs244342510
8:85072796-85072796	downstream_gene_variant	MODIFIER	Wdr83	ENSMUSG00000005150	Transcript	ENSMUST00000093357	protein_coding	rs244342510
8:85072796-85072796	non_coding_transcript_exon_va	MODIFIER	Dhps	ENSMUSG00000060038	Transcript	ENSMUST00000129826	retained_intron	rs244342510
8:85072796-85072796	downstream_gene_variant	MODIFIER	Wdr83	ENSMUSG00000005150	Transcript	ENSMUST00000131909	retained_intron	rs244342510
8:85072796-85072796	upstream_gene_variant	MODIFIER	Dhps	ENSMUSG00000060038	Transcript	ENSMUST00000132356	retained_intron	rs244342510
8:85072796-85072796	upstream_gene_variant	MODIFIER	Dhps	ENSMUSG00000060038	Transcript	ENSMUST00000132766	retained_intron	rs244342510
8:85072796-85072796	downstream_gene_variant	MODIFIER	Wdr83	ENSMUSG00000005150	Transcript	ENSMUST00000136696	retained_intron	rs244342510
8:85072796-85072796	downstream_gene_variant	MODIFIER	Wdr83	ENSMUSG00000005150	Transcript	ENSMUST00000142201	protein_coding	rs244342510
8:85072796-85072796	intron_variant,non_coding_trans	MODIFIER	Dhps	ENSMUSG00000060038	Transcript	ENSMUST00000142210	retained_intron	rs244342510
8:85072796-85072796	upstream_gene_variant	MODIFIER	Dhps	ENSMUSG00000060038	Transcript	ENSMUST00000142422	retained_intron	rs244342510
8:85072796-85072796	downstream_gene_variant	MODIFIER	Wdr83	ENSMUSG00000005150	Transcript	ENSMUST00000145880	retained_intron	rs244342510

8:85072796-85072796	downstream_gene_variant	MODIFIER	Wdr83	ENSMUSG00000005150	Transcript	ENSMUST00000146900	retained_intron	rs244342510
8:85072796-85072796	downstream_gene_variant	MODIFIER	Wdr83	ENSMUSG00000005150	Transcript	ENSMUST00000149050	nonsense_mediated_decay	rs244342510
8:85072796-85072796	downstream_gene_variant	MODIFIER	Wdr83	ENSMUSG00000005150	Transcript	ENSMUST00000152186	retained_intron	rs244342510
8:85072796-85072796	downstream_gene_variant	MODIFIER	Wdr83	ENSMUSG00000005150	Transcript	ENSMUST00000152785	protein_coding	rs244342510
8:85072796-85072796	intron_variant,NMD_transcript_v	MODIFIER	Dhps	ENSMUSG00000060038	Transcript	ENSMUST00000152871	nonsense_mediated_decay	rs244342510
8:85072796-85072796	upstream_gene_variant	MODIFIER	Dhps	ENSMUSG00000060038	Transcript	ENSMUST00000154575	retained_intron	rs244342510
8:85072796-85072796	upstream_gene_variant	MODIFIER	Gm5741	ENSMUSG00000095845	Transcript	ENSMUST00000177563	protein_coding	rs244342510
8:85072796-85072796	regulatory_region_variant	MODIFIER	-	-	RegulatoryFeature	ENSMUSR00000262313	promoter	rs244342510
8:85073894-85073894	intron_variant	MODIFIER	Dhps	ENSMUSG00000060038	Transcript	ENSMUST00000078665	protein_coding	rs33590953
8:85073894-85073894	downstream_gene_variant	MODIFIER	Wdr83	ENSMUSG00000005150	Transcript	ENSMUST00000093357	protein_coding	rs33590953
8:85073894-85073894	downstream_gene_variant	MODIFIER	Dhps	ENSMUSG00000060038	Transcript	ENSMUST00000129826	retained_intron	rs33590953
8:85073894-85073894	downstream_gene_variant	MODIFIER	Wdr83	ENSMUSG00000005150	Transcript	ENSMUST00000131909	retained_intron	rs33590953
8:85073894-85073894	upstream_gene_variant	MODIFIER	Dhps	ENSMUSG00000060038	Transcript	ENSMUST00000132356	retained_intron	rs33590953
8:85073894-85073894	intron_variant,non_coding_trans	MODIFIER	Dhps	ENSMUSG00000060038	Transcript	ENSMUST00000132766	retained_intron	rs33590953
8:85073894-85073894	downstream_gene_variant	MODIFIER	Wdr83	ENSMUSG00000005150	Transcript	ENSMUST00000136696	retained_intron	rs33590953
8:85073894-85073894	downstream_gene_variant	MODIFIER	Wdr83	ENSMUSG00000005150	Transcript	ENSMUST00000142201	protein_coding	rs33590953
8:85073894-85073894	downstream_gene_variant	MODIFIER	Dhps	ENSMUSG00000060038	Transcript	ENSMUST00000142210	retained_intron	rs33590953
8:85073894-85073894	intron_variant,non_coding_trans	MODIFIER	Dhps	ENSMUSG00000060038	Transcript	ENSMUST00000142422	retained_intron	rs33590953
8:85073894-85073894	downstream_gene_variant	MODIFIER	Wdr83	ENSMUSG00000005150	Transcript	ENSMUST00000145880	retained_intron	rs33590953
8:85073894-85073894	downstream_gene_variant	MODIFIER	Wdr83	ENSMUSG00000005150	Transcript	ENSMUST00000146900	retained_intron	rs33590953
8:85073894-85073894	downstream_gene_variant	MODIFIER	Wdr83	ENSMUSG00000005150	Transcript	ENSMUST00000149050	nonsense_mediated_decay	rs33590953
8:85073894-85073894	downstream_gene_variant	MODIFIER	Wdr83	ENSMUSG00000005150	Transcript	ENSMUST00000152186	retained_intron	rs33590953
8:85073894-85073894	downstream_gene_variant	MODIFIER	Wdr83	ENSMUSG00000005150	Transcript	ENSMUST00000152785	protein_coding	rs33590953
8:85073894-85073894	intron_variant,NMD_transcript_v	MODIFIER	Dhps	ENSMUSG00000060038	Transcript	ENSMUST00000152871	nonsense_mediated_decay	rs33590953
8:85073894-85073894	non_coding_transcript_exon_vai	MODIFIER	Dhps	ENSMUSG00000060038	Transcript	ENSMUST00000154575	retained_intron	rs33590953
8:85076315-85076315	downstream_gene_variant	MODIFIER	Dhps	ENSMUSG00000060038	Transcript	ENSMUST00000078665	protein_coding	rs260826795
8:85076315-85076315	upstream_gene_variant	MODIFIER	Wdr83os	ENSMUSG00000059355	Transcript	ENSMUST00000079764	protein_coding	rs260826795
8:85076315-85076315	intron_variant	MODIFIER	Wdr83	ENSMUSG00000005150	Transcript	ENSMUST00000093357	protein_coding	rs260826795
8:85076315-85076315	downstream_gene_variant	MODIFIER	Dhps	ENSMUSG00000060038	Transcript	ENSMUST00000129826	retained_intron	rs260826795
8:85076315-85076315	intron_variant,non_coding_trans	MODIFIER	Wdr83	ENSMUSG00000005150	Transcript	ENSMUST00000131909	retained_intron	rs260826795
8:85076315-85076315	downstream_gene_variant	MODIFIER	Dhps	ENSMUSG00000060038	Transcript	ENSMUST00000132356	retained_intron	rs260826795
8:85076315-85076315	downstream_gene_variant	MODIFIER	Dhps	ENSMUSG00000060038	Transcript	ENSMUST00000132766	retained_intron	rs260826795
8:85076315-85076315	downstream_gene_variant	MODIFIER	Wdr83	ENSMUSG00000005150	Transcript	ENSMUST00000135463	retained_intron	rs260826795
8:85076315-85076315	intron_variant,non_coding_trans	MODIFIER	Wdr83	ENSMUSG00000005150	Transcript	ENSMUST00000136696	retained_intron	rs260826795
8:85076315-85076315	upstream_gene_variant	MODIFIER	Wdr83os	ENSMUSG00000059355	Transcript	ENSMUST00000140621	protein_coding	rs260826795
8:85076315-85076315	upstream_gene_variant	MODIFIER	Wdr83	ENSMUSG00000005150	Transcript	ENSMUST00000142201	protein_coding	rs260826795
8:85076315-85076315	downstream_gene_variant	MODIFIER	Dhps	ENSMUSG00000060038	Transcript	ENSMUST00000142210	retained_intron	rs260826795
8:85076315-85076315	downstream_gene_variant	MODIFIER	Dhps	ENSMUSG00000060038	Transcript	ENSMUST00000142422	retained_intron	rs260826795
8:85076315-85076315	intron_variant,non_coding_trans	MODIFIER	Wdr83	ENSMUSG00000005150	Transcript	ENSMUST00000145880	retained_intron	rs260826795
8:85076315-85076315	upstream_gene_variant	MODIFIER	Wdr83	ENSMUSG00000005150	Transcript	ENSMUST00000146900	retained_intron	rs260826795
8:85076315-85076315	upstream_gene_variant	MODIFIER	Wdr83os	ENSMUSG00000059355	Transcript	ENSMUST00000148592	retained_intron	rs260826795
8:85076315-85076315	intron_variant,NMD_transcript_v	MODIFIER	Wdr83	ENSMUSG00000005150	Transcript	ENSMUST00000149050	nonsense_mediated_decay	rs260826795
8:85076315-85076315	intron_variant,non_coding_trans	MODIFIER	Wdr83	ENSMUSG00000005150	Transcript	ENSMUST00000152186	retained_intron	rs260826795
8:85076315-85076315	intron_variant	MODIFIER	Wdr83	ENSMUSG00000005150	Transcript	ENSMUST00000152785	protein_coding	rs260826795
8:85076315-85076315	downstream_gene_variant	MODIFIER	Dhps	ENSMUSG00000060038	Transcript	ENSMUST00000152871	nonsense_mediated_decay	rs260826795
8:85076315-85076315	downstream_gene_variant	MODIFIER	Dhps	ENSMUSG00000060038	Transcript	ENSMUST00000154575	retained_intron	rs260826795
8:85076315-85076315	regulatory_region_variant	MODIFIER	-	-	RegulatoryFeature	ENSMUSR00000456760	CTCF_binding_site	rs260826795
8:85076315-85076315	regulatory_region_variant	MODIFIER	-	-	RegulatoryFeature	ENSMUSR00000735784	TF_binding_site	rs260826795
8:85076316-85076316	downstream_gene_variant	MODIFIER	Dhps	ENSMUSG00000060038	Transcript	ENSMUST00000078665	protein_coding	rs253086696
8:85076316-85076316	upstream_gene_variant	MODIFIER	Wdr83os	ENSMUSG00000059355	Transcript	ENSMUST00000079764	protein_coding	rs253086696
8:85076316-85076316	intron_variant	MODIFIER	Wdr83	ENSMUSG00000005150	Transcript	ENSMUST00000093357	protein_coding	rs253086696
8:85076316-85076316	downstream_gene_variant	MODIFIER	Dhps	ENSMUSG00000060038	Transcript	ENSMUST00000129826	retained_intron	rs253086696
8:85076316-85076316	intron_variant,non_coding_trans	MODIFIER	Wdr83	ENSMUSG00000005150	Transcript	ENSMUST00000131909	retained_intron	rs253086696
8:85076316-85076316	downstream_gene_variant	MODIFIER	Dhps	ENSMUSG00000060038	Transcript	ENSMUST00000132356	retained_intron	rs253086696
8:85076316-85076316	downstream_gene_variant	MODIFIER	Dhps	ENSMUSG00000060038	Transcript	ENSMUST00000132766	retained_intron	rs253086696
8:85076316-85076316	downstream_gene_variant	MODIFIER	Wdr83	ENSMUSG00000005150	Transcript	ENSMUST00000135463	retained_intron	rs253086696
8:85076316-85076316	intron_variant,non_coding_trans	MODIFIER	Wdr83	ENSMUSG00000005150	Transcript	ENSMUST00000136696	retained_intron	rs253086696
8:85076316-85076316	upstream_gene_variant	MODIFIER	Wdr83os	ENSMUSG00000059355	Transcript	ENSMUST00000140621	protein_coding	rs253086696
8:85076316-85076316	upstream_gene_variant	MODIFIER	Wdr83	ENSMUSG00000005150	Transcript	ENSMUST00000142201	protein_coding	rs253086696
8:85076316-85076316	downstream_gene_variant	MODIFIER	Dhps	ENSMUSG00000060038	Transcript	ENSMUST00000142210	retained_intron	rs253086696
8:85076316-85076316	downstream_gene_variant	MODIFIER	Dhps	ENSMUSG00000060038	Transcript	ENSMUST00000142422	retained_intron	rs253086696
8:85076316-85076316	intron_variant,non_coding_trans	MODIFIER	Wdr83	ENSMUSG00000005150	Transcript	ENSMUST00000145880	retained_intron	rs253086696
8:85076316-85076316	upstream_gene_variant	MODIFIER	Wdr83	ENSMUSG00000005150	Transcript	ENSMUST00000146900	retained_intron	rs253086696
8:85076316-85076316	upstream_gene_variant	MODIFIER	Wdr83os	ENSMUSG00000059355	Transcript	ENSMUST00000148592	retained_intron	rs253086696
8:85076316-85076316	intron_variant,NMD_transcript_v	MODIFIER	Wdr83	ENSMUSG00000005150	Transcript	ENSMUST00000149050	nonsense_mediated_decay	rs253086696
8:85076316-85076316	intron_variant,non_coding_trans	MODIFIER	Wdr83	ENSMUSG00000005150	Transcript	ENSMUST00000152186	retained_intron	rs253086696
8:85076316-85076316	intron_variant	MODIFIER	Wdr83	ENSMUSG00000005150	Transcript	ENSMUST00000152785	protein_coding	rs253086696
8:85076316-85076316	downstream_gene_variant	MODIFIER	Dhps	ENSMUSG00000060038	Transcript	ENSMUST00000152871	nonsense_mediated_decay	rs253086696
8:85076316-85076316	downstream_gene_variant	MODIFIER	Dhps	ENSMUSG00000060038	Transcript	ENSMUST00000154575	retained_intron	rs253086696

8:85076316-85076316	regulatory_region_variant	MODIFIER	-	-	RegulatoryFeature	ENSMUSR00000456760	CTCF_binding_site	rs253086696
8:85076316-85076316	regulatory_region_variant	MODIFIER	-	-	RegulatoryFeature	ENSMUSR00000735784	TF_binding_site	rs253086696
8:85078582-85078582	upstream_gene_variant	MODIFIER	Man2b1	ENSMUSG00000005142	Transcript	ENSMUST00000034121	protein_coding	rs47634958
8:85078582-85078582	downstream_gene_variant	MODIFIER	Dhps	ENSMUSG00000060038	Transcript	ENSMUST00000078665	protein_coding	rs47634958
8:85078582-85078582	upstream_gene_variant	MODIFIER	Wdr83os	ENSMUSG00000059355	Transcript	ENSMUST00000079764	protein_coding	rs47634958
8:85078582-85078582	intron_variant	MODIFIER	Wdr83	ENSMUSG00000005150	Transcript	ENSMUST00000093357	protein_coding	rs47634958
8:85078582-85078582	intron_variant,non_coding_trans	MODIFIER	Wdr83	ENSMUSG00000005150	Transcript	ENSMUST00000131909	retained_intron	rs47634958
8:85078582-85078582	downstream_gene_variant	MODIFIER	Dhps	ENSMUSG00000060038	Transcript	ENSMUST00000132356	retained_intron	rs47634958
8:85078582-85078582	downstream_gene_variant	MODIFIER	Dhps	ENSMUSG00000060038	Transcript	ENSMUST00000132766	retained_intron	rs47634958
8:85078582-85078582	downstream_gene_variant	MODIFIER	Wdr83	ENSMUSG00000005150	Transcript	ENSMUST00000135463	retained_intron	rs47634958
8:85078582-85078582	intron_variant,non_coding_trans	MODIFIER	Wdr83	ENSMUSG00000005150	Transcript	ENSMUST00000136696	retained_intron	rs47634958
8:85078582-85078582	upstream_gene_variant	MODIFIER	Wdr83os	ENSMUSG00000059355	Transcript	ENSMUST00000140621	protein_coding	rs47634958
8:85078582-85078582	upstream_gene_variant	MODIFIER	Wdr83	ENSMUSG00000005150	Transcript	ENSMUST00000142201	protein_coding	rs47634958
8:85078582-85078582	downstream_gene_variant	MODIFIER	Dhps	ENSMUSG00000060038	Transcript	ENSMUST00000142422	retained_intron	rs47634958
8:85078582-85078582	intron_variant,non_coding_trans	MODIFIER	Wdr83	ENSMUSG00000005150	Transcript	ENSMUST00000145880	retained_intron	rs47634958
8:85078582-85078582	upstream_gene_variant	MODIFIER	Wdr83	ENSMUSG00000005150	Transcript	ENSMUST00000146900	retained_intron	rs47634958
8:85078582-85078582	upstream_gene_variant	MODIFIER	Wdr83os	ENSMUSG00000059355	Transcript	ENSMUST00000148592	retained_intron	rs47634958
8:85078582-85078582	intron_variant,NMD_transcript_v	MODIFIER	Wdr83	ENSMUSG00000005150	Transcript	ENSMUST00000149050	nonsense_mediated_decay	rs47634958
8:85078582-85078582	intron_variant,non_coding_trans	MODIFIER	Wdr83	ENSMUSG00000005150	Transcript	ENSMUST00000152186	retained_intron	rs47634958
8:85078582-85078582	intron_variant	MODIFIER	Wdr83	ENSMUSG00000005150	Transcript	ENSMUST00000152785	protein_coding	rs47634958
8:85078582-85078582	downstream_gene_variant	MODIFIER	Dhps	ENSMUSG00000060038	Transcript	ENSMUST00000152871	nonsense_mediated_decay	rs47634958
8:85078582-85078582	downstream_gene_variant	MODIFIER	Dhps	ENSMUSG00000060038	Transcript	ENSMUST00000154575	retained_intron	rs47634958
8:85078582-85078582	upstream_gene_variant	MODIFIER	Man2b1	ENSMUSG00000005142	Transcript	ENSMUST00000209264	nonsense_mediated_decay	rs47634958
8:85078582-85078582	upstream_gene_variant	MODIFIER	Man2b1	ENSMUSG00000005142	Transcript	ENSMUST00000209361	nonsense_mediated_decay	rs47634958
8:85078582-85078582	upstream_gene_variant	MODIFIER	Man2b1	ENSMUSG00000005142	Transcript	ENSMUST00000210991	retained_intron	rs47634958
8:85086501-85086501	intron_variant	MODIFIER	Man2b1	ENSMUSG00000005142	Transcript	ENSMUST00000034121	protein_coding	rs33475081
8:85086501-85086501	downstream_gene_variant	MODIFIER	Wdr83os	ENSMUSG00000059355	Transcript	ENSMUST00000079764	protein_coding	rs33475081
8:85086501-85086501	downstream_gene_variant	MODIFIER	Wdr83os	ENSMUSG00000059355	Transcript	ENSMUST00000140621	protein_coding	rs33475081
8:85086501-85086501	downstream_gene_variant	MODIFIER	Wdr83os	ENSMUSG00000059355	Transcript	ENSMUST00000148592	retained_intron	rs33475081
8:85086501-85086501	downstream_gene_variant	MODIFIER	Man2b1	ENSMUSG00000005142	Transcript	ENSMUST00000209264	nonsense_mediated_decay	rs33475081
8:85086501-85086501	intron_variant,NMD_transcript_v	MODIFIER	Man2b1	ENSMUSG00000005142	Transcript	ENSMUST00000209361	nonsense_mediated_decay	rs33475081
8:85086501-85086501	downstream_gene_variant	MODIFIER	Man2b1	ENSMUSG00000005142	Transcript	ENSMUST00000210991	retained_intron	rs33475081
8:85086501-85086501	non_coding_transcript_exon_var	MODIFIER	Man2b1	ENSMUSG00000005142	Transcript	ENSMUST00000211223	retained_intron	rs33475081
8:85086501-85086501	non_coding_transcript_exon_var	MODIFIER	Man2b1	ENSMUSG00000005142	Transcript	ENSMUST00000211379	retained_intron	rs33475081
8:85086501-85086501	regulatory_region_variant	MODIFIER	-	-	RegulatoryFeature	ENSMUSR00000456763	CTCF_binding_site	rs33475081
8:85087476-85087476	intron_variant	MODIFIER	Man2b1	ENSMUSG00000005142	Transcript	ENSMUST00000034121	protein_coding	rs33242493
8:85087476-85087476	downstream_gene_variant	MODIFIER	Man2b1	ENSMUSG00000005142	Transcript	ENSMUST00000209264	nonsense_mediated_decay	rs33242493
8:85087476-85087476	downstream_gene_variant	MODIFIER	Man2b1	ENSMUSG00000005142	Transcript	ENSMUST00000209361	nonsense_mediated_decay	rs33242493
8:85087476-85087476	downstream_gene_variant	MODIFIER	Man2b1	ENSMUSG00000005142	Transcript	ENSMUST00000210991	retained_intron	rs33242493
8:85087476-85087476	intron_variant,non_coding_trans	MODIFIER	Man2b1	ENSMUSG00000005142	Transcript	ENSMUST00000211223	retained_intron	rs33242493
8:85087476-85087476	intron_variant,non_coding_trans	MODIFIER	Man2b1	ENSMUSG00000005142	Transcript	ENSMUST00000211379	retained_intron	rs33242493
8:85091632-85091632	intron_variant	MODIFIER	Man2b1	ENSMUSG00000005142	Transcript	ENSMUST00000034121	protein_coding	rs32725145
8:85091632-85091632	downstream_gene_variant	MODIFIER	Man2b1	ENSMUSG00000005142	Transcript	ENSMUST00000209361	nonsense_mediated_decay	rs32725145
8:85091632-85091632	downstream_gene_variant	MODIFIER	Man2b1	ENSMUSG00000005142	Transcript	ENSMUST00000211223	retained_intron	rs32725145
8:85091632-85091632	downstream_gene_variant	MODIFIER	Man2b1	ENSMUSG00000005142	Transcript	ENSMUST00000211379	retained_intron	rs32725145
8:85094652-85094658	intron_variant	MODIFIER	Man2b1	ENSMUSG00000005142	Transcript	ENSMUST00000034121	protein_coding	rs247191075
8:85094652-85094658	downstream_gene_variant	MODIFIER	Man2b1	ENSMUSG00000005142	Transcript	ENSMUST00000211379	retained_intron	rs247191075
8:85303054-85303054	upstream_gene_variant	MODIFIER	Vps35	ENSMUSG00000031696	Transcript	ENSMUST00000034131	protein_coding	rs32998051
8:85303054-85303054	intron_variant	MODIFIER	Orc6	ENSMUSG00000031697	Transcript	ENSMUST00000034132	protein_coding	rs32998051
8:85303054-85303054	intron_variant	MODIFIER	Orc6	ENSMUSG00000031697	Transcript	ENSMUST00000170141	protein_coding	rs32998051
8:85303054-85303054	upstream_gene_variant	MODIFIER	Vps35	ENSMUSG00000031696	Transcript	ENSMUST00000209228	processed_transcript	rs32998051
8:85303054-85303054	downstream_gene_variant	MODIFIER	Orc6	ENSMUSG00000031697	Transcript	ENSMUST00000209733	retained_intron	rs32998051
8:85303054-85303054	intron_variant,NMD_transcript_v	MODIFIER	Orc6	ENSMUSG00000031697	Transcript	ENSMUST00000210146	nonsense_mediated_decay	rs32998051
8:85303054-85303054	upstream_gene_variant	MODIFIER	Orc6	ENSMUSG00000031697	Transcript	ENSMUST00000210458	retained_intron	rs32998051
8:85303054-85303054	upstream_gene_variant	MODIFIER	Vps35	ENSMUSG00000031696	Transcript	ENSMUST00000211154	processed_transcript	rs32998051
8:85303054-85303054	intron_variant	MODIFIER	Orc6	ENSMUSG00000031697	Transcript	ENSMUST00000211396	protein_coding	rs32998051
8:85303054-85303054	upstream_gene_variant	MODIFIER	Vps35	ENSMUSG00000031696	Transcript	ENSMUST00000211479	retained_intron	rs32998051
8:85303054-85303054	intron_variant	MODIFIER	Orc6	ENSMUSG00000031697	Transcript	ENSMUST00000211597	protein_coding	rs32998051
8:85518021-85518021	synonymous_variant	LOW	Gpt2	ENSMUSG00000031700	Transcript	ENSMUST00000034136	protein_coding	rs13472149
8:85518021-85518021	3_prime_UTR_variant,NMD_tran	MODIFIER	Gpt2	ENSMUSG00000031700	Transcript	ENSMUST00000132932	nonsense_mediated_decay	rs13472149
8:85518021-85518021	non_coding_transcript_exon_var	MODIFIER	Gpt2	ENSMUSG00000031700	Transcript	ENSMUST00000140189	retained_intron	rs13472149
8:85518021-85518021	upstream_gene_variant	MODIFIER	Gpt2	ENSMUSG00000031700	Transcript	ENSMUST00000143846	processed_transcript	rs13472149
8:85521350-85521350	synonymous_variant	LOW	Gpt2	ENSMUSG00000031700	Transcript	ENSMUST00000034136	protein_coding	rs32795953
8:85521350-85521350	3_prime_UTR_variant,NMD_tran	MODIFIER	Gpt2	ENSMUSG00000031700	Transcript	ENSMUST00000132932	nonsense_mediated_decay	rs32795953
8:85521350-85521350	non_coding_transcript_exon_var	MODIFIER	Gpt2	ENSMUSG00000031700	Transcript	ENSMUST00000140189	retained_intron	rs32795953
8:85521350-85521350	non_coding_transcript_exon_var	MODIFIER	Gpt2	ENSMUSG00000031700	Transcript	ENSMUST00000143846	processed_transcript	rs32795953
8:85526930-85526930	3_prime_UTR_variant	MODIFIER	Gpt2	ENSMUSG00000031700	Transcript	ENSMUST00000034136	protein_coding	rs4227299
8:85526930-85526930	downstream_gene_variant	MODIFIER	Gpt2	ENSMUSG00000031700	Transcript	ENSMUST00000132932	nonsense_mediated_decay	rs4227299
8:85526930-85526930	downstream_gene_variant	MODIFIER	Gpt2	ENSMUSG00000031700	Transcript	ENSMUST00000143846	processed_transcript	rs4227299

8:85540615-85540615	intron_variant	MODIFIER	Dnaja2	ENSMUSG00000031701	Transcript	ENSMUST00000034138	protein_coding	rs33211735
8:85541016-85541016	intron_variant	MODIFIER	Dnaja2	ENSMUSG00000031701	Transcript	ENSMUST00000034138	protein_coding	rs33279848
8:85541636-85541636	intron_variant	MODIFIER	Dnaja2	ENSMUSG00000031701	Transcript	ENSMUST00000034138	protein_coding	rs51956125
8:85541961-85541961	intron_variant	MODIFIER	Dnaja2	ENSMUSG00000031701	Transcript	ENSMUST00000034138	protein_coding	rs36282735
8:85541961-85541961	downstream_gene_variant	MODIFIER	Dnaja2	ENSMUSG00000031701	Transcript	ENSMUST00000211630	retained_intron	rs36282735
8:85542118-85542118	intron_variant	MODIFIER	Dnaja2	ENSMUSG00000031701	Transcript	ENSMUST00000034138	protein_coding	rs50206689
8:85542118-85542118	downstream_gene_variant	MODIFIER	Dnaja2	ENSMUSG00000031701	Transcript	ENSMUST00000211630	retained_intron	rs50206689
8:85542597-85542597	intron_variant	MODIFIER	Dnaja2	ENSMUSG00000031701	Transcript	ENSMUST00000034138	protein_coding	rs36298745
8:85542597-85542597	downstream_gene_variant	MODIFIER	Dnaja2	ENSMUSG00000031701	Transcript	ENSMUST00000211630	retained_intron	rs36298745
8:85544503-85544503	intron_variant	MODIFIER	Dnaja2	ENSMUSG00000031701	Transcript	ENSMUST00000034138	protein_coding	rs246589420
8:85544503-85544503	downstream_gene_variant	MODIFIER	Dnaja2	ENSMUSG00000031701	Transcript	ENSMUST00000211630	retained_intron	rs246589420
8:85546289-85546289	intron_variant	MODIFIER	Dnaja2	ENSMUSG00000031701	Transcript	ENSMUST00000034138	protein_coding	rs33171266
8:85546289-85546289	downstream_gene_variant	MODIFIER	Dnaja2	ENSMUSG00000031701	Transcript	ENSMUST00000211630	retained_intron	rs33171266
8:85554241-85554241	intron_variant	MODIFIER	Dnaja2	ENSMUSG00000031701	Transcript	ENSMUST00000034138	protein_coding	rs33134687
8:85554241-85554241	upstream_gene_variant	MODIFIER	Dnaja2	ENSMUSG00000031701	Transcript	ENSMUST00000211630	retained_intron	rs33134687
8:85554241-85554241	regulatory_region_variant	MODIFIER	-	-	RegulatoryFeature	ENSMUSR00000262417	promoter	rs33134687
8:85841291-85841291	upstream_gene_variant	MODIFIER	Itfg1	ENSMUSG00000031703	Transcript	ENSMUST00000034140	protein_coding	rs33463582
8:85841291-85841291	intron_variant	MODIFIER	Phkb	ENSMUSG00000036879	Transcript	ENSMUST00000053771	protein_coding	rs33463582
8:85841291-85841291	intron_variant,non_coding_trans	MODIFIER	Phkb	ENSMUSG00000036879	Transcript	ENSMUST00000159716	processed_transcript	rs33463582
8:85841291-85841291	intron_variant,non_coding_trans	MODIFIER	Phkb	ENSMUSG00000036879	Transcript	ENSMUST00000159882	processed_transcript	rs33463582
8:85841291-85841291	intron_variant,NMD_transcript_v	MODIFIER	Phkb	ENSMUSG00000036879	Transcript	ENSMUST00000160611	nonsense_mediated_decay	rs33463582
8:85841291-85841291	intron_variant	MODIFIER	Phkb	ENSMUSG00000036879	Transcript	ENSMUST00000161850	protein_coding	rs33463582
8:85841291-85841291	upstream_gene_variant	MODIFIER	Phkb	ENSMUSG00000036879	Transcript	ENSMUST00000162464	protein_coding	rs33463582
8:85841291-85841291	upstream_gene_variant	MODIFIER	Phkb	ENSMUSG00000036879	Transcript	ENSMUST00000162981	nonsense_mediated_decay	rs33463582
8:85841291-85841291	regulatory_region_variant	MODIFIER	-	-	RegulatoryFeature	ENSMUSR00000262450	promoter	rs33463582
8:85842275-85842275	upstream_gene_variant	MODIFIER	Itfg1	ENSMUSG00000031703	Transcript	ENSMUST00000034140	protein_coding	rs47203178
8:85842275-85842275	intron_variant	MODIFIER	Phkb	ENSMUSG00000036879	Transcript	ENSMUST00000053771	protein_coding	rs47203178
8:85842275-85842275	intron_variant,non_coding_trans	MODIFIER	Phkb	ENSMUSG00000036879	Transcript	ENSMUST00000159716	processed_transcript	rs47203178
8:85842275-85842275	intron_variant,non_coding_trans	MODIFIER	Phkb	ENSMUSG00000036879	Transcript	ENSMUST00000159882	processed_transcript	rs47203178
8:85842275-85842275	intron_variant,NMD_transcript_v	MODIFIER	Phkb	ENSMUSG00000036879	Transcript	ENSMUST00000160611	nonsense_mediated_decay	rs47203178
8:85842275-85842275	intron_variant	MODIFIER	Phkb	ENSMUSG00000036879	Transcript	ENSMUST00000161850	protein_coding	rs47203178
8:85842275-85842275	upstream_gene_variant	MODIFIER	Phkb	ENSMUSG00000036879	Transcript	ENSMUST00000162464	protein_coding	rs47203178
8:85842275-85842275	upstream_gene_variant	MODIFIER	Phkb	ENSMUSG00000036879	Transcript	ENSMUST00000162981	nonsense_mediated_decay	rs47203178
8:85842275-85842275	regulatory_region_variant	MODIFIER	-	-	RegulatoryFeature	ENSMUSR00000735876	promoter	rs47203178
8:85937416-85937416	intron_variant	MODIFIER	Phkb	ENSMUSG00000036879	Transcript	ENSMUST00000053771	protein_coding	rs49468284
8:85937416-85937416	downstream_gene_variant	MODIFIER	Phkb	ENSMUSG00000036879	Transcript	ENSMUST00000160611	nonsense_mediated_decay	rs49468284
8:85937416-85937416	regulatory_region_variant	MODIFIER	-	-	RegulatoryFeature	ENSMUSR00000735884	open_chromatin_region	rs49468284
8:88163759-88163759	intron_variant	MODIFIER	Heatr3	ENSMUSG00000031657	Transcript	ENSMUST00000034079	protein_coding	rs48244720
8:88163759-88163759	intron_variant	MODIFIER	Heatr3	ENSMUSG00000031657	Transcript	ENSMUST00000121949	protein_coding	rs48244720
8:88163759-88163759	upstream_gene_variant	MODIFIER	Heatr3	ENSMUSG00000031657	Transcript	ENSMUST00000137452	retained_intron	rs48244720
8:88163759-88163759	intron_variant,non_coding_trans	MODIFIER	Heatr3	ENSMUSG00000031657	Transcript	ENSMUST00000146946	retained_intron	rs48244720
8:88255035-88255035	intron_variant	MODIFIER	Papd5	ENSMUSG00000036779	Transcript	ENSMUST00000066748	protein_coding	rs33154920
8:88255035-88255035	intron_variant	MODIFIER	Papd5	ENSMUSG00000036779	Transcript	ENSMUST00000118952	protein_coding	rs33154920
8:88255035-88255035	intron_variant	MODIFIER	Papd5	ENSMUSG00000036779	Transcript	ENSMUST00000119033	protein_coding	rs33154920
8:88255035-88255035	intron_variant	MODIFIER	Papd5	ENSMUSG00000036779	Transcript	ENSMUST00000154115	protein_coding	rs33154920
8:97164648-97164648	upstream_gene_variant	MODIFIER	Gm45703	ENSMUSG00000110567	Transcript	ENSMUST00000212293	lincRNA	-
8:97164648-97164648	non_coding_transcript_exon_va	MODIFIER	Gm5131	ENSMUSG00000110715	Transcript	ENSMUST00000212776	processed_pseudogene	-
9:35070069-35070069	intron_variant	MODIFIER	St3gal4	ENSMUSG00000032038	Transcript	ENSMUST00000034537	protein_coding	rs238036721
9:35070069-35070069	intron_variant	MODIFIER	St3gal4	ENSMUSG00000032038	Transcript	ENSMUST00000213526	protein_coding	rs238036721
9:35070069-35070069	intron_variant	MODIFIER	St3gal4	ENSMUSG00000032038	Transcript	ENSMUST00000214526	protein_coding	rs238036721
9:35070069-35070069	intron_variant	MODIFIER	St3gal4	ENSMUSG00000032038	Transcript	ENSMUST00000215089	protein_coding	rs238036721
9:35070069-35070069	intron_variant	MODIFIER	St3gal4	ENSMUSG00000032038	Transcript	ENSMUST00000215638	protein_coding	rs238036721
9:35070069-35070069	intron_variant	MODIFIER	St3gal4	ENSMUSG00000032038	Transcript	ENSMUST00000216557	protein_coding	rs238036721
9:35070069-35070069	intron_variant	MODIFIER	St3gal4	ENSMUSG00000032038	Transcript	ENSMUST00000217149	protein_coding	rs238036721
9:35070069-35070069	intron_variant	MODIFIER	St3gal4	ENSMUSG00000032038	Transcript	ENSMUST00000217542	protein_coding	rs238036721
9:35098214-35098214	intron_variant	MODIFIER	St3gal4	ENSMUSG00000032038	Transcript	ENSMUST00000034537	protein_coding	rs46286313
9:35098214-35098214	intron_variant	MODIFIER	St3gal4	ENSMUSG00000032038	Transcript	ENSMUST00000213526	protein_coding	rs46286313
9:35098214-35098214	intron_variant	MODIFIER	St3gal4	ENSMUSG00000032038	Transcript	ENSMUST00000214526	protein_coding	rs46286313
9:35098214-35098214	intron_variant	MODIFIER	St3gal4	ENSMUSG00000032038	Transcript	ENSMUST00000215089	protein_coding	rs46286313
9:35098214-35098214	intron_variant	MODIFIER	St3gal4	ENSMUSG00000032038	Transcript	ENSMUST00000216557	protein_coding	rs46286313
9:35098214-35098214	intron_variant	MODIFIER	St3gal4	ENSMUSG00000032038	Transcript	ENSMUST00000217149	protein_coding	rs46286313
9:35098326-35098326	intron_variant	MODIFIER	St3gal4	ENSMUSG00000032038	Transcript	ENSMUST00000034537	protein_coding	-
9:35098326-35098326	intron_variant	MODIFIER	St3gal4	ENSMUSG00000032038	Transcript	ENSMUST00000213526	protein_coding	-
9:35098326-35098326	intron_variant	MODIFIER	St3gal4	ENSMUSG00000032038	Transcript	ENSMUST00000214526	protein_coding	-
9:35098326-35098326	intron_variant	MODIFIER	St3gal4	ENSMUSG00000032038	Transcript	ENSMUST00000215089	protein_coding	-
9:35098326-35098326	intron_variant	MODIFIER	St3gal4	ENSMUSG00000032038	Transcript	ENSMUST00000216557	protein_coding	-
9:35098326-35098326	intron_variant	MODIFIER	St3gal4	ENSMUSG00000032038	Transcript	ENSMUST00000217149	protein_coding	-
9:36738780-36738780	intron_variant	MODIFIER	Stt3a	ENSMUSG00000032116	Transcript	ENSMUST00000120381	protein_coding	rs30382764
9:36738780-36738780	intron_variant	MODIFIER	Stt3a	ENSMUSG00000032116	Transcript	ENSMUST00000135934	protein_coding	rs30382764

9:37348134-37348134	5_prime_UTR_variant	MODIFIER	Ccdc15	ENSMUSG00000034303	Transcript	ENSMUST00000037275	protein_coding	rs30280017
9:37348134-37348134	intron_variant	MODIFIER	Ccdc15	ENSMUSG00000034303	Transcript	ENSMUST00000213633	protein_coding	rs30280017
9:37348134-37348134	5_prime_UTR_variant,NMD_trar	MODIFIER	Ccdc15	ENSMUSG00000034303	Transcript	ENSMUST00000215116	nonsense_mediated_decay	rs30280017
9:37348134-37348134	intron_variant	MODIFIER	Ccdc15	ENSMUSG00000034303	Transcript	ENSMUST00000216042	protein_coding	rs30280017
9:37348134-37348134	non_coding_transcript_exon_va	MODIFIER	Ccdc15	ENSMUSG00000034303	Transcript	ENSMUST00000216060	retained_intron	rs30280017
9:37348134-37348134	5_prime_UTR_variant	MODIFIER	Ccdc15	ENSMUSG00000034303	Transcript	ENSMUST00000217238	protein_coding	rs30280017
9:37348134-37348134	regulatory_region_variant	MODIFIER	-	-	RegulatoryFeature	ENSMUSR00000271801	promoter	rs30280017
9:37651591-37651591	downstream_gene_variant	MODIFIER	Siae	ENSMUSG00000001942	Transcript	ENSMUST00000002007	protein_coding	rs225251701
9:37651591-37651591	intron_variant	MODIFIER	Tbrg1	ENSMUSG00000011114	Transcript	ENSMUST00000117654	protein_coding	rs225251701
9:37651591-37651591	downstream_gene_variant	MODIFIER	Tbrg1	ENSMUSG00000011114	Transcript	ENSMUST00000126062	retained_intron	rs225251701
9:37651591-37651591	downstream_gene_variant	MODIFIER	Tbrg1	ENSMUSG00000011114	Transcript	ENSMUST00000134711	retained_intron	rs225251701
9:37651591-37651591	intron_variant,NMD_transcript_v	MODIFIER	Tbrg1	ENSMUSG00000011114	Transcript	ENSMUST00000142736	nonsense_mediated_decay	rs225251701
9:37651591-37651591	downstream_gene_variant	MODIFIER	Siae	ENSMUSG00000001942	Transcript	ENSMUST00000213126	protein_coding	rs225251701
9:37651591-37651591	downstream_gene_variant	MODIFIER	Siae	ENSMUSG00000001942	Transcript	ENSMUST00000215474	protein_coding	rs225251701
9:65902572-65902572	3_prime_UTR_variant	MODIFIER	Pclaf	ENSMUSG00000040204	Transcript	ENSMUST00000045802	protein_coding	-
9:65902572-65902572	downstream_gene_variant	MODIFIER	Gm23248	ENSMUSG00000065301	Transcript	ENSMUST00000083367	snRNA	-
9:65902572-65902572	intron_variant	MODIFIER	Trip4	ENSMUSG00000032386	Transcript	ENSMUST00000119245	protein_coding	-
9:65902572-65902572	intron_variant,non_coding_trans	MODIFIER	Trip4	ENSMUSG00000032386	Transcript	ENSMUST00000126517	processed_transcript	-
9:65902572-65902572	intron_variant,non_coding_trans	MODIFIER	Trip4	ENSMUSG00000032386	Transcript	ENSMUST00000133747	processed_transcript	-
9:65902572-65902572	intron_variant	MODIFIER	Trip4	ENSMUSG00000032386	Transcript	ENSMUST00000134338	protein_coding	-
9:65902572-65902572	intron_variant,non_coding_trans	MODIFIER	Trip4	ENSMUSG00000032386	Transcript	ENSMUST00000139346	retained_intron	-
9:65902572-65902572	intron_variant,non_coding_trans	MODIFIER	Trip4	ENSMUSG00000032386	Transcript	ENSMUST00000143864	processed_transcript	-
9:65902572-65902572	intron_variant	MODIFIER	Trip4	ENSMUSG00000032386	Transcript	ENSMUST00000179395	protein_coding	-
10:112925433-112925433	3_prime_UTR_variant	MODIFIER	Atxn7l3b	ENSMUSG00000074748	Transcript	ENSMUST00000099276	protein_coding	-
10:112925433-112925433	downstream_gene_variant	MODIFIER	Atxn7l3b	ENSMUSG00000074748	Transcript	ENSMUST00000165438	processed_transcript	-
10:112925433-112925433	upstream_gene_variant	MODIFIER	Gm26596	ENSMUSG00000097185	Transcript	ENSMUST00000180464	TEC	-
10:4154510-4154510	intron_variant	MODIFIER	Mthfd1l	ENSMUSG00000040675	Transcript	ENSMUST00000043735	protein_coding	rs29374712
10:4154510-4154510	intron_variant	MODIFIER	Mthfd1l	ENSMUSG00000040675	Transcript	ENSMUST00000117291	protein_coding	rs29374712
10:4154510-4154510	intron_variant	MODIFIER	Mthfd1l	ENSMUSG00000040675	Transcript	ENSMUST00000120585	protein_coding	rs29374712
10:4154510-4154510	regulatory_region_variant	MODIFIER	-	-	RegulatoryFeature	ENSMUSR00000496730	enhancer	rs29374712
10:4398164-4398164	downstream_gene_variant	MODIFIER	Rmnd1	ENSMUSG00000019763	Transcript	ENSMUST00000042251	protein_coding	rs29357159
10:4398164-4398164	downstream_gene_variant	MODIFIER	Gm21781	ENSMUSG00000095123	Transcript	ENSMUST00000178920	lincRNA	rs29357159
10:4398168-4398168	downstream_gene_variant	MODIFIER	Rmnd1	ENSMUSG00000019763	Transcript	ENSMUST00000042251	protein_coding	rs29338922
10:4398168-4398168	downstream_gene_variant	MODIFIER	Gm21781	ENSMUSG00000095123	Transcript	ENSMUST00000178920	lincRNA	rs29338922
10:4432327-4432327	5_prime_UTR_variant	MODIFIER	Rmnd1	ENSMUSG00000019763	Transcript	ENSMUST00000042251	protein_coding	rs29364026
10:4432327-4432327	upstream_gene_variant	MODIFIER	Armt1	ENSMUSG00000061759	Transcript	ENSMUST00000095893	protein_coding	rs29364026
10:4432327-4432327	upstream_gene_variant	MODIFIER	Armt1	ENSMUSG00000061759	Transcript	ENSMUST00000117489	protein_coding	rs29364026
10:4432327-4432327	upstream_gene_variant	MODIFIER	Armt1	ENSMUSG00000061759	Transcript	ENSMUST00000118544	protein_coding	rs29364026
10:4432327-4432327	5_prime_UTR_variant	MODIFIER	Rmnd1	ENSMUSG00000019763	Transcript	ENSMUST00000126102	protein_coding	rs29364026
10:4432327-4432327	upstream_gene_variant	MODIFIER	Armt1	ENSMUSG00000061759	Transcript	ENSMUST00000131636	processed_transcript	rs29364026
10:4432327-4432327	5_prime_UTR_variant	MODIFIER	Rmnd1	ENSMUSG00000019763	Transcript	ENSMUST00000131853	protein_coding	rs29364026
10:4432327-4432327	upstream_gene_variant	MODIFIER	Armt1	ENSMUSG00000061759	Transcript	ENSMUST00000134484	retained_intron	rs29364026
10:4432327-4432327	upstream_gene_variant	MODIFIER	Armt1	ENSMUSG00000061759	Transcript	ENSMUST00000143037	processed_transcript	rs29364026
10:4432327-4432327	upstream_gene_variant	MODIFIER	Rmnd1	ENSMUSG00000019763	Transcript	ENSMUST00000148566	retained_intron	rs29364026
10:4432327-4432327	upstream_gene_variant	MODIFIER	Armt1	ENSMUSG00000061759	Transcript	ENSMUST00000152294	nonsense_mediated_decay	rs29364026
10:4432327-4432327	upstream_gene_variant	MODIFIER	Rmnd1	ENSMUSG00000019763	Transcript	ENSMUST00000155172	protein_coding	rs29364026
10:4432327-4432327	non_coding_transcript_exon_va	MODIFIER	Rmnd1	ENSMUSG00000019763	Transcript	ENSMUST00000156940	retained_intron	rs29364026
10:4432327-4432327	regulatory_region_variant	MODIFIER	-	-	RegulatoryFeature	ENSMUSR00000021288	promoter	rs29364026
10:77916969-77916969	intron_variant,non_coding_trans	MODIFIER	Trpm2	ENSMUSG00000009292	Transcript	ENSMUST00000105400	processed_transcript	-
10:77916969-77916969	intron_variant	MODIFIER	Trpm2	ENSMUSG00000009292	Transcript	ENSMUST00000105401	protein_coding	-
10:77916969-77916969	downstream_gene_variant	MODIFIER	Trpm2	ENSMUSG00000009292	Transcript	ENSMUST00000126206	retained_intron	-
10:77916969-77916969	non_coding_transcript_exon_va	MODIFIER	Trpm2	ENSMUSG00000009292	Transcript	ENSMUST00000138238	retained_intron	-
10:77916969-77916969	intron_variant,non_coding_trans	MODIFIER	Trpm2	ENSMUSG00000009292	Transcript	ENSMUST00000140471	retained_intron	-
10:77916969-77916969	intron_variant,non_coding_trans	MODIFIER	Trpm2	ENSMUSG00000009292	Transcript	ENSMUST00000153842	retained_intron	-
10:77916969-77916969	upstream_gene_variant	MODIFIER	Gm22871	ENSMUSG00000093443	Transcript	ENSMUST00000177097	snoRNA	-
10:77916969-77916969	upstream_gene_variant	MODIFIER	Gm22394	ENSMUSG00000093784	Transcript	ENSMUST00000177209	snoRNA	-
10:77916969-77916969	downstream_gene_variant	MODIFIER	Trpm2	ENSMUSG00000009292	Transcript	ENSMUST00000217806	retained_intron	-
10:77916969-77916969	regulatory_region_variant	MODIFIER	-	-	RegulatoryFeature	ENSMUSR00000311846	CTCF_binding_site	-
10:77916969-77916969	regulatory_region_variant	MODIFIER	-	-	RegulatoryFeature	ENSMUSR00000311847	TF_binding_site	-
10:13178391-13178391	3_prime_UTR_variant	MODIFIER	Ltv1	ENSMUSG00000019814	Transcript	ENSMUST00000019950	protein_coding	rs29363713
10:13178391-13178391	upstream_gene_variant	MODIFIER	Zc2hc1b	ENSMUSG00000019815	Transcript	ENSMUST00000019954	protein_coding	rs29363713
10:13178391-13178391	downstream_gene_variant	MODIFIER	Ltv1	ENSMUSG00000019814	Transcript	ENSMUST00000219824	processed_transcript	rs29363713
10:13178391-13178391	upstream_gene_variant	MODIFIER	Zc2hc1b	ENSMUSG00000019815	Transcript	ENSMUST00000219970	processed_transcript	rs29363713
10:14403831-14403831	3_prime_UTR_variant	MODIFIER	Adgrg6	ENSMUSG00000039116	Transcript	ENSMUST00000041168	protein_coding	rs29323801
10:14403831-14403831	downstream_gene_variant	MODIFIER	Adgrg6	ENSMUSG00000039116	Transcript	ENSMUST00000208429	protein_coding	rs29323801
10:14403831-14403831	regulatory_region_variant	MODIFIER	-	-	RegulatoryFeature	ENSMUSR00000307430	CTCF_binding_site	rs29323801
10:14403851-14403851	3_prime_UTR_variant	MODIFIER	Adgrg6	ENSMUSG00000039116	Transcript	ENSMUST00000041168	protein_coding	rs29338864
10:14403851-14403851	downstream_gene_variant	MODIFIER	Adgrg6	ENSMUSG00000039116	Transcript	ENSMUST00000208429	protein_coding	rs29338864
10:14403851-14403851	regulatory_region_variant	MODIFIER	-	-	RegulatoryFeature	ENSMUSR00000307430	CTCF_binding_site	rs29338864

10:14404135-14404135	3_prime_UTR_variant	MODIFIER	Adgrg6	ENSMUSG00000039116	Transcript	ENSMUST00000041168	protein_coding	rs29366370
10:14404135-14404135	downstream_gene_variant	MODIFIER	Adgrg6	ENSMUSG00000039116	Transcript	ENSMUST00000208429	protein_coding	rs29366370
10:14404135-14404135	regulatory_region_variant	MODIFIER	-	-	RegulatoryFeature	ENSMUSR00000307430	CTCF_binding_site	rs29366370
10:14404135-14404135	regulatory_region_variant	MODIFIER	-	-	RegulatoryFeature	ENSMUSR00000497860	TF_binding_site	rs29366370
10:14404145-14404145	3_prime_UTR_variant	MODIFIER	Adgrg6	ENSMUSG00000039116	Transcript	ENSMUST00000041168	protein_coding	rs29363372
10:14404145-14404145	downstream_gene_variant	MODIFIER	Adgrg6	ENSMUSG00000039116	Transcript	ENSMUST00000208429	protein_coding	rs29363372
10:14404145-14404145	regulatory_region_variant	MODIFIER	-	-	RegulatoryFeature	ENSMUSR00000307430	CTCF_binding_site	rs29363372
10:14404145-14404145	regulatory_region_variant	MODIFIER	-	-	RegulatoryFeature	ENSMUSR00000497860	TF_binding_site	rs29363372
10:14404569-14404569	3_prime_UTR_variant	MODIFIER	Adgrg6	ENSMUSG00000039116	Transcript	ENSMUST00000041168	protein_coding	rs29312342
10:14404569-14404569	downstream_gene_variant	MODIFIER	Adgrg6	ENSMUSG00000039116	Transcript	ENSMUST00000208429	protein_coding	rs29312342
10:14404569-14404569	regulatory_region_variant	MODIFIER	-	-	RegulatoryFeature	ENSMUSR00000307430	CTCF_binding_site	rs29312342
10:14404863-14404863	3_prime_UTR_variant	MODIFIER	Adgrg6	ENSMUSG00000039116	Transcript	ENSMUST00000041168	protein_coding	rs29358617
10:14404863-14404863	downstream_gene_variant	MODIFIER	Adgrg6	ENSMUSG00000039116	Transcript	ENSMUST00000208429	protein_coding	rs29358617
10:18019258-18019258	intron_variant	MODIFIER	Abracl	ENSMUSG00000078453	Transcript	ENSMUST00000020002	protein_coding	rs33598044
10:18019258-18019258	intron_variant	MODIFIER	Abracl	ENSMUSG00000078453	Transcript	ENSMUST00000218994	protein_coding	rs33598044
10:18019258-18019258	intron_variant	MODIFIER	Abracl	ENSMUSG00000078453	Transcript	ENSMUST00000220110	protein_coding	rs33598044
10:18019258-18019258	intron_variant	MODIFIER	Abracl	ENSMUSG00000078453	Transcript	ENSMUST00000220433	protein_coding	rs33598044
11:101578871-101578871	upstream_gene_variant	MODIFIER	Tmem106a	ENSMUSG00000034947	Transcript	ENSMUST00000039581	protein_coding	-
11:101578871-101578871	intron_variant	MODIFIER	Nbr1	ENSMUSG00000017119	Transcript	ENSMUST00000071537	protein_coding	-
11:101578871-101578871	upstream_gene_variant	MODIFIER	Tmem106a	ENSMUSG00000034947	Transcript	ENSMUST00000100403	protein_coding	-
11:101578871-101578871	intron_variant	MODIFIER	Nbr1	ENSMUSG00000017119	Transcript	ENSMUST00000103098	protein_coding	-
11:101578871-101578871	intron_variant	MODIFIER	Nbr1	ENSMUSG00000017119	Transcript	ENSMUST00000103099	protein_coding	-
11:101578871-101578871	upstream_gene_variant	MODIFIER	Tmem106a	ENSMUSG00000034947	Transcript	ENSMUST00000107194	protein_coding	-
11:101578871-101578871	intron_variant	MODIFIER	Nbr1	ENSMUSG00000017119	Transcript	ENSMUST00000107208	protein_coding	-
11:101578871-101578871	intron_variant	MODIFIER	Nbr1	ENSMUSG00000017119	Transcript	ENSMUST00000107212	protein_coding	-
11:101578871-101578871	intron_variant	MODIFIER	Nbr1	ENSMUSG00000017119	Transcript	ENSMUST00000107213	protein_coding	-
11:101578871-101578871	intron_variant	MODIFIER	Nbr1	ENSMUSG00000017119	Transcript	ENSMUST00000107218	protein_coding	-
11:101578871-101578871	intron_variant,NMD_transcript_v	MODIFIER	Nbr1	ENSMUSG00000017119	Transcript	ENSMUST00000123558	nonsense_mediated_decay	-
11:101578871-101578871	upstream_gene_variant	MODIFIER	Tmem106a	ENSMUSG00000034947	Transcript	ENSMUST00000128614	protein_coding	-
11:101578871-101578871	intron_variant,non_coding_trans	MODIFIER	Nbr1	ENSMUSG00000017119	Transcript	ENSMUST00000141170	retained_intron	-
11:101578871-101578871	upstream_gene_variant	MODIFIER	Tmem106a	ENSMUSG00000034947	Transcript	ENSMUST00000143045	processed_transcript	-
11:101578871-101578871	downstream_gene_variant	MODIFIER	Nbr1	ENSMUSG00000017119	Transcript	ENSMUST00000148805	retained_intron	-
11:101578871-101578871	intron_variant	MODIFIER	Nbr1	ENSMUSG00000017119	Transcript	ENSMUST00000149019	protein_coding	-
11:101578871-101578871	downstream_gene_variant	MODIFIER	Nbr1	ENSMUSG00000017119	Transcript	ENSMUST00000172744	retained_intron	-
11:116123864-116123864	downstream_gene_variant	MODIFIER	Trim65	ENSMUSG00000054517	Transcript	ENSMUST00000067632	protein_coding	-
11:116123864-116123864	3_prime_UTR_variant	MODIFIER	Trim65	ENSMUSG00000054517	Transcript	ENSMUST00000106440	protein_coding	-
11:116123864-116123864	intron_variant,non_coding_trans	MODIFIER	Trim47	ENSMUSG00000020773	Transcript	ENSMUST00000133039	processed_transcript	-
11:116123864-116123864	downstream_gene_variant	MODIFIER	Trim65	ENSMUSG00000054517	Transcript	ENSMUST00000154061	processed_transcript	-
11:17944548-17944548	intron_variant	MODIFIER	Etaa1	ENSMUSG00000016984	Transcript	ENSMUST00000076661	protein_coding	rs237839084
11:3920136-3920136	intron_variant	MODIFIER	Tcn2	ENSMUSG00000020432	Transcript	ENSMUST00000020710	protein_coding	-
11:3920136-3920136	intron_variant	MODIFIER	Tcn2	ENSMUSG00000020432	Transcript	ENSMUST00000109988	protein_coding	-
11:3920136-3920136	intron_variant	MODIFIER	Tcn2	ENSMUSG00000020432	Transcript	ENSMUST00000109989	protein_coding	-
11:3920136-3920136	intron_variant	MODIFIER	Tcn2	ENSMUSG00000020432	Transcript	ENSMUST00000109990	protein_coding	-
11:3920136-3920136	intron_variant	MODIFIER	Tcn2	ENSMUSG00000020432	Transcript	ENSMUST00000109991	protein_coding	-
11:3920136-3920136	intron_variant	MODIFIER	Tcn2	ENSMUSG00000020432	Transcript	ENSMUST00000109992	protein_coding	-
11:3920136-3920136	intron_variant	MODIFIER	Tcn2	ENSMUSG00000020432	Transcript	ENSMUST00000109993	protein_coding	-
11:53399900-53399900	frameshift_variant	HIGH	Aff4	ENSMUSG00000049470	Transcript	ENSMUST00000060945	protein_coding	-
11:53399900-53399900	downstream_gene_variant	MODIFIER	Aff4	ENSMUSG00000049470	Transcript	ENSMUST00000130152	processed_transcript	-
11:53399900-53399900	downstream_gene_variant	MODIFIER	Aff4	ENSMUSG00000049470	Transcript	ENSMUST00000152616	protein_coding	-
11:53399900-53399900	upstream_gene_variant	MODIFIER	Aff4	ENSMUSG00000049470	Transcript	ENSMUST00000154499	retained_intron	-
11:79944351-79944351	intron_variant	MODIFIER	Utp6	ENSMUSG00000035575	Transcript	ENSMUST00000043152	protein_coding	-
11:79944351-79944351	intron_variant	MODIFIER	Utp6	ENSMUSG00000035575	Transcript	ENSMUST00000108241	protein_coding	-
11:79944351-79944351	upstream_gene_variant	MODIFIER	Utp6	ENSMUSG00000035575	Transcript	ENSMUST00000131921	retained_intron	-
12:113879380-113879380	synonymous_variant	LOW	Ighv2-9	ENSMUSG00000096638	Transcript	ENSMUST00000103451	IG_V_gene	-
12:113879380-113879380	upstream_gene_variant	MODIFIER	Ighv5-18	ENSMUSG00000103893	Transcript	ENSMUST00000193834	IG_V_pseudogene	-
12:113879380-113879380	upstream_gene_variant	MODIFIER	Ighv2-8	ENSMUSG00000102878	Transcript	ENSMUST00000193935	IG_V_pseudogene	-
12:113879380-113879380	regulatory_region_variant	MODIFIER	-	-	RegulatoryFeature	ENSMUSR00000335516	CTCF_binding_site	-
12:113896566-113896566	missense_variant	MODERATE	Ighv7-1	ENSMUSG00000076665	Transcript	ENSMUST00000103474	IG_V_gene	-
12:113896566-113896566	regulatory_region_variant	MODIFIER	-	-	RegulatoryFeature	ENSMUSR00000335518	CTCF_binding_site	-
12:115848145-115848145	synonymous_variant	LOW	Ighv1-76	ENSMUSG00000093896	Transcript	ENSMUST00000169155	IG_V_gene	-
12:115848145-115848145	synonymous_variant	LOW	Ighv1-76	ENSMUSG00000093896	Transcript	ENSMUST00000197537	IG_V_gene	-
12:115848145-115848145	upstream_gene_variant	MODIFIER	Ighv8-15	ENSMUSG00000104542	Transcript	ENSMUST00000197770	IG_V_pseudogene	-
12:26326424-26326424	intron_variant	MODIFIER	Rnf144a	ENSMUSG00000020642	Transcript	ENSMUST00000020971	protein_coding	rs47376046
12:26326424-26326424	intron_variant	MODIFIER	Rnf144a	ENSMUSG00000020642	Transcript	ENSMUST00000062149	protein_coding	rs47376046
12:26326424-26326424	intron_variant	MODIFIER	Rnf144a	ENSMUSG00000020642	Transcript	ENSMUST00000222082	protein_coding	rs47376046
12:26471162-26471162	intron_variant	MODIFIER	Cmpk2	ENSMUSG00000020638	Transcript	ENSMUST00000020969	protein_coding	rs48163592
12:26471162-26471162	non_coding_transcript_exon_va	MODIFIER	Cmpk2	ENSMUSG00000020638	Transcript	ENSMUST00000220466	processed_transcript	rs48163592
12:26471162-26471162	upstream_gene_variant	MODIFIER	Cmpk2	ENSMUSG00000020638	Transcript	ENSMUST00000221060	retained_intron	rs48163592
12:26471162-26471162	non_coding_transcript_exon_va	MODIFIER	Cmpk2	ENSMUSG00000020638	Transcript	ENSMUST00000221826	retained_intron	rs48163592

12:26471162-26471162	regulatory_region_variant	MODIFIER	-	-	RegulatoryFeature	ENSMUSR00000057227	promoter	rs48163592
12:28634415-28634415	intron_variant	MODIFIER	Rps7	ENSMUSG00000061477	Transcript	ENSMUST00000074267	protein_coding	rs50750970
12:28634415-28634415	intron_variant,non_coding_trans	MODIFIER	Rps7	ENSMUSG00000061477	Transcript	ENSMUST00000220860	retained_intron	rs50750970
12:28634415-28634415	intron_variant	MODIFIER	Rps7	ENSMUSG00000061477	Transcript	ENSMUST00000221871	protein_coding	rs50750970
12:28634415-28634415	downstream_gene_variant	MODIFIER	Rps7	ENSMUSG00000061477	Transcript	ENSMUST00000222228	retained_intron	rs50750970
12:28634415-28634415	upstream_gene_variant	MODIFIER	Rps7	ENSMUSG00000061477	Transcript	ENSMUST00000222808	retained_intron	rs50750970
12:28634415-28634415	upstream_gene_variant	MODIFIER	Rps7	ENSMUSG00000061477	Transcript	ENSMUST00000223153	retained_intron	rs50750970
12:28634415-28634415	upstream_gene_variant	MODIFIER	Rps7	ENSMUSG00000061477	Transcript	ENSMUST00000223318	processed_transcript	rs50750970
12:28634415-28634415	regulatory_region_variant	MODIFIER	-	-	RegulatoryFeature	ENSMUSR00000057345	promoter	rs50750970
12:28658700-28658700	intron_variant	MODIFIER	Rnaseh1	ENSMUSG00000020630	Transcript	ENSMUST00000020959	protein_coding	rs48968014
12:28658700-28658700	intron_variant,non_coding_trans	MODIFIER	Rnaseh1	ENSMUSG00000020630	Transcript	ENSMUST00000221496	retained_intron	rs48968014
12:28658700-28658700	downstream_gene_variant	MODIFIER	Rnaseh1	ENSMUSG00000020630	Transcript	ENSMUST00000222767	retained_intron	rs48968014
12:28658700-28658700	intron_variant,NMD_transcript_v	MODIFIER	Rnaseh1	ENSMUSG00000020630	Transcript	ENSMUST00000223322	nonsense_mediated_decay	rs48968014
12:28856099-28856099	intron_variant,NMD_transcript_v	MODIFIER	Eipr1	ENSMUSG00000036613	Transcript	ENSMUST00000035657	nonsense_mediated_decay	rs47296512
12:28856099-28856099	intron_variant	MODIFIER	Eipr1	ENSMUSG00000036613	Transcript	ENSMUST00000221555	protein_coding	rs47296512
12:28856099-28856099	upstream_gene_variant	MODIFIER	Eipr1	ENSMUSG00000036613	Transcript	ENSMUST00000221574	processed_transcript	rs47296512
12:28856099-28856099	intron_variant	MODIFIER	Eipr1	ENSMUSG00000036613	Transcript	ENSMUST00000221877	protein_coding	rs47296512
12:32033121-32033121	intron_variant	MODIFIER	Prkar2b	ENSMUSG00000002997	Transcript	ENSMUST00000003079	protein_coding	rs29221895
12:32033121-32033121	intron_variant	MODIFIER	Prkar2b	ENSMUSG00000002997	Transcript	ENSMUST00000036497	protein_coding	rs29221895
12:32033121-32033121	intron_variant	MODIFIER	Prkar2b	ENSMUSG00000002997	Transcript	ENSMUST00000146865	protein_coding	rs29221895
12:32847944-32847944	intron_variant	MODIFIER	Nampt	ENSMUSG00000020572	Transcript	ENSMUST00000020886	protein_coding	rs50780349
12:33365082-33365082	intron_variant	MODIFIER	Atxn7l1	ENSMUSG00000020564	Transcript	ENSMUST00000090597	protein_coding	rs29180657
12:33365082-33365082	intron_variant	MODIFIER	Atxn7l1	ENSMUSG00000020564	Transcript	ENSMUST00000125192	protein_coding	rs29180657
12:33365082-33365082	intron_variant,non_coding_trans	MODIFIER	Atxn7l1	ENSMUSG00000020564	Transcript	ENSMUST00000126275	retained_intron	rs29180657
12:33365082-33365082	intron_variant,non_coding_trans	MODIFIER	Atxn7l1os2	ENSMUSG00000085664	Transcript	ENSMUST00000138617	antisense	rs29180657
12:33365082-33365082	upstream_gene_variant	MODIFIER	Atxn7l1	ENSMUSG00000020564	Transcript	ENSMUST00000142184	processed_transcript	rs29180657
12:33365082-33365082	intron_variant	MODIFIER	Atxn7l1	ENSMUSG00000020564	Transcript	ENSMUST00000146040	protein_coding	rs29180657
12:33365082-33365082	intron_variant	MODIFIER	Atxn7l1	ENSMUSG00000020564	Transcript	ENSMUST00000154742	protein_coding	rs29180657
12:34047933-34047934	downstream_gene_variant	MODIFIER	Hdac9	ENSMUSG00000004698	Transcript	ENSMUST00000209750	protein_coding	rs1131802444
12:34047933-34047934	3_prime_UTR_variant	MODIFIER	Hdac9	ENSMUSG00000004698	Transcript	ENSMUST00000209902	protein_coding	rs1131802444
12:34047933-34047934	downstream_gene_variant	MODIFIER	Hdac9	ENSMUSG00000004698	Transcript	ENSMUST00000209990	protein_coding	rs1131802444
12:34048381-34048382	downstream_gene_variant	MODIFIER	Hdac9	ENSMUSG00000004698	Transcript	ENSMUST00000209750	protein_coding	-
12:34048381-34048382	3_prime_UTR_variant	MODIFIER	Hdac9	ENSMUSG00000004698	Transcript	ENSMUST00000209902	protein_coding	-
12:34048381-34048382	downstream_gene_variant	MODIFIER	Hdac9	ENSMUSG00000004698	Transcript	ENSMUST00000209990	protein_coding	-
12:35139784-35139784	intron_variant	MODIFIER	Snx13	ENSMUSG00000020590	Transcript	ENSMUST00000048519	protein_coding	rs46642947
12:35139784-35139784	intron_variant,non_coding_trans	MODIFIER	Snx13	ENSMUSG00000020590	Transcript	ENSMUST00000221876	processed_transcript	rs46642947
12:35139784-35139784	upstream_gene_variant	MODIFIER	Gm48137	ENSMUSG00000113571	Transcript	ENSMUST00000223056	TEC	rs46642947
12:35499737-35499737	3_prime_UTR_variant	MODIFIER	Ahr	ENSMUSG00000019256	Transcript	ENSMUST00000116436	protein_coding	rs3021586
12:40321187-40321187	intron_variant	MODIFIER	Zfp277	ENSMUSG00000055917	Transcript	ENSMUST00000069637	protein_coding	rs29212682
12:40321187-40321187	intron_variant	MODIFIER	Zfp277	ENSMUSG00000055917	Transcript	ENSMUST00000069692	protein_coding	rs29212682
12:40321187-40321187	non_coding_transcript_exon_va	MODIFIER	Zfp277	ENSMUSG00000055917	Transcript	ENSMUST00000221604	retained_intron	rs29212682
12:40321187-40321187	downstream_gene_variant	MODIFIER	Zfp277	ENSMUSG00000055917	Transcript	ENSMUST00000222394	retained_intron	rs29212682
12:40321264-40321264	intron_variant	MODIFIER	Zfp277	ENSMUSG00000055917	Transcript	ENSMUST00000069637	protein_coding	rs50250906
12:40321264-40321264	intron_variant	MODIFIER	Zfp277	ENSMUSG00000055917	Transcript	ENSMUST00000069692	protein_coding	rs50250906
12:40321264-40321264	non_coding_transcript_exon_va	MODIFIER	Zfp277	ENSMUSG00000055917	Transcript	ENSMUST00000221604	retained_intron	rs50250906
12:40321264-40321264	downstream_gene_variant	MODIFIER	Zfp277	ENSMUSG00000055917	Transcript	ENSMUST00000222394	retained_intron	rs50250906
12:40321604-40321604	intron_variant	MODIFIER	Zfp277	ENSMUSG00000055917	Transcript	ENSMUST00000069637	protein_coding	rs228099868
12:40321604-40321604	intron_variant	MODIFIER	Zfp277	ENSMUSG00000055917	Transcript	ENSMUST00000069692	protein_coding	rs228099868
12:40321604-40321604	non_coding_transcript_exon_va	MODIFIER	Zfp277	ENSMUSG00000055917	Transcript	ENSMUST00000221604	retained_intron	rs228099868
12:40321604-40321604	downstream_gene_variant	MODIFIER	Zfp277	ENSMUSG00000055917	Transcript	ENSMUST00000222394	retained_intron	rs228099868
12:40321609-40321609	intron_variant	MODIFIER	Zfp277	ENSMUSG00000055917	Transcript	ENSMUST00000069637	protein_coding	rs248350135
12:40321609-40321609	intron_variant	MODIFIER	Zfp277	ENSMUSG00000055917	Transcript	ENSMUST00000069692	protein_coding	rs248350135
12:40321609-40321609	non_coding_transcript_exon_va	MODIFIER	Zfp277	ENSMUSG00000055917	Transcript	ENSMUST00000221604	retained_intron	rs248350135
12:40321609-40321609	downstream_gene_variant	MODIFIER	Zfp277	ENSMUSG00000055917	Transcript	ENSMUST00000222394	retained_intron	rs248350135
12:40321801-40321801	intron_variant	MODIFIER	Zfp277	ENSMUSG00000055917	Transcript	ENSMUST00000069637	protein_coding	rs254505481
12:40321801-40321801	intron_variant	MODIFIER	Zfp277	ENSMUSG00000055917	Transcript	ENSMUST00000069692	protein_coding	rs254505481
12:40321801-40321801	non_coding_transcript_exon_va	MODIFIER	Zfp277	ENSMUSG00000055917	Transcript	ENSMUST00000221604	retained_intron	rs254505481
12:40321801-40321801	downstream_gene_variant	MODIFIER	Zfp277	ENSMUSG00000055917	Transcript	ENSMUST00000222394	retained_intron	rs254505481
12:40322472-40322472	intron_variant	MODIFIER	Zfp277	ENSMUSG00000055917	Transcript	ENSMUST00000069637	protein_coding	rs29171582
12:40322472-40322472	intron_variant	MODIFIER	Zfp277	ENSMUSG00000055917	Transcript	ENSMUST00000069692	protein_coding	rs29171582
12:40322472-40322472	non_coding_transcript_exon_va	MODIFIER	Zfp277	ENSMUSG00000055917	Transcript	ENSMUST00000221604	retained_intron	rs29171582
12:40322472-40322472	downstream_gene_variant	MODIFIER	Zfp277	ENSMUSG00000055917	Transcript	ENSMUST00000222394	retained_intron	rs29171582
12:40672844-40672844	synonymous_variant	LOW	Dock4	ENSMUSG00000035954	Transcript	ENSMUST00000037488	protein_coding	rs47722120
12:40672844-40672844	synonymous_variant	LOW	Dock4	ENSMUSG00000035954	Transcript	ENSMUST00000220912	protein_coding	rs47722120
12:40672844-40672844	non_coding_transcript_exon_va	MODIFIER	Dock4	ENSMUSG00000035954	Transcript	ENSMUST00000221758	retained_intron	rs47722120
12:40672844-40672844	non_coding_transcript_exon_va	MODIFIER	Dock4	ENSMUSG00000035954	Transcript	ENSMUST00000222287	retained_intron	rs47722120
12:4239002-4239003	upstream_gene_variant	MODIFIER	Cenpo	ENSMUSG00000020652	Transcript	ENSMUST00000140975	protein_coding	rs387695635
12:4239002-4239003	3_prime_UTR_variant	MODIFIER	Ptrhd1	ENSMUSG00000096199	Transcript	ENSMUST00000179139	protein_coding	rs387695635
12:4239002-4239003	downstream_gene_variant	MODIFIER	Gm29696	ENSMUSG00000112829	Transcript	ENSMUST00000219209	processed_pseudogene	rs387695635

12:4239002-4239003	upstream_gene_variant	MODIFIER	4921501I09Rik	ENSMUSG00000111916	Transcript	ENSMUST00000220337	antisense	rs387695635
12:51364684-51364684	intron_variant	MODIFIER	G2e3	ENSMUSG00000035293	Transcript	ENSMUST00000054308	protein_coding	rs51985107
12:51364684-51364684	intron_variant	MODIFIER	G2e3	ENSMUSG00000035293	Transcript	ENSMUST00000119211	protein_coding	rs51985107
12:51364684-51364684	intron_variant	MODIFIER	G2e3	ENSMUSG00000035293	Transcript	ENSMUST00000121521	protein_coding	rs51985107
12:51364684-51364684	downstream_gene_variant	MODIFIER	G2e3	ENSMUSG00000035293	Transcript	ENSMUST00000144767	retained_intron	rs51985107
12:51365873-51365873	intron_variant	MODIFIER	G2e3	ENSMUSG00000035293	Transcript	ENSMUST00000054308	protein_coding	rs262181154
12:51365873-51365873	intron_variant	MODIFIER	G2e3	ENSMUSG00000035293	Transcript	ENSMUST00000119211	protein_coding	rs262181154
12:51365873-51365873	intron_variant	MODIFIER	G2e3	ENSMUSG00000035293	Transcript	ENSMUST00000121521	protein_coding	rs262181154
12:51365873-51365873	downstream_gene_variant	MODIFIER	G2e3	ENSMUSG00000035293	Transcript	ENSMUST00000144767	retained_intron	rs262181154
12:51365873-51365873	upstream_gene_variant	MODIFIER	G2e3	ENSMUSG00000035293	Transcript	ENSMUST00000152236	retained_intron	rs262181154
12:51373038-51373041	upstream_gene_variant	MODIFIER	Scfd1	ENSMUSG00000020952	Transcript	ENSMUST00000021335	protein_coding	rs221509553
12:51373038-51373041	3_prime_UTR_variant	MODIFIER	G2e3	ENSMUSG00000035293	Transcript	ENSMUST00000054308	protein_coding	rs221509553
12:51373038-51373041	3_prime_UTR_variant	MODIFIER	G2e3	ENSMUSG00000035293	Transcript	ENSMUST00000119211	protein_coding	rs221509553
12:51373038-51373041	3_prime_UTR_variant	MODIFIER	G2e3	ENSMUSG00000035293	Transcript	ENSMUST00000121521	protein_coding	rs221509553
12:51373038-51373041	non_coding_transcript_exon_vai	MODIFIER	G2e3	ENSMUSG00000035293	Transcript	ENSMUST00000152236	retained_intron	rs221509553
12:51373038-51373041	upstream_gene_variant	MODIFIER	Scfd1	ENSMUSG00000020952	Transcript	ENSMUST00000218131	retained_intron	rs221509553
12:51373038-51373041	upstream_gene_variant	MODIFIER	Scfd1	ENSMUSG00000020952	Transcript	ENSMUST00000219264	retained_intron	rs221509553
12:51373038-51373041	upstream_gene_variant	MODIFIER	Scfd1	ENSMUSG00000020952	Transcript	ENSMUST00000219434	protein_coding	rs221509553
12:51373038-51373041	upstream_gene_variant	MODIFIER	Scfd1	ENSMUSG00000020952	Transcript	ENSMUST00000219799	retained_intron	rs221509553
12:51444090-51444090	intron_variant	MODIFIER	Scfd1	ENSMUSG00000020952	Transcript	ENSMUST00000021335	protein_coding	rs29185499
12:51444090-51444090	intron_variant,NMD_transcript_v	MODIFIER	Scfd1	ENSMUSG00000020952	Transcript	ENSMUST00000218138	nonsense_mediated_decay	rs29185499
12:51444090-51444090	downstream_gene_variant	MODIFIER	Scfd1	ENSMUSG00000020952	Transcript	ENSMUST00000219686	retained_intron	rs29185499
12:51593770-51593770	missense_variant	MODERATE	Coch	ENSMUSG00000020953	Transcript	ENSMUST00000085412	protein_coding	rs51488430
12:51593770-51593770	missense_variant	MODERATE	Coch	ENSMUSG00000020953	Transcript	ENSMUST00000164782	protein_coding	rs51488430
12:51593770-51593770	non_coding_transcript_exon_vai	MODIFIER	Coch	ENSMUSG00000020953	Transcript	ENSMUST00000218382	retained_intron	rs51488430
12:51593770-51593770	non_coding_transcript_exon_vai	MODIFIER	Coch	ENSMUSG00000020953	Transcript	ENSMUST00000220173	retained_intron	rs51488430
12:51593770-51593770	regulatory_region_variant	MODIFIER	-	-	RegulatoryFeature	ENSMUSR00000533198	promoter	rs51488430
12:51595483-51595483	synonymous_variant	LOW	Coch	ENSMUSG00000020953	Transcript	ENSMUST00000085412	protein_coding	rs29206368
12:51595483-51595483	synonymous_variant	LOW	Coch	ENSMUSG00000020953	Transcript	ENSMUST00000164782	protein_coding	rs29206368
12:51595483-51595483	downstream_gene_variant	MODIFIER	Coch	ENSMUSG00000020953	Transcript	ENSMUST00000218382	retained_intron	rs29206368
12:51595483-51595483	downstream_gene_variant	MODIFIER	Coch	ENSMUSG00000020953	Transcript	ENSMUST00000220173	retained_intron	rs29206368
12:51598285-51598285	synonymous_variant	LOW	Coch	ENSMUSG00000020953	Transcript	ENSMUST00000085412	protein_coding	rs108185983
12:51598285-51598285	synonymous_variant	LOW	Coch	ENSMUSG00000020953	Transcript	ENSMUST00000164782	protein_coding	rs108185983
12:51598285-51598285	downstream_gene_variant	MODIFIER	Coch	ENSMUSG00000020953	Transcript	ENSMUST00000218382	retained_intron	rs108185983
12:51598285-51598285	downstream_gene_variant	MODIFIER	Coch	ENSMUSG00000020953	Transcript	ENSMUST00000220173	retained_intron	rs108185983
12:51603510-51603510	synonymous_variant	LOW	Coch	ENSMUSG00000020953	Transcript	ENSMUST00000085412	protein_coding	rs46683073
12:51603510-51603510	synonymous_variant	LOW	Coch	ENSMUSG00000020953	Transcript	ENSMUST00000164782	protein_coding	rs46683073
12:51605034-51605034	downstream_gene_variant	MODIFIER	Strn3	ENSMUSG00000020954	Transcript	ENSMUST00000013130	protein_coding	rs6305027
12:51605034-51605034	3_prime_UTR_variant	MODIFIER	Coch	ENSMUSG00000020953	Transcript	ENSMUST00000085412	protein_coding	rs6305027
12:51605034-51605034	3_prime_UTR_variant	MODIFIER	Coch	ENSMUSG00000020953	Transcript	ENSMUST00000164782	protein_coding	rs6305027
12:51605034-51605034	downstream_gene_variant	MODIFIER	Strn3	ENSMUSG00000020954	Transcript	ENSMUST00000169503	protein_coding	rs6305027
12:51605135-51605135	downstream_gene_variant	MODIFIER	Strn3	ENSMUSG00000020954	Transcript	ENSMUST00000013130	protein_coding	rs6317623
12:51605135-51605135	3_prime_UTR_variant	MODIFIER	Coch	ENSMUSG00000020953	Transcript	ENSMUST00000085412	protein_coding	rs6317623
12:51605135-51605135	3_prime_UTR_variant	MODIFIER	Coch	ENSMUSG00000020953	Transcript	ENSMUST00000164782	protein_coding	rs6317623
12:51605135-51605135	downstream_gene_variant	MODIFIER	Strn3	ENSMUSG00000020954	Transcript	ENSMUST00000169503	protein_coding	rs6317623
12:51605406-51605406	downstream_gene_variant	MODIFIER	Strn3	ENSMUSG00000020954	Transcript	ENSMUST00000013130	protein_coding	rs48556992
12:51605406-51605406	3_prime_UTR_variant	MODIFIER	Coch	ENSMUSG00000020953	Transcript	ENSMUST00000085412	protein_coding	rs48556992
12:51605406-51605406	3_prime_UTR_variant	MODIFIER	Coch	ENSMUSG00000020953	Transcript	ENSMUST00000164782	protein_coding	rs48556992
12:51605406-51605406	downstream_gene_variant	MODIFIER	Strn3	ENSMUSG00000020954	Transcript	ENSMUST00000169503	protein_coding	rs48556992
12:51605503-51605503	downstream_gene_variant	MODIFIER	Strn3	ENSMUSG00000020954	Transcript	ENSMUST00000013130	protein_coding	rs49914855
12:51605503-51605503	3_prime_UTR_variant	MODIFIER	Coch	ENSMUSG00000020953	Transcript	ENSMUST00000085412	protein_coding	rs49914855
12:51605503-51605503	3_prime_UTR_variant	MODIFIER	Coch	ENSMUSG00000020953	Transcript	ENSMUST00000164782	protein_coding	rs49914855
12:51605503-51605503	downstream_gene_variant	MODIFIER	Strn3	ENSMUSG00000020954	Transcript	ENSMUST00000169503	protein_coding	rs49914855
12:51605691-51605691	downstream_gene_variant	MODIFIER	Strn3	ENSMUSG00000020954	Transcript	ENSMUST00000013130	protein_coding	rs29487849
12:51605691-51605691	3_prime_UTR_variant	MODIFIER	Coch	ENSMUSG00000020953	Transcript	ENSMUST00000085412	protein_coding	rs29487849
12:51605691-51605691	3_prime_UTR_variant	MODIFIER	Coch	ENSMUSG00000020953	Transcript	ENSMUST00000164782	protein_coding	rs29487849
12:51605691-51605691	downstream_gene_variant	MODIFIER	Strn3	ENSMUSG00000020954	Transcript	ENSMUST00000169503	protein_coding	rs29487849
12:51833358-51833358	upstream_gene_variant	MODIFIER	Hectd1	ENSMUSG00000035247	Transcript	ENSMUST00000042052	protein_coding	rs223086520
12:51833358-51833358	upstream_gene_variant	MODIFIER	Hectd1	ENSMUSG00000035247	Transcript	ENSMUST00000179265	protein_coding	rs223086520
12:51833358-51833358	upstream_gene_variant	MODIFIER	Hectd1	ENSMUSG00000035247	Transcript	ENSMUST00000217804	retained_intron	rs223086520
12:51885286-51885286	intron_variant	MODIFIER	Heatr5a	ENSMUSG00000035181	Transcript	ENSMUST00000040583	protein_coding	rs29149242
12:51885286-51885286	upstream_gene_variant	MODIFIER	Gm26517	ENSMUSG00000097159	Transcript	ENSMUST00000181279	lincRNA	rs29149242
12:51885286-51885286	downstream_gene_variant	MODIFIER	Heatr5a	ENSMUSG00000035181	Transcript	ENSMUST00000218847	retained_intron	rs29149242
12:51885286-51885286	intron_variant,non_coding_trans	MODIFIER	Heatr5a	ENSMUSG00000035181	Transcript	ENSMUST00000220369	retained_intron	rs29149242
12:51886298-51886298	intron_variant	MODIFIER	Heatr5a	ENSMUSG00000035181	Transcript	ENSMUST00000040583	protein_coding	rs3710688
12:51886298-51886298	upstream_gene_variant	MODIFIER	Gm26517	ENSMUSG00000097159	Transcript	ENSMUST00000181279	lincRNA	rs3710688
12:51886298-51886298	downstream_gene_variant	MODIFIER	Heatr5a	ENSMUSG00000035181	Transcript	ENSMUST00000218847	retained_intron	rs3710688
12:51886298-51886298	intron_variant,non_coding_trans	MODIFIER	Heatr5a	ENSMUSG00000035181	Transcript	ENSMUST00000220369	retained_intron	rs3710688
12:51886298-51886298	regulatory_region_variant	MODIFIER	-	-	RegulatoryFeature	ENSMUSR00000533256	promoter_flanking_region	rs3710688

12:51886682-51886682	intron_variant	MODIFIER	Heatr5a	ENSMUSG00000035181	Transcript	ENSMUST00000040583	protein_coding	rs248084547
12:51886682-51886682	upstream_gene_variant	MODIFIER	Gm26517	ENSMUSG00000097159	Transcript	ENSMUST00000181279	lincRNA	rs248084547
12:51886682-51886682	downstream_gene_variant	MODIFIER	Heatr5a	ENSMUSG00000035181	Transcript	ENSMUST00000218847	retained_intron	rs248084547
12:51886682-51886682	intron_variant,non_coding_trans	MODIFIER	Heatr5a	ENSMUSG00000035181	Transcript	ENSMUST00000220369	retained_intron	rs248084547
12:51886682-51886682	regulatory_region_variant	MODIFIER	-	-	RegulatoryFeature	ENSMUSR00000533256	promoter_flanking_region	rs248084547
12:51889316-51889316	intron_variant	MODIFIER	Heatr5a	ENSMUSG00000035181	Transcript	ENSMUST00000040583	protein_coding	rs29129796
12:51889316-51889316	non_coding_transcript_exon_va	MODIFIER	Gm26517	ENSMUSG00000097159	Transcript	ENSMUST00000181279	lincRNA	rs29129796
12:51889316-51889316	upstream_gene_variant	MODIFIER	Heatr5a	ENSMUSG00000035181	Transcript	ENSMUST00000218847	retained_intron	rs29129796
12:51889316-51889316	intron_variant,non_coding_trans	MODIFIER	Heatr5a	ENSMUSG00000035181	Transcript	ENSMUST00000220369	retained_intron	rs29129796
12:51959102-51959102	intron_variant	MODIFIER	Heatr5a	ENSMUSG00000035181	Transcript	ENSMUST00000040583	protein_coding	rs46053728
12:51959102-51959102	intron_variant,non_coding_trans	MODIFIER	Heatr5a	ENSMUSG00000035181	Transcript	ENSMUST00000218254	retained_intron	rs46053728
12:51959303-51959303	intron_variant	MODIFIER	Heatr5a	ENSMUSG00000035181	Transcript	ENSMUST00000040583	protein_coding	rs216885560
12:51959303-51959303	intron_variant,non_coding_trans	MODIFIER	Heatr5a	ENSMUSG00000035181	Transcript	ENSMUST00000218254	retained_intron	rs216885560
12:51959402-51959402	intron_variant	MODIFIER	Heatr5a	ENSMUSG00000035181	Transcript	ENSMUST00000040583	protein_coding	rs50191425
12:51959402-51959402	intron_variant,non_coding_trans	MODIFIER	Heatr5a	ENSMUSG00000035181	Transcript	ENSMUST00000218254	retained_intron	rs50191425
12:53450295-53450295	intron_variant	MODIFIER	Npas3	ENSMUSG00000021010	Transcript	ENSMUST00000101432	protein_coding	rs6270967
12:53450295-53450295	intron_variant	MODIFIER	Npas3	ENSMUSG00000021010	Transcript	ENSMUST00000220986	protein_coding	rs6270967
12:53450295-53450295	intron_variant	MODIFIER	Npas3	ENSMUSG00000021010	Transcript	ENSMUST00000223057	protein_coding	rs6270967
12:53450295-53450295	intron_variant	MODIFIER	Npas3	ENSMUSG00000021010	Transcript	ENSMUST00000223358	protein_coding	rs6270967
12:53501122-53501122	synonymous_variant	LOW	Npas3	ENSMUSG00000021010	Transcript	ENSMUST00000101432	protein_coding	rs49025284
12:53501122-53501122	synonymous_variant	LOW	Npas3	ENSMUSG00000021010	Transcript	ENSMUST00000220986	protein_coding	rs49025284
12:53501122-53501122	synonymous_variant	LOW	Npas3	ENSMUSG00000021010	Transcript	ENSMUST00000223057	protein_coding	rs49025284
12:53501122-53501122	synonymous_variant	LOW	Npas3	ENSMUSG00000021010	Transcript	ENSMUST00000223358	protein_coding	rs49025284
12:54048844-54048844	synonymous_variant	LOW	Npas3	ENSMUSG00000021010	Transcript	ENSMUST00000101432	protein_coding	rs31846266
12:54048844-54048844	synonymous_variant	LOW	Npas3	ENSMUSG00000021010	Transcript	ENSMUST00000223057	protein_coding	rs31846266
12:54048844-54048844	synonymous_variant	LOW	Npas3	ENSMUSG00000021010	Transcript	ENSMUST00000223358	protein_coding	rs31846266
12:54067745-54067745	synonymous_variant	LOW	Npas3	ENSMUSG00000021010	Transcript	ENSMUST00000101432	protein_coding	rs29205467
12:54067745-54067745	intron_variant,non_coding_trans	MODIFIER	1700060O08Rik	ENSMUSG00000099407	Transcript	ENSMUST00000185390	antisense	rs29205467
12:54067745-54067745	synonymous_variant	LOW	Npas3	ENSMUSG00000021010	Transcript	ENSMUST00000223057	protein_coding	rs29205467
12:54067745-54067745	synonymous_variant	LOW	Npas3	ENSMUSG00000021010	Transcript	ENSMUST00000223358	protein_coding	rs29205467
12:54067745-54067745	regulatory_region_variant	MODIFIER	-	-	RegulatoryFeature	ENSMUSR00000059714	promoter_flanking_region	rs29205467
12:54067760-54067760	synonymous_variant	LOW	Npas3	ENSMUSG00000021010	Transcript	ENSMUST00000101432	protein_coding	rs29186598
12:54067760-54067760	intron_variant,non_coding_trans	MODIFIER	1700060O08Rik	ENSMUSG00000099407	Transcript	ENSMUST00000185390	antisense	rs29186598
12:54067760-54067760	synonymous_variant	LOW	Npas3	ENSMUSG00000021010	Transcript	ENSMUST00000223057	protein_coding	rs29186598
12:54067760-54067760	synonymous_variant	LOW	Npas3	ENSMUSG00000021010	Transcript	ENSMUST00000223358	protein_coding	rs29186598
12:54067760-54067760	regulatory_region_variant	MODIFIER	-	-	RegulatoryFeature	ENSMUSR00000059714	promoter_flanking_region	rs29186598
12:54068897-54068897	synonymous_variant	LOW	Npas3	ENSMUSG00000021010	Transcript	ENSMUST00000101432	protein_coding	rs29126191
12:54068897-54068897	intron_variant,non_coding_trans	MODIFIER	1700060O08Rik	ENSMUSG00000099407	Transcript	ENSMUST00000185390	antisense	rs29126191
12:54068897-54068897	synonymous_variant	LOW	Npas3	ENSMUSG00000021010	Transcript	ENSMUST00000223057	protein_coding	rs29126191
12:54068897-54068897	synonymous_variant	LOW	Npas3	ENSMUSG00000021010	Transcript	ENSMUST00000223358	protein_coding	rs29126191
12:54068897-54068897	regulatory_region_variant	MODIFIER	-	-	RegulatoryFeature	ENSMUSR00000059714	promoter_flanking_region	rs29126191
12:54069940-54069940	3_prime_UTR_variant	MODIFIER	Npas3	ENSMUSG00000021010	Transcript	ENSMUST00000101432	protein_coding	rs213093238
12:54069940-54069940	intron_variant,non_coding_trans	MODIFIER	1700060O08Rik	ENSMUSG00000099407	Transcript	ENSMUST00000185390	antisense	rs213093238
12:54069940-54069940	downstream_gene_variant	MODIFIER	Npas3	ENSMUSG00000021010	Transcript	ENSMUST00000223057	protein_coding	rs213093238
12:54069940-54069940	downstream_gene_variant	MODIFIER	Npas3	ENSMUSG00000021010	Transcript	ENSMUST00000223358	protein_coding	rs213093238
12:54070676-54070676	3_prime_UTR_variant	MODIFIER	Npas3	ENSMUSG00000021010	Transcript	ENSMUST00000101432	protein_coding	rs29131037
12:54070676-54070676	intron_variant,non_coding_trans	MODIFIER	1700060O08Rik	ENSMUSG00000099407	Transcript	ENSMUST00000185390	antisense	rs29131037
12:54070676-54070676	downstream_gene_variant	MODIFIER	Npas3	ENSMUSG00000021010	Transcript	ENSMUST00000223057	protein_coding	rs29131037
12:54070676-54070676	downstream_gene_variant	MODIFIER	Npas3	ENSMUSG00000021010	Transcript	ENSMUST00000223358	protein_coding	rs29131037
12:54071422-54071422	3_prime_UTR_variant	MODIFIER	Npas3	ENSMUSG00000021010	Transcript	ENSMUST00000101432	protein_coding	rs223330646
12:54071422-54071422	intron_variant,non_coding_trans	MODIFIER	1700060O08Rik	ENSMUSG00000099407	Transcript	ENSMUST00000185390	antisense	rs223330646
12:54071422-54071422	downstream_gene_variant	MODIFIER	Npas3	ENSMUSG00000021010	Transcript	ENSMUST00000223057	protein_coding	rs223330646
12:54071422-54071422	downstream_gene_variant	MODIFIER	Npas3	ENSMUSG00000021010	Transcript	ENSMUST00000223358	protein_coding	rs223330646
13:90289706-90289706	intergenic_variant	MODIFIER	-	-	-	-	-	-
14:31381794-31381794	intron_variant	MODIFIER	Sh3bp5	ENSMUSG00000021892	Transcript	ENSMUST00000091903	protein_coding	rs230349184
14:31381794-31381794	intron_variant	MODIFIER	Sh3bp5	ENSMUSG00000021892	Transcript	ENSMUST00000100730	protein_coding	rs230349184
14:31381794-31381794	intron_variant,NMD_transcript_v	MODIFIER	Sh3bp5	ENSMUSG00000021892	Transcript	ENSMUST00000140002	nonsense_mediated_decay	rs230349184
14:31381794-31381794	intron_variant,non_coding_trans	MODIFIER	Sh3bp5	ENSMUSG00000021892	Transcript	ENSMUST00000147586	processed_transcript	rs230349184
14:31381816-31381816	intron_variant	MODIFIER	Sh3bp5	ENSMUSG00000021892	Transcript	ENSMUST00000091903	protein_coding	rs222597081
14:31381816-31381816	intron_variant	MODIFIER	Sh3bp5	ENSMUSG00000021892	Transcript	ENSMUST00000100730	protein_coding	rs222597081
14:31381816-31381816	intron_variant,NMD_transcript_v	MODIFIER	Sh3bp5	ENSMUSG00000021892	Transcript	ENSMUST00000140002	nonsense_mediated_decay	rs222597081
14:31381816-31381816	intron_variant,non_coding_trans	MODIFIER	Sh3bp5	ENSMUSG00000021892	Transcript	ENSMUST00000147586	processed_transcript	rs222597081
14:32182861-32182861	intron_variant	MODIFIER	Timm23	ENSMUSG00000013701	Transcript	ENSMUST00000013845	protein_coding	rs31328711
14:32182861-32182861	downstream_gene_variant	MODIFIER	Ncoa4	ENSMUSG00000056234	Transcript	ENSMUST00000111994	protein_coding	rs31328711
14:32182861-32182861	downstream_gene_variant	MODIFIER	Ncoa4	ENSMUSG00000056234	Transcript	ENSMUST00000163336	protein_coding	rs31328711
14:32182861-32182861	intron_variant,NMD_transcript_v	MODIFIER	Timm23	ENSMUSG00000013701	Transcript	ENSMUST00000163379	nonsense_mediated_decay	rs31328711
14:32182861-32182861	intron_variant,non_coding_trans	MODIFIER	Timm23	ENSMUSG00000013701	Transcript	ENSMUST00000168139	processed_transcript	rs31328711
14:32182861-32182861	downstream_gene_variant	MODIFIER	Ncoa4	ENSMUSG00000056234	Transcript	ENSMUST00000168334	nonsense_mediated_decay	rs31328711
14:32182861-32182861	intron_variant	MODIFIER	Timm23	ENSMUSG00000013701	Transcript	ENSMUST00000170331	protein_coding	rs31328711

14:32182861-32182861	intron_variant	MODIFIER	Timm23	ENSMUSG00000013701	Transcript	ENSMUST00000226683	protein_coding	rs31328711
14:32182861-32182861	downstream_gene_variant	MODIFIER	Ncoa4	ENSMUSG00000056234	Transcript	ENSMUST00000227112	processed_transcript	rs31328711
14:32298865-32298865	downstream_gene_variant	MODIFIER	Parg	ENSMUSG00000021911	Transcript	ENSMUST00000022470	protein_coding	rs47613579
14:32298865-32298865	downstream_gene_variant	MODIFIER	Parg	ENSMUSG00000021911	Transcript	ENSMUST00000163350	protein_coding	rs47613579
14:32298865-32298865	upstream_gene_variant	MODIFIER	Gm17210	ENSMUSG00000090459	Transcript	ENSMUST00000169975	antisense	rs47613579
14:32298865-32298865	downstream_gene_variant	MODIFIER	Parg	ENSMUSG00000021911	Transcript	ENSMUST00000170129	nonsense_mediated_decay	rs47613579
14:32298865-32298865	downstream_gene_variant	MODIFIER	Parg	ENSMUSG00000021911	Transcript	ENSMUST00000170840	protein_coding	rs47613579
14:32298865-32298865	downstream_gene_variant	MODIFIER	Parg	ENSMUSG00000021911	Transcript	ENSMUST00000171871	processed_transcript	rs47613579
14:32298865-32298865	downstream_gene_variant	MODIFIER	Gm48914	ENSMUSG00000115691	Transcript	ENSMUST00000226980	lincRNA	rs47613579
14:32298865-32298865	upstream_gene_variant	MODIFIER	Gm46447	ENSMUSG00000115813	Transcript	ENSMUST00000227042	lincRNA	rs47613579
14:32752735-32752735	intergenic_variant	MODIFIER	-	-	-	-	-	rs31044530
14:32752788-32752788	intergenic_variant	MODIFIER	-	-	-	-	-	rs31138499
14:33021384-33021384	intron_variant	MODIFIER	Wdfy4	ENSMUSG00000051506	Transcript	ENSMUST00000061753	protein_coding	rs46842144
14:33021384-33021384	intron_variant	MODIFIER	Wdfy4	ENSMUSG00000051506	Transcript	ENSMUST00000130509	protein_coding	rs46842144
14:33021384-33021384	intron_variant	MODIFIER	Wdfy4	ENSMUSG00000051506	Transcript	ENSMUST00000132153	protein_coding	rs46842144
14:33380478-33380478	downstream_gene_variant	MODIFIER	Mapk8	ENSMUSG00000021936	Transcript	ENSMUST00000022504	protein_coding	rs30635546
14:33380478-33380478	downstream_gene_variant	MODIFIER	Mapk8	ENSMUSG00000021936	Transcript	ENSMUST00000111942	protein_coding	rs30635546
14:33380478-33380478	downstream_gene_variant	MODIFIER	Mapk8	ENSMUSG00000021936	Transcript	ENSMUST00000111943	protein_coding	rs30635546
14:33380478-33380478	downstream_gene_variant	MODIFIER	Mapk8	ENSMUSG00000021936	Transcript	ENSMUST00000111944	protein_coding	rs30635546
14:33380478-33380478	3_prime_UTR_variant	MODIFIER	Mapk8	ENSMUSG00000021936	Transcript	ENSMUST00000111945	protein_coding	rs30635546
14:33380478-33380478	downstream_gene_variant	MODIFIER	Mapk8	ENSMUSG00000021936	Transcript	ENSMUST00000127143	retained_intron	rs30635546
14:33380478-33380478	downstream_gene_variant	MODIFIER	Mapk8	ENSMUSG00000021936	Transcript	ENSMUST00000150659	processed_transcript	rs30635546
14:34096546-34096546	synonymous_variant	LOW	Anxa8	ENSMUSG00000021950	Transcript	ENSMUST00000022519	protein_coding	rs49473125
14:34096546-34096546	synonymous_variant	LOW	Anxa8	ENSMUSG00000021950	Transcript	ENSMUST00000120077	protein_coding	rs49473125
14:34096546-34096546	intron_variant	MODIFIER	A630023A22Rik	ENSMUSG00000095493	Transcript	ENSMUST00000178958	protein_coding	rs49473125
14:34096546-34096546	intron_variant,non_coding_trans	MODIFIER	A630023A22Rik	ENSMUSG00000095493	Transcript	ENSMUST00000215823	processed_transcript	rs49473125
14:34097883-34097883	synonymous_variant	LOW	Anxa8	ENSMUSG00000021950	Transcript	ENSMUST00000022519	protein_coding	rs46479886
14:34097883-34097883	synonymous_variant	LOW	Anxa8	ENSMUSG00000021950	Transcript	ENSMUST00000120077	protein_coding	rs46479886
14:34097883-34097883	intron_variant	MODIFIER	A630023A22Rik	ENSMUSG00000095493	Transcript	ENSMUST00000178958	protein_coding	rs46479886
14:34097883-34097883	intron_variant,non_coding_trans	MODIFIER	A630023A22Rik	ENSMUSG00000095493	Transcript	ENSMUST00000215823	processed_transcript	rs46479886
14:34231585-34231585	downstream_gene_variant	MODIFIER	Syt15	ENSMUSG00000041479	Transcript	ENSMUST00000035351	protein_coding	rs48364866
14:34231585-34231585	downstream_gene_variant	MODIFIER	Syt15	ENSMUSG00000041479	Transcript	ENSMUST00000119693	protein_coding	rs48364866
14:34231585-34231585	non_coding_transcript_exon_var	MODIFIER	Gm49201	ENSMUSG00000115124	Transcript	ENSMUST00000227403	lincRNA	rs48364866
14:34231585-34231585	regulatory_region_variant	MODIFIER	-	-	RegulatoryFeature	ENSMUSR00000558972	CTCF_binding_site	rs48364866
14:34234983-34234983	downstream_gene_variant	MODIFIER	Syt15	ENSMUSG00000041479	Transcript	ENSMUST00000035351	protein_coding	rs263565489
14:34234983-34234983	downstream_gene_variant	MODIFIER	Shld2	ENSMUSG00000041471	Transcript	ENSMUST00000111917	protein_coding	rs263565489
14:34234983-34234983	upstream_gene_variant	MODIFIER	Gm49201	ENSMUSG00000115124	Transcript	ENSMUST00000227403	lincRNA	rs263565489
14:34234983-34234983	regulatory_region_variant	MODIFIER	-	-	RegulatoryFeature	ENSMUSR00000085786	promoter_flanking_region	rs263565489
14:34236575-34236575	downstream_gene_variant	MODIFIER	Shld2	ENSMUSG00000041471	Transcript	ENSMUST00000111917	protein_coding	rs214795797
14:34236575-34236575	upstream_gene_variant	MODIFIER	Gm49201	ENSMUSG00000115124	Transcript	ENSMUST00000227403	lincRNA	rs214795797
14:34236575-34236575	downstream_gene_variant	MODIFIER	Shld2	ENSMUSG00000041471	Transcript	ENSMUST00000228704	protein_coding	rs214795797
14:34314356-34314356	intron_variant	MODIFIER	Glud1	ENSMUSG00000021794	Transcript	ENSMUST00000022322	protein_coding	rs50822192
14:34314356-34314356	upstream_gene_variant	MODIFIER	Shld2	ENSMUSG00000041471	Transcript	ENSMUST00000111917	protein_coding	rs50822192
14:34314356-34314356	upstream_gene_variant	MODIFIER	Shld2	ENSMUSG00000041471	Transcript	ENSMUST00000227375	protein_coding	rs50822192
14:34314356-34314356	upstream_gene_variant	MODIFIER	Shld2	ENSMUSG00000041471	Transcript	ENSMUST00000228132	processed_transcript	rs50822192
14:34314356-34314356	upstream_gene_variant	MODIFIER	Shld2	ENSMUSG00000041471	Transcript	ENSMUST00000228704	protein_coding	rs50822192
14:34314356-34314356	regulatory_region_variant	MODIFIER	-	-	RegulatoryFeature	ENSMUSR00000085805	promoter	rs50822192
14:34315349-34315349	intron_variant	MODIFIER	Glud1	ENSMUSG00000021794	Transcript	ENSMUST00000022322	protein_coding	rs46065220
14:34315349-34315349	upstream_gene_variant	MODIFIER	Shld2	ENSMUSG00000041471	Transcript	ENSMUST00000111917	protein_coding	rs46065220
14:34315349-34315349	upstream_gene_variant	MODIFIER	Shld2	ENSMUSG00000041471	Transcript	ENSMUST00000227375	protein_coding	rs46065220
14:34315349-34315349	upstream_gene_variant	MODIFIER	Shld2	ENSMUSG00000041471	Transcript	ENSMUST00000228704	protein_coding	rs46065220
14:34315354-34315355	intron_variant	MODIFIER	Glud1	ENSMUSG00000021794	Transcript	ENSMUST00000022322	protein_coding	rs213503027
14:34315354-34315355	upstream_gene_variant	MODIFIER	Shld2	ENSMUSG00000041471	Transcript	ENSMUST00000111917	protein_coding	rs213503027
14:34315354-34315355	upstream_gene_variant	MODIFIER	Shld2	ENSMUSG00000041471	Transcript	ENSMUST00000227375	protein_coding	rs213503027
14:34315354-34315355	upstream_gene_variant	MODIFIER	Shld2	ENSMUSG00000041471	Transcript	ENSMUST00000228704	protein_coding	rs213503027
14:34329528-34329528	intron_variant	MODIFIER	Glud1	ENSMUSG00000021794	Transcript	ENSMUST00000022322	protein_coding	rs237420270
14:34329528-34329528	upstream_gene_variant	MODIFIER	Glud1	ENSMUSG00000021794	Transcript	ENSMUST00000159784	processed_transcript	rs237420270
14:34329528-34329528	intron_variant,non_coding_trans	MODIFIER	Glud1	ENSMUSG00000021794	Transcript	ENSMUST00000162912	retained_intron	rs237420270
14:34329528-34329528	upstream_gene_variant	MODIFIER	Glud1	ENSMUSG00000021794	Transcript	ENSMUST00000163955	protein_coding	rs237420270
14:34330683-34330683	intron_variant	MODIFIER	Glud1	ENSMUSG00000021794	Transcript	ENSMUST00000022322	protein_coding	rs49305699
14:34330683-34330683	upstream_gene_variant	MODIFIER	Glud1	ENSMUSG00000021794	Transcript	ENSMUST00000159784	processed_transcript	rs49305699
14:34330683-34330683	upstream_gene_variant	MODIFIER	Glud1	ENSMUSG00000021794	Transcript	ENSMUST00000161593	processed_transcript	rs49305699
14:34330683-34330683	intron_variant,non_coding_trans	MODIFIER	Glud1	ENSMUSG00000021794	Transcript	ENSMUST00000162912	retained_intron	rs49305699
14:34330683-34330683	upstream_gene_variant	MODIFIER	Glud1	ENSMUSG00000021794	Transcript	ENSMUST00000163955	protein_coding	rs49305699
14:34330683-34330683	regulatory_region_variant	MODIFIER	-	-	RegulatoryFeature	ENSMUSR00000558992	enhancer	rs49305699
14:34330701-34330701	intron_variant	MODIFIER	Glud1	ENSMUSG00000021794	Transcript	ENSMUST00000022322	protein_coding	rs50712471
14:34330701-34330701	upstream_gene_variant	MODIFIER	Glud1	ENSMUSG00000021794	Transcript	ENSMUST00000159784	processed_transcript	rs50712471
14:34330701-34330701	upstream_gene_variant	MODIFIER	Glud1	ENSMUSG00000021794	Transcript	ENSMUST00000161593	processed_transcript	rs50712471
14:34330701-34330701	intron_variant,non_coding_trans	MODIFIER	Glud1	ENSMUSG00000021794	Transcript	ENSMUST00000162912	retained_intron	rs50712471

14:34330701-34330701	upstream_gene_variant	MODIFIER	Glud1	ENSMUSG00000021794	Transcript	ENSMUST00000163955	protein_coding	rs50712471
14:34330701-34330701	regulatory_region_variant	MODIFIER	-	-	RegulatoryFeature	ENSMUSR00000558992	enhancer	rs50712471
14:34333204-34333208	intron_variant	MODIFIER	Glud1	ENSMUSG00000021794	Transcript	ENSMUST00000022322	protein_coding	-
14:34333204-34333208	intron_variant,non_coding_trans	MODIFIER	Glud1	ENSMUSG00000021794	Transcript	ENSMUST00000159784	processed_transcript	-
14:34333204-34333208	upstream_gene_variant	MODIFIER	Glud1	ENSMUSG00000021794	Transcript	ENSMUST00000161593	processed_transcript	-
14:34333204-34333208	intron_variant,non_coding_trans	MODIFIER	Glud1	ENSMUSG00000021794	Transcript	ENSMUST00000162912	retained_intron	-
14:34333204-34333208	upstream_gene_variant	MODIFIER	Glud1	ENSMUSG00000021794	Transcript	ENSMUST00000163955	protein_coding	-
14:34333348-34333348	intron_variant	MODIFIER	Glud1	ENSMUSG00000021794	Transcript	ENSMUST00000022322	protein_coding	rs232600930
14:34333348-34333348	intron_variant,non_coding_trans	MODIFIER	Glud1	ENSMUSG00000021794	Transcript	ENSMUST00000159784	processed_transcript	rs232600930
14:34333348-34333348	upstream_gene_variant	MODIFIER	Glud1	ENSMUSG00000021794	Transcript	ENSMUST00000161593	processed_transcript	rs232600930
14:34333348-34333348	intron_variant,non_coding_trans	MODIFIER	Glud1	ENSMUSG00000021794	Transcript	ENSMUST00000162912	retained_intron	rs232600930
14:34333348-34333348	upstream_gene_variant	MODIFIER	Glud1	ENSMUSG00000021794	Transcript	ENSMUST00000163955	protein_coding	rs232600930
14:34338732-34338732	intron_variant	MODIFIER	Glud1	ENSMUSG00000021794	Transcript	ENSMUST00000022322	protein_coding	rs49772968
14:34338732-34338732	intron_variant,non_coding_trans	MODIFIER	Glud1	ENSMUSG00000021794	Transcript	ENSMUST00000159784	processed_transcript	rs49772968
14:34338732-34338732	intron_variant,non_coding_trans	MODIFIER	Glud1	ENSMUSG00000021794	Transcript	ENSMUST00000161593	processed_transcript	rs49772968
14:34338732-34338732	downstream_gene_variant	MODIFIER	Glud1	ENSMUSG00000021794	Transcript	ENSMUST00000162912	retained_intron	rs49772968
14:34338732-34338732	intron_variant	MODIFIER	Glud1	ENSMUSG00000021794	Transcript	ENSMUST00000163955	protein_coding	rs49772968
14:34339286-34339286	intron_variant	MODIFIER	Glud1	ENSMUSG00000021794	Transcript	ENSMUST00000022322	protein_coding	rs51666756
14:34339286-34339286	downstream_gene_variant	MODIFIER	Glud1	ENSMUSG00000021794	Transcript	ENSMUST00000159784	processed_transcript	rs51666756
14:34339286-34339286	intron_variant,non_coding_trans	MODIFIER	Glud1	ENSMUSG00000021794	Transcript	ENSMUST00000161593	processed_transcript	rs51666756
14:34339286-34339286	intron_variant	MODIFIER	Glud1	ENSMUSG00000021794	Transcript	ENSMUST00000163955	protein_coding	rs51666756
14:34341492-34341492	intron_variant	MODIFIER	Glud1	ENSMUSG00000021794	Transcript	ENSMUST00000022322	protein_coding	rs52021721
14:34341492-34341492	downstream_gene_variant	MODIFIER	Glud1	ENSMUSG00000021794	Transcript	ENSMUST00000159784	processed_transcript	rs52021721
14:34341492-34341492	intron_variant,non_coding_trans	MODIFIER	Glud1	ENSMUSG00000021794	Transcript	ENSMUST00000161593	processed_transcript	rs52021721
14:34341492-34341492	intron_variant	MODIFIER	Glud1	ENSMUSG00000021794	Transcript	ENSMUST00000163955	protein_coding	rs52021721
14:34341492-34341492	upstream_gene_variant	MODIFIER	Gm3219	ENSMUSG00000115431	Transcript	ENSMUST00000226608	processed_pseudogene	rs52021721
14:34341532-34341532	intron_variant	MODIFIER	Glud1	ENSMUSG00000021794	Transcript	ENSMUST00000022322	protein_coding	rs49568589
14:34341532-34341532	downstream_gene_variant	MODIFIER	Glud1	ENSMUSG00000021794	Transcript	ENSMUST00000159784	processed_transcript	rs49568589
14:34341532-34341532	intron_variant,non_coding_trans	MODIFIER	Glud1	ENSMUSG00000021794	Transcript	ENSMUST00000161593	processed_transcript	rs49568589
14:34341532-34341532	intron_variant	MODIFIER	Glud1	ENSMUSG00000021794	Transcript	ENSMUST00000163955	protein_coding	rs49568589
14:34341532-34341532	upstream_gene_variant	MODIFIER	Gm3219	ENSMUSG00000115431	Transcript	ENSMUST00000226608	processed_pseudogene	rs49568589
14:36941260-36941260	5_prime_UTR_variant	MODIFIER	Ccser2	ENSMUSG00000058690	Transcript	ENSMUST00000067700	protein_coding	rs48050647
14:36941260-36941260	5_prime_UTR_variant	MODIFIER	Ccser2	ENSMUSG00000058690	Transcript	ENSMUST00000090024	protein_coding	rs48050647
14:36941260-36941260	upstream_gene_variant	MODIFIER	Ccser2	ENSMUSG00000058690	Transcript	ENSMUST00000182635	processed_transcript	rs48050647
14:45587622-45587622	downstream_gene_variant	MODIFIER	Ddhd1	ENSMUSG00000037697	Transcript	ENSMUST00000087320	protein_coding	rs31281331
14:45587622-45587622	downstream_gene_variant	MODIFIER	Ddhd1	ENSMUSG00000037697	Transcript	ENSMUST00000111828	protein_coding	rs31281331
14:45587622-45587622	upstream_gene_variant	MODIFIER	Gm15601	ENSMUSG00000085913	Transcript	ENSMUST00000122972	antisense	rs31281331
14:45587622-45587622	intron_variant,non_coding_trans	MODIFIER	Gm15601	ENSMUSG00000085913	Transcript	ENSMUST00000226558	antisense	rs31281331
14:45587622-45587622	downstream_gene_variant	MODIFIER	Gm49124	ENSMUSG00000115553	Transcript	ENSMUST00000228948	lincRNA	rs31281331
14:45587622-45587622	regulatory_region_variant	MODIFIER	-	-	RegulatoryFeature	ENSMUSR00000559640	promoter_flanking_region	rs31281331
14:45587813-45587813	downstream_gene_variant	MODIFIER	Ddhd1	ENSMUSG00000037697	Transcript	ENSMUST00000087320	protein_coding	rs216602480
14:45587813-45587813	downstream_gene_variant	MODIFIER	Ddhd1	ENSMUSG00000037697	Transcript	ENSMUST00000111828	protein_coding	rs216602480
14:45587813-45587813	non_coding_transcript_exon_var	MODIFIER	Gm15601	ENSMUSG00000085913	Transcript	ENSMUST00000122972	antisense	rs216602480
14:45587813-45587813	intron_variant,non_coding_trans	MODIFIER	Gm15601	ENSMUSG00000085913	Transcript	ENSMUST00000226558	antisense	rs216602480
14:45587813-45587813	downstream_gene_variant	MODIFIER	Gm49124	ENSMUSG00000115553	Transcript	ENSMUST00000228948	lincRNA	rs216602480
14:45587813-45587813	regulatory_region_variant	MODIFIER	-	-	RegulatoryFeature	ENSMUSR00000559640	promoter_flanking_region	rs216602480
14:45589789-45589789	downstream_gene_variant	MODIFIER	Ddhd1	ENSMUSG00000037697	Transcript	ENSMUST00000051310	protein_coding	rs50149827
14:45589789-45589789	3_prime_UTR_variant	MODIFIER	Ddhd1	ENSMUSG00000037697	Transcript	ENSMUST00000087320	protein_coding	rs50149827
14:45589789-45589789	3_prime_UTR_variant	MODIFIER	Ddhd1	ENSMUSG00000037697	Transcript	ENSMUST00000111828	protein_coding	rs50149827
14:45589789-45589789	intron_variant,non_coding_trans	MODIFIER	Gm15601	ENSMUSG00000085913	Transcript	ENSMUST00000122972	antisense	rs50149827
14:45589789-45589789	downstream_gene_variant	MODIFIER	Ddhd1	ENSMUSG00000037697	Transcript	ENSMUST00000129599	retained_intron	rs50149827
14:45589789-45589789	downstream_gene_variant	MODIFIER	Ddhd1	ENSMUSG00000037697	Transcript	ENSMUST00000149286	nonsense_mediated_decay	rs50149827
14:45589789-45589789	downstream_gene_variant	MODIFIER	Ddhd1	ENSMUSG00000037697	Transcript	ENSMUST00000152110	retained_intron	rs50149827
14:45589789-45589789	intron_variant,non_coding_trans	MODIFIER	Gm15601	ENSMUSG00000085913	Transcript	ENSMUST00000226558	antisense	rs50149827
14:45589789-45589789	downstream_gene_variant	MODIFIER	Gm49124	ENSMUSG00000115553	Transcript	ENSMUST00000228948	lincRNA	rs50149827
14:45589802-45589802	downstream_gene_variant	MODIFIER	Ddhd1	ENSMUSG00000037697	Transcript	ENSMUST00000051310	protein_coding	rs46971765
14:45589802-45589802	3_prime_UTR_variant	MODIFIER	Ddhd1	ENSMUSG00000037697	Transcript	ENSMUST00000087320	protein_coding	rs46971765
14:45589802-45589802	3_prime_UTR_variant	MODIFIER	Ddhd1	ENSMUSG00000037697	Transcript	ENSMUST00000111828	protein_coding	rs46971765
14:45589802-45589802	intron_variant,non_coding_trans	MODIFIER	Gm15601	ENSMUSG00000085913	Transcript	ENSMUST00000122972	antisense	rs46971765
14:45589802-45589802	downstream_gene_variant	MODIFIER	Ddhd1	ENSMUSG00000037697	Transcript	ENSMUST00000129599	retained_intron	rs46971765
14:45589802-45589802	downstream_gene_variant	MODIFIER	Ddhd1	ENSMUSG00000037697	Transcript	ENSMUST00000149286	nonsense_mediated_decay	rs46971765
14:45589802-45589802	downstream_gene_variant	MODIFIER	Ddhd1	ENSMUSG00000037697	Transcript	ENSMUST00000152110	retained_intron	rs46971765
14:45589802-45589802	intron_variant,non_coding_trans	MODIFIER	Gm15601	ENSMUSG00000085913	Transcript	ENSMUST00000226558	antisense	rs46971765
14:45589802-45589802	downstream_gene_variant	MODIFIER	Gm49124	ENSMUSG00000115553	Transcript	ENSMUST00000228948	lincRNA	rs46971765
14:45597307-45597307	intron_variant	MODIFIER	Ddhd1	ENSMUSG00000037697	Transcript	ENSMUST00000051310	protein_coding	rs30798359
14:45597307-45597307	intron_variant	MODIFIER	Ddhd1	ENSMUSG00000037697	Transcript	ENSMUST00000087320	protein_coding	rs30798359
14:45597307-45597307	intron_variant	MODIFIER	Ddhd1	ENSMUSG00000037697	Transcript	ENSMUST00000111828	protein_coding	rs30798359
14:45597307-45597307	non_coding_transcript_exon_var	MODIFIER	Gm15601	ENSMUSG00000085913	Transcript	ENSMUST00000122972	antisense	rs30798359
14:45597307-45597307	intron_variant,non_coding_trans	MODIFIER	Ddhd1	ENSMUSG00000037697	Transcript	ENSMUST00000129599	retained_intron	rs30798359

14:45597307-45597307	intron_variant,NMD_transcript_v	MODIFIER	Ddhd1	ENSMUSG00000037697	Transcript	ENSMUST00000141487	nonsense_mediated_decay	rs30798359
14:45597307-45597307	intron_variant,NMD_transcript_v	MODIFIER	Ddhd1	ENSMUSG00000037697	Transcript	ENSMUST00000149286	nonsense_mediated_decay	rs30798359
14:45597307-45597307	intron_variant,non_coding_trans	MODIFIER	Ddhd1	ENSMUSG00000037697	Transcript	ENSMUST00000152110	retained_intron	rs30798359
14:45597307-45597307	intron_variant,NMD_transcript_v	MODIFIER	Ddhd1	ENSMUSG00000037697	Transcript	ENSMUST00000156758	nonsense_mediated_decay	rs30798359
14:45597307-45597307	downstream_gene_variant	MODIFIER	Gm15601	ENSMUSG00000085913	Transcript	ENSMUST00000226558	antisense	rs30798359
14:45598329-45598329	intron_variant	MODIFIER	Ddhd1	ENSMUSG00000037697	Transcript	ENSMUST00000051310	protein_coding	rs30758853
14:45598329-45598329	intron_variant	MODIFIER	Ddhd1	ENSMUSG00000037697	Transcript	ENSMUST00000087320	protein_coding	rs30758853
14:45598329-45598329	intron_variant	MODIFIER	Ddhd1	ENSMUSG00000037697	Transcript	ENSMUST00000111828	protein_coding	rs30758853
14:45598329-45598329	non_coding_transcript_exon_var	MODIFIER	Gm15601	ENSMUSG00000085913	Transcript	ENSMUST00000122972	antisense	rs30758853
14:45598329-45598329	intron_variant,non_coding_trans	MODIFIER	Ddhd1	ENSMUSG00000037697	Transcript	ENSMUST00000129599	retained_intron	rs30758853
14:45598329-45598329	intron_variant,NMD_transcript_v	MODIFIER	Ddhd1	ENSMUSG00000037697	Transcript	ENSMUST00000141487	nonsense_mediated_decay	rs30758853
14:45598329-45598329	intron_variant,NMD_transcript_v	MODIFIER	Ddhd1	ENSMUSG00000037697	Transcript	ENSMUST00000149286	nonsense_mediated_decay	rs30758853
14:45598329-45598329	intron_variant,non_coding_trans	MODIFIER	Ddhd1	ENSMUSG00000037697	Transcript	ENSMUST00000152110	retained_intron	rs30758853
14:45598329-45598329	intron_variant,NMD_transcript_v	MODIFIER	Ddhd1	ENSMUSG00000037697	Transcript	ENSMUST00000156758	nonsense_mediated_decay	rs30758853
14:45598329-45598329	downstream_gene_variant	MODIFIER	Gm15601	ENSMUSG00000085913	Transcript	ENSMUST00000226558	antisense	rs30758853
14:45598482-45598482	intron_variant	MODIFIER	Ddhd1	ENSMUSG00000037697	Transcript	ENSMUST00000051310	protein_coding	rs36830551
14:45598482-45598482	intron_variant	MODIFIER	Ddhd1	ENSMUSG00000037697	Transcript	ENSMUST00000087320	protein_coding	rs36830551
14:45598482-45598482	intron_variant	MODIFIER	Ddhd1	ENSMUSG00000037697	Transcript	ENSMUST00000111828	protein_coding	rs36830551
14:45598482-45598482	non_coding_transcript_exon_var	MODIFIER	Gm15601	ENSMUSG00000085913	Transcript	ENSMUST00000122972	antisense	rs36830551
14:45598482-45598482	intron_variant,non_coding_trans	MODIFIER	Ddhd1	ENSMUSG00000037697	Transcript	ENSMUST00000129599	retained_intron	rs36830551
14:45598482-45598482	intron_variant,NMD_transcript_v	MODIFIER	Ddhd1	ENSMUSG00000037697	Transcript	ENSMUST00000141487	nonsense_mediated_decay	rs36830551
14:45598482-45598482	intron_variant,NMD_transcript_v	MODIFIER	Ddhd1	ENSMUSG00000037697	Transcript	ENSMUST00000149286	nonsense_mediated_decay	rs36830551
14:45598482-45598482	intron_variant,non_coding_trans	MODIFIER	Ddhd1	ENSMUSG00000037697	Transcript	ENSMUST00000152110	retained_intron	rs36830551
14:45598482-45598482	intron_variant,NMD_transcript_v	MODIFIER	Ddhd1	ENSMUSG00000037697	Transcript	ENSMUST00000156758	nonsense_mediated_decay	rs36830551
14:45598482-45598482	downstream_gene_variant	MODIFIER	Gm15601	ENSMUSG00000085913	Transcript	ENSMUST00000226558	antisense	rs36830551
14:45613904-45613904	intron_variant	MODIFIER	Ddhd1	ENSMUSG00000037697	Transcript	ENSMUST00000051310	protein_coding	rs46318729
14:45613904-45613904	intron_variant	MODIFIER	Ddhd1	ENSMUSG00000037697	Transcript	ENSMUST00000087320	protein_coding	rs46318729
14:45613904-45613904	intron_variant	MODIFIER	Ddhd1	ENSMUSG00000037697	Transcript	ENSMUST00000111828	protein_coding	rs46318729
14:45613904-45613904	upstream_gene_variant	MODIFIER	Ddhd1	ENSMUSG00000037697	Transcript	ENSMUST00000126428	retained_intron	rs46318729
14:45613904-45613904	intron_variant,non_coding_trans	MODIFIER	Ddhd1	ENSMUSG00000037697	Transcript	ENSMUST00000129599	retained_intron	rs46318729
14:45613904-45613904	intron_variant,NMD_transcript_v	MODIFIER	Ddhd1	ENSMUSG00000037697	Transcript	ENSMUST00000141487	nonsense_mediated_decay	rs46318729
14:45613904-45613904	intron_variant,NMD_transcript_v	MODIFIER	Ddhd1	ENSMUSG00000037697	Transcript	ENSMUST00000149286	nonsense_mediated_decay	rs46318729
14:45613904-45613904	intron_variant,non_coding_trans	MODIFIER	Ddhd1	ENSMUSG00000037697	Transcript	ENSMUST00000152110	retained_intron	rs46318729
14:45613904-45613904	non_coding_transcript_exon_var	MODIFIER	Gm49125	ENSMUSG00000115148	Transcript	ENSMUST00000227258	TEC	rs46318729
14:45623741-45623741	intron_variant	MODIFIER	Ddhd1	ENSMUSG00000037697	Transcript	ENSMUST00000051310	protein_coding	rs240626004
14:45623741-45623741	intron_variant	MODIFIER	Ddhd1	ENSMUSG00000037697	Transcript	ENSMUST00000087320	protein_coding	rs240626004
14:45623741-45623741	intron_variant	MODIFIER	Ddhd1	ENSMUSG00000037697	Transcript	ENSMUST00000111828	protein_coding	rs240626004
14:45623741-45623741	upstream_gene_variant	MODIFIER	Ddhd1	ENSMUSG00000037697	Transcript	ENSMUST00000129599	retained_intron	rs240626004
14:45623741-45623741	upstream_gene_variant	MODIFIER	Ddhd1	ENSMUSG00000037697	Transcript	ENSMUST00000141487	nonsense_mediated_decay	rs240626004
14:45623741-45623741	intron_variant,non_coding_trans	MODIFIER	Ddhd1	ENSMUSG00000037697	Transcript	ENSMUST00000147551	processed_transcript	rs240626004
14:45623741-45623741	intron_variant,NMD_transcript_v	MODIFIER	Ddhd1	ENSMUSG00000037697	Transcript	ENSMUST00000149286	nonsense_mediated_decay	rs240626004
14:45623741-45623741	upstream_gene_variant	MODIFIER	Ddhd1	ENSMUSG00000037697	Transcript	ENSMUST00000152110	retained_intron	rs240626004
14:45623741-45623741	downstream_gene_variant	MODIFIER	Ddhd1	ENSMUSG00000037697	Transcript	ENSMUST00000153547	retained_intron	rs240626004
14:45623741-45623741	intron_variant	MODIFIER	Ddhd1	ENSMUSG00000037697	Transcript	ENSMUST00000226301	protein_coding	rs240626004
14:45627494-45627494	intron_variant	MODIFIER	Ddhd1	ENSMUSG00000037697	Transcript	ENSMUST00000051310	protein_coding	rs31280269
14:45627494-45627494	intron_variant	MODIFIER	Ddhd1	ENSMUSG00000037697	Transcript	ENSMUST00000087320	protein_coding	rs31280269
14:45627494-45627494	intron_variant	MODIFIER	Ddhd1	ENSMUSG00000037697	Transcript	ENSMUST00000111828	protein_coding	rs31280269
14:45627494-45627494	upstream_gene_variant	MODIFIER	Ddhd1	ENSMUSG00000037697	Transcript	ENSMUST00000129599	retained_intron	rs31280269
14:45627494-45627494	intron_variant,non_coding_trans	MODIFIER	Ddhd1	ENSMUSG00000037697	Transcript	ENSMUST00000147551	processed_transcript	rs31280269
14:45627494-45627494	intron_variant,NMD_transcript_v	MODIFIER	Ddhd1	ENSMUSG00000037697	Transcript	ENSMUST00000149286	nonsense_mediated_decay	rs31280269
14:45627494-45627494	downstream_gene_variant	MODIFIER	Ddhd1	ENSMUSG00000037697	Transcript	ENSMUST00000153547	retained_intron	rs31280269
14:45627494-45627494	intron_variant	MODIFIER	Ddhd1	ENSMUSG00000037697	Transcript	ENSMUST00000226301	protein_coding	rs31280269
14:45627494-45627494	downstream_gene_variant	MODIFIER	Ddhd1	ENSMUSG00000037697	Transcript	ENSMUST00000226559	retained_intron	rs31280269
14:45629468-45629468	intron_variant	MODIFIER	Ddhd1	ENSMUSG00000037697	Transcript	ENSMUST00000051310	protein_coding	rs30431092
14:45629468-45629468	intron_variant	MODIFIER	Ddhd1	ENSMUSG00000037697	Transcript	ENSMUST00000087320	protein_coding	rs30431092
14:45629468-45629468	intron_variant	MODIFIER	Ddhd1	ENSMUSG00000037697	Transcript	ENSMUST00000111828	protein_coding	rs30431092
14:45629468-45629468	intron_variant,non_coding_trans	MODIFIER	Ddhd1	ENSMUSG00000037697	Transcript	ENSMUST00000147551	processed_transcript	rs30431092
14:45629468-45629468	intron_variant,NMD_transcript_v	MODIFIER	Ddhd1	ENSMUSG00000037697	Transcript	ENSMUST00000149286	nonsense_mediated_decay	rs30431092
14:45629468-45629468	intron_variant,non_coding_trans	MODIFIER	Ddhd1	ENSMUSG00000037697	Transcript	ENSMUST00000153547	retained_intron	rs30431092
14:45629468-45629468	intron_variant	MODIFIER	Ddhd1	ENSMUSG00000037697	Transcript	ENSMUST00000226301	protein_coding	rs30431092
14:45629468-45629468	downstream_gene_variant	MODIFIER	Ddhd1	ENSMUSG00000037697	Transcript	ENSMUST00000226559	retained_intron	rs30431092
14:46778715-46778715	intron_variant	MODIFIER	Cnih1	ENSMUSG00000015759	Transcript	ENSMUST00000015903	protein_coding	rs46155166
14:46778715-46778715	intron_variant	MODIFIER	Cnih1	ENSMUSG00000015759	Transcript	ENSMUST00000146629	protein_coding	rs46155166
14:46778715-46778715	intron_variant,non_coding_trans	MODIFIER	Cnih1	ENSMUSG00000015759	Transcript	ENSMUST00000227583	retained_intron	rs46155166
14:46814046-46814047	intron_variant	MODIFIER	Gmfb	ENSMUSG00000062014	Transcript	ENSMUST00000079314	protein_coding	rs218050845
14:46814046-46814047	intron_variant	MODIFIER	Gmfb	ENSMUSG00000062014	Transcript	ENSMUST00000111817	protein_coding	rs218050845
14:46814046-46814047	downstream_gene_variant	MODIFIER	Gmfb	ENSMUSG00000062014	Transcript	ENSMUST00000145958	processed_transcript	rs218050845
14:46814046-46814047	intron_variant	MODIFIER	Gmfb	ENSMUSG00000062014	Transcript	ENSMUST00000226333	protein_coding	rs218050845
14:46814046-46814047	downstream_gene_variant	MODIFIER	Gmfb	ENSMUSG00000062014	Transcript	ENSMUST00000226842	retained_intron	rs218050845

14:46814046-46814047	intron_variant	MODIFIER	Gmfb	ENSMUSG00000062014	Transcript	ENSMUST00000226937	protein_coding	rs218050845
14:46814046-46814047	intron_variant,non_coding_trans	MODIFIER	Gmfb	ENSMUSG00000062014	Transcript	ENSMUST00000228065	processed_transcript	rs218050845
14:46814046-46814047	intron_variant,non_coding_trans	MODIFIER	Gmfb	ENSMUSG00000062014	Transcript	ENSMUST00000228515	retained_intron	rs218050845
14:46840418-46840418	intron_variant	MODIFIER	Cgrrf1	ENSMUSG00000055128	Transcript	ENSMUST00000068532	protein_coding	rs52003483
14:46840418-46840418	intron_variant,non_coding_trans	MODIFIER	Cgrrf1	ENSMUSG00000055128	Transcript	ENSMUST00000133790	retained_intron	rs52003483
14:46840418-46840418	intron_variant	MODIFIER	Cgrrf1	ENSMUSG00000055128	Transcript	ENSMUST00000133989	protein_coding	rs52003483
14:46840418-46840418	downstream_gene_variant	MODIFIER	Cgrrf1	ENSMUSG00000055128	Transcript	ENSMUST00000140114	protein_coding	rs52003483
14:46840418-46840418	intron_variant,NMD_transcript_v	MODIFIER	Cgrrf1	ENSMUSG00000055128	Transcript	ENSMUST00000226861	nonsense_mediated_decay	rs52003483
14:46840418-46840418	upstream_gene_variant	MODIFIER	Gm48949	ENSMUSG00000115417	Transcript	ENSMUST00000228424	sense_intronic	rs52003483
14:46840583-46840583	intron_variant	MODIFIER	Cgrrf1	ENSMUSG00000055128	Transcript	ENSMUST00000068532	protein_coding	rs30828502
14:46840583-46840583	intron_variant,non_coding_trans	MODIFIER	Cgrrf1	ENSMUSG00000055128	Transcript	ENSMUST00000133790	retained_intron	rs30828502
14:46840583-46840583	intron_variant	MODIFIER	Cgrrf1	ENSMUSG00000055128	Transcript	ENSMUST00000133989	protein_coding	rs30828502
14:46840583-46840583	downstream_gene_variant	MODIFIER	Cgrrf1	ENSMUSG00000055128	Transcript	ENSMUST00000140114	protein_coding	rs30828502
14:46840583-46840583	intron_variant,NMD_transcript_v	MODIFIER	Cgrrf1	ENSMUSG00000055128	Transcript	ENSMUST00000226861	nonsense_mediated_decay	rs30828502
14:46840583-46840583	upstream_gene_variant	MODIFIER	Gm48949	ENSMUSG00000115417	Transcript	ENSMUST00000228424	sense_intronic	rs30828502
14:46842494-46842494	intron_variant	MODIFIER	Cgrrf1	ENSMUSG00000055128	Transcript	ENSMUST00000068532	protein_coding	rs46541737
14:46842494-46842494	intron_variant,non_coding_trans	MODIFIER	Cgrrf1	ENSMUSG00000055128	Transcript	ENSMUST00000133790	retained_intron	rs46541737
14:46842494-46842494	intron_variant	MODIFIER	Cgrrf1	ENSMUSG00000055128	Transcript	ENSMUST00000133989	protein_coding	rs46541737
14:46842494-46842494	downstream_gene_variant	MODIFIER	Cgrrf1	ENSMUSG00000055128	Transcript	ENSMUST00000140114	protein_coding	rs46541737
14:46842494-46842494	3_prime_UTR_variant,NMD_tran	MODIFIER	Cgrrf1	ENSMUSG00000055128	Transcript	ENSMUST00000226861	nonsense_mediated_decay	rs46541737
14:46842494-46842494	upstream_gene_variant	MODIFIER	Gm48949	ENSMUSG00000115417	Transcript	ENSMUST00000228424	sense_intronic	rs46541737
14:46846780-46846780	intron_variant	MODIFIER	Cgrrf1	ENSMUSG00000055128	Transcript	ENSMUST00000068532	protein_coding	rs30827725
14:46846780-46846780	non_coding_transcript_exon_var	MODIFIER	Cgrrf1	ENSMUSG00000055128	Transcript	ENSMUST00000133790	retained_intron	rs30827725
14:46846780-46846780	downstream_gene_variant	MODIFIER	Cgrrf1	ENSMUSG00000055128	Transcript	ENSMUST00000133989	protein_coding	rs30827725
14:46846780-46846780	intron_variant,NMD_transcript_v	MODIFIER	Cgrrf1	ENSMUSG00000055128	Transcript	ENSMUST00000226861	nonsense_mediated_decay	rs30827725
14:46846780-46846780	downstream_gene_variant	MODIFIER	Gm48949	ENSMUSG00000115417	Transcript	ENSMUST00000228424	sense_intronic	rs30827725
14:46847939-46847939	intron_variant	MODIFIER	Cgrrf1	ENSMUSG00000055128	Transcript	ENSMUST00000068532	protein_coding	rs30353058
14:46847939-46847939	non_coding_transcript_exon_var	MODIFIER	Cgrrf1	ENSMUSG00000055128	Transcript	ENSMUST00000133790	retained_intron	rs30353058
14:46847939-46847939	downstream_gene_variant	MODIFIER	Cgrrf1	ENSMUSG00000055128	Transcript	ENSMUST00000133989	protein_coding	rs30353058
14:46847939-46847939	intron_variant,NMD_transcript_v	MODIFIER	Cgrrf1	ENSMUSG00000055128	Transcript	ENSMUST00000226861	nonsense_mediated_decay	rs30353058
14:46847939-46847939	downstream_gene_variant	MODIFIER	Gm48949	ENSMUSG00000115417	Transcript	ENSMUST00000228424	sense_intronic	rs30353058
14:47246358-47246358	intron_variant	MODIFIER	Wdhd1	ENSMUSG00000037572	Transcript	ENSMUST00000111792	protein_coding	rs31424134
14:47246358-47246358	intron_variant	MODIFIER	Wdhd1	ENSMUSG00000037572	Transcript	ENSMUST00000187531	protein_coding	rs31424134
14:47246358-47246358	downstream_gene_variant	MODIFIER	Wdhd1	ENSMUSG00000037572	Transcript	ENSMUST00000227041	protein_coding	rs31424134
14:47246358-47246358	upstream_gene_variant	MODIFIER	Wdhd1	ENSMUSG00000037572	Transcript	ENSMUST00000228810	retained_intron	rs31424134
14:47251360-47251360	intron_variant	MODIFIER	Wdhd1	ENSMUSG00000037572	Transcript	ENSMUST00000111792	protein_coding	rs49087341
14:47251360-47251360	intron_variant	MODIFIER	Wdhd1	ENSMUSG00000037572	Transcript	ENSMUST00000187531	protein_coding	rs49087341
14:47251360-47251360	intron_variant	MODIFIER	Wdhd1	ENSMUSG00000037572	Transcript	ENSMUST00000227041	protein_coding	rs49087341
14:47268030-47268030	intron_variant	MODIFIER	Wdhd1	ENSMUSG00000037572	Transcript	ENSMUST00000111790	protein_coding	rs50829698
14:47268030-47268030	intron_variant	MODIFIER	Wdhd1	ENSMUSG00000037572	Transcript	ENSMUST00000111792	protein_coding	rs50829698
14:47268030-47268030	downstream_gene_variant	MODIFIER	Gm24378	ENSMUSG00000084648	Transcript	ENSMUST00000122699	snRNA	rs50829698
14:47268030-47268030	downstream_gene_variant	MODIFIER	Wdhd1	ENSMUSG00000037572	Transcript	ENSMUST00000139124	retained_intron	rs50829698
14:47268030-47268030	intron_variant	MODIFIER	Wdhd1	ENSMUSG00000037572	Transcript	ENSMUST00000187531	protein_coding	rs50829698
14:47322285-47322285	3_prime_UTR_variant	MODIFIER	Mapk1ip1l	ENSMUSG00000021840	Transcript	ENSMUST00000164235	protein_coding	rs48991040
14:47322285-47322285	3_prime_UTR_variant	MODIFIER	Mapk1ip1l	ENSMUSG00000021840	Transcript	ENSMUST00000166743	protein_coding	rs48991040
14:47322285-47322285	upstream_gene_variant	MODIFIER	Gm49190	ENSMUSG00000115728	Transcript	ENSMUST00000227470	processed_pseudogene	rs48991040
14:47543560-47543560	intron_variant	MODIFIER	Atg14	ENSMUSG00000037526	Transcript	ENSMUST00000042988	protein_coding	rs30159909
14:47543560-47543560	intron_variant	MODIFIER	Atg14	ENSMUSG00000037526	Transcript	ENSMUST00000226299	protein_coding	rs30159909
14:47543560-47543560	intron_variant,non_coding_trans	MODIFIER	Gm49004	ENSMUSG00000115823	Transcript	ENSMUST00000228568	antisense	rs30159909
14:47719654-47719654	intron_variant	MODIFIER	Ktn1	ENSMUSG00000021843	Transcript	ENSMUST00000022391	protein_coding	rs46736992
14:47719654-47719654	intron_variant	MODIFIER	Ktn1	ENSMUSG00000021843	Transcript	ENSMUST00000185343	protein_coding	rs46736992
14:47719654-47719654	intron_variant	MODIFIER	Ktn1	ENSMUSG00000021843	Transcript	ENSMUST00000185940	protein_coding	rs46736992
14:47719654-47719654	intron_variant	MODIFIER	Ktn1	ENSMUSG00000021843	Transcript	ENSMUST00000186627	protein_coding	rs46736992
14:47719654-47719654	downstream_gene_variant	MODIFIER	Ktn1	ENSMUSG00000021843	Transcript	ENSMUST00000186700	retained_intron	rs46736992
14:47719654-47719654	intron_variant	MODIFIER	Ktn1	ENSMUSG00000021843	Transcript	ENSMUST00000186761	protein_coding	rs46736992
14:47719654-47719654	intron_variant	MODIFIER	Ktn1	ENSMUSG00000021843	Transcript	ENSMUST00000187039	protein_coding	rs46736992
14:47719654-47719654	intron_variant	MODIFIER	Ktn1	ENSMUSG00000021843	Transcript	ENSMUST00000187262	protein_coding	rs46736992
14:47719654-47719654	intron_variant	MODIFIER	Ktn1	ENSMUSG00000021843	Transcript	ENSMUST00000187839	protein_coding	rs46736992
14:47719654-47719654	intron_variant,non_coding_trans	MODIFIER	Ktn1	ENSMUSG00000021843	Transcript	ENSMUST00000188049	retained_intron	rs46736992
14:47719654-47719654	intron_variant	MODIFIER	Ktn1	ENSMUSG00000021843	Transcript	ENSMUST00000188330	protein_coding	rs46736992
14:47719654-47719654	intron_variant	MODIFIER	Ktn1	ENSMUSG00000021843	Transcript	ENSMUST00000188553	protein_coding	rs46736992
14:47719654-47719654	intron_variant	MODIFIER	Ktn1	ENSMUSG00000021843	Transcript	ENSMUST00000189101	protein_coding	rs46736992
14:47719654-47719654	intron_variant	MODIFIER	Ktn1	ENSMUSG00000021843	Transcript	ENSMUST00000189533	protein_coding	rs46736992
14:47719654-47719654	intron_variant,NMD_transcript_v	MODIFIER	Ktn1	ENSMUSG00000021843	Transcript	ENSMUST00000189986	nonsense_mediated_decay	rs46736992
14:47719654-47719654	intron_variant	MODIFIER	Ktn1	ENSMUSG00000021843	Transcript	ENSMUST00000190182	protein_coding	rs46736992
14:47719654-47719654	intron_variant	MODIFIER	Ktn1	ENSMUSG00000021843	Transcript	ENSMUST00000190252	protein_coding	rs46736992
14:47719654-47719654	intron_variant	MODIFIER	Ktn1	ENSMUSG00000021843	Transcript	ENSMUST00000190535	protein_coding	rs46736992
14:47719654-47719654	intron_variant	MODIFIER	Ktn1	ENSMUSG00000021843	Transcript	ENSMUST00000190999	protein_coding	rs46736992
14:47719654-47719654	intron_variant	MODIFIER	Ktn1	ENSMUSG00000021843	Transcript	ENSMUST00000191018	protein_coding	rs46736992

14:47719654-47719654	intron_variant	MODIFIER	Ktn1	ENSMUSG00000021843	Transcript	ENSMUST00000191446	protein_coding	rs46736992
14:47719654-47719654	intron_variant	MODIFIER	Ktn1	ENSMUSG00000021843	Transcript	ENSMUST00000191511	protein_coding	rs46736992
14:47729295-47729295	intron_variant	MODIFIER	Ktn1	ENSMUSG00000021843	Transcript	ENSMUST00000022391	protein_coding	rs48819924
14:47729295-47729295	intron_variant	MODIFIER	Ktn1	ENSMUSG00000021843	Transcript	ENSMUST00000185343	protein_coding	rs48819924
14:47729295-47729295	intron_variant	MODIFIER	Ktn1	ENSMUSG00000021843	Transcript	ENSMUST00000185940	protein_coding	rs48819924
14:47729295-47729295	intron_variant	MODIFIER	Ktn1	ENSMUSG00000021843	Transcript	ENSMUST00000186627	protein_coding	rs48819924
14:47729295-47729295	intron_variant	MODIFIER	Ktn1	ENSMUSG00000021843	Transcript	ENSMUST00000186761	protein_coding	rs48819924
14:47729295-47729295	intron_variant	MODIFIER	Ktn1	ENSMUSG00000021843	Transcript	ENSMUST00000187039	protein_coding	rs48819924
14:47729295-47729295	intron_variant	MODIFIER	Ktn1	ENSMUSG00000021843	Transcript	ENSMUST00000187262	protein_coding	rs48819924
14:47729295-47729295	intron_variant	MODIFIER	Ktn1	ENSMUSG00000021843	Transcript	ENSMUST00000187839	protein_coding	rs48819924
14:47729295-47729295	downstream_gene_variant	MODIFIER	Ktn1	ENSMUSG00000021843	Transcript	ENSMUST00000188049	retained_intron	rs48819924
14:47729295-47729295	downstream_gene_variant	MODIFIER	Ktn1	ENSMUSG00000021843	Transcript	ENSMUST00000188263	retained_intron	rs48819924
14:47729295-47729295	intron_variant	MODIFIER	Ktn1	ENSMUSG00000021843	Transcript	ENSMUST00000188330	protein_coding	rs48819924
14:47729295-47729295	intron_variant	MODIFIER	Ktn1	ENSMUSG00000021843	Transcript	ENSMUST00000188553	protein_coding	rs48819924
14:47729295-47729295	intron_variant	MODIFIER	Ktn1	ENSMUSG00000021843	Transcript	ENSMUST00000189101	protein_coding	rs48819924
14:47729295-47729295	intron_variant	MODIFIER	Ktn1	ENSMUSG00000021843	Transcript	ENSMUST00000189533	protein_coding	rs48819924
14:47729295-47729295	intron_variant,NMD_transcript_v	MODIFIER	Ktn1	ENSMUSG00000021843	Transcript	ENSMUST00000189986	nonsense_mediated_decay	rs48819924
14:47729295-47729295	intron_variant	MODIFIER	Ktn1	ENSMUSG00000021843	Transcript	ENSMUST00000190182	protein_coding	rs48819924
14:47729295-47729295	intron_variant	MODIFIER	Ktn1	ENSMUSG00000021843	Transcript	ENSMUST00000190252	protein_coding	rs48819924
14:47729295-47729295	intron_variant	MODIFIER	Ktn1	ENSMUSG00000021843	Transcript	ENSMUST00000190535	protein_coding	rs48819924
14:47729295-47729295	upstream_gene_variant	MODIFIER	Ktn1	ENSMUSG00000021843	Transcript	ENSMUST00000190857	retained_intron	rs48819924
14:47729295-47729295	intron_variant	MODIFIER	Ktn1	ENSMUSG00000021843	Transcript	ENSMUST00000190999	protein_coding	rs48819924
14:47729295-47729295	intron_variant	MODIFIER	Ktn1	ENSMUSG00000021843	Transcript	ENSMUST00000191018	protein_coding	rs48819924
14:47729295-47729295	intron_variant	MODIFIER	Ktn1	ENSMUSG00000021843	Transcript	ENSMUST00000191446	protein_coding	rs48819924
14:47729295-47729295	intron_variant	MODIFIER	Ktn1	ENSMUSG00000021843	Transcript	ENSMUST00000191511	protein_coding	rs48819924
14:47737476-47737476	downstream_gene_variant	MODIFIER	Ktn1	ENSMUSG00000021843	Transcript	ENSMUST00000022391	protein_coding	rs31117213
14:47737476-47737476	downstream_gene_variant	MODIFIER	Ktn1	ENSMUSG00000021843	Transcript	ENSMUST00000185343	protein_coding	rs31117213
14:47737476-47737476	downstream_gene_variant	MODIFIER	Ktn1	ENSMUSG00000021843	Transcript	ENSMUST00000185940	protein_coding	rs31117213
14:47737476-47737476	downstream_gene_variant	MODIFIER	Ktn1	ENSMUSG00000021843	Transcript	ENSMUST00000186627	protein_coding	rs31117213
14:47737476-47737476	downstream_gene_variant	MODIFIER	Ktn1	ENSMUSG00000021843	Transcript	ENSMUST00000186761	protein_coding	rs31117213
14:47737476-47737476	downstream_gene_variant	MODIFIER	Ktn1	ENSMUSG00000021843	Transcript	ENSMUST00000187039	protein_coding	rs31117213
14:47737476-47737476	downstream_gene_variant	MODIFIER	Ktn1	ENSMUSG00000021843	Transcript	ENSMUST00000187262	protein_coding	rs31117213
14:47737476-47737476	3_prime_UTR_variant	MODIFIER	Ktn1	ENSMUSG00000021843	Transcript	ENSMUST00000187839	protein_coding	rs31117213
14:47737476-47737476	downstream_gene_variant	MODIFIER	Ktn1	ENSMUSG00000021843	Transcript	ENSMUST00000188330	protein_coding	rs31117213
14:47737476-47737476	downstream_gene_variant	MODIFIER	Ktn1	ENSMUSG00000021843	Transcript	ENSMUST00000188553	protein_coding	rs31117213
14:47737476-47737476	downstream_gene_variant	MODIFIER	Ktn1	ENSMUSG00000021843	Transcript	ENSMUST00000189101	protein_coding	rs31117213
14:47737476-47737476	downstream_gene_variant	MODIFIER	Ktn1	ENSMUSG00000021843	Transcript	ENSMUST00000189533	protein_coding	rs31117213
14:47737476-47737476	downstream_gene_variant	MODIFIER	Ktn1	ENSMUSG00000021843	Transcript	ENSMUST00000189986	nonsense_mediated_decay	rs31117213
14:47737476-47737476	downstream_gene_variant	MODIFIER	Ktn1	ENSMUSG00000021843	Transcript	ENSMUST00000190182	protein_coding	rs31117213
14:47737476-47737476	downstream_gene_variant	MODIFIER	Ktn1	ENSMUSG00000021843	Transcript	ENSMUST00000190252	protein_coding	rs31117213
14:47737476-47737476	downstream_gene_variant	MODIFIER	Ktn1	ENSMUSG00000021843	Transcript	ENSMUST00000190535	protein_coding	rs31117213
14:47737476-47737476	downstream_gene_variant	MODIFIER	Ktn1	ENSMUSG00000021843	Transcript	ENSMUST00000190857	retained_intron	rs31117213
14:47737476-47737476	downstream_gene_variant	MODIFIER	Ktn1	ENSMUSG00000021843	Transcript	ENSMUST00000190999	protein_coding	rs31117213
14:47737476-47737476	downstream_gene_variant	MODIFIER	Ktn1	ENSMUSG00000021843	Transcript	ENSMUST00000191018	protein_coding	rs31117213
14:47737476-47737476	downstream_gene_variant	MODIFIER	Ktn1	ENSMUSG00000021843	Transcript	ENSMUST00000191446	protein_coding	rs31117213
14:47737476-47737476	downstream_gene_variant	MODIFIER	Ktn1	ENSMUSG00000021843	Transcript	ENSMUST00000191511	protein_coding	rs31117213
14:47739415-47739415	downstream_gene_variant	MODIFIER	Ktn1	ENSMUSG00000021843	Transcript	ENSMUST00000022391	protein_coding	rs48098030
14:47739415-47739415	downstream_gene_variant	MODIFIER	Ktn1	ENSMUSG00000021843	Transcript	ENSMUST00000185343	protein_coding	rs48098030
14:47739415-47739415	downstream_gene_variant	MODIFIER	Ktn1	ENSMUSG00000021843	Transcript	ENSMUST00000185940	protein_coding	rs48098030
14:47739415-47739415	downstream_gene_variant	MODIFIER	Ktn1	ENSMUSG00000021843	Transcript	ENSMUST00000186627	protein_coding	rs48098030
14:47739415-47739415	downstream_gene_variant	MODIFIER	Ktn1	ENSMUSG00000021843	Transcript	ENSMUST00000186761	protein_coding	rs48098030
14:47739415-47739415	downstream_gene_variant	MODIFIER	Ktn1	ENSMUSG00000021843	Transcript	ENSMUST00000187039	protein_coding	rs48098030
14:47739415-47739415	downstream_gene_variant	MODIFIER	Ktn1	ENSMUSG00000021843	Transcript	ENSMUST00000187262	protein_coding	rs48098030
14:47739415-47739415	3_prime_UTR_variant	MODIFIER	Ktn1	ENSMUSG00000021843	Transcript	ENSMUST00000187839	protein_coding	rs48098030
14:47739415-47739415	downstream_gene_variant	MODIFIER	Ktn1	ENSMUSG00000021843	Transcript	ENSMUST00000188330	protein_coding	rs48098030
14:47739415-47739415	downstream_gene_variant	MODIFIER	Ktn1	ENSMUSG00000021843	Transcript	ENSMUST00000188553	protein_coding	rs48098030
14:47739415-47739415	downstream_gene_variant	MODIFIER	Ktn1	ENSMUSG00000021843	Transcript	ENSMUST00000189101	protein_coding	rs48098030
14:47739415-47739415	downstream_gene_variant	MODIFIER	Ktn1	ENSMUSG00000021843	Transcript	ENSMUST00000189533	protein_coding	rs48098030
14:47739415-47739415	downstream_gene_variant	MODIFIER	Ktn1	ENSMUSG00000021843	Transcript	ENSMUST00000189986	nonsense_mediated_decay	rs48098030
14:47739415-47739415	downstream_gene_variant	MODIFIER	Ktn1	ENSMUSG00000021843	Transcript	ENSMUST00000190182	protein_coding	rs48098030
14:47739415-47739415	downstream_gene_variant	MODIFIER	Ktn1	ENSMUSG00000021843	Transcript	ENSMUST00000190252	protein_coding	rs48098030
14:47739415-47739415	downstream_gene_variant	MODIFIER	Ktn1	ENSMUSG00000021843	Transcript	ENSMUST00000190535	protein_coding	rs48098030
14:47739415-47739415	downstream_gene_variant	MODIFIER	Ktn1	ENSMUSG00000021843	Transcript	ENSMUST00000190857	retained_intron	rs48098030
14:47739415-47739415	downstream_gene_variant	MODIFIER	Ktn1	ENSMUSG00000021843	Transcript	ENSMUST00000190999	protein_coding	rs48098030
14:47739415-47739415	downstream_gene_variant	MODIFIER	Ktn1	ENSMUSG00000021843	Transcript	ENSMUST00000191018	protein_coding	rs48098030
14:47739415-47739415	downstream_gene_variant	MODIFIER	Ktn1	ENSMUSG00000021843	Transcript	ENSMUST00000191446	protein_coding	rs48098030
14:47739415-47739415	downstream_gene_variant	MODIFIER	Ktn1	ENSMUSG00000021843	Transcript	ENSMUST00000191511	protein_coding	rs48098030
14:48241292-48241292	intron_variant	MODIFIER	Peli2	ENSMUSG00000021846	Transcript	ENSMUST00000073150	protein_coding	rs48227708
14:48241292-48241292	downstream_gene_variant	MODIFIER	Peli2	ENSMUSG00000021846	Transcript	ENSMUST00000226260	processed_transcript	rs48227708

14:48241292-48241292	intron_variant,NMD_transcript_v	MODIFIER	Peli2	ENSMUSG00000021846	Transcript	ENSMUST00000226828	nonsense_mediated_decay	rs48227708
14:48241292-48241292	intron_variant	MODIFIER	Peli2	ENSMUSG00000021846	Transcript	ENSMUST00000227362	protein_coding	rs48227708
14:48241292-48241292	intron_variant,non_coding_trans	MODIFIER	Peli2	ENSMUSG00000021846	Transcript	ENSMUST00000228519	processed_transcript	rs48227708
14:48501176-48501176	intron_variant	MODIFIER	Tmem260	ENSMUSG00000036339	Transcript	ENSMUST00000111735	protein_coding	rs31093505
14:48501176-48501176	intron_variant	MODIFIER	Tmem260	ENSMUSG00000036339	Transcript	ENSMUST00000124720	protein_coding	rs31093505
14:48501176-48501176	non_coding_transcript_exon_var	MODIFIER	Tmem260	ENSMUSG00000036339	Transcript	ENSMUST00000133744	retained_intron	rs31093505
14:48501176-48501176	intron_variant,NMD_transcript_v	MODIFIER	Tmem260	ENSMUSG00000036339	Transcript	ENSMUST00000153765	nonsense_mediated_decay	rs31093505
14:48501176-48501176	intron_variant,non_coding_trans	MODIFIER	Tmem260	ENSMUSG00000036339	Transcript	ENSMUST00000156480	retained_intron	rs31093505
14:48501176-48501176	upstream_gene_variant	MODIFIER	Tmem260	ENSMUSG00000036339	Transcript	ENSMUST00000226344	retained_intron	rs31093505
14:48501176-48501176	intron_variant	MODIFIER	Tmem260	ENSMUSG00000036339	Transcript	ENSMUST00000226422	protein_coding	rs31093505
14:48501176-48501176	intron_variant	MODIFIER	Tmem260	ENSMUSG00000036339	Transcript	ENSMUST00000227440	protein_coding	rs31093505
14:48501176-48501176	downstream_gene_variant	MODIFIER	Tmem260	ENSMUSG00000036339	Transcript	ENSMUST00000228697	protein_coding	rs31093505
14:48511581-48511581	intron_variant	MODIFIER	Tmem260	ENSMUSG00000036339	Transcript	ENSMUST00000111735	protein_coding	rs48453383
14:48511581-48511581	intron_variant	MODIFIER	Tmem260	ENSMUSG00000036339	Transcript	ENSMUST00000124720	protein_coding	rs48453383
14:48511581-48511581	intron_variant,NMD_transcript_v	MODIFIER	Tmem260	ENSMUSG00000036339	Transcript	ENSMUST00000153765	nonsense_mediated_decay	rs48453383
14:48511581-48511581	downstream_gene_variant	MODIFIER	Tmem260	ENSMUSG00000036339	Transcript	ENSMUST00000156480	retained_intron	rs48453383
14:48511581-48511581	downstream_gene_variant	MODIFIER	Tmem260	ENSMUSG00000036339	Transcript	ENSMUST00000226344	retained_intron	rs48453383
14:48511581-48511581	intron_variant	MODIFIER	Tmem260	ENSMUSG00000036339	Transcript	ENSMUST00000226422	protein_coding	rs48453383
14:48511581-48511581	intron_variant	MODIFIER	Tmem260	ENSMUSG00000036339	Transcript	ENSMUST00000227440	protein_coding	rs48453383
14:49014430-49014430	downstream_gene_variant	MODIFIER	Exoc5	ENSMUSG00000061244	Transcript	ENSMUST00000159651	retained_intron	rs30139075
14:49014430-49014430	downstream_gene_variant	MODIFIER	Exoc5	ENSMUSG00000061244	Transcript	ENSMUST00000160453	retained_intron	rs30139075
14:49014430-49014430	downstream_gene_variant	MODIFIER	Exoc5	ENSMUSG00000061244	Transcript	ENSMUST00000160833	retained_intron	rs30139075
14:49014430-49014430	intron_variant	MODIFIER	Exoc5	ENSMUSG00000061244	Transcript	ENSMUST00000161504	protein_coding	rs30139075
14:49014430-49014430	intron_variant	MODIFIER	Exoc5	ENSMUSG00000061244	Transcript	ENSMUST00000162175	protein_coding	rs30139075
14:49081637-49081637	intron_variant	MODIFIER	Ap5m1	ENSMUSG00000036291	Transcript	ENSMUST00000037473	protein_coding	rs48053724
14:49081637-49081637	upstream_gene_variant	MODIFIER	Ap5m1	ENSMUSG00000036291	Transcript	ENSMUST00000226695	protein_coding	rs48053724
14:49081637-49081637	intron_variant	MODIFIER	Ap5m1	ENSMUSG00000036291	Transcript	ENSMUST00000227066	protein_coding	rs48053724
14:49081637-49081637	intron_variant,NMD_transcript_v	MODIFIER	Ap5m1	ENSMUSG00000036291	Transcript	ENSMUST00000227410	nonsense_mediated_decay	rs48053724
14:49081637-49081637	3_prime_UTR_variant	MODIFIER	Ap5m1	ENSMUSG00000036291	Transcript	ENSMUST00000227431	protein_coding	rs48053724
14:49081637-49081637	intron_variant,NMD_transcript_v	MODIFIER	Ap5m1	ENSMUSG00000036291	Transcript	ENSMUST00000227608	nonsense_mediated_decay	rs48053724
14:49081637-49081637	downstream_gene_variant	MODIFIER	Ap5m1	ENSMUSG00000036291	Transcript	ENSMUST00000227991	protein_coding	rs48053724
14:49081637-49081637	downstream_gene_variant	MODIFIER	Ap5m1	ENSMUSG00000036291	Transcript	ENSMUST00000228238	protein_coding	rs48053724
14:49081920-49081920	intron_variant	MODIFIER	Ap5m1	ENSMUSG00000036291	Transcript	ENSMUST00000037473	protein_coding	rs49729908
14:49081920-49081920	upstream_gene_variant	MODIFIER	Ap5m1	ENSMUSG00000036291	Transcript	ENSMUST00000226695	protein_coding	rs49729908
14:49081920-49081920	intron_variant	MODIFIER	Ap5m1	ENSMUSG00000036291	Transcript	ENSMUST00000227066	protein_coding	rs49729908
14:49081920-49081920	intron_variant,NMD_transcript_v	MODIFIER	Ap5m1	ENSMUSG00000036291	Transcript	ENSMUST00000227410	nonsense_mediated_decay	rs49729908
14:49081920-49081920	3_prime_UTR_variant	MODIFIER	Ap5m1	ENSMUSG00000036291	Transcript	ENSMUST00000227431	protein_coding	rs49729908
14:49081920-49081920	intron_variant,NMD_transcript_v	MODIFIER	Ap5m1	ENSMUSG00000036291	Transcript	ENSMUST00000227608	nonsense_mediated_decay	rs49729908
14:49081920-49081920	downstream_gene_variant	MODIFIER	Ap5m1	ENSMUSG00000036291	Transcript	ENSMUST00000227991	protein_coding	rs49729908
14:49173664-49173664	intron_variant	MODIFIER	Naa30	ENSMUSG00000036282	Transcript	ENSMUST00000037362	protein_coding	rs48797461
14:49173664-49173664	upstream_gene_variant	MODIFIER	Naa30	ENSMUSG00000036282	Transcript	ENSMUST00000134984	retained_intron	rs48797461
14:49173664-49173664	intron_variant	MODIFIER	Naa30	ENSMUSG00000036282	Transcript	ENSMUST00000136995	protein_coding	rs48797461
14:49173664-49173664	upstream_gene_variant	MODIFIER	Naa30	ENSMUSG00000036282	Transcript	ENSMUST00000138478	retained_intron	rs48797461
14:49173664-49173664	intron_variant	MODIFIER	Naa30	ENSMUSG00000036282	Transcript	ENSMUST00000153488	protein_coding	rs48797461
14:49173664-49173664	regulatory_region_variant	MODIFIER	-	-	RegulatoryFeature	ENSMUSR00000087101	promoter	rs48797461
14:49173716-49173716	intron_variant	MODIFIER	Naa30	ENSMUSG00000036282	Transcript	ENSMUST00000037362	protein_coding	rs50393618
14:49173716-49173716	upstream_gene_variant	MODIFIER	Naa30	ENSMUSG00000036282	Transcript	ENSMUST00000134984	retained_intron	rs50393618
14:49173716-49173716	intron_variant	MODIFIER	Naa30	ENSMUSG00000036282	Transcript	ENSMUST00000136995	protein_coding	rs50393618
14:49173716-49173716	upstream_gene_variant	MODIFIER	Naa30	ENSMUSG00000036282	Transcript	ENSMUST00000138478	retained_intron	rs50393618
14:49173716-49173716	intron_variant	MODIFIER	Naa30	ENSMUSG00000036282	Transcript	ENSMUST00000153488	protein_coding	rs50393618
14:49173716-49173716	regulatory_region_variant	MODIFIER	-	-	RegulatoryFeature	ENSMUSR00000087101	promoter	rs50393618
14:49174669-49174669	intron_variant	MODIFIER	Naa30	ENSMUSG00000036282	Transcript	ENSMUST00000037362	protein_coding	rs30390823
14:49174669-49174669	upstream_gene_variant	MODIFIER	Naa30	ENSMUSG00000036282	Transcript	ENSMUST00000134984	retained_intron	rs30390823
14:49174669-49174669	intron_variant	MODIFIER	Naa30	ENSMUSG00000036282	Transcript	ENSMUST00000136995	protein_coding	rs30390823
14:49174669-49174669	upstream_gene_variant	MODIFIER	Naa30	ENSMUSG00000036282	Transcript	ENSMUST00000138478	retained_intron	rs30390823
14:49174669-49174669	intron_variant	MODIFIER	Naa30	ENSMUSG00000036282	Transcript	ENSMUST00000153488	protein_coding	rs30390823
14:49176189-49176189	intron_variant	MODIFIER	Naa30	ENSMUSG00000036282	Transcript	ENSMUST00000037362	protein_coding	rs30941667
14:49176189-49176189	upstream_gene_variant	MODIFIER	Naa30	ENSMUSG00000036282	Transcript	ENSMUST00000134984	retained_intron	rs30941667
14:49176189-49176189	intron_variant	MODIFIER	Naa30	ENSMUSG00000036282	Transcript	ENSMUST00000136995	protein_coding	rs30941667
14:49176189-49176189	upstream_gene_variant	MODIFIER	Naa30	ENSMUSG00000036282	Transcript	ENSMUST00000138478	retained_intron	rs30941667
14:49176189-49176189	intron_variant	MODIFIER	Naa30	ENSMUSG00000036282	Transcript	ENSMUST00000153488	protein_coding	rs30941667
14:50774017-50774017	intron_variant	MODIFIER	Ttc5	ENSMUSG00000006288	Transcript	ENSMUST00000006451	protein_coding	rs50702926
14:50774017-50774017	downstream_gene_variant	MODIFIER	Gm10916	ENSMUSG00000078125	Transcript	ENSMUST00000080330	processed_pseudogene	rs50702926
14:50774017-50774017	downstream_gene_variant	MODIFIER	Ttc5	ENSMUSG00000006288	Transcript	ENSMUST00000226768	protein_coding	rs50702926
14:50774017-50774017	intron_variant,non_coding_trans	MODIFIER	Ttc5	ENSMUSG00000006288	Transcript	ENSMUST00000227327	retained_intron	rs50702926
14:50774017-50774017	upstream_gene_variant	MODIFIER	Ttc5	ENSMUSG00000006288	Transcript	ENSMUST00000227751	nonsense_mediated_decay	rs50702926
14:50774017-50774017	downstream_gene_variant	MODIFIER	Ttc5	ENSMUSG00000006288	Transcript	ENSMUST00000227934	retained_intron	rs50702926
14:50774017-50774017	intron_variant,NMD_transcript_v	MODIFIER	Ttc5	ENSMUSG00000006288	Transcript	ENSMUST00000228440	nonsense_mediated_decay	rs50702926
14:50818433-50818433	intron_variant	MODIFIER	Parp2	ENSMUSG00000036023	Transcript	ENSMUST00000036126	protein_coding	rs36805892

14:50818433-50818433	downstream_gene_variant	MODIFIER	Parp2	ENSMUSG00000036023	Transcript	ENSMUST00000226880	retained_intron	rs36805892
14:50818433-50818433	intron_variant,NMD_transcript_v	MODIFIER	Parp2	ENSMUSG00000036023	Transcript	ENSMUST00000227810	nonsense_mediated_decay	rs36805892
14:50818433-50818433	upstream_gene_variant	MODIFIER	Parp2	ENSMUSG00000036023	Transcript	ENSMUST00000228624	retained_intron	rs36805892
14:50818433-50818433	intron_variant,non_coding_trans	MODIFIER	Parp2	ENSMUSG00000036023	Transcript	ENSMUST00000228672	retained_intron	rs36805892
14:50818433-50818433	upstream_gene_variant	MODIFIER	Parp2	ENSMUSG00000036023	Transcript	ENSMUST00000228833	retained_intron	rs36805892
14:50818433-50818433	upstream_gene_variant	MODIFIER	Parp2	ENSMUSG00000036023	Transcript	ENSMUST00000228872	retained_intron	rs36805892
14:50818851-50818851	intron_variant	MODIFIER	Parp2	ENSMUSG00000036023	Transcript	ENSMUST00000036126	protein_coding	rs36588050
14:50818851-50818851	downstream_gene_variant	MODIFIER	Parp2	ENSMUSG00000036023	Transcript	ENSMUST00000226880	retained_intron	rs36588050
14:50818851-50818851	intron_variant,NMD_transcript_v	MODIFIER	Parp2	ENSMUSG00000036023	Transcript	ENSMUST00000227810	nonsense_mediated_decay	rs36588050
14:50818851-50818851	upstream_gene_variant	MODIFIER	Parp2	ENSMUSG00000036023	Transcript	ENSMUST00000228624	retained_intron	rs36588050
14:50818851-50818851	intron_variant,non_coding_trans	MODIFIER	Parp2	ENSMUSG00000036023	Transcript	ENSMUST00000228672	retained_intron	rs36588050
14:50818851-50818851	upstream_gene_variant	MODIFIER	Parp2	ENSMUSG00000036023	Transcript	ENSMUST00000228833	retained_intron	rs36588050
14:50818851-50818851	upstream_gene_variant	MODIFIER	Parp2	ENSMUSG00000036023	Transcript	ENSMUST00000228872	retained_intron	rs36588050
14:50819902-50819902	downstream_gene_variant	MODIFIER	Tep1	ENSMUSG00000006281	Transcript	ENSMUST00000006444	protein_coding	rs30602072
14:50819902-50819902	intron_variant	MODIFIER	Parp2	ENSMUSG00000036023	Transcript	ENSMUST00000036126	protein_coding	rs30602072
14:50819902-50819902	downstream_gene_variant	MODIFIER	Tep1	ENSMUSG00000006281	Transcript	ENSMUST00000226222	protein_coding	rs30602072
14:50819902-50819902	downstream_gene_variant	MODIFIER	Tep1	ENSMUSG00000006281	Transcript	ENSMUST00000226430	nonsense_mediated_decay	rs30602072
14:50819902-50819902	downstream_gene_variant	MODIFIER	Parp2	ENSMUSG00000036023	Transcript	ENSMUST00000226880	retained_intron	rs30602072
14:50819902-50819902	downstream_gene_variant	MODIFIER	Parp2	ENSMUSG00000036023	Transcript	ENSMUST00000227810	nonsense_mediated_decay	rs30602072
14:50819902-50819902	downstream_gene_variant	MODIFIER	Parp2	ENSMUSG00000036023	Transcript	ENSMUST00000228624	retained_intron	rs30602072
14:50819902-50819902	intron_variant,non_coding_trans	MODIFIER	Parp2	ENSMUSG00000036023	Transcript	ENSMUST00000228672	retained_intron	rs30602072
14:50819902-50819902	downstream_gene_variant	MODIFIER	Parp2	ENSMUSG00000036023	Transcript	ENSMUST00000228833	retained_intron	rs30602072
14:50819902-50819902	upstream_gene_variant	MODIFIER	Parp2	ENSMUSG00000036023	Transcript	ENSMUST00000228872	retained_intron	rs30602072
14:50834793-50834793	intron_variant	MODIFIER	Tep1	ENSMUSG00000006281	Transcript	ENSMUST00000006444	protein_coding	rs52528866
14:50834793-50834793	intron_variant,NMD_transcript_v	MODIFIER	Tep1	ENSMUSG00000006281	Transcript	ENSMUST00000226430	nonsense_mediated_decay	rs52528866
14:50834793-50834793	intron_variant,non_coding_trans	MODIFIER	Tep1	ENSMUSG00000006281	Transcript	ENSMUST00000227193	processed_transcript	rs52528866
14:50834793-50834793	upstream_gene_variant	MODIFIER	Tep1	ENSMUSG00000006281	Transcript	ENSMUST00000227228	retained_intron	rs52528866
14:50834793-50834793	upstream_gene_variant	MODIFIER	Tep1	ENSMUSG00000006281	Transcript	ENSMUST00000227526	nonsense_mediated_decay	rs52528866
14:50834793-50834793	upstream_gene_variant	MODIFIER	Tep1	ENSMUSG00000006281	Transcript	ENSMUST00000228562	retained_intron	rs52528866
14:50834793-50834793	regulatory_region_variant	MODIFIER	-	-	RegulatoryFeature	ENSMUSR00000560348	promoter_flanking_region	rs52528866
14:50872593-50872593	upstream_gene_variant	MODIFIER	Tep1	ENSMUSG00000006281	Transcript	ENSMUST00000006444	protein_coding	rs31043266
14:50872593-50872593	intron_variant,non_coding_trans	MODIFIER	Gm26782	ENSMUSG00000097431	Transcript	ENSMUST00000181482	antisense	rs31043266
14:50872593-50872593	intron_variant,non_coding_trans	MODIFIER	Gm26782	ENSMUSG00000097431	Transcript	ENSMUST00000181697	antisense	rs31043266
14:50872593-50872593	intron_variant,non_coding_trans	MODIFIER	Gm26782	ENSMUSG00000097431	Transcript	ENSMUST00000227103	antisense	rs31043266
14:50872593-50872593	non_coding_transcript_exon_va	MODIFIER	Gm26782	ENSMUSG00000097431	Transcript	ENSMUST00000227207	antisense	rs31043266
14:50872593-50872593	intron_variant,non_coding_trans	MODIFIER	Gm26782	ENSMUSG00000097431	Transcript	ENSMUST00000227351	antisense	rs31043266
14:50872593-50872593	intron_variant,non_coding_trans	MODIFIER	Gm26782	ENSMUSG00000097431	Transcript	ENSMUST00000228078	antisense	rs31043266
14:50872593-50872593	downstream_gene_variant	MODIFIER	Gm26782	ENSMUSG00000097431	Transcript	ENSMUST00000228254	antisense	rs31043266
14:50889348-50889348	downstream_gene_variant	MODIFIER	Klhl33	ENSMUSG00000090799	Transcript	ENSMUST00000164415	protein_coding	rs30772827
14:50889348-50889348	3_prime_UTR_variant	MODIFIER	Klhl33	ENSMUSG00000090799	Transcript	ENSMUST00000227271	protein_coding	rs30772827
14:50889350-50889350	downstream_gene_variant	MODIFIER	Klhl33	ENSMUSG00000090799	Transcript	ENSMUST00000164415	protein_coding	rs30830784
14:50889350-50889350	3_prime_UTR_variant	MODIFIER	Klhl33	ENSMUSG00000090799	Transcript	ENSMUST00000227271	protein_coding	rs30830784
14:50912689-50912689	downstream_gene_variant	MODIFIER	Osgep	ENSMUSG00000006289	Transcript	ENSMUST00000006452	nonsense_mediated_decay	rs49562514
14:50912689-50912689	downstream_gene_variant	MODIFIER	Osgep	ENSMUSG00000006289	Transcript	ENSMUST00000159292	protein_coding	rs49562514
14:50912689-50912689	downstream_gene_variant	MODIFIER	Osgep	ENSMUSG00000006289	Transcript	ENSMUST00000160375	protein_coding	rs49562514
14:50912689-50912689	intron_variant,NMD_transcript_v	MODIFIER	Osgep	ENSMUSG00000006289	Transcript	ENSMUST00000160393	nonsense_mediated_decay	rs49562514
14:50912689-50912689	downstream_gene_variant	MODIFIER	Osgep	ENSMUSG00000006289	Transcript	ENSMUST00000160464	retained_intron	rs49562514
14:50912689-50912689	downstream_gene_variant	MODIFIER	Osgep	ENSMUSG00000006289	Transcript	ENSMUST00000160890	nonsense_mediated_decay	rs49562514
14:50912689-50912689	downstream_gene_variant	MODIFIER	Osgep	ENSMUSG00000006289	Transcript	ENSMUST00000162177	protein_coding	rs49562514
14:50912689-50912689	downstream_gene_variant	MODIFIER	Osgep	ENSMUSG00000006289	Transcript	ENSMUST00000162850	retained_intron	rs49562514
14:50914661-50914661	downstream_gene_variant	MODIFIER	Osgep	ENSMUSG00000006289	Transcript	ENSMUST00000006452	nonsense_mediated_decay	rs49078947
14:50914661-50914661	upstream_gene_variant	MODIFIER	Gm24689	ENSMUSG00000088385	Transcript	ENSMUST00000157760	snRNA	rs49078947
14:50914661-50914661	3_prime_UTR_variant	MODIFIER	Osgep	ENSMUSG00000006289	Transcript	ENSMUST00000159292	protein_coding	rs49078947
14:50914661-50914661	downstream_gene_variant	MODIFIER	Osgep	ENSMUSG00000006289	Transcript	ENSMUST00000160375	protein_coding	rs49078947
14:50914661-50914661	intron_variant,NMD_transcript_v	MODIFIER	Osgep	ENSMUSG00000006289	Transcript	ENSMUST00000160393	nonsense_mediated_decay	rs49078947
14:50914661-50914661	downstream_gene_variant	MODIFIER	Osgep	ENSMUSG00000006289	Transcript	ENSMUST00000160464	retained_intron	rs49078947
14:50914661-50914661	downstream_gene_variant	MODIFIER	Osgep	ENSMUSG00000006289	Transcript	ENSMUST00000160890	nonsense_mediated_decay	rs49078947
14:50914661-50914661	downstream_gene_variant	MODIFIER	Osgep	ENSMUSG00000006289	Transcript	ENSMUST00000162177	protein_coding	rs49078947
14:50914661-50914661	downstream_gene_variant	MODIFIER	Osgep	ENSMUSG00000006289	Transcript	ENSMUST00000162850	retained_intron	rs49078947
14:50920311-50920311	intron_variant,NMD_transcript_v	MODIFIER	Osgep	ENSMUSG00000006289	Transcript	ENSMUST00000006452	nonsense_mediated_decay	rs31295874
14:50920311-50920311	upstream_gene_variant	MODIFIER	Apex1	ENSMUSG00000035960	Transcript	ENSMUST00000049411	protein_coding	rs31295874
14:50920311-50920311	upstream_gene_variant	MODIFIER	Apex1	ENSMUSG00000035960	Transcript	ENSMUST00000128395	protein_coding	rs31295874
14:50920311-50920311	upstream_gene_variant	MODIFIER	Apex1	ENSMUSG00000035960	Transcript	ENSMUST00000136753	protein_coding	rs31295874
14:50920311-50920311	upstream_gene_variant	MODIFIER	Apex1	ENSMUSG00000035960	Transcript	ENSMUST00000154288	protein_coding	rs31295874
14:50920311-50920311	downstream_gene_variant	MODIFIER	Gm24689	ENSMUSG00000088385	Transcript	ENSMUST00000157760	snRNA	rs31295874
14:50920311-50920311	intron_variant	MODIFIER	Osgep	ENSMUSG00000006289	Transcript	ENSMUST00000159292	protein_coding	rs31295874
14:50920311-50920311	intron_variant	MODIFIER	Osgep	ENSMUSG00000006289	Transcript	ENSMUST00000160375	protein_coding	rs31295874
14:50920311-50920311	intron_variant,NMD_transcript_v	MODIFIER	Osgep	ENSMUSG00000006289	Transcript	ENSMUST00000160393	nonsense_mediated_decay	rs31295874
14:50920311-50920311	intron_variant,non_coding_trans	MODIFIER	Osgep	ENSMUSG00000006289	Transcript	ENSMUST00000160464	retained_intron	rs31295874

14:50920311-50920311	intron_variant,NMD_transcript_v	MODIFIER	Osgep	ENSMUSG00000006289	Transcript	ENSMUST00000160890	nonsense_mediated_decay	rs31295874
14:50920311-50920311	intron_variant	MODIFIER	Osgep	ENSMUSG00000006289	Transcript	ENSMUST00000162177	protein_coding	rs31295874
14:50920311-50920311	upstream_gene_variant	MODIFIER	Osgep	ENSMUSG00000006289	Transcript	ENSMUST00000162850	retained_intron	rs31295874
14:50920634-50920634	intron_variant,NMD_transcript_v	MODIFIER	Osgep	ENSMUSG00000006289	Transcript	ENSMUST00000006452	nonsense_mediated_decay	rs46206259
14:50920634-50920634	upstream_gene_variant	MODIFIER	Apex1	ENSMUSG00000035960	Transcript	ENSMUST00000049411	protein_coding	rs46206259
14:50920634-50920634	upstream_gene_variant	MODIFIER	Apex1	ENSMUSG00000035960	Transcript	ENSMUST00000128395	protein_coding	rs46206259
14:50920634-50920634	upstream_gene_variant	MODIFIER	Apex1	ENSMUSG00000035960	Transcript	ENSMUST00000136753	protein_coding	rs46206259
14:50920634-50920634	upstream_gene_variant	MODIFIER	Apex1	ENSMUSG00000035960	Transcript	ENSMUST00000154288	protein_coding	rs46206259
14:50920634-50920634	downstream_gene_variant	MODIFIER	Gm24689	ENSMUSG00000088385	Transcript	ENSMUST00000157760	snRNA	rs46206259
14:50920634-50920634	intron_variant	MODIFIER	Osgep	ENSMUSG00000006289	Transcript	ENSMUST00000159292	protein_coding	rs46206259
14:50920634-50920634	intron_variant	MODIFIER	Osgep	ENSMUSG00000006289	Transcript	ENSMUST00000160375	protein_coding	rs46206259
14:50920634-50920634	intron_variant,NMD_transcript_v	MODIFIER	Osgep	ENSMUSG00000006289	Transcript	ENSMUST00000160393	nonsense_mediated_decay	rs46206259
14:50920634-50920634	intron_variant,non_coding_trans	MODIFIER	Osgep	ENSMUSG00000006289	Transcript	ENSMUST00000160464	retained_intron	rs46206259
14:50920634-50920634	intron_variant,NMD_transcript_v	MODIFIER	Osgep	ENSMUSG00000006289	Transcript	ENSMUST00000160890	nonsense_mediated_decay	rs46206259
14:50920634-50920634	intron_variant	MODIFIER	Osgep	ENSMUSG00000006289	Transcript	ENSMUST00000162177	protein_coding	rs46206259
14:50920634-50920634	upstream_gene_variant	MODIFIER	Osgep	ENSMUSG00000006289	Transcript	ENSMUST00000162850	retained_intron	rs46206259
14:50920793-50920793	intron_variant,NMD_transcript_v	MODIFIER	Osgep	ENSMUSG00000006289	Transcript	ENSMUST00000006452	nonsense_mediated_decay	rs46823131
14:50920793-50920793	upstream_gene_variant	MODIFIER	Apex1	ENSMUSG00000035960	Transcript	ENSMUST00000049411	protein_coding	rs46823131
14:50920793-50920793	upstream_gene_variant	MODIFIER	Apex1	ENSMUSG00000035960	Transcript	ENSMUST00000128395	protein_coding	rs46823131
14:50920793-50920793	upstream_gene_variant	MODIFIER	Apex1	ENSMUSG00000035960	Transcript	ENSMUST00000136753	protein_coding	rs46823131
14:50920793-50920793	upstream_gene_variant	MODIFIER	Apex1	ENSMUSG00000035960	Transcript	ENSMUST00000154288	protein_coding	rs46823131
14:50920793-50920793	downstream_gene_variant	MODIFIER	Gm24689	ENSMUSG00000088385	Transcript	ENSMUST00000157760	snRNA	rs46823131
14:50920793-50920793	intron_variant	MODIFIER	Osgep	ENSMUSG00000006289	Transcript	ENSMUST00000159292	protein_coding	rs46823131
14:50920793-50920793	intron_variant	MODIFIER	Osgep	ENSMUSG00000006289	Transcript	ENSMUST00000160375	protein_coding	rs46823131
14:50920793-50920793	intron_variant,NMD_transcript_v	MODIFIER	Osgep	ENSMUSG00000006289	Transcript	ENSMUST00000160393	nonsense_mediated_decay	rs46823131
14:50920793-50920793	intron_variant,non_coding_trans	MODIFIER	Osgep	ENSMUSG00000006289	Transcript	ENSMUST00000160464	retained_intron	rs46823131
14:50920793-50920793	intron_variant,NMD_transcript_v	MODIFIER	Osgep	ENSMUSG00000006289	Transcript	ENSMUST00000160890	nonsense_mediated_decay	rs46823131
14:50920793-50920793	intron_variant	MODIFIER	Osgep	ENSMUSG00000006289	Transcript	ENSMUST00000162177	protein_coding	rs46823131
14:50920793-50920793	upstream_gene_variant	MODIFIER	Osgep	ENSMUSG00000006289	Transcript	ENSMUST00000162850	retained_intron	rs46823131
14:50923664-50923664	intron_variant,NMD_transcript_v	MODIFIER	Osgep	ENSMUSG00000006289	Transcript	ENSMUST00000006452	nonsense_mediated_decay	rs51535979
14:50923664-50923664	downstream_gene_variant	MODIFIER	Pip4p1	ENSMUSG00000035953	Transcript	ENSMUST00000049312	protein_coding	rs51535979
14:50923664-50923664	upstream_gene_variant	MODIFIER	Apex1	ENSMUSG00000035960	Transcript	ENSMUST00000049411	protein_coding	rs51535979
14:50923664-50923664	upstream_gene_variant	MODIFIER	Apex1	ENSMUSG00000035960	Transcript	ENSMUST00000128395	protein_coding	rs51535979
14:50923664-50923664	upstream_gene_variant	MODIFIER	Apex1	ENSMUSG00000035960	Transcript	ENSMUST00000136753	protein_coding	rs51535979
14:50923664-50923664	upstream_gene_variant	MODIFIER	Apex1	ENSMUSG00000035960	Transcript	ENSMUST00000154288	protein_coding	rs51535979
14:50923664-50923664	intron_variant	MODIFIER	Osgep	ENSMUSG00000006289	Transcript	ENSMUST00000159292	protein_coding	rs51535979
14:50923664-50923664	downstream_gene_variant	MODIFIER	Pip4p1	ENSMUSG00000035953	Transcript	ENSMUST00000159745	retained_intron	rs51535979
14:50923664-50923664	intron_variant	MODIFIER	Osgep	ENSMUSG00000006289	Transcript	ENSMUST00000160375	protein_coding	rs51535979
14:50923664-50923664	intron_variant,NMD_transcript_v	MODIFIER	Osgep	ENSMUSG00000006289	Transcript	ENSMUST00000160393	nonsense_mediated_decay	rs51535979
14:50923664-50923664	intron_variant,non_coding_trans	MODIFIER	Osgep	ENSMUSG00000006289	Transcript	ENSMUST00000160464	retained_intron	rs51535979
14:50923664-50923664	downstream_gene_variant	MODIFIER	Pip4p1	ENSMUSG00000035953	Transcript	ENSMUST00000160835	protein_coding	rs51535979
14:50923664-50923664	intron_variant,NMD_transcript_v	MODIFIER	Osgep	ENSMUSG00000006289	Transcript	ENSMUST00000160890	nonsense_mediated_decay	rs51535979
14:50923664-50923664	downstream_gene_variant	MODIFIER	Pip4p1	ENSMUSG00000035953	Transcript	ENSMUST00000161166	protein_coding	rs51535979
14:50923664-50923664	downstream_gene_variant	MODIFIER	Pip4p1	ENSMUSG00000035953	Transcript	ENSMUST00000161669	protein_coding	rs51535979
14:50923664-50923664	intron_variant	MODIFIER	Osgep	ENSMUSG00000006289	Transcript	ENSMUST00000162177	protein_coding	rs51535979
14:50923664-50923664	downstream_gene_variant	MODIFIER	Pip4p1	ENSMUSG00000035953	Transcript	ENSMUST00000162957	protein_coding	rs51535979
14:50923664-50923664	regulatory_region_variant	MODIFIER	-	-	RegulatoryFeature	ENSMUSR00000087191	promoter	rs51535979
14:50930449-50930449	intron_variant	MODIFIER	Pip4p1	ENSMUSG00000035953	Transcript	ENSMUST00000049312	protein_coding	rs30663181
14:50930449-50930449	downstream_gene_variant	MODIFIER	Apex1	ENSMUSG00000035960	Transcript	ENSMUST00000049411	protein_coding	rs30663181
14:50930449-50930449	downstream_gene_variant	MODIFIER	Apex1	ENSMUSG00000035960	Transcript	ENSMUST00000128395	protein_coding	rs30663181
14:50930449-50930449	downstream_gene_variant	MODIFIER	Apex1	ENSMUSG00000035960	Transcript	ENSMUST00000136753	protein_coding	rs30663181
14:50930449-50930449	downstream_gene_variant	MODIFIER	Apex1	ENSMUSG00000035960	Transcript	ENSMUST00000154288	protein_coding	rs30663181
14:50930449-50930449	upstream_gene_variant	MODIFIER	Pip4p1	ENSMUSG00000035953	Transcript	ENSMUST00000159047	retained_intron	rs30663181
14:50930449-50930449	intron_variant,non_coding_trans	MODIFIER	Pip4p1	ENSMUSG00000035953	Transcript	ENSMUST00000159745	retained_intron	rs30663181
14:50930449-50930449	intron_variant	MODIFIER	Pip4p1	ENSMUSG00000035953	Transcript	ENSMUST00000160538	protein_coding	rs30663181
14:50930449-50930449	intron_variant	MODIFIER	Pip4p1	ENSMUSG00000035953	Transcript	ENSMUST00000160835	protein_coding	rs30663181
14:50930449-50930449	intron_variant	MODIFIER	Pip4p1	ENSMUSG00000035953	Transcript	ENSMUST00000161166	protein_coding	rs30663181
14:50930449-50930449	upstream_gene_variant	MODIFIER	Pip4p1	ENSMUSG00000035953	Transcript	ENSMUST00000161669	protein_coding	rs30663181
14:50930449-50930449	intron_variant,non_coding_trans	MODIFIER	Pip4p1	ENSMUSG00000035953	Transcript	ENSMUST00000162935	retained_intron	rs30663181
14:50930449-50930449	intron_variant	MODIFIER	Pip4p1	ENSMUSG00000035953	Transcript	ENSMUST00000162957	protein_coding	rs30663181
14:50930449-50930449	intron_variant,non_coding_trans	MODIFIER	Pip4p1	ENSMUSG00000035953	Transcript	ENSMUST00000163043	retained_intron	rs30663181
14:50930449-50930449	intron_variant,non_coding_trans	MODIFIER	Pip4p1	ENSMUSG00000035953	Transcript	ENSMUST00000163088	retained_intron	rs30663181
14:50930449-50930449	upstream_gene_variant	MODIFIER	Pnp	ENSMUSG00000115338	Transcript	ENSMUST00000226871	protein_coding	rs30663181
14:50930449-50930449	regulatory_region_variant	MODIFIER	-	-	RegulatoryFeature	ENSMUSR00000087192	promoter	rs30663181
14:50930469-50930469	intron_variant	MODIFIER	Pip4p1	ENSMUSG00000035953	Transcript	ENSMUST00000049312	protein_coding	rs3673597
14:50930469-50930469	downstream_gene_variant	MODIFIER	Apex1	ENSMUSG00000035960	Transcript	ENSMUST00000049411	protein_coding	rs3673597
14:50930469-50930469	downstream_gene_variant	MODIFIER	Apex1	ENSMUSG00000035960	Transcript	ENSMUST00000128395	protein_coding	rs3673597
14:50930469-50930469	downstream_gene_variant	MODIFIER	Apex1	ENSMUSG00000035960	Transcript	ENSMUST00000136753	protein_coding	rs3673597
14:50930469-50930469	downstream_gene_variant	MODIFIER	Apex1	ENSMUSG00000035960	Transcript	ENSMUST00000154288	protein_coding	rs3673597

14:50930469-50930469	upstream_gene_variant	MODIFIER	Pip4p1	ENSMUSG00000035953	Transcript	ENSMUST00000159047	retained_intron	rs3673597
14:50930469-50930469	intron_variant,non_coding_trans	MODIFIER	Pip4p1	ENSMUSG00000035953	Transcript	ENSMUST00000159745	retained_intron	rs3673597
14:50930469-50930469	intron_variant	MODIFIER	Pip4p1	ENSMUSG00000035953	Transcript	ENSMUST00000160538	protein_coding	rs3673597
14:50930469-50930469	intron_variant	MODIFIER	Pip4p1	ENSMUSG00000035953	Transcript	ENSMUST00000160835	protein_coding	rs3673597
14:50930469-50930469	intron_variant	MODIFIER	Pip4p1	ENSMUSG00000035953	Transcript	ENSMUST00000161166	protein_coding	rs3673597
14:50930469-50930469	upstream_gene_variant	MODIFIER	Pip4p1	ENSMUSG00000035953	Transcript	ENSMUST00000161669	protein_coding	rs3673597
14:50930469-50930469	intron_variant,non_coding_trans	MODIFIER	Pip4p1	ENSMUSG00000035953	Transcript	ENSMUST00000162935	retained_intron	rs3673597
14:50930469-50930469	intron_variant	MODIFIER	Pip4p1	ENSMUSG00000035953	Transcript	ENSMUST00000162957	protein_coding	rs3673597
14:50930469-50930469	intron_variant,non_coding_trans	MODIFIER	Pip4p1	ENSMUSG00000035953	Transcript	ENSMUST00000163043	retained_intron	rs3673597
14:50930469-50930469	intron_variant,non_coding_trans	MODIFIER	Pip4p1	ENSMUSG00000035953	Transcript	ENSMUST00000163088	retained_intron	rs3673597
14:50930469-50930469	upstream_gene_variant	MODIFIER	Pnp	ENSMUSG00000115338	Transcript	ENSMUST00000226871	protein_coding	rs3673597
14:50930469-50930469	regulatory_region_variant	MODIFIER	-	-	RegulatoryFeature	ENSMUSR00000087192	promoter	rs3673597
14:50948729-50948729	intron_variant	MODIFIER	Pnp	ENSMUSG00000115338	Transcript	ENSMUST00000048615	protein_coding	rs45639627
14:50948729-50948729	intron_variant	MODIFIER	Pnp	ENSMUSG00000115338	Transcript	ENSMUST00000178092	protein_coding	rs45639627
14:50948729-50948729	downstream_gene_variant	MODIFIER	Gm38316	ENSMUSG00000103730	Transcript	ENSMUST00000194838	TEC	rs45639627
14:50948729-50948729	intron_variant	MODIFIER	Pnp	ENSMUSG00000115338	Transcript	ENSMUST00000226871	protein_coding	rs45639627
14:50948729-50948729	intron_variant	MODIFIER	Gm49342	ENSMUSG00000021871	Transcript	ENSMUST00000227052	protein_coding	rs45639627
14:50948729-50948729	intron_variant,non_coding_trans	MODIFIER	Pnp	ENSMUSG00000115338	Transcript	ENSMUST00000228631	processed_transcript	rs45639627
14:50948729-50948729	downstream_gene_variant	MODIFIER	Pnp	ENSMUSG00000115338	Transcript	ENSMUST00000228712	retained_intron	rs45639627
14:50948734-50948734	intron_variant	MODIFIER	Pnp	ENSMUSG00000115338	Transcript	ENSMUST00000048615	protein_coding	rs47494078
14:50948734-50948734	intron_variant	MODIFIER	Pnp	ENSMUSG00000115338	Transcript	ENSMUST00000178092	protein_coding	rs47494078
14:50948734-50948734	downstream_gene_variant	MODIFIER	Gm38316	ENSMUSG00000103730	Transcript	ENSMUST00000194838	TEC	rs47494078
14:50948734-50948734	intron_variant	MODIFIER	Pnp	ENSMUSG00000115338	Transcript	ENSMUST00000226871	protein_coding	rs47494078
14:50948734-50948734	intron_variant	MODIFIER	Gm49342	ENSMUSG00000021871	Transcript	ENSMUST00000227052	protein_coding	rs47494078
14:50948734-50948734	intron_variant,non_coding_trans	MODIFIER	Pnp	ENSMUSG00000115338	Transcript	ENSMUST00000228631	processed_transcript	rs47494078
14:50948734-50948734	downstream_gene_variant	MODIFIER	Pnp	ENSMUSG00000115338	Transcript	ENSMUST00000228712	retained_intron	rs47494078
14:51096436-51096436	intron_variant	MODIFIER	Rnase4	ENSMUSG00000021876	Transcript	ENSMUST00000022428	protein_coding	rs30894662
14:51096436-51096436	5_prime_UTR_variant	MODIFIER	Ang	ENSMUSG00000072115	Transcript	ENSMUST00000069011	protein_coding	rs30894662
14:51096436-51096436	5_prime_UTR_variant	MODIFIER	Rnase4	ENSMUSG00000021876	Transcript	ENSMUST00000169895	protein_coding	rs30894662
14:51096436-51096436	intron_variant	MODIFIER	Ang	ENSMUSG00000072115	Transcript	ENSMUST00000171688	protein_coding	rs30894662
14:51096436-51096436	regulatory_region_variant	MODIFIER	-	-	RegulatoryFeature	ENSMUSR00000347558	promoter	rs30894662
14:51101915-51101915	intron_variant	MODIFIER	Rnase4	ENSMUSG00000021876	Transcript	ENSMUST00000022428	protein_coding	rs3023408
14:51101915-51101915	3_prime_UTR_variant	MODIFIER	Ang	ENSMUSG00000072115	Transcript	ENSMUST00000069011	protein_coding	rs3023408
14:51101915-51101915	intron_variant	MODIFIER	Rnase4	ENSMUSG00000021876	Transcript	ENSMUST00000169895	protein_coding	rs3023408
14:51101915-51101915	3_prime_UTR_variant	MODIFIER	Ang	ENSMUSG00000072115	Transcript	ENSMUST00000171688	protein_coding	rs3023408
14:52016344-52016344	intron_variant	MODIFIER	Zfp219	ENSMUSG00000049295	Transcript	ENSMUST00000067549	protein_coding	rs212743314
14:52016344-52016344	upstream_gene_variant	MODIFIER	Tmem253	ENSMUSG00000072571	Transcript	ENSMUST00000100638	protein_coding	rs212743314
14:52016344-52016344	intron_variant	MODIFIER	Zfp219	ENSMUSG00000049295	Transcript	ENSMUST00000166169	protein_coding	rs212743314
14:52016344-52016344	intron_variant,non_coding_trans	MODIFIER	Tmem253	ENSMUSG00000072571	Transcript	ENSMUST00000226268	processed_transcript	rs212743314
14:52016344-52016344	upstream_gene_variant	MODIFIER	Zfp219	ENSMUSG00000049295	Transcript	ENSMUST00000226522	protein_coding	rs212743314
14:52016344-52016344	upstream_gene_variant	MODIFIER	Zfp219	ENSMUSG00000049295	Transcript	ENSMUST00000226527	protein_coding	rs212743314
14:52016344-52016344	intron_variant	MODIFIER	Zfp219	ENSMUSG00000049295	Transcript	ENSMUST00000226554	protein_coding	rs212743314
14:52016344-52016344	intron_variant	MODIFIER	Zfp219	ENSMUSG00000049295	Transcript	ENSMUST00000226605	protein_coding	rs212743314
14:52016344-52016344	upstream_gene_variant	MODIFIER	Gm49086	ENSMUSG00000115454	Transcript	ENSMUST00000226970	antisense	rs212743314
14:52016344-52016344	upstream_gene_variant	MODIFIER	Tmem253	ENSMUSG00000072571	Transcript	ENSMUST00000227295	protein_coding	rs212743314
14:52016344-52016344	intron_variant,non_coding_trans	MODIFIER	Zfp219	ENSMUSG00000049295	Transcript	ENSMUST00000227420	processed_transcript	rs212743314
14:52016344-52016344	upstream_gene_variant	MODIFIER	Zfp219	ENSMUSG00000049295	Transcript	ENSMUST00000228051	protein_coding	rs212743314
14:52016344-52016344	intron_variant	MODIFIER	Zfp219	ENSMUSG00000049295	Transcript	ENSMUST00000228162	protein_coding	rs212743314
14:52016344-52016344	upstream_gene_variant	MODIFIER	Tmem253	ENSMUSG00000072571	Transcript	ENSMUST00000228408	protein_coding	rs212743314
14:52016344-52016344	intron_variant	MODIFIER	Zfp219	ENSMUSG00000049295	Transcript	ENSMUST00000228580	protein_coding	rs212743314
14:52272390-52272390	intron_variant	MODIFIER	Rab2b	ENSMUSG00000022159	Transcript	ENSMUST00000022765	protein_coding	rs30398574
14:52272390-52272390	intron_variant	MODIFIER	Rab2b	ENSMUSG00000022159	Transcript	ENSMUST00000100631	protein_coding	rs30398574
14:52272390-52272390	upstream_gene_variant	MODIFIER	Gm23758	ENSMUSG00000088979	Transcript	ENSMUST00000158354	snoRNA	rs30398574
14:52272390-52272390	intron_variant	MODIFIER	Rab2b	ENSMUSG00000022159	Transcript	ENSMUST00000167116	protein_coding	rs30398574
14:52272390-52272390	intron_variant,NMD_transcript_v	MODIFIER	Rab2b	ENSMUSG00000022159	Transcript	ENSMUST00000172488	nonsense_mediated_decay	rs30398574
14:52272390-52272390	intron_variant,NMD_transcript_v	MODIFIER	Rab2b	ENSMUSG00000022159	Transcript	ENSMUST00000172634	nonsense_mediated_decay	rs30398574
14:52272390-52272390	intron_variant,non_coding_trans	MODIFIER	Rab2b	ENSMUSG00000022159	Transcript	ENSMUST00000173046	processed_transcript	rs30398574
14:52272390-52272390	intron_variant,NMD_transcript_v	MODIFIER	Rab2b	ENSMUSG00000022159	Transcript	ENSMUST00000174020	nonsense_mediated_decay	rs30398574
14:52272390-52272390	upstream_gene_variant	MODIFIER	Rab2b	ENSMUSG00000022159	Transcript	ENSMUST00000174585	retained_intron	rs30398574
14:52273706-52273706	intron_variant	MODIFIER	Rab2b	ENSMUSG00000022159	Transcript	ENSMUST00000022765	protein_coding	rs48351748
14:52273706-52273706	intron_variant	MODIFIER	Rab2b	ENSMUSG00000022159	Transcript	ENSMUST00000100631	protein_coding	rs48351748
14:52273706-52273706	upstream_gene_variant	MODIFIER	Gm23758	ENSMUSG00000088979	Transcript	ENSMUST00000158354	snoRNA	rs48351748
14:52273706-52273706	intron_variant	MODIFIER	Rab2b	ENSMUSG00000022159	Transcript	ENSMUST00000167116	protein_coding	rs48351748
14:52273706-52273706	intron_variant,NMD_transcript_v	MODIFIER	Rab2b	ENSMUSG00000022159	Transcript	ENSMUST00000172488	nonsense_mediated_decay	rs48351748
14:52273706-52273706	intron_variant,NMD_transcript_v	MODIFIER	Rab2b	ENSMUSG00000022159	Transcript	ENSMUST00000172634	nonsense_mediated_decay	rs48351748
14:52273706-52273706	intron_variant,non_coding_trans	MODIFIER	Rab2b	ENSMUSG00000022159	Transcript	ENSMUST00000173046	processed_transcript	rs48351748
14:52273706-52273706	intron_variant,NMD_transcript_v	MODIFIER	Rab2b	ENSMUSG00000022159	Transcript	ENSMUST00000174020	nonsense_mediated_decay	rs48351748
14:52273706-52273706	upstream_gene_variant	MODIFIER	Rab2b	ENSMUSG00000022159	Transcript	ENSMUST00000174585	retained_intron	rs48351748
14:52297251-52297251	downstream_gene_variant	MODIFIER	Tox4	ENSMUSG00000016831	Transcript	ENSMUST00000022766	protein_coding	rs48774859

14:52297251-52297251	intron_variant	MODIFIER	Mettl3	ENSMUSG00000022160	Transcript	ENSMUST00000022767	protein_coding	rs48774859
14:52297251-52297251	3_prime_UTR_variant,NMD_trar	MODIFIER	Mettl3	ENSMUSG00000022160	Transcript	ENSMUST00000122962	nonsense_mediated_decay	rs48774859
14:52297251-52297251	non_coding_transcript_exon_vai	MODIFIER	Mettl3	ENSMUSG00000022160	Transcript	ENSMUST00000127797	retained_intron	rs48774859
14:52297251-52297251	upstream_gene_variant	MODIFIER	Mettl3	ENSMUSG00000022160	Transcript	ENSMUST00000130550	retained_intron	rs48774859
14:52297251-52297251	downstream_gene_variant	MODIFIER	Mettl3	ENSMUSG00000022160	Transcript	ENSMUST00000145875	nonsense_mediated_decay	rs48774859
14:52297251-52297251	intron_variant,NMD_transcript_v	MODIFIER	Mettl3	ENSMUSG00000022160	Transcript	ENSMUST00000147768	nonsense_mediated_decay	rs48774859
14:52297251-52297251	downstream_gene_variant	MODIFIER	Tox4	ENSMUSG00000016831	Transcript	ENSMUST00000152493	retained_intron	rs48774859
14:52297251-52297251	intron_variant,non_coding_trans	MODIFIER	Mettl3	ENSMUSG00000022160	Transcript	ENSMUST00000156611	retained_intron	rs48774859
14:52297251-52297251	downstream_gene_variant	MODIFIER	Mettl3	ENSMUSG00000022160	Transcript	ENSMUST00000173138	nonsense_mediated_decay	rs48774859
14:52297251-52297251	upstream_gene_variant	MODIFIER	Mettl3	ENSMUSG00000022160	Transcript	ENSMUST00000173546	retained_intron	rs48774859
14:52297251-52297251	upstream_gene_variant	MODIFIER	Mettl3	ENSMUSG00000022160	Transcript	ENSMUST00000173656	protein_coding	rs48774859
14:52297251-52297251	downstream_gene_variant	MODIFIER	Mettl3	ENSMUSG00000022160	Transcript	ENSMUST00000173896	nonsense_mediated_decay	rs48774859
14:52297251-52297251	intron_variant,NMD_transcript_v	MODIFIER	Mettl3	ENSMUSG00000022160	Transcript	ENSMUST00000174351	nonsense_mediated_decay	rs48774859
14:52297251-52297251	upstream_gene_variant	MODIFIER	Mettl3	ENSMUSG00000022160	Transcript	ENSMUST00000174360	protein_coding	rs48774859
14:52297251-52297251	downstream_gene_variant	MODIFIER	Mettl3	ENSMUSG00000022160	Transcript	ENSMUST00000174853	protein_coding	rs48774859
14:52311921-52311921	3_prime_UTR_variant	MODIFIER	Sall2	ENSMUSG00000049532	Transcript	ENSMUST00000058326	protein_coding	rs30318417
14:52311921-52311921	3_prime_UTR_variant	MODIFIER	Sall2	ENSMUSG00000049532	Transcript	ENSMUST00000135523	protein_coding	rs30318417
14:52311921-52311921	regulatory_region_variant	MODIFIER	-	-	RegulatoryFeature	ENSMUSR00000560522	CTCF_binding_site	rs30318417
14:53797738-53797738	downstream_gene_variant	MODIFIER	B230359F08Rik	ENSMUSG00000087666	Transcript	ENSMUST00000103671	TR_V_gene	rs3140307
14:54139709-54139709	downstream_gene_variant	MODIFIER	Trdj2	ENSMUSG00000076871	Transcript	ENSMUST00000103683	TR_J_gene	rs3021908
14:54139709-54139709	upstream_gene_variant	MODIFIER	Trdc	ENSMUSG00000104876	Transcript	ENSMUST00000196323	TR_C_gene	rs3021908
14:54139709-54139709	intron_variant,non_coding_trans	MODIFIER	Gm43434	ENSMUSG00000106112	Transcript	ENSMUST00000196615	antisense	rs3021908
14:54236847-54236847	intron_variant	MODIFIER	Dad1	ENSMUSG00000022174	Transcript	ENSMUST00000022781	protein_coding	rs48352027
14:54236847-54236847	downstream_gene_variant	MODIFIER	Dad1	ENSMUSG00000022174	Transcript	ENSMUST00000128231	protein_coding	rs48352027
14:54507977-54507977	intron_variant	MODIFIER	Prmt5	ENSMUSG00000023110	Transcript	ENSMUST00000023873	protein_coding	rs30639676
14:54507977-54507977	downstream_gene_variant	MODIFIER	Prmt5	ENSMUSG00000023110	Transcript	ENSMUST00000132227	nonsense_mediated_decay	rs30639676
14:54507977-54507977	downstream_gene_variant	MODIFIER	Prmt5	ENSMUSG00000023110	Transcript	ENSMUST00000139964	protein_coding	rs30639676
14:54546803-54546803	intron_variant	MODIFIER	Haus4	ENSMUSG00000022177	Transcript	ENSMUST00000022784	protein_coding	rs30965041
14:54546803-54546803	upstream_gene_variant	MODIFIER	Haus4	ENSMUSG00000022177	Transcript	ENSMUST00000226250	retained_intron	rs30965041
14:54546803-54546803	intron_variant	MODIFIER	Haus4	ENSMUSG00000022177	Transcript	ENSMUST00000226358	protein_coding	rs30965041
14:54546803-54546803	upstream_gene_variant	MODIFIER	Haus4	ENSMUSG00000022177	Transcript	ENSMUST00000226832	protein_coding	rs30965041
14:54546803-54546803	intron_variant,non_coding_trans	MODIFIER	Haus4	ENSMUSG00000022177	Transcript	ENSMUST00000227954	retained_intron	rs30965041
14:54546803-54546803	downstream_gene_variant	MODIFIER	Haus4	ENSMUSG00000022177	Transcript	ENSMUST00000228883	processed_transcript	rs30965041
14:54546817-54546818	intron_variant	MODIFIER	Haus4	ENSMUSG00000022177	Transcript	ENSMUST00000022784	protein_coding	rs216861770
14:54546817-54546818	upstream_gene_variant	MODIFIER	Haus4	ENSMUSG00000022177	Transcript	ENSMUST00000226250	retained_intron	rs216861770
14:54546817-54546818	intron_variant	MODIFIER	Haus4	ENSMUSG00000022177	Transcript	ENSMUST00000226358	protein_coding	rs216861770
14:54546817-54546818	upstream_gene_variant	MODIFIER	Haus4	ENSMUSG00000022177	Transcript	ENSMUST00000226832	protein_coding	rs216861770
14:54546817-54546818	intron_variant,non_coding_trans	MODIFIER	Haus4	ENSMUSG00000022177	Transcript	ENSMUST00000227954	retained_intron	rs216861770
14:54546817-54546818	downstream_gene_variant	MODIFIER	Haus4	ENSMUSG00000022177	Transcript	ENSMUST00000228883	processed_transcript	rs216861770
14:54874388-54874388	upstream_gene_variant	MODIFIER	Homez	ENSMUSG00000057156	Transcript	ENSMUST00000146642	protein_coding	rs31224553
14:54874388-54874388	downstream_gene_variant	MODIFIER	Ppp1r3e	ENSMUSG00000072494	Transcript	ENSMUST00000168622	protein_coding	rs31224553
14:54874388-54874388	intron_variant	MODIFIER	Ppp1r3e	ENSMUSG00000072494	Transcript	ENSMUST00000177403	protein_coding	rs31224553
14:54874388-54874388	upstream_gene_variant	MODIFIER	Ppp1r3e	ENSMUSG00000072494	Transcript	ENSMUST00000182700	processed_transcript	rs31224553
14:54874388-54874388	downstream_gene_variant	MODIFIER	Ppp1r3e	ENSMUSG00000072494	Transcript	ENSMUST00000194094	retained_intron	rs31224553
14:54888907-54888907	downstream_gene_variant	MODIFIER	Bcl2l2	ENSMUSG00000089682	Transcript	ENSMUST00000022806	protein_coding	rs46731880
14:54888907-54888907	downstream_gene_variant	MODIFIER	Bcl2l2	ENSMUSG00000089682	Transcript	ENSMUST00000131243	retained_intron	rs46731880
14:54888907-54888907	downstream_gene_variant	MODIFIER	Bcl2l2	ENSMUSG00000089682	Transcript	ENSMUST00000133397	protein_coding	rs46731880
14:54888907-54888907	intron_variant	MODIFIER	Gm20521	ENSMUSG00000092232	Transcript	ENSMUST00000134077	protein_coding	rs46731880
14:54888907-54888907	upstream_gene_variant	MODIFIER	Pabpn1	ENSMUSG00000022194	Transcript	ENSMUST00000140691	protein_coding	rs46731880
14:54888907-54888907	downstream_gene_variant	MODIFIER	Bcl2l2	ENSMUSG00000089682	Transcript	ENSMUST00000172844	protein_coding	rs46731880
14:54888907-54888907	downstream_gene_variant	MODIFIER	Bcl2l2	ENSMUSG00000089682	Transcript	ENSMUST00000227108	protein_coding	rs46731880
14:54890393-54890393	downstream_gene_variant	MODIFIER	Bcl2l2	ENSMUSG00000089682	Transcript	ENSMUST00000022806	protein_coding	rs31039289
14:54890393-54890393	upstream_gene_variant	MODIFIER	Pabpn1	ENSMUSG00000022194	Transcript	ENSMUST00000022808	protein_coding	rs31039289
14:54890393-54890393	upstream_gene_variant	MODIFIER	Pabpn1	ENSMUSG00000022194	Transcript	ENSMUST00000116476	protein_coding	rs31039289
14:54890393-54890393	downstream_gene_variant	MODIFIER	Bcl2l2	ENSMUSG00000089682	Transcript	ENSMUST00000133397	protein_coding	rs31039289
14:54890393-54890393	intron_variant	MODIFIER	Gm20521	ENSMUSG00000092232	Transcript	ENSMUST00000134077	protein_coding	rs31039289
14:54890393-54890393	upstream_gene_variant	MODIFIER	Pabpn1	ENSMUSG00000022194	Transcript	ENSMUST00000139985	protein_coding	rs31039289
14:54890393-54890393	upstream_gene_variant	MODIFIER	Pabpn1	ENSMUSG00000022194	Transcript	ENSMUST00000140691	protein_coding	rs31039289
14:54890393-54890393	upstream_gene_variant	MODIFIER	Pabpn1	ENSMUSG00000022194	Transcript	ENSMUST00000141446	protein_coding	rs31039289
14:54890393-54890393	upstream_gene_variant	MODIFIER	Pabpn1	ENSMUSG00000022194	Transcript	ENSMUST00000146271	retained_intron	rs31039289
14:54890393-54890393	upstream_gene_variant	MODIFIER	Pabpn1	ENSMUSG00000022194	Transcript	ENSMUST00000150975	protein_coding	rs31039289
14:54890393-54890393	upstream_gene_variant	MODIFIER	Pabpn1	ENSMUSG00000022194	Transcript	ENSMUST00000172557	protein_coding	rs31039289
14:54890393-54890393	upstream_gene_variant	MODIFIER	Pabpn1	ENSMUSG00000022194	Transcript	ENSMUST00000172695	nonsense_mediated_decay	rs31039289
14:54890393-54890393	downstream_gene_variant	MODIFIER	Bcl2l2	ENSMUSG00000089682	Transcript	ENSMUST00000172844	protein_coding	rs31039289
14:54890870-54890870	downstream_gene_variant	MODIFIER	Bcl2l2	ENSMUSG00000089682	Transcript	ENSMUST00000022806	protein_coding	rs31540841
14:54890870-54890870	upstream_gene_variant	MODIFIER	Pabpn1	ENSMUSG00000022194	Transcript	ENSMUST00000022808	protein_coding	rs31540841
14:54890870-54890870	upstream_gene_variant	MODIFIER	Pabpn1	ENSMUSG00000022194	Transcript	ENSMUST00000116476	protein_coding	rs31540841
14:54890870-54890870	downstream_gene_variant	MODIFIER	Bcl2l2	ENSMUSG00000089682	Transcript	ENSMUST00000133397	protein_coding	rs31540841
14:54890870-54890870	intron_variant	MODIFIER	Gm20521	ENSMUSG00000092232	Transcript	ENSMUST00000134077	protein_coding	rs31540841

14:54890870-54890870	upstream_gene_variant	MODIFIER	Pabpn1	ENSMUSG00000022194	Transcript	ENSMUST00000139985	protein_coding	rs31540841
14:54890870-54890870	upstream_gene_variant	MODIFIER	Pabpn1	ENSMUSG00000022194	Transcript	ENSMUST00000140691	protein_coding	rs31540841
14:54890870-54890870	upstream_gene_variant	MODIFIER	Pabpn1	ENSMUSG00000022194	Transcript	ENSMUST00000141446	protein_coding	rs31540841
14:54890870-54890870	upstream_gene_variant	MODIFIER	Pabpn1	ENSMUSG00000022194	Transcript	ENSMUST00000146271	retained_intron	rs31540841
14:54890870-54890870	upstream_gene_variant	MODIFIER	Pabpn1	ENSMUSG00000022194	Transcript	ENSMUST00000150975	protein_coding	rs31540841
14:54890870-54890870	upstream_gene_variant	MODIFIER	Pabpn1	ENSMUSG00000022194	Transcript	ENSMUST00000172557	protein_coding	rs31540841
14:54890870-54890870	upstream_gene_variant	MODIFIER	Pabpn1	ENSMUSG00000022194	Transcript	ENSMUST00000172695	nonsense_mediated_decay	rs31540841
14:54891731-54891731	downstream_gene_variant	MODIFIER	Bcl2l2	ENSMUSG00000089682	Transcript	ENSMUST00000022806	protein_coding	rs211969829
14:54891731-54891731	upstream_gene_variant	MODIFIER	Pabpn1	ENSMUSG00000022194	Transcript	ENSMUST00000022808	protein_coding	rs211969829
14:54891731-54891731	upstream_gene_variant	MODIFIER	Pabpn1	ENSMUSG00000022194	Transcript	ENSMUST00000116476	protein_coding	rs211969829
14:54891731-54891731	downstream_gene_variant	MODIFIER	Bcl2l2	ENSMUSG00000089682	Transcript	ENSMUST00000133397	protein_coding	rs211969829
14:54891731-54891731	intron_variant	MODIFIER	Gm20521	ENSMUSG00000092232	Transcript	ENSMUST00000134077	protein_coding	rs211969829
14:54891731-54891731	upstream_gene_variant	MODIFIER	Pabpn1	ENSMUSG00000022194	Transcript	ENSMUST00000139985	protein_coding	rs211969829
14:54891731-54891731	upstream_gene_variant	MODIFIER	Pabpn1	ENSMUSG00000022194	Transcript	ENSMUST00000140691	protein_coding	rs211969829
14:54891731-54891731	upstream_gene_variant	MODIFIER	Pabpn1	ENSMUSG00000022194	Transcript	ENSMUST00000141446	protein_coding	rs211969829
14:54891731-54891731	upstream_gene_variant	MODIFIER	Pabpn1	ENSMUSG00000022194	Transcript	ENSMUST00000146271	retained_intron	rs211969829
14:54891731-54891731	upstream_gene_variant	MODIFIER	Pabpn1	ENSMUSG00000022194	Transcript	ENSMUST00000150975	protein_coding	rs211969829
14:54891731-54891731	upstream_gene_variant	MODIFIER	Pabpn1	ENSMUSG00000022194	Transcript	ENSMUST00000172557	protein_coding	rs211969829
14:54891731-54891731	upstream_gene_variant	MODIFIER	Pabpn1	ENSMUSG00000022194	Transcript	ENSMUST00000172695	nonsense_mediated_decay	rs211969829
14:54891843-54891843	downstream_gene_variant	MODIFIER	Bcl2l2	ENSMUSG00000089682	Transcript	ENSMUST00000022806	protein_coding	rs236779742
14:54891843-54891843	upstream_gene_variant	MODIFIER	Pabpn1	ENSMUSG00000022194	Transcript	ENSMUST00000022808	protein_coding	rs236779742
14:54891843-54891843	upstream_gene_variant	MODIFIER	Pabpn1	ENSMUSG00000022194	Transcript	ENSMUST00000116476	protein_coding	rs236779742
14:54891843-54891843	downstream_gene_variant	MODIFIER	Bcl2l2	ENSMUSG00000089682	Transcript	ENSMUST00000133397	protein_coding	rs236779742
14:54891843-54891843	intron_variant	MODIFIER	Gm20521	ENSMUSG00000092232	Transcript	ENSMUST00000134077	protein_coding	rs236779742
14:54891843-54891843	upstream_gene_variant	MODIFIER	Pabpn1	ENSMUSG00000022194	Transcript	ENSMUST00000139985	protein_coding	rs236779742
14:54891843-54891843	upstream_gene_variant	MODIFIER	Pabpn1	ENSMUSG00000022194	Transcript	ENSMUST00000140691	protein_coding	rs236779742
14:54891843-54891843	upstream_gene_variant	MODIFIER	Pabpn1	ENSMUSG00000022194	Transcript	ENSMUST00000141446	protein_coding	rs236779742
14:54891843-54891843	upstream_gene_variant	MODIFIER	Pabpn1	ENSMUSG00000022194	Transcript	ENSMUST00000146271	retained_intron	rs236779742
14:54891843-54891843	upstream_gene_variant	MODIFIER	Pabpn1	ENSMUSG00000022194	Transcript	ENSMUST00000150975	protein_coding	rs236779742
14:54891843-54891843	upstream_gene_variant	MODIFIER	Pabpn1	ENSMUSG00000022194	Transcript	ENSMUST00000172557	protein_coding	rs236779742
14:54891843-54891843	upstream_gene_variant	MODIFIER	Pabpn1	ENSMUSG00000022194	Transcript	ENSMUST00000172695	nonsense_mediated_decay	rs236779742
14:55017172-55017172	intron_variant	MODIFIER	Ngdn	ENSMUSG00000022204	Transcript	ENSMUST00000022815	protein_coding	rs49311004
14:55017172-55017172	upstream_gene_variant	MODIFIER	Ngdn	ENSMUSG00000022204	Transcript	ENSMUST00000226258	retained_intron	rs49311004
14:55017172-55017172	upstream_gene_variant	MODIFIER	Ngdn	ENSMUSG00000022204	Transcript	ENSMUST00000226658	retained_intron	rs49311004
14:55017172-55017172	upstream_gene_variant	MODIFIER	Ngdn	ENSMUSG00000022204	Transcript	ENSMUST00000227833	retained_intron	rs49311004
14:55018244-55018244	intron_variant	MODIFIER	Ngdn	ENSMUSG00000022204	Transcript	ENSMUST00000022815	protein_coding	rs260858043
14:55018244-55018244	upstream_gene_variant	MODIFIER	Ngdn	ENSMUSG00000022204	Transcript	ENSMUST00000226258	retained_intron	rs260858043
14:55018244-55018244	upstream_gene_variant	MODIFIER	Ngdn	ENSMUSG00000022204	Transcript	ENSMUST00000226658	retained_intron	rs260858043
14:55018244-55018244	upstream_gene_variant	MODIFIER	Ngdn	ENSMUSG00000022204	Transcript	ENSMUST00000227833	retained_intron	rs260858043
14:55022532-55022532	intron_variant	MODIFIER	Ngdn	ENSMUSG00000022204	Transcript	ENSMUST00000022815	protein_coding	rs47102783
14:55022532-55022532	downstream_gene_variant	MODIFIER	Ngdn	ENSMUSG00000022204	Transcript	ENSMUST00000226258	retained_intron	rs47102783
14:55022532-55022532	downstream_gene_variant	MODIFIER	Ngdn	ENSMUSG00000022204	Transcript	ENSMUST00000226658	retained_intron	rs47102783
14:55022532-55022532	downstream_gene_variant	MODIFIER	Ngdn	ENSMUSG00000022204	Transcript	ENSMUST00000227833	retained_intron	rs47102783
14:55067573-55067573	synonymous_variant	LOW	Zfhx2	ENSMUSG00000040721	Transcript	ENSMUST00000036328	protein_coding	rs31339308
14:55067573-55067573	non_coding_transcript_exon_var	MODIFIER	Zfhx2	ENSMUSG00000040721	Transcript	ENSMUST00000176665	retained_intron	rs31339308
14:55067573-55067573	intron_variant,non_coding_trans	MODIFIER	Zfhx2os	ENSMUSG00000093452	Transcript	ENSMUST00000183750	antisense	rs31339308
14:55067573-55067573	intron_variant,non_coding_trans	MODIFIER	Gm20687	ENSMUSG00000100162	Transcript	ENSMUST00000183822	antisense	rs31339308
14:55067573-55067573	intron_variant,non_coding_trans	MODIFIER	Gm20687	ENSMUSG00000100162	Transcript	ENSMUST00000183993	antisense	rs31339308
14:55067573-55067573	intron_variant,non_coding_trans	MODIFIER	Gm20687	ENSMUSG00000100162	Transcript	ENSMUST00000185121	antisense	rs31339308
14:55068120-55068120	synonymous_variant	LOW	Zfhx2	ENSMUSG00000040721	Transcript	ENSMUST00000036328	protein_coding	rs47182693
14:55068120-55068120	non_coding_transcript_exon_var	MODIFIER	Zfhx2	ENSMUSG00000040721	Transcript	ENSMUST00000176665	retained_intron	rs47182693
14:55068120-55068120	intron_variant,non_coding_trans	MODIFIER	Zfhx2os	ENSMUSG00000093452	Transcript	ENSMUST00000183750	antisense	rs47182693
14:55068120-55068120	intron_variant,non_coding_trans	MODIFIER	Gm20687	ENSMUSG00000100162	Transcript	ENSMUST00000183822	antisense	rs47182693
14:55068120-55068120	intron_variant,non_coding_trans	MODIFIER	Gm20687	ENSMUSG00000100162	Transcript	ENSMUST00000183993	antisense	rs47182693
14:55068120-55068120	intron_variant,non_coding_trans	MODIFIER	Gm20687	ENSMUSG00000100162	Transcript	ENSMUST00000185121	antisense	rs47182693
14:61612683-61612683	3_prime_UTR_variant	MODIFIER	Kcnrg	ENSMUSG00000046168	Transcript	ENSMUST00000051184	protein_coding	rs30462687
14:61612683-61612683	intron_variant,non_coding_trans	MODIFIER	Dleu2	ENSMUSG00000097589	Transcript	ENSMUST00000182259	lincRNA	rs30462687
14:61612683-61612683	intron_variant,non_coding_trans	MODIFIER	Dleu2	ENSMUSG00000097589	Transcript	ENSMUST00000182325	lincRNA	rs30462687
14:61612683-61612683	intron_variant,non_coding_trans	MODIFIER	Dleu2	ENSMUSG00000097589	Transcript	ENSMUST00000182768	lincRNA	rs30462687
14:61612683-61612683	intron_variant,non_coding_trans	MODIFIER	Dleu2	ENSMUSG00000097589	Transcript	ENSMUST00000183054	lincRNA	rs30462687
14:61612683-61612683	intron_variant,non_coding_trans	MODIFIER	Dleu2	ENSMUSG00000097589	Transcript	ENSMUST00000183066	lincRNA	rs30462687
14:61612683-61612683	3_prime_UTR_variant	MODIFIER	Kcnrg	ENSMUSG00000046168	Transcript	ENSMUST00000225582	protein_coding	rs30462687
14:62693850-62693850	intron_variant	MODIFIER	Ints6	ENSMUSG00000035161	Transcript	ENSMUST00000053959	protein_coding	rs45951550
14:62693850-62693850	downstream_gene_variant	MODIFIER	Serpine3	ENSMUSG00000091155	Transcript	ENSMUST00000171692	protein_coding	rs45951550
14:62693850-62693850	intron_variant	MODIFIER	Ints6	ENSMUSG00000035161	Transcript	ENSMUST00000223585	protein_coding	rs45951550
14:63144541-63144541	3_prime_UTR_variant	MODIFIER	Ctsb	ENSMUSG00000021939	Transcript	ENSMUST00000006235	protein_coding	-
14:63144541-63144541	downstream_gene_variant	MODIFIER	Fdft1	ENSMUSG00000021273	Transcript	ENSMUST00000054963	protein_coding	-
14:63144541-63144541	downstream_gene_variant	MODIFIER	Fdft1	ENSMUSG00000021273	Transcript	ENSMUST00000224625	protein_coding	-
14:63144541-63144541	downstream_gene_variant	MODIFIER	Ctsb	ENSMUSG00000021939	Transcript	ENSMUST00000225540	retained_intron	-

14:63144541-63144541	regulatory_region_variant	MODIFIER	-	-	RegulatoryFeature	ENSMUSR00000562215	open_chromatin_region	-
14:63524649-63524649	intron_variant	MODIFIER	Mtmr9	ENSMUSG00000035078	Transcript	ENSMUST00000058679	protein_coding	rs31013204
14:63524649-63524649	intron_variant	MODIFIER	Mtmr9	ENSMUSG00000035078	Transcript	ENSMUST00000225449	protein_coding	rs31013204
14:65394668-65394668	intron_variant	MODIFIER	Zfp395	ENSMUSG00000034522	Transcript	ENSMUST00000066994	protein_coding	rs31105555
14:65394668-65394668	upstream_gene_variant	MODIFIER	Zfp395	ENSMUSG00000034522	Transcript	ENSMUST00000225512	retained_intron	rs31105555
14:69243134-69243137	3_prime_UTR_variant	MODIFIER	Slc25a37	ENSMUSG00000034248	Transcript	ENSMUST00000037064	protein_coding	rs217663790
14:69243134-69243137	non_coding_transcript_exon_va	MODIFIER	Slc25a37	ENSMUSG00000034248	Transcript	ENSMUST00000184497	retained_intron	rs217663790
14:69243134-69243137	downstream_gene_variant	MODIFIER	Slc25a37	ENSMUSG00000034248	Transcript	ENSMUST00000184914	nonsense_mediated_decay	rs217663790
14:69697818-69697818	downstream_gene_variant	MODIFIER	Loxl2	ENSMUSG00000034205	Transcript	ENSMUST00000022660	protein_coding	rs30353057
14:69697818-69697818	downstream_gene_variant	MODIFIER	Loxl2	ENSMUSG00000034205	Transcript	ENSMUST00000100420	protein_coding	rs30353057
14:69697818-69697818	intron_variant	MODIFIER	R3hcc1	ENSMUSG00000034194	Transcript	ENSMUST00000118374	protein_coding	rs30353057
14:69697818-69697818	intron_variant	MODIFIER	R3hcc1	ENSMUSG00000034194	Transcript	ENSMUST00000121142	protein_coding	rs30353057
14:69697818-69697818	downstream_gene_variant	MODIFIER	R3hcc1	ENSMUSG00000034194	Transcript	ENSMUST00000138326	retained_intron	rs30353057
14:69697818-69697818	intron_variant	MODIFIER	R3hcc1	ENSMUSG00000034194	Transcript	ENSMUST00000216152	protein_coding	rs30353057
14:7943204-7943204	intron_variant	MODIFIER	Flnb	ENSMUSG00000025278	Transcript	ENSMUST00000052678	protein_coding	rs50779522
14:7943204-7943204	regulatory_region_variant	MODIFIER	-	-	RegulatoryFeature	ENSMUSR00000344611	enhancer	rs50779522
14:7944021-7944021	intron_variant	MODIFIER	Flnb	ENSMUSG00000025278	Transcript	ENSMUST00000052678	protein_coding	rs48896371
14:7944021-7944021	regulatory_region_variant	MODIFIER	-	-	RegulatoryFeature	ENSMUSR00000344611	enhancer	rs48896371
14:7944065-7944065	intron_variant	MODIFIER	Flnb	ENSMUSG00000025278	Transcript	ENSMUST00000052678	protein_coding	rs51343795
14:7944065-7944065	regulatory_region_variant	MODIFIER	-	-	RegulatoryFeature	ENSMUSR00000344611	enhancer	rs51343795
14:7945611-7945611	intron_variant	MODIFIER	Flnb	ENSMUSG00000025278	Transcript	ENSMUST00000052678	protein_coding	rs107933386
14:7945624-7945624	intron_variant	MODIFIER	Flnb	ENSMUSG00000025278	Transcript	ENSMUST00000052678	protein_coding	rs108416006
14:8056653-8056653	downstream_gene_variant	MODIFIER	Abhd6	ENSMUSG00000025277	Transcript	ENSMUST00000026313	protein_coding	rs253972729
14:8056653-8056653	3_prime_UTR_variant	MODIFIER	Abhd6	ENSMUSG00000025277	Transcript	ENSMUST00000166497	protein_coding	rs253972729
14:8056653-8056653	3_prime_UTR_variant	MODIFIER	Abhd6	ENSMUSG00000025277	Transcript	ENSMUST00000225234	protein_coding	rs253972729
14:8162296-8162296	downstream_gene_variant	MODIFIER	Pdhb	ENSMUSG00000021748	Transcript	ENSMUST00000022268	protein_coding	rs50907424
14:8162296-8162296	intron_variant	MODIFIER	Pxk	ENSMUSG00000033885	Transcript	ENSMUST00000036682	protein_coding	rs50907424
14:8162296-8162296	intron_variant	MODIFIER	Pxk	ENSMUSG00000033885	Transcript	ENSMUST00000112689	protein_coding	rs50907424
14:8162296-8162296	intron_variant	MODIFIER	Pxk	ENSMUSG00000033885	Transcript	ENSMUST00000225653	protein_coding	rs50907424
14:8162296-8162296	downstream_gene_variant	MODIFIER	Pdhb	ENSMUSG00000021748	Transcript	ENSMUST00000225747	retained_intron	rs50907424
14:8165329-8165329	downstream_gene_variant	MODIFIER	Pdhb	ENSMUSG00000021748	Transcript	ENSMUST00000022268	protein_coding	rs31461919
14:8165329-8165329	downstream_gene_variant	MODIFIER	Pxk	ENSMUSG00000033885	Transcript	ENSMUST00000036682	protein_coding	rs31461919
14:8165329-8165329	downstream_gene_variant	MODIFIER	Pxk	ENSMUSG00000033885	Transcript	ENSMUST00000112689	protein_coding	rs31461919
14:8165329-8165329	downstream_gene_variant	MODIFIER	Pdhb	ENSMUSG00000021748	Transcript	ENSMUST00000224466	processed_transcript	rs31461919
14:8165329-8165329	downstream_gene_variant	MODIFIER	Pdhb	ENSMUSG00000021748	Transcript	ENSMUST00000225274	processed_transcript	rs31461919
14:8165329-8165329	downstream_gene_variant	MODIFIER	Pxk	ENSMUSG00000033885	Transcript	ENSMUST00000225653	protein_coding	rs31461919
14:8165329-8165329	downstream_gene_variant	MODIFIER	Pdhb	ENSMUSG00000021748	Transcript	ENSMUST00000225747	retained_intron	rs31461919
14:8214985-8214985	intron_variant	MODIFIER	Kctd6	ENSMUSG00000021752	Transcript	ENSMUST00000022272	protein_coding	rs258689661
14:8214985-8214985	5_prime_UTR_variant	MODIFIER	Kctd6	ENSMUSG00000021752	Transcript	ENSMUST00000170111	protein_coding	rs258689661
14:8214985-8214985	regulatory_region_variant	MODIFIER	-	-	RegulatoryFeature	ENSMUSR00000082367	promoter	rs258689661
14:8217551-8217551	intron_variant	MODIFIER	Kctd6	ENSMUSG00000021752	Transcript	ENSMUST00000022272	protein_coding	rs236042515
14:8217551-8217551	intron_variant	MODIFIER	Kctd6	ENSMUSG00000021752	Transcript	ENSMUST00000170111	protein_coding	rs236042515
14:13957841-13957841	intron_variant	MODIFIER	Thoc7	ENSMUSG00000053453	Transcript	ENSMUST00000065865	protein_coding	rs45877323
14:13957841-13957841	intron_variant,NMD_transcript_v	MODIFIER	Thoc7	ENSMUSG00000053453	Transcript	ENSMUST00000223832	nonsense_mediated_decay	rs45877323
14:13957841-13957841	upstream_gene_variant	MODIFIER	Atxn7	ENSMUSG00000021738	Transcript	ENSMUST00000223880	protein_coding	rs45877323
14:13957841-13957841	intron_variant,non_coding_trans	MODIFIER	Thoc7	ENSMUSG00000053453	Transcript	ENSMUST00000224015	processed_transcript	rs45877323
14:13957841-13957841	upstream_gene_variant	MODIFIER	Thoc7	ENSMUSG00000053453	Transcript	ENSMUST00000224652	processed_transcript	rs45877323
14:13957841-13957841	upstream_gene_variant	MODIFIER	Thoc7	ENSMUSG00000053453	Transcript	ENSMUST00000224764	retained_intron	rs45877323
14:13957841-13957841	intron_variant,non_coding_trans	MODIFIER	Thoc7	ENSMUSG00000053453	Transcript	ENSMUST00000225202	processed_transcript	rs45877323
14:13957841-13957841	intron_variant,non_coding_trans	MODIFIER	Thoc7	ENSMUSG00000053453	Transcript	ENSMUST00000225317	retained_intron	rs45877323
14:13957841-13957841	intron_variant	MODIFIER	Thoc7	ENSMUSG00000053453	Transcript	ENSMUST00000225325	protein_coding	rs45877323
14:13957841-13957841	intron_variant,non_coding_trans	MODIFIER	Thoc7	ENSMUSG00000053453	Transcript	ENSMUST00000225401	processed_transcript	rs45877323
14:13957841-13957841	upstream_gene_variant	MODIFIER	Thoc7	ENSMUSG00000053453	Transcript	ENSMUST00000225559	nonsense_mediated_decay	rs45877323
14:13957841-13957841	intron_variant,NMD_transcript_v	MODIFIER	Thoc7	ENSMUSG00000053453	Transcript	ENSMUST00000225590	nonsense_mediated_decay	rs45877323
14:13957841-13957841	intron_variant	MODIFIER	Thoc7	ENSMUSG00000053453	Transcript	ENSMUST00000225891	protein_coding	rs45877323
14:13957841-13957841	upstream_gene_variant	MODIFIER	Thoc7	ENSMUSG00000053453	Transcript	ENSMUST00000226008	processed_transcript	rs45877323
14:13960372-13960372	intron_variant	MODIFIER	Thoc7	ENSMUSG00000053453	Transcript	ENSMUST00000065865	protein_coding	rs49865615
14:13960372-13960372	intron_variant,NMD_transcript_v	MODIFIER	Thoc7	ENSMUSG00000053453	Transcript	ENSMUST00000223832	nonsense_mediated_decay	rs49865615
14:13960372-13960372	upstream_gene_variant	MODIFIER	Atxn7	ENSMUSG00000021738	Transcript	ENSMUST00000223880	protein_coding	rs49865615
14:13960372-13960372	intron_variant,non_coding_trans	MODIFIER	Thoc7	ENSMUSG00000053453	Transcript	ENSMUST00000224015	processed_transcript	rs49865615
14:13960372-13960372	upstream_gene_variant	MODIFIER	Thoc7	ENSMUSG00000053453	Transcript	ENSMUST00000224652	processed_transcript	rs49865615
14:13960372-13960372	upstream_gene_variant	MODIFIER	Thoc7	ENSMUSG00000053453	Transcript	ENSMUST00000224764	retained_intron	rs49865615
14:13960372-13960372	intron_variant,non_coding_trans	MODIFIER	Thoc7	ENSMUSG00000053453	Transcript	ENSMUST00000225202	processed_transcript	rs49865615
14:13960372-13960372	intron_variant,non_coding_trans	MODIFIER	Thoc7	ENSMUSG00000053453	Transcript	ENSMUST00000225317	retained_intron	rs49865615
14:13960372-13960372	intron_variant	MODIFIER	Thoc7	ENSMUSG00000053453	Transcript	ENSMUST00000225325	protein_coding	rs49865615
14:13960372-13960372	intron_variant,non_coding_trans	MODIFIER	Thoc7	ENSMUSG00000053453	Transcript	ENSMUST00000225401	processed_transcript	rs49865615
14:13960372-13960372	intron_variant,NMD_transcript_v	MODIFIER	Thoc7	ENSMUSG00000053453	Transcript	ENSMUST00000225590	nonsense_mediated_decay	rs49865615
14:13960372-13960372	intron_variant	MODIFIER	Thoc7	ENSMUSG00000053453	Transcript	ENSMUST00000225891	protein_coding	rs49865615
14:13960372-13960372	upstream_gene_variant	MODIFIER	Thoc7	ENSMUSG00000053453	Transcript	ENSMUST00000226008	processed_transcript	rs49865615

14:13960372-13960372	regulatory_region_variant	MODIFIER	-	-	RegulatoryFeature	ENSMUSR00000082855	promoter	rs49865615
14:14098198-14098198	intron_variant	MODIFIER	Atxn7	ENSMUSG00000021738	Transcript	ENSMUST00000022257	protein_coding	rs49473443
14:14098198-14098198	intron_variant	MODIFIER	Atxn7	ENSMUSG00000021738	Transcript	ENSMUST00000223714	protein_coding	rs49473443
14:14098198-14098198	intron_variant	MODIFIER	Atxn7	ENSMUSG00000021738	Transcript	ENSMUST00000223880	protein_coding	rs49473443
14:14098198-14098198	intron_variant,non_coding_trans	MODIFIER	Atxn7	ENSMUSG00000021738	Transcript	ENSMUST00000223932	retained_intron	rs49473443
14:14098198-14098198	intron_variant	MODIFIER	Atxn7	ENSMUSG00000021738	Transcript	ENSMUST00000224315	protein_coding	rs49473443
14:14098198-14098198	regulatory_region_variant	MODIFIER	-	-	RegulatoryFeature	ENSMUSR00000344937	CTCF_binding_site	rs49473443
14:16295267-16295267	intron_variant	MODIFIER	Ngly1	ENSMUSG00000021785	Transcript	ENSMUST00000022310	protein_coding	rs30833843
14:16295267-16295267	intron_variant	MODIFIER	Ngly1	ENSMUSG00000021785	Transcript	ENSMUST00000224656	protein_coding	rs30833843
14:16300908-16300908	intron_variant	MODIFIER	Ngly1	ENSMUSG00000021785	Transcript	ENSMUST00000022310	protein_coding	rs252456526
14:16300908-16300908	upstream_gene_variant	MODIFIER	Gm47798	ENSMUSG00000114253	Transcript	ENSMUST00000224494	sense_intronic	rs252456526
14:16300908-16300908	intron_variant	MODIFIER	Ngly1	ENSMUSG00000021785	Transcript	ENSMUST00000224656	protein_coding	rs252456526
14:18214035-18214035	intron_variant	MODIFIER	Nr1d2	ENSMUSG00000021775	Transcript	ENSMUST00000090543	protein_coding	rs30194888
14:18214035-18214035	downstream_gene_variant	MODIFIER	Nr1d2	ENSMUSG00000021775	Transcript	ENSMUST00000143308	retained_intron	rs30194888
14:18214035-18214035	upstream_gene_variant	MODIFIER	Nr1d2	ENSMUSG00000021775	Transcript	ENSMUST00000225308	retained_intron	rs30194888
14:18214035-18214035	intron_variant	MODIFIER	Nr1d2	ENSMUSG00000021775	Transcript	ENSMUST00000225491	protein_coding	rs30194888
14:18283470-18283470	downstream_gene_variant	MODIFIER	Nkiras1	ENSMUSG00000021772	Transcript	ENSMUST00000022294	nonsense_mediated_decay	rs30927562
14:18283470-18283470	intron_variant	MODIFIER	Ube2e1	ENSMUSG00000021774	Transcript	ENSMUST00000022296	protein_coding	rs30927562
14:18283470-18283470	3_prime_UTR_variant	MODIFIER	Nkiras1	ENSMUSG00000021772	Transcript	ENSMUST00000132374	protein_coding	rs30927562
14:18283470-18283470	downstream_gene_variant	MODIFIER	Nkiras1	ENSMUSG00000021772	Transcript	ENSMUST00000133460	protein_coding	rs30927562
14:18283470-18283470	downstream_gene_variant	MODIFIER	Nkiras1	ENSMUSG00000021772	Transcript	ENSMUST00000156186	processed_transcript	rs30927562
14:18283470-18283470	downstream_gene_variant	MODIFIER	Ube2e1	ENSMUSG00000021774	Transcript	ENSMUST00000160266	retained_intron	rs30927562
14:18283470-18283470	downstream_gene_variant	MODIFIER	Ube2e1	ENSMUSG00000021774	Transcript	ENSMUST00000162831	processed_transcript	rs30927562
14:18283470-18283470	intron_variant	MODIFIER	Ube2e1	ENSMUSG00000021774	Transcript	ENSMUST00000225612	protein_coding	rs30927562
14:19879762-19879762	intron_variant	MODIFIER	Gng2	ENSMUSG00000043004	Transcript	ENSMUST00000055100	protein_coding	rs30673412
14:19879762-19879762	intron_variant	MODIFIER	Gng2	ENSMUSG00000043004	Transcript	ENSMUST00000159028	protein_coding	rs30673412
14:19879762-19879762	intron_variant	MODIFIER	Gng2	ENSMUSG00000043004	Transcript	ENSMUST00000159073	protein_coding	rs30673412
14:19879762-19879762	intron_variant	MODIFIER	Gng2	ENSMUSG00000043004	Transcript	ENSMUST00000160013	protein_coding	rs30673412
14:19879762-19879762	intron_variant	MODIFIER	Gng2	ENSMUSG00000043004	Transcript	ENSMUST00000161247	protein_coding	rs30673412
14:19879762-19879762	intron_variant	MODIFIER	Gng2	ENSMUSG00000043004	Transcript	ENSMUST00000162425	protein_coding	rs30673412
14:19884101-19884101	intron_variant	MODIFIER	Gng2	ENSMUSG00000043004	Transcript	ENSMUST00000055100	protein_coding	rs30688347
14:19884101-19884101	intron_variant	MODIFIER	Gng2	ENSMUSG00000043004	Transcript	ENSMUST00000159028	protein_coding	rs30688347
14:19884101-19884101	intron_variant	MODIFIER	Gng2	ENSMUSG00000043004	Transcript	ENSMUST00000159073	protein_coding	rs30688347
14:19884101-19884101	intron_variant	MODIFIER	Gng2	ENSMUSG00000043004	Transcript	ENSMUST00000160013	protein_coding	rs30688347
14:19884101-19884101	intron_variant	MODIFIER	Gng2	ENSMUSG00000043004	Transcript	ENSMUST00000161247	protein_coding	rs30688347
14:19884101-19884101	intron_variant	MODIFIER	Gng2	ENSMUSG00000043004	Transcript	ENSMUST00000162425	protein_coding	rs30688347
14:27000870-27000870	missense_variant	MODERATE	Hesx1	ENSMUSG00000040726	Transcript	ENSMUST00000035433	protein_coding	rs30787589
14:27000870-27000870	missense_variant	MODERATE	Hesx1	ENSMUSG00000040726	Transcript	ENSMUST00000224331	protein_coding	rs30787589
14:27000870-27000870	regulatory_region_variant	MODIFIER	-	-	RegulatoryFeature	ENSMUSR00000346003	promoter	rs30787589
14:27001445-27001445	synonymous_variant	LOW	Hesx1	ENSMUSG00000040726	Transcript	ENSMUST00000035433	protein_coding	rs30786772
14:27001445-27001445	synonymous_variant	LOW	Hesx1	ENSMUSG00000040726	Transcript	ENSMUST00000224331	protein_coding	rs30786772
14:27001511-27001511	synonymous_variant	LOW	Hesx1	ENSMUSG00000040726	Transcript	ENSMUST00000035433	protein_coding	rs30786771
14:27001511-27001511	synonymous_variant	LOW	Hesx1	ENSMUSG00000040726	Transcript	ENSMUST00000224331	protein_coding	rs30786771
14:27001896-27001896	synonymous_variant	LOW	Hesx1	ENSMUSG00000040726	Transcript	ENSMUST00000035433	protein_coding	rs30786766
14:27001896-27001896	synonymous_variant	LOW	Hesx1	ENSMUSG00000040726	Transcript	ENSMUST00000224331	protein_coding	rs30786766
14:27002071-27002071	synonymous_variant	LOW	Hesx1	ENSMUSG00000040726	Transcript	ENSMUST00000035433	protein_coding	rs47195088
14:27002071-27002071	synonymous_variant	LOW	Hesx1	ENSMUSG00000040726	Transcript	ENSMUST00000224331	protein_coding	rs47195088
14:27002073-27002073	missense_variant	MODERATE	Hesx1	ENSMUSG00000040726	Transcript	ENSMUST00000035433	protein_coding	rs49120517
14:27002073-27002073	missense_variant	MODERATE	Hesx1	ENSMUSG00000040726	Transcript	ENSMUST00000224331	protein_coding	rs49120517
14:27002801-27002801	downstream_gene_variant	MODIFIER	Hesx1	ENSMUSG00000040726	Transcript	ENSMUST00000035433	protein_coding	rs30785940
14:27002801-27002801	downstream_gene_variant	MODIFIER	Hesx1	ENSMUSG00000040726	Transcript	ENSMUST00000224331	protein_coding	rs30785940
14:27003084-27003084	downstream_gene_variant	MODIFIER	Hesx1	ENSMUSG00000040726	Transcript	ENSMUST00000035433	protein_coding	rs260976678
14:27003084-27003084	downstream_gene_variant	MODIFIER	Hesx1	ENSMUSG00000040726	Transcript	ENSMUST00000224331	protein_coding	rs260976678
14:31366680-31366680	intron_variant	MODIFIER	Capn7	ENSMUSG00000021893	Transcript	ENSMUST00000022451	protein_coding	rs16797100
14:31366680-31366680	intron_variant,NMD_transcript_v	MODIFIER	Sh3bp5	ENSMUSG00000021892	Transcript	ENSMUST00000140002	nonsense_mediated_decay	rs16797100
14:31366680-31366680	intron_variant,NMD_transcript_v	MODIFIER	Capn7	ENSMUSG00000021893	Transcript	ENSMUST00000143472	nonsense_mediated_decay	rs16797100
14:31366680-31366680	intron_variant,NMD_transcript_v	MODIFIER	Capn7	ENSMUSG00000021893	Transcript	ENSMUST00000152182	nonsense_mediated_decay	rs16797100
14:31366680-31366680	intron_variant,non_coding_trans	MODIFIER	Capn7	ENSMUSG00000021893	Transcript	ENSMUST00000228237	retained_intron	rs16797100
14:31377107-31377107	intron_variant	MODIFIER	Sh3bp5	ENSMUSG00000021892	Transcript	ENSMUST00000091903	protein_coding	rs47944161
14:31377107-31377107	intron_variant	MODIFIER	Sh3bp5	ENSMUSG00000021892	Transcript	ENSMUST00000100730	protein_coding	rs47944161
14:31377107-31377107	intron_variant,NMD_transcript_v	MODIFIER	Sh3bp5	ENSMUSG00000021892	Transcript	ENSMUST00000140002	nonsense_mediated_decay	rs47944161
14:31377107-31377107	downstream_gene_variant	MODIFIER	Sh3bp5	ENSMUSG00000021892	Transcript	ENSMUST00000147586	processed_transcript	rs47944161
15:55255684-55255684	downstream_gene_variant	MODIFIER	Deptor	ENSMUSG00000022419	Transcript	ENSMUST00000023056	protein_coding	-
15:55255684-55255684	3_prime_UTR_variant	MODIFIER	Deptor	ENSMUSG00000022419	Transcript	ENSMUST00000096433	protein_coding	-
15:85371021-85371021	intron_variant	MODIFIER	Atxn10	ENSMUSG00000016541	Transcript	ENSMUST00000163242	protein_coding	rs580772718
15:95941990-95941990	intron_variant	MODIFIER	Ano6	ENSMUSG00000064210	Transcript	ENSMUST00000071874	protein_coding	rs32217448
15:95941990-95941990	intron_variant,non_coding_trans	MODIFIER	Ano6	ENSMUSG00000064210	Transcript	ENSMUST00000226761	retained_intron	rs32217448
15:95941990-95941990	intron_variant,NMD_transcript_v	MODIFIER	Ano6	ENSMUSG00000064210	Transcript	ENSMUST00000227151	nonsense_mediated_decay	rs32217448
15:95941990-95941990	intron_variant	MODIFIER	Ano6	ENSMUSG00000064210	Transcript	ENSMUST00000227791	protein_coding	rs32217448

15:96584229-96584229	intron_variant	MODIFIER	Slc38a1	ENSMUSG00000023169	Transcript	ENSMUST00000088452	protein_coding	rs50190857
15:96584229-96584229	intron_variant	MODIFIER	Slc38a1	ENSMUSG00000023169	Transcript	ENSMUST00000088454	protein_coding	rs50190857
15:96584229-96584229	intron_variant	MODIFIER	Slc38a1	ENSMUSG00000023169	Transcript	ENSMUST00000100262	protein_coding	rs50190857
15:96584229-96584229	upstream_gene_variant	MODIFIER	Slc38a1	ENSMUSG00000023169	Transcript	ENSMUST00000230607	retained_intron	rs50190857
15:96584229-96584229	non_coding_transcript_exon_vai	MODIFIER	Slc38a1	ENSMUSG00000023169	Transcript	ENSMUST00000230756	retained_intron	rs50190857
15:96589941-96589941	intron_variant	MODIFIER	Slc38a1	ENSMUSG00000023169	Transcript	ENSMUST00000088452	protein_coding	rs49027448
15:96589941-96589941	intron_variant	MODIFIER	Slc38a1	ENSMUSG00000023169	Transcript	ENSMUST00000088454	protein_coding	rs49027448
15:96589941-96589941	intron_variant	MODIFIER	Slc38a1	ENSMUSG00000023169	Transcript	ENSMUST00000100262	protein_coding	rs49027448
15:96589941-96589941	intron_variant,non_coding_trans	MODIFIER	Slc38a1	ENSMUSG00000023169	Transcript	ENSMUST00000230756	retained_intron	rs49027448
15:96638321-96638321	intron_variant	MODIFIER	Slc38a1	ENSMUSG00000023169	Transcript	ENSMUST00000088452	protein_coding	rs31878648
15:96638321-96638321	intron_variant	MODIFIER	Slc38a1	ENSMUSG00000023169	Transcript	ENSMUST00000088454	protein_coding	rs31878648
15:96638321-96638321	intron_variant	MODIFIER	Slc38a1	ENSMUSG00000023169	Transcript	ENSMUST00000100262	protein_coding	rs31878648
15:96638321-96638321	downstream_gene_variant	MODIFIER	Slc38a1	ENSMUSG00000023169	Transcript	ENSMUST00000229253	retained_intron	rs31878648
15:96638321-96638321	intron_variant,non_coding_trans	MODIFIER	Slc38a1	ENSMUSG00000023169	Transcript	ENSMUST00000230756	retained_intron	rs31878648
15:96638321-96638321	intron_variant	MODIFIER	Slc38a1	ENSMUSG00000023169	Transcript	ENSMUST00000230767	protein_coding	rs31878648
15:96643369-96643369	upstream_gene_variant	MODIFIER	Slc38a1	ENSMUSG00000023169	Transcript	ENSMUST00000088452	protein_coding	rs107608993
15:96643369-96643369	upstream_gene_variant	MODIFIER	Slc38a1	ENSMUSG00000023169	Transcript	ENSMUST00000088454	protein_coding	rs107608993
15:96643369-96643369	upstream_gene_variant	MODIFIER	Slc38a1	ENSMUSG00000023169	Transcript	ENSMUST00000100262	protein_coding	rs107608993
15:96643369-96643369	upstream_gene_variant	MODIFIER	Slc38a1	ENSMUSG00000023169	Transcript	ENSMUST00000229253	retained_intron	rs107608993
15:96643369-96643369	upstream_gene_variant	MODIFIER	Slc38a1	ENSMUSG00000023169	Transcript	ENSMUST00000230756	retained_intron	rs107608993
15:96643369-96643369	upstream_gene_variant	MODIFIER	Slc38a1	ENSMUSG00000023169	Transcript	ENSMUST00000230767	protein_coding	rs107608993
15:96643369-96643369	regulatory_region_variant	MODIFIER	-	-	RegulatoryFeature	ENSMUSR00000358205	promoter	rs107608993
15:96694872-96694872	intron_variant	MODIFIER	Slc38a2	ENSMUSG00000022462	Transcript	ENSMUST00000023099	protein_coding	rs32371387
15:96694872-96694872	non_coding_transcript_exon_vai	MODIFIER	Slc38a2	ENSMUSG00000022462	Transcript	ENSMUST00000229141	retained_intron	rs32371387
15:97801560-97801560	intron_variant	MODIFIER	Hdac7	ENSMUSG00000022475	Transcript	ENSMUST00000079838	protein_coding	rs32034330
15:97801560-97801560	intron_variant	MODIFIER	Hdac7	ENSMUSG00000022475	Transcript	ENSMUST00000088402	protein_coding	rs32034330
15:97801560-97801560	intron_variant	MODIFIER	Hdac7	ENSMUSG00000022475	Transcript	ENSMUST00000116408	protein_coding	rs32034330
15:97801560-97801560	intron_variant	MODIFIER	Hdac7	ENSMUSG00000022475	Transcript	ENSMUST00000116409	protein_coding	rs32034330
15:97801560-97801560	intron_variant	MODIFIER	Hdac7	ENSMUSG00000022475	Transcript	ENSMUST00000118294	protein_coding	rs32034330
15:97801560-97801560	intron_variant	MODIFIER	Hdac7	ENSMUSG00000022475	Transcript	ENSMUST00000119670	protein_coding	rs32034330
15:97801560-97801560	intron_variant	MODIFIER	Hdac7	ENSMUSG00000022475	Transcript	ENSMUST00000120683	protein_coding	rs32034330
15:97801560-97801560	intron_variant	MODIFIER	Hdac7	ENSMUSG00000022475	Transcript	ENSMUST00000121514	protein_coding	rs32034330
15:97801560-97801560	intron_variant	MODIFIER	Hdac7	ENSMUSG00000022475	Transcript	ENSMUST00000134258	protein_coding	rs32034330
15:97801560-97801560	intron_variant	MODIFIER	Hdac7	ENSMUSG00000022475	Transcript	ENSMUST00000135651	protein_coding	rs32034330
15:97801560-97801560	intron_variant,NMD_transcript_v	MODIFIER	Hdac7	ENSMUSG00000022475	Transcript	ENSMUST00000156045	nonsense_mediated_decay	rs32034330
15:97801560-97801560	non_coding_transcript_exon_vai	MODIFIER	Hdac7	ENSMUSG00000022475	Transcript	ENSMUST00000228466	retained_intron	rs32034330
15:97854782-97854782	3_prime_UTR_variant	MODIFIER	Vdr	ENSMUSG00000022479	Transcript	ENSMUST00000023119	protein_coding	rs16805377
15:97854782-97854782	downstream_gene_variant	MODIFIER	Vdr	ENSMUSG00000022479	Transcript	ENSMUST00000139656	retained_intron	rs16805377
15:97856825-97856825	3_prime_UTR_variant	MODIFIER	Vdr	ENSMUSG00000022479	Transcript	ENSMUST00000023119	protein_coding	rs13473169
15:97856825-97856825	downstream_gene_variant	MODIFIER	Vdr	ENSMUSG00000022479	Transcript	ENSMUST00000139656	retained_intron	rs13473169
15:97856826-97856826	3_prime_UTR_variant	MODIFIER	Vdr	ENSMUSG00000022479	Transcript	ENSMUST00000023119	protein_coding	rs16805410
15:97856826-97856826	downstream_gene_variant	MODIFIER	Vdr	ENSMUSG00000022479	Transcript	ENSMUST00000139656	retained_intron	rs16805410
15:98136655-98136655	downstream_gene_variant	MODIFIER	Pfkm	ENSMUSG00000033065	Transcript	ENSMUST00000051226	protein_coding	rs31893433
15:98136655-98136655	intron_variant	MODIFIER	Asb8	ENSMUSG00000048175	Transcript	ENSMUST00000059112	protein_coding	rs31893433
15:98136655-98136655	intron_variant	MODIFIER	Asb8	ENSMUSG00000048175	Transcript	ENSMUST00000123626	protein_coding	rs31893433
15:98136655-98136655	intron_variant	MODIFIER	Asb8	ENSMUSG00000048175	Transcript	ENSMUST00000123922	protein_coding	rs31893433
15:98136655-98136655	intron_variant	MODIFIER	Asb8	ENSMUSG00000048175	Transcript	ENSMUST00000143400	protein_coding	rs31893433
15:98136655-98136655	downstream_gene_variant	MODIFIER	Pfkm	ENSMUSG00000033065	Transcript	ENSMUST00000163507	protein_coding	rs31893433
15:98136655-98136655	downstream_gene_variant	MODIFIER	Pfkm	ENSMUSG00000033065	Transcript	ENSMUST00000230445	protein_coding	rs31893433
15:99405992-99405992	intron_variant	MODIFIER	Tmbim6	ENSMUSG00000023010	Transcript	ENSMUST00000023749	protein_coding	rs51545327
15:99405992-99405992	intron_variant	MODIFIER	Tmbim6	ENSMUSG00000023010	Transcript	ENSMUST00000159209	protein_coding	rs51545327
15:99405992-99405992	intron_variant	MODIFIER	Tmbim6	ENSMUSG00000023010	Transcript	ENSMUST00000159531	protein_coding	rs51545327
15:99405992-99405992	non_coding_transcript_exon_vai	MODIFIER	Tmbim6	ENSMUSG00000023010	Transcript	ENSMUST00000160454	retained_intron	rs51545327
15:99405992-99405992	intron_variant	MODIFIER	Tmbim6	ENSMUSG00000023010	Transcript	ENSMUST00000160635	protein_coding	rs51545327
15:99405992-99405992	intron_variant	MODIFIER	Tmbim6	ENSMUSG00000023010	Transcript	ENSMUST00000161250	protein_coding	rs51545327
15:99405992-99405992	intron_variant	MODIFIER	Tmbim6	ENSMUSG00000023010	Transcript	ENSMUST00000161778	protein_coding	rs51545327
15:99405992-99405992	downstream_gene_variant	MODIFIER	Tmbim6	ENSMUSG00000023010	Transcript	ENSMUST00000162274	protein_coding	rs51545327
15:99405992-99405992	intron_variant	MODIFIER	Tmbim6	ENSMUSG00000023010	Transcript	ENSMUST00000162624	protein_coding	rs51545327
15:99405992-99405992	intron_variant	MODIFIER	Tmbim6	ENSMUSG00000023010	Transcript	ENSMUST00000229392	protein_coding	rs51545327
15:99405992-99405992	intron_variant,non_coding_trans	MODIFIER	Tmbim6	ENSMUSG00000023010	Transcript	ENSMUST00000231147	retained_intron	rs51545327
15:99405992-99405992	upstream_gene_variant	MODIFIER	Tmbim6	ENSMUSG00000023010	Transcript	ENSMUST00000231173	protein_coding	rs51545327
15:99422483-99422483	3_prime_UTR_variant	MODIFIER	Nckap5l	ENSMUSG00000023009	Transcript	ENSMUST00000023747	protein_coding	rs225319147
15:99422483-99422483	downstream_gene_variant	MODIFIER	Nckap5l	ENSMUSG00000023009	Transcript	ENSMUST00000160500	processed_transcript	rs225319147
15:99422483-99422483	downstream_gene_variant	MODIFIER	Nckap5l	ENSMUSG00000023009	Transcript	ENSMUST00000161004	protein_coding	rs225319147
15:99422483-99422483	regulatory_region_variant	MODIFIER	-	-	RegulatoryFeature	ENSMUSR00000105865	promoter_flanking_region	rs225319147
15:99425633-99425633	synonymous_variant	LOW	Nckap5l	ENSMUSG00000023009	Transcript	ENSMUST00000023747	protein_coding	rs13482737
15:99425633-99425633	upstream_gene_variant	MODIFIER	Nckap5los	ENSMUSG00000089968	Transcript	ENSMUST00000159738	antisense	rs13482737
15:99425633-99425633	upstream_gene_variant	MODIFIER	Nckap5l	ENSMUSG00000023009	Transcript	ENSMUST00000160500	processed_transcript	rs13482737
15:99425633-99425633	upstream_gene_variant	MODIFIER	Nckap5l	ENSMUSG00000023009	Transcript	ENSMUST00000161004	protein_coding	rs13482737

15:99425633-99425633	downstream_gene_variant	MODIFIER	Nckap5l	ENSMUSG00000023009	Transcript	ENSMUST00000161948	protein_coding	rs13482737
15:99425858-99425858	synonymous_variant	LOW	Nckap5l	ENSMUSG00000023009	Transcript	ENSMUST00000023747	protein_coding	rs32022082
15:99425858-99425858	upstream_gene_variant	MODIFIER	Nckap5los	ENSMUSG00000089968	Transcript	ENSMUST00000159738	antisense	rs32022082
15:99425858-99425858	upstream_gene_variant	MODIFIER	Nckap5l	ENSMUSG00000023009	Transcript	ENSMUST00000160500	processed_transcript	rs32022082
15:99425858-99425858	upstream_gene_variant	MODIFIER	Nckap5l	ENSMUSG00000023009	Transcript	ENSMUST00000161004	protein_coding	rs32022082
15:99425858-99425858	downstream_gene_variant	MODIFIER	Nckap5l	ENSMUSG00000023009	Transcript	ENSMUST00000161948	protein_coding	rs32022082
16:11203472-11203472	upstream_gene_variant	MODIFIER	Rsl1d1	ENSMUSG00000005846	Transcript	ENSMUST00000119953	protein_coding	-
16:11203472-11203472	upstream_gene_variant	MODIFIER	Rsl1d1	ENSMUSG00000005846	Transcript	ENSMUST00000124454	retained_intron	-
16:11203472-11203472	non_coding_transcript_exon_vai	MODIFIER	2610020C07Rik	ENSMUSG00000097537	Transcript	ENSMUST00000181526	antisense	-
16:11203472-11203472	upstream_gene_variant	MODIFIER	2610020C07Rik	ENSMUSG00000097537	Transcript	ENSMUST00000229756	antisense	-
16:11203472-11203472	upstream_gene_variant	MODIFIER	Rsl1d1	ENSMUSG00000005846	Transcript	ENSMUST00000230002	protein_coding	-
16:11203472-11203472	upstream_gene_variant	MODIFIER	2610020C07Rik	ENSMUSG00000097537	Transcript	ENSMUST00000230166	antisense	-
16:11203472-11203472	upstream_gene_variant	MODIFIER	Rsl1d1	ENSMUSG00000005846	Transcript	ENSMUST00000230232	nonsense_mediated_decay	-
16:11203472-11203472	upstream_gene_variant	MODIFIER	2610020C07Rik	ENSMUSG00000097537	Transcript	ENSMUST00000230245	antisense	-
16:11203472-11203472	regulatory_region_variant	MODIFIER	-	-	RegulatoryFeature	ENSMUSR00000107727	promoter	-
16:55865709-55865709	intron_variant	MODIFIER	Nxpe3	ENSMUSG00000075033	Transcript	ENSMUST00000099705	protein_coding	rs107935613
16:64762269-64762269	3_prime_UTR_variant	MODIFIER	4930453N24Rik	ENSMUSG00000059920	Transcript	ENSMUST00000076991	protein_coding	-
16:64762269-64762269	intron_variant,non_coding_trans	MODIFIER	4930453N24Rik	ENSMUSG00000059920	Transcript	ENSMUST00000162732	processed_transcript	-
16:75601406-75601406	intron_variant	MODIFIER	Rbm11	ENSMUSG00000032940	Transcript	ENSMUST00000046378	protein_coding	-
16:75601406-75601406	3_prime_UTR_variant	MODIFIER	Rbm11	ENSMUSG00000032940	Transcript	ENSMUST00000114249	protein_coding	-
16:75601406-75601406	3_prime_UTR_variant	MODIFIER	Rbm11	ENSMUSG00000032940	Transcript	ENSMUST00000114253	protein_coding	-
16:97463249-97463249	upstream_gene_variant	MODIFIER	Mx1	ENSMUSG00000000386	Transcript	ENSMUST00000023655	protein_coding	rs47985692
16:97463249-97463249	upstream_gene_variant	MODIFIER	Mx1	ENSMUSG00000000386	Transcript	ENSMUST00000113768	protein_coding	rs47985692
16:97463249-97463249	upstream_gene_variant	MODIFIER	Mx1	ENSMUSG00000000386	Transcript	ENSMUST00000135184	nonsense_mediated_decay	rs47985692
16:97463249-97463249	upstream_gene_variant	MODIFIER	Mx1	ENSMUSG00000000386	Transcript	ENSMUST00000142883	protein_coding	rs47985692
16:97463249-97463249	upstream_gene_variant	MODIFIER	Mx1	ENSMUSG00000000386	Transcript	ENSMUST00000155233	nonsense_mediated_decay	rs47985692
16:97463249-97463249	downstream_gene_variant	MODIFIER	AC164088.2	ENSMUSG00000116929	Transcript	ENSMUST00000231393	TEC	rs47985692
16:97463249-97463249	upstream_gene_variant	MODIFIER	Mx1	ENSMUSG00000000386	Transcript	ENSMUST00000231455	retained_intron	rs47985692
16:97463249-97463249	upstream_gene_variant	MODIFIER	Mx1	ENSMUSG00000000386	Transcript	ENSMUST00000231461	processed_transcript	rs47985692
16:97463249-97463249	upstream_gene_variant	MODIFIER	Mx1	ENSMUSG00000000386	Transcript	ENSMUST00000232193	protein_coding	rs47985692
16:97463249-97463249	upstream_gene_variant	MODIFIER	Mx1	ENSMUSG00000000386	Transcript	ENSMUST00000232282	nonsense_mediated_decay	rs47985692
17:14099574-14099575	regulatory_region_variant	MODIFIER	-	-	RegulatoryFeature	ENSMUSR00000591644	promoter_flanking_region	-
17:14099574-14099575	intergenic_variant	MODIFIER	-	-	-	-	-	-
17:31433176-31433176	intron_variant	MODIFIER	Pde9a	ENSMUSG00000041119	Transcript	ENSMUST00000047168	protein_coding	rs108457086
17:31433176-31433176	intron_variant,NMD_transcript_v	MODIFIER	Pde9a	ENSMUSG00000041119	Transcript	ENSMUST00000124902	nonsense_mediated_decay	rs108457086
17:31433176-31433176	intron_variant	MODIFIER	Pde9a	ENSMUSG00000041119	Transcript	ENSMUST00000127929	protein_coding	rs108457086
17:31433176-31433176	intron_variant,NMD_transcript_v	MODIFIER	Pde9a	ENSMUSG00000041119	Transcript	ENSMUST00000131417	nonsense_mediated_decay	rs108457086
17:31433176-31433176	intron_variant	MODIFIER	Pde9a	ENSMUSG00000041119	Transcript	ENSMUST00000134525	protein_coding	rs108457086
17:31433176-31433176	5_prime_UTR_variant	MODIFIER	Pde9a	ENSMUSG00000041119	Transcript	ENSMUST00000136384	protein_coding	rs108457086
17:31433176-31433176	intron_variant,NMD_transcript_v	MODIFIER	Pde9a	ENSMUSG00000041119	Transcript	ENSMUST00000137927	nonsense_mediated_decay	rs108457086
17:31433176-31433176	intron_variant,NMD_transcript_v	MODIFIER	Pde9a	ENSMUSG00000041119	Transcript	ENSMUST00000141314	nonsense_mediated_decay	rs108457086
17:31433176-31433176	intron_variant,NMD_transcript_v	MODIFIER	Pde9a	ENSMUSG00000041119	Transcript	ENSMUST00000143549	nonsense_mediated_decay	rs108457086
17:31433176-31433176	intron_variant,non_coding_trans	MODIFIER	Pde9a	ENSMUSG00000041119	Transcript	ENSMUST00000154567	retained_intron	rs108457086
17:31433176-31433176	regulatory_region_variant	MODIFIER	-	-	RegulatoryFeature	ENSMUSR00000121934	promoter	rs108457086
17:31467198-31467198	intron_variant	MODIFIER	Pde9a	ENSMUSG00000041119	Transcript	ENSMUST00000047168	protein_coding	rs29520998
17:31467198-31467198	intron_variant,NMD_transcript_v	MODIFIER	Pde9a	ENSMUSG00000041119	Transcript	ENSMUST00000124902	nonsense_mediated_decay	rs29520998
17:31467198-31467198	intron_variant	MODIFIER	Pde9a	ENSMUSG00000041119	Transcript	ENSMUST00000127929	protein_coding	rs29520998
17:31467198-31467198	intron_variant,NMD_transcript_v	MODIFIER	Pde9a	ENSMUSG00000041119	Transcript	ENSMUST00000131417	nonsense_mediated_decay	rs29520998
17:31467198-31467198	intron_variant	MODIFIER	Pde9a	ENSMUSG00000041119	Transcript	ENSMUST00000134525	protein_coding	rs29520998
17:31467198-31467198	intron_variant,NMD_transcript_v	MODIFIER	Pde9a	ENSMUSG00000041119	Transcript	ENSMUST00000137927	nonsense_mediated_decay	rs29520998
17:31467198-31467198	intron_variant,NMD_transcript_v	MODIFIER	Pde9a	ENSMUSG00000041119	Transcript	ENSMUST00000143549	nonsense_mediated_decay	rs29520998
17:31467198-31467198	intron_variant	MODIFIER	Pde9a	ENSMUSG00000041119	Transcript	ENSMUST00000154392	protein_coding	rs29520998
17:31467198-31467198	intron_variant,non_coding_trans	MODIFIER	Pde9a	ENSMUSG00000041119	Transcript	ENSMUST00000154567	retained_intron	rs29520998
17:31467198-31467198	upstream_gene_variant	MODIFIER	Pde9a	ENSMUSG00000041119	Transcript	ENSMUST00000155113	processed_transcript	rs29520998
17:31467198-31467198	regulatory_region_variant	MODIFIER	-	-	RegulatoryFeature	ENSMUSR00000368660	CTCF_binding_site	rs29520998
17:31467564-31467564	intron_variant	MODIFIER	Pde9a	ENSMUSG00000041119	Transcript	ENSMUST00000047168	protein_coding	rs33679962
17:31467564-31467564	intron_variant,NMD_transcript_v	MODIFIER	Pde9a	ENSMUSG00000041119	Transcript	ENSMUST00000124902	nonsense_mediated_decay	rs33679962
17:31467564-31467564	intron_variant	MODIFIER	Pde9a	ENSMUSG00000041119	Transcript	ENSMUST00000127929	protein_coding	rs33679962
17:31467564-31467564	intron_variant,NMD_transcript_v	MODIFIER	Pde9a	ENSMUSG00000041119	Transcript	ENSMUST00000131417	nonsense_mediated_decay	rs33679962
17:31467564-31467564	intron_variant	MODIFIER	Pde9a	ENSMUSG00000041119	Transcript	ENSMUST00000134525	protein_coding	rs33679962
17:31467564-31467564	intron_variant,NMD_transcript_v	MODIFIER	Pde9a	ENSMUSG00000041119	Transcript	ENSMUST00000137927	nonsense_mediated_decay	rs33679962
17:31467564-31467564	intron_variant,NMD_transcript_v	MODIFIER	Pde9a	ENSMUSG00000041119	Transcript	ENSMUST00000143549	nonsense_mediated_decay	rs33679962
17:31467564-31467564	intron_variant	MODIFIER	Pde9a	ENSMUSG00000041119	Transcript	ENSMUST00000154392	protein_coding	rs33679962
17:31467564-31467564	intron_variant,non_coding_trans	MODIFIER	Pde9a	ENSMUSG00000041119	Transcript	ENSMUST00000154567	retained_intron	rs33679962
17:31467564-31467564	upstream_gene_variant	MODIFIER	Pde9a	ENSMUSG00000041119	Transcript	ENSMUST00000155113	processed_transcript	rs33679962
17:31469366-31469366	intron_variant	MODIFIER	Pde9a	ENSMUSG00000041119	Transcript	ENSMUST00000047168	protein_coding	rs51138857
17:31469366-31469366	intron_variant,NMD_transcript_v	MODIFIER	Pde9a	ENSMUSG00000041119	Transcript	ENSMUST00000124902	nonsense_mediated_decay	rs51138857
17:31469366-31469366	intron_variant	MODIFIER	Pde9a	ENSMUSG00000041119	Transcript	ENSMUST00000127929	protein_coding	rs51138857
17:31469366-31469366	intron_variant,NMD_transcript_v	MODIFIER	Pde9a	ENSMUSG00000041119	Transcript	ENSMUST00000131417	nonsense_mediated_decay	rs51138857

17:31469366-31469366	intron_variant	MODIFIER	Pde9a	ENSMUSG00000041119	Transcript	ENSMUST00000134525	protein_coding	rs51138857
17:31469366-31469366	intron_variant,NMD_transcript_v	MODIFIER	Pde9a	ENSMUSG00000041119	Transcript	ENSMUST00000137927	nonsense_mediated_decay	rs51138857
17:31469366-31469366	intron_variant,NMD_transcript_v	MODIFIER	Pde9a	ENSMUSG00000041119	Transcript	ENSMUST00000143549	nonsense_mediated_decay	rs51138857
17:31469366-31469366	intron_variant	MODIFIER	Pde9a	ENSMUSG00000041119	Transcript	ENSMUST00000154392	protein_coding	rs51138857
17:31469366-31469366	intron_variant,non_coding_trans	MODIFIER	Pde9a	ENSMUSG00000041119	Transcript	ENSMUST00000154567	retained_intron	rs51138857
17:31469366-31469366	upstream_gene_variant	MODIFIER	Pde9a	ENSMUSG00000041119	Transcript	ENSMUST00000155113	processed_transcript	rs51138857
17:31469381-31469381	intron_variant	MODIFIER	Pde9a	ENSMUSG00000041119	Transcript	ENSMUST00000047168	protein_coding	rs33130940
17:31469381-31469381	intron_variant,NMD_transcript_v	MODIFIER	Pde9a	ENSMUSG00000041119	Transcript	ENSMUST00000124902	nonsense_mediated_decay	rs33130940
17:31469381-31469381	intron_variant	MODIFIER	Pde9a	ENSMUSG00000041119	Transcript	ENSMUST00000127929	protein_coding	rs33130940
17:31469381-31469381	intron_variant,NMD_transcript_v	MODIFIER	Pde9a	ENSMUSG00000041119	Transcript	ENSMUST00000131417	nonsense_mediated_decay	rs33130940
17:31469381-31469381	intron_variant	MODIFIER	Pde9a	ENSMUSG00000041119	Transcript	ENSMUST00000134525	protein_coding	rs33130940
17:31469381-31469381	intron_variant,NMD_transcript_v	MODIFIER	Pde9a	ENSMUSG00000041119	Transcript	ENSMUST00000137927	nonsense_mediated_decay	rs33130940
17:31469381-31469381	intron_variant,NMD_transcript_v	MODIFIER	Pde9a	ENSMUSG00000041119	Transcript	ENSMUST00000143549	nonsense_mediated_decay	rs33130940
17:31469381-31469381	intron_variant	MODIFIER	Pde9a	ENSMUSG00000041119	Transcript	ENSMUST00000154392	protein_coding	rs33130940
17:31469381-31469381	intron_variant,non_coding_trans	MODIFIER	Pde9a	ENSMUSG00000041119	Transcript	ENSMUST00000154567	retained_intron	rs33130940
17:31469381-31469381	upstream_gene_variant	MODIFIER	Pde9a	ENSMUSG00000041119	Transcript	ENSMUST00000155113	processed_transcript	rs33130940
17:31469437-31469437	intron_variant	MODIFIER	Pde9a	ENSMUSG00000041119	Transcript	ENSMUST00000047168	protein_coding	rs50117613
17:31469437-31469437	intron_variant,NMD_transcript_v	MODIFIER	Pde9a	ENSMUSG00000041119	Transcript	ENSMUST00000124902	nonsense_mediated_decay	rs50117613
17:31469437-31469437	intron_variant	MODIFIER	Pde9a	ENSMUSG00000041119	Transcript	ENSMUST00000127929	protein_coding	rs50117613
17:31469437-31469437	intron_variant,NMD_transcript_v	MODIFIER	Pde9a	ENSMUSG00000041119	Transcript	ENSMUST00000131417	nonsense_mediated_decay	rs50117613
17:31469437-31469437	intron_variant	MODIFIER	Pde9a	ENSMUSG00000041119	Transcript	ENSMUST00000134525	protein_coding	rs50117613
17:31469437-31469437	intron_variant,NMD_transcript_v	MODIFIER	Pde9a	ENSMUSG00000041119	Transcript	ENSMUST00000137927	nonsense_mediated_decay	rs50117613
17:31469437-31469437	intron_variant,NMD_transcript_v	MODIFIER	Pde9a	ENSMUSG00000041119	Transcript	ENSMUST00000143549	nonsense_mediated_decay	rs50117613
17:31469437-31469437	intron_variant	MODIFIER	Pde9a	ENSMUSG00000041119	Transcript	ENSMUST00000154392	protein_coding	rs50117613
17:31469437-31469437	intron_variant,non_coding_trans	MODIFIER	Pde9a	ENSMUSG00000041119	Transcript	ENSMUST00000154567	retained_intron	rs50117613
17:31469437-31469437	upstream_gene_variant	MODIFIER	Pde9a	ENSMUSG00000041119	Transcript	ENSMUST00000155113	processed_transcript	rs50117613
17:31469755-31469755	intron_variant	MODIFIER	Pde9a	ENSMUSG00000041119	Transcript	ENSMUST00000047168	protein_coding	rs46553420
17:31469755-31469755	intron_variant,NMD_transcript_v	MODIFIER	Pde9a	ENSMUSG00000041119	Transcript	ENSMUST00000124902	nonsense_mediated_decay	rs46553420
17:31469755-31469755	intron_variant	MODIFIER	Pde9a	ENSMUSG00000041119	Transcript	ENSMUST00000127929	protein_coding	rs46553420
17:31469755-31469755	intron_variant,NMD_transcript_v	MODIFIER	Pde9a	ENSMUSG00000041119	Transcript	ENSMUST00000131417	nonsense_mediated_decay	rs46553420
17:31469755-31469755	intron_variant	MODIFIER	Pde9a	ENSMUSG00000041119	Transcript	ENSMUST00000134525	protein_coding	rs46553420
17:31469755-31469755	intron_variant,NMD_transcript_v	MODIFIER	Pde9a	ENSMUSG00000041119	Transcript	ENSMUST00000137927	nonsense_mediated_decay	rs46553420
17:31469755-31469755	intron_variant,NMD_transcript_v	MODIFIER	Pde9a	ENSMUSG00000041119	Transcript	ENSMUST00000143549	nonsense_mediated_decay	rs46553420
17:31469755-31469755	intron_variant	MODIFIER	Pde9a	ENSMUSG00000041119	Transcript	ENSMUST00000154392	protein_coding	rs46553420
17:31469755-31469755	intron_variant,non_coding_trans	MODIFIER	Pde9a	ENSMUSG00000041119	Transcript	ENSMUST00000154567	retained_intron	rs46553420
17:31469755-31469755	upstream_gene_variant	MODIFIER	Pde9a	ENSMUSG00000041119	Transcript	ENSMUST00000155113	processed_transcript	rs46553420
17:31471362-31471362	intron_variant	MODIFIER	Pde9a	ENSMUSG00000041119	Transcript	ENSMUST00000047168	protein_coding	rs48282767
17:31471362-31471362	intron_variant,NMD_transcript_v	MODIFIER	Pde9a	ENSMUSG00000041119	Transcript	ENSMUST00000124902	nonsense_mediated_decay	rs48282767
17:31471362-31471362	intron_variant	MODIFIER	Pde9a	ENSMUSG00000041119	Transcript	ENSMUST00000127929	protein_coding	rs48282767
17:31471362-31471362	intron_variant,NMD_transcript_v	MODIFIER	Pde9a	ENSMUSG00000041119	Transcript	ENSMUST00000131417	nonsense_mediated_decay	rs48282767
17:31471362-31471362	intron_variant	MODIFIER	Pde9a	ENSMUSG00000041119	Transcript	ENSMUST00000134525	protein_coding	rs48282767
17:31471362-31471362	intron_variant,NMD_transcript_v	MODIFIER	Pde9a	ENSMUSG00000041119	Transcript	ENSMUST00000137927	nonsense_mediated_decay	rs48282767
17:31471362-31471362	intron_variant,NMD_transcript_v	MODIFIER	Pde9a	ENSMUSG00000041119	Transcript	ENSMUST00000143549	nonsense_mediated_decay	rs48282767
17:31471362-31471362	intron_variant	MODIFIER	Pde9a	ENSMUSG00000041119	Transcript	ENSMUST00000154392	protein_coding	rs48282767
17:31471362-31471362	intron_variant,non_coding_trans	MODIFIER	Pde9a	ENSMUSG00000041119	Transcript	ENSMUST00000154567	retained_intron	rs48282767
17:31471362-31471362	intron_variant,non_coding_trans	MODIFIER	Pde9a	ENSMUSG00000041119	Transcript	ENSMUST00000155113	processed_transcript	rs48282767
17:31471362-31471362	regulatory_region_variant	MODIFIER	-	-	RegulatoryFeature	ENSMUSR00000594165	open_chromatin_region	rs48282767
17:3310923-3310923	downstream_gene_variant	MODIFIER	AC165953.1	ENSMUSG00000116838	Transcript	ENSMUST00000231500	processed_pseudogene	rs108618004
17:3310923-3310923	downstream_gene_variant	MODIFIER	AC165953.2	ENSMUSG00000116858	Transcript	ENSMUST00000231987	lincRNA	rs108618004
17:3310923-3310923	non_coding_transcript_exon_va	MODIFIER	AC165953.2	ENSMUSG00000116858	Transcript	ENSMUST00000232042	lincRNA	rs108618004
17:33304468-33304468	downstream_gene_variant	MODIFIER	Zfp955b	ENSMUSG00000096910	Transcript	ENSMUST00000099414	protein_coding	rs33335748
17:33304468-33304468	downstream_gene_variant	MODIFIER	Zfp422-ps	ENSMUSG00000091515	Transcript	ENSMUST00000182230	unprocessed_pseudogene	rs33335748
17:33304481-33304481	downstream_gene_variant	MODIFIER	Zfp955b	ENSMUSG00000096910	Transcript	ENSMUST00000099414	protein_coding	rs29520403
17:33304481-33304481	downstream_gene_variant	MODIFIER	Zfp422-ps	ENSMUSG00000091515	Transcript	ENSMUST00000182230	unprocessed_pseudogene	rs29520403
17:39845013-39845013	upstream_gene_variant	MODIFIER	Gm42418	ENSMUSG00000098178	Transcript	ENSMUST00000182010	lincRNA	-
17:39845013-39845013	upstream_gene_variant	MODIFIER	Gm26917	ENSMUSG00000097971	Transcript	ENSMUST00000182520	lincRNA	-
17:39845013-39845013	non_coding_transcript_exon_va	MODIFIER	Gm26917	ENSMUSG00000097971	Transcript	ENSMUST00000192833	lincRNA	-
17:39845013-39845013	downstream_gene_variant	MODIFIER	CT010467.1	ENSMUSG00000106106	Transcript	ENSMUST00000198477	rRNA	-
17:39845013-39845013	downstream_gene_variant	MODIFIER	AY036118	ENSMUSG00000105361	Transcript	ENSMUST00000200021	lincRNA	-
17:39845013-39845013	downstream_gene_variant	MODIFIER	CT010467.1	ENSMUSG00000106106	Transcript	ENSMUST00000205406	pseudogene	-
17:39845013-39845013	regulatory_region_variant	MODIFIER	-	-	RegulatoryFeature	ENSMUSR00000595179	promoter_flanking_region	-
17:39848110-39848110	non_coding_transcript_exon_va	MODIFIER	Gm42418	ENSMUSG00000098178	Transcript	ENSMUST00000182010	lincRNA	rs108312641
17:39848110-39848110	downstream_gene_variant	MODIFIER	Gm26917	ENSMUSG00000097971	Transcript	ENSMUST00000182520	lincRNA	rs108312641
17:39848110-39848110	downstream_gene_variant	MODIFIER	Gm26917	ENSMUSG00000097971	Transcript	ENSMUST00000192833	lincRNA	rs108312641
17:39848110-39848110	non_coding_transcript_exon_va	MODIFIER	CT010467.1	ENSMUSG00000106106	Transcript	ENSMUST00000198477	rRNA	rs108312641
17:39848110-39848110	upstream_gene_variant	MODIFIER	AY036118	ENSMUSG00000105361	Transcript	ENSMUST00000200021	lincRNA	rs108312641
17:39848110-39848110	non_coding_transcript_exon_va	MODIFIER	CT010467.1	ENSMUSG00000106106	Transcript	ENSMUST00000205406	pseudogene	rs108312641
17:39848110-39848110	regulatory_region_variant	MODIFIER	-	-	RegulatoryFeature	ENSMUSR00000369412	CTCF_binding_site	rs108312641
17:39848110-39848110	regulatory_region_variant	MODIFIER	-	-	RegulatoryFeature	ENSMUSR00000595179	promoter_flanking_region	rs108312641

17:56176004-56176004	3_prime_UTR_variant	MODIFIER	Mydgf	ENSMUSG00000019579	Transcript	ENSMUST00000019723	protein_coding	rs864305593
17:56176004-56176004	downstream_gene_variant	MODIFIER	Tnfaip8l1	ENSMUSG00000044469	Transcript	ENSMUST00000077788	protein_coding	rs864305593
17:56176004-56176004	downstream_gene_variant	MODIFIER	Mydgf	ENSMUSG00000019579	Transcript	ENSMUST00000140885	retained_intron	rs864305593
17:56176004-56176004	downstream_gene_variant	MODIFIER	Mydgf	ENSMUSG00000019579	Transcript	ENSMUST00000151093	processed_transcript	rs864305593
17:56176004-56176004	upstream_gene_variant	MODIFIER	Gm44397	ENSMUSG00000106418	Transcript	ENSMUST00000195950	miRNA	rs864305593
17:71228688-71228688	intron_variant	MODIFIER	Lpin2	ENSMUSG00000024052	Transcript	ENSMUST00000126681	protein_coding	rs33533179
17:71228688-71228688	intron_variant	MODIFIER	Lpin2	ENSMUSG00000024052	Transcript	ENSMUST00000129635	protein_coding	rs33533179
17:71228688-71228688	upstream_gene_variant	MODIFIER	Lpin2	ENSMUSG00000024052	Transcript	ENSMUST00000130750	retained_intron	rs33533179
17:71228688-71228688	intron_variant,NMD_transcript_v	MODIFIER	Lpin2	ENSMUSG00000024052	Transcript	ENSMUST00000135589	nonsense_mediated_decay	rs33533179
17:71228688-71228688	intron_variant,non_coding_trans	MODIFIER	Lpin2	ENSMUSG00000024052	Transcript	ENSMUST00000142842	retained_intron	rs33533179
17:71228688-71228688	intron_variant	MODIFIER	Lpin2	ENSMUSG00000024052	Transcript	ENSMUST00000156570	protein_coding	rs33533179
17:71228688-71228688	intron_variant,non_coding_trans	MODIFIER	Gm26561	ENSMUSG00000097625	Transcript	ENSMUST00000180743	lincRNA	rs33533179
17:71254664-71254664	intron_variant	MODIFIER	Emilin2	ENSMUSG00000024053	Transcript	ENSMUST00000024849	protein_coding	rs49168605
17:71254664-71254664	downstream_gene_variant	MODIFIER	Lpin2	ENSMUSG00000024052	Transcript	ENSMUST00000129635	protein_coding	rs49168605
17:71254664-71254664	intron_variant,non_coding_trans	MODIFIER	Gm26561	ENSMUSG00000097625	Transcript	ENSMUST00000180743	lincRNA	rs49168605
17:71254664-71254664	intron_variant	MODIFIER	Emilin2	ENSMUSG00000024053	Transcript	ENSMUST00000232777	protein_coding	rs49168605
17:71254664-71254664	intron_variant	MODIFIER	Emilin2	ENSMUSG00000024053	Transcript	ENSMUST00000233057	protein_coding	rs49168605
17:71254664-71254664	intron_variant,non_coding_trans	MODIFIER	Emilin2	ENSMUSG00000024053	Transcript	ENSMUST00000233083	retained_intron	rs49168605
17:71254664-71254664	intron_variant	MODIFIER	Emilin2	ENSMUSG00000024053	Transcript	ENSMUST00000233148	protein_coding	rs49168605
17:71254664-71254664	intron_variant	MODIFIER	Emilin2	ENSMUSG00000024053	Transcript	ENSMUST00000233245	protein_coding	rs49168605
17:71254664-71254664	downstream_gene_variant	MODIFIER	Emilin2	ENSMUSG00000024053	Transcript	ENSMUST00000233677	retained_intron	rs49168605
17:71254664-71254664	downstream_gene_variant	MODIFIER	Emilin2	ENSMUSG00000024053	Transcript	ENSMUST00000233698	processed_transcript	rs49168605
17:71254664-71254664	regulatory_region_variant	MODIFIER	-	-	RegulatoryFeature	ENSMUSR00000126636	promoter_flanking_region	rs49168605
17:71254664-71254664	regulatory_region_variant	MODIFIER	-	-	RegulatoryFeature	ENSMUSR00000371568	CTCF_binding_site	rs49168605
17:71265477-71265477	intron_variant	MODIFIER	Emilin2	ENSMUSG00000024053	Transcript	ENSMUST00000024849	protein_coding	rs51803912
17:71265477-71265477	intron_variant,non_coding_trans	MODIFIER	Gm26561	ENSMUSG00000097625	Transcript	ENSMUST00000180743	lincRNA	rs51803912
17:71265477-71265477	intron_variant	MODIFIER	Emilin2	ENSMUSG00000024053	Transcript	ENSMUST00000232777	protein_coding	rs51803912
17:71265477-71265477	intron_variant	MODIFIER	Emilin2	ENSMUSG00000024053	Transcript	ENSMUST00000233057	protein_coding	rs51803912
17:71265477-71265477	intron_variant	MODIFIER	Emilin2	ENSMUSG00000024053	Transcript	ENSMUST00000233148	protein_coding	rs51803912
17:71265477-71265477	intron_variant	MODIFIER	Emilin2	ENSMUSG00000024053	Transcript	ENSMUST00000233245	protein_coding	rs51803912
17:71265477-71265477	upstream_gene_variant	MODIFIER	Emilin2	ENSMUSG00000024053	Transcript	ENSMUST00000233698	processed_transcript	rs51803912
17:71265477-71265477	regulatory_region_variant	MODIFIER	-	-	RegulatoryFeature	ENSMUSR00000126640	promoter_flanking_region	rs51803912
17:71365890-71365890	intron_variant	MODIFIER	Smchd1	ENSMUSG00000024054	Transcript	ENSMUST00000127430	protein_coding	rs33380475
17:71365890-71365890	upstream_gene_variant	MODIFIER	Smchd1	ENSMUSG00000024054	Transcript	ENSMUST00000147111	retained_intron	rs33380475
17:71365890-71365890	intron_variant,non_coding_trans	MODIFIER	Gm26561	ENSMUSG00000097625	Transcript	ENSMUST00000180743	lincRNA	rs33380475
17:71365890-71365890	upstream_gene_variant	MODIFIER	Smchd1	ENSMUSG00000024054	Transcript	ENSMUST00000182107	retained_intron	rs33380475
17:71365890-71365890	downstream_gene_variant	MODIFIER	Gm37639	ENSMUSG00000102863	Transcript	ENSMUST00000191623	TEC	rs33380475
18:10002708-10002708	intron_variant	MODIFIER	Usp14	ENSMUSG00000047879	Transcript	ENSMUST00000092096	protein_coding	rs30984331
18:10002708-10002708	intron_variant	MODIFIER	Usp14	ENSMUSG00000047879	Transcript	ENSMUST00000116669	protein_coding	rs30984331
18:10002708-10002708	upstream_gene_variant	MODIFIER	Usp14	ENSMUSG00000047879	Transcript	ENSMUST00000133594	retained_intron	rs30984331
18:10002708-10002708	intron_variant,non_coding_trans	MODIFIER	Usp14	ENSMUSG00000047879	Transcript	ENSMUST00000154088	retained_intron	rs30984331
18:10010102-10010102	intron_variant	MODIFIER	Usp14	ENSMUSG00000047879	Transcript	ENSMUST00000092096	protein_coding	rs30981425
18:10010102-10010102	intron_variant	MODIFIER	Usp14	ENSMUSG00000047879	Transcript	ENSMUST00000116669	protein_coding	rs30981425
18:10010102-10010102	intron_variant,non_coding_trans	MODIFIER	Usp14	ENSMUSG00000047879	Transcript	ENSMUST00000128334	retained_intron	rs30981425
18:10010102-10010102	downstream_gene_variant	MODIFIER	Usp14	ENSMUSG00000047879	Transcript	ENSMUST00000145929	retained_intron	rs30981425
18:10010102-10010102	upstream_gene_variant	MODIFIER	Usp14	ENSMUSG00000047879	Transcript	ENSMUST00000154088	retained_intron	rs30981425
18:10069709-10069709	intron_variant	MODIFIER	Rock1	ENSMUSG00000024290	Transcript	ENSMUST00000067947	protein_coding	rs30017970
18:10070111-10070111	intron_variant	MODIFIER	Rock1	ENSMUSG00000024290	Transcript	ENSMUST00000067947	protein_coding	rs584745048
18:10557120-10557120	intron_variant	MODIFIER	Greb1l	ENSMUSG00000042942	Transcript	ENSMUST00000048977	protein_coding	rs243485023
18:10557120-10557120	intron_variant,non_coding_trans	MODIFIER	Greb1l	ENSMUSG00000042942	Transcript	ENSMUST00000173261	processed_transcript	rs243485023
18:10558734-10558734	intron_variant	MODIFIER	Greb1l	ENSMUSG00000042942	Transcript	ENSMUST00000048977	protein_coding	rs247048245
18:10558734-10558734	intron_variant,non_coding_trans	MODIFIER	Greb1l	ENSMUSG00000042942	Transcript	ENSMUST00000173261	processed_transcript	rs247048245
18:10623820-10623820	intron_variant	MODIFIER	Snrpd1	ENSMUSG00000002477	Transcript	ENSMUST00000002551	protein_coding	rs213197332
18:10623872-10623872	intron_variant	MODIFIER	Snrpd1	ENSMUSG00000002477	Transcript	ENSMUST00000002551	protein_coding	rs235916212
18:10625942-10625942	intron_variant	MODIFIER	Snrpd1	ENSMUSG00000002477	Transcript	ENSMUST00000002551	protein_coding	rs51601502
18:10625945-10625945	intron_variant	MODIFIER	Snrpd1	ENSMUSG00000002477	Transcript	ENSMUST00000002551	protein_coding	rs46386758
18:10626144-10626144	intron_variant	MODIFIER	Snrpd1	ENSMUSG00000002477	Transcript	ENSMUST00000002551	protein_coding	rs256857636
18:10647772-10647772	synonymous_variant	LOW	Abhd3	ENSMUSG00000002475	Transcript	ENSMUST00000002549	protein_coding	rs30951978
18:10647772-10647772	synonymous_variant	LOW	Abhd3	ENSMUSG00000002475	Transcript	ENSMUST00000117726	protein_coding	rs30951978
18:10647772-10647772	synonymous_variant	LOW	Abhd3	ENSMUSG00000002475	Transcript	ENSMUST00000117828	protein_coding	rs30951978
18:10647772-10647772	synonymous_variant	LOW	Abhd3	ENSMUSG00000002475	Transcript	ENSMUST00000144150	protein_coding	rs30951978
18:11982459-11982459	3_prime_UTR_variant,NMD_trar	MODIFIER	Tmem241	ENSMUSG00000049411	Transcript	ENSMUST00000050228	nonsense_mediated_decay	rs31012381
18:11982459-11982459	3_prime_UTR_variant	MODIFIER	Tmem241	ENSMUSG00000049411	Transcript	ENSMUST00000055447	protein_coding	rs31012381
18:11982459-11982459	intron_variant	MODIFIER	Tmem241	ENSMUSG00000049411	Transcript	ENSMUST00000209628	protein_coding	rs31012381
18:11982459-11982459	downstream_gene_variant	MODIFIER	Tmem241	ENSMUSG00000049411	Transcript	ENSMUST00000209859	protein_coding	rs31012381
18:11982459-11982459	3_prime_UTR_variant,NMD_trar	MODIFIER	Tmem241	ENSMUSG00000049411	Transcript	ENSMUST00000211298	nonsense_mediated_decay	rs31012381
18:12137616-12137616	intron_variant	MODIFIER	Riok3	ENSMUSG00000024404	Transcript	ENSMUST00000025270	protein_coding	rs6289920
18:12138764-12138764	intron_variant	MODIFIER	Riok3	ENSMUSG00000024404	Transcript	ENSMUST00000025270	protein_coding	rs213920180
18:12140183-12140183	intron_variant	MODIFIER	Riok3	ENSMUSG00000024404	Transcript	ENSMUST00000025270	protein_coding	rs583612945

18:12140183-12140183	regulatory_region_variant	MODIFIER	-	-	RegulatoryFeature	ENSMUSR00000130743	open_chromatin_region	rs583612945
18:12140543-12140543	intron_variant	MODIFIER	Riok3	ENSMUSG00000024404	Transcript	ENSMUST00000025270	protein_coding	rs222777175
18:12140550-12140550	intron_variant	MODIFIER	Riok3	ENSMUSG00000024404	Transcript	ENSMUST00000025270	protein_coding	rs240953681
18:12140862-12140862	intron_variant	MODIFIER	Riok3	ENSMUSG00000024404	Transcript	ENSMUST00000025270	protein_coding	rs30995854
18:12141838-12141838	intron_variant	MODIFIER	Riok3	ENSMUSG00000024404	Transcript	ENSMUST00000025270	protein_coding	rs239564160
18:12146058-12146058	intron_variant	MODIFIER	Riok3	ENSMUSG00000024404	Transcript	ENSMUST00000025270	protein_coding	rs255933219
18:12146084-12146087	intron_variant	MODIFIER	Riok3	ENSMUSG00000024404	Transcript	ENSMUST00000025270	protein_coding	rs212207833
18:12146375-12146375	intron_variant	MODIFIER	Riok3	ENSMUSG00000024404	Transcript	ENSMUST00000025270	protein_coding	rs250017563
18:12153465-12153465	intron_variant	MODIFIER	Riok3	ENSMUSG00000024404	Transcript	ENSMUST00000025270	protein_coding	rs256786052
18:12154284-12154284	intron_variant	MODIFIER	Riok3	ENSMUSG00000024404	Transcript	ENSMUST00000025270	protein_coding	rs30996576
18:12154284-12154284	regulatory_region_variant	MODIFIER	-	-	RegulatoryFeature	ENSMUSR00000374022	TF_binding_site	rs30996576
18:12154744-12154745	intron_variant	MODIFIER	Riok3	ENSMUSG00000024404	Transcript	ENSMUST00000025270	protein_coding	rs227118896
18:12154773-12154773	intron_variant	MODIFIER	Riok3	ENSMUSG00000024404	Transcript	ENSMUST00000025270	protein_coding	rs30995680
18:12180588-12180588	intron_variant	MODIFIER	Rmc1	ENSMUSG00000024410	Transcript	ENSMUST00000025276	protein_coding	rs30994919
18:12180588-12180588	intron_variant,non_coding_trans	MODIFIER	Rmc1	ENSMUSG00000024410	Transcript	ENSMUST00000126523	retained_intron	rs30994919
18:12180588-12180588	intron_variant,non_coding_trans	MODIFIER	Rmc1	ENSMUSG00000024410	Transcript	ENSMUST00000127123	retained_intron	rs30994919
18:12180588-12180588	intron_variant	MODIFIER	Rmc1	ENSMUSG00000024410	Transcript	ENSMUST00000134046	protein_coding	rs30994919
18:12180588-12180588	intron_variant,NMD_transcript_v	MODIFIER	Rmc1	ENSMUSG00000024410	Transcript	ENSMUST00000138866	nonsense_mediated_decay	rs30994919
18:12180588-12180588	upstream_gene_variant	MODIFIER	Rmc1	ENSMUSG00000024410	Transcript	ENSMUST00000139151	retained_intron	rs30994919
18:12180588-12180588	downstream_gene_variant	MODIFIER	Rmc1	ENSMUSG00000024410	Transcript	ENSMUST00000155431	retained_intron	rs30994919
18:12189967-12189968	3_prime_UTR_variant	MODIFIER	Rmc1	ENSMUSG00000024410	Transcript	ENSMUST00000025276	protein_coding	rs234365168
18:12189967-12189968	3_prime_UTR_variant	MODIFIER	Npc1	ENSMUSG00000024413	Transcript	ENSMUST00000025279	protein_coding	rs234365168
18:12189967-12189968	non_coding_transcript_exon_vai	MODIFIER	Rmc1	ENSMUSG00000024410	Transcript	ENSMUST00000127123	retained_intron	rs234365168
18:12189967-12189968	non_coding_transcript_exon_vai	MODIFIER	Rmc1	ENSMUSG00000024410	Transcript	ENSMUST00000134756	retained_intron	rs234365168
18:12189967-12189968	downstream_gene_variant	MODIFIER	Rmc1	ENSMUSG00000024410	Transcript	ENSMUST00000138866	nonsense_mediated_decay	rs234365168
18:12189967-12189968	non_coding_transcript_exon_vai	MODIFIER	Rmc1	ENSMUSG00000024410	Transcript	ENSMUST00000139151	retained_intron	rs234365168
18:12189967-12189968	downstream_gene_variant	MODIFIER	Rmc1	ENSMUSG00000024410	Transcript	ENSMUST00000153233	retained_intron	rs234365168
18:12189967-12189968	upstream_gene_variant	MODIFIER	Gm15956	ENSMUSG00000086935	Transcript	ENSMUST00000153352	antisense	rs234365168
18:12190114-12190114	downstream_gene_variant	MODIFIER	Rmc1	ENSMUSG00000024410	Transcript	ENSMUST00000025276	protein_coding	rs13473454
18:12190114-12190114	3_prime_UTR_variant	MODIFIER	Npc1	ENSMUSG00000024413	Transcript	ENSMUST00000025279	protein_coding	rs13473454
18:12190114-12190114	downstream_gene_variant	MODIFIER	Rmc1	ENSMUSG00000024410	Transcript	ENSMUST00000127123	retained_intron	rs13473454
18:12190114-12190114	downstream_gene_variant	MODIFIER	Rmc1	ENSMUSG00000024410	Transcript	ENSMUST00000134756	retained_intron	rs13473454
18:12190114-12190114	downstream_gene_variant	MODIFIER	Rmc1	ENSMUSG00000024410	Transcript	ENSMUST00000138866	nonsense_mediated_decay	rs13473454
18:12190114-12190114	downstream_gene_variant	MODIFIER	Rmc1	ENSMUSG00000024410	Transcript	ENSMUST00000139151	retained_intron	rs13473454
18:12190114-12190114	downstream_gene_variant	MODIFIER	Rmc1	ENSMUSG00000024410	Transcript	ENSMUST00000153233	retained_intron	rs13473454
18:12190114-12190114	upstream_gene_variant	MODIFIER	Gm15956	ENSMUSG00000086935	Transcript	ENSMUST00000153352	antisense	rs13473454
18:12210061-12210061	intron_variant	MODIFIER	Npc1	ENSMUSG00000024413	Transcript	ENSMUST00000025279	protein_coding	rs30984496
18:12210061-12210061	downstream_gene_variant	MODIFIER	Npc1	ENSMUSG00000024413	Transcript	ENSMUST00000145771	processed_transcript	rs30984496
18:12643527-12643527	5_prime_UTR_variant	MODIFIER	Ttc39c	ENSMUSG00000024424	Transcript	ENSMUST00000025294	protein_coding	rs248193761
18:12643527-12643527	non_coding_transcript_exon_vai	MODIFIER	Ttc39c	ENSMUSG00000024424	Transcript	ENSMUST00000141060	retained_intron	rs248193761
18:12643527-12643527	intron_variant	MODIFIER	Ttc39c	ENSMUSG00000024424	Transcript	ENSMUST00000169401	protein_coding	rs248193761
18:12643527-12643527	regulatory_region_variant	MODIFIER	-	-	RegulatoryFeature	ENSMUSR00000130823	promoter	rs248193761
18:12686954-12686954	splice_region_variant,synonymo	LOW	Ttc39c	ENSMUSG00000024424	Transcript	ENSMUST00000025294	protein_coding	rs29679431
18:12686954-12686954	splice_region_variant,non_codin	LOW	Ttc39c	ENSMUSG00000024424	Transcript	ENSMUST00000141060	retained_intron	rs29679431
18:12686954-12686954	splice_region_variant,synonymo	LOW	Ttc39c	ENSMUSG00000024424	Transcript	ENSMUST00000169401	protein_coding	rs29679431
18:12728664-12728664	missense_variant	MODERATE	Ttc39c	ENSMUSG00000024424	Transcript	ENSMUST00000025294	protein_coding	rs46768393
18:12728664-12728664	missense_variant	MODERATE	Ttc39c	ENSMUSG00000024424	Transcript	ENSMUST00000169401	protein_coding	rs46768393
18:12926462-12926462	intron_variant	MODIFIER	Osbpl1a	ENSMUSG00000044252	Transcript	ENSMUST00000074352	protein_coding	rs31061008
18:12926462-12926462	intron_variant	MODIFIER	Osbpl1a	ENSMUSG00000044252	Transcript	ENSMUST00000122175	protein_coding	rs31061008
18:12926462-12926462	intron_variant,non_coding_trans	MODIFIER	Osbpl1a	ENSMUSG00000044252	Transcript	ENSMUST00000132594	retained_intron	rs31061008
18:12926462-12926462	downstream_gene_variant	MODIFIER	Osbpl1a	ENSMUSG00000044252	Transcript	ENSMUST00000141651	retained_intron	rs31061008
18:12926462-12926462	intron_variant,NMD_transcript_v	MODIFIER	Osbpl1a	ENSMUSG00000044252	Transcript	ENSMUST00000147197	nonsense_mediated_decay	rs31061008
18:12926462-12926462	regulatory_region_variant	MODIFIER	-	-	RegulatoryFeature	ENSMUSR00000602883	enhancer	rs31061008
18:12927590-12927590	intron_variant	MODIFIER	Osbpl1a	ENSMUSG00000044252	Transcript	ENSMUST00000074352	protein_coding	rs250466392
18:12927590-12927590	intron_variant	MODIFIER	Osbpl1a	ENSMUSG00000044252	Transcript	ENSMUST00000122175	protein_coding	rs250466392
18:12927590-12927590	intron_variant,non_coding_trans	MODIFIER	Osbpl1a	ENSMUSG00000044252	Transcript	ENSMUST00000132594	retained_intron	rs250466392
18:12927590-12927590	downstream_gene_variant	MODIFIER	Osbpl1a	ENSMUSG00000044252	Transcript	ENSMUST00000141651	retained_intron	rs250466392
18:12927590-12927590	intron_variant,NMD_transcript_v	MODIFIER	Osbpl1a	ENSMUSG00000044252	Transcript	ENSMUST00000147197	nonsense_mediated_decay	rs250466392
18:12927971-12927971	intron_variant	MODIFIER	Osbpl1a	ENSMUSG00000044252	Transcript	ENSMUST00000074352	protein_coding	rs243224283
18:12927971-12927971	intron_variant	MODIFIER	Osbpl1a	ENSMUSG00000044252	Transcript	ENSMUST00000122175	protein_coding	rs243224283
18:12927971-12927971	intron_variant,non_coding_trans	MODIFIER	Osbpl1a	ENSMUSG00000044252	Transcript	ENSMUST00000132594	retained_intron	rs243224283
18:12927971-12927971	downstream_gene_variant	MODIFIER	Osbpl1a	ENSMUSG00000044252	Transcript	ENSMUST00000141651	retained_intron	rs243224283
18:12927971-12927971	intron_variant,NMD_transcript_v	MODIFIER	Osbpl1a	ENSMUSG00000044252	Transcript	ENSMUST00000147197	nonsense_mediated_decay	rs243224283
18:12941650-12941650	5_prime_UTR_variant	MODIFIER	Osbpl1a	ENSMUSG00000044252	Transcript	ENSMUST00000074352	protein_coding	rs228090504
18:12941650-12941650	5_prime_UTR_variant	MODIFIER	Osbpl1a	ENSMUSG00000044252	Transcript	ENSMUST00000122175	protein_coding	rs228090504
18:12941650-12941650	non_coding_transcript_exon_vai	MODIFIER	Osbpl1a	ENSMUSG00000044252	Transcript	ENSMUST00000132594	retained_intron	rs228090504
18:12941650-12941650	non_coding_transcript_exon_vai	MODIFIER	Osbpl1a	ENSMUSG00000044252	Transcript	ENSMUST00000141651	retained_intron	rs228090504
18:12941650-12941650	non_coding_transcript_exon_vai	MODIFIER	Osbpl1a	ENSMUSG00000044252	Transcript	ENSMUST00000142467	processed_transcript	rs228090504
18:12941650-12941650	5_prime_UTR_variant,NMD_trar	MODIFIER	Osbpl1a	ENSMUSG00000044252	Transcript	ENSMUST00000147197	nonsense_mediated_decay	rs228090504

18:12941650-12941650	regulatory_region_variant	MODIFIER	-	-	RegulatoryFeature	ENSMUSR00000130917	promoter	rs228090504
18:12957810-12957810	upstream_gene_variant	MODIFIER	Gm26407	ENSMUSG00000089129	Transcript	ENSMUST00000158504	misc_RNA	rs29720003
18:12957810-12957810	regulatory_region_variant	MODIFIER	-	-	RegulatoryFeature	ENSMUSR00000130921	promoter_flanking_region	rs29720003
18:12959499-12959499	upstream_gene_variant	MODIFIER	Gm26407	ENSMUSG00000089129	Transcript	ENSMUST00000158504	misc_RNA	rs251693533
18:12965624-12965624	downstream_gene_variant	MODIFIER	Gm26407	ENSMUSG00000089129	Transcript	ENSMUST00000158504	misc_RNA	rs51216894
18:12967918-12967918	upstream_gene_variant	MODIFIER	Impact	ENSMUSG00000024423	Transcript	ENSMUST00000025290	protein_coding	rs31058810
18:12971989-12971989	upstream_gene_variant	MODIFIER	Impact	ENSMUSG00000024423	Transcript	ENSMUST00000025290	protein_coding	rs6384990
18:12979939-12979939	intron_variant	MODIFIER	Impact	ENSMUSG00000024423	Transcript	ENSMUST00000025290	protein_coding	rs108196979
18:14571023-14571023	downstream_gene_variant	MODIFIER	Gm6457	ENSMUSG00000053740	Transcript	ENSMUST00000066378	processed_pseudogene	-
18:14762131-14762131	3_prime_UTR_variant	MODIFIER	Psma8	ENSMUSG00000036743	Transcript	ENSMUST00000040860	protein_coding	rs30016825
18:20699319-20699319	intron_variant	MODIFIER	B4galt6	ENSMUSG00000056124	Transcript	ENSMUST00000070080	protein_coding	rs31316994
18:20829356-20829356	intron_variant	MODIFIER	Trappc8	ENSMUSG00000033382	Transcript	ENSMUST00000025177	protein_coding	rs31308543
18:20829356-20829356	non_coding_transcript_exon_va	MODIFIER	Trappc8	ENSMUSG00000033382	Transcript	ENSMUST00000225502	retained_intron	rs31308543
18:20829356-20829356	intron_variant	MODIFIER	Trappc8	ENSMUSG00000033382	Transcript	ENSMUST00000225661	protein_coding	rs31308543
18:30342741-30342741	missense_variant	MODERATE	Pik3c3	ENSMUSG00000033628	Transcript	ENSMUST00000091978	protein_coding	rs49405313
18:30342741-30342741	intron_variant	MODIFIER	Pik3c3	ENSMUSG00000033628	Transcript	ENSMUST00000115812	protein_coding	rs49405313
18:30342741-30342741	intron_variant,NMD_transcript_v	MODIFIER	Pik3c3	ENSMUSG00000033628	Transcript	ENSMUST00000131405	nonsense_mediated_decay	rs49405313
18:31597312-31597312	intron_variant	MODIFIER	Slc25a46	ENSMUSG00000024259	Transcript	ENSMUST00000060396	protein_coding	rs29871182
18:31618829-31618829	intergenic_variant	MODIFIER	-	-	-	-	-	rs30051486
18:31865295-31865295	intron_variant	MODIFIER	Wdr33	ENSMUSG00000024400	Transcript	ENSMUST00000025264	protein_coding	rs30262899
18:31865295-31865295	upstream_gene_variant	MODIFIER	Gm26823	ENSMUSG00000096926	Transcript	ENSMUST00000181425	lincRNA	rs30262899
18:31907862-31907862	3_prime_UTR_variant	MODIFIER	Wdr33	ENSMUSG00000024400	Transcript	ENSMUST00000025264	protein_coding	rs48229800
18:31907862-31907862	downstream_gene_variant	MODIFIER	Sft2d3	ENSMUSG00000044982	Transcript	ENSMUST00000054984	protein_coding	rs48229800
18:33795620-33795620	downstream_gene_variant	MODIFIER	Epb41l4a	ENSMUSG00000024376	Transcript	ENSMUST00000025234	protein_coding	rs51979167
18:33795620-33795620	intron_variant,non_coding_trans	MODIFIER	Epb41l4aos	ENSMUSG00000087590	Transcript	ENSMUST00000146010	lincRNA	rs51979167
18:33795620-33795620	downstream_gene_variant	MODIFIER	Gm23639	ENSMUSG00000092819	Transcript	ENSMUST00000175078	snoRNA	rs51979167
18:33795620-33795620	intron_variant,non_coding_trans	MODIFIER	Epb41l4aos	ENSMUSG00000087590	Transcript	ENSMUST00000179138	lincRNA	rs51979167
18:33795620-33795620	regulatory_region_variant	MODIFIER	-	-	RegulatoryFeature	ENSMUSR00000132500	promoter	rs51979167
18:33795621-33795621	downstream_gene_variant	MODIFIER	Epb41l4a	ENSMUSG00000024376	Transcript	ENSMUST00000025234	protein_coding	rs107733986
18:33795621-33795621	intron_variant,non_coding_trans	MODIFIER	Epb41l4aos	ENSMUSG00000087590	Transcript	ENSMUST00000146010	lincRNA	rs107733986
18:33795621-33795621	downstream_gene_variant	MODIFIER	Gm23639	ENSMUSG00000092819	Transcript	ENSMUST00000175078	snoRNA	rs107733986
18:33795621-33795621	intron_variant,non_coding_trans	MODIFIER	Epb41l4aos	ENSMUSG00000087590	Transcript	ENSMUST00000179138	lincRNA	rs107733986
18:33795621-33795621	regulatory_region_variant	MODIFIER	-	-	RegulatoryFeature	ENSMUSR00000132500	promoter	rs107733986
18:34333007-34333007	intron_variant	MODIFIER	Srp19	ENSMUSG00000014504	Transcript	ENSMUST00000072576	protein_coding	rs31610221
18:34333007-34333007	intron_variant	MODIFIER	Srp19	ENSMUSG00000014504	Transcript	ENSMUST00000119329	protein_coding	rs31610221
18:34333007-34333007	intron_variant,non_coding_trans	MODIFIER	Srp19	ENSMUSG00000014504	Transcript	ENSMUST00000126518	processed_transcript	rs31610221
18:34333007-34333007	intron_variant,non_coding_trans	MODIFIER	Srp19	ENSMUSG00000014504	Transcript	ENSMUST00000131713	processed_transcript	rs31610221
18:34333007-34333007	non_coding_transcript_exon_va	MODIFIER	Srp19	ENSMUSG00000014504	Transcript	ENSMUST00000140279	retained_intron	rs31610221
18:34333007-34333007	3_prime_UTR_variant,NMD_tran	MODIFIER	Srp19	ENSMUSG00000014504	Transcript	ENSMUST00000142010	nonsense_mediated_decay	rs31610221
18:34635017-34635017	intron_variant	MODIFIER	Cdc23	ENSMUSG00000024370	Transcript	ENSMUST00000025228	protein_coding	rs242255940
18:34635017-34635017	downstream_gene_variant	MODIFIER	Cdc23	ENSMUSG00000024370	Transcript	ENSMUST00000133162	retained_intron	rs242255940
18:34635017-34635017	intron_variant	MODIFIER	Cdc23	ENSMUSG00000024370	Transcript	ENSMUST00000133181	protein_coding	rs242255940
18:34635017-34635017	downstream_gene_variant	MODIFIER	Cdc23	ENSMUSG00000024370	Transcript	ENSMUST00000136949	retained_intron	rs242255940
18:34635017-34635017	upstream_gene_variant	MODIFIER	Cdc23	ENSMUSG00000024370	Transcript	ENSMUST00000138400	retained_intron	rs242255940
18:34635017-34635017	downstream_gene_variant	MODIFIER	Cdc23	ENSMUSG00000024370	Transcript	ENSMUST00000155307	retained_intron	rs242255940
18:34635017-34635017	downstream_gene_variant	MODIFIER	Kif20a	ENSMUSG00000003779	Transcript	ENSMUST00000166044	protein_coding	rs242255940
18:34635017-34635017	downstream_gene_variant	MODIFIER	Kif20a	ENSMUSG00000003779	Transcript	ENSMUST00000167161	protein_coding	rs242255940
18:34764161-34764161	intron_variant	MODIFIER	Fam53c	ENSMUSG00000034300	Transcript	ENSMUST00000049281	protein_coding	rs47136197
18:34764161-34764161	intron_variant	MODIFIER	Fam53c	ENSMUSG00000034300	Transcript	ENSMUST00000097622	protein_coding	rs47136197
18:34813368-34813368	intron_variant	MODIFIER	Kdm3b	ENSMUSG00000038773	Transcript	ENSMUST00000043775	protein_coding	rs29885409
18:34813368-34813368	upstream_gene_variant	MODIFIER	Kdm3b	ENSMUSG00000038773	Transcript	ENSMUST00000224065	processed_transcript	rs29885409
18:34813368-34813368	intron_variant,NMD_transcript_v	MODIFIER	Kdm3b	ENSMUSG00000038773	Transcript	ENSMUST00000225195	nonsense_mediated_decay	rs29885409
18:34917804-34917804	intron_variant	MODIFIER	Etf1	ENSMUSG00000024360	Transcript	ENSMUST00000025218	protein_coding	rs220699929
18:34917804-34917804	regulatory_region_variant	MODIFIER	-	-	RegulatoryFeature	ENSMUSR00000132713	promoter_flanking_region	rs220699929
18:34932802-34932803	downstream_gene_variant	MODIFIER	Hspa9	ENSMUSG00000024359	Transcript	ENSMUST00000025217	protein_coding	rs239770975
18:34932802-34932803	upstream_gene_variant	MODIFIER	Etf1	ENSMUSG00000024360	Transcript	ENSMUST00000025218	protein_coding	rs239770975
18:34932802-34932803	upstream_gene_variant	MODIFIER	Etf1	ENSMUSG00000024360	Transcript	ENSMUST00000180351	protein_coding	rs239770975
18:34932802-34932803	regulatory_region_variant	MODIFIER	-	-	RegulatoryFeature	ENSMUSR00000132718	promoter	rs239770975
18:34933786-34933786	downstream_gene_variant	MODIFIER	Hspa9	ENSMUSG00000024359	Transcript	ENSMUST00000025217	protein_coding	rs223140497
18:34933786-34933786	upstream_gene_variant	MODIFIER	Etf1	ENSMUSG00000024360	Transcript	ENSMUST00000025218	protein_coding	rs223140497
18:34933786-34933786	downstream_gene_variant	MODIFIER	Hspa9	ENSMUSG00000024359	Transcript	ENSMUST00000172829	retained_intron	rs223140497
18:34933786-34933786	downstream_gene_variant	MODIFIER	Hspa9	ENSMUSG00000024359	Transcript	ENSMUST00000173701	retained_intron	rs223140497
18:34933786-34933786	upstream_gene_variant	MODIFIER	Etf1	ENSMUSG00000024360	Transcript	ENSMUST00000180351	protein_coding	rs223140497
18:34933786-34933786	regulatory_region_variant	MODIFIER	-	-	RegulatoryFeature	ENSMUSR00000132718	promoter	rs223140497
18:34933978-34933978	downstream_gene_variant	MODIFIER	Hspa9	ENSMUSG00000024359	Transcript	ENSMUST00000025217	protein_coding	rs31816692
18:34933978-34933978	upstream_gene_variant	MODIFIER	Etf1	ENSMUSG00000024360	Transcript	ENSMUST00000025218	protein_coding	rs31816692
18:34933978-34933978	downstream_gene_variant	MODIFIER	Hspa9	ENSMUSG00000024359	Transcript	ENSMUST00000172829	retained_intron	rs31816692
18:34933978-34933978	downstream_gene_variant	MODIFIER	Hspa9	ENSMUSG00000024359	Transcript	ENSMUST00000173701	retained_intron	rs31816692
18:34933978-34933978	upstream_gene_variant	MODIFIER	Etf1	ENSMUSG00000024360	Transcript	ENSMUST00000180351	protein_coding	rs31816692

18:34933978-34933978	regulatory_region_variant	MODIFIER	-	-	RegulatoryFeature	ENSMUSR00000604667	CTCF_binding_site	rs31816692
18:35650677-35650677	upstream_gene_variant	MODIFIER	Mzb1	ENSMUSG00000024353	Transcript	ENSMUST00000025211	protein_coding	rs243462458
18:35650677-35650677	downstream_gene_variant	MODIFIER	Prob1	ENSMUSG00000073600	Transcript	ENSMUST00000097619	protein_coding	rs243462458
18:35650677-35650677	non_coding_transcript_exon_var	MODIFIER	Prob1	ENSMUSG00000073600	Transcript	ENSMUST00000186951	processed_transcript	rs243462458
18:35650677-35650677	3_prime_UTR_variant	MODIFIER	Prob1	ENSMUSG00000073600	Transcript	ENSMUST00000190196	protein_coding	rs243462458
18:35671710-35671710	3_prime_UTR_variant	MODIFIER	Dnajc18	ENSMUSG00000024350	Transcript	ENSMUST00000025208	protein_coding	rs256819209
18:35671710-35671710	regulatory_region_variant	MODIFIER	-	-	RegulatoryFeature	ENSMUSR00000375377	promoter_flanking_region	rs256819209
18:35671925-35671925	3_prime_UTR_variant	MODIFIER	Dnajc18	ENSMUSG00000024350	Transcript	ENSMUST00000025208	protein_coding	rs264632657
18:35671925-35671925	regulatory_region_variant	MODIFIER	-	-	RegulatoryFeature	ENSMUSR00000375377	promoter_flanking_region	rs264632657
18:35673317-35673318	3_prime_UTR_variant	MODIFIER	Dnajc18	ENSMUSG00000024350	Transcript	ENSMUST00000025208	protein_coding	rs248460133
18:35673317-35673318	regulatory_region_variant	MODIFIER	-	-	RegulatoryFeature	ENSMUSR00000375377	promoter_flanking_region	rs248460133
18:35673421-35673421	3_prime_UTR_variant	MODIFIER	Dnajc18	ENSMUSG00000024350	Transcript	ENSMUST00000025208	protein_coding	rs237527297
18:35673421-35673421	regulatory_region_variant	MODIFIER	-	-	RegulatoryFeature	ENSMUSR00000375377	promoter_flanking_region	rs237527297
18:35735360-35735360	downstream_gene_variant	MODIFIER	1700066B19Rik	ENSMUSG00000073598	Transcript	ENSMUST00000097617	protein_coding	rs45914274
18:35735360-35735360	intron_variant	MODIFIER	Tmem173	ENSMUSG00000024349	Transcript	ENSMUST00000115728	protein_coding	rs45914274
18:35736698-35736698	intron_variant	MODIFIER	Tmem173	ENSMUSG00000024349	Transcript	ENSMUST00000115728	protein_coding	rs31809053
18:35737513-35737513	intron_variant	MODIFIER	Tmem173	ENSMUSG00000024349	Transcript	ENSMUST00000115728	protein_coding	rs250876225
18:35737668-35737668	intron_variant	MODIFIER	Tmem173	ENSMUSG00000024349	Transcript	ENSMUST00000115728	protein_coding	rs236736179
18:35738026-35738026	intron_variant	MODIFIER	Tmem173	ENSMUSG00000024349	Transcript	ENSMUST00000115728	protein_coding	rs31809052
18:35738465-35738465	intron_variant	MODIFIER	Tmem173	ENSMUSG00000024349	Transcript	ENSMUST00000115728	protein_coding	rs226434147
18:36015804-36015804	downstream_gene_variant	MODIFIER	Nrg2	ENSMUSG00000060275	Transcript	ENSMUST00000115713	protein_coding	rs29766190
18:36015804-36015804	downstream_gene_variant	MODIFIER	Psd2	ENSMUSG00000024347	Transcript	ENSMUST00000115716	protein_coding	rs29766190
18:36015804-36015804	downstream_gene_variant	MODIFIER	Psd2	ENSMUSG00000024347	Transcript	ENSMUST00000175734	protein_coding	rs29766190
18:36015804-36015804	downstream_gene_variant	MODIFIER	Psd2	ENSMUSG00000024347	Transcript	ENSMUST00000176472	nonsense_mediated_decay	rs29766190
18:36015804-36015804	downstream_gene_variant	MODIFIER	Psd2	ENSMUSG00000024347	Transcript	ENSMUST00000176873	protein_coding	rs29766190
18:36015804-36015804	downstream_gene_variant	MODIFIER	Psd2	ENSMUSG00000024347	Transcript	ENSMUST00000177432	protein_coding	rs29766190
18:36015821-36015821	downstream_gene_variant	MODIFIER	Nrg2	ENSMUSG00000060275	Transcript	ENSMUST00000115713	protein_coding	rs29679550
18:36015821-36015821	downstream_gene_variant	MODIFIER	Psd2	ENSMUSG00000024347	Transcript	ENSMUST00000115716	protein_coding	rs29679550
18:36015821-36015821	downstream_gene_variant	MODIFIER	Psd2	ENSMUSG00000024347	Transcript	ENSMUST00000175734	protein_coding	rs29679550
18:36015821-36015821	downstream_gene_variant	MODIFIER	Psd2	ENSMUSG00000024347	Transcript	ENSMUST00000176472	nonsense_mediated_decay	rs29679550
18:36015821-36015821	downstream_gene_variant	MODIFIER	Psd2	ENSMUSG00000024347	Transcript	ENSMUST00000176873	protein_coding	rs29679550
18:36015821-36015821	downstream_gene_variant	MODIFIER	Psd2	ENSMUSG00000024347	Transcript	ENSMUST00000177432	protein_coding	rs29679550
18:36015860-36015860	downstream_gene_variant	MODIFIER	Nrg2	ENSMUSG00000060275	Transcript	ENSMUST00000115713	protein_coding	rs30261808
18:36015860-36015860	downstream_gene_variant	MODIFIER	Psd2	ENSMUSG00000024347	Transcript	ENSMUST00000115716	protein_coding	rs30261808
18:36015860-36015860	downstream_gene_variant	MODIFIER	Psd2	ENSMUSG00000024347	Transcript	ENSMUST00000175734	protein_coding	rs30261808
18:36015860-36015860	downstream_gene_variant	MODIFIER	Psd2	ENSMUSG00000024347	Transcript	ENSMUST00000176472	nonsense_mediated_decay	rs30261808
18:36015860-36015860	downstream_gene_variant	MODIFIER	Psd2	ENSMUSG00000024347	Transcript	ENSMUST00000176873	protein_coding	rs30261808
18:36015860-36015860	downstream_gene_variant	MODIFIER	Psd2	ENSMUSG00000024347	Transcript	ENSMUST00000177432	protein_coding	rs30261808
18:36016006-36016006	downstream_gene_variant	MODIFIER	Nrg2	ENSMUSG00000060275	Transcript	ENSMUST00000115713	protein_coding	rs30306412
18:36016006-36016006	downstream_gene_variant	MODIFIER	Psd2	ENSMUSG00000024347	Transcript	ENSMUST00000115716	protein_coding	rs30306412
18:36016006-36016006	downstream_gene_variant	MODIFIER	Psd2	ENSMUSG00000024347	Transcript	ENSMUST00000175734	protein_coding	rs30306412
18:36016006-36016006	downstream_gene_variant	MODIFIER	Psd2	ENSMUSG00000024347	Transcript	ENSMUST00000176472	nonsense_mediated_decay	rs30306412
18:36016006-36016006	downstream_gene_variant	MODIFIER	Psd2	ENSMUSG00000024347	Transcript	ENSMUST00000176873	protein_coding	rs30306412
18:36016006-36016006	downstream_gene_variant	MODIFIER	Psd2	ENSMUSG00000024347	Transcript	ENSMUST00000177432	protein_coding	rs30306412
18:36033743-36033743	intron_variant,non_coding_trans	MODIFIER	Nrg2	ENSMUSG00000060275	Transcript	ENSMUST00000115705	processed_transcript	rs30308720
18:36033743-36033743	intron_variant	MODIFIER	Nrg2	ENSMUSG00000060275	Transcript	ENSMUST00000115712	protein_coding	rs30308720
18:36033743-36033743	intron_variant	MODIFIER	Nrg2	ENSMUSG00000060275	Transcript	ENSMUST00000115713	protein_coding	rs30308720
18:36033743-36033743	intron_variant,non_coding_trans	MODIFIER	Nrg2	ENSMUSG00000060275	Transcript	ENSMUST00000225173	processed_transcript	rs30308720
18:36660759-36660759	downstream_gene_variant	MODIFIER	Ankhd1	ENSMUSG00000024483	Transcript	ENSMUST00000006205	nonsense_mediated_decay	rs31793384
18:36660759-36660759	upstream_gene_variant	MODIFIER	Eif4ebp3	ENSMUSG00000090264	Transcript	ENSMUST00000036765	protein_coding	rs31793384
18:36660759-36660759	downstream_gene_variant	MODIFIER	Ankhd1	ENSMUSG00000024483	Transcript	ENSMUST00000037072	protein_coding	rs31793384
18:36660759-36660759	downstream_gene_variant	MODIFIER	Ankhd1	ENSMUSG00000024483	Transcript	ENSMUST00000116653	protein_coding	rs31793384
18:36660759-36660759	intron_variant	MODIFIER	Ankhd1	ENSMUSG00000024483	Transcript	ENSMUST00000140061	protein_coding	rs31793384
18:36660759-36660759	intron_variant,NMD_transcript_v	MODIFIER	Ankhd1	ENSMUSG00000024483	Transcript	ENSMUST00000142977	nonsense_mediated_decay	rs31793384
18:36660759-36660759	downstream_gene_variant	MODIFIER	Ankhd1	ENSMUSG00000024483	Transcript	ENSMUST00000155329	protein_coding	rs31793384
18:36679731-36679731	upstream_gene_variant	MODIFIER	Sra1	ENSMUSG00000006050	Transcript	ENSMUST00000001415	protein_coding	rs30101280
18:36679731-36679731	intron_variant	MODIFIER	Slc35a4	ENSMUSG00000033272	Transcript	ENSMUST00000036158	protein_coding	rs30101280
18:36679731-36679731	intron_variant	MODIFIER	Slc35a4	ENSMUSG00000033272	Transcript	ENSMUST00000050476	protein_coding	rs30101280
18:36679731-36679731	intron_variant,non_coding_trans	MODIFIER	Slc35a4	ENSMUSG00000033272	Transcript	ENSMUST00000168343	processed_transcript	rs30101280
18:36679731-36679731	intron_variant,non_coding_trans	MODIFIER	Slc35a4	ENSMUSG00000033272	Transcript	ENSMUST00000170288	processed_transcript	rs30101280
18:36679731-36679731	intron_variant	MODIFIER	Slc35a4	ENSMUSG00000033272	Transcript	ENSMUST00000185899	protein_coding	rs30101280
18:36679731-36679731	intron_variant	MODIFIER	Slc35a4	ENSMUSG00000033272	Transcript	ENSMUST00000186538	protein_coding	rs30101280
18:36679731-36679731	regulatory_region_variant	MODIFIER	-	-	RegulatoryFeature	ENSMUSR00000133085	promoter	rs30101280
18:36747459-36747459	intron_variant	MODIFIER	Ik	ENSMUSG00000024474	Transcript	ENSMUST00000007042	protein_coding	rs31789898
18:36747459-36747459	upstream_gene_variant	MODIFIER	Ndufa2	ENSMUSG00000014294	Transcript	ENSMUST00000014438	protein_coding	rs31789898
18:36747459-36747459	intron_variant,non_coding_trans	MODIFIER	Ik	ENSMUSG00000024474	Transcript	ENSMUST00000224284	processed_transcript	rs31789898
18:36767035-36767035	intron_variant	MODIFIER	Hars	ENSMUSG00000001380	Transcript	ENSMUST00000001416	protein_coding	rs31790950
18:36767035-36767035	downstream_gene_variant	MODIFIER	Wdr55	ENSMUSG00000042660	Transcript	ENSMUST00000049323	protein_coding	rs31790950
18:36767035-36767035	upstream_gene_variant	MODIFIER	Dnd1	ENSMUSG00000044595	Transcript	ENSMUST00000061522	protein_coding	rs31790950

18:36767035-36767035	upstream_gene_variant	MODIFIER	Dnd1	ENSMUSG00000044595	Transcript	ENSMUST00000124038	retained_intron	rs31790950
18:36767035-36767035	upstream_gene_variant	MODIFIER	Dnd1	ENSMUSG00000044595	Transcript	ENSMUST00000155827	retained_intron	rs31790950
18:37846857-37846857	downstream_gene_variant	MODIFIER	Pcdhgb6	ENSMUSG00000103088	Transcript	ENSMUST00000003599	protein_coding	rs31758919
18:37846857-37846857	intron_variant	MODIFIER	Diaph1	ENSMUSG00000024456	Transcript	ENSMUST00000025337	protein_coding	rs31758919
18:37846857-37846857	downstream_gene_variant	MODIFIER	Pcdhga12	ENSMUSG00000102428	Transcript	ENSMUST00000044851	protein_coding	rs31758919
18:37846857-37846857	downstream_gene_variant	MODIFIER	Pcdhgc5	ENSMUSG00000102543	Transcript	ENSMUST00000055935	protein_coding	rs31758919
18:37846857-37846857	downstream_gene_variant	MODIFIER	Pcdhga11	ENSMUSG00000102742	Transcript	ENSMUST00000061279	protein_coding	rs31758919
18:37846857-37846857	downstream_gene_variant	MODIFIER	Pcdhgc4	ENSMUSG00000023036	Transcript	ENSMUST00000066140	protein_coding	rs31758919
18:37846857-37846857	downstream_gene_variant	MODIFIER	Pcdhga8	ENSMUSG00000103897	Transcript	ENSMUST00000066149	protein_coding	rs31758919
18:37846857-37846857	downstream_gene_variant	MODIFIER	Pcdhga3	ENSMUSG00000104346	Transcript	ENSMUST00000073447	protein_coding	rs31758919
18:37846857-37846857	downstream_gene_variant	MODIFIER	Pcdhgc3	ENSMUSG00000102918	Transcript	ENSMUST00000076807	protein_coding	rs31758919
18:37846857-37846857	intron_variant	MODIFIER	Diaph1	ENSMUSG00000024456	Transcript	ENSMUST00000080033	protein_coding	rs31758919
18:37846857-37846857	downstream_gene_variant	MODIFIER	Pcdhga9	ENSMUSG00000102440	Transcript	ENSMUST00000091935	protein_coding	rs31758919
18:37846857-37846857	intron_variant	MODIFIER	Diaph1	ENSMUSG00000024456	Transcript	ENSMUST00000115629	protein_coding	rs31758919
18:37846857-37846857	intron_variant	MODIFIER	Diaph1	ENSMUSG00000024456	Transcript	ENSMUST00000115631	protein_coding	rs31758919
18:37846857-37846857	intron_variant	MODIFIER	Diaph1	ENSMUSG00000024456	Transcript	ENSMUST00000115634	protein_coding	rs31758919
18:37846857-37846857	intron_variant,non_coding_trans	MODIFIER	Diaph1	ENSMUSG00000024456	Transcript	ENSMUST00000127346	processed_transcript	rs31758919
18:37846857-37846857	downstream_gene_variant	MODIFIER	Pcdhgc3	ENSMUSG00000102918	Transcript	ENSMUST00000192103	nonsense_mediated_decay	rs31758919
18:37846857-37846857	downstream_gene_variant	MODIFIER	Pcdhga7	ENSMUSG00000103472	Transcript	ENSMUST00000192511	protein_coding	rs31758919
18:37846857-37846857	downstream_gene_variant	MODIFIER	Pcdhgb5	ENSMUSG00000103749	Transcript	ENSMUST00000192535	protein_coding	rs31758919
18:37846857-37846857	downstream_gene_variant	MODIFIER	Pcdhgb1	ENSMUSG00000103037	Transcript	ENSMUST00000192931	protein_coding	rs31758919
18:37846857-37846857	downstream_gene_variant	MODIFIER	Pcdhga10	ENSMUSG00000102222	Transcript	ENSMUST00000193404	protein_coding	rs31758919
18:37846857-37846857	downstream_gene_variant	MODIFIER	Pcdhga5	ENSMUSG00000103567	Transcript	ENSMUST00000193414	protein_coding	rs31758919
18:37846857-37846857	downstream_gene_variant	MODIFIER	Pcdhga2	ENSMUSG00000103332	Transcript	ENSMUST00000193869	protein_coding	rs31758919
18:37846857-37846857	downstream_gene_variant	MODIFIER	Pcdhga1	ENSMUSG00000103144	Transcript	ENSMUST00000194190	protein_coding	rs31758919
18:37846857-37846857	downstream_gene_variant	MODIFIER	Pcdhga4	ENSMUSG00000103677	Transcript	ENSMUST00000194418	protein_coding	rs31758919
18:37846857-37846857	downstream_gene_variant	MODIFIER	Pcdhgb7	ENSMUSG00000104063	Transcript	ENSMUST00000194928	protein_coding	rs31758919
18:37846857-37846857	downstream_gene_variant	MODIFIER	Pcdhgb2	ENSMUSG00000102748	Transcript	ENSMUST00000195112	protein_coding	rs31758919
18:37846857-37846857	downstream_gene_variant	MODIFIER	Pcdhgc4	ENSMUSG00000023036	Transcript	ENSMUST00000195239	protein_coding	rs31758919
18:37846857-37846857	downstream_gene_variant	MODIFIER	Pcdhgb4	ENSMUSG00000103585	Transcript	ENSMUST00000195363	protein_coding	rs31758919
18:37846857-37846857	downstream_gene_variant	MODIFIER	Pcdhgb8	ENSMUSG00000103081	Transcript	ENSMUST00000195764	polymorphic_pseudogene	rs31758919
18:37846857-37846857	downstream_gene_variant	MODIFIER	Pcdhga6	ENSMUSG00000103793	Transcript	ENSMUST00000195823	protein_coding	rs31758919
18:37846996-37846996	intron_variant	MODIFIER	Diaph1	ENSMUSG00000024456	Transcript	ENSMUST00000025337	protein_coding	rs52158212
18:37846996-37846996	intron_variant	MODIFIER	Diaph1	ENSMUSG00000024456	Transcript	ENSMUST00000080033	protein_coding	rs52158212
18:37846996-37846996	intron_variant	MODIFIER	Diaph1	ENSMUSG00000024456	Transcript	ENSMUST00000115629	protein_coding	rs52158212
18:37846996-37846996	intron_variant	MODIFIER	Diaph1	ENSMUSG00000024456	Transcript	ENSMUST00000115631	protein_coding	rs52158212
18:37846996-37846996	intron_variant	MODIFIER	Diaph1	ENSMUSG00000024456	Transcript	ENSMUST00000115634	protein_coding	rs52158212
18:37846996-37846996	intron_variant,non_coding_trans	MODIFIER	Diaph1	ENSMUSG00000024456	Transcript	ENSMUST00000127346	processed_transcript	rs52158212
18:37851507-37851508	intron_variant	MODIFIER	Diaph1	ENSMUSG00000024456	Transcript	ENSMUST00000025337	protein_coding	rs225434146
18:37851507-37851508	intron_variant	MODIFIER	Diaph1	ENSMUSG00000024456	Transcript	ENSMUST00000080033	protein_coding	rs225434146
18:37851507-37851508	intron_variant	MODIFIER	Diaph1	ENSMUSG00000024456	Transcript	ENSMUST00000115629	protein_coding	rs225434146
18:37851507-37851508	intron_variant	MODIFIER	Diaph1	ENSMUSG00000024456	Transcript	ENSMUST00000115631	protein_coding	rs225434146
18:37851507-37851508	intron_variant	MODIFIER	Diaph1	ENSMUSG00000024456	Transcript	ENSMUST00000115634	protein_coding	rs225434146
18:37851507-37851508	downstream_gene_variant	MODIFIER	Diaph1	ENSMUSG00000024456	Transcript	ENSMUST00000124822	retained_intron	rs225434146
18:37851507-37851508	intron_variant,non_coding_trans	MODIFIER	Diaph1	ENSMUSG00000024456	Transcript	ENSMUST00000127346	processed_transcript	rs225434146
18:37851507-37851508	downstream_gene_variant	MODIFIER	Mir6979	ENSMUSG00000098706	Transcript	ENSMUST00000183927	miRNA	rs225434146
18:37854141-37854141	intron_variant	MODIFIER	Diaph1	ENSMUSG00000024456	Transcript	ENSMUST00000025337	protein_coding	rs31758037
18:37854141-37854141	intron_variant	MODIFIER	Diaph1	ENSMUSG00000024456	Transcript	ENSMUST00000080033	protein_coding	rs31758037
18:37854141-37854141	intron_variant	MODIFIER	Diaph1	ENSMUSG00000024456	Transcript	ENSMUST00000115629	protein_coding	rs31758037
18:37854141-37854141	intron_variant	MODIFIER	Diaph1	ENSMUSG00000024456	Transcript	ENSMUST00000115631	protein_coding	rs31758037
18:37854141-37854141	intron_variant	MODIFIER	Diaph1	ENSMUSG00000024456	Transcript	ENSMUST00000115634	protein_coding	rs31758037
18:37854141-37854141	downstream_gene_variant	MODIFIER	Diaph1	ENSMUSG00000024456	Transcript	ENSMUST00000124822	retained_intron	rs31758037
18:37854141-37854141	upstream_gene_variant	MODIFIER	Diaph1	ENSMUSG00000024456	Transcript	ENSMUST00000127346	processed_transcript	rs31758037
18:37854141-37854141	downstream_gene_variant	MODIFIER	Mir6979	ENSMUSG00000098706	Transcript	ENSMUST00000183927	miRNA	rs31758037
18:37974779-37974779	intron_variant	MODIFIER	Arap3	ENSMUSG00000024451	Transcript	ENSMUST00000042944	protein_coding	rs229222587
18:37974779-37974779	upstream_gene_variant	MODIFIER	Mir6981	ENSMUSG00000098608	Transcript	ENSMUST00000184293	miRNA	rs229222587
18:37990624-37990624	splice_region_variant,intron_vari	LOW	Arap3	ENSMUSG00000024451	Transcript	ENSMUST00000042944	protein_coding	rs233848426
18:37990624-37990624	downstream_gene_variant	MODIFIER	Mir6980	ENSMUSG00000098297	Transcript	ENSMUST00000185063	miRNA	rs233848426
18:38259100-38259100	intron_variant	MODIFIER	Dele1	ENSMUSG00000024442	Transcript	ENSMUST00000025314	protein_coding	rs31742830
18:38259241-38259241	intron_variant	MODIFIER	Dele1	ENSMUSG00000024442	Transcript	ENSMUST00000025314	protein_coding	rs31742827
18:38260773-38260773	intron_variant	MODIFIER	Dele1	ENSMUSG00000024442	Transcript	ENSMUST00000025314	protein_coding	rs51623195
18:38307632-38307632	intron_variant	MODIFIER	Rnf14	ENSMUSG00000060450	Transcript	ENSMUST00000072376	protein_coding	rs237256251
18:38307632-38307632	intron_variant	MODIFIER	Rnf14	ENSMUSG00000060450	Transcript	ENSMUST00000170811	protein_coding	rs237256251
18:38307632-38307632	intron_variant	MODIFIER	Rnf14	ENSMUSG00000060450	Transcript	ENSMUST00000171461	protein_coding	rs237256251
18:38307632-38307632	regulatory_region_variant	MODIFIER	-	-	RegulatoryFeature	ENSMUSR00000605253	enhancer	rs237256251
18:38318748-38318748	downstream_gene_variant	MODIFIER	Rnf14	ENSMUSG00000060450	Transcript	ENSMUST00000072376	protein_coding	rs263376449
18:38318748-38318748	downstream_gene_variant	MODIFIER	Rnf14	ENSMUSG00000060450	Transcript	ENSMUST00000170811	protein_coding	rs263376449
18:38318748-38318748	downstream_gene_variant	MODIFIER	Rnf14	ENSMUSG00000060450	Transcript	ENSMUST00000171461	protein_coding	rs263376449
18:38318782-38318782	downstream_gene_variant	MODIFIER	Rnf14	ENSMUSG00000060450	Transcript	ENSMUST00000072376	protein_coding	rs237486057

18:38318782-38318782	downstream_gene_variant	MODIFIER	Rnf14	ENSMUSG00000060450	Transcript	ENSMUST00000170811	protein_coding	rs237486057
18:38318782-38318782	downstream_gene_variant	MODIFIER	Rnf14	ENSMUSG00000060450	Transcript	ENSMUST00000171461	protein_coding	rs237486057
18:38331776-38331776	intron_variant	MODIFIER	Gnpda1	ENSMUSG00000052102	Transcript	ENSMUST00000063814	protein_coding	rs257077529
18:38331776-38331776	downstream_gene_variant	MODIFIER	Gnpda1	ENSMUSG00000052102	Transcript	ENSMUST00000129062	retained_intron	rs257077529
18:38331776-38331776	downstream_gene_variant	MODIFIER	Gnpda1	ENSMUSG00000052102	Transcript	ENSMUST00000139885	protein_coding	rs257077529
18:38331776-38331776	intron_variant,non_coding_trans	MODIFIER	Gnpda1	ENSMUSG00000052102	Transcript	ENSMUST00000153291	processed_transcript	rs257077529
18:38332792-38332792	intron_variant	MODIFIER	Gnpda1	ENSMUSG00000052102	Transcript	ENSMUST00000063814	protein_coding	rs245526608
18:38332792-38332792	downstream_gene_variant	MODIFIER	Gnpda1	ENSMUSG00000052102	Transcript	ENSMUST00000129062	retained_intron	rs245526608
18:38332792-38332792	downstream_gene_variant	MODIFIER	Gnpda1	ENSMUSG00000052102	Transcript	ENSMUST00000139885	protein_coding	rs245526608
18:38332792-38332792	upstream_gene_variant	MODIFIER	Gnpda1	ENSMUSG00000052102	Transcript	ENSMUST00000153291	processed_transcript	rs245526608
18:38332795-38332795	intron_variant	MODIFIER	Gnpda1	ENSMUSG00000052102	Transcript	ENSMUST00000063814	protein_coding	rs31733638
18:38332795-38332795	downstream_gene_variant	MODIFIER	Gnpda1	ENSMUSG00000052102	Transcript	ENSMUST00000129062	retained_intron	rs31733638
18:38332795-38332795	downstream_gene_variant	MODIFIER	Gnpda1	ENSMUSG00000052102	Transcript	ENSMUST00000139885	protein_coding	rs31733638
18:38332795-38332795	upstream_gene_variant	MODIFIER	Gnpda1	ENSMUSG00000052102	Transcript	ENSMUST00000153291	processed_transcript	rs31733638
18:38334603-38334603	intron_variant	MODIFIER	Gnpda1	ENSMUSG00000052102	Transcript	ENSMUST00000063814	protein_coding	rs247265927
18:38334603-38334603	intron_variant,non_coding_trans	MODIFIER	Gnpda1	ENSMUSG00000052102	Transcript	ENSMUST00000129062	retained_intron	rs247265927
18:38334603-38334603	intron_variant	MODIFIER	Gnpda1	ENSMUSG00000052102	Transcript	ENSMUST00000139885	protein_coding	rs247265927
18:38334603-38334603	upstream_gene_variant	MODIFIER	Gnpda1	ENSMUSG00000052102	Transcript	ENSMUST00000153291	processed_transcript	rs247265927
18:38334608-38334608	intron_variant	MODIFIER	Gnpda1	ENSMUSG00000052102	Transcript	ENSMUST00000063814	protein_coding	rs31741649
18:38334608-38334608	intron_variant,non_coding_trans	MODIFIER	Gnpda1	ENSMUSG00000052102	Transcript	ENSMUST00000129062	retained_intron	rs31741649
18:38334608-38334608	intron_variant	MODIFIER	Gnpda1	ENSMUSG00000052102	Transcript	ENSMUST00000139885	protein_coding	rs31741649
18:38334608-38334608	upstream_gene_variant	MODIFIER	Gnpda1	ENSMUSG00000052102	Transcript	ENSMUST00000153291	processed_transcript	rs31741649
18:38336113-38336113	intron_variant	MODIFIER	Gnpda1	ENSMUSG00000052102	Transcript	ENSMUST00000063814	protein_coding	rs31740089
18:38336113-38336113	upstream_gene_variant	MODIFIER	Gnpda1	ENSMUSG00000052102	Transcript	ENSMUST00000129062	retained_intron	rs31740089
18:38336113-38336113	intron_variant	MODIFIER	Gnpda1	ENSMUSG00000052102	Transcript	ENSMUST00000139885	protein_coding	rs31740089
18:38336113-38336113	upstream_gene_variant	MODIFIER	Gnpda1	ENSMUSG00000052102	Transcript	ENSMUST00000153291	processed_transcript	rs31740089
18:38359699-38359699	downstream_gene_variant	MODIFIER	Gm24690	ENSMUSG00000088384	Transcript	ENSMUST00000157759	rRNA	rs231957407
18:38359706-38359706	downstream_gene_variant	MODIFIER	Gm24690	ENSMUSG00000088384	Transcript	ENSMUST00000157759	rRNA	rs242104995
18:38449000-38449000	intron_variant	MODIFIER	Ndfip1	ENSMUSG00000024425	Transcript	ENSMUST00000025293	protein_coding	rs263737500
18:38449005-38449005	intron_variant	MODIFIER	Ndfip1	ENSMUSG00000024425	Transcript	ENSMUST00000025293	protein_coding	rs226631525
18:38450570-38450570	intron_variant	MODIFIER	Ndfip1	ENSMUSG00000024425	Transcript	ENSMUST00000025293	protein_coding	rs264083262
18:38451072-38451072	intron_variant	MODIFIER	Ndfip1	ENSMUSG00000024425	Transcript	ENSMUST00000025293	protein_coding	rs50128826
18:38460474-38460474	intron_variant	MODIFIER	Ndfip1	ENSMUSG00000024425	Transcript	ENSMUST00000025293	protein_coding	rs224678089
18:38461008-38461008	intron_variant	MODIFIER	Ndfip1	ENSMUSG00000024425	Transcript	ENSMUST00000025293	protein_coding	rs31730914
18:39357853-39357853	intron_variant	MODIFIER	Arhgap26	ENSMUSG00000036452	Transcript	ENSMUST00000097593	protein_coding	rs46012708
18:39357853-39357853	upstream_gene_variant	MODIFIER	Arhgap26	ENSMUSG00000036452	Transcript	ENSMUST00000133247	processed_transcript	rs46012708
18:39357853-39357853	intron_variant	MODIFIER	Arhgap26	ENSMUSG00000036452	Transcript	ENSMUST00000137497	protein_coding	rs46012708
18:39357853-39357853	intron_variant	MODIFIER	Arhgap26	ENSMUSG00000036452	Transcript	ENSMUST00000141058	protein_coding	rs46012708
18:39357853-39357853	intron_variant,NMD_transcript_v	MODIFIER	Arhgap26	ENSMUSG00000036452	Transcript	ENSMUST00000151757	nonsense_mediated_decay	rs46012708
18:39357853-39357853	intron_variant	MODIFIER	Arhgap26	ENSMUSG00000036452	Transcript	ENSMUST00000154551	protein_coding	rs46012708
18:39357853-39357853	intron_variant	MODIFIER	Arhgap26	ENSMUSG00000036452	Transcript	ENSMUST00000155576	protein_coding	rs46012708
18:39357853-39357853	regulatory_region_variant	MODIFIER	-	-	RegulatoryFeature	ENSMUSR00000605428	enhancer	rs46012708
18:50057718-50057718	intron_variant	MODIFIER	Tnfaip8	ENSMUSG00000062210	Transcript	ENSMUST00000126666	protein_coding	rs32092848
18:50057718-50057718	intron_variant	MODIFIER	Tnfaip8	ENSMUSG00000062210	Transcript	ENSMUST00000128377	protein_coding	rs32092848
18:50057718-50057718	upstream_gene_variant	MODIFIER	C030005K06Rik	ENSMUSG00000086600	Transcript	ENSMUST00000129164	processed_transcript	rs32092848
18:50057718-50057718	intron_variant	MODIFIER	Tnfaip8	ENSMUSG00000062210	Transcript	ENSMUST00000134348	protein_coding	rs32092848
18:50057718-50057718	intron_variant	MODIFIER	Tnfaip8	ENSMUSG00000062210	Transcript	ENSMUST00000145726	protein_coding	rs32092848
18:50057718-50057718	upstream_gene_variant	MODIFIER	C030005K06Rik	ENSMUSG00000086600	Transcript	ENSMUST00000147073	processed_transcript	rs32092848
18:50057718-50057718	intron_variant	MODIFIER	Tnfaip8	ENSMUSG00000062210	Transcript	ENSMUST00000148159	protein_coding	rs32092848
18:50057718-50057718	intron_variant	MODIFIER	Tnfaip8	ENSMUSG00000062210	Transcript	ENSMUST00000148989	protein_coding	rs32092848
18:50057718-50057718	upstream_gene_variant	MODIFIER	C030005K06Rik	ENSMUSG00000086600	Transcript	ENSMUST00000151719	processed_transcript	rs32092848
18:50057718-50057718	upstream_gene_variant	MODIFIER	C030005K06Rik	ENSMUSG00000086600	Transcript	ENSMUST00000153361	processed_transcript	rs32092848
18:50057718-50057718	intron_variant,non_coding_trans	MODIFIER	Tnfaip8	ENSMUSG00000062210	Transcript	ENSMUST00000153545	processed_transcript	rs32092848
18:50057718-50057718	intron_variant	MODIFIER	Tnfaip8	ENSMUSG00000062210	Transcript	ENSMUST00000153873	protein_coding	rs32092848
18:50057718-50057718	downstream_gene_variant	MODIFIER	Tnfaip8	ENSMUSG00000062210	Transcript	ENSMUST00000155403	retained_intron	rs32092848
18:50057718-50057718	intron_variant	MODIFIER	Tnfaip8	ENSMUSG00000062210	Transcript	ENSMUST00000179937	protein_coding	rs32092848
18:50058271-50058271	intron_variant	MODIFIER	Tnfaip8	ENSMUSG00000062210	Transcript	ENSMUST00000126666	protein_coding	rs32091880
18:50058271-50058271	intron_variant	MODIFIER	Tnfaip8	ENSMUSG00000062210	Transcript	ENSMUST00000128377	protein_coding	rs32091880
18:50058271-50058271	upstream_gene_variant	MODIFIER	C030005K06Rik	ENSMUSG00000086600	Transcript	ENSMUST00000129164	processed_transcript	rs32091880
18:50058271-50058271	intron_variant	MODIFIER	Tnfaip8	ENSMUSG00000062210	Transcript	ENSMUST00000134348	protein_coding	rs32091880
18:50058271-50058271	intron_variant	MODIFIER	Tnfaip8	ENSMUSG00000062210	Transcript	ENSMUST00000145726	protein_coding	rs32091880
18:50058271-50058271	upstream_gene_variant	MODIFIER	C030005K06Rik	ENSMUSG00000086600	Transcript	ENSMUST00000147073	processed_transcript	rs32091880
18:50058271-50058271	intron_variant	MODIFIER	Tnfaip8	ENSMUSG00000062210	Transcript	ENSMUST00000148159	protein_coding	rs32091880
18:50058271-50058271	intron_variant	MODIFIER	Tnfaip8	ENSMUSG00000062210	Transcript	ENSMUST00000148989	protein_coding	rs32091880
18:50058271-50058271	upstream_gene_variant	MODIFIER	C030005K06Rik	ENSMUSG00000086600	Transcript	ENSMUST00000151719	processed_transcript	rs32091880
18:50058271-50058271	upstream_gene_variant	MODIFIER	C030005K06Rik	ENSMUSG00000086600	Transcript	ENSMUST00000153361	processed_transcript	rs32091880
18:50058271-50058271	intron_variant,non_coding_trans	MODIFIER	Tnfaip8	ENSMUSG00000062210	Transcript	ENSMUST00000153545	processed_transcript	rs32091880
18:50058271-50058271	intron_variant	MODIFIER	Tnfaip8	ENSMUSG00000062210	Transcript	ENSMUST00000153873	protein_coding	rs32091880
18:50058271-50058271	downstream_gene_variant	MODIFIER	Tnfaip8	ENSMUSG00000062210	Transcript	ENSMUST00000155403	retained_intron	rs32091880

18:50058271-50058271	intron_variant	MODIFIER	Tnfaip8	ENSMUSG00000062210	Transcript	ENSMUST00000179937	protein_coding	rs32091880
18:50058271-50058271	regulatory_region_variant	MODIFIER	-	-	RegulatoryFeature	ENSMUSR00000606413	enhancer	rs32091880
18:50063188-50063188	intron_variant	MODIFIER	Tnfaip8	ENSMUSG00000062210	Transcript	ENSMUST00000126666	protein_coding	rs32092639
18:50063188-50063188	intron_variant	MODIFIER	Tnfaip8	ENSMUSG00000062210	Transcript	ENSMUST00000128377	protein_coding	rs32092639
18:50063188-50063188	intron_variant	MODIFIER	Tnfaip8	ENSMUSG00000062210	Transcript	ENSMUST00000134348	protein_coding	rs32092639
18:50063188-50063188	intron_variant	MODIFIER	Tnfaip8	ENSMUSG00000062210	Transcript	ENSMUST00000145726	protein_coding	rs32092639
18:50063188-50063188	intron_variant	MODIFIER	Tnfaip8	ENSMUSG00000062210	Transcript	ENSMUST00000148159	protein_coding	rs32092639
18:50063188-50063188	intron_variant	MODIFIER	Tnfaip8	ENSMUSG00000062210	Transcript	ENSMUST00000148989	protein_coding	rs32092639
18:50063188-50063188	intron_variant,non_coding_trans	MODIFIER	Tnfaip8	ENSMUSG00000062210	Transcript	ENSMUST00000153545	processed_transcript	rs32092639
18:50063188-50063188	intron_variant	MODIFIER	Tnfaip8	ENSMUSG00000062210	Transcript	ENSMUST00000153873	protein_coding	rs32092639
18:50063188-50063188	intron_variant	MODIFIER	Tnfaip8	ENSMUSG00000062210	Transcript	ENSMUST00000179937	protein_coding	rs32092639
18:50063188-50063188	regulatory_region_variant	MODIFIER	-	-	RegulatoryFeature	ENSMUSR00000134559	promoter_flanking_region	rs32092639
18:50063216-50063216	intron_variant	MODIFIER	Tnfaip8	ENSMUSG00000062210	Transcript	ENSMUST00000126666	protein_coding	rs32092637
18:50063216-50063216	intron_variant	MODIFIER	Tnfaip8	ENSMUSG00000062210	Transcript	ENSMUST00000128377	protein_coding	rs32092637
18:50063216-50063216	intron_variant	MODIFIER	Tnfaip8	ENSMUSG00000062210	Transcript	ENSMUST00000134348	protein_coding	rs32092637
18:50063216-50063216	intron_variant	MODIFIER	Tnfaip8	ENSMUSG00000062210	Transcript	ENSMUST00000145726	protein_coding	rs32092637
18:50063216-50063216	intron_variant	MODIFIER	Tnfaip8	ENSMUSG00000062210	Transcript	ENSMUST00000148159	protein_coding	rs32092637
18:50063216-50063216	intron_variant	MODIFIER	Tnfaip8	ENSMUSG00000062210	Transcript	ENSMUST00000148989	protein_coding	rs32092637
18:50063216-50063216	intron_variant,non_coding_trans	MODIFIER	Tnfaip8	ENSMUSG00000062210	Transcript	ENSMUST00000153545	processed_transcript	rs32092637
18:50063216-50063216	intron_variant	MODIFIER	Tnfaip8	ENSMUSG00000062210	Transcript	ENSMUST00000153873	protein_coding	rs32092637
18:50063216-50063216	intron_variant	MODIFIER	Tnfaip8	ENSMUSG00000062210	Transcript	ENSMUST00000179937	protein_coding	rs32092637
18:50063216-50063216	regulatory_region_variant	MODIFIER	-	-	RegulatoryFeature	ENSMUSR00000134559	promoter_flanking_region	rs32092637
18:50067473-50067473	intron_variant	MODIFIER	Tnfaip8	ENSMUSG00000062210	Transcript	ENSMUST00000126666	protein_coding	rs50596137
18:50067473-50067473	intron_variant	MODIFIER	Tnfaip8	ENSMUSG00000062210	Transcript	ENSMUST00000128377	protein_coding	rs50596137
18:50067473-50067473	intron_variant	MODIFIER	Tnfaip8	ENSMUSG00000062210	Transcript	ENSMUST00000134348	protein_coding	rs50596137
18:50067473-50067473	intron_variant	MODIFIER	Tnfaip8	ENSMUSG00000062210	Transcript	ENSMUST00000145726	protein_coding	rs50596137
18:50067473-50067473	intron_variant	MODIFIER	Tnfaip8	ENSMUSG00000062210	Transcript	ENSMUST00000148159	protein_coding	rs50596137
18:50067473-50067473	intron_variant	MODIFIER	Tnfaip8	ENSMUSG00000062210	Transcript	ENSMUST00000148989	protein_coding	rs50596137
18:50067473-50067473	intron_variant,non_coding_trans	MODIFIER	Tnfaip8	ENSMUSG00000062210	Transcript	ENSMUST00000153545	processed_transcript	rs50596137
18:50067473-50067473	intron_variant	MODIFIER	Tnfaip8	ENSMUSG00000062210	Transcript	ENSMUST00000153873	protein_coding	rs50596137
18:50067473-50067473	intron_variant	MODIFIER	Tnfaip8	ENSMUSG00000062210	Transcript	ENSMUST00000179937	protein_coding	rs50596137
18:50067474-50067474	intron_variant	MODIFIER	Tnfaip8	ENSMUSG00000062210	Transcript	ENSMUST00000126666	protein_coding	rs49144351
18:50067474-50067474	intron_variant	MODIFIER	Tnfaip8	ENSMUSG00000062210	Transcript	ENSMUST00000128377	protein_coding	rs49144351
18:50067474-50067474	intron_variant	MODIFIER	Tnfaip8	ENSMUSG00000062210	Transcript	ENSMUST00000134348	protein_coding	rs49144351
18:50067474-50067474	intron_variant	MODIFIER	Tnfaip8	ENSMUSG00000062210	Transcript	ENSMUST00000145726	protein_coding	rs49144351
18:50067474-50067474	intron_variant	MODIFIER	Tnfaip8	ENSMUSG00000062210	Transcript	ENSMUST00000148159	protein_coding	rs49144351
18:50067474-50067474	intron_variant	MODIFIER	Tnfaip8	ENSMUSG00000062210	Transcript	ENSMUST00000148989	protein_coding	rs49144351
18:50067474-50067474	intron_variant,non_coding_trans	MODIFIER	Tnfaip8	ENSMUSG00000062210	Transcript	ENSMUST00000153545	processed_transcript	rs49144351
18:50067474-50067474	intron_variant	MODIFIER	Tnfaip8	ENSMUSG00000062210	Transcript	ENSMUST00000153873	protein_coding	rs49144351
18:50067474-50067474	intron_variant	MODIFIER	Tnfaip8	ENSMUSG00000062210	Transcript	ENSMUST00000179937	protein_coding	rs49144351
18:5063594-5063594	missense_variant	MODERATE	Svil	ENSMUSG00000024236	Transcript	ENSMUST00000025079	protein_coding	rs107684718
18:5063594-5063594	upstream_gene_variant	MODIFIER	Svil	ENSMUSG00000024236	Transcript	ENSMUST00000125512	nonsense_mediated_decay	rs107684718
18:5063594-5063594	missense_variant	MODERATE	Svil	ENSMUSG00000024236	Transcript	ENSMUST00000126977	protein_coding	rs107684718
18:5063594-5063594	missense_variant	MODERATE	Svil	ENSMUSG00000024236	Transcript	ENSMUST00000127297	protein_coding	rs107684718
18:5063594-5063594	missense_variant,NMD_transcrip	MODERATE	Svil	ENSMUSG00000024236	Transcript	ENSMUST00000131609	nonsense_mediated_decay	rs107684718
18:5063594-5063594	missense_variant	MODERATE	Svil	ENSMUSG00000024236	Transcript	ENSMUST00000140448	protein_coding	rs107684718
18:5063594-5063594	missense_variant	MODERATE	Svil	ENSMUSG00000024236	Transcript	ENSMUST00000143254	protein_coding	rs107684718
18:5063594-5063594	missense_variant	MODERATE	Svil	ENSMUSG00000024236	Transcript	ENSMUST00000210707	protein_coding	rs107684718
18:53383253-53383253	intron_variant	MODIFIER	Snx24	ENSMUSG00000024535	Transcript	ENSMUST00000025417	protein_coding	rs32106262
18:53383253-53383253	intron_variant	MODIFIER	Snx24	ENSMUSG00000024535	Transcript	ENSMUST00000165032	protein_coding	rs32106262
18:53386456-53386456	intron_variant	MODIFIER	Snx24	ENSMUSG00000024535	Transcript	ENSMUST00000025417	protein_coding	rs29676818
18:53386456-53386456	intron_variant	MODIFIER	Snx24	ENSMUSG00000024535	Transcript	ENSMUST00000165032	protein_coding	rs29676818
18:53387902-53387902	intron_variant	MODIFIER	Snx24	ENSMUSG00000024535	Transcript	ENSMUST00000025417	protein_coding	rs29562370
18:53387902-53387902	intron_variant	MODIFIER	Snx24	ENSMUSG00000024535	Transcript	ENSMUST00000165032	protein_coding	rs29562370
18:57384338-57384338	intron_variant	MODIFIER	Prrc1	ENSMUSG00000024594	Transcript	ENSMUST00000025490	protein_coding	rs29870199
18:57384338-57384338	intron_variant,non_coding_trans	MODIFIER	4930511M06Rik	ENSMUSG00000086607	Transcript	ENSMUST00000130696	processed_transcript	rs29870199
18:57384338-57384338	regulatory_region_variant	MODIFIER	-	-	RegulatoryFeature	ENSMUSR00000607310	open_chromatin_region	rs29870199
18:57388230-57388230	intron_variant	MODIFIER	Prrc1	ENSMUSG00000024594	Transcript	ENSMUST00000025490	protein_coding	rs29679547
18:57388230-57388230	intron_variant,non_coding_trans	MODIFIER	4930511M06Rik	ENSMUSG00000086607	Transcript	ENSMUST00000130696	processed_transcript	rs29679547
18:57391477-57391477	3_prime_UTR_variant	MODIFIER	Prrc1	ENSMUSG00000024594	Transcript	ENSMUST00000025490	protein_coding	rs257071317
18:57391477-57391477	intron_variant,non_coding_trans	MODIFIER	4930511M06Rik	ENSMUSG00000086607	Transcript	ENSMUST00000130696	processed_transcript	rs257071317
18:60392083-60392083	3_prime_UTR_variant	MODIFIER	Iigp1	ENSMUSG00000054072	Transcript	ENSMUST00000032473	protein_coding	rs387859917
18:60392083-60392083	3_prime_UTR_variant	MODIFIER	Iigp1	ENSMUSG00000054072	Transcript	ENSMUST00000066912	protein_coding	rs387859917
18:60817338-60817338	downstream_gene_variant	MODIFIER	Cd74	ENSMUSG00000024610	Transcript	ENSMUST00000050487	protein_coding	rs264897002
18:60817338-60817338	downstream_gene_variant	MODIFIER	Cd74	ENSMUSG00000024610	Transcript	ENSMUST00000097563	protein_coding	rs264897002
18:60817338-60817338	intron_variant,NMD_transcript_v	MODIFIER	Tcof1	ENSMUSG00000024613	Transcript	ENSMUST00000163446	nonsense_mediated_decay	rs264897002
18:60817338-60817338	downstream_gene_variant	MODIFIER	Cd74	ENSMUSG00000024610	Transcript	ENSMUST00000167610	protein_coding	rs264897002
18:60817338-60817338	intron_variant	MODIFIER	Tcof1	ENSMUSG00000024613	Transcript	ENSMUST00000175934	protein_coding	rs264897002
18:60817338-60817338	intron_variant	MODIFIER	Tcof1	ENSMUSG00000024613	Transcript	ENSMUST00000176630	protein_coding	rs264897002

18:60817338-60817338	downstream_gene_variant	MODIFIER	Tcof1	ENSMUSG00000024613	Transcript	ENSMUST00000177172	protein_coding	rs264897002
18:60918366-60918366	3_prime_UTR_variant	MODIFIER	Arsi	ENSMUSG00000036412	Transcript	ENSMUST00000040359	protein_coding	rs29862747
18:60918366-60918366	regulatory_region_variant	MODIFIER	-	-	RegulatoryFeature	ENSMUSR00000607735	enhancer	rs29862747
18:61297721-61297721	downstream_gene_variant	MODIFIER	Ppargc1b	ENSMUSG00000033871	Transcript	ENSMUST00000063307	protein_coding	rs29861794
18:61297721-61297721	downstream_gene_variant	MODIFIER	Ppargc1b	ENSMUSG00000033871	Transcript	ENSMUST00000075299	protein_coding	rs29861794
18:61503254-61503254	intron_variant	MODIFIER	Arhgef37	ENSMUSG00000045094	Transcript	ENSMUST00000171629	protein_coding	-
18:6207128-6207128	intron_variant	MODIFIER	Kif5b	ENSMUSG00000006740	Transcript	ENSMUST00000025083	protein_coding	rs29723092
18:6207128-6207128	intron_variant,non_coding_trans	MODIFIER	Kif5b	ENSMUSG00000006740	Transcript	ENSMUST00000168031	retained_intron	rs29723092
18:62187348-62187348	downstream_gene_variant	MODIFIER	Gm9949	ENSMUSG00000054589	Transcript	ENSMUST00000067743	protein_coding	rs37559711
18:62187348-62187348	regulatory_region_variant	MODIFIER	-	-	RegulatoryFeature	ENSMUSR00000377434	promoter_flanking_region	rs37559711
18:62187841-62187841	downstream_gene_variant	MODIFIER	Gm9949	ENSMUSG00000054589	Transcript	ENSMUST00000067743	protein_coding	rs257620084
18:62187841-62187841	regulatory_region_variant	MODIFIER	-	-	RegulatoryFeature	ENSMUSR00000377434	promoter_flanking_region	rs257620084
18:62542607-62542607	intron_variant	MODIFIER	Fbxo38	ENSMUSG00000042211	Transcript	ENSMUST00000048688	protein_coding	rs4138105
18:63666386-63666386	intron_variant	MODIFIER	Txnl1	ENSMUSG00000024583	Transcript	ENSMUST00000025476	protein_coding	rs46365352
18:6445660-6445660	intron_variant	MODIFIER	Epc1	ENSMUSG00000024240	Transcript	ENSMUST00000028100	protein_coding	rs220744388
18:6445660-6445660	intron_variant	MODIFIER	Epc1	ENSMUSG00000024240	Transcript	ENSMUST00000115870	protein_coding	rs220744388
18:6445660-6445660	upstream_gene_variant	MODIFIER	Epc1	ENSMUSG00000024240	Transcript	ENSMUST00000124926	protein_coding	rs220744388
18:64501060-64501060	intron_variant	MODIFIER	Nars	ENSMUSG00000024587	Transcript	ENSMUST00000025483	protein_coding	rs243162240
18:64501060-64501060	regulatory_region_variant	MODIFIER	-	-	RegulatoryFeature	ENSMUSR00000608262	CTCF_binding_site	rs243162240
18:64501060-64501060	regulatory_region_variant	MODIFIER	-	-	RegulatoryFeature	ENSMUSR00000608263	TF_binding_site	rs243162240
18:64501156-64501156	intron_variant	MODIFIER	Nars	ENSMUSG00000024587	Transcript	ENSMUST00000025483	protein_coding	rs39355409
18:64501156-64501156	regulatory_region_variant	MODIFIER	-	-	RegulatoryFeature	ENSMUSR00000608262	CTCF_binding_site	rs39355409
18:64501156-64501156	regulatory_region_variant	MODIFIER	-	-	RegulatoryFeature	ENSMUSR00000608263	TF_binding_site	rs39355409
18:65444344-65444344	intron_variant	MODIFIER	Malt1	ENSMUSG00000032688	Transcript	ENSMUST00000049248	protein_coding	rs48030056
18:65444344-65444344	intron_variant	MODIFIER	Malt1	ENSMUSG00000032688	Transcript	ENSMUST00000224056	protein_coding	rs48030056
18:65444344-65444344	upstream_gene_variant	MODIFIER	Malt1	ENSMUSG00000032688	Transcript	ENSMUST00000224229	retained_intron	rs48030056
18:65444344-65444344	intron_variant,non_coding_trans	MODIFIER	Malt1	ENSMUSG00000032688	Transcript	ENSMUST00000224265	processed_transcript	rs48030056
18:65444344-65444344	intron_variant,non_coding_trans	MODIFIER	Malt1	ENSMUSG00000032688	Transcript	ENSMUST00000225085	retained_intron	rs48030056
18:65444344-65444344	regulatory_region_variant	MODIFIER	-	-	RegulatoryFeature	ENSMUSR00000608454	enhancer	rs48030056
18:65455244-65455244	intron_variant	MODIFIER	Malt1	ENSMUSG00000032688	Transcript	ENSMUST00000049248	protein_coding	rs30270946
18:65455244-65455244	intron_variant	MODIFIER	Malt1	ENSMUSG00000032688	Transcript	ENSMUST00000224056	protein_coding	rs30270946
18:65455244-65455244	non_coding_transcript_exon_va	MODIFIER	Malt1	ENSMUSG00000032688	Transcript	ENSMUST00000224229	retained_intron	rs30270946
18:65455244-65455244	intron_variant,non_coding_trans	MODIFIER	Malt1	ENSMUSG00000032688	Transcript	ENSMUST00000224265	processed_transcript	rs30270946
18:65455244-65455244	downstream_gene_variant	MODIFIER	Malt1	ENSMUSG00000032688	Transcript	ENSMUST00000225085	retained_intron	rs30270946
18:65455244-65455244	downstream_gene_variant	MODIFIER	Malt1	ENSMUSG00000032688	Transcript	ENSMUST00000225659	retained_intron	rs30270946
18:67647086-67647086	intron_variant	MODIFIER	Psmg2	ENSMUSG00000024537	Transcript	ENSMUST00000025418	protein_coding	rs237487890
18:67666980-67666980	intron_variant	MODIFIER	Ptpn2	ENSMUSG00000024539	Transcript	ENSMUST00000025420	protein_coding	rs30210988
18:67666980-67666980	intron_variant	MODIFIER	Ptpn2	ENSMUSG00000024539	Transcript	ENSMUST00000120934	protein_coding	rs30210988
18:67666980-67666980	downstream_gene_variant	MODIFIER	Ptpn2	ENSMUSG00000024539	Transcript	ENSMUST00000122412	protein_coding	rs30210988
18:67666980-67666980	regulatory_region_variant	MODIFIER	-	-	RegulatoryFeature	ENSMUSR00000136871	promoter_flanking_region	rs30210988
18:67669025-67669025	intron_variant	MODIFIER	Ptpn2	ENSMUSG00000024539	Transcript	ENSMUST00000025420	protein_coding	rs29775556
18:67669025-67669025	intron_variant	MODIFIER	Ptpn2	ENSMUSG00000024539	Transcript	ENSMUST00000120934	protein_coding	rs29775556
18:67669025-67669025	downstream_gene_variant	MODIFIER	Ptpn2	ENSMUSG00000024539	Transcript	ENSMUST00000122412	protein_coding	rs29775556
18:67669025-67669025	regulatory_region_variant	MODIFIER	-	-	RegulatoryFeature	ENSMUSR00000136872	promoter_flanking_region	rs29775556
18:67671918-67671918	intron_variant	MODIFIER	Ptpn2	ENSMUSG00000024539	Transcript	ENSMUST00000025420	protein_coding	rs30121094
18:67671918-67671918	intron_variant	MODIFIER	Ptpn2	ENSMUSG00000024539	Transcript	ENSMUST00000120934	protein_coding	rs30121094
18:67671918-67671918	3_prime_UTR_variant	MODIFIER	Ptpn2	ENSMUSG00000024539	Transcript	ENSMUST00000122412	protein_coding	rs30121094
18:67671918-67671918	downstream_gene_variant	MODIFIER	Ptpn2	ENSMUSG00000024539	Transcript	ENSMUST00000128169	processed_transcript	rs30121094
18:67789839-67789839	intron_variant	MODIFIER	Seh1l	ENSMUSG00000079614	Transcript	ENSMUST00000025421	protein_coding	rs29541917
18:68449187-68449187	intergenic_variant	MODIFIER	-	-	-	-	-	rs30301945
18:69680109-69680109	intron_variant	MODIFIER	Tcf4	ENSMUSG00000053477	Transcript	ENSMUST00000066717	protein_coding	rs29636191
18:69680109-69680109	intron_variant	MODIFIER	Tcf4	ENSMUSG00000053477	Transcript	ENSMUST00000078486	protein_coding	rs29636191
18:69680109-69680109	intron_variant	MODIFIER	Tcf4	ENSMUSG00000053477	Transcript	ENSMUST00000114977	protein_coding	rs29636191
18:69680109-69680109	intron_variant	MODIFIER	Tcf4	ENSMUSG00000053477	Transcript	ENSMUST00000114978	protein_coding	rs29636191
18:69680109-69680109	intron_variant	MODIFIER	Tcf4	ENSMUSG00000053477	Transcript	ENSMUST00000114980	protein_coding	rs29636191
18:69680109-69680109	intron_variant	MODIFIER	Tcf4	ENSMUSG00000053477	Transcript	ENSMUST00000114982	protein_coding	rs29636191
18:69680109-69680109	intron_variant	MODIFIER	Tcf4	ENSMUSG00000053477	Transcript	ENSMUST00000114985	protein_coding	rs29636191
18:69680109-69680109	upstream_gene_variant	MODIFIER	Tcf4	ENSMUSG00000053477	Transcript	ENSMUST00000136019	retained_intron	rs29636191
18:69680109-69680109	non_coding_transcript_exon_va	MODIFIER	Tcf4	ENSMUSG00000053477	Transcript	ENSMUST00000146908	retained_intron	rs29636191
18:69680109-69680109	downstream_gene_variant	MODIFIER	Tcf4	ENSMUSG00000053477	Transcript	ENSMUST00000201037	protein_coding	rs29636191
18:69680109-69680109	intron_variant	MODIFIER	Tcf4	ENSMUSG00000053477	Transcript	ENSMUST00000201094	protein_coding	rs29636191
18:69680109-69680109	intron_variant	MODIFIER	Tcf4	ENSMUSG00000053477	Transcript	ENSMUST00000201205	protein_coding	rs29636191
18:69680109-69680109	intron_variant	MODIFIER	Tcf4	ENSMUSG00000053477	Transcript	ENSMUST00000201410	protein_coding	rs29636191
18:69680109-69680109	intron_variant	MODIFIER	Tcf4	ENSMUSG00000053477	Transcript	ENSMUST00000201631	protein_coding	rs29636191
18:69680109-69680109	intron_variant	MODIFIER	Tcf4	ENSMUSG00000053477	Transcript	ENSMUST00000201781	protein_coding	rs29636191
18:69680109-69680109	intron_variant	MODIFIER	Tcf4	ENSMUSG00000053477	Transcript	ENSMUST00000202057	protein_coding	rs29636191
18:69680109-69680109	intron_variant	MODIFIER	Tcf4	ENSMUSG00000053477	Transcript	ENSMUST00000202116	protein_coding	rs29636191
18:69680109-69680109	intron_variant	MODIFIER	Tcf4	ENSMUSG00000053477	Transcript	ENSMUST00000202354	protein_coding	rs29636191
18:69680109-69680109	intron_variant	MODIFIER	Tcf4	ENSMUSG00000053477	Transcript	ENSMUST00000202435	protein_coding	rs29636191

18:69680109-69680109	intron_variant	MODIFIER	Tcf4	ENSMUSG00000053477	Transcript	ENSMUST00000202458	protein_coding	rs29636191
18:69680109-69680109	intron_variant	MODIFIER	Tcf4	ENSMUSG00000053477	Transcript	ENSMUST00000202474	protein_coding	rs29636191
18:69680109-69680109	intron_variant	MODIFIER	Tcf4	ENSMUSG00000053477	Transcript	ENSMUST00000202477	protein_coding	rs29636191
18:69680109-69680109	intron_variant	MODIFIER	Tcf4	ENSMUSG00000053477	Transcript	ENSMUST00000202610	protein_coding	rs29636191
18:69680109-69680109	intron_variant	MODIFIER	Tcf4	ENSMUSG00000053477	Transcript	ENSMUST00000202674	protein_coding	rs29636191
18:69680109-69680109	intron_variant,NMD_transcript_v	MODIFIER	Tcf4	ENSMUSG00000053477	Transcript	ENSMUST00000202751	nonsense_mediated_decay	rs29636191
18:69680109-69680109	intron_variant	MODIFIER	Tcf4	ENSMUSG00000053477	Transcript	ENSMUST00000202772	protein_coding	rs29636191
18:69680109-69680109	intron_variant	MODIFIER	Tcf4	ENSMUSG00000053477	Transcript	ENSMUST00000202937	protein_coding	rs29636191
18:69680109-69680109	regulatory_region_variant	MODIFIER	-	-	RegulatoryFeature	ENSMUSR00000609097	enhancer	rs29636191
18:69681869-69681869	intron_variant	MODIFIER	Tcf4	ENSMUSG00000053477	Transcript	ENSMUST00000066717	protein_coding	rs29882287
18:69681869-69681869	intron_variant	MODIFIER	Tcf4	ENSMUSG00000053477	Transcript	ENSMUST00000078486	protein_coding	rs29882287
18:69681869-69681869	intron_variant	MODIFIER	Tcf4	ENSMUSG00000053477	Transcript	ENSMUST00000114977	protein_coding	rs29882287
18:69681869-69681869	intron_variant	MODIFIER	Tcf4	ENSMUSG00000053477	Transcript	ENSMUST00000114978	protein_coding	rs29882287
18:69681869-69681869	intron_variant	MODIFIER	Tcf4	ENSMUSG00000053477	Transcript	ENSMUST00000114980	protein_coding	rs29882287
18:69681869-69681869	intron_variant	MODIFIER	Tcf4	ENSMUSG00000053477	Transcript	ENSMUST00000114982	protein_coding	rs29882287
18:69681869-69681869	intron_variant	MODIFIER	Tcf4	ENSMUSG00000053477	Transcript	ENSMUST00000114985	protein_coding	rs29882287
18:69681869-69681869	upstream_gene_variant	MODIFIER	Tcf4	ENSMUSG00000053477	Transcript	ENSMUST00000136019	retained_intron	rs29882287
18:69681869-69681869	intron_variant,non_coding_trans	MODIFIER	Tcf4	ENSMUSG00000053477	Transcript	ENSMUST00000146908	retained_intron	rs29882287
18:69681869-69681869	intron_variant	MODIFIER	Tcf4	ENSMUSG00000053477	Transcript	ENSMUST00000201094	protein_coding	rs29882287
18:69681869-69681869	intron_variant	MODIFIER	Tcf4	ENSMUSG00000053477	Transcript	ENSMUST00000201205	protein_coding	rs29882287
18:69681869-69681869	intron_variant	MODIFIER	Tcf4	ENSMUSG00000053477	Transcript	ENSMUST00000201410	protein_coding	rs29882287
18:69681869-69681869	intron_variant	MODIFIER	Tcf4	ENSMUSG00000053477	Transcript	ENSMUST00000201631	protein_coding	rs29882287
18:69681869-69681869	intron_variant	MODIFIER	Tcf4	ENSMUSG00000053477	Transcript	ENSMUST00000201781	protein_coding	rs29882287
18:69681869-69681869	intron_variant	MODIFIER	Tcf4	ENSMUSG00000053477	Transcript	ENSMUST00000202057	protein_coding	rs29882287
18:69681869-69681869	intron_variant	MODIFIER	Tcf4	ENSMUSG00000053477	Transcript	ENSMUST00000202116	protein_coding	rs29882287
18:69681869-69681869	intron_variant	MODIFIER	Tcf4	ENSMUSG00000053477	Transcript	ENSMUST00000202354	protein_coding	rs29882287
18:69681869-69681869	intron_variant	MODIFIER	Tcf4	ENSMUSG00000053477	Transcript	ENSMUST00000202435	protein_coding	rs29882287
18:69681869-69681869	intron_variant	MODIFIER	Tcf4	ENSMUSG00000053477	Transcript	ENSMUST00000202458	protein_coding	rs29882287
18:69681869-69681869	intron_variant	MODIFIER	Tcf4	ENSMUSG00000053477	Transcript	ENSMUST00000202474	protein_coding	rs29882287
18:69681869-69681869	intron_variant	MODIFIER	Tcf4	ENSMUSG00000053477	Transcript	ENSMUST00000202477	protein_coding	rs29882287
18:69681869-69681869	intron_variant	MODIFIER	Tcf4	ENSMUSG00000053477	Transcript	ENSMUST00000202610	protein_coding	rs29882287
18:69681869-69681869	intron_variant	MODIFIER	Tcf4	ENSMUSG00000053477	Transcript	ENSMUST00000202674	protein_coding	rs29882287
18:69681869-69681869	intron_variant,NMD_transcript_v	MODIFIER	Tcf4	ENSMUSG00000053477	Transcript	ENSMUST00000202751	nonsense_mediated_decay	rs29882287
18:69681869-69681869	intron_variant	MODIFIER	Tcf4	ENSMUSG00000053477	Transcript	ENSMUST00000202772	protein_coding	rs29882287
18:69681869-69681869	intron_variant	MODIFIER	Tcf4	ENSMUSG00000053477	Transcript	ENSMUST00000202937	protein_coding	rs29882287
18:69682469-69682469	intron_variant	MODIFIER	Tcf4	ENSMUSG00000053477	Transcript	ENSMUST00000066717	protein_coding	rs30260846
18:69682469-69682469	intron_variant	MODIFIER	Tcf4	ENSMUSG00000053477	Transcript	ENSMUST00000078486	protein_coding	rs30260846
18:69682469-69682469	intron_variant	MODIFIER	Tcf4	ENSMUSG00000053477	Transcript	ENSMUST00000114977	protein_coding	rs30260846
18:69682469-69682469	intron_variant	MODIFIER	Tcf4	ENSMUSG00000053477	Transcript	ENSMUST00000114978	protein_coding	rs30260846
18:69682469-69682469	intron_variant	MODIFIER	Tcf4	ENSMUSG00000053477	Transcript	ENSMUST00000114980	protein_coding	rs30260846
18:69682469-69682469	intron_variant	MODIFIER	Tcf4	ENSMUSG00000053477	Transcript	ENSMUST00000114982	protein_coding	rs30260846
18:69682469-69682469	intron_variant	MODIFIER	Tcf4	ENSMUSG00000053477	Transcript	ENSMUST00000114985	protein_coding	rs30260846
18:69682469-69682469	non_coding_transcript_exon_var	MODIFIER	Tcf4	ENSMUSG00000053477	Transcript	ENSMUST00000136019	retained_intron	rs30260846
18:69682469-69682469	intron_variant,non_coding_trans	MODIFIER	Tcf4	ENSMUSG00000053477	Transcript	ENSMUST00000146908	retained_intron	rs30260846
18:69682469-69682469	intron_variant	MODIFIER	Tcf4	ENSMUSG00000053477	Transcript	ENSMUST00000201094	protein_coding	rs30260846
18:69682469-69682469	intron_variant	MODIFIER	Tcf4	ENSMUSG00000053477	Transcript	ENSMUST00000201205	protein_coding	rs30260846
18:69682469-69682469	intron_variant	MODIFIER	Tcf4	ENSMUSG00000053477	Transcript	ENSMUST00000201410	protein_coding	rs30260846
18:69682469-69682469	intron_variant	MODIFIER	Tcf4	ENSMUSG00000053477	Transcript	ENSMUST00000201631	protein_coding	rs30260846
18:69682469-69682469	intron_variant	MODIFIER	Tcf4	ENSMUSG00000053477	Transcript	ENSMUST00000201781	protein_coding	rs30260846
18:69682469-69682469	intron_variant	MODIFIER	Tcf4	ENSMUSG00000053477	Transcript	ENSMUST00000202057	protein_coding	rs30260846
18:69682469-69682469	intron_variant	MODIFIER	Tcf4	ENSMUSG00000053477	Transcript	ENSMUST00000202116	protein_coding	rs30260846
18:69682469-69682469	intron_variant	MODIFIER	Tcf4	ENSMUSG00000053477	Transcript	ENSMUST00000202354	protein_coding	rs30260846
18:69682469-69682469	intron_variant	MODIFIER	Tcf4	ENSMUSG00000053477	Transcript	ENSMUST00000202435	protein_coding	rs30260846
18:69682469-69682469	intron_variant	MODIFIER	Tcf4	ENSMUSG00000053477	Transcript	ENSMUST00000202458	protein_coding	rs30260846
18:69682469-69682469	intron_variant	MODIFIER	Tcf4	ENSMUSG00000053477	Transcript	ENSMUST00000202474	protein_coding	rs30260846
18:69682469-69682469	intron_variant	MODIFIER	Tcf4	ENSMUSG00000053477	Transcript	ENSMUST00000202477	protein_coding	rs30260846
18:69682469-69682469	intron_variant	MODIFIER	Tcf4	ENSMUSG00000053477	Transcript	ENSMUST00000202610	protein_coding	rs30260846
18:69682469-69682469	intron_variant	MODIFIER	Tcf4	ENSMUSG00000053477	Transcript	ENSMUST00000202674	protein_coding	rs30260846
18:69682469-69682469	intron_variant,NMD_transcript_v	MODIFIER	Tcf4	ENSMUSG00000053477	Transcript	ENSMUST00000202751	nonsense_mediated_decay	rs30260846
18:69682469-69682469	intron_variant	MODIFIER	Tcf4	ENSMUSG00000053477	Transcript	ENSMUST00000202772	protein_coding	rs30260846
18:69682469-69682469	intron_variant	MODIFIER	Tcf4	ENSMUSG00000053477	Transcript	ENSMUST00000202937	protein_coding	rs30260846
18:69683864-69683865	3_prime_UTR_variant	MODIFIER	Tcf4	ENSMUSG00000053477	Transcript	ENSMUST00000066717	protein_coding	rs259200904
18:69683864-69683865	3_prime_UTR_variant	MODIFIER	Tcf4	ENSMUSG00000053477	Transcript	ENSMUST00000078486	protein_coding	rs259200904
18:69683864-69683865	3_prime_UTR_variant	MODIFIER	Tcf4	ENSMUSG00000053477	Transcript	ENSMUST00000114977	protein_coding	rs259200904
18:69683864-69683865	downstream_gene_variant	MODIFIER	Tcf4	ENSMUSG00000053477	Transcript	ENSMUST00000114978	protein_coding	rs259200904
18:69683864-69683865	downstream_gene_variant	MODIFIER	Tcf4	ENSMUSG00000053477	Transcript	ENSMUST00000114980	protein_coding	rs259200904
18:69683864-69683865	3_prime_UTR_variant	MODIFIER	Tcf4	ENSMUSG00000053477	Transcript	ENSMUST00000114982	protein_coding	rs259200904
18:69683864-69683865	3_prime_UTR_variant	MODIFIER	Tcf4	ENSMUSG00000053477	Transcript	ENSMUST00000114985	protein_coding	rs259200904
18:69683864-69683865	downstream_gene_variant	MODIFIER	Tcf4	ENSMUSG00000053477	Transcript	ENSMUST00000136019	retained_intron	rs259200904

18:69683864-69683865	non_coding_transcript_exon_va	MODIFIER	Tcf4	ENSMUSG00000053477	Transcript	ENSMUST00000146908	retained_intron	rs259200904
18:69683864-69683865	downstream_gene_variant	MODIFIER	Tcf4	ENSMUSG00000053477	Transcript	ENSMUST00000201094	protein_coding	rs259200904
18:69683864-69683865	downstream_gene_variant	MODIFIER	Tcf4	ENSMUSG00000053477	Transcript	ENSMUST00000201205	protein_coding	rs259200904
18:69683864-69683865	downstream_gene_variant	MODIFIER	Tcf4	ENSMUSG00000053477	Transcript	ENSMUST00000201410	protein_coding	rs259200904
18:69683864-69683865	downstream_gene_variant	MODIFIER	Tcf4	ENSMUSG00000053477	Transcript	ENSMUST00000201631	protein_coding	rs259200904
18:69683864-69683865	downstream_gene_variant	MODIFIER	Tcf4	ENSMUSG00000053477	Transcript	ENSMUST00000201781	protein_coding	rs259200904
18:69683864-69683865	downstream_gene_variant	MODIFIER	Tcf4	ENSMUSG00000053477	Transcript	ENSMUST00000202057	protein_coding	rs259200904
18:69683864-69683865	downstream_gene_variant	MODIFIER	Tcf4	ENSMUSG00000053477	Transcript	ENSMUST00000202116	protein_coding	rs259200904
18:69683864-69683865	downstream_gene_variant	MODIFIER	Tcf4	ENSMUSG00000053477	Transcript	ENSMUST00000202354	protein_coding	rs259200904
18:69683864-69683865	downstream_gene_variant	MODIFIER	Tcf4	ENSMUSG00000053477	Transcript	ENSMUST00000202435	protein_coding	rs259200904
18:69683864-69683865	downstream_gene_variant	MODIFIER	Tcf4	ENSMUSG00000053477	Transcript	ENSMUST00000202458	protein_coding	rs259200904
18:69683864-69683865	downstream_gene_variant	MODIFIER	Tcf4	ENSMUSG00000053477	Transcript	ENSMUST00000202474	protein_coding	rs259200904
18:69683864-69683865	downstream_gene_variant	MODIFIER	Tcf4	ENSMUSG00000053477	Transcript	ENSMUST00000202477	protein_coding	rs259200904
18:69683864-69683865	downstream_gene_variant	MODIFIER	Tcf4	ENSMUSG00000053477	Transcript	ENSMUST00000202610	protein_coding	rs259200904
18:69683864-69683865	downstream_gene_variant	MODIFIER	Tcf4	ENSMUSG00000053477	Transcript	ENSMUST00000202674	protein_coding	rs259200904
18:69683864-69683865	downstream_gene_variant	MODIFIER	Tcf4	ENSMUSG00000053477	Transcript	ENSMUST00000202751	nonsense_mediated_decay	rs259200904
18:69683864-69683865	downstream_gene_variant	MODIFIER	Tcf4	ENSMUSG00000053477	Transcript	ENSMUST00000202772	protein_coding	rs259200904
18:69683864-69683865	downstream_gene_variant	MODIFIER	Tcf4	ENSMUSG00000053477	Transcript	ENSMUST00000202937	protein_coding	rs259200904
18:69684302-69684302	3_prime_UTR_variant	MODIFIER	Tcf4	ENSMUSG00000053477	Transcript	ENSMUST00000066717	protein_coding	rs52483006
18:69684302-69684302	3_prime_UTR_variant	MODIFIER	Tcf4	ENSMUSG00000053477	Transcript	ENSMUST00000078486	protein_coding	rs52483006
18:69684302-69684302	3_prime_UTR_variant	MODIFIER	Tcf4	ENSMUSG00000053477	Transcript	ENSMUST00000114977	protein_coding	rs52483006
18:69684302-69684302	downstream_gene_variant	MODIFIER	Tcf4	ENSMUSG00000053477	Transcript	ENSMUST00000114978	protein_coding	rs52483006
18:69684302-69684302	downstream_gene_variant	MODIFIER	Tcf4	ENSMUSG00000053477	Transcript	ENSMUST00000114980	protein_coding	rs52483006
18:69684302-69684302	3_prime_UTR_variant	MODIFIER	Tcf4	ENSMUSG00000053477	Transcript	ENSMUST00000114982	protein_coding	rs52483006
18:69684302-69684302	3_prime_UTR_variant	MODIFIER	Tcf4	ENSMUSG00000053477	Transcript	ENSMUST00000114985	protein_coding	rs52483006
18:69684302-69684302	downstream_gene_variant	MODIFIER	Tcf4	ENSMUSG00000053477	Transcript	ENSMUST00000136019	retained_intron	rs52483006
18:69684302-69684302	non_coding_transcript_exon_va	MODIFIER	Tcf4	ENSMUSG00000053477	Transcript	ENSMUST00000146908	retained_intron	rs52483006
18:69684302-69684302	downstream_gene_variant	MODIFIER	Tcf4	ENSMUSG00000053477	Transcript	ENSMUST00000201094	protein_coding	rs52483006
18:69684302-69684302	downstream_gene_variant	MODIFIER	Tcf4	ENSMUSG00000053477	Transcript	ENSMUST00000201205	protein_coding	rs52483006
18:69684302-69684302	downstream_gene_variant	MODIFIER	Tcf4	ENSMUSG00000053477	Transcript	ENSMUST00000201410	protein_coding	rs52483006
18:69684302-69684302	downstream_gene_variant	MODIFIER	Tcf4	ENSMUSG00000053477	Transcript	ENSMUST00000201631	protein_coding	rs52483006
18:69684302-69684302	downstream_gene_variant	MODIFIER	Tcf4	ENSMUSG00000053477	Transcript	ENSMUST00000201781	protein_coding	rs52483006
18:69684302-69684302	downstream_gene_variant	MODIFIER	Tcf4	ENSMUSG00000053477	Transcript	ENSMUST00000202057	protein_coding	rs52483006
18:69684302-69684302	downstream_gene_variant	MODIFIER	Tcf4	ENSMUSG00000053477	Transcript	ENSMUST00000202116	protein_coding	rs52483006
18:69684302-69684302	downstream_gene_variant	MODIFIER	Tcf4	ENSMUSG00000053477	Transcript	ENSMUST00000202354	protein_coding	rs52483006
18:69684302-69684302	downstream_gene_variant	MODIFIER	Tcf4	ENSMUSG00000053477	Transcript	ENSMUST00000202435	protein_coding	rs52483006
18:69684302-69684302	downstream_gene_variant	MODIFIER	Tcf4	ENSMUSG00000053477	Transcript	ENSMUST00000202458	protein_coding	rs52483006
18:69684302-69684302	downstream_gene_variant	MODIFIER	Tcf4	ENSMUSG00000053477	Transcript	ENSMUST00000202474	protein_coding	rs52483006
18:69684302-69684302	downstream_gene_variant	MODIFIER	Tcf4	ENSMUSG00000053477	Transcript	ENSMUST00000202477	protein_coding	rs52483006
18:69684302-69684302	downstream_gene_variant	MODIFIER	Tcf4	ENSMUSG00000053477	Transcript	ENSMUST00000202610	protein_coding	rs52483006
18:69684302-69684302	downstream_gene_variant	MODIFIER	Tcf4	ENSMUSG00000053477	Transcript	ENSMUST00000202674	protein_coding	rs52483006
18:69684302-69684302	downstream_gene_variant	MODIFIER	Tcf4	ENSMUSG00000053477	Transcript	ENSMUST00000202751	nonsense_mediated_decay	rs52483006
18:69684302-69684302	downstream_gene_variant	MODIFIER	Tcf4	ENSMUSG00000053477	Transcript	ENSMUST00000202772	protein_coding	rs52483006
18:69684302-69684302	downstream_gene_variant	MODIFIER	Tcf4	ENSMUSG00000053477	Transcript	ENSMUST00000202937	protein_coding	rs52483006
18:69687560-69687560	downstream_gene_variant	MODIFIER	Tcf4	ENSMUSG00000053477	Transcript	ENSMUST00000066717	protein_coding	rs30262728
18:69687560-69687560	3_prime_UTR_variant	MODIFIER	Tcf4	ENSMUSG00000053477	Transcript	ENSMUST00000078486	protein_coding	rs30262728
18:69687560-69687560	downstream_gene_variant	MODIFIER	Tcf4	ENSMUSG00000053477	Transcript	ENSMUST00000114977	protein_coding	rs30262728
18:69687560-69687560	downstream_gene_variant	MODIFIER	Tcf4	ENSMUSG00000053477	Transcript	ENSMUST00000114978	protein_coding	rs30262728
18:69687560-69687560	downstream_gene_variant	MODIFIER	Tcf4	ENSMUSG00000053477	Transcript	ENSMUST00000114980	protein_coding	rs30262728
18:69687560-69687560	downstream_gene_variant	MODIFIER	Tcf4	ENSMUSG00000053477	Transcript	ENSMUST00000114982	protein_coding	rs30262728
18:69687560-69687560	3_prime_UTR_variant	MODIFIER	Tcf4	ENSMUSG00000053477	Transcript	ENSMUST00000114985	protein_coding	rs30262728
18:69687560-69687560	downstream_gene_variant	MODIFIER	Tcf4	ENSMUSG00000053477	Transcript	ENSMUST00000136019	retained_intron	rs30262728
18:69687560-69687560	downstream_gene_variant	MODIFIER	Tcf4	ENSMUSG00000053477	Transcript	ENSMUST00000146908	retained_intron	rs30262728
18:69687560-69687560	downstream_gene_variant	MODIFIER	Tcf4	ENSMUSG00000053477	Transcript	ENSMUST00000201094	protein_coding	rs30262728
18:69687560-69687560	downstream_gene_variant	MODIFIER	Tcf4	ENSMUSG00000053477	Transcript	ENSMUST00000201205	protein_coding	rs30262728
18:69687560-69687560	downstream_gene_variant	MODIFIER	Tcf4	ENSMUSG00000053477	Transcript	ENSMUST00000201410	protein_coding	rs30262728
18:69687560-69687560	downstream_gene_variant	MODIFIER	Tcf4	ENSMUSG00000053477	Transcript	ENSMUST00000201631	protein_coding	rs30262728
18:69687560-69687560	downstream_gene_variant	MODIFIER	Tcf4	ENSMUSG00000053477	Transcript	ENSMUST00000201781	protein_coding	rs30262728
18:69687560-69687560	downstream_gene_variant	MODIFIER	Tcf4	ENSMUSG00000053477	Transcript	ENSMUST00000202057	protein_coding	rs30262728
18:69687560-69687560	downstream_gene_variant	MODIFIER	Tcf4	ENSMUSG00000053477	Transcript	ENSMUST00000202116	protein_coding	rs30262728
18:69687560-69687560	downstream_gene_variant	MODIFIER	Tcf4	ENSMUSG00000053477	Transcript	ENSMUST00000202354	protein_coding	rs30262728
18:69687560-69687560	downstream_gene_variant	MODIFIER	Tcf4	ENSMUSG00000053477	Transcript	ENSMUST00000202435	protein_coding	rs30262728
18:69687560-69687560	downstream_gene_variant	MODIFIER	Tcf4	ENSMUSG00000053477	Transcript	ENSMUST00000202458	protein_coding	rs30262728
18:69687560-69687560	downstream_gene_variant	MODIFIER	Tcf4	ENSMUSG00000053477	Transcript	ENSMUST00000202474	protein_coding	rs30262728
18:69687560-69687560	downstream_gene_variant	MODIFIER	Tcf4	ENSMUSG00000053477	Transcript	ENSMUST00000202477	protein_coding	rs30262728
18:69687560-69687560	downstream_gene_variant	MODIFIER	Tcf4	ENSMUSG00000053477	Transcript	ENSMUST00000202610	protein_coding	rs30262728
18:69687560-69687560	downstream_gene_variant	MODIFIER	Tcf4	ENSMUSG00000053477	Transcript	ENSMUST00000202674	protein_coding	rs30262728
18:69687560-69687560	downstream_gene_variant	MODIFIER	Tcf4	ENSMUSG00000053477	Transcript	ENSMUST00000202751	nonsense_mediated_decay	rs30262728
18:69687560-69687560	downstream_gene_variant	MODIFIER	Tcf4	ENSMUSG00000053477	Transcript	ENSMUST00000202772	protein_coding	rs30262728

18:69687560-69687560	downstream_gene_variant	MODIFIER	Tcf4	ENSMUSG00000053477	Transcript	ENSMUST00000202937	protein_coding	rs30262728
18:73643301-73643301	intron_variant	MODIFIER	Smad4	ENSMUSG00000024515	Transcript	ENSMUST00000025393	protein_coding	rs29561353
18:73643301-73643301	intron_variant	MODIFIER	Smad4	ENSMUSG00000024515	Transcript	ENSMUST00000114939	protein_coding	rs29561353
18:73643301-73643301	upstream_gene_variant	MODIFIER	Smad4	ENSMUSG00000024515	Transcript	ENSMUST00000131339	processed_transcript	rs29561353
18:73643301-73643301	intron_variant,non_coding_trans	MODIFIER	Smad4	ENSMUSG00000024515	Transcript	ENSMUST00000142672	processed_transcript	rs29561353
18:73643301-73643301	intron_variant,non_coding_trans	MODIFIER	Smad4	ENSMUSG00000024515	Transcript	ENSMUST00000147315	processed_transcript	rs29561353
18:73643301-73643301	regulatory_region_variant	MODIFIER	-	-	RegulatoryFeature	ENSMUSR00000609403	TF_binding_site	rs29561353
18:73735843-73735843	3_prime_UTR_variant	MODIFIER	Elac1	ENSMUSG00000036941	Transcript	ENSMUST00000041138	protein_coding	rs37686795
18:73736329-73736329	3_prime_UTR_variant	MODIFIER	Elac1	ENSMUSG00000036941	Transcript	ENSMUST00000041138	protein_coding	-
18:73739359-73739359	intron_variant	MODIFIER	Elac1	ENSMUSG00000036941	Transcript	ENSMUST00000041138	protein_coding	rs29731576
18:73740753-73740753	intron_variant	MODIFIER	Elac1	ENSMUSG00000036941	Transcript	ENSMUST00000041138	protein_coding	rs235731950
18:73769700-73769701	downstream_gene_variant	MODIFIER	Me2	ENSMUSG00000024556	Transcript	ENSMUST00000025439	protein_coding	-
18:73812072-73812072	intron_variant	MODIFIER	Me2	ENSMUSG00000024556	Transcript	ENSMUST00000025439	protein_coding	rs48175534
18:74201291-74201291	intron_variant	MODIFIER	Ska1	ENSMUSG00000036223	Transcript	ENSMUST00000040188	protein_coding	rs47310168
18:74201291-74201291	intron_variant	MODIFIER	Ska1	ENSMUSG00000036223	Transcript	ENSMUST00000177604	protein_coding	rs47310168
18:75000596-75000596	intron_variant	MODIFIER	Rpl17	ENSMUSG00000062328	Transcript	ENSMUST00000079716	protein_coding	rs50329905
18:75000596-75000596	upstream_gene_variant	MODIFIER	Gm23301	ENSMUSG00000064647	Transcript	ENSMUST00000082713	snoRNA	rs50329905
18:75000596-75000596	upstream_gene_variant	MODIFIER	Gm26202	ENSMUSG00000064844	Transcript	ENSMUST00000082910	snoRNA	rs50329905
18:75000596-75000596	upstream_gene_variant	MODIFIER	Snord58b	ENSMUSG00000064871	Transcript	ENSMUST00000082937	snoRNA	rs50329905
18:75000596-75000596	regulatory_region_variant	MODIFIER	-	-	RegulatoryFeature	ENSMUSR00000378446	promoter	rs50329905
18:75000637-75000637	intron_variant	MODIFIER	Rpl17	ENSMUSG00000062328	Transcript	ENSMUST00000079716	protein_coding	rs49281387
18:75000637-75000637	upstream_gene_variant	MODIFIER	Gm23301	ENSMUSG00000064647	Transcript	ENSMUST00000082713	snoRNA	rs49281387
18:75000637-75000637	upstream_gene_variant	MODIFIER	Gm26202	ENSMUSG00000064844	Transcript	ENSMUST00000082910	snoRNA	rs49281387
18:75000637-75000637	upstream_gene_variant	MODIFIER	Snord58b	ENSMUSG00000064871	Transcript	ENSMUST00000082937	snoRNA	rs49281387
18:75000637-75000637	regulatory_region_variant	MODIFIER	-	-	RegulatoryFeature	ENSMUSR00000378446	promoter	rs49281387
18:75002881-75002881	upstream_gene_variant	MODIFIER	BC031181	ENSMUSG00000036299	Transcript	ENSMUST00000040284	protein_coding	rs36477182
18:75002881-75002881	intron_variant	MODIFIER	Rpl17	ENSMUSG00000062328	Transcript	ENSMUST00000079716	protein_coding	rs36477182
18:75002881-75002881	downstream_gene_variant	MODIFIER	Gm23301	ENSMUSG00000064647	Transcript	ENSMUST00000082713	snoRNA	rs36477182
18:75002881-75002881	downstream_gene_variant	MODIFIER	Gm26202	ENSMUSG00000064844	Transcript	ENSMUST00000082910	snoRNA	rs36477182
18:75002881-75002881	downstream_gene_variant	MODIFIER	Snord58b	ENSMUSG00000064871	Transcript	ENSMUST00000082937	snoRNA	rs36477182
18:75009156-75009156	intron_variant	MODIFIER	BC031181	ENSMUSG00000036299	Transcript	ENSMUST00000040284	protein_coding	rs254618033
18:77874445-77874445	intron_variant	MODIFIER	Pstpip2	ENSMUSG00000025429	Transcript	ENSMUST00000114741	protein_coding	rs47094517
18:9848423-9848423	synonymous_variant	LOW	Colec12	ENSMUSG00000036103	Transcript	ENSMUST00000040069	protein_coding	rs51891562
18:9848456-9848456	synonymous_variant	LOW	Colec12	ENSMUSG00000036103	Transcript	ENSMUST00000040069	protein_coding	rs250421350
18:9959908-9959908	intron_variant	MODIFIER	Thoc1	ENSMUSG00000024287	Transcript	ENSMUST00000025137	protein_coding	rs29868877
18:9992291-9992291	intron_variant	MODIFIER	Thoc1	ENSMUSG00000024287	Transcript	ENSMUST00000025137	protein_coding	rs49176855
18:9992291-9992291	downstream_gene_variant	MODIFIER	Usp14	ENSMUSG00000047879	Transcript	ENSMUST00000092096	protein_coding	rs49176855
18:9992291-9992291	downstream_gene_variant	MODIFIER	Usp14	ENSMUSG00000047879	Transcript	ENSMUST00000116669	protein_coding	rs49176855
18:9992291-9992291	downstream_gene_variant	MODIFIER	Usp14	ENSMUSG00000047879	Transcript	ENSMUST00000133594	retained_intron	rs49176855
19:11426832-11426832	3_prime_UTR_variant	MODIFIER	Ms4a4c	ENSMUSG00000024675	Transcript	ENSMUST00000072729	protein_coding	-
19:11426832-11426832	downstream_gene_variant	MODIFIER	Ms4a4c	ENSMUSG00000024675	Transcript	ENSMUST00000119366	protein_coding	-
19:17221071-17221071	3_prime_UTR_variant	MODIFIER	Prune2	ENSMUSG00000039126	Transcript	ENSMUST00000087689	protein_coding	-
19:17221071-17221071	3_prime_UTR_variant	MODIFIER	Prune2	ENSMUSG00000039126	Transcript	ENSMUST00000223920	protein_coding	-
19:17221071-17221071	non_coding_transcript_exon_va	MODIFIER	Prune2	ENSMUSG00000039126	Transcript	ENSMUST00000224059	processed_transcript	-
19:17221071-17221071	downstream_gene_variant	MODIFIER	Prune2	ENSMUSG00000039126	Transcript	ENSMUST00000224117	retained_intron	-
19:17221071-17221071	3_prime_UTR_variant	MODIFIER	Prune2	ENSMUSG00000039126	Transcript	ENSMUST00000225351	protein_coding	-
19:17221071-17221071	3_prime_UTR_variant	MODIFIER	Prune2	ENSMUSG00000039126	Transcript	ENSMUST00000226052	protein_coding	-
19:17221139-17221139	3_prime_UTR_variant	MODIFIER	Prune2	ENSMUSG00000039126	Transcript	ENSMUST00000087689	protein_coding	rs45996895
19:17221139-17221139	3_prime_UTR_variant	MODIFIER	Prune2	ENSMUSG00000039126	Transcript	ENSMUST00000223920	protein_coding	rs45996895
19:17221139-17221139	non_coding_transcript_exon_va	MODIFIER	Prune2	ENSMUSG00000039126	Transcript	ENSMUST00000224059	processed_transcript	rs45996895
19:17221139-17221139	downstream_gene_variant	MODIFIER	Prune2	ENSMUSG00000039126	Transcript	ENSMUST00000224117	retained_intron	rs45996895
19:17221139-17221139	3_prime_UTR_variant	MODIFIER	Prune2	ENSMUSG00000039126	Transcript	ENSMUST00000225351	protein_coding	rs45996895
19:17221139-17221139	3_prime_UTR_variant	MODIFIER	Prune2	ENSMUSG00000039126	Transcript	ENSMUST00000226052	protein_coding	rs45996895
19:17221282-17221282	3_prime_UTR_variant	MODIFIER	Prune2	ENSMUSG00000039126	Transcript	ENSMUST00000087689	protein_coding	rs50085178
19:17221282-17221282	3_prime_UTR_variant	MODIFIER	Prune2	ENSMUSG00000039126	Transcript	ENSMUST00000223920	protein_coding	rs50085178
19:17221282-17221282	downstream_gene_variant	MODIFIER	Prune2	ENSMUSG00000039126	Transcript	ENSMUST00000224059	processed_transcript	rs50085178
19:17221282-17221282	downstream_gene_variant	MODIFIER	Prune2	ENSMUSG00000039126	Transcript	ENSMUST00000224117	retained_intron	rs50085178
19:17221282-17221282	3_prime_UTR_variant	MODIFIER	Prune2	ENSMUSG00000039126	Transcript	ENSMUST00000225351	protein_coding	rs50085178
19:17221282-17221282	3_prime_UTR_variant	MODIFIER	Prune2	ENSMUSG00000039126	Transcript	ENSMUST00000226052	protein_coding	rs50085178
19:17221840-17221840	3_prime_UTR_variant	MODIFIER	Prune2	ENSMUSG00000039126	Transcript	ENSMUST00000087689	protein_coding	rs36450760
19:17221840-17221840	downstream_gene_variant	MODIFIER	Prune2	ENSMUSG00000039126	Transcript	ENSMUST00000223920	protein_coding	rs36450760
19:17221840-17221840	downstream_gene_variant	MODIFIER	Prune2	ENSMUSG00000039126	Transcript	ENSMUST00000224059	processed_transcript	rs36450760
19:17221840-17221840	downstream_gene_variant	MODIFIER	Prune2	ENSMUSG00000039126	Transcript	ENSMUST00000224117	retained_intron	rs36450760
19:17221840-17221840	downstream_gene_variant	MODIFIER	Prune2	ENSMUSG00000039126	Transcript	ENSMUST00000225351	protein_coding	rs36450760
19:17221840-17221840	downstream_gene_variant	MODIFIER	Prune2	ENSMUSG00000039126	Transcript	ENSMUST00000226052	protein_coding	rs36450760
19:17223452-17223452	3_prime_UTR_variant	MODIFIER	Prune2	ENSMUSG00000039126	Transcript	ENSMUST00000087689	protein_coding	rs48024103
19:17223452-17223452	downstream_gene_variant	MODIFIER	Prune2	ENSMUSG00000039126	Transcript	ENSMUST00000223920	protein_coding	rs48024103
19:17223452-17223452	downstream_gene_variant	MODIFIER	Prune2	ENSMUSG00000039126	Transcript	ENSMUST00000224059	processed_transcript	rs48024103
19:17223452-17223452	downstream_gene_variant	MODIFIER	Prune2	ENSMUSG00000039126	Transcript	ENSMUST00000224117	retained_intron	rs48024103

19:17223452-17223452	downstream_gene_variant	MODIFIER	Prune2	ENSMUSG00000039126	Transcript	ENSMUST00000225351	protein_coding	rs48024103
19:17223452-17223452	downstream_gene_variant	MODIFIER	Prune2	ENSMUSG00000039126	Transcript	ENSMUST00000226052	protein_coding	rs48024103
19:17223452-17223452	regulatory_region_variant	MODIFIER	-	-	RegulatoryFeature	ENSMUSR00000613340	enhancer	rs48024103
19:18591177-18591177	intron_variant	MODIFIER	Ostf1	ENSMUSG00000024725	Transcript	ENSMUST00000025631	protein_coding	rs223867303
19:18591177-18591177	downstream_gene_variant	MODIFIER	Ostf1	ENSMUSG00000024725	Transcript	ENSMUST00000138860	retained_intron	rs223867303
19:18591177-18591177	intron_variant,non_coding_trans	MODIFIER	Ostf1	ENSMUSG00000024725	Transcript	ENSMUST00000156908	retained_intron	rs223867303
19:18591177-18591177	regulatory_region_variant	MODIFIER	-	-	RegulatoryFeature	ENSMUSR00000613493	enhancer	rs223867303
19:21408893-21408893	intron_variant	MODIFIER	Gda	ENSMUSG00000058624	Transcript	ENSMUST00000087600	protein_coding	rs30693308
19:21408893-21408893	intron_variant	MODIFIER	Gda	ENSMUSG00000058624	Transcript	ENSMUST00000121725	protein_coding	rs30693308
19:25168535-25168535	intron_variant	MODIFIER	Dock8	ENSMUSG00000052085	Transcript	ENSMUST00000025831	protein_coding	rs30446420
19:25168535-25168535	upstream_gene_variant	MODIFIER	Gm24252	ENSMUSG00000065634	Transcript	ENSMUST00000083700	snoRNA	rs30446420
19:25191955-25191955	intron_variant	MODIFIER	Dock8	ENSMUSG00000052085	Transcript	ENSMUST00000025831	protein_coding	rs31109605
19:27764914-27764914	downstream_gene_variant	MODIFIER	Rfx3	ENSMUSG00000040929	Transcript	ENSMUST00000046898	protein_coding	rs30359004
19:27764914-27764914	3_prime_UTR_variant	MODIFIER	Rfx3	ENSMUSG00000040929	Transcript	ENSMUST00000165566	protein_coding	rs30359004
19:27764914-27764914	downstream_gene_variant	MODIFIER	Rfx3	ENSMUSG00000040929	Transcript	ENSMUST00000172498	processed_transcript	rs30359004
19:27764914-27764914	downstream_gene_variant	MODIFIER	Rfx3	ENSMUSG00000040929	Transcript	ENSMUST00000172907	protein_coding	rs30359004
19:27764914-27764914	downstream_gene_variant	MODIFIER	Rfx3	ENSMUSG00000040929	Transcript	ENSMUST00000173863	nonsense_mediated_decay	rs30359004
19:27764914-27764914	downstream_gene_variant	MODIFIER	Rfx3	ENSMUSG00000040929	Transcript	ENSMUST00000174850	protein_coding	rs30359004
19:29351367-29351367	intron_variant	MODIFIER	Plgrkt	ENSMUSG00000016495	Transcript	ENSMUST00000016639	protein_coding	rs37262074
19:29351367-29351367	intron_variant	MODIFIER	Plgrkt	ENSMUSG00000016495	Transcript	ENSMUST00000126800	protein_coding	rs37262074
19:29351367-29351367	non_coding_transcript_exon_va	MODIFIER	Plgrkt	ENSMUSG00000016495	Transcript	ENSMUST00000141838	retained_intron	rs37262074
19:29351367-29351367	intron_variant	MODIFIER	Plgrkt	ENSMUSG00000016495	Transcript	ENSMUST00000143467	protein_coding	rs37262074
19:29351367-29351367	intron_variant	MODIFIER	Plgrkt	ENSMUSG00000016495	Transcript	ENSMUST00000152936	protein_coding	rs37262074
19:29351367-29351367	intron_variant	MODIFIER	Plgrkt	ENSMUSG00000016495	Transcript	ENSMUST00000155367	protein_coding	rs37262074
19:29352302-29352302	intron_variant	MODIFIER	Plgrkt	ENSMUSG00000016495	Transcript	ENSMUST00000016639	protein_coding	rs251977700
19:29352302-29352302	intron_variant	MODIFIER	Plgrkt	ENSMUSG00000016495	Transcript	ENSMUST00000126800	protein_coding	rs251977700
19:29352302-29352302	upstream_gene_variant	MODIFIER	Plgrkt	ENSMUSG00000016495	Transcript	ENSMUST00000141838	retained_intron	rs251977700
19:29352302-29352302	intron_variant	MODIFIER	Plgrkt	ENSMUSG00000016495	Transcript	ENSMUST00000143467	protein_coding	rs251977700
19:29352302-29352302	intron_variant	MODIFIER	Plgrkt	ENSMUSG00000016495	Transcript	ENSMUST00000152936	protein_coding	rs251977700
19:29352302-29352302	intron_variant	MODIFIER	Plgrkt	ENSMUSG00000016495	Transcript	ENSMUST00000155367	protein_coding	rs251977700
19:29353297-29353297	intron_variant	MODIFIER	Plgrkt	ENSMUSG00000016495	Transcript	ENSMUST00000016639	protein_coding	rs215508169
19:29353297-29353297	intron_variant	MODIFIER	Plgrkt	ENSMUSG00000016495	Transcript	ENSMUST00000126800	protein_coding	rs215508169
19:29353297-29353297	upstream_gene_variant	MODIFIER	Plgrkt	ENSMUSG00000016495	Transcript	ENSMUST00000141838	retained_intron	rs215508169
19:29353297-29353297	intron_variant	MODIFIER	Plgrkt	ENSMUSG00000016495	Transcript	ENSMUST00000143467	protein_coding	rs215508169
19:29353297-29353297	intron_variant	MODIFIER	Plgrkt	ENSMUSG00000016495	Transcript	ENSMUST00000152936	protein_coding	rs215508169
19:29353297-29353297	intron_variant	MODIFIER	Plgrkt	ENSMUSG00000016495	Transcript	ENSMUST00000155367	protein_coding	rs215508169
19:29357614-29357614	intron_variant	MODIFIER	Plgrkt	ENSMUSG00000016495	Transcript	ENSMUST00000016639	protein_coding	rs36880543
19:29357614-29357614	intron_variant	MODIFIER	Plgrkt	ENSMUSG00000016495	Transcript	ENSMUST00000126800	protein_coding	rs36880543
19:29357614-29357614	downstream_gene_variant	MODIFIER	Plgrkt	ENSMUSG00000016495	Transcript	ENSMUST00000129608	processed_transcript	rs36880543
19:29357614-29357614	downstream_gene_variant	MODIFIER	Plgrkt	ENSMUSG00000016495	Transcript	ENSMUST00000138051	protein_coding	rs36880543
19:29357614-29357614	downstream_gene_variant	MODIFIER	Plgrkt	ENSMUSG00000016495	Transcript	ENSMUST00000139860	processed_transcript	rs36880543
19:29357614-29357614	intron_variant	MODIFIER	Plgrkt	ENSMUSG00000016495	Transcript	ENSMUST00000143467	protein_coding	rs36880543
19:29357614-29357614	intron_variant	MODIFIER	Plgrkt	ENSMUSG00000016495	Transcript	ENSMUST00000152936	protein_coding	rs36880543
19:29357614-29357614	intron_variant	MODIFIER	Plgrkt	ENSMUSG00000016495	Transcript	ENSMUST00000155367	protein_coding	rs36880543
19:29383648-29383648	intron_variant	MODIFIER	Cd274	ENSMUSG00000016496	Transcript	ENSMUST00000016640	protein_coding	rs36631654
19:32014247-32014247	synonymous_variant	LOW	Asah2	ENSMUSG00000024887	Transcript	ENSMUST00000096119	protein_coding	rs46947027
19:34593600-34593600	downstream_gene_variant	MODIFIER	Ifit3	ENSMUSG00000074896	Transcript	ENSMUST00000102825	protein_coding	rs13467978
19:34593600-34593600	3_prime_UTR_variant	MODIFIER	Ifit1bl1	ENSMUSG00000079339	Transcript	ENSMUST00000112467	protein_coding	rs13467978
19:34593600-34593600	3_prime_UTR_variant	MODIFIER	Ifit1bl1	ENSMUSG00000079339	Transcript	ENSMUST00000168254	protein_coding	rs13467978
19:37986003-37986003	synonymous_variant	LOW	Myof	ENSMUSG00000048612	Transcript	ENSMUST00000041475	protein_coding	rs31283892
19:37986003-37986003	synonymous_variant	LOW	Myof	ENSMUSG00000048612	Transcript	ENSMUST00000172095	protein_coding	rs31283892
19:37986003-37986003	non_coding_transcript_exon_va	MODIFIER	Myof	ENSMUSG00000048612	Transcript	ENSMUST00000223650	processed_transcript	rs31283892
19:37986003-37986003	synonymous_variant	LOW	Myof	ENSMUSG00000048612	Transcript	ENSMUST00000226068	protein_coding	rs31283892
19:37986003-37986003	non_coding_transcript_exon_va	MODIFIER	Myof	ENSMUSG00000048612	Transcript	ENSMUST00000226084	retained_intron	rs31283892
19:38055168-38055168	upstream_gene_variant	MODIFIER	I830134H01Rik	ENSMUSG00000069554	Transcript	ENSMUST00000092265	lincRNA	rs47317165
19:38055168-38055168	intron_variant	MODIFIER	Cep55	ENSMUSG00000024989	Transcript	ENSMUST00000096096	protein_coding	rs47317165
19:38055168-38055168	intron_variant	MODIFIER	Cep55	ENSMUSG00000024989	Transcript	ENSMUST00000116506	protein_coding	rs47317165
19:38055168-38055168	5_prime_UTR_variant	MODIFIER	Cep55	ENSMUSG00000024989	Transcript	ENSMUST00000169673	protein_coding	rs47317165
19:38055168-38055168	upstream_gene_variant	MODIFIER	I830134H01Rik	ENSMUSG00000069554	Transcript	ENSMUST00000181592	lincRNA	rs47317165
19:38055168-38055168	regulatory_region_variant	MODIFIER	-	-	RegulatoryFeature	ENSMUSR00000144503	promoter	rs47317165
19:38055181-38055181	upstream_gene_variant	MODIFIER	I830134H01Rik	ENSMUSG00000069554	Transcript	ENSMUST00000092265	lincRNA	rs51891577
19:38055181-38055181	intron_variant	MODIFIER	Cep55	ENSMUSG00000024989	Transcript	ENSMUST00000096096	protein_coding	rs51891577
19:38055181-38055181	intron_variant	MODIFIER	Cep55	ENSMUSG00000024989	Transcript	ENSMUST00000116506	protein_coding	rs51891577
19:38055181-38055181	5_prime_UTR_variant	MODIFIER	Cep55	ENSMUSG00000024989	Transcript	ENSMUST00000169673	protein_coding	rs51891577
19:38055181-38055181	upstream_gene_variant	MODIFIER	I830134H01Rik	ENSMUSG00000069554	Transcript	ENSMUST00000181592	lincRNA	rs51891577
19:38055181-38055181	regulatory_region_variant	MODIFIER	-	-	RegulatoryFeature	ENSMUSR00000144503	promoter	rs51891577
19:38070719-38070719	upstream_gene_variant	MODIFIER	Gm23300	ENSMUSG00000064646	Transcript	ENSMUST00000082712	snoRNA	rs30513637
19:38070719-38070719	intron_variant	MODIFIER	Cep55	ENSMUSG00000024989	Transcript	ENSMUST00000096096	protein_coding	rs30513637
19:38070719-38070719	intron_variant	MODIFIER	Cep55	ENSMUSG00000024989	Transcript	ENSMUST00000116506	protein_coding	rs30513637

19:38070719-38070719	intron_variant	MODIFIER	Cep55	ENSMUSG00000024989	Transcript	ENSMUST00000169673	protein_coding	rs30513637
19:38222774-38222774	intron_variant	MODIFIER	Fra10ac1	ENSMUSG00000054237	Transcript	ENSMUST00000067167	protein_coding	rs46108007
19:38223278-38223278	intron_variant	MODIFIER	Fra10ac1	ENSMUSG00000054237	Transcript	ENSMUST00000067167	protein_coding	rs46627200
19:38223278-38223278	regulatory_region_variant	MODIFIER	-	-	RegulatoryFeature	ENSMUSR00000144528	promoter	rs46627200
19:41926471-41926471	intron_variant	MODIFIER	Exosc1	ENSMUSG00000034321	Transcript	ENSMUST00000075280	protein_coding	rs30931558
19:41926471-41926471	intron_variant	MODIFIER	Exosc1	ENSMUSG00000034321	Transcript	ENSMUST00000112123	protein_coding	rs30931558
19:41928593-41928593	upstream_gene_variant	MODIFIER	Zdhhc16	ENSMUSG00000025157	Transcript	ENSMUST00000026154	protein_coding	rs238228379
19:41928593-41928593	intron_variant	MODIFIER	Exosc1	ENSMUSG00000034321	Transcript	ENSMUST00000075280	protein_coding	rs238228379
19:41928593-41928593	intron_variant	MODIFIER	Exosc1	ENSMUSG00000034321	Transcript	ENSMUST00000112123	protein_coding	rs238228379
19:41928593-41928593	upstream_gene_variant	MODIFIER	Zdhhc16	ENSMUSG00000025157	Transcript	ENSMUST00000224258	protein_coding	rs238228379
19:41928593-41928593	upstream_gene_variant	MODIFIER	Zdhhc16	ENSMUSG00000025157	Transcript	ENSMUST00000224896	protein_coding	rs238228379
19:41929818-41929818	upstream_gene_variant	MODIFIER	Zdhhc16	ENSMUSG00000025157	Transcript	ENSMUST00000026154	protein_coding	rs36416337
19:41929818-41929818	intron_variant	MODIFIER	Exosc1	ENSMUSG00000034321	Transcript	ENSMUST00000075280	protein_coding	rs36416337
19:41929818-41929818	intron_variant	MODIFIER	Exosc1	ENSMUSG00000034321	Transcript	ENSMUST00000112123	protein_coding	rs36416337
19:41929818-41929818	upstream_gene_variant	MODIFIER	Zdhhc16	ENSMUSG00000025157	Transcript	ENSMUST00000224258	protein_coding	rs36416337
19:41929818-41929818	upstream_gene_variant	MODIFIER	Zdhhc16	ENSMUSG00000025157	Transcript	ENSMUST00000224896	protein_coding	rs36416337
19:41931440-41931440	upstream_gene_variant	MODIFIER	Zdhhc16	ENSMUSG00000025157	Transcript	ENSMUST00000026154	protein_coding	rs36570438
19:41931440-41931440	intron_variant	MODIFIER	Exosc1	ENSMUSG00000034321	Transcript	ENSMUST00000075280	protein_coding	rs36570438
19:41931440-41931440	intron_variant	MODIFIER	Exosc1	ENSMUSG00000034321	Transcript	ENSMUST00000112123	protein_coding	rs36570438
19:41931440-41931440	upstream_gene_variant	MODIFIER	Zdhhc16	ENSMUSG00000025157	Transcript	ENSMUST00000224258	protein_coding	rs36570438
19:41931440-41931440	upstream_gene_variant	MODIFIER	Zdhhc16	ENSMUSG00000025157	Transcript	ENSMUST00000224896	protein_coding	rs36570438
19:41932827-41932827	upstream_gene_variant	MODIFIER	Zdhhc16	ENSMUSG00000025157	Transcript	ENSMUST00000026154	protein_coding	rs30566072
19:41932827-41932827	intron_variant	MODIFIER	Exosc1	ENSMUSG00000034321	Transcript	ENSMUST00000075280	protein_coding	rs30566072
19:41932827-41932827	intron_variant	MODIFIER	Exosc1	ENSMUSG00000034321	Transcript	ENSMUST00000112123	protein_coding	rs30566072
19:41932827-41932827	upstream_gene_variant	MODIFIER	Zdhhc16	ENSMUSG00000025157	Transcript	ENSMUST00000224258	protein_coding	rs30566072
19:41932827-41932827	upstream_gene_variant	MODIFIER	Zdhhc16	ENSMUSG00000025157	Transcript	ENSMUST00000224562	protein_coding	rs30566072
19:41932827-41932827	upstream_gene_variant	MODIFIER	Zdhhc16	ENSMUSG00000025157	Transcript	ENSMUST00000224896	protein_coding	rs30566072
19:41932827-41932827	upstream_gene_variant	MODIFIER	Zdhhc16	ENSMUSG00000025157	Transcript	ENSMUST00000225968	nonsense_mediated_decay	rs30566072
19:41932827-41932827	regulatory_region_variant	MODIFIER	-	-	RegulatoryFeature	ENSMUSR00000145125	promoter	rs30566072
19:42013958-42013958	intron_variant	MODIFIER	Ubtd1	ENSMUSG00000025171	Transcript	ENSMUST00000026170	protein_coding	rs50162804
19:42593841-42593841	downstream_gene_variant	MODIFIER	R3hcc1l	ENSMUSG00000025184	Transcript	ENSMUST00000026188	protein_coding	rs3715545
19:42593841-42593841	downstream_gene_variant	MODIFIER	Loxl4	ENSMUSG00000025185	Transcript	ENSMUST00000026190	protein_coding	rs3715545
19:42593841-42593841	downstream_gene_variant	MODIFIER	R3hcc1l	ENSMUSG00000025184	Transcript	ENSMUST00000160107	protein_coding	rs3715545
19:42593841-42593841	downstream_gene_variant	MODIFIER	Loxl4	ENSMUSG00000025185	Transcript	ENSMUST00000164786	protein_coding	rs3715545
19:42593841-42593841	downstream_gene_variant	MODIFIER	Loxl4	ENSMUSG00000025185	Transcript	ENSMUST00000171432	protein_coding	rs3715545
19:42594458-42594458	downstream_gene_variant	MODIFIER	R3hcc1l	ENSMUSG00000025184	Transcript	ENSMUST00000026188	protein_coding	rs30598323
19:42594458-42594458	3_prime_UTR_variant	MODIFIER	Loxl4	ENSMUSG00000025185	Transcript	ENSMUST00000026190	protein_coding	rs30598323
19:42594458-42594458	downstream_gene_variant	MODIFIER	R3hcc1l	ENSMUSG00000025184	Transcript	ENSMUST00000160107	protein_coding	rs30598323
19:42594458-42594458	downstream_gene_variant	MODIFIER	Loxl4	ENSMUSG00000025185	Transcript	ENSMUST00000164786	protein_coding	rs30598323
19:42594458-42594458	downstream_gene_variant	MODIFIER	Loxl4	ENSMUSG00000025185	Transcript	ENSMUST00000171432	protein_coding	rs30598323
19:42594485-42594485	downstream_gene_variant	MODIFIER	R3hcc1l	ENSMUSG00000025184	Transcript	ENSMUST00000026188	protein_coding	rs31149344
19:42594485-42594485	3_prime_UTR_variant	MODIFIER	Loxl4	ENSMUSG00000025185	Transcript	ENSMUST00000026190	protein_coding	rs31149344
19:42594485-42594485	downstream_gene_variant	MODIFIER	R3hcc1l	ENSMUSG00000025184	Transcript	ENSMUST00000160107	protein_coding	rs31149344
19:42594485-42594485	downstream_gene_variant	MODIFIER	Loxl4	ENSMUSG00000025185	Transcript	ENSMUST00000164786	protein_coding	rs31149344
19:42594485-42594485	downstream_gene_variant	MODIFIER	Loxl4	ENSMUSG00000025185	Transcript	ENSMUST00000171432	protein_coding	rs31149344
19:42602183-42602183	synonymous_variant	LOW	Loxl4	ENSMUSG00000025185	Transcript	ENSMUST00000026190	protein_coding	rs38201618
19:42602183-42602183	non_coding_transcript_exon_va	MODIFIER	Loxl4	ENSMUSG00000025185	Transcript	ENSMUST00000164014	processed_transcript	rs38201618
19:42602183-42602183	synonymous_variant	LOW	Loxl4	ENSMUSG00000025185	Transcript	ENSMUST00000164786	protein_coding	rs38201618
19:42602183-42602183	synonymous_variant	LOW	Loxl4	ENSMUSG00000025185	Transcript	ENSMUST00000171432	protein_coding	rs38201618
19:42606704-42606704	synonymous_variant	LOW	Loxl4	ENSMUSG00000025185	Transcript	ENSMUST00000026190	protein_coding	rs3705236
19:42606704-42606704	upstream_gene_variant	MODIFIER	Loxl4	ENSMUSG00000025185	Transcript	ENSMUST00000164014	processed_transcript	rs3705236
19:42606704-42606704	synonymous_variant	LOW	Loxl4	ENSMUSG00000025185	Transcript	ENSMUST00000164786	protein_coding	rs3705236
19:42606704-42606704	downstream_gene_variant	MODIFIER	Loxl4	ENSMUSG00000025185	Transcript	ENSMUST00000166128	protein_coding	rs3705236
19:42606704-42606704	synonymous_variant	LOW	Loxl4	ENSMUSG00000025185	Transcript	ENSMUST00000171432	protein_coding	rs3705236
19:42607655-42607655	missense_variant	MODERATE	Loxl4	ENSMUSG00000025185	Transcript	ENSMUST00000026190	protein_coding	rs51280541
19:42607655-42607655	upstream_gene_variant	MODIFIER	Loxl4	ENSMUSG00000025185	Transcript	ENSMUST00000164014	processed_transcript	rs51280541
19:42607655-42607655	missense_variant	MODERATE	Loxl4	ENSMUSG00000025185	Transcript	ENSMUST00000164786	protein_coding	rs51280541
19:42607655-42607655	missense_variant	MODERATE	Loxl4	ENSMUSG00000025185	Transcript	ENSMUST00000166128	protein_coding	rs51280541
19:42607655-42607655	missense_variant	MODERATE	Loxl4	ENSMUSG00000025185	Transcript	ENSMUST00000171432	protein_coding	rs51280541
19:42608346-42608346	synonymous_variant	LOW	Loxl4	ENSMUSG00000025185	Transcript	ENSMUST00000026190	protein_coding	rs51669286
19:42608346-42608346	synonymous_variant	LOW	Loxl4	ENSMUSG00000025185	Transcript	ENSMUST00000164786	protein_coding	rs51669286
19:42608346-42608346	synonymous_variant	LOW	Loxl4	ENSMUSG00000025185	Transcript	ENSMUST00000166128	protein_coding	rs51669286
19:42608346-42608346	synonymous_variant	LOW	Loxl4	ENSMUSG00000025185	Transcript	ENSMUST00000171432	protein_coding	rs51669286
19:42608417-42608417	missense_variant	MODERATE	Loxl4	ENSMUSG00000025185	Transcript	ENSMUST00000026190	protein_coding	rs48954133
19:42608417-42608417	missense_variant	MODERATE	Loxl4	ENSMUSG00000025185	Transcript	ENSMUST00000164786	protein_coding	rs48954133
19:42608417-42608417	missense_variant	MODERATE	Loxl4	ENSMUSG00000025185	Transcript	ENSMUST00000166128	protein_coding	rs48954133
19:42608417-42608417	missense_variant	MODERATE	Loxl4	ENSMUSG00000025185	Transcript	ENSMUST00000171432	protein_coding	rs48954133
19:42608463-42608463	synonymous_variant	LOW	Loxl4	ENSMUSG00000025185	Transcript	ENSMUST00000026190	protein_coding	rs51599104
19:42608463-42608463	synonymous_variant	LOW	Loxl4	ENSMUSG00000025185	Transcript	ENSMUST00000164786	protein_coding	rs51599104

19:42608463-42608463	synonymous_variant	LOW	Loxl4	ENSMUSG00000025185	Transcript	ENSMUST00000166128	protein_coding	rs51599104
19:42608463-42608463	synonymous_variant	LOW	Loxl4	ENSMUSG00000025185	Transcript	ENSMUST00000171432	protein_coding	rs51599104
19:42737219-42737220	intron_variant	MODIFIER	Pyroxd2	ENSMUSG00000060224	Transcript	ENSMUST00000076505	protein_coding	rs254473498
19:42737321-42737323	intron_variant	MODIFIER	Pyroxd2	ENSMUSG00000060224	Transcript	ENSMUST00000076505	protein_coding	rs231220175
19:42737392-42737392	intron_variant	MODIFIER	Pyroxd2	ENSMUSG00000060224	Transcript	ENSMUST00000076505	protein_coding	rs52120205
19:42737485-42737486	intron_variant	MODIFIER	Pyroxd2	ENSMUSG00000060224	Transcript	ENSMUST00000076505	protein_coding	-
19:42737487-42737488	intron_variant	MODIFIER	Pyroxd2	ENSMUSG00000060224	Transcript	ENSMUST00000076505	protein_coding	-
19:42748073-42748073	intron_variant	MODIFIER	Pyroxd2	ENSMUSG00000060224	Transcript	ENSMUST00000076505	protein_coding	rs50698867
19:42748182-42748182	intron_variant	MODIFIER	Pyroxd2	ENSMUSG00000060224	Transcript	ENSMUST00000076505	protein_coding	rs49357333
19:42748187-42748187	intron_variant	MODIFIER	Pyroxd2	ENSMUSG00000060224	Transcript	ENSMUST00000076505	protein_coding	rs47050691
19:42749079-42749079	intron_variant	MODIFIER	Pyroxd2	ENSMUSG00000060224	Transcript	ENSMUST00000076505	protein_coding	rs36719178
19:42749506-42749506	intron_variant	MODIFIER	Pyroxd2	ENSMUSG00000060224	Transcript	ENSMUST00000076505	protein_coding	rs38708442
19:42749532-42749532	intron_variant	MODIFIER	Pyroxd2	ENSMUSG00000060224	Transcript	ENSMUST00000076505	protein_coding	rs38455178
19:42749562-42749562	intron_variant	MODIFIER	Pyroxd2	ENSMUSG00000060224	Transcript	ENSMUST00000076505	protein_coding	rs36624216
19:42749608-42749608	intron_variant	MODIFIER	Pyroxd2	ENSMUSG00000060224	Transcript	ENSMUST00000076505	protein_coding	rs48833767
19:42750161-42750161	intron_variant	MODIFIER	Pyroxd2	ENSMUSG00000060224	Transcript	ENSMUST00000076505	protein_coding	rs36950602
19:42750161-42750161	downstream_gene_variant	MODIFIER	Hps1	ENSMUSG00000025188	Transcript	ENSMUST00000162004	protein_coding	rs36950602
19:42752591-42752591	downstream_gene_variant	MODIFIER	Hps1	ENSMUSG00000025188	Transcript	ENSMUST00000026194	protein_coding	rs37395859
19:42752591-42752591	5_prime_UTR_variant	MODIFIER	Pyroxd2	ENSMUSG00000060224	Transcript	ENSMUST00000076505	protein_coding	rs37395859
19:42752591-42752591	downstream_gene_variant	MODIFIER	Hps1	ENSMUSG00000025188	Transcript	ENSMUST00000159974	retained_intron	rs37395859
19:42752591-42752591	downstream_gene_variant	MODIFIER	Hps1	ENSMUSG00000025188	Transcript	ENSMUST00000160455	protein_coding	rs37395859
19:42752591-42752591	downstream_gene_variant	MODIFIER	Hps1	ENSMUSG00000025188	Transcript	ENSMUST00000161252	nonsense_mediated_decay	rs37395859
19:42752591-42752591	downstream_gene_variant	MODIFIER	Hps1	ENSMUSG00000025188	Transcript	ENSMUST00000162004	protein_coding	rs37395859
19:42752591-42752591	regulatory_region_variant	MODIFIER	-	-	RegulatoryFeature	ENSMUSR00000145293	promoter	rs37395859
19:42758175-42758175	intron_variant	MODIFIER	Hps1	ENSMUSG00000025188	Transcript	ENSMUST00000026194	protein_coding	rs45819372
19:42758175-42758175	3_prime_UTR_variant	MODIFIER	Hps1	ENSMUSG00000025188	Transcript	ENSMUST00000069298	protein_coding	rs45819372
19:42758175-42758175	intron_variant,non_coding_trans	MODIFIER	Hps1	ENSMUSG00000025188	Transcript	ENSMUST00000159974	retained_intron	rs45819372
19:42758175-42758175	intron_variant	MODIFIER	Hps1	ENSMUSG00000025188	Transcript	ENSMUST00000160455	protein_coding	rs45819372
19:42758175-42758175	downstream_gene_variant	MODIFIER	Hps1	ENSMUSG00000025188	Transcript	ENSMUST00000160621	retained_intron	rs45819372
19:42758175-42758175	upstream_gene_variant	MODIFIER	Hps1	ENSMUSG00000025188	Transcript	ENSMUST00000161252	nonsense_mediated_decay	rs45819372
19:42758175-42758175	intron_variant	MODIFIER	Hps1	ENSMUSG00000025188	Transcript	ENSMUST00000162004	protein_coding	rs45819372
19:42758175-42758175	downstream_gene_variant	MODIFIER	Hps1	ENSMUSG00000025188	Transcript	ENSMUST00000162061	protein_coding	rs45819372
19:42758196-42758196	intron_variant	MODIFIER	Hps1	ENSMUSG00000025188	Transcript	ENSMUST00000026194	protein_coding	rs46320958
19:42758196-42758196	3_prime_UTR_variant	MODIFIER	Hps1	ENSMUSG00000025188	Transcript	ENSMUST00000069298	protein_coding	rs46320958
19:42758196-42758196	intron_variant,non_coding_trans	MODIFIER	Hps1	ENSMUSG00000025188	Transcript	ENSMUST00000159974	retained_intron	rs46320958
19:42758196-42758196	intron_variant	MODIFIER	Hps1	ENSMUSG00000025188	Transcript	ENSMUST00000160455	protein_coding	rs46320958
19:42758196-42758196	downstream_gene_variant	MODIFIER	Hps1	ENSMUSG00000025188	Transcript	ENSMUST00000160621	retained_intron	rs46320958
19:42758196-42758196	upstream_gene_variant	MODIFIER	Hps1	ENSMUSG00000025188	Transcript	ENSMUST00000161252	nonsense_mediated_decay	rs46320958
19:42758196-42758196	intron_variant	MODIFIER	Hps1	ENSMUSG00000025188	Transcript	ENSMUST00000162004	protein_coding	rs46320958
19:42758196-42758196	downstream_gene_variant	MODIFIER	Hps1	ENSMUSG00000025188	Transcript	ENSMUST00000162061	protein_coding	rs46320958
19:42758663-42758663	intron_variant	MODIFIER	Hps1	ENSMUSG00000025188	Transcript	ENSMUST00000026194	protein_coding	rs365436344
19:42758663-42758663	3_prime_UTR_variant	MODIFIER	Hps1	ENSMUSG00000025188	Transcript	ENSMUST00000069298	protein_coding	rs365436344
19:42758663-42758663	intron_variant,non_coding_trans	MODIFIER	Hps1	ENSMUSG00000025188	Transcript	ENSMUST00000159974	retained_intron	rs365436344
19:42758663-42758663	intron_variant	MODIFIER	Hps1	ENSMUSG00000025188	Transcript	ENSMUST00000160455	protein_coding	rs365436344
19:42758663-42758663	downstream_gene_variant	MODIFIER	Hps1	ENSMUSG00000025188	Transcript	ENSMUST00000160621	retained_intron	rs365436344
19:42758663-42758663	upstream_gene_variant	MODIFIER	Hps1	ENSMUSG00000025188	Transcript	ENSMUST00000161252	nonsense_mediated_decay	rs365436344
19:42758663-42758663	intron_variant	MODIFIER	Hps1	ENSMUSG00000025188	Transcript	ENSMUST00000162004	protein_coding	rs365436344
19:42758663-42758663	3_prime_UTR_variant	MODIFIER	Hps1	ENSMUSG00000025188	Transcript	ENSMUST00000162061	protein_coding	rs365436344
19:42759162-42759162	intron_variant	MODIFIER	Hps1	ENSMUSG00000025188	Transcript	ENSMUST00000026194	protein_coding	rs36752388
19:42759162-42759162	3_prime_UTR_variant	MODIFIER	Hps1	ENSMUSG00000025188	Transcript	ENSMUST00000069298	protein_coding	rs36752388
19:42759162-42759162	intron_variant,non_coding_trans	MODIFIER	Hps1	ENSMUSG00000025188	Transcript	ENSMUST00000159974	retained_intron	rs36752388
19:42759162-42759162	intron_variant	MODIFIER	Hps1	ENSMUSG00000025188	Transcript	ENSMUST00000160455	protein_coding	rs36752388
19:42759162-42759162	downstream_gene_variant	MODIFIER	Hps1	ENSMUSG00000025188	Transcript	ENSMUST00000160621	retained_intron	rs36752388
19:42759162-42759162	upstream_gene_variant	MODIFIER	Hps1	ENSMUSG00000025188	Transcript	ENSMUST00000161252	nonsense_mediated_decay	rs36752388
19:42759162-42759162	intron_variant	MODIFIER	Hps1	ENSMUSG00000025188	Transcript	ENSMUST00000162004	protein_coding	rs36752388
19:42759162-42759162	3_prime_UTR_variant	MODIFIER	Hps1	ENSMUSG00000025188	Transcript	ENSMUST00000162061	protein_coding	rs36752388
19:42759200-42759200	intron_variant	MODIFIER	Hps1	ENSMUSG00000025188	Transcript	ENSMUST00000026194	protein_coding	rs36373442
19:42759200-42759200	synonymous_variant	LOW	Hps1	ENSMUSG00000025188	Transcript	ENSMUST00000069298	protein_coding	rs36373442
19:42759200-42759200	intron_variant,non_coding_trans	MODIFIER	Hps1	ENSMUSG00000025188	Transcript	ENSMUST00000159974	retained_intron	rs36373442
19:42759200-42759200	intron_variant	MODIFIER	Hps1	ENSMUSG00000025188	Transcript	ENSMUST00000160455	protein_coding	rs36373442
19:42759200-42759200	downstream_gene_variant	MODIFIER	Hps1	ENSMUSG00000025188	Transcript	ENSMUST00000160621	retained_intron	rs36373442
19:42759200-42759200	upstream_gene_variant	MODIFIER	Hps1	ENSMUSG00000025188	Transcript	ENSMUST00000161252	nonsense_mediated_decay	rs36373442
19:42759200-42759200	intron_variant	MODIFIER	Hps1	ENSMUSG00000025188	Transcript	ENSMUST00000162004	protein_coding	rs36373442
19:42759200-42759200	synonymous_variant	LOW	Hps1	ENSMUSG00000025188	Transcript	ENSMUST00000162061	protein_coding	rs36373442
19:42766725-42766728	inframe_deletion	MODERATE	Hps1	ENSMUSG00000025188	Transcript	ENSMUST00000026194	protein_coding	rs241325659
19:42766725-42766728	inframe_deletion	MODERATE	Hps1	ENSMUSG00000025188	Transcript	ENSMUST00000069298	protein_coding	rs241325659
19:42766725-42766728	non_coding_transcript_exon_va	MODIFIER	Hps1	ENSMUSG00000025188	Transcript	ENSMUST00000159974	retained_intron	rs241325659
19:42766725-42766728	inframe_deletion	MODERATE	Hps1	ENSMUSG00000025188	Transcript	ENSMUST00000160455	protein_coding	rs241325659
19:42766725-42766728	upstream_gene_variant	MODIFIER	Hps1	ENSMUSG00000025188	Transcript	ENSMUST00000160621	retained_intron	rs241325659

19:42766725-42766728	downstream_gene_variant	MODIFIER	Hps1	ENSMUSG00000025188	Transcript	ENSMUST00000161761	retained_intron	rs241325659
19:42766725-42766728	inframe_deletion	MODERATE	Hps1	ENSMUSG00000025188	Transcript	ENSMUST00000162004	protein_coding	rs241325659
19:42766725-42766728	inframe_deletion	MODERATE	Hps1	ENSMUSG00000025188	Transcript	ENSMUST00000162061	protein_coding	rs241325659
19:43505812-43505812	intron_variant	MODIFIER	Got1	ENSMUSG00000025190	Transcript	ENSMUST00000026196	protein_coding	rs30759675
19:43505812-43505812	upstream_gene_variant	MODIFIER	Got1	ENSMUSG00000025190	Transcript	ENSMUST00000132504	retained_intron	rs30759675
19:43505812-43505812	intron_variant,NMD_transcript_v	MODIFIER	Got1	ENSMUSG00000025190	Transcript	ENSMUST00000133325	nonsense_mediated_decay	rs30759675
19:43505812-43505812	regulatory_region_variant	MODIFIER	-	-	RegulatoryFeature	ENSMUSR00000616731	open_chromatin_region	rs30759675
19:43836746-43836746	synonymous_variant	LOW	Abcc2	ENSMUSG00000025194	Transcript	ENSMUST00000026208	protein_coding	rs8238775
19:43836746-43836746	upstream_gene_variant	MODIFIER	Abcc2	ENSMUSG00000025194	Transcript	ENSMUST00000099413	protein_coding	rs8238775
19:43838137-43838137	3_prime_UTR_variant	MODIFIER	Abcc2	ENSMUSG00000025194	Transcript	ENSMUST00000026208	protein_coding	rs37152801
19:43838137-43838137	upstream_gene_variant	MODIFIER	Abcc2	ENSMUSG00000025194	Transcript	ENSMUST00000099413	protein_coding	rs37152801
19:44069465-44069465	downstream_gene_variant	MODIFIER	Chuk	ENSMUSG00000025199	Transcript	ENSMUST00000026217	protein_coding	rs30359070
19:44069465-44069465	intron_variant	MODIFIER	Erlin1	ENSMUSG00000025198	Transcript	ENSMUST00000071698	protein_coding	rs30359070
19:44069465-44069465	intron_variant	MODIFIER	Erlin1	ENSMUSG00000025198	Transcript	ENSMUST00000112028	protein_coding	rs30359070
19:44069465-44069465	downstream_gene_variant	MODIFIER	Chuk	ENSMUSG00000025199	Transcript	ENSMUST00000119591	protein_coding	rs30359070
19:44069465-44069465	downstream_gene_variant	MODIFIER	Chuk	ENSMUSG00000025199	Transcript	ENSMUST00000149091	retained_intron	rs30359070
19:44069465-44069465	intron_variant,non_coding_trans	MODIFIER	Erlin1	ENSMUSG00000025198	Transcript	ENSMUST00000168602	retained_intron	rs30359070
19:44069465-44069465	intron_variant	MODIFIER	Erlin1	ENSMUSG00000025198	Transcript	ENSMUST00000169092	protein_coding	rs30359070
19:44069465-44069465	non_coding_transcript_exon_var	MODIFIER	Erlin1	ENSMUSG00000025198	Transcript	ENSMUST00000170577	retained_intron	rs30359070
19:44069465-44069465	5_prime_UTR_variant	MODIFIER	Erlin1	ENSMUSG00000025198	Transcript	ENSMUST00000170801	protein_coding	rs30359070
19:44069465-44069465	upstream_gene_variant	MODIFIER	Erlin1	ENSMUSG00000025198	Transcript	ENSMUST00000171952	nonsense_mediated_decay	rs30359070
19:44069465-44069465	intron_variant	MODIFIER	Erlin1	ENSMUSG00000025198	Transcript	ENSMUST00000172041	protein_coding	rs30359070
19:44069465-44069465	upstream_gene_variant	MODIFIER	Erlin1	ENSMUSG00000025198	Transcript	ENSMUST00000172262	protein_coding	rs30359070
19:44069465-44069465	regulatory_region_variant	MODIFIER	-	-	RegulatoryFeature	ENSMUSR00000145484	promoter	rs30359070
19:44079558-44079558	intron_variant	MODIFIER	Chuk	ENSMUSG00000025199	Transcript	ENSMUST00000026217	protein_coding	rs30453151
19:44079558-44079558	intron_variant	MODIFIER	Chuk	ENSMUSG00000025199	Transcript	ENSMUST00000119591	protein_coding	rs30453151
19:44079558-44079558	upstream_gene_variant	MODIFIER	Chuk	ENSMUSG00000025199	Transcript	ENSMUST00000123806	retained_intron	rs30453151
19:44079558-44079558	intron_variant,non_coding_trans	MODIFIER	Chuk	ENSMUSG00000025199	Transcript	ENSMUST00000134495	retained_intron	rs30453151
19:44079558-44079558	upstream_gene_variant	MODIFIER	Chuk	ENSMUSG00000025199	Transcript	ENSMUST00000144286	retained_intron	rs30453151
19:44079558-44079558	downstream_gene_variant	MODIFIER	Chuk	ENSMUSG00000025199	Transcript	ENSMUST00000147423	retained_intron	rs30453151
19:44079558-44079558	upstream_gene_variant	MODIFIER	Chuk	ENSMUSG00000025199	Transcript	ENSMUST00000149091	retained_intron	rs30453151
19:44109425-44109425	upstream_gene_variant	MODIFIER	Chuk	ENSMUSG00000025199	Transcript	ENSMUST00000026217	protein_coding	rs215686473
19:44109425-44109425	3_prime_UTR_variant	MODIFIER	Cwf19l1	ENSMUSG00000025200	Transcript	ENSMUST00000026218	protein_coding	rs215686473
19:44109425-44109425	downstream_gene_variant	MODIFIER	Gm24336	ENSMUSG00000077391	Transcript	ENSMUST00000104203	snoRNA	rs215686473
19:44109425-44109425	upstream_gene_variant	MODIFIER	Chuk	ENSMUSG00000025199	Transcript	ENSMUST00000119591	protein_coding	rs215686473
19:44109425-44109425	upstream_gene_variant	MODIFIER	Chuk	ENSMUSG00000025199	Transcript	ENSMUST00000146861	retained_intron	rs215686473
19:44109425-44109425	upstream_gene_variant	MODIFIER	Chuk	ENSMUSG00000025199	Transcript	ENSMUST00000147423	retained_intron	rs215686473
19:45021411-45021411	5_prime_UTR_variant	MODIFIER	Lzts2	ENSMUSG00000035342	Transcript	ENSMUST00000039016	protein_coding	rs30420166
19:45021411-45021411	5_prime_UTR_variant	MODIFIER	Lzts2	ENSMUSG00000035342	Transcript	ENSMUST00000178087	protein_coding	rs30420166
19:45021411-45021411	5_prime_UTR_variant	MODIFIER	Lzts2	ENSMUSG00000035342	Transcript	ENSMUST00000179108	protein_coding	rs30420166
19:45055658-45055660	3_prime_UTR_variant	MODIFIER	Sfxn3	ENSMUSG00000025212	Transcript	ENSMUST00000062213	protein_coding	-
19:45055658-45055660	3_prime_UTR_variant	MODIFIER	Sfxn3	ENSMUSG00000025212	Transcript	ENSMUST00000084493	protein_coding	-
19:45055658-45055660	3_prime_UTR_variant	MODIFIER	Sfxn3	ENSMUSG00000025212	Transcript	ENSMUST00000111954	protein_coding	-
19:46360749-46360749	downstream_gene_variant	MODIFIER	Gm24610	ENSMUSG00000065204	Transcript	ENSMUST00000083270	snoRNA	rs30610370
19:46360749-46360749	intron_variant	MODIFIER	Mfsd13a	ENSMUSG00000025227	Transcript	ENSMUST00000086969	protein_coding	rs30610370
19:46360749-46360749	intron_variant	MODIFIER	Mfsd13a	ENSMUSG00000025227	Transcript	ENSMUST00000128041	protein_coding	rs30610370
19:46360749-46360749	intron_variant	MODIFIER	Mfsd13a	ENSMUSG00000025227	Transcript	ENSMUST00000128455	protein_coding	rs30610370
19:46360749-46360749	intron_variant,NMD_transcript_v	MODIFIER	Mfsd13a	ENSMUSG00000025227	Transcript	ENSMUST00000142994	nonsense_mediated_decay	rs30610370
19:46596493-46596493	3_prime_UTR_variant	MODIFIER	Sfxn2	ENSMUSG00000025036	Transcript	ENSMUST00000026011	protein_coding	rs13468955
19:46596493-46596493	upstream_gene_variant	MODIFIER	Wbp1l	ENSMUSG00000047731	Transcript	ENSMUST00000099376	protein_coding	rs13468955
19:46596493-46596493	upstream_gene_variant	MODIFIER	Wbp1l	ENSMUSG00000047731	Transcript	ENSMUST00000132202	nonsense_mediated_decay	rs13468955
19:46596493-46596493	upstream_gene_variant	MODIFIER	Wbp1l	ENSMUSG00000047731	Transcript	ENSMUST00000138302	protein_coding	rs13468955
19:46596493-46596493	upstream_gene_variant	MODIFIER	Wbp1l	ENSMUSG00000047731	Transcript	ENSMUST00000175937	processed_transcript	rs13468955
19:47113437-47113443	inframe_deletion	MODERATE	Pdcd11	ENSMUSG00000025047	Transcript	ENSMUST00000072141	protein_coding	rs242270201
19:47113437-47113443	intron_variant	MODIFIER	Calhm2	ENSMUSG00000033033	Transcript	ENSMUST00000140512	protein_coding	rs242270201
19:47128794-47128794	downstream_gene_variant	MODIFIER	Calhm2	ENSMUSG00000033033	Transcript	ENSMUST00000035822	protein_coding	rs30317883
19:47128794-47128794	intron_variant	MODIFIER	Pdcd11	ENSMUSG00000025047	Transcript	ENSMUST00000072141	protein_coding	rs30317883
19:47128794-47128794	intron_variant	MODIFIER	Calhm2	ENSMUSG00000033033	Transcript	ENSMUST00000140512	protein_coding	rs30317883
19:47129328-47129328	downstream_gene_variant	MODIFIER	Calhm2	ENSMUSG00000033033	Transcript	ENSMUST00000035822	protein_coding	rs3709676
19:47129328-47129328	intron_variant	MODIFIER	Pdcd11	ENSMUSG00000025047	Transcript	ENSMUST00000072141	protein_coding	rs3709676
19:47129328-47129328	intron_variant	MODIFIER	Calhm2	ENSMUSG00000033033	Transcript	ENSMUST00000140512	protein_coding	rs3709676
19:47129339-47129339	downstream_gene_variant	MODIFIER	Calhm2	ENSMUSG00000033033	Transcript	ENSMUST00000035822	protein_coding	rs3709692
19:47129339-47129339	intron_variant	MODIFIER	Pdcd11	ENSMUSG00000025047	Transcript	ENSMUST00000072141	protein_coding	rs3709692
19:47129339-47129339	intron_variant	MODIFIER	Calhm2	ENSMUSG00000033033	Transcript	ENSMUST00000140512	protein_coding	rs3709692
19:47860859-47860859	intron_variant,non_coding_trans	MODIFIER	Cfap43	ENSMUSG00000044948	Transcript	ENSMUST00000026048	retained_intron	rs30658931
19:47860859-47860859	intron_variant	MODIFIER	Gsto1	ENSMUSG00000025068	Transcript	ENSMUST00000026050	protein_coding	rs30658931
19:47860859-47860859	upstream_gene_variant	MODIFIER	Gsto2	ENSMUSG00000025069	Transcript	ENSMUST00000056159	protein_coding	rs30658931
19:47860859-47860859	upstream_gene_variant	MODIFIER	Gsto2	ENSMUSG00000025069	Transcript	ENSMUST00000120645	protein_coding	rs30658931
19:47860859-47860859	upstream_gene_variant	MODIFIER	Gsto1	ENSMUSG00000025068	Transcript	ENSMUST00000143694	retained_intron	rs30658931

19:47862736-47862736	intron_variant,non_coding_trans	MODIFIER	Cfap43	ENSMUSG00000044948	Transcript	ENSMUST00000026048	retained_intron	rs30913462
19:47862736-47862736	intron_variant	MODIFIER	Gsto1	ENSMUSG00000025068	Transcript	ENSMUST00000026050	protein_coding	rs30913462
19:47862736-47862736	upstream_gene_variant	MODIFIER	Gsto2	ENSMUSG00000025069	Transcript	ENSMUST00000056159	protein_coding	rs30913462
19:47862736-47862736	upstream_gene_variant	MODIFIER	Gsto2	ENSMUSG00000025069	Transcript	ENSMUST00000120645	protein_coding	rs30913462
19:47862736-47862736	upstream_gene_variant	MODIFIER	Gsto1	ENSMUSG00000025068	Transcript	ENSMUST00000143694	retained_intron	rs30913462
19:53387469-53387469	intron_variant	MODIFIER	Smndc1	ENSMUSG00000025024	Transcript	ENSMUST00000025997	protein_coding	rs31013803
19:57034895-57034895	3_prime_UTR_variant	MODIFIER	Ablim1	ENSMUSG00000025085	Transcript	ENSMUST00000079360	protein_coding	-
19:57034895-57034895	3_prime_UTR_variant	MODIFIER	Ablim1	ENSMUSG00000025085	Transcript	ENSMUST00000099294	protein_coding	-
19:57034895-57034895	3_prime_UTR_variant	MODIFIER	Ablim1	ENSMUSG00000025085	Transcript	ENSMUST00000104902	protein_coding	-
19:57034895-57034895	downstream_gene_variant	MODIFIER	Ablim1	ENSMUSG00000025085	Transcript	ENSMUST00000111526	protein_coding	-
19:57034895-57034895	downstream_gene_variant	MODIFIER	Ablim1	ENSMUSG00000025085	Transcript	ENSMUST00000111528	protein_coding	-
19:57034895-57034895	3_prime_UTR_variant	MODIFIER	Ablim1	ENSMUSG00000025085	Transcript	ENSMUST00000111529	protein_coding	-
19:57034895-57034895	downstream_gene_variant	MODIFIER	Ablim1	ENSMUSG00000025085	Transcript	ENSMUST00000111544	protein_coding	-
19:57034895-57034895	downstream_gene_variant	MODIFIER	Ablim1	ENSMUSG00000025085	Transcript	ENSMUST00000111546	protein_coding	-
19:57034895-57034895	downstream_gene_variant	MODIFIER	Ablim1	ENSMUSG00000025085	Transcript	ENSMUST00000111550	protein_coding	-
19:57034895-57034895	downstream_gene_variant	MODIFIER	Ablim1	ENSMUSG00000025085	Transcript	ENSMUST00000111555	protein_coding	-
19:57034895-57034895	downstream_gene_variant	MODIFIER	Ablim1	ENSMUSG00000025085	Transcript	ENSMUST00000111558	protein_coding	-
19:57034895-57034895	downstream_gene_variant	MODIFIER	Ablim1	ENSMUSG00000025085	Transcript	ENSMUST00000111559	protein_coding	-
19:57034895-57034895	downstream_gene_variant	MODIFIER	Ablim1	ENSMUSG00000025085	Transcript	ENSMUST00000137389	processed_transcript	-
19:57034895-57034895	downstream_gene_variant	MODIFIER	Ablim1	ENSMUSG00000025085	Transcript	ENSMUST00000156316	retained_intron	-
19:57034896-57034906	3_prime_UTR_variant	MODIFIER	Ablim1	ENSMUSG00000025085	Transcript	ENSMUST00000079360	protein_coding	-
19:57034896-57034906	3_prime_UTR_variant	MODIFIER	Ablim1	ENSMUSG00000025085	Transcript	ENSMUST00000099294	protein_coding	-
19:57034896-57034906	3_prime_UTR_variant	MODIFIER	Ablim1	ENSMUSG00000025085	Transcript	ENSMUST00000104902	protein_coding	-
19:57034896-57034906	downstream_gene_variant	MODIFIER	Ablim1	ENSMUSG00000025085	Transcript	ENSMUST00000111526	protein_coding	-
19:57034896-57034906	downstream_gene_variant	MODIFIER	Ablim1	ENSMUSG00000025085	Transcript	ENSMUST00000111528	protein_coding	-
19:57034896-57034906	3_prime_UTR_variant	MODIFIER	Ablim1	ENSMUSG00000025085	Transcript	ENSMUST00000111529	protein_coding	-
19:57034896-57034906	downstream_gene_variant	MODIFIER	Ablim1	ENSMUSG00000025085	Transcript	ENSMUST00000111544	protein_coding	-
19:57034896-57034906	downstream_gene_variant	MODIFIER	Ablim1	ENSMUSG00000025085	Transcript	ENSMUST00000111546	protein_coding	-
19:57034896-57034906	downstream_gene_variant	MODIFIER	Ablim1	ENSMUSG00000025085	Transcript	ENSMUST00000111550	protein_coding	-
19:57034896-57034906	downstream_gene_variant	MODIFIER	Ablim1	ENSMUSG00000025085	Transcript	ENSMUST00000111555	protein_coding	-
19:57034896-57034906	downstream_gene_variant	MODIFIER	Ablim1	ENSMUSG00000025085	Transcript	ENSMUST00000111558	protein_coding	-
19:57034896-57034906	downstream_gene_variant	MODIFIER	Ablim1	ENSMUSG00000025085	Transcript	ENSMUST00000111559	protein_coding	-
19:57034896-57034906	downstream_gene_variant	MODIFIER	Ablim1	ENSMUSG00000025085	Transcript	ENSMUST00000137389	processed_transcript	-
19:57034896-57034906	downstream_gene_variant	MODIFIER	Ablim1	ENSMUSG00000025085	Transcript	ENSMUST00000156316	retained_intron	-
19:59904597-59904597	3_prime_UTR_variant	MODIFIER	Rab11fip2	ENSMUSG00000040022	Transcript	ENSMUST00000051996	protein_coding	rs13463850
19:59904597-59904597	upstream_gene_variant	MODIFIER	Gm17203	ENSMUSG00000090965	Transcript	ENSMUST00000164379	lincRNA	rs13463850
19:59904597-59904597	downstream_gene_variant	MODIFIER	Rab11fip2	ENSMUSG00000040022	Transcript	ENSMUST00000170819	protein_coding	rs13463850
19:59904597-59904597	3_prime_UTR_variant	MODIFIER	Rab11fip2	ENSMUSG00000040022	Transcript	ENSMUST00000171986	protein_coding	rs13463850
19:59904597-59904597	regulatory_region_variant	MODIFIER	-	-	RegulatoryFeature	ENSMUSR00000619156	enhancer	rs13463850
19:60532827-60532827	intron_variant	MODIFIER	Cacul1	ENSMUSG00000033417	Transcript	ENSMUST00000081790	protein_coding	rs48232195
19:60532827-60532827	intron_variant	MODIFIER	Cacul1	ENSMUSG00000033417	Transcript	ENSMUST00000111460	protein_coding	rs48232195
19:60532827-60532827	intron_variant	MODIFIER	Cacul1	ENSMUSG00000033417	Transcript	ENSMUST00000166712	protein_coding	rs48232195
19:60582908-60582908	upstream_gene_variant	MODIFIER	Cacul1	ENSMUSG00000033417	Transcript	ENSMUST00000081790	protein_coding	rs30405708
19:60582908-60582908	upstream_gene_variant	MODIFIER	Cacul1	ENSMUSG00000033417	Transcript	ENSMUST00000111460	protein_coding	rs30405708
19:60582908-60582908	upstream_gene_variant	MODIFIER	Cacul1	ENSMUSG00000033417	Transcript	ENSMUST00000166712	protein_coding	rs30405708
19:60582908-60582908	downstream_gene_variant	MODIFIER	Gm25238	ENSMUSG00000092655	Transcript	ENSMUST00000174914	miRNA	rs30405708
19:60830708-60830708	intron_variant	MODIFIER	Fam45a	ENSMUSG00000024993	Transcript	ENSMUST00000025957	protein_coding	rs46518173
19:60830708-60830708	intron_variant	MODIFIER	Fam45a	ENSMUSG00000024993	Transcript	ENSMUST00000119633	protein_coding	rs46518173
19:60830708-60830708	intron_variant	MODIFIER	Fam45a	ENSMUSG00000024993	Transcript	ENSMUST00000128357	protein_coding	rs46518173
19:60830708-60830708	non_coding_transcript_exon_vai	MODIFIER	Fam45a	ENSMUSG00000024993	Transcript	ENSMUST00000142375	retained_intron	rs46518173
19:60836073-60836073	3_prime_UTR_variant	MODIFIER	Fam45a	ENSMUSG00000024993	Transcript	ENSMUST00000025957	protein_coding	rs49879947
19:60836073-60836073	downstream_gene_variant	MODIFIER	Sfxn4	ENSMUSG00000063698	Transcript	ENSMUST00000080806	nonsense_mediated_decay	rs49879947
19:60836073-60836073	3_prime_UTR_variant	MODIFIER	Fam45a	ENSMUSG00000024993	Transcript	ENSMUST00000119633	protein_coding	rs49879947
19:60836073-60836073	downstream_gene_variant	MODIFIER	Sfxn4	ENSMUSG00000063698	Transcript	ENSMUST00000124921	nonsense_mediated_decay	rs49879947
19:60836073-60836073	downstream_gene_variant	MODIFIER	Sfxn4	ENSMUSG00000063698	Transcript	ENSMUST00000135808	protein_coding	rs49879947
19:60836073-60836073	downstream_gene_variant	MODIFIER	Fam45a	ENSMUSG00000024993	Transcript	ENSMUST00000142375	retained_intron	rs49879947
19:60836074-60836074	3_prime_UTR_variant	MODIFIER	Fam45a	ENSMUSG00000024993	Transcript	ENSMUST00000025957	protein_coding	rs51979367
19:60836074-60836074	downstream_gene_variant	MODIFIER	Sfxn4	ENSMUSG00000063698	Transcript	ENSMUST00000080806	nonsense_mediated_decay	rs51979367
19:60836074-60836074	3_prime_UTR_variant	MODIFIER	Fam45a	ENSMUSG00000024993	Transcript	ENSMUST00000119633	protein_coding	rs51979367
19:60836074-60836074	downstream_gene_variant	MODIFIER	Sfxn4	ENSMUSG00000063698	Transcript	ENSMUST00000124921	nonsense_mediated_decay	rs51979367
19:60836074-60836074	downstream_gene_variant	MODIFIER	Sfxn4	ENSMUSG00000063698	Transcript	ENSMUST00000135808	protein_coding	rs51979367
19:60836074-60836074	downstream_gene_variant	MODIFIER	Fam45a	ENSMUSG00000024993	Transcript	ENSMUST00000142375	retained_intron	rs51979367
19:60865590-60865590	intron_variant	MODIFIER	Prdx3	ENSMUSG00000024997	Transcript	ENSMUST00000025961	protein_coding	rs30793214
19:60865590-60865590	upstream_gene_variant	MODIFIER	Sfxn4	ENSMUSG00000063698	Transcript	ENSMUST00000080806	nonsense_mediated_decay	rs30793214
19:60865590-60865590	upstream_gene_variant	MODIFIER	Sfxn4	ENSMUSG00000063698	Transcript	ENSMUST00000124921	nonsense_mediated_decay	rs30793214
19:60865590-60865590	upstream_gene_variant	MODIFIER	Sfxn4	ENSMUSG00000063698	Transcript	ENSMUST00000135808	protein_coding	rs30793214
19:61126674-61126674	intron_variant,NMD_transcript_v	MODIFIER	Zfp950	ENSMUSG00000074733	Transcript	ENSMUST00000122927	nonsense_mediated_decay	rs30353198
19:61126674-61126674	intron_variant,NMD_transcript_v	MODIFIER	Zfp950	ENSMUSG00000074733	Transcript	ENSMUST00000127117	nonsense_mediated_decay	rs30353198
19:61126674-61126674	intron_variant,NMD_transcript_v	MODIFIER	Zfp950	ENSMUSG00000074733	Transcript	ENSMUST00000127290	nonsense_mediated_decay	rs30353198

19:61126674-61126674	3_prime_UTR_variant	MODIFIER	Zfp950	ENSMUSG00000074733	Transcript	ENSMUST00000143264	protein_coding	rs30353198
19:61126674-61126674	downstream_gene_variant	MODIFIER	Zfp950	ENSMUSG00000074733	Transcript	ENSMUST00000148569	protein_coding	rs30353198
19:61126674-61126674	intron_variant	MODIFIER	Zfp950	ENSMUSG00000074733	Transcript	ENSMUST00000180544	protein_coding	rs30353198
19:61126674-61126674	intron_variant,NMD_transcript_v	MODIFIER	Zfp950	ENSMUSG00000074733	Transcript	ENSMUST00000205712	nonsense_mediated_decay	rs30353198
19:61126674-61126674	intron_variant	MODIFIER	Zfp950	ENSMUSG00000074733	Transcript	ENSMUST00000205854	protein_coding	rs30353198
GL456221.1:113467-113467	missense_variant	MODERATE	Csprs	ENSMUSG00000062783	Transcript	ENSMUST00000078827	protein_coding	-
JH584304.1:48591-48591	downstream_gene_variant	MODIFIER	AC149090.1	ENSMUSG00000095041	Transcript	ENSMUST00000178343	protein_coding	-
JH584304.1:48591-48591	downstream_gene_variant	MODIFIER	AC149090.1	ENSMUSG00000095041	Transcript	ENSMUST00000179505	protein_coding	-
X:152910095-152910095	intron_variant,non_coding_trans	MODIFIER	Rpl7a-ps12	ENSMUSG00000083440	Transcript	ENSMUST00000120384	processed_pseudogene	rs579355720
X:152910095-152910095	non_coding_transcript_exon_va	MODIFIER	Gm6472	ENSMUSG00000095597	Transcript	ENSMUST00000178147	processed_transcript	rs579355720
X:152910095-152910095	non_coding_transcript_exon_va	MODIFIER	Gm6472	ENSMUSG00000095597	Transcript	ENSMUST00000187973	transcribed_processed_pseudog	rs579355720
X:152910095-152910095	regulatory_region_variant	MODIFIER	-	-	RegulatoryFeature	ENSMUSR00000291676	promoter	rs579355720
X:155504004-155504004	downstream_gene_variant	MODIFIER	Gm8606	ENSMUSG00000083362	Transcript	ENSMUST00000121482	processed_pseudogene	-
X:155504008-155504008	downstream_gene_variant	MODIFIER	Gm8606	ENSMUSG00000083362	Transcript	ENSMUST00000121482	processed_pseudogene	-
X:74988109-74988109	downstream_gene_variant	MODIFIER	Gab3	ENSMUSG00000032750	Transcript	ENSMUST00000037374	protein_coding	-
X:74988109-74988109	downstream_gene_variant	MODIFIER	Gab3	ENSMUSG00000032750	Transcript	ENSMUST00000114104	protein_coding	-
X:74988109-74988109	intron_variant	MODIFIER	Gab3	ENSMUSG00000032750	Transcript	ENSMUST00000114109	protein_coding	-
X:74988109-74988109	downstream_gene_variant	MODIFIER	Gab3	ENSMUSG00000032750	Transcript	ENSMUST00000129495	processed_transcript	-
Y:1016750-1016751	intron_variant	MODIFIER	Eif2s3y	ENSMUSG00000069049	Transcript	ENSMUST00000091197	protein_coding	-
Y:1016750-1016751	downstream_gene_variant	MODIFIER	Eif2s3y	ENSMUSG00000069049	Transcript	ENSMUST00000137006	retained_intron	-
Y:1016750-1016751	downstream_gene_variant	MODIFIER	Eif2s3y	ENSMUSG00000069049	Transcript	ENSMUST00000139083	processed_transcript	-
Y:1016750-1016751	non_coding_transcript_exon_va	MODIFIER	Eif2s3y	ENSMUSG00000069049	Transcript	ENSMUST00000148961	retained_intron	-
Y:1016750-1016751	downstream_gene_variant	MODIFIER	Eif2s3y	ENSMUSG00000069049	Transcript	ENSMUST00000154556	retained_intron	-
Y:4389605-4389607	intergenic_variant	MODIFIER	-	-	-	-	-	-
